# Tunable Aromatic Sulfoxides and Sulfones as Cysteine-Targeting Warheads: Exploring the Structure–Reactivity Relationship

**DOI:** 10.1021/acs.jmedchem.5c03536

**Published:** 2026-03-09

**Authors:** Hampus Nyström, Anna P. Valaka, Hanna A. Kalesse, Liliana Håversen, Thomas Olsson, Anders Gunnarsson, Fritz Schweikart, Jan Borén, Morten Grøtli

**Affiliations:** † Department of Chemistry and Molecular Biology, 3570 University of Gothenburg, 405 30 Gothenburg, Sweden; ‡ Department of Molecular and Clinical Medicine, University of Gothenburg and Sahlgrenska University Hospital, 413 45 Gothenburg, Sweden; § Discovery Sciences, R&D Gothenburg, AstraZeneca, 431 83 Mölndal, Sweden; ∥ Pharmaceutical Sciences, Mass Spectrometry/Structural Elucidation, R&D Gothenburg, AstraZeneca, 431 83 Mölndal, Sweden

## Abstract

Covalent modalities are powerful tools in medicinal chemistry and chemical biology, enabling selective protein inhibition and functional labeling through precise tuning of warhead reactivity. We report the design and synthesis of a 48-member library of aromatic sulfoxide and sulfone warheads capable of nucleophilic aromatic substitution (S_N_Ar). Systematic variation of the aromatic core, leaving-group electronics, and sulfur oxidation state revealed structure–reactivity relationships, correlating intrinsic reactivity with structural features. Kinetic assays demonstrated chemoselectivity toward cysteine thiols, with rates primarily dictated by the aromatic scaffold. Selected warheads were incorporated into ligand-directed probes targeting Bruton’s tyrosine kinase (BTK), identifying a pyrazine-based warhead suitable for cellular applications. Molecular dynamics guided the design of the ibrutinib-derived probe **Ibr-2** with optimized warhead-Cys481 geometry. **Ibr-2** enabled potent and traceless BTK labeling in cells while preserving enzymatic activity. These findings highlight the potential of tunable S_N_Ar warheads for the development of traceless covalent probes targeting kinases with noncatalytic cysteines.

## Graphical abstract

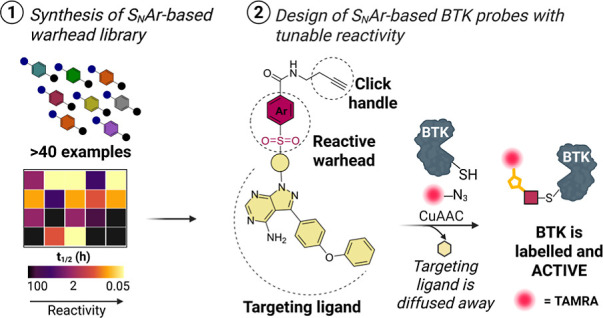

## Introduction

The development of covalent modalities has transformed medicinal chemistry and chemical biology by enabling selective inhibition and labeling of target proteins through covalent bond formation with small molecules.^1,2^ Targeted covalent inhibitors (TCIs), which engage poorly conserved, noncatalytic amino acids,^3^ have proven successful in drug discovery programs in recent years, particularly within the protein kinase field.^1^ The first covalent kinase inhibitors, afatinib and ibrutinib, targeted the epidermal growth factor receptor (EGFR) and Bruton’s tyrosine kinase (BTK), respectively. Both compounds were FDA approved in 2013, pioneering the approach of engaging noncatalytic cysteines.^1,4^ Furthermore, covalent ligands have been employed to develop chemical probes for the selective labeling and modification of proteins.^5,6^ These probes have emerged as a powerful tool for elucidating the structure, function, localization, and dynamics of proteins under study.^7^

TCIs combine a protein-binding ligand with a reactive group known as a “warhead” to form a covalent bond with a specific amino acid of the target protein.^1,8^ TCIs act through a two-step mechanism. First, a reversible binding event forms a protein–ligand complex, governed by the association (*k*_on_) and dissociation (*k*_off_) rate constants.^9^ This is followed by irreversible covalent bond formation between the electrophilic warhead and a nucleophilic amino acid. The latter step is described by the first-order rate constant *k*_inact_, which represents the maximal rate of covalent modification under saturating inhibitor concentration. The value of *k*_inact_ depends on the intrinsic reactivity of the warhead and the amino acid, and their spatial arrangement, which is dictated by the preorientation of the protein–ligand complex.^8^ TCI potency is best measured by the second-order rate constant *k*_inact_/*K*_I_ describing the efficiency of covalent modification, where *K*_I_ is the inactivation constant defined as the inhibitor concentration at which the reaction reaches half of its maximal rate (1/2 *k*_inact_).^9^ Unlike classical bioconjugation methods, which have high chemoselectivity but often lack regio-specificity, TCIs ensure site-specific protein modification.^8^ This is mainly due to the reaction being proximity-accelerated by the prior reversible binding bringing the reaction partners together. Consequently, TCIs offer the potential to develop drugs with increased potency, selectivity, and duration of action by careful optimization of both the ligand and the warhead.^2,8^

In order to be selective, it is essential that the warhead possesses a well-balanced reactivity profile.^1,10^ The intrinsic reactivity should be adequate to allow for fast covalent bond formation when reversibly bound to the target, but sufficiently low to minimize attachment to off-targets or depletion by cellular nucleophiles such as glutathione (GSH) and water. Until recent years, the intentional development of covalent drugs was largely avoided due to toxicity concerns arising from historical experiences with hepatotoxic reactive metabolites and idiosyncratic adverse effects.^3^ Contemporary drug design now emphasizes controlled electrophilicity, in which the ideal warhead for a TCI reacts only in the target-bound conformation.^11^ Additionally, for use in vivo, the warhead must be nontoxic and exhibit stability toward metabolic enzymes.^1^

Tunable electrophiles enable targeting strategies that can be optimized for proteins with different turnover rates. Proteins with low turnover rates, such as BTK (half-life 12–24 h),^12^ may benefit from warheads that are rapidly cleared following target engagement, thereby achieving kinetic selectivity by limiting time-dependent off-target reactivity.^13,14^ In contrast, targets with high turnover rates, including ITK and JAK3 (half-lives 2–3 h),^12^ benefit from more stable warheads, which allow continuous labeling of newly synthesized protein. The most successful approach in drug discovery has been to target cysteine, due to its low abundance and high nucleophilicity, using acrylamides and related α,β-unsaturated amides as warheads.^8,10,15^ Concurrently, maleimides have been extensively employed to modify cysteines in bioconjugate chemistry.^16^ However, these Michael acceptors have limitations. The cysteine adducts of maleimides are susceptible to cleavage under physiological conditions via retro-Michael reactions, thiol exchange, hydrolysis, or aminolysis,^17^ while acrylamide warheads offer limited tunability. Furthermore, cysteine residues across the proteome vary in nucleophilicity and accessibility owing to their local microenvironment, and therefore the reactivity and geometry of acrylamides may not be suitable for all targets.^10,11^ It is thus highly important to expand the medicinal chemistry toolbox with novel, tunable warheads for incorporation into TCIs and labeling probes.

Although nucleophilic aromatic substitution (S_N_Ar) reactions have been extensively applied to covalent protein modification, they have received relatively little attention in the field of TCIs.^15,18^ Recently, however, there has been a growing interest in exploring electron-deficient aromatics as cysteine-targeting warheads.^1,18^ In S_N_Ar reactions, the kinetics are enhanced by increasing the electron deficiency of the aromatic ring, achieved through the introduction of electron-withdrawing groups (EWGs) at the ortho- or para-positions, or by incorporating heteroatoms into the ring.^1^ The reaction follows a stepwise addition–elimination mechanism, and these modifications stabilize the anionic Meisenheimer intermediate, accelerating the reaction. The nucleophilic addition typically serves as the rate-determining step because it disrupts the aromaticity, making it advantageous to use strongly electron-withdrawing leaving groups to enhance reaction rates. Halides have commonly been used as leaving groups with 2-chloropyridines activated by an additional EWG (e.g., nitro groups),^19,20^ and with 2-chloropyrimidines,^21,22^ both being prevalent scaffolds.

Heteroaryl sulfoxides and sulfones have emerged as alternative leaving groups with excellent potential for fine-tuning the reactivity.^18^ Reported scaffolds include benzothiazoles,^23,24^ tetrazoles,^24–27^ 2-sulfinylpyridines,^28^ and 2-sulfonylpyrimidines^8,17,29–33^ (Figure 1a) among others.^34–40^ Sulfoxides and sulfones, with oxidation states IV and VI respectively, are strongly electron-withdrawing and possess an additional alkyl or aryl group compared to halides, which are monovalent. Although simple mesyl groups (−SO_2_CH_3_) are most commonly used, it has been shown that the reactivity can be regulated by altering the sterics,^17,28^ electronics,^17,24^ and oxidation state^24,28,34^ of the sulfur-based leaving group. These electrophiles have found vast applications as a result of their tunability, including protein bioconjugation in vitro,^36^ the development of covalent inhibitors,^29,32^ and as thiol blocking reagents for proteomic studies.^25^ Heteroaryl sulfoxides and sulfones react preferentially with cysteine over other amino acids.^17^ Unlike maleimides, they do not react with oxidized thiol functionalities, such as *S*-nitrosothiols (-SNO) or sulfenic acids (-SOH), offering enhanced chemoselectivity in vivo. Furthermore, the structural rigidity of these warheads enables precise targeting.^14^ Other advantages include synthetic accessibility^14^ and formation of cysteine adducts that are generally more stable than those of Michael acceptors.^17^ However, highly electron-deficient rings may undergo reversible thiol exchange or irreversible S_N_Ar reactions with amines,^1^ and this reactivity has been exploited for reversible covalent cysteine modifications.^41^ Despite this progress, it remains essential to acquire additional structure–reactivity data for this diverse class of electrophiles to facilitate the development of new TCIs and labeling probes.

**Figure 1:**
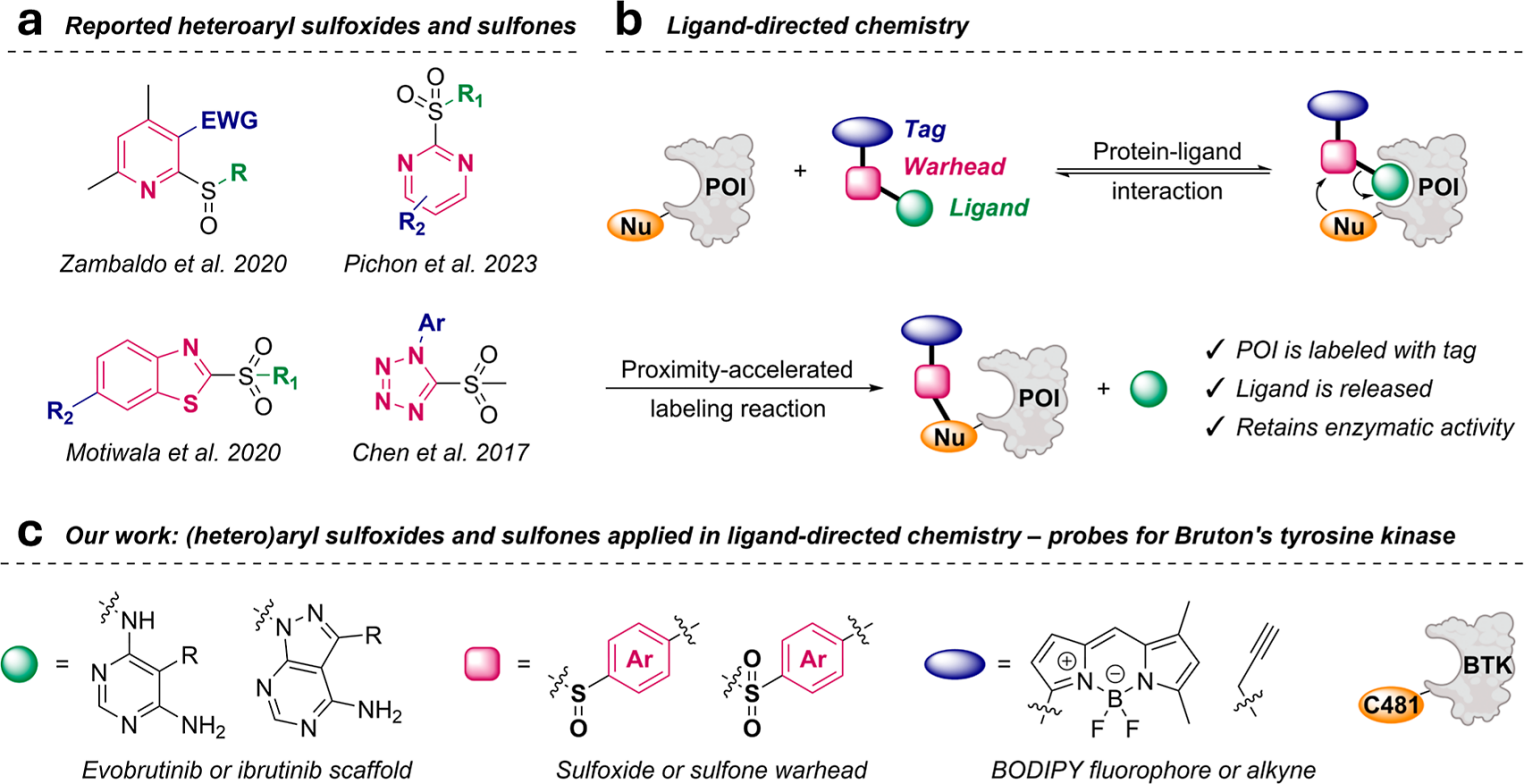
Incorporating aryl sulfoxides and sulfones as cleavable warheads into ligand-directed chemistry probes for Bruton’s tyrosine kinase (BTK). (a) Representative examples of heteroaryl sulfoxides and sulfones reported to react with cysteine through nucleophilic aromatic substitution (S_N_Ar) reactions. (b) The principles of ligand-directed chemistry. This technique can be used to selectively label a protein of interest (POI) within its native cellular environment. A targeting ligand binds reversibly to the POI and positions a cleavable warhead in proximity to a nucleophilic residue (Nu). Upon reaction, the ligand is released, resulting in a labeled protein with preserved enzymatic activity. (c) Our work involved development of labeling probes for BTK utilizing ligand-directed chemistry. The probes were comprised of the evobrutinib or ibrutinib core as a ligand, (hetero)­aryl sulfoxides or sulfones as cleavable warheads, and a BODIPY fluorophore or an alkyne handle as tags. The warhead reacts with Cys481 of BTK.

Ligand-directed (LD) chemistry further broadens the toolkit for protein labeling and functional studies by enabling selective covalent modifications of a protein of interest (POI) within its native cellular environment.^42,43^ LD chemistry employs small-molecule probes consisting of a targeting ligand and a tag (e.g., a fluorophore), linked through a cleavable warhead (Figure 1b). The ligand guides the probe to POI, where it binds reversibly and positions the warhead in proximity to a nucleophilic residue. Upon reaction, the tag is transferred to the residue, releasing the ligand and leaving the binding site vacant (Figure 1b). Labeling a residue outside the substrate-binding pocket provides a traceless approach that preserves enzymatic activity.^42^ This technique circumvents the limitations of genetic tagging methods, which can disrupt native protein expression and function, making LD chemistry ideal for endogenous systems. LD chemistry was first described by Hamachi and co-workers in the labeling of endogenous FKBP12,^44,45^ and has since expanded with the development of new cleavable warhead chemistries.^43,46^

Developing novel warheads for LD chemistry expands its potential to target challenging proteins, such as BTK. BTK, a nonreceptor tyrosine kinase of the Tec family, is predominantly expressed in hematopoietic cells, including macrophages, monocytes, and B-cells, but not T-cells.^47^ BTK plays a central role in B-cell development, differentiation, survival, and signaling, making it a validated target for treating B-cell malignancies^48^ and autoimmune diseases such as multiple sclerosis.^49,50^ Currently, six small-molecule BTK inhibitors are approved for treating hematological cancers, five of which are covalent inhibitors targeting the noncatalytic Cys481 residue near the ATP-binding pocket.^51,52^ Recently, Baud and colleagues demonstrated the potential of 2-sulfonylpyrimidines as S_N_Ar warheads for BTK inhibition.^8^ LD chemistry probes for BTK have previously been reported by London and co-workers,^7^ and by our group,^53^ using methacrylamide warheads. Although useful, these probes exhibit only moderate selectivity due to off-target binding, and it remains of great interest to develop more selective variants.

Herein, we present a structure–reactivity relationship study of sulfur-based S_N_Ar warheads, exploring functionalization of the aromatic core, the leaving-group electronics, and the sulfur oxidation state. This study includes the synthesis and characterization of a library of warhead fragments, showcasing their tunable reactivity toward cysteine. Using BTK as a clinically relevant model system, selected scaffolds were incorporated into ligand-directed BTK labeling probes (Figure 1c) and evaluated. The probes labeled recombinant BTK with intensities correlating with the cysteine reactivity assay, and a pyrazine scaffold was identified as a suitable warhead for cellular studies. Probe optimization using molecular dynamics simulations resulted in the probe **Ibr-2**, which was able to label cellular BTK with high potency and improved selectivity, while preserving BTK enzymatic activity. Finally, binding studies using surface plasmon resonance (SPR), and time-course protein mass spectrometry, were used to support the ligand-release mechanism of the probe and demonstrate complete Cys481 modification within 10 min.

## Results and Discussion

### Design and Synthesis of Model Warheads

We aimed to develop a tunable series of cysteine-reactive warheads suitable for LD chemistry probes. The warheads were designed to balance reactivity and stability while allowing straightforward conjugation to targeting ligands and tags. Aromatic sulfoxides and sulfones were chosen as electrophilic scaffolds since they undergo S_N_Ar reactions with cysteine thiols, yielding stable aryl-cysteine adducts and releasing sulfenate or sulfinate leaving groups (Figure 2).

**Figure 2:**
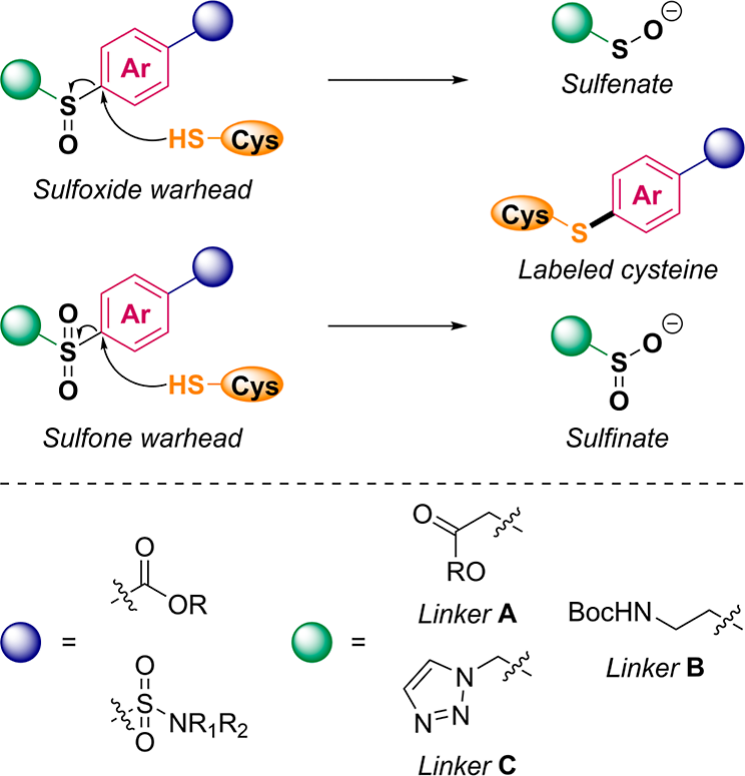
Design of cysteine-reactive warheads. Aromatic sulfoxides and sulfones were selected as cysteine-reactive electrophiles. The warhead is cleaved through a nucleophilic aromatic substitution (S_N_Ar) reaction resulting in labeling of the cysteine. To investigate the impact of electronic properties on reactivity, a series of model warheads was designed with variations in the aromatic core (red), electron-withdrawing linker (blue), leaving group (green), and sulfur oxidation state (black).

A library of model warheads was synthesized to probe structure–reactivity relationships by systematically varying (i) the aromatic core, (ii) the leaving-group electronics, and (iii) the sulfur oxidation state (Figure 2). The cores included phenyl and heteroaromatic rings (pyridine, pyrimidine, pyridazine, pyrazine, and thiazole) functionalized with EWGs including –NO_2_, –CN, and –CF_3_. All scaffolds contained either an ester or a sulfonamide substituent in the para-position, which served as electron-withdrawing groups for subsequent probe functionalization with tags (Figure 2).

To explore linker effects on reactivity, three different leaving groups were compared (Figure 2). Both ester linker **A** and Boc-protected amine linker **B** enabled attachment of the targeting ligand via amide bond formation. We hypothesized that the electron-withdrawing ester group in linker **A** placed near the sulfur atom would further accelerate the S_N_Ar reaction, whereas positioning the carbamate group further from the aromatic ring in linker **B** would yield a more electron-neutral linker. In addition, triazole linker **C** (Figure 2) was introduced to facilitate probe synthesis through click chemistry, allowing warhead and ligand to be coupled through complementary azide–alkyne handles. Finally, to investigate the influence of oxidation state on reactivity, both sulfoxide and sulfone variants were prepared for each scaffold.

The synthesis of the warhead fragments began with the preparation of thioethers using two strategies. The first method used aromatic thiols as starting materials, where three heteroaromatic thiols were synthesized on gram-scale using condensation protocols described in the literature (Schemes S1–S3).^54–57^ *S*-alkylation of the thiols using *tert*-butyl bromoacetate followed by further derivatization yielded thioethers incorporating linker **A** (Scheme S4).

Due to the limited availability of commercial thiols, a second approach to obtain thioethers in two steps employed readily accessible and inexpensive aryl halides as starting materials. First, the carboxylic acid and sulfonyl chloride functionalities were converted into their corresponding ester and sulfonamide derivatives. Subsequently, the aryl halides were displaced by thiols through S_N_Ar reactions (Figure S1). Thiolations using methyl thioglycolate or 2-(Boc-amino)­ethanethiol afforded thioethers with linkers **A** and **B**, respectively, in moderate to excellent yields (39–97%). These thiolations proceeded efficiently at room temperature in DMF. Fluoride was the preferred leaving group for the less electron-deficient phenyl rings, whereas chloride or bromide was sufficient for the heteroaromatics.

The warhead library was completed by oxidizing each thioether scaffold using mCPBA in chloroform, yielding pairs of sulfoxides and sulfones (Figure S2) in low to quantitative yields (19–100%). The oxidation to sulfoxide proceeded rapidly at 0–25 °C, with complete conversion achieved in under 2 h. In contrast, the further oxidation to sulfones was significantly slower, with reaction kinetics strongly dependent on the electron-deficiency of the substrate. Scaffolds with CF_3_ and NO_2_ substituents in the ortho-position, including **6b**, **6g**, **7a**, and **7f** (Figure S2), required heating at 50 °C and increased equivalents of mCPBA to reach completion. Selective isolation of sulfoxides was achieved by early quenching of the reaction, as the two oxidation states were readily separable by column chromatography. Full synthetic procedures and characterization by NMR and HRMS for all compounds are available in the Supporting Information.

### In Vitro Reactivity and Stability Studies

Next structure–reactivity relationships of the synthesized warhead fragments were studied (Figure 3). The intrinsic reactivity was evaluated using *N*-acetyl cysteine (NAC) as a model nucleophile in phosphate-buffered saline (PBS, pH 7.4) at 23 °C (Figure 3a). Reactions were conducted under pseudo-first-order conditions (1:50 ratio of warhead/NAC) using HPLC monitoring for determination of half-lives (*t*_1/2_). The corresponding cysteine adducts were confirmed by LC–MS. In addition, the hydrolytic stability of the warheads was assessed under identical conditions in PBS without addition of a nucleophile (Figure 3b). Kinetic plots for each compound are shown in Figures S3 and S4 with the corresponding LC–MS data shown in Figures S303–S347.

**Figure 3:**
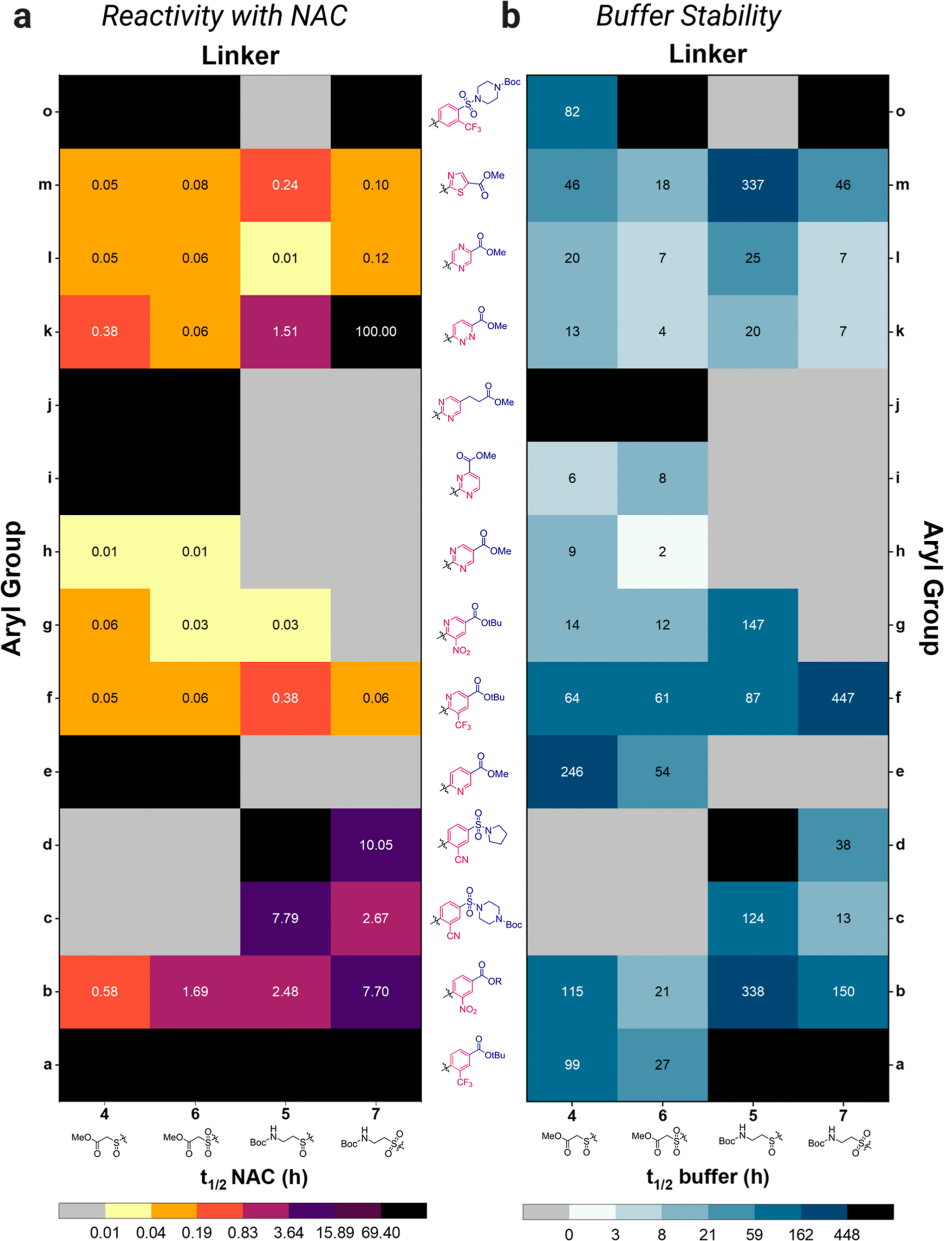
In vitro determination of electrophilic reactivity and hydrolysis resistance of the warhead fragments. (a) Heat map showing the measured *t*_1/2_ of the tested fragments (100 μM) against NAC (5 mM, 50 equiv) in PBS buffer at pH 7.4. (b) Heat map showing the measured *t*_1/2_ for the stability of the tested fragments (100 μM) in PBS buffer at pH 7.4 over a period of 7 days. Black color indicates no reaction with NAC and no decomposition in buffer respectively, while gray denotes compounds that were not synthesized. The *y*-axis represents the aryl groups **a–o**, and their structures are shown at the interference of the two heat maps. The structures of the linkers **4–7** are depicted below the *x*-axis of each heat map. The scale and coloring of the *t*_1/2_ is presented underneath each heat map.

Reactivity was strongly influenced by the electronic properties of the aromatic core. For the phenyl-based warheads (**a**-**d**, **o**), substitution at the ortho-position with strong mesomeric (-M) EWGs such as –NO_2_ (**b**) or –CN (**c**-**d**) afforded reactive scaffolds with *t*_1/2_ in the range of 0.6–10 h, whereas analogues with solely inductive (-I) –CF_3_ groups (**a**, **o**) were unreactive (Figure 3a). As predicted, the presence of the electron-withdrawing ester linker **A** (**4b**/**6b**) resulted in a 4-fold increase in reaction rate compared with the more neutral amino linker **B** (**5b**/**7b**). The most reactive oxidation state varied between scaffolds. The nitrophenyl sulfoxides (**4b**/**5b**) reacted three times faster than their corresponding sulfones (**6b**/**7b**), whereas the opposite trend was observed for aryl groups **c-d**, for which the sulfones were more reactive (Figure 3a). Although heteroaryl nitriles have been reported as cysteine-reactive warheads,^14^ no cysteine addition to nitriles was observed for any of our compounds.

Replacement of the phenyl ring with a pyridine core lacking ortho activation (**e**) was completely unreactive, but addition of a second EWG at the ortho-position, such as –CF_3_ (**f**) or –NO_2_ (**g**), drastically enhanced reactivity (Figure 3a). Nitropyridine **5g** (*t*_1/2_ = 0.03 h) was roughly 85 times more reactive than nitrophenyl **5b**, resulting in complete formation of the cysteine adduct within minutes after addition of NAC. Upon replacing the core with more electron-deficient heterocycles including pyrimidine (**h**), pyrazine **(l)**, and thiazole (**m**), comparable reactivity (*t*_1/2_ = 0.01–0.24 h) to that of the ortho-substituted pyridines was observed (Figure 3a). The role of the ester in the para-position was investigated for the highly reactive pyrimidine scaffold. Analogues where the ester was moved to the meta-position (**i**) or exchanged for a weakly electron-donating alkyl chain (**j**) resulted in complete loss of reactivity (Figure 3a). Hence the pyrimidine motif alone was not enough for cysteine conjugation. Among the diazines, the pyridazine series (**k**) displayed a broad range of half-lives, with **7k** reacting markedly slower within the set, highlighting the tunability imparted by both oxidation state and leaving-group electronics (Figure 3a). In addition, all compounds were tested for reactivity with *N*_α_-acetyl lysine and *N*-Boc serine in borate buffer (pH 8.5) at 37 °C to assess whether the S_N_Ar-reaction could occur with amine or alcohol nucleophiles. No lysine or serine adducts were detected for any compound, confirming excellent chemoselectivity toward thiols.

To compare the intrinsic reactivity of the S_N_Ar warhead library with acrylamide electrophiles, we used two common TCIs, ibrutinib and afatinib, as benchmarks in the NAC assay. The EGFR inhibitor afatinib exhibits a lower *k*_inact_ (0.9 ms^–1^)^58^ than the BTK inhibitor ibrutinib (18.4 ms^–1^).^8^ However, *k*_inact_ reflects reactivity toward a specific target residue and does not necessarily correlate with intrinsic electrophilic reactivity. Afatinib has been reported to exhibit higher inherent thiol reactivity than ibrutinib toward GSH in PBS buffer,^1,59^ a trend that was also observed with NAC (Figures S5 and S6). After 16 h of reaction, afatinib was almost completely consumed (Figure S5) whereas no NAC adduct was observed for ibrutinib (Figure S6). These results indicate that the most reactive S_N_Ar scaffolds (Figure 3a, aryl groups **f,g,h,l,m**) are far more reactive than afatinib. However, incorporation of small S_N_Ar fragments into drug-sized molecules such as TCIs is likely to reduce their reactivity due to steric effects. Finally, given that ibrutinib showed no detectable NAC reactivity, some of the nonreactive S_N_Ar scaffolds may still hold potential for TCI development when coupled to high-affinity ligands.

Hydrolytic stability exhibited an inverse relationship with electrophilicity. Sixteen out of 42 compounds decomposed within 24 h in PBS, with the nitropyridine and diazine scaffolds being most susceptible (Figure 3b). Sulfoxides were consistently more stable than their corresponding sulfones, and linker **B** improved stability relative to linker **A**. Despite the lack of NAC reactivity for pyridazine **7k** and meta-substituted pyridines **4i** and **6i**, these compounds were prone to hydrolysis (Figure 3b). LC–MS analysis indicated that hydrolysis occurred exclusively at the electron-deficient methyl ester, with no S_N_Ar displacement observed at the sulfoxide or sulfone. Linker **A** was discovered to be incompatible with ortho-cyano substituents (Scheme S5). The protons located between the ester group and the sulfur atom in cyanopyridines **4n** and **6n** were sufficiently acidic to form an enolate in buffer, which underwent intramolecular cyclization with the nitrile. This side reaction was not present in linker **B** analogues (Figures 3, 5 **c-d** and 7 **c-d**). Overall, reactivity was primarily dictated by the aromatic scaffold, with linker and oxidation state providing additional fine-tuning.

### Molecular Design of BTK Probes

To enable the use of click chemistry in probe synthesis, we investigated triazole linker **C** (Figure 2) and its impact on warhead reactivity. The nitrophenyl scaffold was selected due to its moderate reactivity that varied depending on the leaving group (*t*_1/2_ = 0.6–7.7 h) (Figure 3a, aryl group **b**). We first synthesized a sulfoxide/sulfone pair bearing a 1-substituted triazole (Scheme S6a, **13** and **14**), corresponding to an azido functionality on the warhead fragment. Upon testing, both compounds were completely unreactive with NAC (Figure S7). To determine whether the connectivity of the triazole nitrogen and the sulfur atom to the same carbon caused this inactivity, we prepared the 4-substituted triazoles **15** and **16** (Scheme S6b). Once again both compounds showed no reactivity with NAC (Figure S7). Collectively, these findings suggest that the triazole moiety perturbs the electronic properties of the warhead, likely through conjugation with the aromatic ring, rendering the warhead inactive for S_N_Ar reaction with cysteine. Consequently, triazole linkers were not pursued further.

To translate the warhead reactivity data into protein-targeting probes, selected scaffolds were incorporated into LD chemistry probes for BTK. We aimed to examine how the intrinsic warhead reactivity observed in the NAC assay correlated with protein labeling in biochemical and cellular settings. Three warheads containing linker **A** spanning distinct orders of reactivity were selected: the unreactive pyridine sulfone **6e**, the moderately reactive nitrophenyl sulfoxide **4b** (*t*_1/2_ = 0.6 h), and the highly reactive pyrazine sulfoxide **4l** (*t*_1/2_ = 0.05 h) (Figure 3a). First, the reactivity of the aforementioned warheads toward the biologically relevant thiol GSH was evaluated (Figure S8 and Table S1). The nitrophenyl derivative **4b** reacted three times faster with GSH (*t*_1/2_ = 0.2 h) than with NAC, whereas all pyrazine derivatives were consumed almost instantly (*t*_1/2_ = 0.01 h). Although unreactive toward NAC, the pyridine derivative **6e** exhibited slow reactivity with GSH (*t*_1/2_ = 40 h), as confirmed by detection of the arylated product. This behavior may be attributed to differences in the p*K*_a_ values of the thiol groups in GSH (9.2)^60^ and NAC (9.4).^61^ The greater acidity of GSH results in a higher fraction of the more reactive thiolate anion in solution.

To evaluate how the different warheads perform in a protein context, the three warheads were conjugated to the BTK inhibitor evobrutinib through amide coupling at the piperidine ring and functionalized with a BODIPY fluorophore to yield probes **Evo-3**, **Evo-4**, and **Evo-5**, respectively (Figure 4a). Although pyridine **6e** did not react with NAC in solution, we investigated whether protein binding might facilitate the reaction by positioning the warhead in proximity to Cys481. In addition, the p*K*_a_ of cysteines can vary greatly in proteins depending on the local microenvironment.^62^ Two analogous acrylamide-based probes from our previous work (**Evo-1** and **Evo-2**) were included as reference compounds (Figure 4a).^53^ **Evo-2** possesses lower inherent reactivity than **Evo-1**, due to its more sterically hindered tertiary amide.

**Figure 4:**
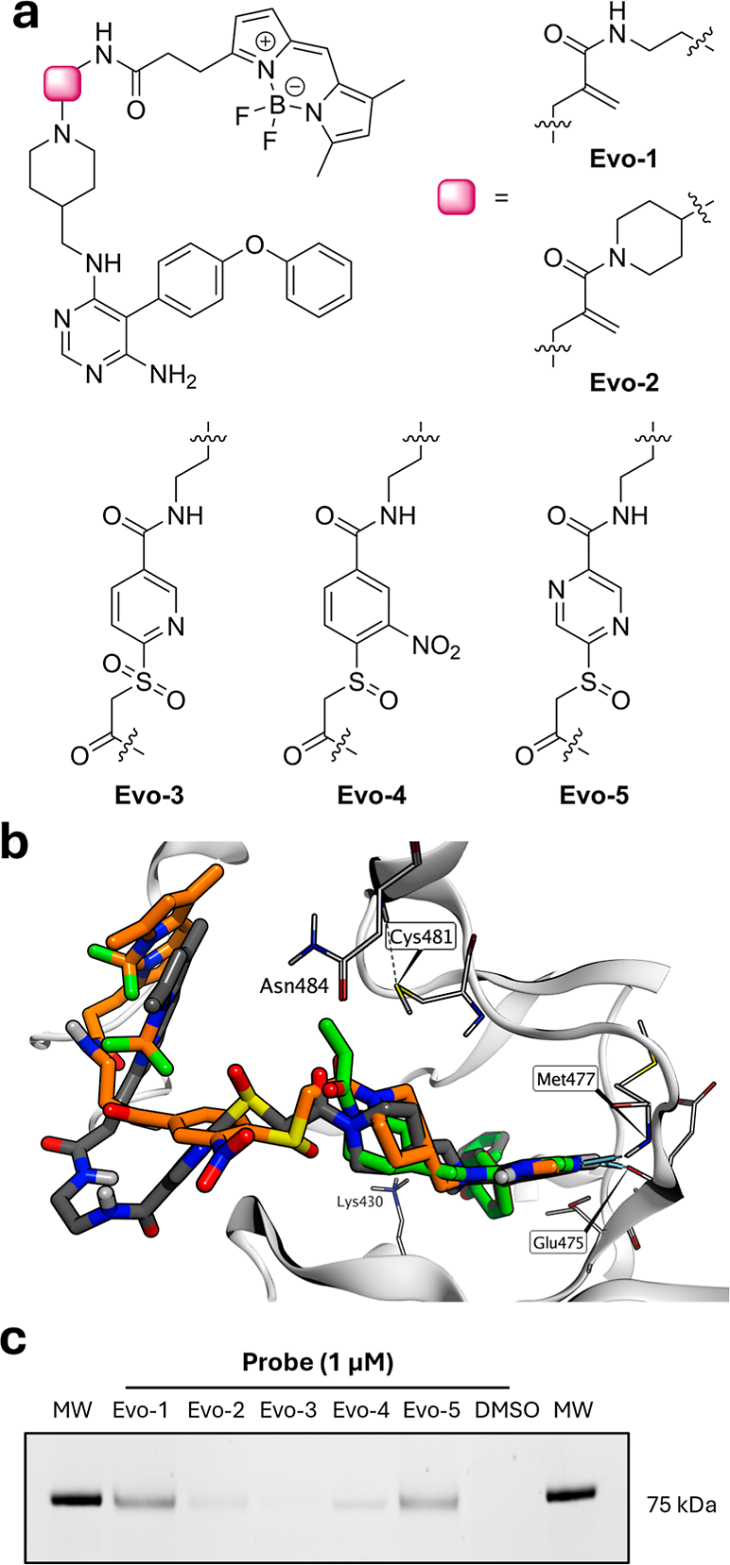
Incorporation of selected warheads into BTK probes. (a) Structures of BTK probes. Pyridine, nitrophenyl, and pyrazine warheads were incorporated into ligand-directed BTK probes using the evobrutinib scaffold and functionalized with a BODIPY fluorophore. Acrylamide probes **Evo-1** and **Evo-2** reported in previous work,^53^ were included as references. (b) Molecular docking structures of **Evo-4** (orange carbons) and **Evo-5** (gray carbons) bound to the BTK kinase domain, superimposed on the crystal structure of evobrutinib (light-green carbons, PBD ID: 6OMU). (c) Biochemical evaluation of probe reactivity toward full-length recombinant BTK using in-gel fluorescence scanning. Recombinant BTK (100 ng) was incubated with 1 μM of **Evo-1** to **Evo-5** for 1 h at room temperature and separated by SDS-PAGE. The gel was imaged with ChemiDoc imaging system (blue LED, 530/28 filter). The molecular weight (MW) of the fluorescent marker 75 kDa is shown to the right of the gel. Full gel image is presented in Figure S9.

Molecular docking using the BTK-evobrutinib crystal structure (PDB ID: 6OMU) produced binding poses for **Evo-4** and **Evo-5**, which overlapped closely with evobrutinib in the active site (Figure 4b). For both probes, the aminopyrimidine motif preserved the hinge-binding hydrogen bonds with Glu475 and Met477, which are essential for target engagement. However, the predicted distance between the reactive ipso-carbon of the warhead and Cys481 was longer compared to evobrutinib or the reference probes (Figure 4b). **Evo-3** failed to generate a meaningful docking pose.

The synthesis of the probes is outlined in Schemes S7–S9. The orthogonal methyl and *tert*-butyl ester groups of the warheads were selectively removed using NaOH and TFA, respectively, to afford the corresponding carboxylic acids. Subsequently, evobrutinib and BODIPY were installed through amide coupling reactions. For pyridine probe **Evo-3**, oxidation to the sulfone was performed early in the synthesis (Scheme S7). In contrast, for the more reactive nitrophenyl and pyrazine probes, it was crucial to perform *S*-oxidation last to avoid undesired S_N_Ar reactions of amines with the warhead (Schemes S8 and S9). **Evo-4** and **Evo-5** were isolated as sulfoxide racemates due to difficulties associated with preparing the corresponding sulfones. Attempts to further oxidize sulfoxides to sulfones using mCPBA led to N-oxidation of the pyrimidine ring of evobrutinib.

Recombinant BTK was incubated with 1 μM of each BODIPY probe, and labeling was visualized by in-gel fluorescence (Figures 4c and S9). The labeling intensities correlated with the NAC reactivity trends: **Evo-3** showed no detectable modification, **Evo-4** labeled BTK with moderate intensity comparable to **Evo-2**, and **Evo-5** exhibited the strongest labeling on par with the acrylamide reference **Evo-1**. These results demonstrate that the tunable S_N_Ar warheads can be applied to proteins in a predictable manner.

The nitrophenyl probe **Evo-4** showed poor aqueous solubility. Consequently, **Evo-5** was selected for cellular studies due to its high potency and its improved solubility. Ramos B-cells treated with **Evo-5** exhibited weak BTK labeling and extensive off-target engagement across the tested concentration range (Figures S10 and S11). This broad reactivity is consistent with the high intrinsic electrophilicity of the pyrazine scaffold, causing nonspecific cysteine arylations. The low selectivity for BTK in cells prompted further efforts to optimize probe labeling.

To enhance selectivity, we next explored modifications of the targeting ligand and the linker to promote favorable hydrogen bonding between the pyrazine warhead and Cys481. In parallel, the BODIPY fluorophore was replaced with an alkyne tag to enable chemoproteomic profiling via click chemistry. Accordingly, a series of pyrazine-based probes with alkyne tags was designed (Figure 5a). The set comprised **Evo-6**, **Ibr-1**, and **Ibr-2**, allowing systematic evaluation of how the ligand and linker affect labeling. **Evo-6** was the alkyne-tag analogue of **Evo-5**; **Ibr-1** incorporated the ibrutinib core with the same piperidine linker; and **Ibr-2** employed a shorter ethylene linker lacking the electron-withdrawing piperidine amide, expected to lower its intrinsic reactivity.

**Figure 5:**
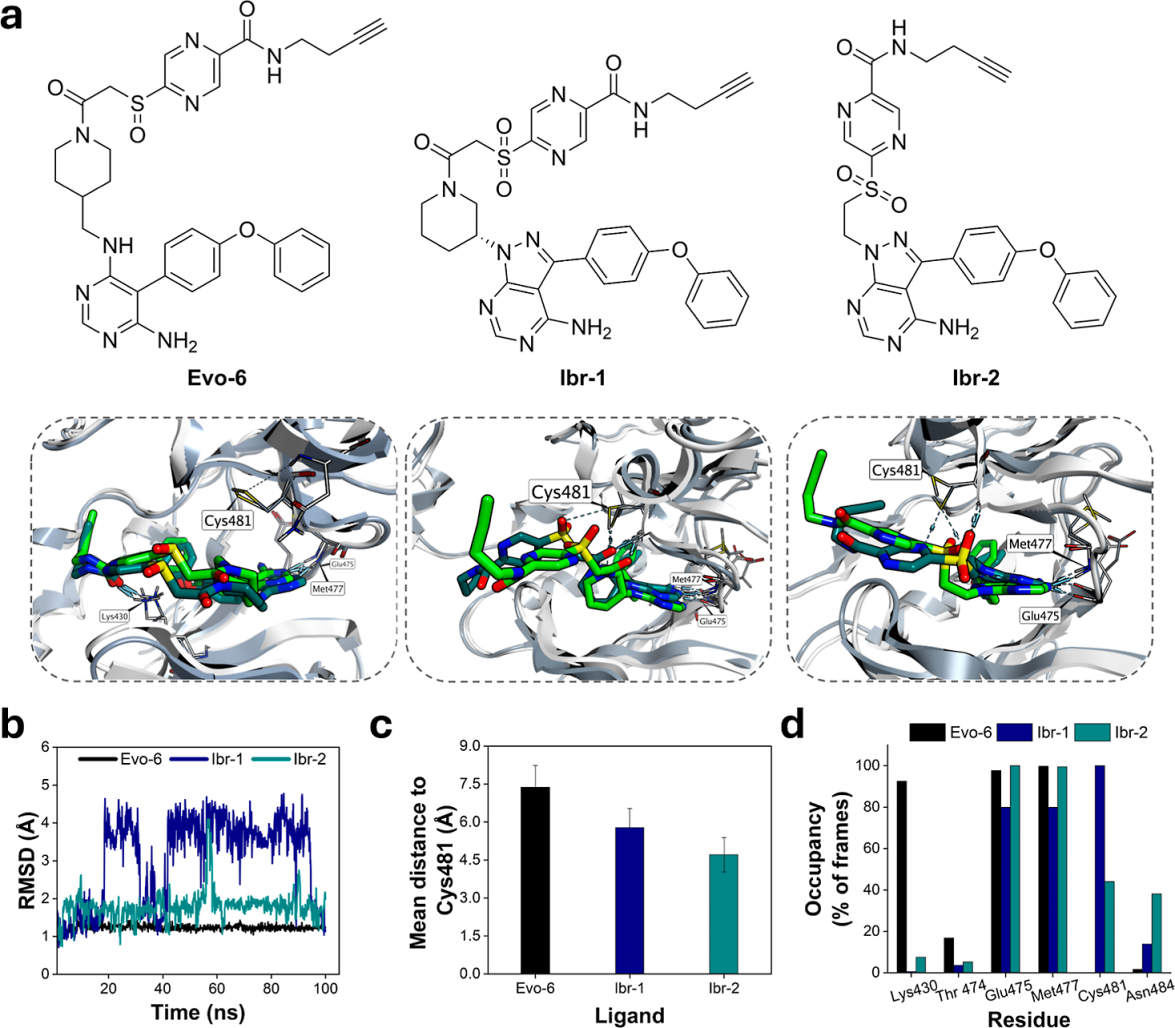
Molecular dynamics (MD) studies of BTK-probe complexes. The alkyne probes **Evo-6**, **Ibr-1**, and **Ibr-2** were designed to investigate the role of the affinity ligand and linker in BTK selectivity. (a) Most populated clusters of the MD trajectories for BTK-**Evo-6** (PDB ID: 6OMU), BTK-**Ibr-1** (PDB ID: 5P9I), and BTK-**Ibr-2** (PDB ID: 5P9I). Ligands are shown in light-green (cluster 1) and petrol (cluster 2), while proteins and side chains are shown in white and blue-gray, respectively. Key residues are labeled, and polar interactions (hydrogen bonds or ionic interactions) are indicated by dashed lines. (b) Root-mean square deviation (RMSD) plot of the three simulated systems. (c) Average distance to Cys481, measured from the ipso carbon of the warhead to the reactive sulfur atom of Cys481, throughout the entire trajectory for each system. The average distance was calculated after the system had equilibrated (after 20 ns). The error bars represent the standard deviation for the frames analyzed. (d) Hydrogen-bond occupancy analysis along the BTK active site, expressed as percentage occupancy across the simulation period.

Docking studies were next performed in Schrödinger using the crystal structures of BTK in complex with evobrutinib (PDB ID: 6OMU), ibrutinib (PDB ID: 5P9J), and a noncovalent ibrutinib analogue (PDB ID: 5P9I). All three alkyne probes maintained the key hinge-binding interactions observed for the parent inhibitor (Figure S12). Upon replacement of the evobrutinib scaffold with ibrutinib, the sulfone group formed hydrogen bonds with either the side chain (**Ibr-1**) or the backbone nitrogen (**Ibr-2**) of Cys481. In contrast, **Evo-6** lacked such interactions due to suboptimal warhead orientation (Figure S12).

As static docking does not capture conformational flexibility or solvent effects, all-atom classical molecular dynamics (MD) simulations were performed, to investigate the warhead’s positioning relative to Cys481 for an optimal reaction trajectory. 100 ns MD simulations of **Evo-6**, **Ibr-1**, and **Ibr-2** were conducted, initiated from their docked poses (Figure S12). The resulting trajectories were analyzed by root-mean-square deviation (RMSD)-based conformational clustering, and the most populated clusters for each system are shown in Figure 5a. All ligands maintained stable interactions throughout the simulations, as seen from the low RMSD plot (Figure 5b). Among the probes, **Ibr-1** showed the highest average RMSD (3.02 Å), which is probably attributed to conformational fluctuations in the solvent-exposed region rather than ligand-displacement.

Analysis of the average distance to Cys481 from the warhead’s ipso-carbon (Figure 5c), revealed that **Evo-6** consistently occupied the farthest position from the reactive cysteine, compared to the ibrutinib-based analogues. Hydrogen-bond occupancy analysis (Figure 5d) showed persistent Cys481 contacts for **Ibr-1** (≈100%) and moderate occupancy for **Ibr-2** (≈40%). This difference is attributed to a hydrogen bond interaction between the amide carbonyl of the piperidine ring of **Ibr-1** and the backbone nitrogen of Cys481 (Figure 5a), which stabilizes the ligand in an orientation that restricts the conformational flexibility of the aromatic warhead. In contrast, for **Ibr-2**, the pyrazine ring adapts a more perpendicular geometry to Cys481 (Figure 5a), favoring the S_N_Ar reaction mechanism. Additionally, analysis of the most populated **Ibr-2** clusters (Figure 5a) indicates that the pyrazine ring engages in hydrogen-bonding interactions with the thiol group of Cys481, which may promote activation of the aromatic ring toward an S_N_Ar reaction. Collectively, these predicted structural differences in combination with the distinct reactivity profile of the S_N_Ar warhead might influence the labeling efficacy of BTK.

The synthesis of **Evo-6**, **Ibr-1**, and **Ibr-2** is outlined in Schemes S10–S12. **Evo-6** was prepared as a sulfoxide racemate using the same synthetic route as for **Evo-5**, but through amide coupling with 1-amino-3-butyne instead of BODIPY (Scheme S10). While evobrutinib possesses a 4-substituted piperidine ring, the 3-substituted piperidine in ibrutinib gives rise to a chiral center. Formation of a second chiral center at sulfur, as a sulfoxide, would create diastereomers. To avoid this, it was desirable to prepare sulfones of the ibrutinib probes and various oxidizing agents were screened on a model compound to address the issue with N-oxidation. All oxidants initially formed sulfoxide. Subsequent oxidation afforded exclusively *N*-oxide for mCPBA, exclusively sulfone for H_2_O_2_/Na_2_WO_4_, and a mixture with Oxone. While both H_2_O_2_/Na_2_WO_4_ and Oxone formed the sulfone slowly, RuCl_3_/NaIO_4_ was found to give rapid and complete sulfone formation within 1 h. The system generates RuO_4_ in situ as a very potent oxidant, soluble in the CCl_4_ of a biphasic solvent system (CCl_4_/MeCN/H_2_O). The protocol was applied to prepare sulfone probes **Ibr-1** and **Ibr-2** in 43% and 35% isolated yield, respectively (Schemes S11 and S12). An observed side product arose from sequential oxidative cleavage of the alkyne to a carboxylic acid, which was minimized by using 3.0 equiv. NaIO_4_ and carefully monitoring the reaction.

### Biological Evaluation of Alkyne-Functionalized BTK Probes

The labeling profiles of the alkyne-tagged probes was assessed by in-gel fluorescence following Cu-catalyzed azide–alkyne cycloaddition with TAMRA-N_3_. Ramos cells were treated with 10–500 nM of **Evo-6**, **Ibr-1**, or **Ibr-2** for 1 h (Figures 6a and S13–S15) to determine a suitable concentration for BTK labeling with minimal off-target engagement. **Evo-6** produced hardly any detectable BTK labeling and was the least potent of the three probes. Replacement of the evobrutinib scaffold with ibrutinib markedly increased labeling efficiency; **Ibr-1** labeled BTK at 50 nM, whereas **Ibr-2** gave the strongest signal with detectable modification at 25 nM (Figure 6a). These results are consistent with the greater potency reported for ibrutinib (IC_50_ = 0.2 nM) compared with evobrutinib (IC_50_ = 8.9 nM).^63^ At higher concentrations (>250 nM), the BTK band became saturated, likely reflecting complete labeling of cellular BTK, and off-target signals increased (Figures S13–S15). A concentration of 100 nM was therefore selected as optimal for further experiments, providing an intense BTK band with adequate selectivity. These results agreed with the MD predictions, which positioned **Ibr-2** closest to Cys481 and in an orientation favorable for S_N_Ar reaction (Figure 5a,c).

**Figure 6:**
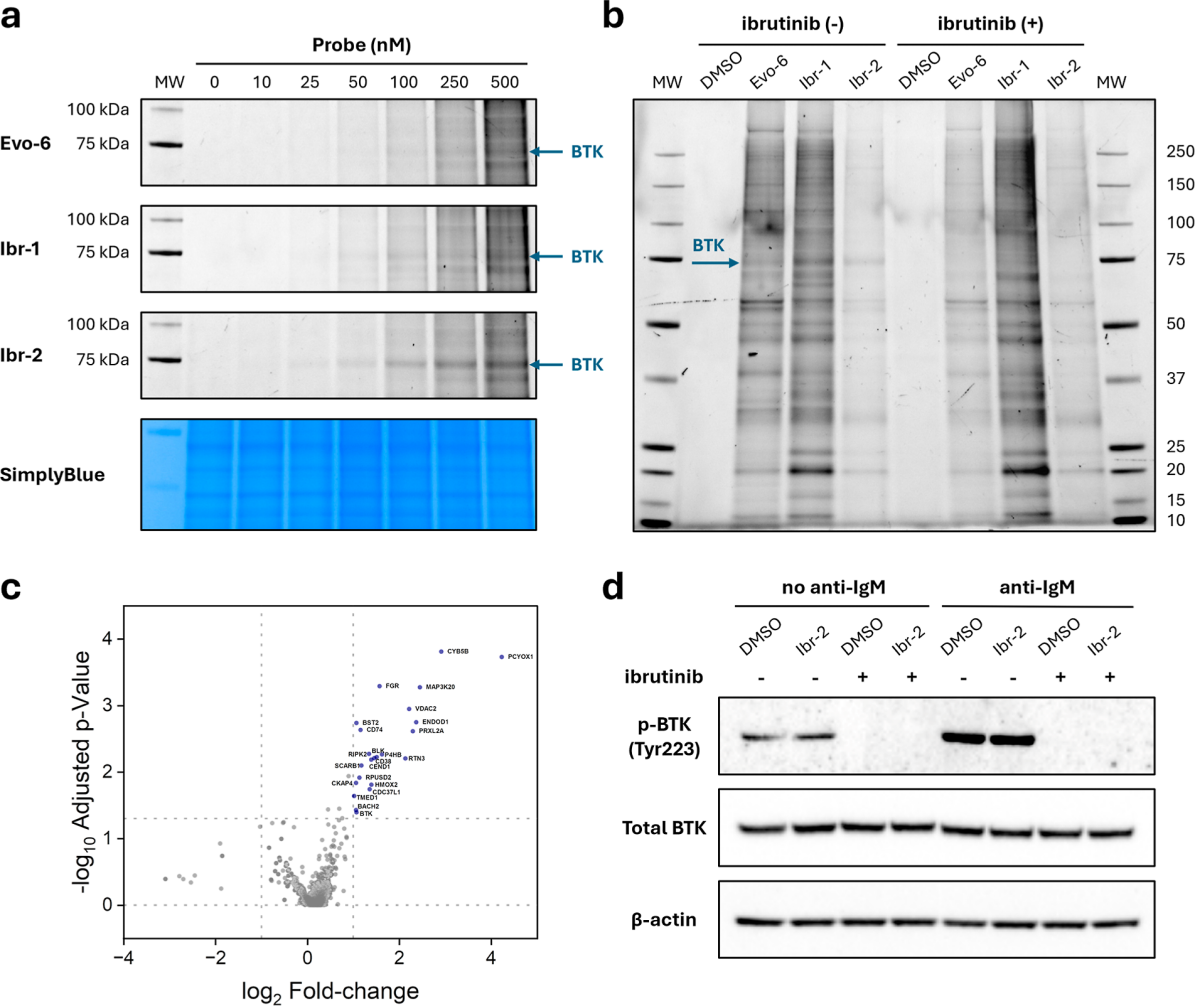
Biological evaluation of alkyne probes in living cells. (a) Dose-dependent labeling of cellular BTK. Ramos cells were treated with 10–500 nM probe for 1 h, and the resulting cell lysates were modified by click reaction with TAMRA-N_3_. Proteins were separated by SDS-PAGE, and the gel was imaged with ChemiDoc imaging system at two channels (green LED, 605/50 filter for TAMRA, red LED 695/50 filter for MW markers). Images were merged to generate the composite image. The blue arrow indicates the BTK band. The gels were subsequently stained with SimplyBlue for total protein visualization. Full gel images are presented in Figures S13–S15. (b) Cellular protein labeling profiles of the probes, together with ibrutinib competition, confirms BTK binding. Ramos cells were pretreated with either DMSO or 1 μM ibrutinib for 30 min, followed by treatment with 100 nM probe for 1 h. The resulting cell lysates were modified and analyzed as in (a). The blue arrow indicates the BTK band. (c) Volcano plot obtained from TMT-based quantitative proteomics analysis of the pull-down performed with **Ibr-2**. Ramos cells were treated with 250 nM **Ibr-2**, lysed, and conjugated to biotin-N_3_. Proteins with a log_2_ fold-change >1 compared to the DMSO control and an adjusted *p*-value <0.05 were considered significantly enriched (highlighted in blue and annotated). An enlarged version of the volcano plot is presented in Figure S19. (d) **Ibr-2** does not affect BTK activity in Ramos cells, as measured by BTK autophosphorylation. Cells were pretreated with either DMSO or 1 μM ibrutinib for 30 min, followed by treatment with 100 nM **Ibr-2** for 1 h. Thereafter, the cells were washed before BCR-stimulation with antihuman IgM (10 μg/mL) for 10 min. Proteins were separated by SDS-PAGE, transferred to nitrocellulose membranes, and immunoblotted with antibodies against phospho-BTK (Tyr223), total BTK, and β-actin.

Time-course experiments were performed with 100 nM of **Ibr-2** to assess the labeling kinetics, which revealed robust labeling of cellular BTK within 30–60 min (Figure S16). To directly compare labeling profiles and validate BTK binding, 100 nM of each probe was run on the same gel with and without preincubation with 1 μM ibrutinib as a competition experiment (Figures 6b and S17). Although **Ibr-1** exhibited a strong BTK band, both **Evo-6** and **Ibr-1** showed a broad reactivity resulting in extensive off-target labeling. Changing the linker (**Ibr-2**) preserved BTK binding while significantly improving selectivity (Figure 6b). Consistent with our NAC assay, removal of the electron-withdrawing amide reduced the warhead’s intrinsic reactivity, reflected by the substantially fewer bands observed for **Ibr-2** compared with the other probes. Preincubation with ibrutinib completely abolished the BTK band (Figure 6b), while most other bands were unaffected. This suggests that residual labeling likely arises from reactions with exposed cysteines unrelated to BTK recognition. The BTK band identity was further confirmed by a fluorescent Western blot (Figure S18), where the TAMRA-labeled band overlapped with an Alexa633-conjugated secondary antibody against BTK.

To gain a better understanding of the cellular proteins labeled by **Ibr-2**, we performed a pull-down proteomics experiment. Following a 1 h treatment of Ramos cells with either DMSO or 250 nM **Ibr-2**, the cells were washed, lysed, and conjugated to biotin-N_3_ via click chemistry. Sample preparation was carried out according to the SP2E workflow introduced by Becker et al.,^64^ including enrichment on streptavidin beads, digestion with trypsin, and LC–MS/MS analysis. Quantification of the **Ibr-2**-modified proteins using tandem mass tag (TMT) labeling showed that BTK was among the enriched proteins relative to the DMSO-treated control cells (Figures 6c and S19; Supporting Information Set). In addition to BTK-enrichment, several off-targets were identified for the probe.

To assess whether cellular BTK activity is affected by probe labeling, we measured autophosphorylation of BTK in Ramos cells. B-cell activation occurs through binding of specific antigens to the B-cell receptor (BCR), triggering a signaling cascade leading to BTK phosphorylation at Tyr551 by Src family kinases such as Lyn and Syk. Activated BTK subsequently undergoes autophosphorylation at Tyr223 to stabilize its active conformation.^65,66^ Western blotting showed a small amount of p-BTK (Tyr223) in resting Ramos cells, which increased substantially upon BCR-stimulation with antihuman IgM (Figure 6d). Pretreatment with 1 μM ibrutinib completely abolished BTK activity. No observable difference in phosphorylation at Tyr223 was detected between DMSO treated and **Ibr-2** treated cells, indicating that **Ibr-2** preserves the BTK enzymatic activity (Figure 6d). Furthermore, the toxicity of **Evo-6**, **Ibr-1**, and **Ibr-2** was evaluated in Ramos cells using the CellTiter-Glo assay (Figure S20). No significant effect on ATP levels in cell lysate was observed for concentrations up to 1 μM for any of the probes, indicating no cytotoxicity under the conditions used for cellular experiments.

### Mechanistic Investigations of Probe Binding

The IC_50_ values of the probes were determined using the ADP-Glo Kinase Assay to assess their effects on BTK enzymatic activity (Figure S21). Although IC_50_ values for covalent inhibitors are time-dependent because of bond formation, using a consistent incubation time across compounds allow their relative potencies to be determined. Ibrutinib displayed an IC_50_-value of 9.77 nM under our assay conditions, whereas the probes **Ibr-1** (340 nM) and **Ibr-2** (403 nM) were 35–40-fold less potent than their parent inhibitor. The weak inhibitory effect of the probes is expected, as these compounds act as noncovalent inhibitors that leave a silent tag on BTK. Since no washing step was performed, the released ligand remains present and behaves as a competitive inhibitor after labeling. To further evaluate whether **Ibr-2** interferes with ATP-binding, BTK was incubated with 500 nM **Ibr-2** or ibrutinib, followed by treatment with increasing concentrations of ATP. While ibrutinib strongly suppressed BTK activity, the luminescence signal observed with **Ibr-2** was comparable to that of the DMSO control (Figure S22), indicating preserved kinase activity postlabeling. This finding is consistent with unaffected cellular BTK activity upon treatment with 100 nM **Ibr-2** (Figure 6d).

Ibrutinib, its analogue lacking the acrylamide warhead **(Ibr-NH)**, **Ibr-1**, and **Ibr-2**, were investigated in a direct binding assay using surface plasmon resonance (SPR) biosensor. The sequential methodology of SPR measurements, that compounds are injected one at a time over the same immobilized protein, typically renders SPR incompatible with irreversible compounds. To circumvent this shortcoming, we utilized a regenerable protein immobilization using SwitchAvidin.^67^ Here, biotinylated BTK was premixed with SwitchAvidin and immobilized as a complex that could subsequently be stripped from the sensor surface to enable immobilization of new BTK (Figure 7a).^68^

**Figure 7:**
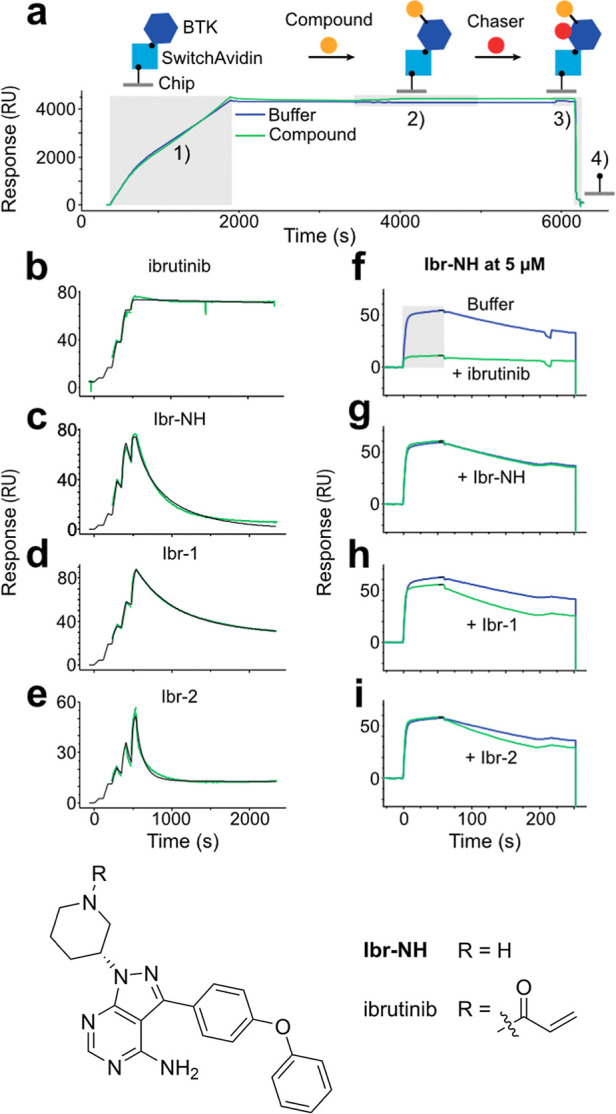
Surface plasmon resonance (SPR) binding assay support mode-of-action of **Ibr-1** and **Ibr-2**. (a) Regenerable assay setup including (1) protein immobilization, (2) single-cycle kinetics (SCK) titration followed by (3) chaser injection before (4) surface regeneration. (b–e) Binding trace during SCK of ibrutinib, **Ibr-NH**, **Ibr-1**, and **Ibr-2**. Increasing concentrations of compound (up to 3 μM) were injected for 60 s each. (f–i) Corresponding binding trace of chaser. After 30 min washing, 5 μM **Ibr-NH** was injected to probe the binding capacity. The gray square in (f) highlights duration of compound injection (60 s), sufficient to reach saturated binding.

Using this strategy, we could estimate the binding affinities (*K*_D_) of ibrutinib and **Ibr-NH** as 0.2 ± 0.1 and 15 ± 5 nM respectively (Figures 7b,c), in line with previous reports.^63^ Importantly, these compounds also served as positive and negative control for the subsequent chaser injection (5 μM **Ibr-NH**) after 30 min of continuous washing (Figure 7a). The chaser reports on the occupancy of the binding pocket (inversely proportional to the amplitude of chaser binding at saturation). A buffer sample was used to calibrate for 100% free binding pocket (blue curves, Figure 7f–i). As expected, the irreversible binding of ibrutinib renders the binding pocket nearly fully occupied despite 30 min continuous washing, as evident from the reduced amplitude of the chaser (Figure 7f). In contrast, the reversible analogue completely washed off resulting in identical amplitude of the chaser versus the buffer sample (Figure 7g).

For **Ibr-1** and **Ibr-2**, the binding is more complex and cannot be estimated with a 1:1 interaction (Figure 7d,e). This is expected as the probes upon binding not only will react covalently with BTK, but by doing so release bound mass from the surface. Using a heterogeneous fit, the covalently bound fraction can be estimated to 30 ± 5% and 25 ± 5% for **Ibr-1** and **Ibr-2**, respectively. This is close in line with the molecular mass of the remaining covalent adduct (175 Da) from **Ibr-1** (666 Da) and **Ibr-2** (569 Da). Importantly, the subsequent chaser injections suggest that the leaving group has been washed off resulting in an almost completely free binding pocket for both probes (Figure 7h,i). Interestingly, the influence of the adduct can still be indirectly observed as it slightly modulates the dissociation of the chaser, making it 1.5-fold faster. Corresponding analyses were also generated for evobrutinib and **Evo-6**. Both compounds displayed weak reversible binding resulting in only partial occupancy of BTK in the time frame of the experiment, which prevented a quantitative analysis.

To further validate the ligand-release mechanism and gain an insight into the reaction kinetics, a time-dependent incubation experiment of full-length recombinant BTK was performed with various concentrations of **Ibr-2**. Intact mass spectrometry analysis revealed that BTK was at least 3-times phosphorylated (Figure S23). In order to obtain more accurate results, the labeling quantification was performed on peptide level after tryptic digestion (denaturation and cysteine alkylation with iodoacetamide). Relative quantification of the labeled/unlabeled Cys481-containing peptides revealed that labeling is complete after 10 min and the degree of modification increased with probe concentration (Figures S24 and S25a). Due to the rapid reaction and the experimental setup, extrapolation of the kinetic parameters *k*_inact_ and *K*_I_ was not possible. Additionally, a mass shift of 116 Da was observed between the **Ibr-2**-labeled and carbamidomethylated tryptic peptides containing the reactive Cys481 (Figure S25b), confirming the arylation of Cys481 by **Ibr-2**. Partial modification of few other cysteines in the sequence was observed only for the higher concentrations of **Ibr-2**. Taken together, these results, in combination with the SPR data further strengthen the proposed ligand-release mechanism of the S_N_Ar-probes.

## Conclusions

In summary, we have developed a tunable platform of sulfur-based S_N_Ar warheads bearing two orthogonal handles for ligand and tag installation, enabling their streamlined incorporation into ligand-directed chemistry probes. Forty-eight warhead variants were synthesized, systematically exploring the influence of the aromatic core, leaving-group electronics, and sulfur oxidation state on reactivity and stability. These studies revealed that the aromatic scaffold is the primary determinant of cysteine reactivity, with leaving group and oxidation state modifications providing additional fine-tuning, whereas triazole linkers completely suppressed reactivity. Importantly, all warheads showed excellent chemoselectivity for cysteine thiols over lysine and serine derivatives. Buffer stability correlated strongly with cysteine reactivity, with the most reactive scaffolds exhibiting the fastest decomposition. In addition, sulfoxides were consistently more stable than their corresponding sulfones.

Selected scaffolds were incorporated into BTK-targeting probes, which demonstrated labeling of recombinant BTK with intensities predictable from the reactivity assay. A pyrazine-based scaffold was identified with suitable reactivity for cellular labeling at nanomolar concentrations but exhibited limited cellular selectivity in evobrutinib-based constructs. Molecular dynamics simulations revealed differences in hydrogen-bond occupancy and geometrical orientation between ibrutinib- and evobrutinib-based probes, which may influence the warhead’s reactivity with Cys481. Probe optimization produced the ibrutinib-derived probe **Ibr-2** which exhibited potent BTK labeling in cells with improved selectivity over other probes used in this study. Furthermore, **Ibr-2** preserved BTK enzymatic activity and showed no measurable cytotoxicity. Its flexible alkyne tag enables further modifications and was used in chemoproteomic profiling to identify probe off-targets. SPR measurements using SwitchAvidin for regenerable protein immobilization enabled direct analysis of irreversible probe binding and supported the ligand-release mechanism, demonstrating that the BTK active site remains unoccupied after covalent labeling. Finally, time-course protein MS analysis revealed rapid reaction kinetics, with complete modification of Cys481 of BTK within 10 min.

Together, these findings establish general design principles for incorporating tunable S_N_Ar warheads into labeling probes that modify Cys481 of BTK. The aromatic sulfoxide and sulfone scaffolds emerge as a promising platform for the development of selective, traceless covalent probes for other proteins, particularly kinases containing accessible noncatalytic cysteines, and represent a valuable addition to the chemical biology toolbox.

## Experimental Section

### General Information

All solvents and reagents were obtained from commercial suppliers, stored as indicated by the suppliers, and used without further purification unless otherwise stated. Ibrutinib and TAMRA-N_3_ were purchased from MedChemExpress. Biotin-N_3_ was purchased from Merck. Dry DCM, DMF, and THF were obtained from a solvent purification system (PS-MD-5/7 Inert technology). Reactions were monitored by TLC, LC–MS, and/or HPLC. ^1^H NMR, ^13^C NMR, and ^19^F NMR spectra were recorded on a 600 MHz Bruker Avance Neo, a 700 MHz Bruker Avance III, or an 800 MHz Bruker Advance III HD spectrometer at 25 °C. All chemical shifts (^1^H, ^13^C) are reported in parts per million (δ) relative to the residual solvent peak (CDCl_3_: 7.26 ppm, 77.16 ppm; (CD_3_)_2_SO: 2.50 ppm, 39.52 ppm; (CD_3_)_2_CO: 2.05 ppm, 29.84 ppm; CD_3_OD: 3.31 ppm, 49.00 ppm). The following abbreviations are used to denote the multiplicities: s = singlet, br s = broad singlet, d = doublet, dd = doublet of doublets, t = triplet, td = triplet of doublets, tt = triplet of triplets, q = quartet, qd = quartet of doublets, m = multiplet. Coupling constants (*J*) are reported in Hz.

TLC was conducted on silica-gel-coated aluminum sheets for normal-phase (Merck TLC Silica gel 60 F_254_) respectively reverse-phase (Merck TLC Silica gel 60 RP-18 F_254_s) and were visualized by UV light (λ = 254 or 366 nm). Preparative TLC was carried out silica-gel-coated glass plates (Analtech Uniplate Silica gel GF). GC–MS was performed on an Agilent 7820A GC system with an Agilent 5977E MSD mass detector. LC–MS was performed on a Waters Acquity system (Acquity Arc HPLC system; 2489 UV/vis Detector; XBridge BEH C18 column, 130 Å, 2.5 μm, 2.1 × 50 mm; XBridge BEH C18 Guard column, V–Gd Cart 2.5 μm, 2.1 × 5 mm; Acquity QDa Mass Detector; MeCN/water (0.01% formic acid), 40 °C).

Analytical HPLC was performed on a Waters system (2690 Separation Module; 996 Photodiode Array Detector; Chromolith SpeedROD RP-18 end-capped 50–4.6 HPLC column; MeCN/water (0.1% TFA)). Preparative HPLC was performed on a Waters system (1525 Binary HPLC Pump; 2998 Photodiode Array Detector; Atlantis Prep T3 OBD column; MeCN/water (0.1% TFA)), by injecting the crude dissolved in MeOH. Column chromatography was performed on a Selekt or Isolera One flash chromatography system (Biotage) for normal-phase or reverse-phase, respectively. The silica gel was Sfär Silica D Duo 60 μm or Sfär Silica HC D High Capacity Duo 20 μm cartridges for normal-phase, and Sfär C18 D Duo 100 Å 30 μm cartridges for reverse-phase (Biotage). For all column chromatography, dry loading was performed using the same type of silica as the stationary phase.

HRMS data were recorded with a QExactive HF Orbitrap mass spectrometer interfaced with Dionex Ultimate 3000 liquid chromatography system (Thermo Fisher Scientific). The instrument operated in full MS mode only, where the ion mass spectra were acquired at a resolution of 120,000, maximum injection time 200 ms for 3 × 10^6^ ions. The Orbitrap was calibrated with Pierce LTQ ESI Positive Ion Calibration Solution prior to the analysis, resulting in mass accuracy better than 5 ppm. Electrospray ionization was performed at 4 kV and 320 °C using a metal emitter in the ion source. The sample (1 or 10 μL) was injected onto a reversed-phase XBridge BEH C18 column (3.5 μm, 2.1 × 50 mm, Waters). The analysis was performed using a linear gradient over 2.5 min from 10 to 100% solvent B, followed by isocratic eluted with 100% solvent B for 17.5 min with a flow of 0.300 mL/min (solvent A: water with 0.1% formic acid; solvent B: 80% acetonitrile in water with 0.1% formic acid). Data analysis was performed using the Xcalibur software (Thermo Fischer Scientific).

Synthesis procedures and characterization data of all prior intermediates in the route toward the final compounds can be found in the Supporting Information. All compounds are >95% pure by HPLC analysis.

### General Procedure for mCPBA-Mediated S-Oxidation of Aryl Thioethers for the Preparation of Aryl Sulfoxides and Aryl Sulfones

In a round-bottom flask, the respective aryl thioether (1.0 equiv) was dissolved in CHCl_3_ and cooled to 0 °C. Then a solution of mCPBA (1.0–5.0 equiv) in CHCl_3_ was added dropwise and the reaction mixture was stirred at 0 °C for 30 min. The mixture was allowed to warm to room temperature and was further stirred until TLC and LC–MS analysis indicated complete reaction. For some less-reactive substrates, heating of the mixture to 50 °C was required. The reaction mixture was then partitioned between CHCl_3_ and an aqueous solution of saturated Na_2_S_2_O_3_ and Na_2_CO_3_ (1:1). The aqueous layer was extracted twice with CHCl_3_. The combined organic layers were washed twice with saturated aqueous Na_2_CO_3_, washed once with brine, dried over Na_2_SO_4_, and concentrated under reduced pressure. The crude was then charged on silica and purified by column chromatography to afford the respective aryl sulfoxides and aryl sulfones. In some cases, the crude product was of high purity, and no column purification was required. Notes: (a) In some cases, the respective thioether was oxidized to yield a mixture of sulfoxide and sulfone, where both products were isolated from the same reaction mixture following column chromatography. (b) In some cases, isolated aryl sulfoxide (1.0 equiv) was oxidized with mCPBA to yield the respective sulfone.

### Synthesis and Characterization of Aryl Sulfoxides (**4a-4o**, **5a-5m**)

#### *tert*-butyl 4-((2-methoxy-2-oxoethyl)­sulfinyl)-3-(trifluoromethyl)­benzoate (**4a**)

Following GP, thioether **2a** (70 mg, 0.20 mmol, 1.0 equiv) was oxidized with mCPBA (54 mg, 0.24 mmol, 1.2 equiv) for 30 min at 0 °C followed by 1 h at room temperature, to afford the title compound (71 mg, 98%) as a white crystalline solid. The crude did not require further purification. Rf = 0.27 (EtOAc/pentane 1:4). ^1^H NMR (600 MHz, CDCl_3_) δ: 8.38 (dd, *J* = 8.3, 1.7 Hz, 1H), 8.34–8.30 (m, 2H), 3.83 (d, *J* = 13.9 Hz, 1H), 3.76 (s, 3H), 3.61 (d, *J* = 14.0 Hz, 1H), 1.61 (s, 9H); ^13^C NMR (151 MHz, CDCl_3_) δ: 164.7, 163.3, 147.0, 135.5, 133.9, 127.7 (q, ^3^ *J*_CF_ = 5 Hz), 126.9 (q, ^2^ *J*_CF_ = 34 Hz), 126.0, 123.1 (q, ^1^ *J*_CF_ = 275 Hz), 83.1, 61.1, 53.1, 28.2; ^19^F NMR (564 MHz, CDCl_3_) δ: −57.46; HRMS (ESI) *m*/*z*: [M + H]^+^ calcd for C_15_H_18_F_3_O_5_S, 367.0822; found, 367.0815.

#### *tert*-butyl 4-((2-methoxy-2-oxoethyl)­sulfinyl)-3-nitrobenzoate (**4b**)

Following GP, thioether **2b** (73 mg, 0.22 mmol, 1.0 equiv) was oxidized with mCPBA (65 mg, 0.29 mmol, 1.3 equiv) for 30 min at 0 °C followed by 15 min at room temperature, to afford the title compound (74 mg, 96%) as a yellow crystalline solid. The crude did not require further purification. Rf = 0.40 (EtOAc/pentane 3:7). ^1^H NMR (600 MHz, CDCl_3_) δ: 8.84 (d, *J* = 1.7 Hz, 1H), 8.51 (dd, *J* = 8.2, 1.7 Hz, 1H), 8.35 (d, *J* = 8.1 Hz, 1H), 4.11 (d, *J* = 13.6 Hz, 1H), 3.80 (d, *J* = 13.6 Hz, 1H), 3.72 (s, 3H), 1.62 (s, 9H); ^13^C NMR (151 MHz, CDCl_3_) δ: 165.0, 162.6, 146.3, 144.9, 136.3, 135.7, 127.6, 126.1, 83.6, 59.8, 53.0, 28.2; HRMS (ESI) *m*/*z*: [M + H]^+^ calcd for C_14_H_18_NO_7_S, 344.0798; found, 344.0790.

#### Methyl 6-((2-(*tert*-butoxy)-2-oxoethyl)­sulfinyl)­nicotinate (**4e**)

Following GP, thioether **2e** (97 mg, 0.34 mmol, 1.0 equiv) was oxidized with mCPBA (77 mg, 0.34 mmol, 1.0 equiv) for 30 min at 0 °C followed by 15 min at room temperature, to afford the title compound (78 mg, 77%) as a white crystalline solid. The crude was purified by column chromatography (silica 10 g, 0–50% EtOAc/pentane) with the product eluting at 50% EtOAc. ^1^H NMR (600 MHz, CDCl_3_) δ: 9.16 (d, *J* = 2.1 Hz, 1H), 8.52 (dd, *J* = 8.1, 2.1 Hz, 1H), 8.09 (d, *J* = 8.1 Hz, 1H), 4.02 (d, *J* = 14.0 Hz, 1H), 3.96 (s, 3H), 3.77 (d, *J* = 14.0 Hz, 1H), 1.40 (s, 9H); ^13^C NMR (151 MHz, CDCl_3_) δ: 168.6, 164.8, 163.6, 150.6, 139.1, 127.1, 120.2, 83.5, 59.1, 52.9, 28.0; HRMS (ESI) *m*/*z*: [M + H]^+^ calcd for C_13_H_18_NO_5_S, 300.0900; found, 300.0901.

#### *tert*-butyl 6-((2-methoxy-2-oxoethyl)­sulfinyl)-5-(trifluoromethyl)­nicotinate (**4f**)

Following GP, thioether **2f** (30 mg, 0.086 mmol, 1.0 equiv) was oxidized with mCPBA (23 mg, 0.10 mmol, 1.2 equiv) for 30 min at 0 °C followed by 1 h at room temperature, to afford the title compound (26 mg, 83%) as a pale-yellow crystalline solid. The crude did not require further purification. Rf = 0.10 (EtOAc/pentane 1:4). ^1^H NMR (600 MHz, CDCl_3_) δ: 9.45 (d, *J* = 2.0 Hz, 1H), 8.59 (d, *J* = 2.0 Hz, 1H), 4.22 (d, *J* = 14.0 Hz, 1H), 4.06 (d, *J* = 14.0 Hz, 1H), 3.70 (s, 3H), 1.63 (s, 9H); ^13^C NMR (151 MHz, CDCl_3_) δ: 164.7, 163.5, 161.9, 154.4, 136.3 (q, ^3^ *J*_CF_ = 7 Hz), 130.1, 125.7 (q, ^2^ *J*_CF_ = 35 Hz), 122.3 (q, ^1^ *J*_CF_ = 274 Hz), 84.3, 57.5, 53.1, 28.2; ^19^F NMR (564 MHz, CDCl_3_) δ: −57.76; HRMS (ESI) *m*/*z*: [M + H]^+^ calcd for C_14_H_17_F_3_NO_5_S, 368.0774; found, 368.0768.

#### *tert*-butyl 6-((2-methoxy-2-oxoethyl)­sulfinyl)-5-nitronicotinate (**4g**)

Following GP, thioether **2g** (75 mg, 0.23 mmol, 1.0 equiv) was oxidized with mCPBA (66 mg, 0.30 mmol, 1.3 equiv) for 30 min at 0 °C followed by 1 h at room temperature, to afford the title compound (38 mg, 49%) as a red crystalline solid. The crude was purified by column chromatography (silica 5 g, 20–80% EtOAc/pentane) with the product eluting at 70% EtOAc. Rf = 0.27 (EtOAc/pentane 3:2). ^1^H NMR (600 MHz, CDCl_3_) δ: 9.57 (d, *J* = 1.8 Hz, 1H), 8.98 (d, *J* = 1.9 Hz, 1H), 4.12 (d, *J* = 13.5 Hz, 1H), 4.01 (d, *J* = 13.4 Hz, 1H), 3.74 (s, 3H), 1.64 (s, 9H); ^13^C NMR (151 MHz, CDCl_3_) δ: 165.0, 163.4, 161.3, 155.5, 143.0, 134.2, 130.8, 84.8, 58.3, 53.3, 28.2; HRMS (ESI) *m*/*z*: [M + H]^+^ calcd for C_13_H_17_N_2_O_7_S, 345.0751; found, 345.0742.

#### Methyl 2-((2-(*tert*-butoxy)-2-oxoethyl)­sulfinyl)­pyrimidine-5-carboxylate (**4h**)

Following GP, thioether **2h** (120 mg, 0.42 mmol, 1.0 equiv) was oxidized with mCPBA (123 mg, 0.55 mmol, 1.3 equiv) for 30 min at 0 °C followed by 30 min at room temperature, to afford the title compound (68 mg, 54%) as a white solid. The crude was purified by column chromatography (high-capacity silica 5 g, 20–100% EtOAc/pentane) with the product eluting at 90% EtOAc. Rf = 0.28 (EtOAc/pentane 3:2). ^1^H NMR (600 MHz, CDCl_3_) δ: 9.34 (s, 2H), 4.10 (d, *J* = 14.2 Hz, 1H), 4.01–3.96 (m, 4H), 1.37 (s, 9H); ^13^C NMR (151 MHz, CDCl_3_) δ: 176.4, 163.4, 163.2, 159.3, 124.4, 83.7, 58.0, 53.2, 28.0; HRMS (ESI) *m*/*z*: [M + Na]^+^ calcd for C_12_H_16_N_2_NaO_5_S, 323.0672; found, 323.0666.

#### Methyl 2-((2-methoxy-2-oxoethyl)­sulfinyl)­pyrimidine-4-carboxylate (**4i**)

Following GP, thioether **2i** (14 mg, 0.059 mmol, 1.0 equiv) was oxidized with mCPBA (14 mg, 0.064 mmol, 1.1 equiv) for 30 min at 0 °C followed by 1.5 h at room temperature, to afford the title compound (6.2 mg, 41%) as a red oil. The crude was purified by column chromatography (high-capacity silica 5 g, 50–100% EtOAc/pentane, then 0–25% MeOH/EtOAc) with the product eluting at 5–10% MeOH. Rf = 0.14 (EtOAc). ^1^H NMR (600 MHz, CDCl_3_) δ: 9.15 (d, *J* = 4.9 Hz, 1H), 8.07 (d, *J* = 4.9 Hz, 1H), 4.23 (d, *J* = 14.1 Hz, 1H), 4.09 (d, *J* = 14.1 Hz, 1H), 4.03 (s, 3H), 3.73 (s, 3H); ^13^C NMR (151 MHz, CDCl_3_) δ: 173.4, 165.1, 163.5, 161.0, 156.2, 121.5, 57.2, 53.8, 53.1; HRMS (ESI) *m*/*z*: [M + H]^+^ calcd for C_9_H_11_N_2_O_5_S, 259.0383; found, 259.0382.

#### Methyl 3-(2-((2-(*tert*-butoxy)-2-oxoethyl)­sulfinyl)­pyrimidin-5-yl)­propanoate (**4j**)

Following GP, thioether **2j** (61 mg, 0.19 mmol, 1.0 equiv) was oxidized with mCPBA (48 mg, 0.21 mmol, 1.1 equiv) for 30 min at 0 °C followed by 2 h at room temperature, to afford the title compound (38 mg, 60%) as a white crystalline solid. The crude was purified by column chromatography (silica 10 g, 20–100% EtOAc/pentane) with the product eluting at 80% EtOAc. ^1^H NMR (600 MHz, CDCl_3_) δ: 8.74 (s, 2H), 4.06 (d, *J* = 14.1 Hz, 1H), 3.93 (d, *J* = 14.1 Hz, 1H), 3.65 (s, 3H), 3.01 (t, *J* = 7.2 Hz, 2H), 2.69 (t, *J* = 7.2 Hz, 2H), 1.39 (s, 9H); ^13^C NMR (151 MHz, CDCl_3_) δ: 172.0, 170.6, 163.8, 158.5, 134.8, 83.4, 58.5, 52.1, 34.3, 28.0, 25.3; HRMS (ESI) *m*/*z*: [M + H]^+^ calcd for C_14_H_21_N_2_O_5_S, 329.1166; found, 329.1168.

#### Methyl 6-((2-methoxy-2-oxoethyl)­sulfinyl)­pyridazine-3-carboxylate (**4k**)

Following GP, thioether **2k** (49 mg, 0.20 mmol, 1.0 equiv) was oxidized with mCPBA (50 mg, 0.22 mmol, 1.1 equiv) for 30 min at 0 °C followed by 1.5 h at room temperature, to afford the title compound (18 mg, 35%) as a white crystalline solid. The crude was purified by column chromatography (silica 5 g, 20–100% EtOAc/pentane) with the product eluting at 100% EtOAc. Rf = 0.15 (EtOAc/pentane 3:2). ^1^H NMR (600 MHz, CDCl_3_) δ: 8.45 (d, *J* = 8.7 Hz, 1H), 8.32 (d, *J* = 8.6 Hz, 1H), 4.33 (d, *J* = 14.4 Hz, 1H), 4.10 (s, 3H), 4.06 (d, *J* = 14.4 Hz, 1H), 3.73 (s, 3H); ^13^C NMR (151 MHz, CDCl_3_) δ: 170.9, 164.5, 163.8, 152.0, 129.4, 125.0, 58.1, 53.8, 53.2; HRMS (ESI) *m*/*z*: [M + H]^+^ calcd for C_9_H_11_N_2_O_5_S, 259.0383; found, 259.0384.

#### Methyl 5-((2-methoxy-2-oxoethyl)­sulfinyl)­pyrazine-2-carboxylate (**4l**)

Following GP, thioether **2l** (38 mg, 0.16 mmol, 1.0 equiv) was oxidized with mCPBA (39 mg, 0.17 mmol, 1.1 equiv) for 30 min at 0 °C followed by 1 h at room temperature, to afford the title compound (27 mg, 68%) as a white solid. The crude was purified by column chromatography (silica 5 g, 20–100% EtOAc/pentane) with the product eluting at 100% EtOAc. Rf = 0.17 (EtOAc/pentane 3:2). ^1^H NMR (800 MHz, CDCl_3_) δ: 9.33–9.25 (m, 2H), 4.22 (d, *J* = 14.2 Hz, 1H), 4.08 (s, 3H), 3.98 (d, *J* = 14.2 Hz, 1H), 3.74 (s, 3H); ^13^C NMR (201 MHz, CDCl_3_) δ: 164.6, 163.7, 163.1, 145.0, 144.2, 142.2, 57.5, 53.7, 53.2; HRMS (ESI) *m*/*z*: [M + H]^+^ calcd for C_9_H_11_N_2_O_5_S, 259.0383; found, 259.0382.

#### Methyl 2-((2-methoxy-2-oxoethyl)­sulfinyl)­thiazole-5-carboxylate (**4m**)

Following GP, thioether **2m** (28 mg, 0.11 mmol, 1.0 equiv) was oxidized with mCPBA (33 mg, 0.15 mmol, 1.3 equiv) for 30 min at 0 °C followed by 15 min at room temperature, to afford the title compound (22 mg, 74%) as a clear oil. The crude was purified by column chromatography (silica 5 g, 30–60% EtOAc/pentane) with the product eluting at 40% EtOAc. ^1^H NMR (800 MHz, CDCl_3_) δ: 8.47 (s, 1H), 4.19 (d, *J* = 14.3 Hz, 1H), 4.03 (d, *J* = 14.3 Hz, 1H), 3.94 (s, 3H), 3.79 (s, 3H); ^13^C NMR (201 MHz, CDCl_3_) δ: 180.5, 164.3, 160.8, 149.6, 133.9, 60.2, 53.3, 53.1; HRMS (ESI) *m*/*z*: [M + H]^+^ calcd for C_8_H_10_NO_5_S_2_, 263.9995; found, 263.9993.

#### Ethyl 6-((2-(*tert*-butoxy)-2-oxoethyl)­sulfinyl)-5-cyano-2-methylnicotinate (**4n**)

Following GP, thioether **2n** (82 mg, 0.24 mmol, 1.0 equiv) was oxidized with mCPBA (60 mg, 0.27 mmol, 1.1 equiv) for 30 min at 0 °C followed by 15 min at room temperature, to afford the title compound (33 mg, 38%) as a yellow crystalline solid. The crude was purified by column chromatography (silica 10 g, 0–50% EtOAc/pentane) with the product eluting at 40% EtOAc. Rf = 0.51 (EtOAc/pentane 1:1). ^1^H NMR (600 MHz, CDCl_3_) δ: 8.61 (s, 1H), 4.43 (q, *J* = 7.1 Hz, 2H), 4.24 (d, *J* = 14.1 Hz, 1H), 4.01 (d, *J* = 14.1 Hz, 1H), 2.94 (s, 3H), 1.45–1.39 (m, 12H); ^13^C NMR (151 MHz, CDCl_3_) δ: 166.3, 164.5, 163.7, 163.3, 145.1, 127.2, 113.2, 107.3, 84.2, 62.7, 58.1, 28.0, 25.3, 14.3; LC–MS (ESI) *m*/*z*: [M + H-*t*Bu]^+^ calcd for C_12_H_13_N_2_O_5_S, 297.05; found, 297.04 (fragmentation of *t*-Bu group).

#### *tert*-butyl 4-((4-((2-methoxy-2-oxoethyl)­sulfinyl)-2-(trifluoromethyl)­phenyl)­sulfonyl)­piperazine-1-carboxylate (**4o**)

Following GP, thioether **2o** (9.3 mg, 0.019 mmol, 1.0 equiv) was oxidized with mCPBA (4.6 mg, 0.021 mmol, 1.1 equiv) for 30 min at 0 °C followed by 1.5 h at room temperature, to afford the title compound (5.7 mg, 59%) as a white solid. The crude was purified by column chromatography (silica 5 g, 10–70% EtOAc/pentane) with the product eluting at 70% EtOAc. Rf = 0.21 (EtOAc/pentane 2:3). ^1^H NMR (600 MHz, CDCl_3_) δ: 8.29 (d, *J* = 8.3 Hz, 1H), 8.18 (d, *J* = 1.8 Hz, 1H), 8.04 (dd, *J* = 8.3, 1.8 Hz, 1H), 3.90 (d, *J* = 14.1 Hz, 1H), 3.82 (d, *J* = 14.1 Hz, 1H), 3.76 (s, 3H), 3.51 (t, *J* = 5.0 Hz, 4H), 3.25 (t, *J* = 5.1 Hz, 4H), 1.44 (s, 9H); ^13^C NMR (151 MHz, CDCl_3_) δ: 164.6, 154.3, 149.4, 140.9, 133.1, 129.6 (q, ^2^ *J*_CF_ = 34 Hz), 128.3, 124.7 (q, ^3^ *J*_CF_ = 7 Hz), 121.9 (q, ^1^ *J*_CF_ = 274 Hz), 80.8, 61.0, 53.3, 45.7, 44.1, 43.0, 28.5; ^19^F NMR (564 MHz, CDCl_3_) δ: −57.58; HRMS (ESI) *m*/*z*: [M + NH_4_]^+^ calcd for C_19_H_29_F_3_N_3_O_7_S_2_, 532.1394; found, 532.1399.

#### *tert*-butyl 4-((2-((*tert*-butoxycarbonyl)­amino)­ethyl)­sulfinyl)-3-(trifluoromethyl)­benzoate (**5a**)

Following GP, thioether **3a** (311 mg, 0.74 mmol, 1.0 equiv) was oxidized with mCPBA (174 mg, 0.77 mmol, 1.05 equiv) for 30 min at 0 °C followed by 1.5 h at room temperature, to afford the title compound (139 mg, 43%) as a white gummy solid. The crude was purified by column chromatography (silica 10 g, 0–50% EtOAc/pentane) with the product eluting at 50% EtOAc. ^1^H NMR (600 MHz, CDCl_3_) δ: 8.36 (dd, *J* = 8.3, 1.7 Hz, 1H), 8.32–8.28 (m, 2H), 5.22 (t, *J* = 5.9 Hz, 1H), 3.68–3.51 (m, 2H), 3.31–3.22 (m, *J* = 1H), 2.77 (dt, *J* = 13.7, 4.6 Hz, 1H), 1.60 (s, 9H), 1.41 (s, 9H); ^13^C NMR (151 MHz, CDCl_3_) δ: 163.4, 155.8, 147.9, 135.1, 133.8, 127.8 (q, ^3^ *J*_CF_ = 5 Hz), 126.8 (q, ^2^ *J*_CF_ = 33 Hz), 125.7, 123.2 (q, ^1^ *J*_CF_ = 275 Hz), 83.0, 80.0, 57.2, 35.2, 28.4, 28.2. ^19^F NMR (564 MHz, CDCl_3_) δ: −57.48; HRMS (ESI) *m*/*z*: [M + H]^+^ calcd for C_19_H_27_F_3_NO_5_S, 438.1557; found, 438.1558.

#### Methyl 4-((2-((*tert*-butoxycarbonyl)­amino)­ethyl)­sulfinyl)-3-nitrobenzoate (**5b**)

Following GP, thioether **3b** (190 mg, 0.53 mmol, 1.0 equiv) was oxidized with mCPBA (239 mg, 1.07 mmol, 2.0 equiv) for 30 min at 0 °C followed by warming the reaction mixture to room temperature and subsequently heating at 50 °C for 48 h, to afford the title compound (74 mg, 37%) as a yellow solid. The crude was purified by column chromatography (silica 5 g, 0–50% EtOAc/pentane) with the product eluting at 50% EtOAc. ^1^H NMR (600 MHz, CDCl_3_) δ: 8.89 (d, *J* = 1.7 Hz, 1H), 8.54 (dd, *J* = 8.2, 1.6 Hz, 1H), 8.38 (d, *J* = 8.1 Hz, 1H), 5.25 (t, *J* = 6.2 Hz, 1H), 4.00 (s, 3H), 3.72–3.45 (m, 3H), 2.91 (dt, *J* = 12.7, 4.3 Hz, 1H), 1.40 (s, 9H); ^13^C NMR (151 MHz, CDCl_3_) δ: 164.2, 155.8, 148.0, 144.7, 135.7, 133.9, 127.4, 126.4, 80.0, 56.1, 53.2, 35.4, 28.4; HRMS (ESI) *m*/*z*: [M + H]^+^ calcd for C_15_H_21_N_2_O_7_S, 373.1064; found, 373.1066.

#### *tert*-butyl 4-((4-((2-((*tert*-butoxycarbonyl)­amino)­ethyl)­sulfinyl)-3-cyanophenyl)­sulfonyl)­piperazine-1-carboxylate (**5c**)

Following GP, thioether **3c** (80 mg, 0.15 mmol, 1.0 equiv) was oxidized with mCPBA (51 mg, 0.23 mmol, 1.5 equiv) for 30 min at 0 °C followed by 30 min at room temperature, to afford the title compound (34 mg, 41%) as a white solid. The crude was purified by column chromatography (silica 5 g, 0–70% EtOAc/pentane) with the product eluting at 70% EtOAc. ^1^H NMR (600 MHz, CDCl_3_) δ: 8.25 (d, *J* = 8.3 Hz, 1H), 8.15 (dd, *J* = 8.2, 1.8 Hz, 1H), 8.08 (d, *J* = 1.7 Hz, 1H), 5.16 (t, *J* = 6.0 Hz, 1H), 3.68–3.55 (m, 2H), 3.53 (t, *J* = 5.1 Hz, 4H), 3.47–3.39 (m, 1H), 3.10–3.00 (m, 5H), 1.42–1.39 (m, 18H); ^13^C NMR (151 MHz, CDCl_3_) δ: 155.8, 154.1, 153.1, 140.0, 132.5, 132.4, 126.8, 113.5, 110.0, 80.9, 80.4, 55.5, 46.0, 43.5, 42.6, 35.0, 28.4; HRMS (ESI) *m*/*z*: [M + H]^+^ calcd for C_23_H_35_N_4_O_7_S_2_, 543.1942; found, 543.1940. Note: Sulfoxide **5c** and sulfone **7c** were obtained from the same reaction mixture by S-oxidation of thioether **3c** followed by separation using column chromatography.

#### *tert*-butyl (2-((2-cyano-4-(pyrrolidin-1-ylsulfonyl)­phenyl)­sulfinyl)­ethyl)­carbamate (**5d**)

Following GP, thioether **3d** (15 mg, 0.036 mmol, 1.0 equiv) was oxidized with mCPBA (12 mg, 0.055 mmol, 1.5 equiv) for 30 min at 0 °C followed by 30 min at room temperature, to afford the title compound (6.0 mg, 39%) as a white solid. The crude was purified by column chromatography (silica 5 g, 0–100% EtOAc/pentane) with the product eluting at 100% EtOAc. ^1^H NMR (800 MHz, CDCl_3_) δ: 8.24 (s, 2H), 8.18 (s, 1H), 5.10 (t, *J* = 6.0 Hz, 1H), 3.69–3.56 (m, 2H), 3.43 (dt, *J* = 13.3, 6.3 Hz, 1H), 3.30 (t, *J* = 6.4 Hz, 4H), 3.11–3.04 (m, 1H), 1.86 (t, *J* = 6.5 Hz, 4H), 1.42 (s, 9H); ^13^C NMR (201 MHz, CDCl_3_) δ: 155.8, 152.2, 141.5, 132.2, 126.7, 113.7, 109.7, 80.3, 55.4, 48.3, 34.9, 28.4, 25.6; HRMS (ESI) *m*/*z*: [M + H]^+^ calcd for C_18_H_26_N_3_O_5_S_2_, 428.1308; found, 543.1307. Note: Sulfoxide **5d** and sulfone **7d** were obtained from the same reaction mixture by S-oxidation of thioether **3d** followed by separation using column chromatography.

#### *tert*-butyl 6-((2-((*tert*-butoxycarbonyl)­amino)­ethyl)­sulfinyl)-5-(trifluoromethyl)­nicotinate (**5f**)

Following GP, thioether **3f** (38 mg, 0.090 mmol, 1.0 equiv) was oxidized with mCPBA (22 mg, 0.099 mmol, 1.1 equiv) for 30 min at 0 °C followed by 30 min at room temperature, to afford the title compound (20 mg, 51%) as a white solid. The crude did not require further purification. ^1^H NMR (600 MHz, CDCl_3_) δ: 9.45 (d, *J* = 1.9 Hz, 1H), 8.55 (d, *J* = 1.9 Hz, 1H), 5.15 (t, *J* = 6.0 Hz, 1H), 3.73–3.60 (m, 2H), 3.30–3.20 (m, 1H), 3.14 (dt, *J* = 13.2, 4.9 Hz, 1H), 1.63 (s, 9H), 1.41 (s, 9H); ^13^C NMR (151 MHz, CDCl_3_) δ: 165.1, 162.0, 155.8, 154.4, 136.2 (q, ^3^ *J*_CF_ = 5 Hz), 129.7, 124.8 (q, ^2^ *J*_CF_ = 34 Hz), 122.4 (q, ^1^ *J*_CF_ = 274 Hz), 84.2, 80.0, 54.5, 34.9, 28.4, 28.2; ^19^F NMR (564 MHz, CDCl_3_) δ: −57.87; HRMS (ESI) *m*/*z*: [M + H]^+^ calcd for C_18_H_26_F_3_N_2_O_5_S, 439.1509; found, 439.1510.

#### *tert*-butyl 6-((2-((*tert*-butoxycarbonyl)­amino)­ethyl)­sulfinyl)-5-nitronicotinate (**5g**)

Following GP, thioether **3g** (130 mg, 0.33 mmol, 1.0 equiv) was oxidized with mCPBA (241 mg, 1.07 mmol, 3.3 equiv) for 30 min at 0 °C followed by 24 h at room temperature, to afford the title compound (27 mg, 20%) as a yellow solid. The crude was purified by column chromatography (high-capacity silica 10 g, 0–100% EtOAc/pentane) with the product eluting at 70% EtOAc. ^1^H NMR (600 MHz, CDCl_3_) δ: 9.55 (d, *J* = 1.8 Hz, 1H), 8.98 (d, *J* = 1.8 Hz, 1H), 5.13 (t, *J* = 6.2 Hz, 1H), 3.75–3.64 (m, 2H), 3.61–3.53 (m, 1H), 3.23–3.14 (m, 1H), 1.64 (s, 9H), 1.36 (s, 9H); ^13^C NMR (151 MHz, CDCl_3_) δ: 165.0, 161.4, 155.7, 155.3, 142.5, 134.4, 130.4, 84.8, 79.9, 53.8, 34.8, 28.4, 28.2; HRMS (ESI) *m*/*z*: [M + H]^+^ calcd for C_17_H_26_N_3_O_7_S, 416.1486; found, 416.1486. Note: The scaffold was difficult to oxidize. The sulfoxide was isolated, but the respective sulfone could not be synthesized due to low stability and decomposition.

#### Methyl 6-((2-((*tert*-butoxycarbonyl)­amino)­ethyl)­sulfinyl)­pyridazine-3-carboxylate (**5k**)

Following GP, thioether **3k** (106 mg, 0.34 mmol, 1.0 equiv) was oxidized with mCPBA (80 mg, 0.36 mmol, 1.05 equiv) for 30 min at 0 °C followed by 15 min at room temperature, to afford the title compound (40 mg, 36%) as a white solid. The crude was purified by column chromatography (silica 5 g, 0–100% EtOAc/pentane, then 0–10% MeOH/EtOAc) with the product eluting at 0–5% MeOH. ^1^H NMR (600 MHz, CDCl_3_) δ: 8.44 (d, *J* = 8.6 Hz, 1H), 8.33 (d, *J* = 8.6 Hz, 1H), 4.99 (s, 1H), 4.12 (s, 3H), 3.73–3.63 (m, 1H), 3.62–3.53 (m, 2H), 3.38–3.29 (m, 1H), 1.40 (s, 9H); ^13^C NMR (151 MHz, CDCl_3_) δ: 171.8, 163.9, 155.6, 151.8, 129.4, 124.5, 80.1, 54.6, 53.9, 34.8, 28.4; HRMS (ESI) *m*/*z*: [M + H]^+^ calcd for C_13_H_20_N_3_O_5_S, 330.1118; found, 330.1117.

#### Methyl 5-((2-((*tert*-butoxycarbonyl)­amino)­ethyl)­sulfinyl)­pyrazine-2-carboxylate (**5l**)

Following GP, thioether **3l** (104 mg, 0.33 mmol, 1.0 equiv) was oxidized with mCPBA (82 mg, 0.36 mmol, 1.1 equiv) for 30 min at 0 °C followed by 30 min at room temperature, to afford the title compound (59 mg, 54%) as a white solid. The crude was purified by column chromatography (silica 5 g, 0–100% EtOAc/pentane) with the product eluting at 100% EtOAc. ^1^H NMR (600 MHz, CDCl_3_) δ: 9.27–9.22 (m, 2H), 5.07 (t, *J* = 6.1 Hz, 1H), 4.04 (s, 3H), 3.67–3.50 (m, 2H), 3.48–3.39 (m, 1H), 3.32–3.21 (m, 1H), 1.33 (s, 9H); ^13^C NMR (151 MHz, CDCl_3_) δ: 164.1, 163.7, 155.6, 145.1, 143.7, 141.7, 79.9, 53.6, 53.3, 34.3, 28.3; HRMS (ESI) *m*/*z*: [M + H]^+^ calcd for C_13_H_20_N_3_O_5_S, 330.1118; found, 330.1119. Note: Sulfoxide **5l** and sulfone **7l** were obtained from the same reaction mixture by S-oxidation of thioether **3l** followed by separation using column chromatography.

#### Methyl 2-((2-((*tert*-butoxycarbonyl)­amino)­ethyl)­sulfinyl)­thiazole-5-carboxylate (**5m**)

Following GP, thioether **3m** (134 mg, 0.42 mmol, 1.0 equiv) was oxidized with mCPBA (104 mg, 0.46 mmol, 1.1 equiv) for 30 min at 0 °C followed by 15 min at room temperature, to afford the title compound (47 mg, 33%) as a white solid. The crude was purified by column chromatography (high-capacity silica 5 g, 0–50% EtOAc/pentane) with the product eluting at 50% EtOAc. ^1^H NMR (600 MHz, CDCl_3_) δ: 8.45 (s, 1H), 5.09 (t, *J* = 6.0 Hz, 1H), 3.92 (s, 3H), 3.72–3.62 (m, 1H), 3.62–3.53 (m, 1H), 3.51–3.42 (m, 1H), 3.35–3.28 (m, 1H), 1.39 (s, 9H); ^13^C NMR (151 MHz, CDCl_3_) δ: 169.8, 160.4, 155.6, 149.4, 135.6, 80.3, 55.0, 53.4, 34.8, 28.4; HRMS (ESI) *m*/*z*: [M + Na]^+^ calcd for C_12_H_18_N_2_NaO_5_S_2_, 357.0549; found, 357.0549. Note: Sulfoxide **5m** and sulfone **7m** were obtained from the same reaction mixture by S-oxidation of thioether **3m** followed by separation using column chromatography.

### Synthesis and Characterization of Aryl Sulfones (**6a-6o**, **7a-7o**)

#### *tert*-butyl 4-((2-methoxy-2-oxoethyl)­sulfonyl)-3-(trifluoromethyl)­benzoate (**6a**)

Following GP, thioether **2a** (60 mg, 0.17 mmol, 1.0 equiv) was oxidized with mCPBA (191 mg, 0.85 mmol, 5.0 equiv) for 30 min at 0 °C followed by warming the reaction mixture to room temperature and subsequently heating at 50 °C for 24 h, to afford the title compound (65 mg, 100%) as a white crystalline solid. The crude did not require further purification. Rf = 0.44 (EtOAc/pentane 1:4). ^1^H NMR (600 MHz, CDCl_3_) δ: 8.48 (s, 1H), 8.35–8.31 (m, 2H), 4.32 (s, 2H), 3.69 (s, 3H), 1.62 (s, 9H); ^13^C NMR (151 MHz, CDCl_3_) δ: 162.8, 162.6, 140.2, 137.6, 134.3, 133.1, 129.4 (q, ^3^ *J*_CF_ = 7 Hz), 129.1 (q, ^2^ *J*_CF_ = 34 Hz), 122.4 (q, ^1^ *J*_CF_ = 274 Hz), 83.6, 61.0, 53.4, 28.1; ^19^F NMR (564 MHz, CDCl_3_) δ: −56.80; HRMS (ESI) *m*/*z*: [M-H]^−^ calcd for C_15_H_16_F_3_O_6_S, 381.0625; found, 381.0629.

#### *tert*-butyl 4-((2-methoxy-2-oxoethyl)­sulfonyl)-3-nitrobenzoate (**6b**)

Following GP, thioether **2b** (59 mg, 0.18 mmol, 1.0 equiv) was oxidized with mCPBA (201 mg, 0.90 mmol, 5.0 equiv) for 30 min at 0 °C followed by warming the reaction mixture to room temperature and subsequently heating at 50 °C for 48 h, to afford the title compound (52 mg, 81%) as a clear oil. The crude was purified by column chromatography (silica 5 g, 0–40% EtOAc/pentane) with the product eluting at 30% EtOAc. Rf = 0.68 (EtOAc/pentane 3:7). ^1^H NMR (600 MHz, CDCl_3_) δ: 8.39 (d, *J* = 1.6 Hz, 1H), 8.35 (dd, *J* = 8.2, 1.6 Hz, 1H), 8.25 (d, *J* = 8.1 Hz, 1H), 4.46 (s, 2H), 3.74 (s, 3H), 1.61 (s, 9H); ^13^C NMR (151 MHz, CDCl_3_) δ: 162.9, 162.0, 149.1, 138.8, 135.3, 133.8, 133.0, 125.8, 84.1, 60.7, 53.4, 28.1; HRMS (ESI) *m*/*z*: [M-H]^−^ calcd for C_14_H_16_NO_8_S, 358.0602; found, 358.0603.

#### Methyl 6-((2-(*tert*-butoxy)-2-oxoethyl)­sulfonyl)­nicotinate (**6e**)

Following GP, thioether **2e** (206 mg, 0.73 mmol, 1.0 equiv) was oxidized with mCPBA (407 mg, 1.82 mmol, 2.5 equiv) for 30 min at 0 °C followed by 4 h at room temperature, to afford the title compound (225 mg, 98%) as a white crystalline solid. The crude did not require further purification. ^1^H NMR (600 MHz, CDCl_3_) δ: 9.26 (d, *J* = 1.2 Hz, 1H), 8.54 (dd, *J* = 8.1, 2.1 Hz, 1H), 8.14 (d, *J* = 9.1 Hz, 1H), 4.41 (s, 2H), 3.97 (s, 3H), 1.27 (s, 9H); ^13^C NMR (151 MHz, CDCl_3_) δ: 164.1, 161.2, 159.9, 151.1, 139.4, 129.2, 121.8, 83.9, 56.9, 53.1, 27.7; HRMS (ESI) *m*/*z*: [M + H]^+^ calcd for C_13_H_18_NO_8_S, 316.0849; found, 316.0850.

#### *tert*-butyl 6-((2-methoxy-2-oxoethyl)­sulfonyl)-5-(trifluoromethyl)­nicotinate (**6f**)

Following GP, thioether **2f** (26 mg, 0.074 mmol, 1.0 equiv) was oxidized with mCPBA (58 mg, 0.26 mmol, 3.5 equiv) for 30 min at 0 °C followed by warming the reaction mixture to room temperature and subsequently heating at 50 °C for 24 h, to afford the title compound (10 mg, 36%) as a pale-yellow oil. The crude was purified by column chromatography (high-capacity silica 5 g, 50–100% DCM/pentane) with the product eluting at 100% DCM. Rf = 0.36 (DCM). ^1^H NMR (600 MHz, CDCl_3_) δ: 9.27 (d, *J* = 1.9 Hz, 1H), 8.74 (d, *J* = 2.0 Hz, 1H), 4.71 (s, 2H), 3.71 (s, 3H), 1.64 (s, 9H); ^13^C NMR (151 MHz, CDCl_3_) δ: 163.2, 161.5, 158.0, 151.8, 138.3 (q, ^3^ *J*_CF_ = 6 Hz), 130.8, 125.1 (q, ^2^ *J*_CF_ = 37 Hz), 121.5 (q, ^1^ *J*_CF_ = 275 Hz), 84.6, 56.3, 53.3, 28.2; ^19^F NMR (564 MHz, CDCl_3_) δ: −58.09; HRMS (ESI) *m*/*z*: [M + H]^+^ calcd for C_14_H_17_F_3_NO_6_S, 384.0723; found, 384.0719.

#### *tert*-butyl 6-((2-methoxy-2-oxoethyl)­sulfonyl)-5-nitronicotinate (**6g**)

Following GP, thioether **2g** (44 mg, 0.13 mmol, 1.0 equiv) was oxidized with mCPBA (74 mg, 0.33 mmol, 2.5 equiv) for 30 min at 0 °C followed by warming the reaction mixture to room temperature and subsequently heating at 50 °C for 24 h, to afford the title compound (12 mg, 25%) as a clear oil. The crude was purified by column chromatography (silica 5 g, 0–40% EtOAc/pentane) with the product eluting at 40% EtOAc. Rf = 0.50 (EtOAc/pentane 3:7). Further purification by preparative-HPLC (C18 column, 20–80% MeCN/water with 0.1% TFA) was performed to remove a residual impurity. ^1^H NMR (600 MHz, CDCl_3_) δ: 9.31 (d, *J* = 1.7 Hz, 1H), 8.72 (d, *J* = 1.7 Hz, 1H), 4.69 (s, 2H), 3.72 (s, 3H), 1.64 (s, 9H); ^13^C NMR (151 MHz, CDCl_3_) δ: 162.8, 160.7, 151.9, 151.8, 144.9, 134.6, 132.2, 85.2, 56.8, 53.4, 28.2; HRMS (ESI) *m*/*z*: [M + H]^+^ calcd for C_13_H_17_N_2_O_8_S, 361.0700; found, 361.0690.

#### Methyl 2-((2-(*tert*-butoxy)-2-oxoethyl)­sulfonyl)­pyrimidine-5-carboxylate (**6h**)

Following GP, thioether **2h** (40 mg, 0.14 mmol, 1.0 equiv) was oxidized with mCPBA (78 mg, 0.35 mmol, 2.5 equiv) for 30 min at 0 °C followed by 24 h at room temperature, to afford the title compound (44 mg, 100%) as a white crystalline solid. The crude did not require further purification. ^1^H NMR (600 MHz, CDCl_3_) δ: 9.44 (s, 2H), 4.55 (s, 2H), 4.04 (s, 3H), 1.32 (s, 9H); ^13^C NMR (151 MHz, CDCl_3_) δ: 167.5, 162.6, 161.2, 159.7, 126.1, 84.4, 53.5, 27.8; HRMS (ESI) *m*/*z*: [M + NH_4_]^+^ calcd for C_12_H_20_N_3_O_6_S, 334.1067; found, 334.1059.

#### Methyl 2-((2-methoxy-2-oxoethyl)­sulfonyl)­pyrimidine-4-carboxylate (**6i**)

Following GP, thioether **2i** (15 mg, 0.061 mmol, 1.0 equiv) was oxidized with mCPBA (34 mg, 0.15 mmol, 2.5 equiv) for 30 min at 0 °C followed by 48 h at room temperature, to afford the title compound (5.4 mg, 32%) as a pale-yellow oil. The crude was purified by column chromatography (high-capacity silica 5 g, 30–90% EtOAc/pentane) with the product eluting at 70% EtOAc. Rf = 0.36 (EtOAc/pentane 3:2). ^1^H NMR (600 MHz, CDCl_3_) δ: 9.19 (d, *J* = 4.9 Hz, 1H), 8.21 (d, *J* = 4.9 Hz, 1H), 4.66 (s, 2H), 4.05 (s, 3H), 3.71 (s, 3H); ^13^C NMR (151 MHz, CDCl_3_) δ: 165.6, 163.1, 163.0, 161.0, 156.7, 123.4, 55.3, 53.9, 53.4; HRMS (ESI) *m*/*z*: [M + H]^+^ calcd for C_9_H_11_N_2_O_6_S, 275.0332; found, 275.0330.

#### Methyl 3-(2-((2-(*tert*-butoxy)-2-oxoethyl)­sulfonyl)­pyrimidin-5-yl)­propanoate (**6j**)

Following GP, thioether **2j** (90 mg, 0.29 mmol, 1.0 equiv) was oxidized with mCPBA (162 mg, 0.72 mmol, 2.5 equiv) for 30 min at 0 °C followed by 16 h at room temperature, to afford the title compound (77 mg, 78%) as a white crystalline solid. The crude was purified by column chromatography (silica 10 g, 0–70% EtOAc/pentane) with the product eluting at 40% EtOAc. Rf = 0.38 (EtOAc/pentane 2:3). ^1^H NMR (600 MHz, CDCl_3_) δ: 8.79 (s, 2H), 4.46 (s, 2H), 3.63 (s, 3H), 3.04 (t, *J* = 7.1 Hz, 2H), 2.70 (t, *J* = 7.1 Hz, 2H), 1.27 (s, 9H); ^13^C NMR (151 MHz, CDCl_3_) δ: 171.8, 163.5, 161.2, 158.5, 137.1, 83.9, 56.7, 52.1, 34.0, 27.7, 25.3; HRMS (ESI) *m*/*z*: [M + H]^+^ calcd for C_14_H_21_N_2_O_6_S, 345.1115; found, 345.1118.

#### Methyl 6-((2-methoxy-2-oxoethyl)­sulfonyl)­pyridazine-3-carboxylate (**6k**)

Following GP, thioether **2k** (49 mg, 0.20 mmol, 1.0 equiv) was oxidized with mCPBA (226 mg, 1.01 mmol, 5.0 equiv) for 30 min at 0 °C followed by 24 h at room temperature, to afford the title compound (11 mg, 19%) as a clear oil. The crude was purified by column chromatography (high-capacity silica 5 g, 0–100% EtOAc/pentane) with the product eluting at 75% EtOAc. Rf = 0.79 (EtOAc/pentane 3:2). ^1^H NMR (600 MHz, CDCl_3_) δ: 8.47 (d, *J* = 8.5 Hz, 1H), 8.35 (d, *J* = 8.7 Hz, 1H), 4.74 (s, 2H), 4.13 (s, 3H), 3.68 (s, 3H); ^13^C NMR (151 MHz, CDCl_3_) δ: 163.3, 162.9, 162.8, 153.3, 129.7, 126.0, 56.1, 54.0, 53.4; HRMS (ESI) *m*/*z*: [M + H]^+^ calcd for C_9_H_11_N_2_O_6_S, 275.0332; found, 275.0330.

#### Methyl 5-((2-methoxy-2-oxoethyl)­sulfonyl)­pyrazine-2-carboxylate (**6l**)

Following GP, thioether **2l** (42 mg, 0.17 mmol, 1.0 equiv) was oxidized with mCPBA (96 mg, 0.43 mmol, 2.5 equiv) for 30 min at 0 °C followed by 24 h at room temperature, to afford the title compound (19 mg, 40%) as a clear oil. The crude was purified by column chromatography (silica 5 g, 0–100% EtOAc/pentane) with the product eluting at 80% EtOAc. Rf = 0.44 (EtOAc/pentane 3:2). ^1^H NMR (800 MHz, CDCl_3_) δ: 9.41–9.35 (m, 2H), 4.53 (s, 2H), 4.10 (s, 3H), 3.70 (s, 3H); ^13^C NMR (201 MHz, CDCl_3_) δ: 163.0, 162.6, 154.5, 146.2, 145.7, 142.9, 56.1, 53.9, 53.5; HRMS (ESI) *m*/*z*: [M + H]^+^ calcd for C_9_H_11_N_2_O_6_S, 275.0332; found, 275.0330.

#### Methyl 2-((2-methoxy-2-oxoethyl)­sulfonyl)­thiazole-5-carboxylate (**6m**)

Following GP, thioether **2m** (41 mg, 0.16 mmol, 1.0 equiv) was oxidized with mCPBA (92 mg, 0.41 mmol, 2.5 equiv) for 30 min at 0 °C followed by 30 h at room temperature, to afford the title compound (23 mg, 50%) as a clear oil. The crude was purified by column chromatography (high-capacity silica 5 g, 20–50% EtOAc/pentane) with the product eluting at 40% EtOAc. ^1^H NMR (800 MHz, CDCl_3_) δ: 8.54 (s, 1H), 4.49 (s, 2H), 3.97 (s, 3H), 3.74 (s, 3H); ^13^C NMR (201 MHz, CDCl_3_) δ: 168.6, 162.1, 160.4, 149.2, 136.0, 58.4, 53.6, 53.4; HRMS (ESI) *m*/*z*: [M + H]^+^ calcd for C_8_H_10_NO_6_S_2_, 279.9944; found, 279.9939.

#### Ethyl 6-((2-(*tert*-butoxy)-2-oxoethyl)­sulfonyl)-5-cyano-2-methylnicotinate (**6n**)

Following GP, thioether **2n** (60 mg, 0.18 mmol, 1.0 equiv) was oxidized with mCPBA (101 mg, 0.45 mmol, 2.5 equiv) for 30 min at 0 °C followed by 48 h at room temperature, to afford the title compound (22 mg, 34%) as an orange crystalline solid. The crude was purified by column chromatography (high-capacity silica 5 g, 0–40% EtOAc/pentane) with the product eluting at 35% EtOAc. Rf = 0.62 (EtOAc/pentane 3:7). ^1^H NMR (600 MHz, CDCl_3_) δ: 8.69 (s, 1H), 4.50 (s, 2H), 4.46 (q, *J* = 7.1 Hz, 2H), 2.97 (s, 3H), 1.44 (t, *J* = 7.1 Hz, 3H), 1.37 (s, 9H); ^13^C NMR (151 MHz, CDCl_3_) δ: 164.2, 163.3, 161.2, 158.4, 145.7, 128.7, 112.9, 105.8, 84.6, 63.0, 57.0, 27.8, 25.3, 14.3; LC–MS (ESI) *m*/*z*: [M + H-*t*Bu]^+^ calcd for C_12_H_13_N_2_O_6_S, 313.05; found, 313.11 (fragmentation of *t*-Bu group).

#### *tert*-butyl 4-((4-((2-methoxy-2-oxoethyl)­sulfonyl)-2-(trifluoromethyl)­phenyl)­sulfonyl) Piperazine-1-carboxylate (**6o**)

Following GP, thioether **2o** (9.5 mg, 0.019 mmol, 1.0 equiv) was oxidized with mCPBA (11 mg, 0.048 mmol, 2.5 equiv) for 30 min at 0 °C followed by 24 h at room temperature, to afford the title compound (8.7 mg, 86%) as a white solid. The crude did not require further purification. Rf = 0.66 (EtOAc/pentane 2:3). ^1^H NMR (600 MHz, CDCl_3_) δ: 8.44 (d, *J* = 1.8 Hz, 1H), 8.35–8.27 (m, 2H), 4.21 (s, 2H), 3.75 (s, 3H), 3.52 (t, *J* = 5.0 Hz, 4H), 3.27 (t, *J* = 5.0 Hz, 4H), 1.44 (s, 9H); ^13^C NMR (151 MHz, CDCl_3_) δ: 162.4, 154.3, 143.4, 143.0, 133.0, 132.9, 129.4 (q, ^2^ *J*_CF_ = 34 Hz), 129.2 (q, ^3^ *J*_CF_ = 7 Hz), 121.7 (q, ^1^ *J*_CF_ = 275 Hz), 80.8, 60.4, 53.6, 45.8, 44.1, 43.0, 28.5; ^19^F NMR (564 MHz, CDCl_3_) δ: −57.69; HRMS (ESI) *m*/*z*: [M + NH_4_]^+^ calcd for C_19_H_29_F_3_N_3_O_8_S_2_, 548.1343; found, 548.1344.

#### *tert*-butyl 4-((2-((*tert*-butoxycarbonyl)­amino)­ethyl)­sulfonyl)-3-(trifluoromethyl)-benzoate (**7a**)

Following GP, sulfoxide **5a** (100 mg, 0.23 mmol, 1.0 equiv) was oxidized with mCPBA (154 mg, 0.69 mmol, 3.0 equiv) for 30 min at 0 °C followed by warming the reaction mixture to room temperature and subsequently heating at 50 °C for 6 h, to afford the title compound (79 mg, 76%) as a clear oil. The crude was purified by column chromatography (silica 5 g, 0–50% EtOAc/pentane) with the product eluting at 45% EtOAc. ^1^H NMR (600 MHz, CDCl_3_) δ: 8.46 (d, *J* = 1.4 Hz, 1H), 8.35–8.27 (m, 2H), 5.13 (t, *J* = 6.4 Hz, 1H), 3.60 (q, *J* = 5.9 Hz, 2H), 3.49–3.45 (m, 2H), 1.61 (s, 9H), 1.38 (s, 9H); ^13^C NMR (151 MHz, CDCl_3_) δ: 162.8, 155.6, 141.3, 137.3, 133.5, 133.1, 129.5 (q, ^3^ *J*_CF_ = 6 Hz), 129.1 (q, ^2^ *J*_CF_ = 34 Hz), 122.3 (q, ^1^ *J*_CF_ = 274 Hz), 83.5, 80.1, 56.6, 34.7, 28.4, 28.1; ^19^F NMR (564 MHz, CDCl_3_) δ: −56.73; HRMS (ESI) *m*/*z*: [M + NH_4_]^+^ calcd for C_19_H_30_F_3_N_2_O_6_S, 471.1771; found, 471.1771. Note: This sulfone was obtained by S-oxidation of the isolated sulfoxide instead of the thioether.

#### Methyl 4-((2-((*tert*-butoxycarbonyl)­amino)­ethyl)­sulfonyl)-3-nitrobenzoate (**7b**)

Following GP, sulfoxide **5b** (70 mg, 0.19 mmol, 1.0 equiv) was oxidized with mCPBA (126 mg, 0.56 mmol, 3.0 equiv) for 30 min at 0 °C followed by warming the reaction mixture to room temperature and subsequently heating at 50 °C for 6 h, to afford the title compound (40 mg, 55%) as a yellow solid. The crude was purified by column chromatography (high-capacity silica 5 g, 0–65% EtOAc/pentane) with the product eluting at 65% EtOAc. ^1^H NMR (800 MHz, CDCl_3_) δ: 8.43 (s, 1H), 8.39 (d, *J* = 8.1 Hz, 1H), 8.24 (d, *J* = 8.1 Hz, 1H), 5.14 (t, *J* = 6.3 Hz, 1H), 4.00 (s, 3H), 3.81 (t, *J* = 5.9 Hz, 2H), 3.69 (q, *J* = 6.2 Hz, 2H), 1.40 (s, 9H); ^13^C NMR (201 MHz, CDCl_3_) δ: 163.5, 155.7, 149.2, 136.7, 136.5, 133.5, 132.5, 126.0, 80.3, 56.9, 53.5, 34.7, 28.4; HRMS (ESI) *m*/*z*: [M + NH_4_]^+^ calcd for C_15_H_24_N_3_O_8_S, 406.1279; found, 406.1278. Note: This sulfone was obtained by S-oxidation of the isolated sulfoxide instead of the thioether.

#### *tert*-butyl 4-((4-((2-((*tert*-butoxycarbonyl)­amino)­ethyl)­sulfonyl)-3-cyanophenyl)­sulfonyl)­piperazine-1-carboxylate (**7c**)

Following GP, thioether **3c** (80 mg, 0.15 mmol, 1.0 equiv) was oxidized with mCPBA (51 mg, 0.23 mmol, 1.5 equiv) for 30 min at 0 °C followed by 30 min at room temperature, to afford the title compound (7.2 mg, 8%) as a white solid. The crude was purified by column chromatography (silica 5 g, 0–70% EtOAc/pentane) with the product eluting at 60% EtOAc. ^1^H NMR (600 MHz, CDCl_3_) δ: 8.35 (d, *J* = 8.2 Hz, 1H), 8.23 (d, *J* = 1.7 Hz, 1H), 8.13 (dd, *J* = 8.2, 1.8 Hz, 1H), 5.03 (s, 1H), 3.68–3.62 (m, 4H), 3.55 (t, *J* = 5.0 Hz, 4H), 3.08 (t, *J* = 5.1 Hz, 4H), 1.42 (s, 9H), 1.39 (s, 9H); ^13^C NMR (151 MHz, CDCl_3_) δ: 155.6, 154.1, 145.4, 142.7, 134.0, 132.0, 131.7, 114.3, 113.2, 81.0, 80.6, 55.2, 46.0, 43.6, 42.7, 34.7, 28.4, 28.4; HRMS (ESI) *m*/*z*: [M + H]^+^ calcd for C_23_H_35_N_4_O_8_S_2_, 559.1891; found, 559.1889. Note: Sulfoxide **5c** and sulfone **7c** were obtained from the same reaction mixture by S-oxidation of thioether **3c** followed by separation using column chromatography.

#### *tert*-butyl (2-((2-cyano-4-(pyrrolidin-1-ylsulfonyl)­phenyl)­sulfonyl)­ethyl)­carbamate (**7d**)

Following GP, thioether **3d** (15 mg, 0.036 mmol, 1.0 equiv) was oxidized with mCPBA (12 mg, 0.055 mmol, 1.5 equiv) for 30 min at 0 °C followed by 30 min at room temperature, to afford the title compound (6.0 mg, 37%) as a white solid. The crude was purified by column chromatography (silica 5 g, 0–100% EtOAc/pentane) with the product eluting at 70% EtOAc. ^1^H NMR (800 MHz, CDCl_3_) δ: 8.35–8.29 (m, 2H), 8.21 (dd, *J* = 8.2, 1.8 Hz, 1H), 5.03 (s, 1H), 3.70–3.61 (m, 4H), 3.34–3.29 (m, 4H), 1.89–1.85 (m, 4H), 1.39 (s, 9H); ^13^C NMR (201 MHz, CDCl_3_) δ: 155.6, 144.8, 143.9, 133.8, 131.7, 131.6, 114.5, 112.9, 80.5, 55.2, 48.3, 34.7, 28.4, 25.6; HRMS (ESI) *m*/*z*: [M + NH_4_]^+^ calcd for C_18_H_29_N_4_O_6_S_2_, 461.1523; found, 461.1519. Note: Sulfoxide **5d** and sulfone **7d** were obtained from the same reaction mixture by S-oxidation of thioether **3d** followed by separation using column chromatography.

#### *tert*-butyl 6-((2-((*tert*-butoxycarbonyl)­amino)­ethyl)­sulfonyl)-5-(trifluoromethyl)­nicotinate (**7f**)

Following GP, sulfoxide **5f** (20 mg, 0.046 mmol, 1.0 equiv) was oxidized with mCPBA (31 mg, 0.14 mmol, 3.0 equiv) for 30 min at 0 °C followed by warming the reaction mixture to room temperature and subsequently heating at 50 °C for 16 h, to afford the title compound (20 mg, 95%) as a clear oil. The crude did not require further purification. ^1^H NMR (600 MHz, CDCl_3_) 9.28 (d, *J* = 1.9 Hz, 1H), 8.73 (d, *J* = 1.9 Hz, 1H), 5.16 (t, *J* = 6.2 Hz, 1H), 3.88 (t, *J* = 5.8 Hz, 2H), 3.71 (q, *J* = 6.0 Hz, 2H), 1.63 (s, 9H), 1.40 (s, 9H); ^13^C NMR (151 MHz, CDCl_3_) δ: 161.5, 158.7, 155.7, 151.9, 138.4 (q, ^3^ *J*_CF_ = 6 Hz), 130.7, 125.1 (q, ^2^ *J*_CF_ = 37 Hz), 121.6 (d, ^1^ *J*_CF_ = 275 Hz), 84.6, 80.1, 53.1, 34.9, 28.4, 28.2; ^19^F NMR (564 MHz, CDCl_3_) δ: −57.89; HRMS (ESI) *m*/*z*: [M + H]^+^ calcd for C_18_H_26_F_3_N_2_O_6_S, 455.1458; found, 455.1458. Note: This sulfone was obtained by S-oxidation of the isolated sulfoxide instead of the thioether.

#### Methyl 6-((2-((*tert*-butoxycarbonyl)­amino)­ethyl)­sulfonyl)­pyridazine-3-carboxylate (**7k**)

Following GP, thioether **3k** (40 mg, 0.13 mmol, 1.0 equiv) was oxidized with mCPBA (72 mg, 0.32 mmol, 2.5 equiv) for 30 min at 0 °C followed by 16 h at room temperature, to afford the title compound (9.3 mg, 21%) as a white solid. The crude was purified by column chromatography (silica 5 g, 0–100% EtOAc/pentane) with the product eluting at 75% EtOAc. ^1^H NMR (800 MHz, CDCl_3_) δ: 8.47 (d, *J* = 8.6 Hz, 1H), 8.34 (d, *J* = 8.7 Hz, 1H), 5.11 (t, *J* = 6.5 Hz, 1H), 4.13 (s, 3H), 3.87 (t, *J* = 5.8 Hz, 2H), 3.74 (q, *J* = 6.2 Hz, 2H), 1.40 (s, 9H); ^13^C NMR (201 MHz, CDCl_3_) δ: 163.6, 163.3, 155.6, 153.3, 129.9, 125.5, 80.3, 54.1, 52.9, 34.7, 28.4; HRMS (ESI) *m*/*z*: [M + NH_4_]^+^ calcd for C_13_H_23_N_4_O_6_S, 363.1333; found, 363.1333.

#### Methyl 5-((2-((*tert*-butoxycarbonyl)­amino)­ethyl)­sulfonyl)­pyrazine-2-carboxylate (**7l**)

Following GP, thioether **3l** (104 mg, 0.33 mmol, 1.0 equiv) was oxidized with mCPBA (82 mg, 0.36 mmol, 1.1 equiv) for 30 min at 0 °C followed by 30 min at room temperature, to afford the title compound (7.9 mg, 7%) as a white solid. The crude was purified by column chromatography (silica 5 g, 0–100% EtOAc/pentane) with the product eluting at 50% EtOAc. ^1^H NMR (600 MHz, CDCl_3_) δ: 9.37 (s, 2H), 5.06 (s, 1H), 4.10 (s, 3H), 3.72–3.60 (m, 4H), 1.39 (s, 9H); ^13^C NMR (151 MHz, CDCl_3_) δ: 163.0, 155.6, 155.3, 146.2, 145.7, 142.5, 80.4, 53.9, 52.7, 34.7, 28.4; HRMS (ESI) *m*/*z*: [M + NH_4_]^+^ calcd for C_13_H_23_N_4_O_6_S, 363.1333; found, 363.1332. Note: Sulfoxide **5l** and sulfone **7l** were obtained from the same reaction mixture by S-oxidation of thioether **3l** followed by separation using column chromatography.

#### Methyl 2-((2-((*tert*-butoxycarbonyl)­amino)­ethyl)­sulfonyl)­thiazole-5-carboxylate (**7m**)

Following GP, thioether **3m** (134 mg, 0.42 mmol, 1.0 equiv) was oxidized with mCPBA (104 mg, 0.46 mmol, 1.1 equiv) for 30 min at 0 °C followed by 15 min at room temperature, to afford the title compound (4.5 mg, 3%) as a white solid. The crude was purified by column chromatography (high-capacity silica 5 g, 0–50% EtOAc/pentane) with the product eluting at 50% EtOAc ^1^H NMR (600 MHz, CDCl_3_) δ: 8.53 (s, 1H), 5.12 (s, 1H), 3.97 (s, 3H), 3.71–3.64 (m, 4H), 1.41 (s, 9H); ^13^C NMR (151 MHz, CDCl_3_) δ: 169.8, 160.4, 155.6, 149.4, 135.6, 80.3, 55.0, 53.4, 34.8, 28.4; HRMS (ESI) *m*/*z*: [M + H]^+^ calcd for C_12_H_19_N_2_O_6_S_2_, 351.0679; found, 351.0678. Note: Sulfoxide **5m** and sulfone **7m** were obtained from the same reaction mixture by S-oxidation of thioether **3m** followed by separation using column chromatography.

#### *tert*-butyl 4-((4-((2-((*tert*-butoxycarbonyl)­amino)­ethyl)­sulfonyl)-2-(trifluoromethyl)-phenyl) sulfonyl)­piperazine-1-carboxylate (**7o**)

Following GP, thioether **3o** (38 mg, 0.067 mmol, 1.0 equiv) was oxidized with mCPBA (30 mg, 0.13 mmol, 2.0 equiv) for 30 min at 0 °C followed by 30 min at room temperature, to afford the title compound (14 mg, 35%) as a white solid. The crude did not require further purification. ^1^H NMR (600 MHz, CDCl_3_) δ: 8.39 (d, *J* = 1.9 Hz, 1H), 8.32 (d, *J* = 8.3 Hz, 1H), 8.24 (dd, *J* = 8.3, 1.9 Hz, 1H), 5.06 (t, *J* = 6.2 Hz, 1H), 3.61 (q, *J* = 6.0 Hz, 2H), 3.54–3.49 (m, 4H), 3.43 (t, *J* = 6.0 Hz, 2H), 3.29–3.23 (m, 4H), 1.44 (s, 9H), 1.39 (s, 9H); ^13^C NMR (151 MHz, CDCl_3_) δ: 155.6, 154.3, 143.9, 143.2, 133.4, 131.9, 129.8 (q, ^2^ *J*_CF_ = 35 Hz), 128.4 (q, ^3^ *J*_CF_ = 7 Hz), 121.6 (q, ^1^ *J*_CF_ = 275 Hz), 80.8, 80.5, 55.8, 45.8, 44.1, 43.0, 34.8, 28.5, 28.4; ^19^F NMR (564 MHz, CDCl_3_) δ: −57.65; HRMS (ESI) *m*/*z*: [M + H]^+^ calcd for C_23_H_35_F_3_N_3_O_8_S_2_, 602.1812; found, 602.1809. Note: The corresponding sulfoxide was not isolated due to rapid overoxidation to the sulfone.

### Synthesis and Characterization of Warheads with Triazole Linker

#### *tert*-butyl 4-(((1*H*-1,2,3-triazol-1-yl)­methyl)­sulfinyl)-3-nitrobenzoate (**13**)

Following GP, thioether **18** (49 mg, 0.15 mmol, 1.0 equiv) was oxidized with mCPBA (36 mg, 0.16 mmol, 1.1 equiv) for 30 min at 0 °C followed by 30 min at room temperature, to afford the title compound (36 mg, 71%) as a pale-yellow crystalline solid. The crude was purified by column chromatography (high-capacity silica 5 g, 0–50% EtOAc/pentane) with the product eluting at 50% EtOAc. Rf = 0.31 (EtOAc/pentane 3:2). ^1^H NMR (600 MHz, CDCl_3_) δ: 8.83 (d, *J* = 1.6 Hz, 1H), 8.20 (dd, *J* = 8.1, 1.6 Hz, 1H), 7.89 (d, *J* = 1.1 Hz, 1H), 7.62 (d, *J* = 1.2 Hz, 1H), 7.50 (d, *J* = 8.1 Hz, 1H), 6.06 (d, *J* = 13.4 Hz, 1H), 5.61 (d, *J* = 13.4 Hz, 1H), 1.61 (s, 9H); ^13^C NMR (151 MHz, CDCl_3_) δ: 162.4, 145.2, 141.9, 136.8, 135.4, 133.7, 127.3, 126.4, 126.2, 83.6, 67.2, 28.1; HRMS (ESI) *m*/*z*: [M + H]^+^ calcd for C_14_H_17_N_4_O_5_S, 353.0914; found, 353.0912.

#### *tert*-butyl 4-(((1*H*-1,2,3-triazol-1-yl)­methyl)­sulfonyl)-3-nitrobenzoate (**14**)

Following GP, thioether **18** (46 mg, 0.14 mmol, 1.0 equiv) was oxidized with mCPBA (77 mg, 0.34 mmol, 2.5 equiv) for 30 min at 0 °C followed by warming the reaction mixture to room temperature and subsequently heating at 50 °C for 24 h, to afford the title compound (36 mg, 70%) as a white crystalline solid. The crude was purified by column chromatography (high-capacity silica 5 g, 0–75% EtOAc/pentane) with the product eluting at 50% EtOAc. Rf = 0.67 (EtOAc/pentane 3:2). ^1^H NMR (600 MHz, CDCl_3_) δ: 8.43 (d, *J* = 1.6 Hz, 1H), 8.16 (dd, *J* = 8.1, 1.6 Hz, 1H), 7.93 (d, *J* = 1.2 Hz, 1H), 7.75 (d, *J* = 1.2 Hz, 1H), 7.59 (d, *J* = 8.2 Hz, 1H), 6.22 (s, 2H), 1.60 (s, 9H); ^13^C NMR (151 MHz, CDCl_3_) δ: 161.7, 149.2, 139.6, 134.9, 133.4, 133.1, 132.5, 126.3, 125.9, 84.3, 68.5, 28.1; HRMS (ESI) *m*/*z*: [M + H]^+^ calcd for C_14_H_17_N_4_O_6_S, 369.0863; found, 369.0860.

#### *tert*-butyl 4-(((1*H*-1,2,3-triazol-4-yl)­methyl)­sulfinyl)-3-nitrobenzoate (**15**)

Following GP, thioether **20** (51 mg, 0.15 mmol, 1.0 equiv) was oxidized with mCPBA (37 mg, 0.17 mmol, 1.1 equiv) for 30 min at 0 °C followed by 30 min at room temperature, to afford the title compound (40 mg, 76%) as a pale-yellow crystalline solid. The crude did not require further purification. Rf = 0.17 (EtOAc/DCM 3:7). ^1^H NMR (600 MHz, CDCl_3_) δ: 8.82 (d, *J* = 1.6 Hz, 1H), 8.29 (dd, *J* = 8.1, 1.7 Hz, 1H), 7.77 (d, *J* = 8.1 Hz, 1H), 7.63 (s, 1H), 4.62 (d, *J* = 13.9 Hz, 1H), 4.48 (d, *J* = 14.0 Hz, 1H), 1.61 (s, 9H); ^13^C NMR (151 MHz, CDCl_3_) δ: 162.7, 145.0, 136.1, 135.4, 135.3, 132.1, 127.8, 126.2, 83.6, 50.8, 28.2; HRMS (ESI) *m*/*z*: [M + H]^+^ calcd for C_14_H_17_N_4_O_5_S, 353.0914; found, 353.0909.

#### *tert*-butyl 4-(((1*H*-1,2,3-triazol-4-yl)­methyl)­sulfonyl)-3-nitrobenzoate (**16**)

Following GP, thioether **20** (57 mg, 0.17 mmol, 1.0 equiv) was oxidized with mCPBA (95 mg, 0.42 mmol, 2.5 equiv) for 30 min at 0 °C followed by warming the reaction mixture to room temperature and subsequently heating at 50 °C for 24 h, to afford the title compound (26 mg, 41%) as a white crystalline solid. The crude was purified by column chromatography (high-capacity silica 5 g, 50–100% DCM/pentane, then 0–30% EtOAc/DCM) with the product eluting at 30% EtOAc. Rf = 0.45 (EtOAc/DCM 3:7). ^1^H NMR (600 MHz, CDCl_3_) δ: 8.35 (d, *J* = 1.6 Hz, 1H), 8.19 (dd, *J* = 8.1, 1.6 Hz, 1H), 7.85–7.80 (m, 2H), 5.08 (s, 2H), 1.60 (s, 9H); ^13^C NMR (151 MHz, CDCl_3_) δ: 162.1, 149.3, 138.7, 134.8, 134.5, 133.1, 133.0, 125.9, 84.2, 53.8, 28.1; HRMS (ESI) *m*/*z*: [M + H]^+^ calcd for C_14_H_17_N_4_O_6_S, 369.0863; found, 369.0860.

### Synthesis and Characterization of BTK Labeling Probes

Synthesis of evobrutinib, **Evo-1**, **Evo-2**, evobrutinib-precursor **22**, and BODIPY-intermediate **25**, were performed as described by Valaka, A. P. et al.^53^

#### 6-((2-(4-(((6-amino-5-(4-phenoxyphenyl)­pyrimidin-4-yl)­amino)­methyl)­piperidin-1-yl)-2-oxoethyl)­sulfonyl)-*N*-(2-(3-(5,5-difluoro-7,9-dimethyl-5*H*-4λ^4^,5λ^4^-dipyrrolo­[1,2-*c*:2′,1′-*f*]­[1,3,2]­diazaborinin-3-yl)­propanamido)­ethyl)­nicotinamide (**Evo-3**)

In a 10 mL round-bottom flask purged with N_2_, **25** (19 mg, 0.051 mmol, 1.2 equiv) was dissolved in dry DCM (1 mL) and basified by addition of DIPEA (9 μL, 0.051 mmol, 1.2 equiv). The red solution was stirred at room temperature for 10 min. A 25 mL round-bottom flask was charged with **24** (26 mg, 0.043 mmol, 1.0 equiv) and HATU (28 mg, 0.072 mmol, 1.7 equiv), and the flask was purged with N_2_. Then, dry DCM (2 mL), DIPEA (13 μL, 0.072 mmol, 1.7 equiv), and the amine solution in the other flask, were all added in rapid succession. The red solution was stirred at room temperature until LC–MS indicated complete reaction. After 2 h, the reaction mixture was concentrated under reduced pressure, and the crude was purified by preparative-HPLC (C18 column, 5–95% MeCN/water with 0.1% TFA) to afford **Evo-3** as a red solid (19 mg, 49%). Rf = 0.31 (MeOH/DCM 1:9). ^1^H NMR (600 MHz, (CD_3_)_2_CO) δ: 9.10 (d, *J* = 1.9 Hz, 1H), 8.43 (dd, *J* = 8.2, 2.0 Hz, 1H), 8.37 (t, *J* = 4.8 Hz, 1H), 8.34 (s, 1H), 8.07 (d, *J* = 8.0 Hz, 1H), 7.59 (br s, 1H), 7.46 (s, 1H), 7.44–7.39 (m, 4H), 7.19 (tt, *J* = 7.4, 1.1 Hz, 1H), 7.16 (d, *J* = 8.6 Hz, 2H), 7.08 (dd, *J* = 8.7, 1.1 Hz, 2H), 6.93 (d, *J* = 4.0 Hz, 1H), 6.66 (t, *J* = 6.2 Hz, 1H), 6.33 (d, *J* = 4.0 Hz, 1H), 6.24 (s, 1H), 4.75 (d, *J* = 14.9 Hz, 1H), 4.68 (d, *J* = 15.5 Hz, 1H), 4.33 (d, *J* = 13.2 Hz, 1H), 4.12 (d, *J* = 14.0 Hz, 1H), 3.57–3.47 (m, 4H), 3.46–3.40 (m, 2H), 3.24 (t, *J* = 7.5 Hz, 2H), 3.16–3.08 (m, 1H), 2.64 (t, *J* = 7.5 Hz, 2H), 2.57–2.52 (m, 1H), 2.51 (s, 3H), 2.27 (s, 3H), 1.98–1.90 (m, 1H), 1.79 (d, *J* = 12.7 Hz, 1H), 1.69 (d, *J* = 11.9 Hz, 1H), 1.35–1.25 (m, 1H), 1.02 (qd, *J* = 12.3, 4.2 Hz, 1H); ^13^C NMR (151 MHz, (CD_3_)_2_CO) δ: 173.4, 173.3, 164.8, 164.7, 161.5, 161.5, 160.5, 160.2, 159.1, 158.9, 157.5, 154.1, 149.7, 149.4, 145.1, 138.0, 135.9, 134.4, 134.3, 133.5, 130.9, 129.5, 125.7, 124.8, 124.6, 122.4, 121.1, 120.0, 117.5, 96.4, 55.6, 47.3, 47.1, 42.5, 41.6, 39.6, 37.1, 35.3, 30.9, 25.1, 14.9, 11.3; ^19^F NMR (564 MHz, (CD_3_)_2_CO) δ: −145.04 (dd, *J* = 65.8, 32.7 Hz); HRMS (ESI) *m*/*z*: [M + H]^+^ calcd for C_46_H_50_BF_2_N_10_O_6_S, 919.3691; found, 919.3677.

#### 4-((2-(4-(((6-amino-5-(4-phenoxyphenyl)­pyrimidin-4-yl)­amino)­methyl)­piperidin-1-yl)-2-oxoethyl)­sulfinyl)-*N*-(2-(3-(5,5-difluoro-7,9-dimethyl-5*H*-4λ^4^,5λ^4^-dipyrrolo­[1,2-*c*:2′,1′-*f*]­[1,3,2]­diazaborinin-3-yl)­propanamido)­ethyl)-3-nitrobenzamide (**Evo-4**)

In a 5 mL microwave vial, **29** (45 mg, 0.048 mmol, 1.0 equiv) was dissolved in CHCl_3_ (1 mL) and cooled to 0 °C. Then a solution of mCPBA (32 mg, 0.14 mmol, 3.0 equiv) in CHCl_3_ (1 mL) was added dropwise. The dark-red solution was stirred at 0 °C for 30 min and then stirred at room temperature until LC–MS indicated full consumption of the starting material and formation of a mixture of mono- and dioxidation. After 20 h, the reaction mixture was concentrated under reduced pressure. The crude was purified by column chromatography (high-capacity silica 5 g, 0–20% MeOH/DCM) to elute the compound at 8% MeOH. Further purification by preparative TLC (MeOH/DCM 1:9) was performed to afford **Evo-4** as an orange solid (9.0 mg, 20%). Rf = 0.32 (MeOH/DCM 1:9). ^1^H NMR (600 MHz, CDCl_3_) δ: 8.76 (dd, *J* = 2.8, 1.5 Hz, 1H), 8.33–8.25 (m, 2H), 8.15 (d, *J* = 1.7 Hz, 1H), 8.12–8.06 (m, 1H), 7.42–7.37 (m, 2H), 7.24 (dd, *J* = 8.6, 1.9 Hz, 2H), 7.20–7.16 (m, 1H), 7.14–7.07 (m, 4H), 7.02 (s, 1H), 6.77 (dd, *J* = 4.0, 1.3 Hz, 1H), 6.57–6.50 (m, 1H), 6.19 (dd, *J* = 4.0, 1.2 Hz, 1H), 6.11 (s, 1H), 4.56–4.39 (m, 4H), 4.28–4.17 (m, 1H), 3.88–3.73 (m, 2H), 3.55–3.43 (m, 4H), 3.35–3.19 (m, 4H), 3.10–2.99 (m, 1H), 2.70 (td, *J* = 7.3, 2.5 Hz, 2H), 2.64–2.52 (m, 1H), 2.51 (s, 3H), 2.22 (s, 3H), 1.89–1.80 (m, 1H), 1.78–1.64 (m, 2H), 1.18–1.00 (m, 2H); ^13^C NMR (151 MHz, CDCl_3_) δ: 174.6, 164.2, 162.3, 162.1, 161.1, 160.2, 159.6, 158.3, 156.9, 156.3, 156.1, 145.6, 145.2, 145.1, 145.0, 144.6, 138.3, 135.4, 133.3, 133.3, 131.9, 130.2, 128.1, 127.8, 127.7, 126.5, 124.4, 124.4, 124.0, 120.9, 120.0, 119.9, 117.1, 97.6, 60.1, 59.0, 46.7, 46.3, 46.2, 42.9, 42.4, 39.3, 36.2, 36.1, 35.6, 30.8, 30.4, 29.7, 29.4, 24.9, 15.1, 11.5; ^19^F NMR (564 MHz, CDCl_3_) δ: −144.11 (ddd, *J* = 68.1, 32.6, 14.7 Hz); HRMS (ESI) *m*/*z*: [M + H]^+^ calcd for C_47_H_50_BF_2_N_10_O_7_S, 947.3640; found, 947.3628. Notes: (a) The oxidation was carried out with 3 eq. mCPBA to afford a mixture of sulfoxide/sulfone and try to isolate both compounds. However, NMR confirmed that the second oxidation was N-oxidation of the pyrimidine ring of evobrutinib and not formation of the sulfone. The low yield of sulfoxide is attributed to isolation of the N-oxide. (b) The^1^H/^13^C/^19^F NMR shows complex splitting indicating two species in solution. This is likely due to rotamers of the piperidine tertiary amide in proximity to the chiral sulfoxide.

#### 5-((2-(4-(((6-amino-5-(4-phenoxyphenyl)­pyrimidin-4-yl)­amino)­methyl)­piperidin-1-yl)-2-oxoethyl)­sulfinyl)-*N*-(2-(3-(5,5-difluoro-7,9-dimethyl-5*H*-4λ^4^,5λ^4^-dipyrrolo­[1,2-*c*:2′,1′-*f*]­[1,3,2]­diazaborinin-3-yl)­propanamido)­ethyl)­pyrazine-2-carboxamide (**Evo-5**)

In a 2 mL microwave vial, **34** (17 mg, 0.019 mmol, 1.0 equiv) was dissolved in CHCl_3_ (1 mL) and cooled to 0 °C. Then a solution of mCPBA (6.8 mg, 0.031 mmol, 1.6 equiv) in CHCl_3_ (0.1 mL) was added dropwise. The orange solution was stirred at 0 °C for 30 min and then stirred at room temperature until LC–MS indicated full consumption of the starting material and formation of a mixture of mono- and dioxidation. After 3 h, the reaction mixture was concentrated under reduced pressure. The crude was purified by column chromatography (high-capacity silica 5 g, 0–15% MeOH/DCM) to elute the compound at 10% MeOH. Further purification by preparative TLC (MeOH/DCM 3:97) was performed to afford **Evo-5** as a red solid (5.6 mg, 32%). ^1^H NMR (600 MHz, CDCl_3_) δ: 9.31 (dd, *J* = 3.2, 1.4 Hz, 1H), 9.05 (dd, *J* = 11.2, 1.4 Hz, 1H), 8.22 (t, *J* = 5.9 Hz, 1H), 8.15 (s, 1H), 7.40 (dd, *J* = 8.5, 7.4 Hz, 2H), 7.23 (d, *J* = 8.5 Hz, 2H), 7.19 (tt, *J* = 7.4, 1.1 Hz, 1H), 7.12 (d, *J* = 8.7 Hz, 2H), 7.09 (dd, *J* = 8.6, 1.1 Hz, 2H), 7.05 (s, 1H), 6.81 (d, *J* = 4.0 Hz, 1H), 6.38–6.32 (m, 1H), 6.25 (d, *J* = 4.0 Hz, 1H), 6.11 (s, 1H), 5.07 (br s, 2H), 4.62 (dt, *J* = 16.8, 6.2 Hz, 1H), 4.54–4.48 (m, 1H), 4.39–4.27 (m, 1H), 4.05–3.93 (m, 1H), 3.78 (d, *J* = 13.0 Hz, 1H), 3.57–3.52 (m, 2H), 3.47 (q, *J* = 5.7 Hz, 2H), 3.35–3.26 (m, 2H), 3.23 (t, *J* = 7.4 Hz, 2H), 3.04 (t, *J* = 12.9 Hz, 1H), 2.65 (t, *J* = 7.4 Hz, 2H), 2.62–2.55 (m, 1H), 2.52 (s, 3H), 2.24 (s, 3H), 1.88–1.79 (m, 1H), 1.75–1.65 (m, 2H), 1.20–1.01 (m, 2H); ^13^C NMR (151 MHz, CDCl_3_) δ: 172.9, 162.9, 162.6, 162.5, 162.2, 161.9, 160.6, 160.2, 158.7, 158.2, 157.1, 155.9, 154.7, 145.4, 144.3, 143.5, 140.9, 135.3, 133.4, 131.9, 130.2, 128.3, 125.2, 124.6, 124.0, 120.7, 120.1, 119.9, 117.4, 97.2, 58.6, 57.9, 46.6, 46.3, 42.3, 40.3, 39.4, 36.2, 35.8, 30.6, 30.4, 29.4, 24.9, 15.1, 11.5; ^19^F NMR (564 MHz, CDCl_3_) δ: −144.37 (dd, *J* = 66.7, 32.8 Hz); HRMS (ESI) *m*/*z*: [M + H]^+^ calcd for C_45_H_49_BF_2_N_11_O_5_S, 904.3694; found, 904.3685. Notes: (a) The oxidation was carried out with 1.6 eq. mCPBA to afford a mixture of sulfoxide/sulfone and try to isolate both compounds. However, NMR confirmed that the second oxidation was N-oxidation of the pyrimidine ring of evobrutinib and not formation of the sulfone. (b) The^1^H/^13^C NMR shows complex splitting indicating two species in solution. This is likely due to rotamers of the piperidine tertiary amide in proximity to the chiral sulfoxide.

#### 5-((2-(4-(((6-amino-5-(4-phenoxyphenyl)­pyrimidin-4-yl)­amino)­methyl)­piperidin-1-yl)-2-oxoethyl)­sulfinyl)-*N*-(but-3-yn-1-yl)­pyrazine-2-carboxamide (**Evo-6**)

In a 10 mL round-bottom flask, **35** (24 mg, 0.039 mmol, 1.0 equiv) was dissolved in CHCl_3_ (1 mL) and cooled to 0 °C. Then a solution of mCPBA (8.8 mg, 0.039 mmol, 1.0 equiv) in CHCl_3_ (1.5 mL) was added dropwise. The colorless solution was stirred at 0 °C for 30 min and then stirred at room temperature until LC–MS indicated full consumption of the starting material. After 1.5 h, the reaction mixture was concentrated under reduced pressure. The crude was purified by column chromatography (high-capacity silica 10 g, 0–10% MeOH/DCM) to afford **Evo-6** (eluted at 10% MeOH) as a white crystalline solid (10 mg, 40%). ^1^H NMR (600 MHz, CDCl_3_) δ: 9.37 (t, *J* = 1.5 Hz, 1H), 9.10 (dd, *J* = 17.7, 1.4 Hz, 1H), 8.17 (s, 1H), 8.12 (t, *J* = 6.3 Hz, 1H), 7.41 (dd, *J* = 8.5, 7.4 Hz, 2H), 7.23 (dd, *J* = 8.7, 2.0 Hz, 2H), 7.20 (tt, *J* = 7.4, 1.2 Hz, 1H), 7.14 (d, *J* = 7.8 Hz, 2H), 7.10 (dd, *J* = 7.6, 1.1 Hz, 2H), 5.12 (br s, 2H), 4.69 (q, *J* = 6.3 Hz, 1H), 4.57–4.50 (m, 1H), 4.45–4.33 (m, 1H), 4.06–3.94 (m, 1H), 3.81 (d, *J* = 11.5 Hz, 1H), 3.67 (q, *J* = 6.5 Hz, 2H), 3.38–3.27 (m, 2H), 3.12–3.04 (m, 1H), 2.64–2.57 (m, 1H), 2.55 (td, *J* = 6.5, 2.7 Hz, 2H), 2.06 (td, *J* = 2.7, 1.0 Hz, 1H), 1.90–1.81 (m, 1H), 1.79–1.72 (m, 1H), 1.69 (d, *J* = 13.6 Hz, 1H), 1.28–1.03 (m, 2H); ^13^C NMR (151 MHz, CDCl_3_) δ: 162.8, 162.7, 162.3, 162.1, 161.9, 160.3, 158.9, 157.1, 155.8, 153.5, 145.3, 145.2, 143.6, 143.5, 141.0, 140.9, 131.9, 130.2, 124.7, 124.5, 120.2, 119.9, 97.1, 81.1, 70.6, 58.4, 57.5, 46.6, 46.4, 42.3, 38.3, 36.3, 36.2, 30.6, 30.4, 29.6, 29.4, 19.6; HRMS (ESI) *m*/*z*: [M + H]^+^ calcd for C_33_H_35_N_8_O_4_S, 639.2496; found, 639.2487. Note: The^1^H/^13^C NMR shows complex splitting indicating two species in solution. This is likely due to rotamers of the piperidine tertiary amide in proximity to the chiral sulfoxide.

#### (*R*)-5-((2-(3-(4-amino-3-(4-phenoxyphenyl)-1*H*-pyrazolo­[3,4-*d*]­pyrimidin-1-yl)­piperidin-1-yl)-2-oxoethyl)­sulfonyl)-*N*-(but-3-yn-1-yl)­pyrazine-2-carboxamide (**Ibr-1**)

In a 5 mL microwave vial, **39** (33 mg, 0.052 mmol, 1.0 equiv) was dissolved in a mixture of CCl_4_ (0.5 mL), MeCN (1 mL), and H_2_O (0.5 mL). The biphasic mixture was stirred vigorously to ensure proper mixing of the phases. Then NaIO_4_ (33 mg, 0.16 mmol, 3.0 equiv) followed by catalytic RuCl_3_ (1.1 mg, 0.0052 mmol, 10 mol %) were added and the black reaction mixture was stirred at room temperature. A white precipitate crashed out during the reaction. The reaction was monitored by LC–MS to optimize the yield of the sulfone (consume sulfide/sulfoxide and avoid further oxidative cleavage of the alkyne to form a carboxylic acid). After 3 h, the reaction was quenched by addition of aqueous saturated Na_2_S_2_O_3_. The vial was rinsed with DCM and concentrated under reduced pressure. The crude was purified by column chromatography (high-capacity silica 5 g, 0–20% MeOH/DCM) to afford **Ibr-1** (eluted at 8% MeOH) as an off-white crystalline solid (15 mg, 43%). Rf = 0.45 (MeOH/DCM 1:9). ^1^H NMR (600 MHz, CDCl_3_) δ: 9.50 (d, *J* = 1.4 Hz, 1H), 9.44 (d, *J* = 1.4 Hz, 1H), 9.21 (d, *J* = 1.4 Hz, 1H), 9.19 (d, *J* = 1.4 Hz, 1H), 8.38 (s, 1H), 8.32 (s, 1H), 8.11 (q, *J* = 5.9 Hz, 2H), 7.62 (dd, *J* = 16.3, 8.6 Hz, 4H), 7.41–7.35 (m, 4H), 7.19–7.12 (m, 6H), 7.11–7.05 (m, 4H), 5.70 (br s, 4H), 4.96–4.90 (m, 1H), 4.87–4.80 (m, 1H), 4.75–4.54 (m, 5H), 4.20–4.15 (m, 1H), 4.08 (dd, *J* = 13.6, 4.0 Hz, 1H), 3.98–3.91 (m, 2H), 3.70–3.63 (m, 4H), 3.42–3.30 (m, 2H), 3.05–2.98 (m, 1H), 2.57–2.50 (m, 4H), 2.49–2.41 (m, 1H), 2.34 (qd, *J* = 12.7, 4.3 Hz, 1H), 2.29–2.21 (m, 2H), 2.11–2.04 (m, 3H), 1.94–1.86 (m, 3H), 1.69–1.61 (m, 1H); ^13^C NMR (151 MHz, CDCl_3_) δ: 161.6, 161.6, 160.3, 159.9, 158.8, 158.7, 158.1, 158.0, 156.4, 156.4, 156.1, 155.9, 155.0, 154.6, 154.5, 154.3, 146.8, 146.7, 144.3, 144.2, 144.1, 144.0, 141.8, 141.6, 130.1, 130.1, 127.7, 127.6, 124.3, 124.2, 119.8, 119.7, 119.2, 98.8, 98.6, 81.0, 70.7, 55.9, 55.6, 53.2, 52.3, 50.6, 47.1, 46.2, 42.6, 38.4, 30.0, 29.5, 24.9, 23.4, 19.5; HRMS (ESI) *m*/*z*: [M + H]^+^ calcd for C_33_H_32_N_9_O_5_S, 666.2242; found, 666.2230. Note: The^1^H/^13^C NMR shows two sets of peaks indicating two species in solution. This is likely due to cis–trans rotamers of the piperidine tertiary amide in proximity to the stereocenter. The two species were present at roughly a 1:1 ratio. All signals for the pair are listed above.

#### 5-((2-(4-amino-3-(4-phenoxyphenyl)-1*H*-pyrazolo­[3,4-*d*]­pyrimidin-1-yl)­ethyl)­sulfonyl)-*N*-(but-3-yn-1-yl)­pyrazine-2-carboxamide (**Ibr-2**)

In a 5 mL microwave vial, **44** (51 mg, 0.095 mmol, 1.0 equiv) was dissolved in a mixture of CCl_4_ (0.75 mL), MeCN (1.5 mL), and H_2_O (0.75 mL). The biphasic mixture was stirred vigorously to ensure proper mixing of the phases. Then NaIO_4_ (61 mg, 0.29 mmol, 3.0 equiv) followed by catalytic RuCl_3_ (2.0 mg, 0.0095 mmol, 10 mol %) were added and the gray reaction mixture was stirred at room temperature. A white precipitate crashed out during the reaction. The reaction was monitored by LC–MS to optimize the yield of the sulfone (consume sulfide/sulfoxide and avoid further oxidative cleavage of the alkyne to form a carboxylic acid). After 2 h, the reaction was quenched by addition of aqueous saturated Na_2_S_2_O_3_. The vial was rinsed with DCM and concentrated under reduced pressure. The crude was purified by column chromatography (high-capacity silica 10 g, 50–100% EtOAc/DCM, then 0–20% MeOH/EtOAc) to afford **Ibr-2** (eluted at 65% EtOAc/DCM) as an off-white solid (19 mg, 35%).

^1^H NMR (600 MHz, CD_3_)_2_SO) δ: 9.06 (t, *J* = 6.1 Hz, 1H), 8.90 (d, *J* = 1.4 Hz, 1H), 8.87 (d, *J* = 1.4 Hz, 1H), 8.19 (s, 1H), 7.50–7.43 (m, 4H), 7.21 (tt, *J* = 7.4, 1.1 Hz, 1H), 7.15 (d, *J* = 7.7 Hz, 2H), 7.10 (d, *J* = 8.6 Hz, 2H), 4.78–4.74 (m, 2H), 4.34–4.29 (m, 2H), 3.40 (td, *J* = 7.4, 6.0 Hz, 2H), 2.83 (t, *J* = 2.6 Hz, 1H), 2.43 (td, *J* = 7.4, 2.7 Hz, 2H); ^13^C NMR (151 MHz, CD_3_)_2_SO) δ: 161.6, 158.2, 157.8, 156.6, 156.3, 154.5, 153.2, 147.4, 144.0, 143.5, 141.0, 130.6, 130.3, 127.5, 124.4, 119.7, 119.2, 97.3, 82.3, 72.8, 51.5, 41.2, 38.6, 18.9; HRMS (ESI) *m*/*z*: [M + H]^+^ calcd for C_28_H_25_N_8_O_4_S, 569.1714; found, 569.1711.

### Warhead Reactivity and Stability Assays

#### Reactivity Assay for Warhead Fragments

100 μM of the electrophile was incubated with 100 μM 2-methyl-3-nitrobenzoic acid as internal standard and 5 mM *N*-acetyl cysteine in 100 mM PBS buffer pH 7.4. For electrophiles **4j**, **6j**, **5k**-**l**, **7k**-**l**, benzophenone was used as internal standard. Reaction mixtures were kept at 23 °C. After various points 15 μL of the reaction mixture was injected into the HPLC. The reaction was monitored by measuring the peak area of the electrophile, normalized to the area of the internal standard. The natural logarithm of the remaining electrophile over time were fitted to linear regression, and *t*_1/2_ was calculated as *t*_1/2_ = ln (2)/–slope. All measurements were conducted twice.

Similar reactivity profiling was performed using 5 mM of *N*_α_-acetyl lysine or 5 mM *N*-Boc serine as cellular nucleophile. The experiment was conducted as described above, except that borate buffer (pH 8.5) was used and the reaction mixtures were incubated at 37 °C.

For the GSH reactivity assay with **4b**, **6e**, **4l**, **6l**, and **7l**, the assay was performed similar to the NAC assay where 5 mM GSH was used instead of NAC as nucleophile. For the benchmark experiment, afatinib or ibrutinib (100 μM) were incubated with NAC (5 mM) in 50 mM PBS buffer (pH 8.0, containing 5% DMSO) and the reaction was monitored by HPLC/MS.

#### Buffer Stability Assay for Warhead Fragments

100 μM of the electrophile was incubated with 100 μM 2-methyl-3-nitrobenzoic acid as internal standard in 100 mM PBS buffer pH 7.4. For electrophiles **4j**, **6j**, **5k**–**l**, **7k**–**l**, **6m** benzophenone was used as internal standard. Reaction mixtures were kept at 23 °C. Every 24 h, 15 μL of the reaction mixture was injected into the HPLC. For electrophiles that decomposed rapidly within 24 h, buffer stability was monitored at various time points over 24 h. The reaction was monitored by measuring the peak area of the electrophile, normalized to the area of the internal standard. Survival rates were fitted to a single-phase exponential decay model to determine *t*_1/2_ of the electrophile in PBS buffer. All measurements were conducted twice.

### Molecular Modeling and MD Simulations

Molecular docking and MD simulations were performed using Schrödinger and the detailed procedures can be found in the Supporting Information.

### Biochemical Assays

#### In-Gel Fluorescence Labeling of Recombinant BTK

For covalent binding tests of probes to BTK, 100 ng (1 μL of 100 ng/μL stock) of full length-recombinant BTK (MRC PPU Reagents, University of Dundee, UK, #DU12110) was mixed with 1 μM probe (1 μL of 20 μM working solution in DMSO) and 18 μL PBS and incubated for 1 h at room temperature. Samples were mixed with 7.7 μL of 4X NuPAGE LDS sample buffer (Invitrogen #NP0007) and 3 μL of 10X NuPAGE sample reducing agent (Invitrogen #NP0009), and denatured at 70 °C for 10 min. Proteins were separated by electrophoresis on 4–12% NuPAGE Bis-Tris gels (Invitrogen #NP0321BOX) in 1X NuPAGE MOPS SDS running buffer (Invitrogen #NP000102) at 150 V for 75 min. Twenty μL sample was loaded on the gel along with 5 μL of Precision Plus Protein Dual Color Standards (Bio-Rad #1610374) diluted 1:10. A ChemiDoc Imaging system (Bio-Rad, blue LED, 530/28 filter) was used for fluorescence detection.

#### ADP-Glo Kinase Assay—Determination of IC_50_ Values

The ADP-Glo Kinase Assay (Promega) was performed using the supplied protocol with full-length recombinant BTK (MRC PPU Reagents, University of Dundee, UK, #DU12110). All procedures were at room temperature, with dilutions in Tris buffer (40 mM Tris–HCl, 20 mM MgCl_2_, 2 mM MnCl_2_, 0.1 mg/mL BSA, 50 μM DTT, pH 7.5). The kinase reaction quadruplicates, (5 μL reaction volume) were carried out in a 384-well White Polystyrene Microplate (Corning model 3824) with 10 ng BTK, 50 μM ATP, 0.2 μg/μL Poly­(Glu_4_Tyr_1_), and test compounds (0–100 μM). Final DMSO concentration was 1%. After preincubation (1 μL compound and 2 μL BTK solution for 30 min), 2 μL substrate solution was added. The reaction was incubated for 60 min, followed by 5 μL ADP-Glo Reagent, and another 40 min incubation. Kinase Detection Reagent (10 μL) was added, and the solution was incubated for 60 min. Luminescence was measured on a SpectraMax iD5Microplate Reader (Molecular Devices) using 1000 ms integration time. Luminescence values were converted to % activity, normalized to the positive control, and plotted using nonlinear regression of the Sigmoidal dose–response curve. The IC_50_ values were determined and shown as mean ± SD.

#### ADP-Glo Kinase Assay—ATP Titration Experiment

To determine ATP-binding interference by **Ibr-2** and ibrutinib, BTK reactions were performed in quadruplicates as described above using the following conditions: 10 ng BTK, 500 nM test compound, 6.25–200 μM ATP, 0.2 μg/μL Poly­(Glu_4_Tyr_1_), 1% DMSO. BTK was preincubated with compound for 30 min, followed by substrate addition. The resulting kinase reaction was incubated for 60 min and then the ADP-Glo Kinase Assay was performed as described above. Relative luminescence units (RLU) given as mean ± SD were plotted versus the ATP concentration for each compound.

### Surface Plasmon Resonance

#### Expression and Purification of Recombinant BTK

Biotinylated recombinant human full-length Bruton’s tyrosine kinase (GSGS-Avi-GSGS-FL-BTK[2–659]) was produced in Sf21 cells using baculovirus infection. Cells were inoculated with virus and grown at 27 °C, 140 rpm, for 48 h before harvested by centrifugation (6500 rpm, 4 °C, 15 min). Cell pellet was lysed by passing through chilled French press followed by centrifugation (16,000 rpm, 4 °C, 4 h). The supernatant was collected. Protein was purified by affinity (Ni excel resin, ÄKTA, Cytiva) and size-exclusion chromatography (Superdex200, ÄKTA, Cytiva), before biotinylation using BirA. The protein was finally concentrated to 40 μM in 20 mM Tris pH 8.0, 150 mM NaCl, 5% glycerol and 2 mM TCEP, snap frozen in liquid N_2_, and stored at −80 °C.

#### Surface Plasmon Resonance Binding Assay

Compound affinities (*K*_D_) to recombinant BTK were determined in a direct binding assay using an 8K surface plasmon resonance (SPR) biosensor (Cytiva) at 20 °C. Briefly, biotinylated BTK was mixed in a 2:1 molar ratio with SwitchAvidin^67^ and immobilized as a complex on a BD200 M sensor chip (Xantec). This strategy enables a regenerable surface immobilization, compatible with irreversible covalent compound binding.^68^ The surface was washed with 10 mM NaOH, 1 M NaCl followed by immobilization of protein. Immobilization levels were typically 5000 RU. The reference spot was treated as described, omitting the protein-complex injection. Compound concentration series were injected (60 s) over the immobilized protein in increasing concentrations (up to 3 μM) using single cycle kinetics (SCK) in running buffer (10 mM HEPES pH = 7.4, 150 mM NaCl, 0.05% Tween-20). After 30 min washing, the binding capacity of the surface was probed with a single injection (60 s) of 5 μM **Ibr-NH**. Each cycle ended with regeneration of the surface using 0.25% SDS, 2.5% citric acid. A Langmuir 1:1 interaction model was fitted to the experimental traces of ibrutinib and **Ibr-NH** for determination of *K*_D_, while a heterogeneous fit model was used for **Ibr-1** and **Ibr-2**.

### Mass Spectrometry on Recombinant BTK

#### Intact Protein Mass Spectrometry Analysis

Intact protein mass of recombinant BTK was determined using a Bruker Maxis-II ETD II-QTOF MS instrument. BTK (0.92 mg/mL, 11.57 μM, Dundee University, #DU12110) was diluted 1:10 in formic acid (final concentration 0.1 mg/mL) before separation. UPLC separation was performed on a C4 column (300 Å, 1.7 μm, 2.1 mm × 100 mm), column temperature 60 °C, flow 0.3 mL/min. Mobile phase A: 0.1% formic acid/H_2_O, B: 0.1% formic acid/MeCN. Gradient: 20% B for 5 min, increasing linearly to 100% B for 3 min, holding at 100% B for 4 min, back to 20% B in 0.1 min. For data evaluation, data have been processed with Bruker software DataAnalysis6.2.

#### Time-Course Labeling Experiment of BTK with Ibr-2

Recombinant BTK (0.92 mg/mL, 11.57 μM, Dundee University, #DU12110) was diluted to 200 nM in 50 mM ABC buffer pH 8.0 followed by addition of **Ibr-2** at the specific final concentration (100, 200, 400, 600 nM) from a 100X working solution in 50 mM ABC buffer pH 8. Reaction volume was 680 μL. The reaction mixtures were incubated at 23 °C (550 rpm) and at certain time points, 170 μL were removed and denatured at 95 °C (5 min, 750 rpm). Aliquots were kept at −80 °C until tryptic digestion.

#### Tryptic Digest and Peptide Analysis

The aliquots of various time points (170 μL) were dried in a speedvac. The protein was dissolved in 6 M urea, 80 mM ABC buffer, and 30 mM DTT, and was reduced for 10 min at 40 °C with shaking. Addition of 1.1 equiv. iodoacetamide followed by further reaction for 5 min at 40 °C was performed to modify the cysteine residues into their carbamidomethyl derivatives. By addition of H_2_O, the final urea concentration was lowered to ∼ 3 M in 50 mM ABC buffer. The digest was started with 1.5 μg (1 μg/μL H_2_O) Trypsin (ThermoScientific, Pierce MS grade). Digestion was run for 2 h at 30 °C and left at room temperature overnight. The total volume of the digest was 55 μL. The further analysis and separation of peptides have been performed by reversed phase HPLC-MS on an Agilent 1290 HPLC system connected to a Bruker Maxis ETD II QTof MS instrument. For LC–MS analysis, 10 μL of digest were diluted 1:4 with 0.1% formic acid; typically 15 μL has been injected on a Waters Aquity Premier CSH C18, 2.1 × 150 mm, 1.7 μm column. Chromatography have been performed at 0.3 mL/min, 40 °C, mobile phase A: 0.2% formic acid/H_2_O, B: 0.2% formic acid/MeCN. Gradient: 0–35%B within 36 min. For data evaluation, data have been processed with Bruker software DataAnalysis6.2 and BioTools3.2. Quantification was performed by measuring the relative area of the peak corresponding to the tryptic peptide containing Cys481 (Q_467_RPIFIITEYMANG**C**LLNYLR_487_).

### Cellular Assays

#### Cell Lines

Ramos B-cells were purchased from the American Type Culture Collection (ATCC). Cells were maintained in Iscove’s Modified Dulbecco’s Medium with 25 mM HEPES (1X IMDM GlutaMAX, Gibco #31980022) supplemented with 10% heat-inactivated fetal bovine serum (FBS, Gibco #A5670801), penicillin (100 U/mL) and streptomycin (100 μg/mL) (HyClone, Cytiva #SV30010), and 50 μM β-mercaptoethanol (Gibco #31350010). Cells were grown at 37 °C in a humidified 5% CO_2_ atmosphere, and tested negative for mycoplasma contamination (Mycostrip, Invivogen).

### Labeling of Ramos Cells by Probes—Gel Scanning and Western Blotting

#### Incubation of Cells

Ramos cells (7 × 10^6^ cells) were treated at 37 °C in a 60 cm^2^ dish (10 mL cell suspension; 7 × 10^5^ cells/mL) according to the experiment:

- Concentrations of **Evo-5** : treatment with DMSO, 4 μM **Evo-2**, or 0.1–4 μM **Evo-5** for 2 h.
- Concentrations of alkyne probes: treatment with DMSO, or 10–500 nM of **Evo-6**, **Ibr-1**, or **Ibr-2** for 1 h.
- Incubationtimes of**Ibr-2** : treatment with DMSO or 100 nM **Ibr-2** for 10, 30, or 60 min.
- Competition experiment of alkyne probes: pretreatment of cells with DMSO or 1 μM ibrutinib for 30 min followed by treatment with DMSO, or 100 nM of **Evo-6**, **Ibr-1**, or **Ibr-2** for 1 h. The corresponding Western blot used treatment with DMSO or 500 nM of probes.
- BTK activity measurements: pretreatment of cells with DMSO or 1 μM ibrutinib for 30 min followed by treatment with DMSO or 100 nM **Ibr-2** for 1 h. Cells were washed twice with ice-cold PBS, resuspended in 2 mL fresh medium in a 6-well plate, and incubated with or without 10 μg/mL goat antihuman IgM (Jackson ImmunoResearch #109-006-129) for 10 min at 37 °C to stimulate BCR signaling.

#### Preparation of Cell Lysates

Cells were harvested, washed twice with ice-cold PBS, and lysed at 4 °C for 30 min in 1X RIPA buffer (Cell Signaling #9806) supplemented with 1X protease and phosphatase inhibitors (Halt Protease and Phosphatase Inhibitor Cocktail, Thermo Fisher Scientific #87785). Lysates were sonicated (3 × 30 s), centrifuged (14,000*g*, 4 °C, 10 min), and the supernatant collected. The protein concentration was determined using the BCA protein assay (Pierce BCA Protein Assay Kit, Thermo Fisher Scientific #23227) and adjusted to the same concentration with PBS. BODIPY-treated samples were denatured directly, while alkyne-treated samples first underwent modification with TAMRA-N_3_.

#### Modification of Lysates with TAMRA-N_3_ through Click Chemistry

CuSO_4_ (50 mM in water) and sodium ascorbate (100 mM in water) stocks were prepared fresh for each experiment. THPTA (50 mM in water) and TAMRA-N_3_ (10 mM in DMSO, M) stocks were prepared and stored at −20 °C. In 1.5 mL Eppendorf tubes, alkyne-treated cell lysate was diluted in PBS buffer and a premixed click solution of TAMRA-N_3_, CuSO_4_, and THPTA was added. The reaction was initiated by addition of sodium ascorbate. Final concentrations were 1 mg/mL cell lysate, 40 μM TAMRA-N_3_, 3 mM CuSO_4_, 3 mM THPTA, and 3.7 mM sodium ascorbate (100 μL reaction volume). The reaction mixture was incubated at room temperature for 1 h under shaking (700 rpm). Afterward, 400 μL of cold acetone (−20 °C) was added. The tube was vortexed, and the mixture was left at −20 °C for 1 h to precipitate proteins. The precipitate was centrifuged (14,000*g*, 4 °C, 10 min), the supernatant was removed, and the proteins were resuspended in 100 μL of PBS buffer using sonication. Samples were then denatured.

#### Denaturation and Gel Electrophoresis

100 μL cell lysate (BODIPY-treated or TAMRA-modified) was mixed with 38.5 μL of 4X NuPAGE LDS sample buffer (Invitrogen #NP0007) and 15.4 μL of 10X NuPAGE sample reducing agent (Invitrogen #NP0009), and denatured at 70 °C for 10 min. Proteins were separated by electrophoresis on 4–12% NuPAGE Bis-Tris gels (Invitrogen #NP0321BOX) in 1X NuPAGE MOPS SDS running buffer (Invitrogen #NP000102) at 150 V for 75 min. Twenty μL sample (11.8–22.7 μg total protein) was loaded on the gel along with 5 μL molecular weight markers diluted 1:10. Precision Plus Protein Dual Color Standards (Bio-Rad #1610374) was used for BODIPY fluorophores and Precision Plus Protein All Blue Prestained Protein Standards (Bio-Rad #1610373) for TAMRA fluorophores.

#### In-Gel Fluorescence Detection and Protein Staining

A ChemiDoc imaging system (Bio-Rad) was used for fluorescence detection. Gels with BODIPY-samples were scanned using blue LED (530/28 filter) to detect fluorescence from both BODIPY and molecular weight markers in a single-channel. Gels with TAMRA-samples were scanned using green LED (605/50 filter) for TAMRA and red LED (695/50 filter) for molecular weight markers, to create a merged picture of the two channels. After fluorescence scanning, gels were stained for total protein content using SimplyBlue SafeStain (Invitrogen #LC6065) according to the supplied manual.

#### Western Blotting

After electrophoresis, proteins were transferred to 0.2 μm nitrocellulose membranes (GenScript) using wet transfer with XCell II Blot Module (Invitrogen). The transfer used 1X Tris-Glycine transfer buffer with 20% methanol and ran at 15 V, overnight at 4 °C. 0.1% Tween-20 in Tris-buffered saline (TBS-T) was used for membrane washes and preparation of blocking buffers. Membranes were blocked with 5% BSA in TBS-T (for BTK detection) and 5% milk in TBS-T (for p-BTK and β-actin detection) for 1 h at room temperature.

Total BTK was probed using a primary mouse anti-BTK monoclonal antibody (Cell Signaling #56044), diluted 1:1000 in 5% BSA in TBS-T, at 4 °C overnight. This was followed by a secondary HRP-conjugated sheep antimouse antibody (Cytiva #NA931 V), diluted 1:5000 in milk in TBS-T, at room temperature for 30 min, for chemiluminescence detection, or a secondary Alexa633-conjugated goat antimouse antibody (Invitrogen #A21050), diluted 1:5000 in 5% BSA in TBS-T, at room temperature for 1 h, for fluorescence detection.

Phosphorylated BTK was probed using a primary rabbit anti-p-BTK (Tyr223) monoclonal antibody (Cell Signaling #87141), diluted 1:1000 in 5% milk in TBS-T, at 4 °C overnight. This was followed by a secondary HRP-conjugated donkey antirabbit antibody (Cytiva #NA934 V), diluted 1:5000 in milk in TBS-T, at room temperature for 30 min β-actin was probed using a primary HRP-conjugated mouse anti-β-actin monoclonal antibody (Invitrogen, #MA5-15739-HRP), diluted 1:1000 in 5% milk in TBS-T, at room temperature for 1 h.

Membranes were washed with TBS-T for 3 × 5 min after unconjugated antibodies and for 3 × 10 min after conjugated antibodies. Fluorescence from Alexa633 was detected with a ChemiDoc imaging system (Bio-Rad) using red LED (695/50 filter). Chemiluminescence signals from HRP were developed with SuperSignal West Pico PLUS Chemiluminescent Substrate (Thermo Fisher Scientific #34580) according to the supplied manual, and images were acquired with a ChemiDoc imaging system. Membranes were stripped between detections using Restore PLUS Western Blot Stripping Buffer (Thermo Fisher Scientific #46430) at 37 °C for 15 min according to the supplied manual.

### Pull-Down Proteomics

#### Preparation of Cell Lysates

For proteomics experiments, the IMDM medium contained the same supplements as specified above except no fetal-bovine serum was added to simplify downstream analysis. Ramos cells (6 × 10^7^ cells) were treated at 37 °C in dishes (20 mL cell suspension; 3 × 10^6^ cells/mL) with DMSO or 250 nM **Ibr-2** for 1 h. The experiment was conducted in three biological replicates using different batches of cells. Cell lysis was performed as described above.

#### Modification of Lysates with Biotin-N_3_ and Pull-Down

For proteome labeling using the SP2E workflow, a modified protocol by Becker et al.^64^ was followed. A total of 800 μg of protein (2 mg/mL) of **Ibr-2**-treated or DMSO-treated lysates was transferred into a 2 mL Eppendorf tube. Both conditions were performed in triplicates. Each replicate was clicked with biotin-N_3_ (100 μM final concentration, 3.6 mM CuSO_4_, 3.6 mM THPTA, and 4.5 mM sodium ascorbate) in a shaker (1 h, 800 rpm, room temperature) in a total reaction volume of 400 μL. Afterward, the click reaction was stopped by addition of 400 μL 8 M urea. A total of 100 μL of mixed hydrophobic and hydrophilic carboxylate-coated magnetic beads (1:1, prewashed with PBS, Cytiva) was added to the click reaction mixture, followed by 600 μL of absolute EtOH to precipitate the proteins. After resuspending the beads via vortexing, the suspension was incubated for 5 min at 950 rpm, room temperature. The beads were washed thrice with 500 μL 80% ethanol in water using a magnetic rack and the proteins were separately eluted by the addition of 0.5 mL 0.2% SDS in PBS. For this, the beads were resuspended, incubated for 5 min at 950 rpm, room temperature, and the supernatant was directly transferred onto 50 μL of equilibrated streptavidin-coated magnetic beads (Dynabeads MyOne T1, Invitrogen, three times prewashed with 500 μL of 0.2% SDS in PBS). The procedure was repeated once more and the supernatants were combined and incubated for 1 h, 950 rpm, room temperature, for biotin/streptavidin binding. The streptavidin-coated magnetic bead mixture was washed thrice with 300 μL PBS pH 7.4 and 300 μL MQ water. Washed beads were resuspended in 80 μL 50 mM HEPES buffer.

#### Proteomic Sample Preparation

Relative quantification was performed to compare protein expression in control and probe samples. The beads with attached proteins were washed twice with 1 mL 50 mM HEPES, dissolved in 50 μL 50 mM HEPES, reduced (5 mM dithiothreitol (DTT), 30 min, 37 °C) and alkylated (10 mM iodoacetamide (IAA), 30 min, room temperature). Samples were digested by addition of 0.4 μg LysC/Trypsin (Promega, 37 °C) for 3 h. Supernatant was removed, and beads were washed with 40 μL 50 mM HEPES and combined. Peptide samples were digested overnight after extra addition of 0.4 μg LysC/Trypsin and labeled using TMTpro 18-plex isobaric mass tagging reagents (Thermo Fisher Scientific). The labeled samples were pooled into one TMT-set and purified using HiPPR Detergent Removal Resin and Pierce peptide desalting spin columns (both Thermo Scientific), according to the manufactureŕs instructions. The TMT-set was fractionated with High-pH Spin Column into 10 fractions using a gradient from 8% to 50% acetonitrile, 0.1% triethylamine in water (Pierce, Thermo Scientific). The fractions were evaporated in speed vac system and reconstituted in 20 μL 3% acetonitrile, 0.1% trifluoroacetic for LC–MS3 analysis. The detailed procedure about LC–MS and data analysis can be found in the Supporting Information.

### Cell Viability Assay

100 μL of Ramos cell suspension (250,000 cells/ml) was seeded in a Sterile White Flat Bottom 96-well Microplate (Corning #3917) to afford 25,000 cells/well. The cells were treated in quadruplicates with DMSO (vehicle) or 10–1000 nM of **Evo-6**, **Ibr-1**, or **Ibr-2** for 1 h at 37 °C. Next, the CellTiter-Glo 2.0 Cell Viability Assay (Promega #G9242) was performed according to the supplied manual. The plate was equilibrated to room temperature for 30 min followed by addition of 100 μL CellTiter-Glo 2.0 Reagent. The contents were mixed by orbital shaking for 2 min to induce cell lysis. The plate was incubated for 10 min at room temperature and luminescence was measured on a SpectraMax iD5Microplate Reader (Molecular Devices) using 1000 ms integration time. Relative luminescence units (RLUs) were calculated as mean ± SD, and each concentration was compared to the vehicle to assess the cell viability.

## Supplementary Material



































## Data Availability

All unprocessed HPLC chromatograms from the warhead reactivity and stability assays have been deposited to Swedish National Data Service (SND) and are available at: DOI: 10.5878/nmx0-m480.

## Notes

Supporting figures and tables, synthetic schemes, supplementary methods, chemical synthesis of starting materials (**1a-o, 9–12, 17, 19**), aryl thioethers (**2a-2o, 3a-3o, 18, 20**), and intermediate compounds for probe synthesis (**21, 23, 24, 26–35, 37–40, 42–44**), NMR spectra, LC–MS data from the reactivity assays with NAC and GSH, and HPLC traces of probes (PDF)
Docking structures of probes and structural files from MD simulations Evo-1 (PDB)
Evo-2 (PDB)
Evo-4 (PDB)
Evo-5 (PDB)
Evo-6 (PDB)
Evo-6_MD_cluster1 (PDB)
Evo-6_MD_cluster2 (PDB)
Ibr-1 (PDB)
Ibr-1_MD_cluster1 (PDB)
Ibr-1_MD_cluster2 (PDB)
Ibr-2 (PDB)
Ibr-2_MD_cluster1 (PDB)
Proteomics Data Set (XLSX)
Ibr-2_MD_cluster2 (PDB)
Molecular formula strings with associated *t*_1/2_ and biochemical data (CSV)
Ibrutinib (PDB)
