# Supplementary material for: Tunable Aromatic Sulfoxides and Sulfones as Cysteine-Targeting Warheads: Exploring the Structure–Reactivity Relationship
Source: J Med Chem. 2026 Mar 9;69(6):7214–37. doi: 10.1021/acs.jmedchem.5c03536 (PMC13036770; doi:10.1021/acs.jmedchem.5c03536)
Supplement: Supplementary file 1 [file jm5c03536_si_001.pdf]

Supporting Information

Tunable Aromatic Sulfoxides and Sulfones as Cysteine-Targeting Warheads: Exploring the Structure-Reactivity Relationship

Hampus Nyström[a§], Anna P. Valaka[a§], Hanna A. Kalesse[a], Liliana Håversen[b], Thomas Olsson[a], Anders Gunnarsson[c], Fritz Schweikart[d], Jan Borén[b], and Morten Grøtli[a]*

[a] Department of Chemistry and Molecular Biology, University of Gothenburg, 405 30, Gothenburg, Sweden

[b] Department of Molecular and Clinical Medicine, University of Gothenburg and Sahlgrenska University Hospital, 413 45, Gothenburg, Sweden

[c]Discovery Sciences, R&D Gothenburg, AstraZeneca, 431 83, Mölndal, Sweden

[d]Pharmaceutical Development, Mass Spectrometry/Structural Elucidation, R&D Gothenburg, AstraZeneca, 431 83, Mölndal, Sweden

* Email: grotli@chem.gu.se

§ *H.N. and A.P.V. contributed equally to this work*

Table of Contents

1 Supporting Figures and Schemes 2

2 Supplementary Methods 32

2.1 Molecular Modeling 32

2.2 Pull-Down Proteomics Analysis 33

3 Chemical Synthesis of Starting Materials and Intermediates 34

3.1 General Procedures 34

3.2 Synthesis and Characterization of Starting Materials 34

3.3 Synthesis and Characterization of Aryl Thioethers (2a-2o, 3a-3o, 18, 20) 40

3.4 Synthesis and Characterization of Intermediates for BTK Labeling Probes 47

4 References 56

5 NMR Spectra 57

6 LC-MS Traces of Reactivity Assays with NAC and GSH 196

7 HPLC Traces of Probes 241

1 Supporting Figures and Schemes

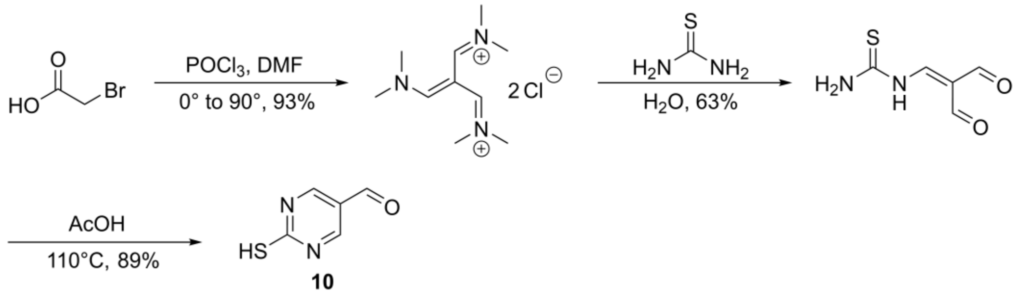

Scheme S1. Synthesis of 2-mercaptopyrimidine-5-carbaldehyde (**10**) from bromoacetic acid.

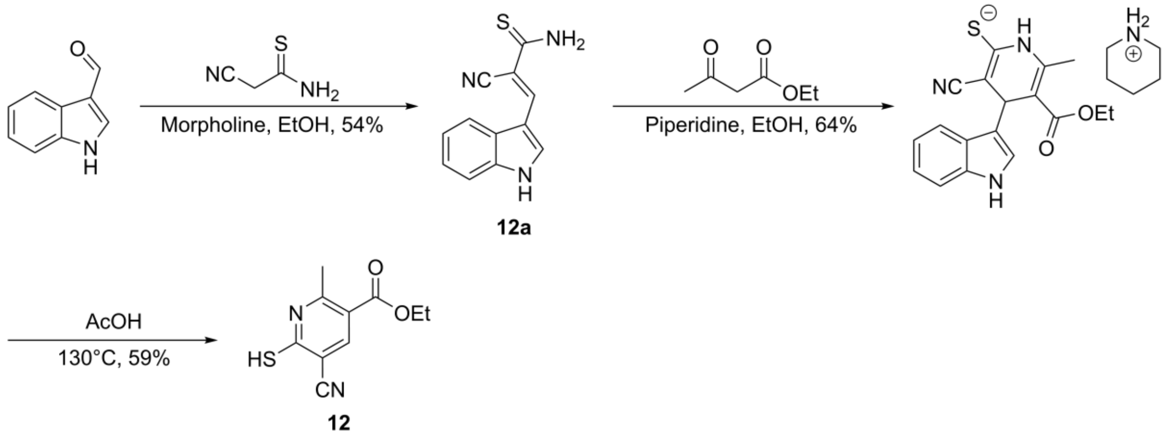

Scheme S2. Synthesis of ethyl 5-cyano-6-mercapto-2-methylnicotinate (**12**) from indole-3-carboxaldehyde and 2-cyanothioacetamide.

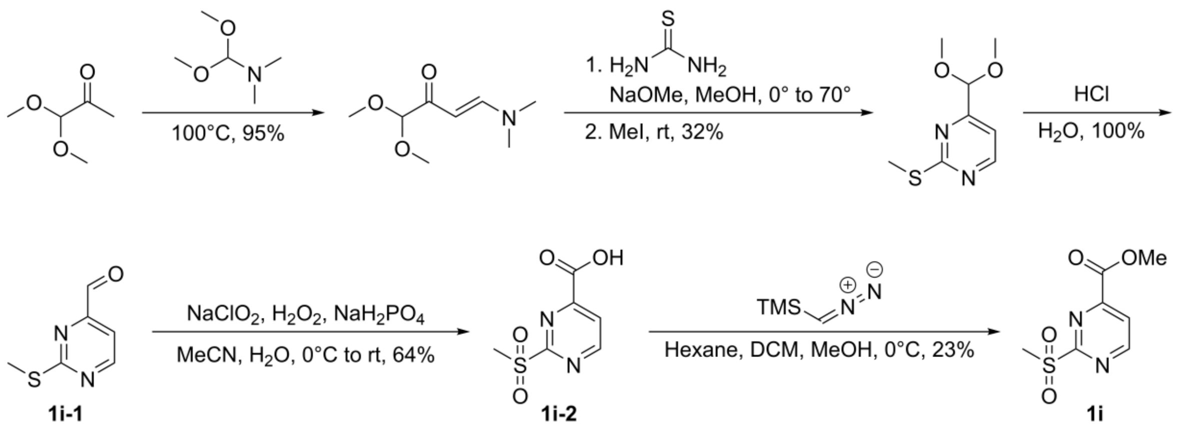

Scheme S3. Synthesis of methyl 2-(methylsulfonyl)pyrimidine-4-carboxylate (**1i**) from 1,1-dimethoxyacetone and *N*,*N*-dimethylformamide dimethyl acetal.

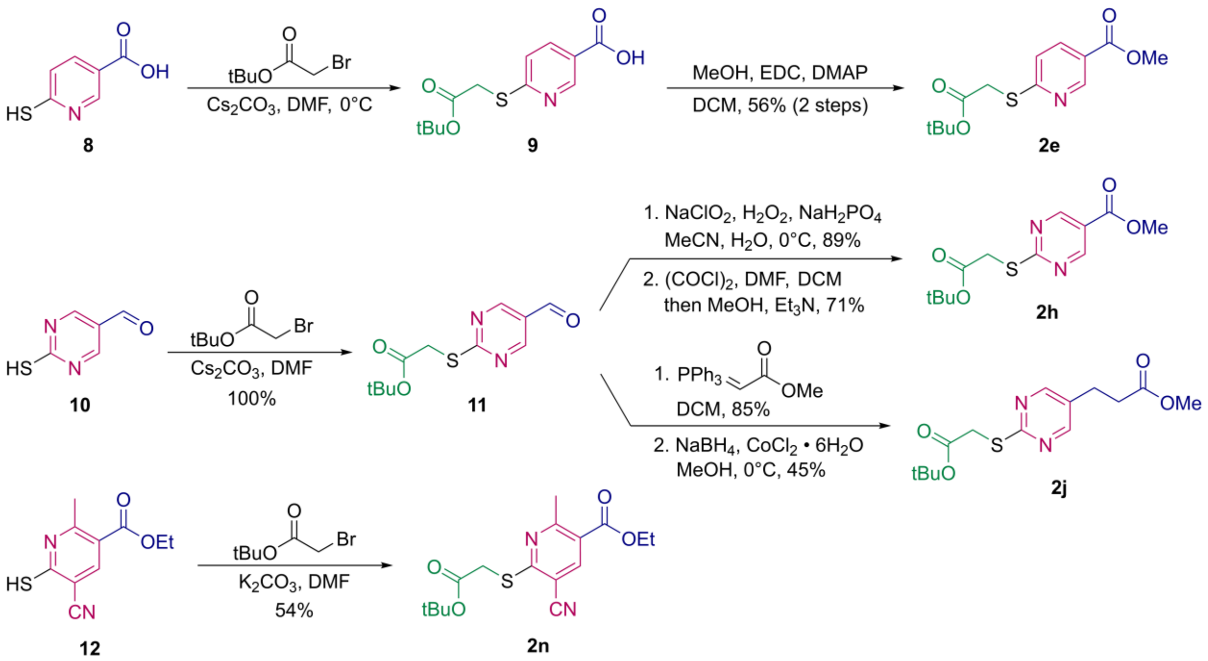

Scheme S4. Synthesis of thioethers via *S*-alkylation of heteroaromatic thiols, followed by further transformations to install the ester linkers highlighted in blue.

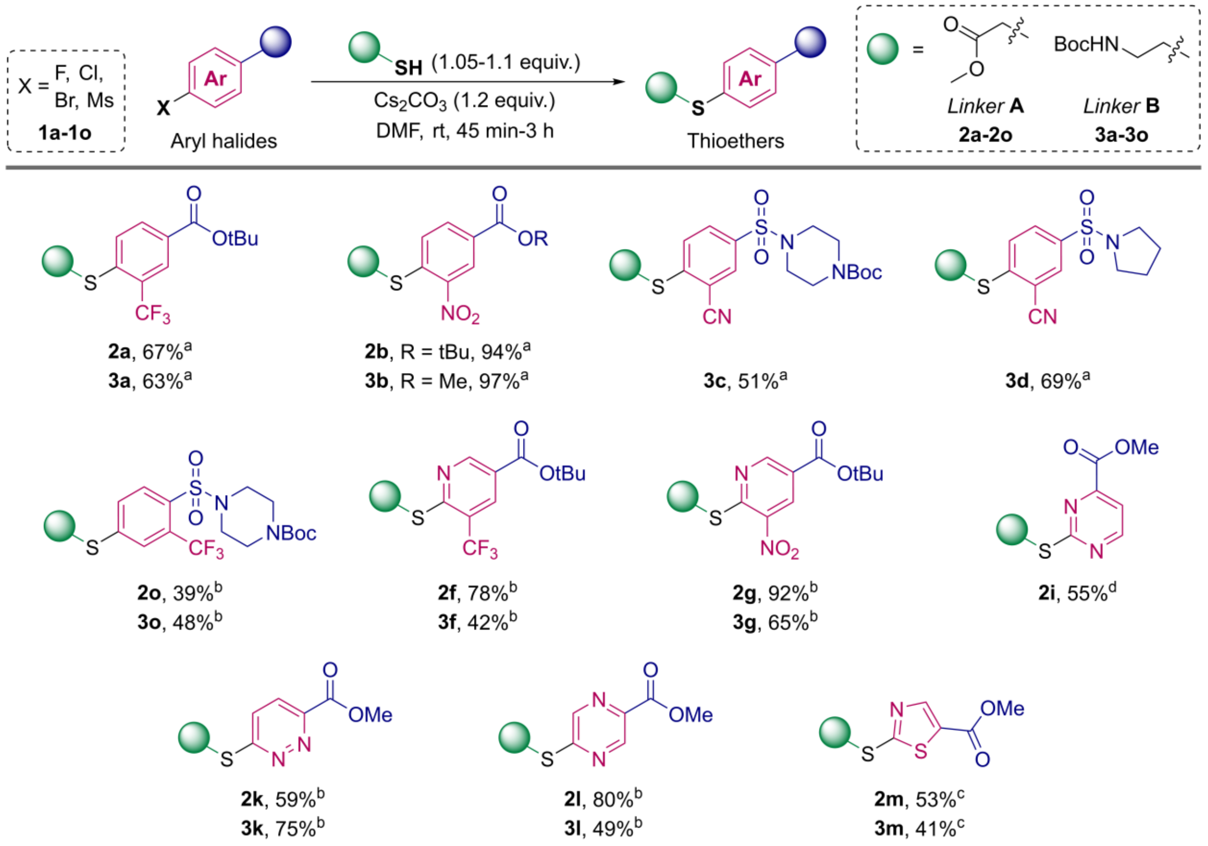

Figure S1. Synthesis of thioethers through nucleophilic aromatic substitution reactions. Aryl halides were displaced by methyl thioglycolate or 2-(Boc-amino)ethanethiol to afford thioethers with linkers **A** and **B**, respectively. The aryl halides had the following leaving groups: [a]X = F, [b]X = Cl, [c]X = Br, [d]X = Ms.

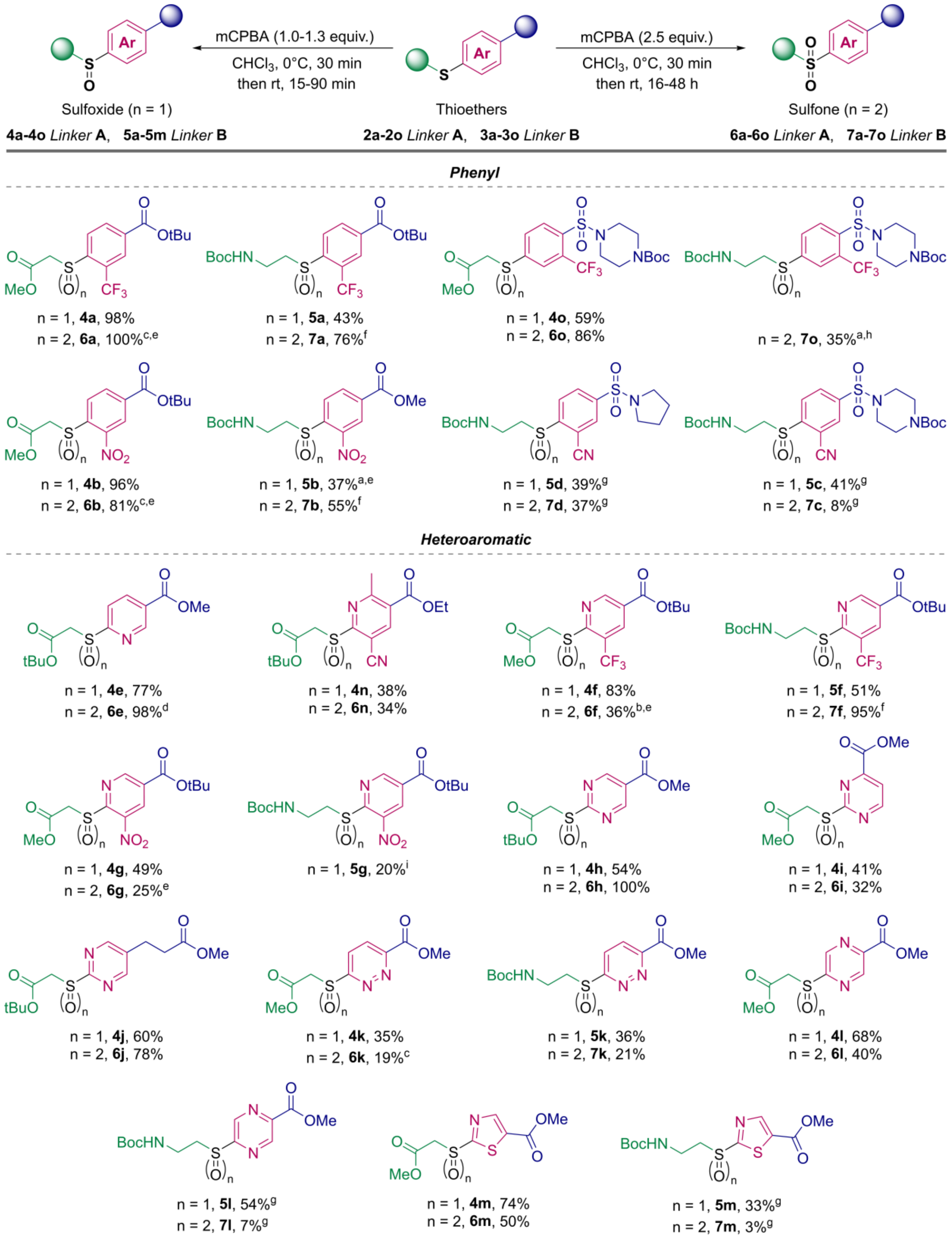

Figure S2. *S*-oxidation of thioethers to sulfoxides and sulfones. [a]2.0 equiv. of mCPBA were used. [b]3.5 equiv. of mCPBA were used. [c]5.0 equiv. of mCPBA were used. [d]Reaction was complete in 4 h at room temperature. [e]After 30 min at 0°C, the reaction mixture was warmed up to room temperature and subsequently heated to 50°C for 24-48 h. [f]The sulfone was obtained by *S*-oxidation of the corresponding isolated sulfoxide. Conditions: mCPBA (3.0 equiv.), $CHCl_3$, 0°C, 30 min, then rt to 50°C, 6-16 h. [g]The sulfoxide and sulfone were obtained from the same reaction mixture by *S*-oxidation of the corresponding thioether. Conditions: mCPBA (1.1-1.5 equiv.), $CHCl_3$, 0°C, 30 min, then rt, 30 min. The reaction was monitored by LC-MS and

quenched with $Na_2S_2O_3$ to obtain a mixture of sulfoxide and sulfone, which was separated by column chromatography. The reaction yield should be regarded as the sum of yields for the sulfoxide and the sulfone. [h]Reaction was complete in 1 h at room temperature. The sulfoxide was not isolated due to rapid overoxidation to the sulfone. [i]The scaffold was difficult to oxidize. The sulfoxide was isolated, while the sulfone was unstable and decomposed.

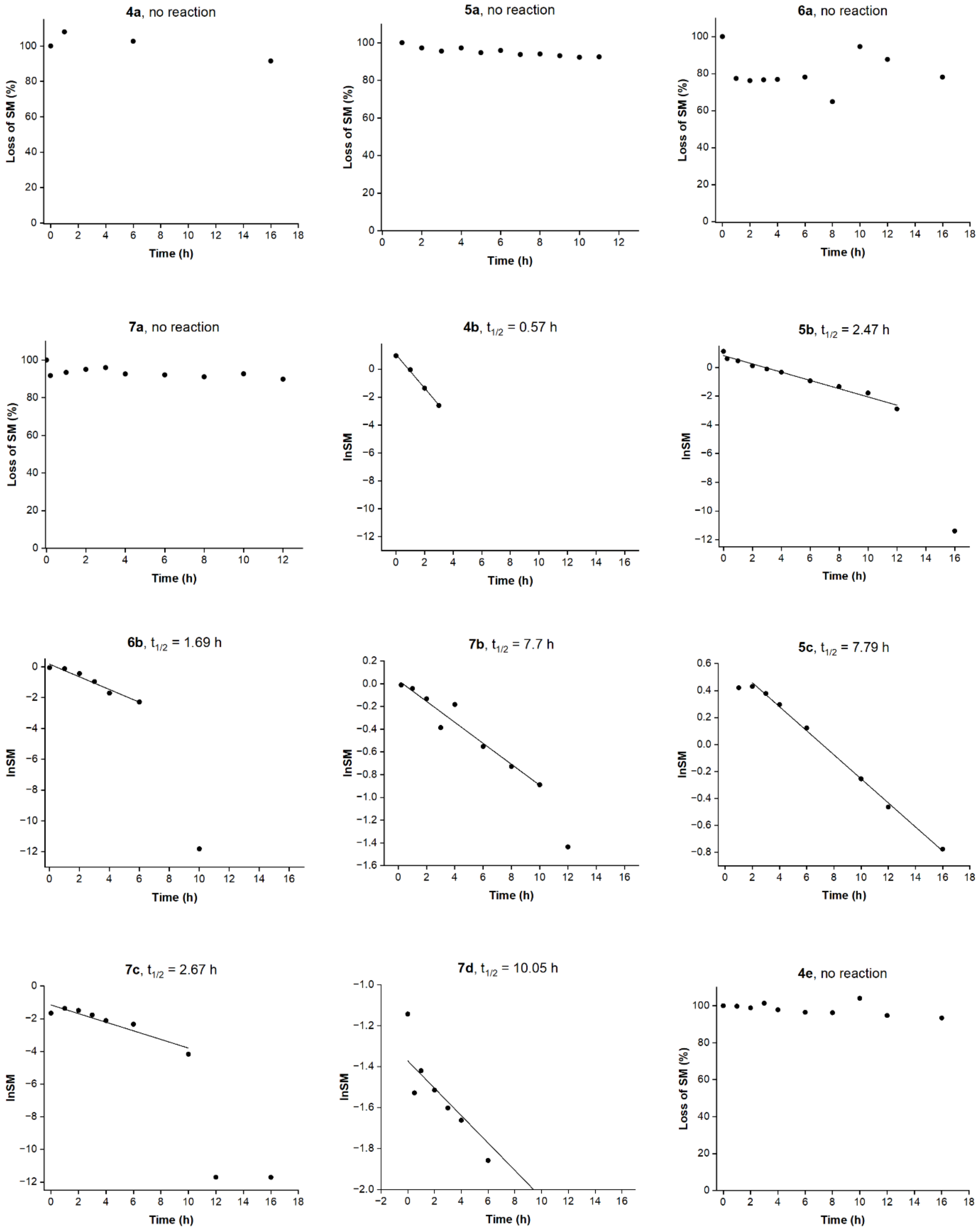
4a, no reaction
Loss of SM (%)
Time (h)
5a, no reaction
Loss of SM (%)
Time (h)
6a, no reaction
Loss of SM (%)
Time (h)
7a, no reaction
Loss of SM (%)
Time (h)
4b, $t_{1/2}$ = 0.57 h
lnSM
Time (h)
5b, $t_{1/2}$ = 2.47 h
lnSM
Time (h)
6b, $t_{1/2}$ = 1.69 h
lnSM
Time (h)
7b, $t_{1/2}$ = 7.7 h
lnSM
Time (h)
5c, $t_{1/2}$ = 7.79 h
lnSM
Time (h)
7c, $t_{1/2}$ = 2.67 h
lnSM
Time (h)
7d, $t_{1/2}$ = 10.05 h
lnSM
Time (h)
4e, no reaction
Loss of SM (%)
Time (h)

6e, no reaction

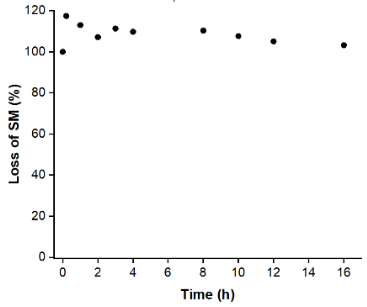

4f, $t_{1/2}$ = 0.05 h

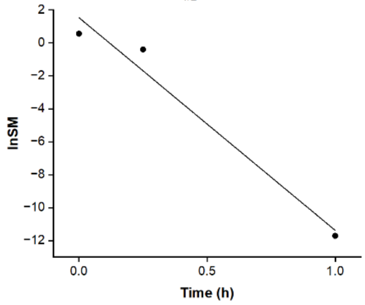

5f, $t_{1/2}$ = 0.38 h

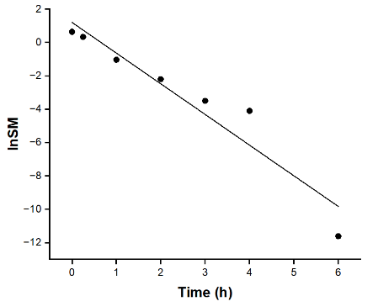

6f, $t_{1/2}$ = 0.06 h

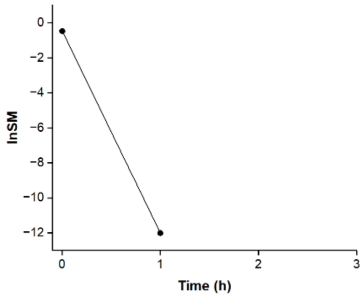

7f, $t_{1/2}$ = 0.06 h

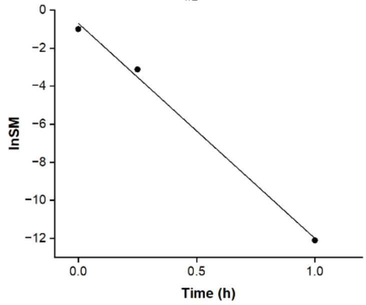

4g, $t_{1/2}$ = 0.06 h

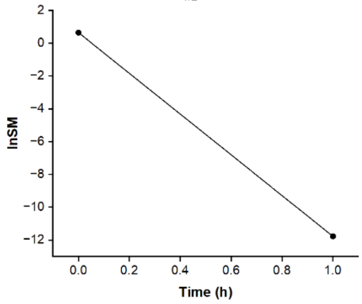

5g, $t_{1/2}$ = 0.03 h

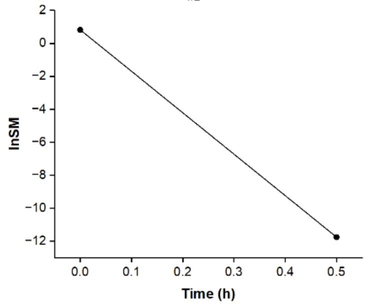

6g, $t_{1/2}$ = 0.03 h

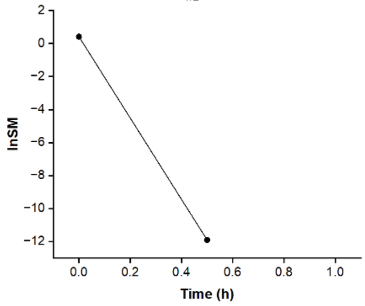

4h, $t_{1/2}$ = 0.01 h

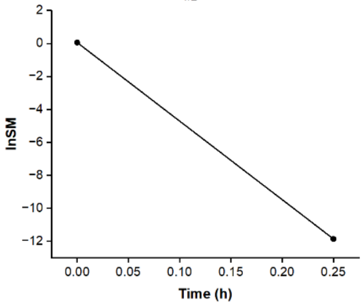

6h, $t_{1/2}$ = 0.01 h

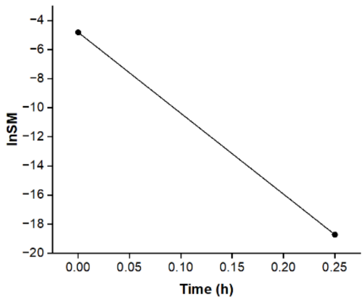

4i, no reaction

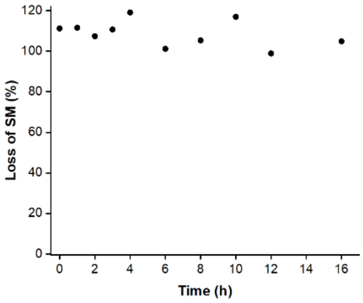

6i, no reaction

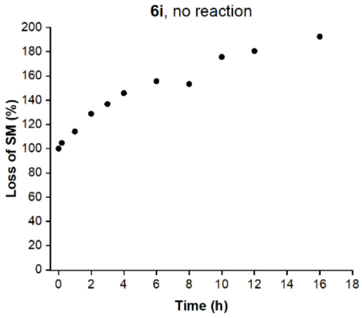

4j, no reaction

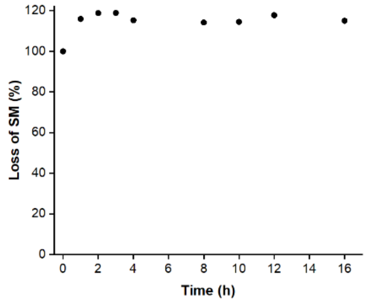

6j, no reaction

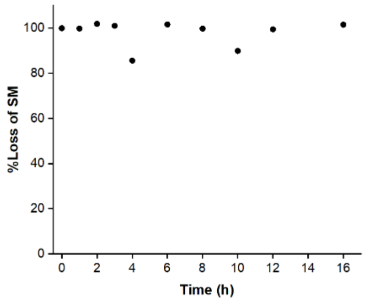

4k, $t_{1/2}$ = 0.38 h

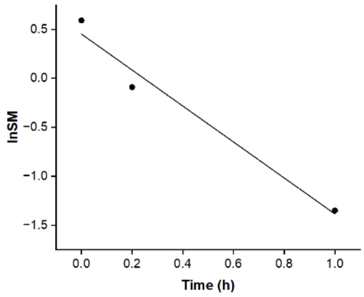

5k, $t_{1/2}$ = 1.51 h

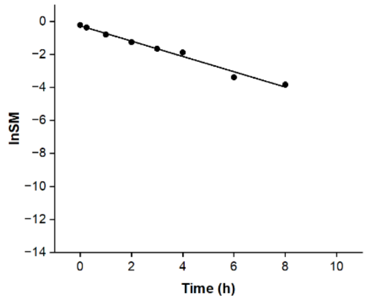

6k, $t_{1/2}$ = 0.06 h

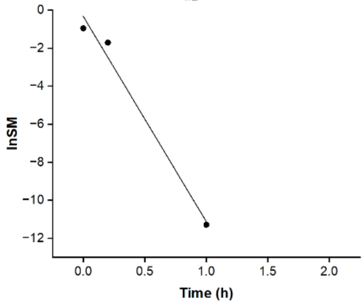

7k, $t_{1/2}$ = 100 h

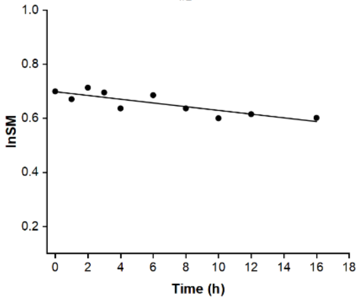

4l, $t_{1/2}$ = 0.05 h

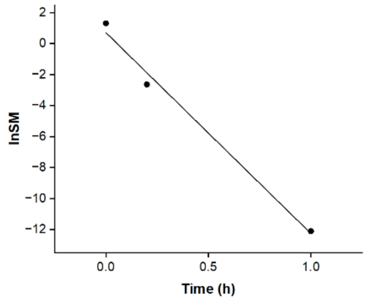

5l, $t_{1/2}$ = 0.01 h

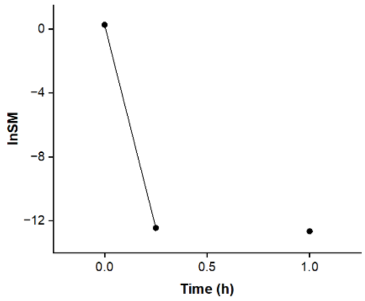

6l, $t_{1/2}$ = 0.06 h

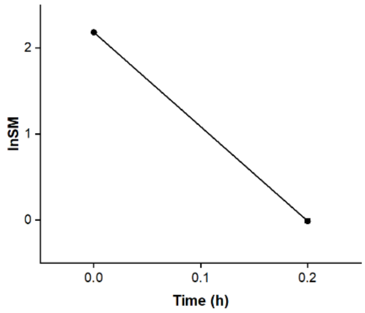

7l, $t_{1/2}$ = 0.12 h

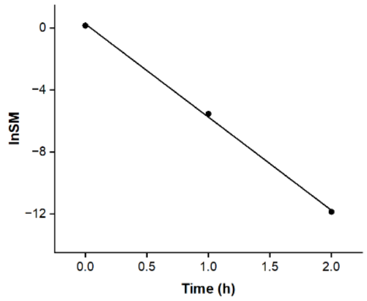

4m, $t_{1/2}$ = 0.05 h

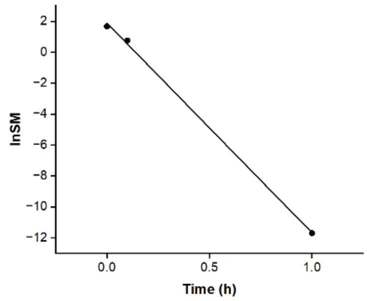

5m, $t_{1/2}$ = 0.24 h

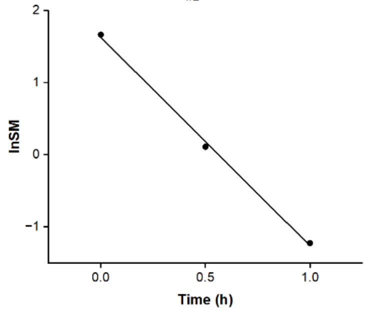

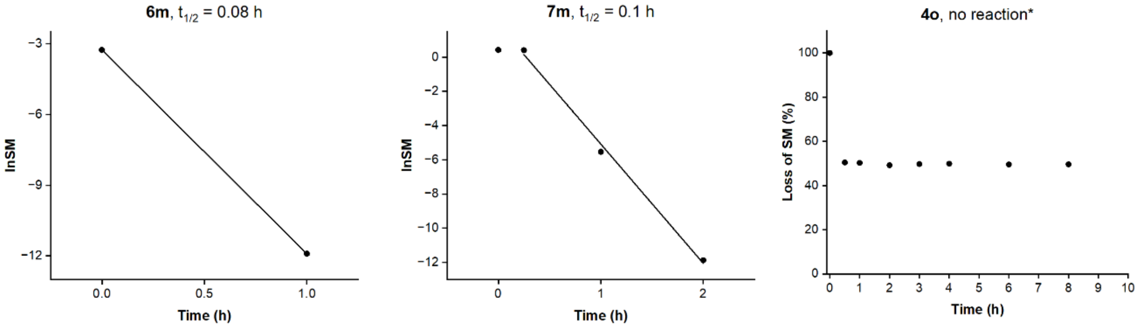

Figure S3. Determination of $t_{1/2}$ of warhead fragments (100 µM) against NAC (5 mM, 50 equiv.) in PBS buffer at pH 7.4. The reactions were monitored using HPLC and measuring the peak area of the electrophile, normalized to the area of an internal standard. The natural logarithm of the remaining starting material (lnSM) over time was fitted to linear regression, and $t_{1/2}$ was calculated as $t_{1/2}$ = ln (2)/–slope. Compound **6i** did not react with NAC and the apparent increase in peak area of the electrophile was due to issues with the HPLC baseline. Compound **4o** did not react with NAC and the apparent decrease in the peak area of the electrophile was due to solubility issues. Similarly, compounds **6o** and **7o** were not tested due to low solubility in buffer.

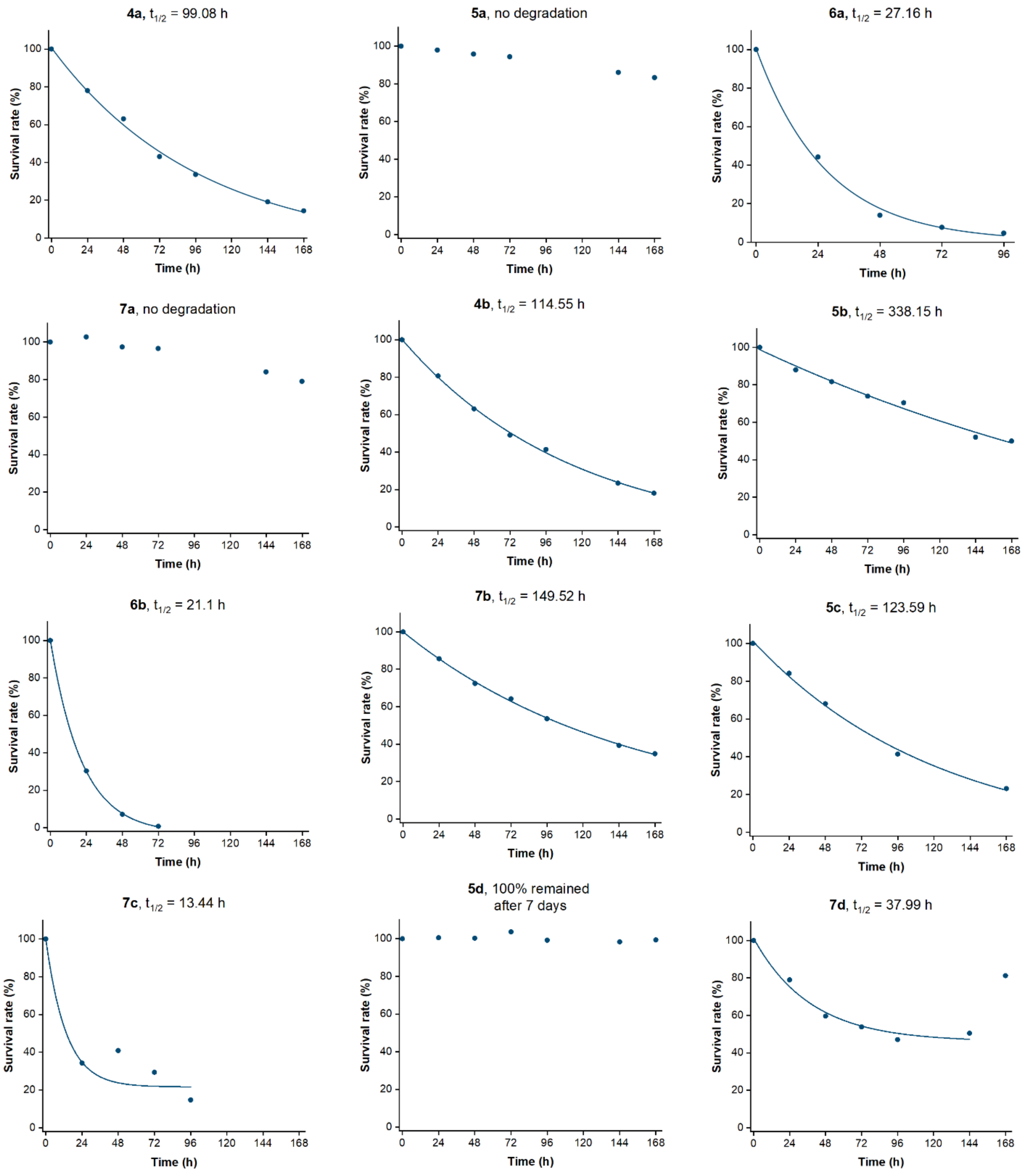

4a, t1/2 = 99.08 h
5a, no degradation
6a, t1/2 = 27.16 h
7a, no degradation
4b, t1/2 = 114.55 h
5b, t1/2 = 338.15 h
6b, t1/2 = 21.1 h
7b, t1/2 = 149.52 h
5c, t1/2 = 123.59 h
7c, t1/2 = 13.44 h
5d, 100% remained after 7 days
7d, t1/2 = 37.99 h
Survival rate (%)
Time (h)

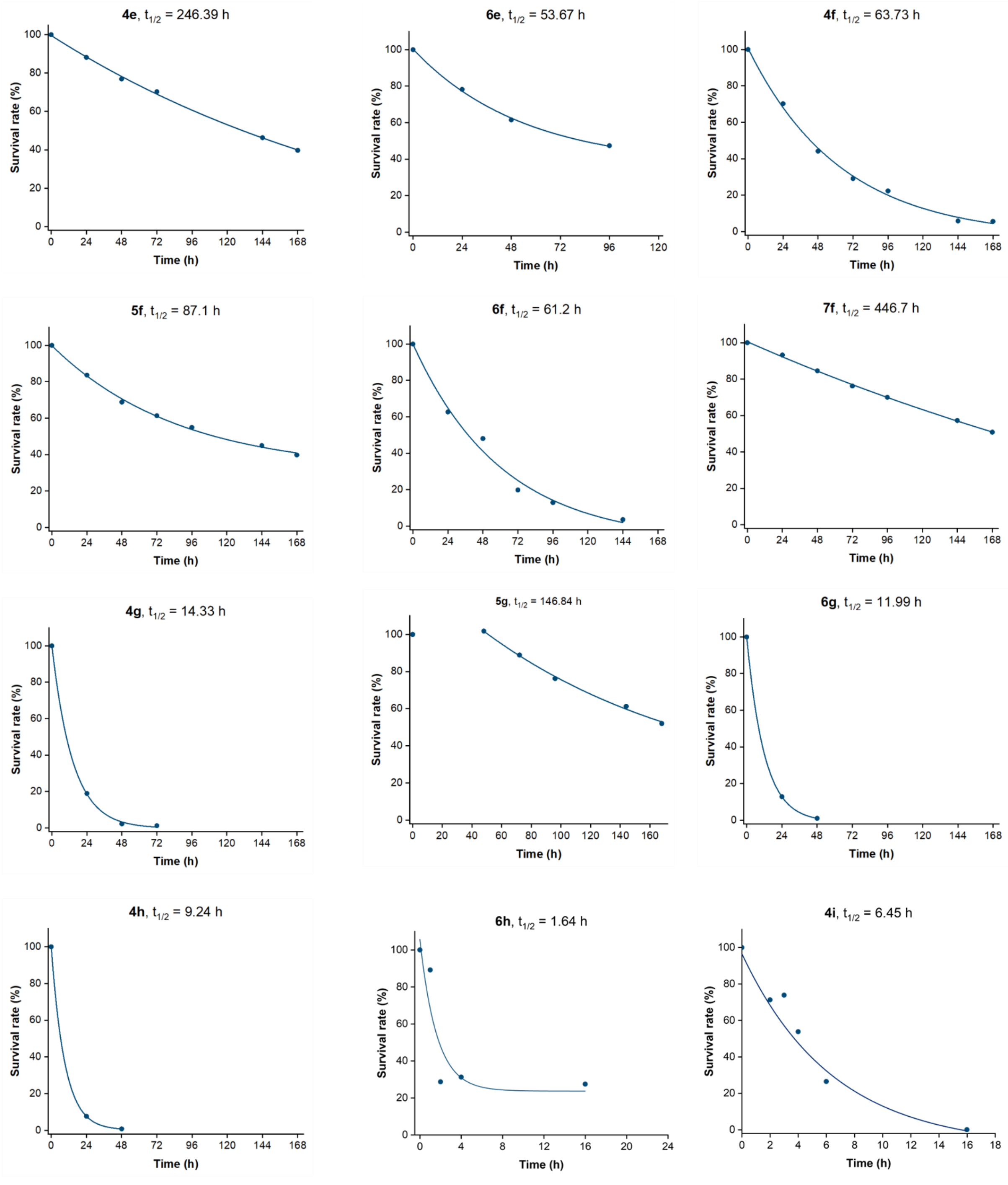
4e, t1/2 = 246.39 h
6e, t1/2 = 53.67 h
4f, t1/2 = 63.73 h
5f, t1/2 = 87.1 h
6f, t1/2 = 61.2 h
7f, t1/2 = 446.7 h
4g, t1/2 = 14.33 h
5g, t1/2 = 146.84 h
6g, t1/2 = 11.99 h
4h, t1/2 = 9.24 h
6h, t1/2 = 1.64 h
4i, t1/2 = 6.45 h
Survival rate (%)
Time (h)

6i, $t_{1/2}$ = 7.7 h

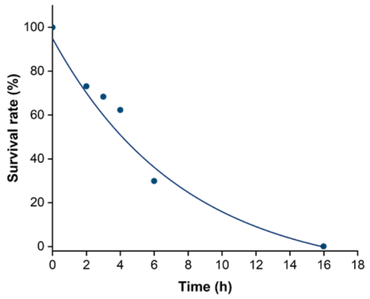

4j, no degradation

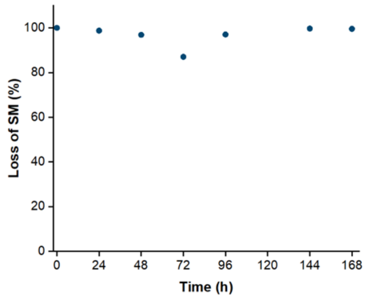

6j, 60% remained after 6 days

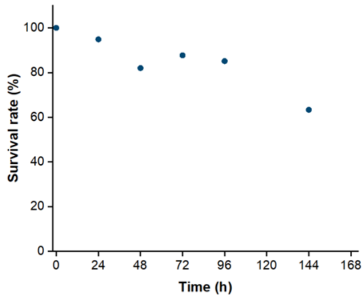

4k, $t_{1/2}$ = 13.37 h

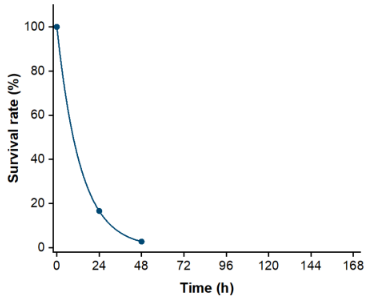

5k, $t_{1/2}$ = 19.87 h

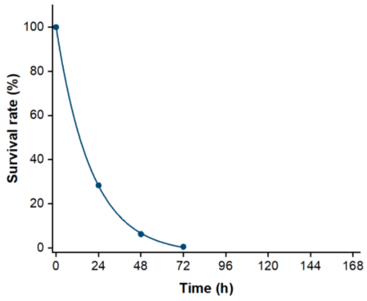

6k, $t_{1/2}$ = 4.47 h

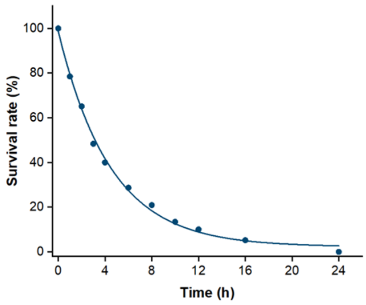

7k, $t_{1/2}$ = 7.23 h

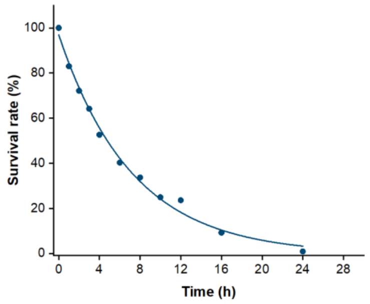

4l, $t_{1/2}$ = 20.47 h

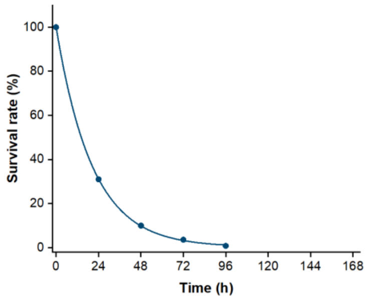

5l, $t_{1/2}$ = 25.2 h

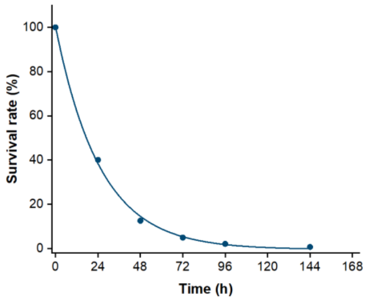

6l, $t_{1/2}$ = 7.01 h

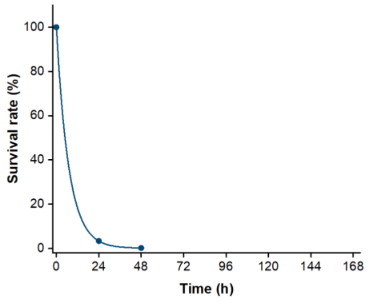

7l, $t_{1/2}$ = 7.03 h

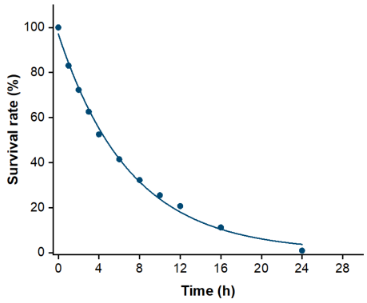

4m, $t_{1/2}$ = 46.19 h

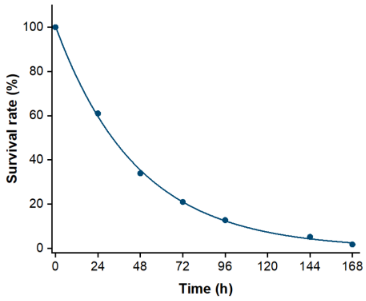

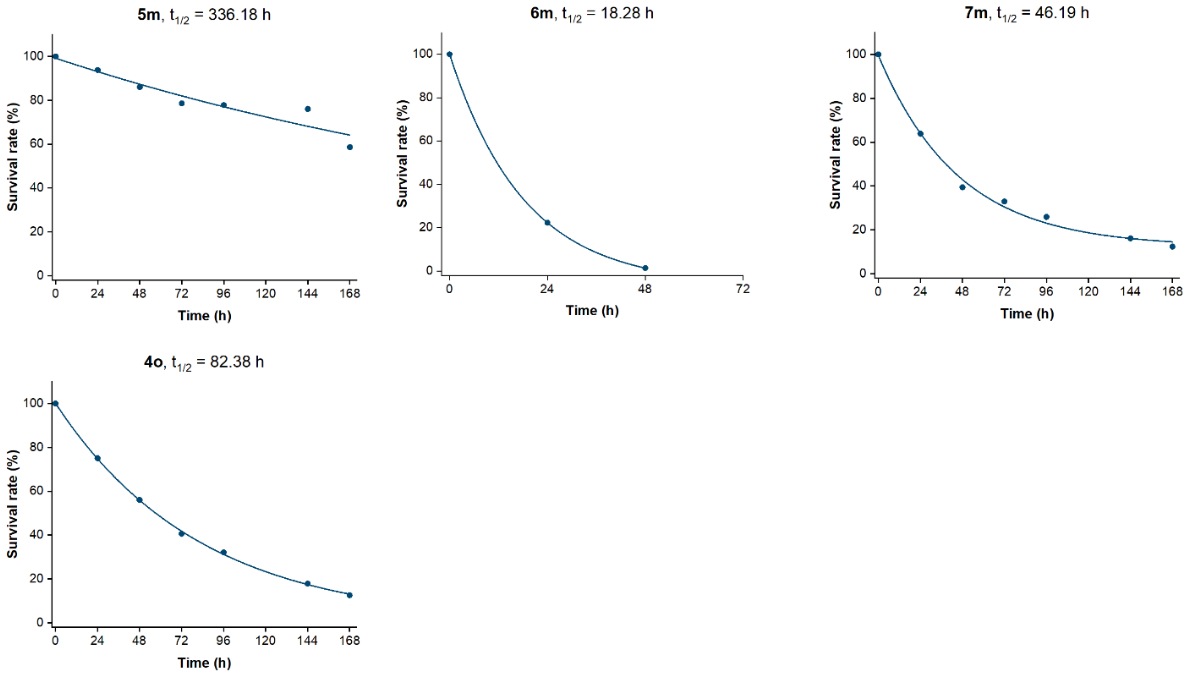

Figure S4. Time course plots of hydrolysis reaction in PBS buffer at pH 7.4. The reactions were monitored using HPLC and measuring the peak area of the electrophile (100 μM), normalized to the area of an internal standard. Survival rate over time was fitted to a single-phase exponential decay model to determine the $t_{1/2}$ of the warhead fragment in PBS buffer. Compounds **6o** and **7o** were not tested due to low solubility in buffer.

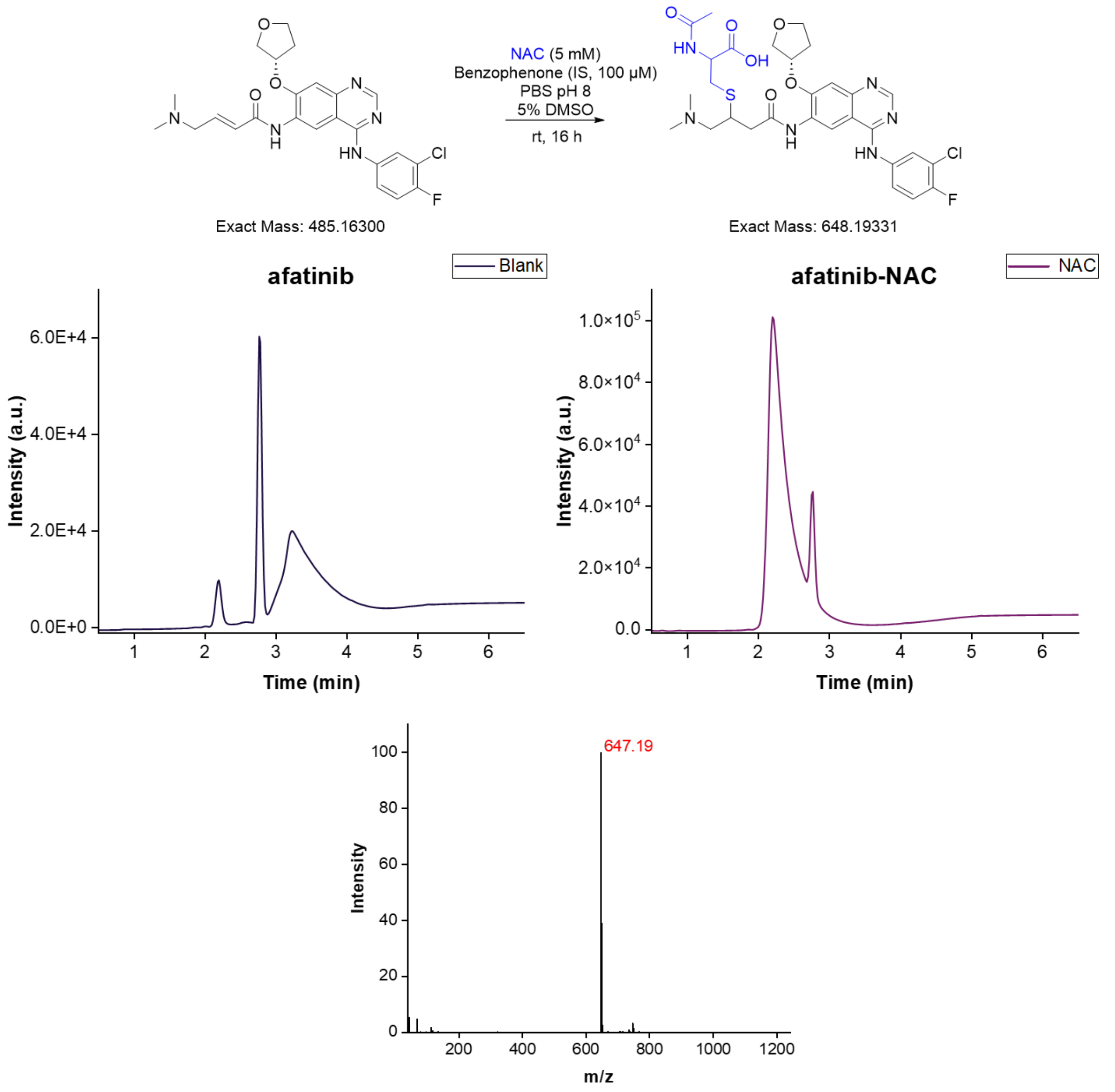

Figure S5. UV (sum of absorption at 230 nm and 254 nm) and MS spectra of the LC-MS analysis of 100 µM of afatinib incubated with 5 mM NAC for 16 h in PBS buffer pH 8 containing 5% DMSO at room temperature. The blank sample is shown in black while the reaction in magenta. Internal standard: RT = 2.7 min; afatinib: RT = 2.3 min, NAC-adduct: RT = 2.2 min, *m/z* = 647.19 [M-H]$^-$.

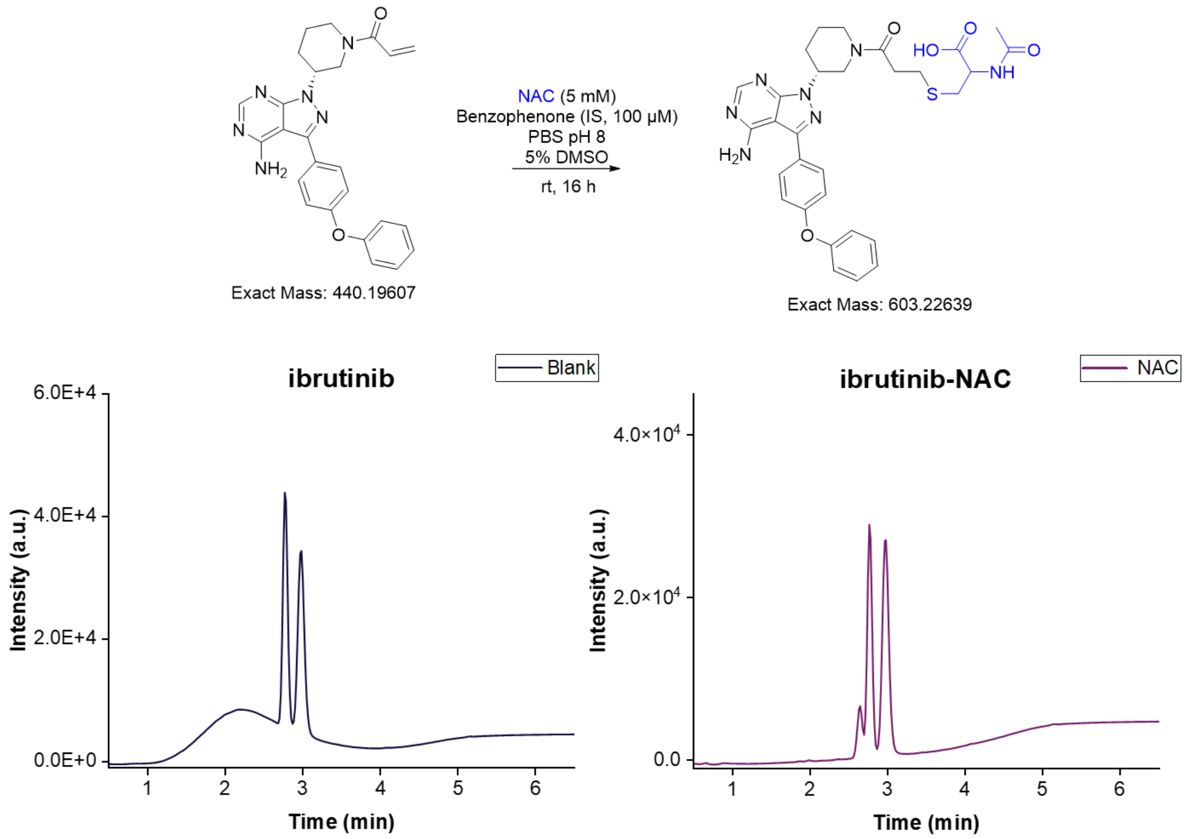

Figure S6. UV (sum of absorption at 230 nm and 254 nm) and MS spectra of the LC-MS analysis of 100 µM of ibrutinib incubated with 5 mM NAC for 16 h in PBS buffer pH 8 containing 5% DMSO at room temperature. The blank sample is shown in black while the reaction in magenta. Internal standard: RT = 2.7 min; ibrutinib: RT = 2.6 min.

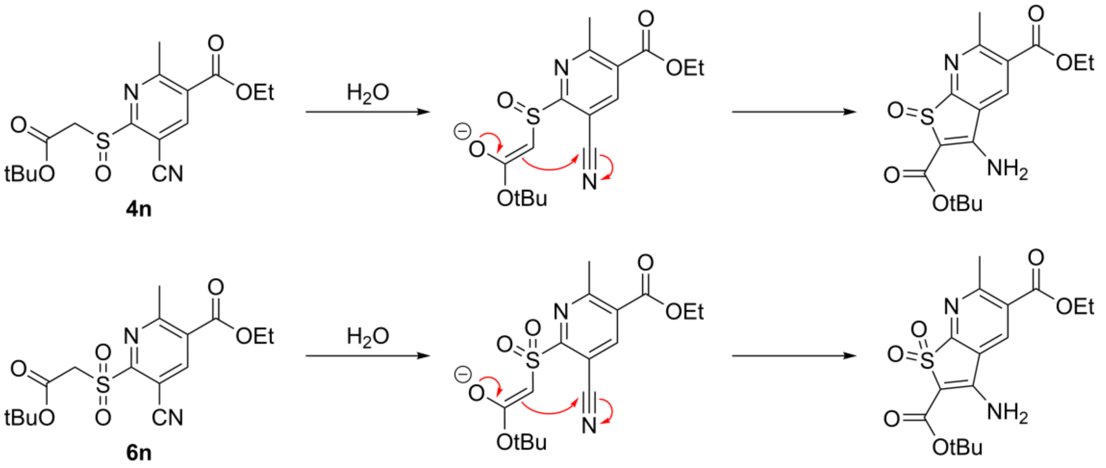

Scheme S5. Cyanopyridines **4n** and **6n** were not stable in aqueous solutions. The acidic protons between the ester and sulfur atom are deprotonated to form an enolate that undergoes nucleophilic addition to the nitrile to form a [5,6]-bicyclic compound. LC-MS showed complete cyclization after 1 h.

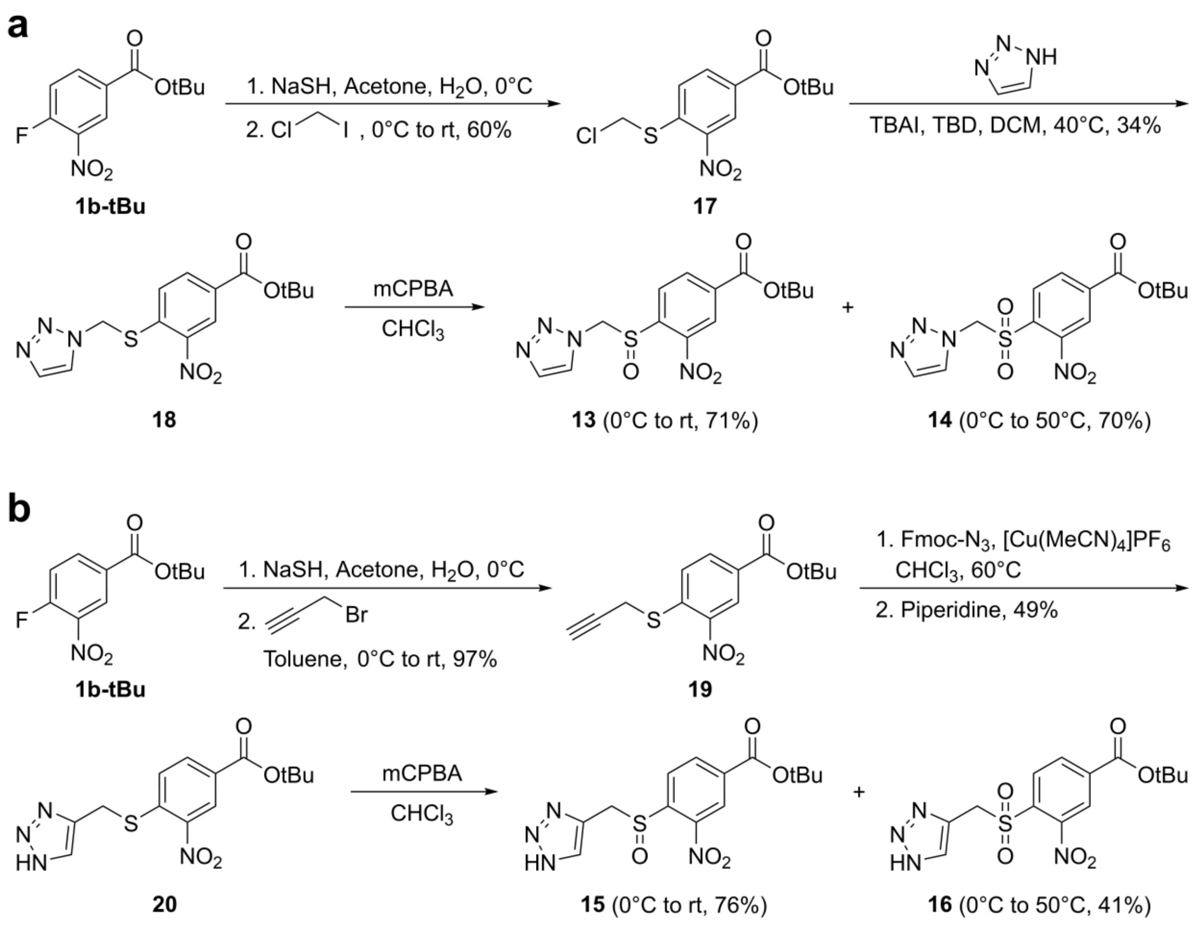

Scheme S6. Synthesis of model warheads of the nitrophenyl scaffold with triazole linker **C**. A pair of sulfoxide/sulfone was prepared for both 1-substituted (a) and 4-substituted (b) triazoles.

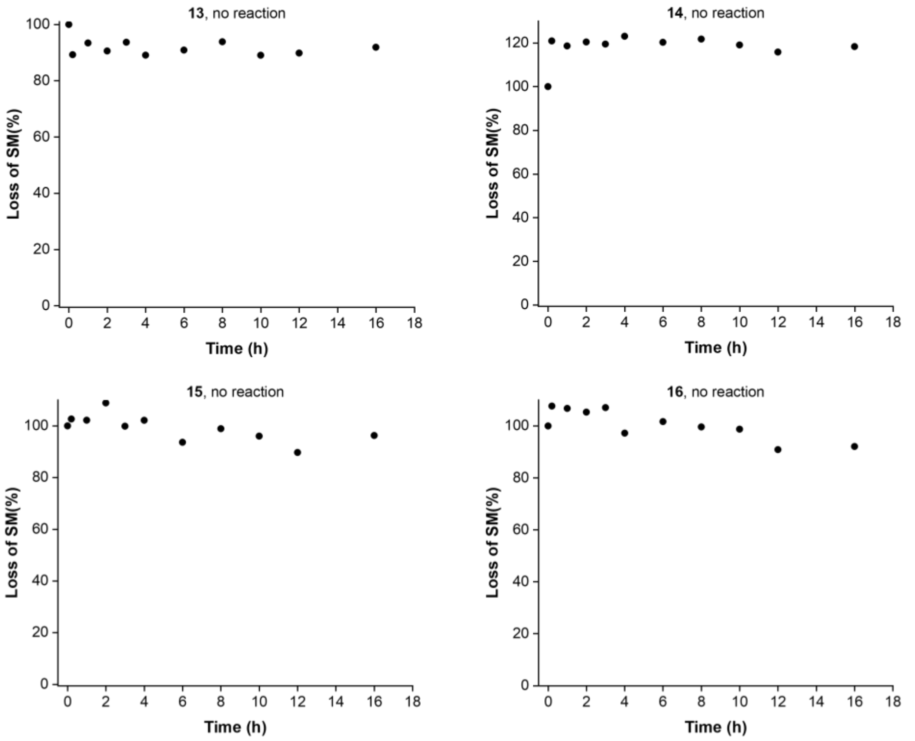

Figure S7. Reactivity plots of triazole warheads **13**-**16** (100 µM) against NAC (5 mM, 50 equiv.) in PBS buffer at pH 7.4. The reactions were monitored using HPLC with an internal standard. The compounds showed no reactivity.

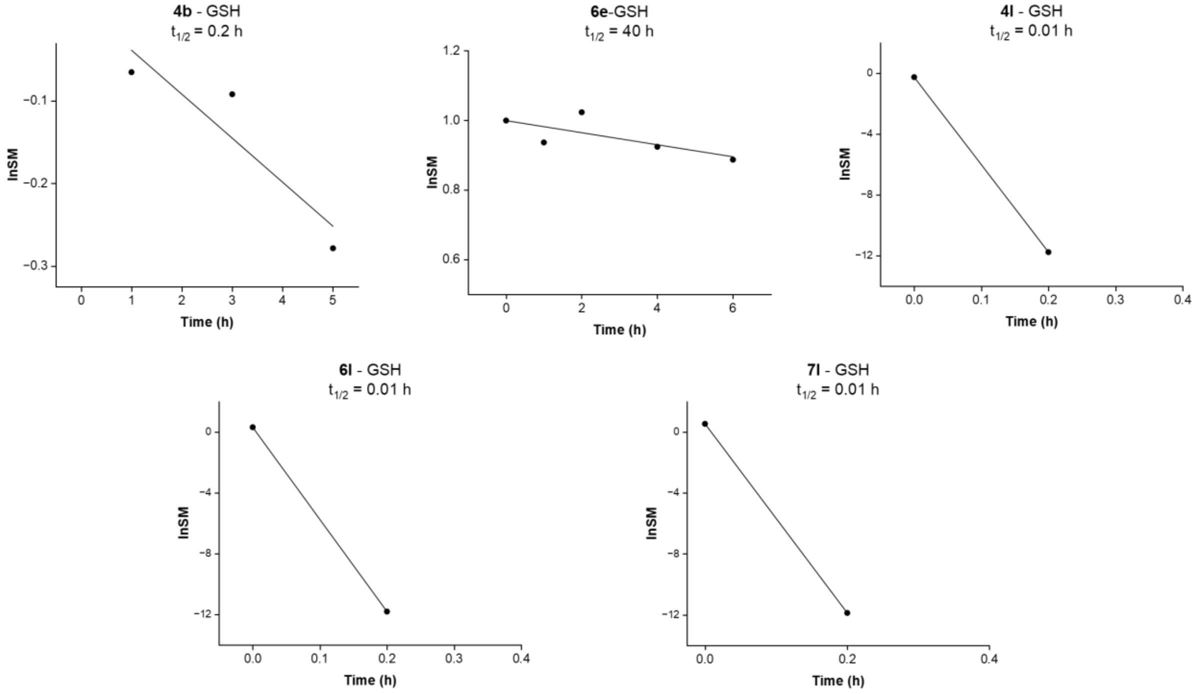

Figure S8. Determination of $t_{1/2}$ of selected warhead fragments (100 µM) against GSH (5 mM, 50 equiv.) in PBS buffer at pH 7.4. The reactions were monitored using HPLC and measuring the peak area of the electrophile, normalized to the area of an internal standard. The natural logarithm of the remaining starting material (lnSM) over time was fitted to linear regression, and $t_{1/2}$ was calculated as $t_{1/2}$ = ln (2)/–slope.

Compound	$t_{1/2}$ NAC (h)	$t_{1/2}$ GSH (h)
**4b**	0.58	0.2
**6e**	n.r.	40
**4l**	0.05	0.01
**6l**	0.06	0.01
**7l**	0.12	0.01

Table S1. Summary of NAC and GSH $t_{1/2}$ for selected scaffolds. n.r. = no reaction.

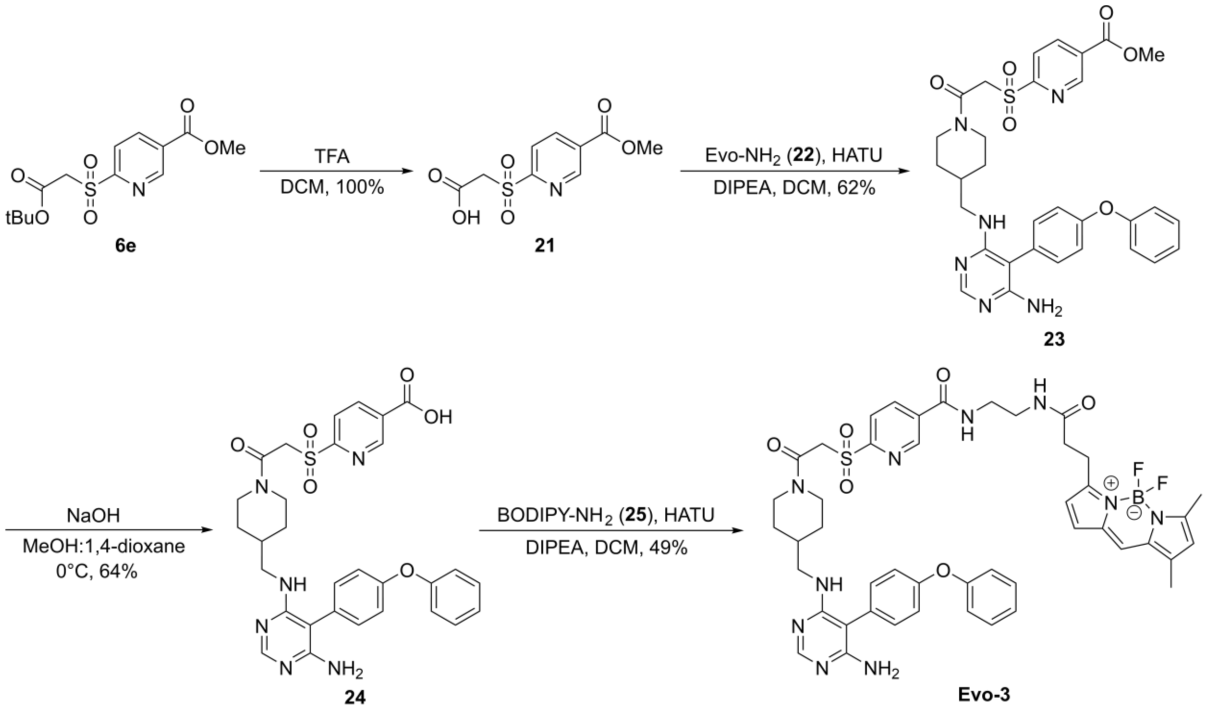

Scheme S7. Synthesis of BTK probe **Evo-3**.

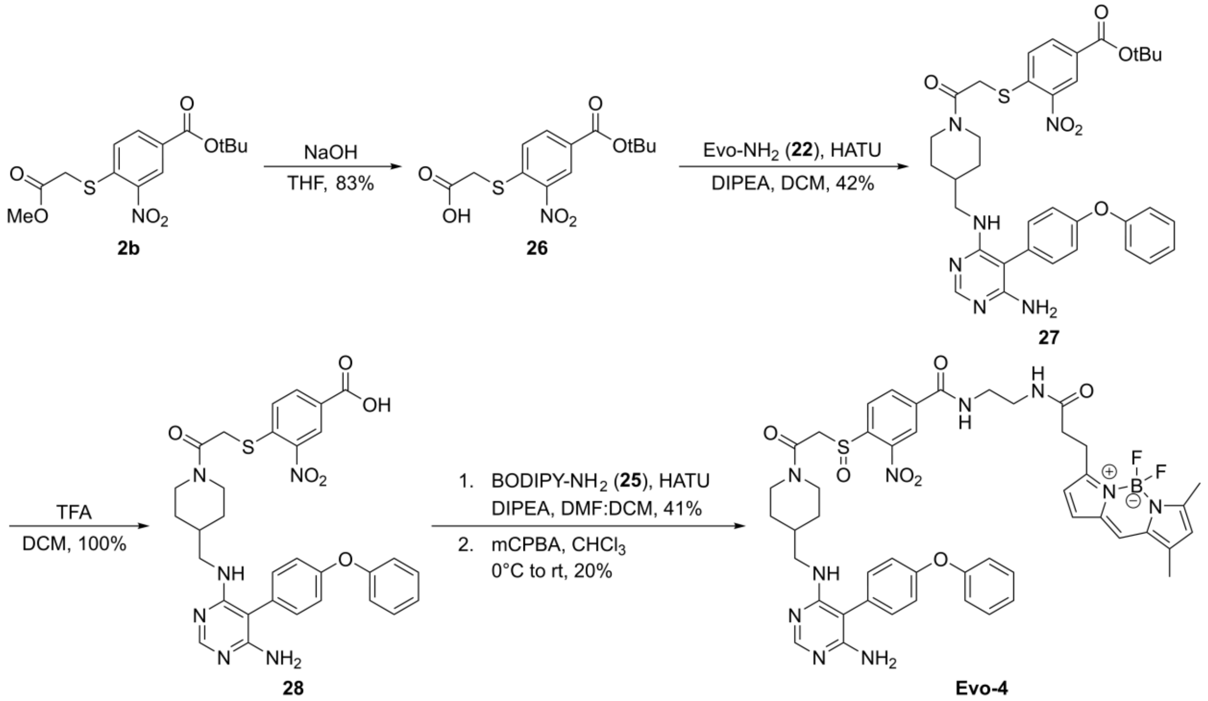

Scheme S8. Synthesis of BTK probe **Evo-4**.

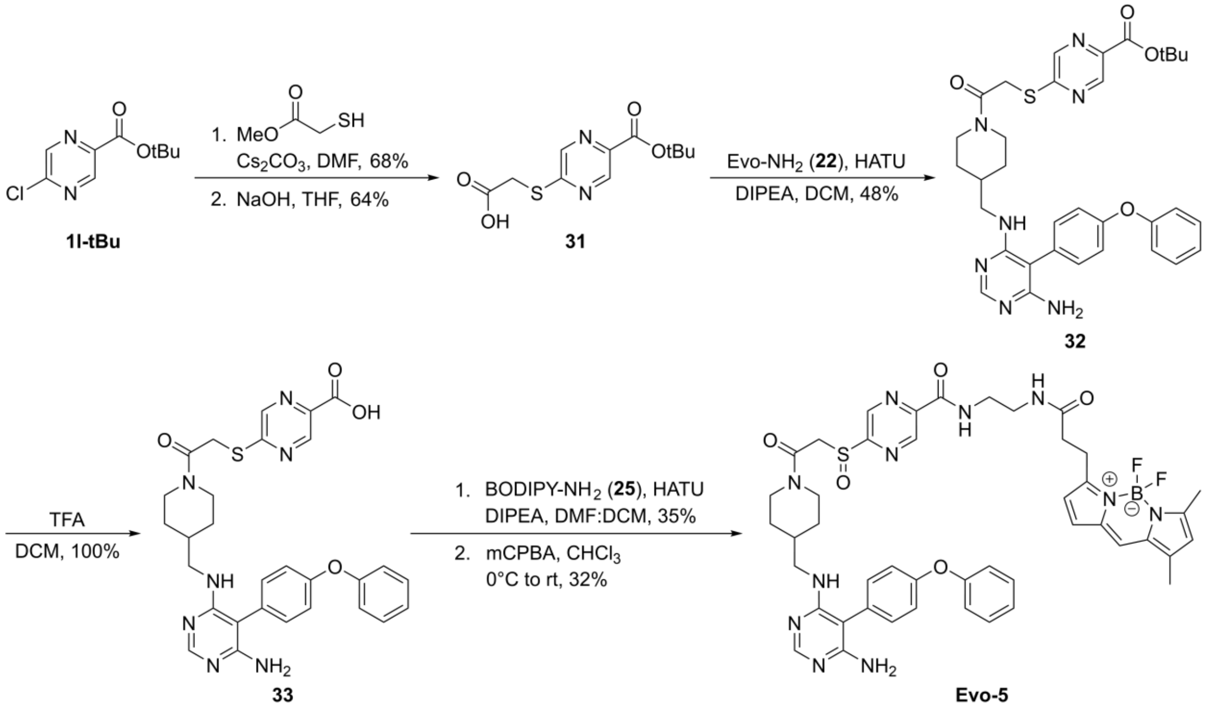

Scheme S9. Synthesis of BTK probe **Evo-5**.

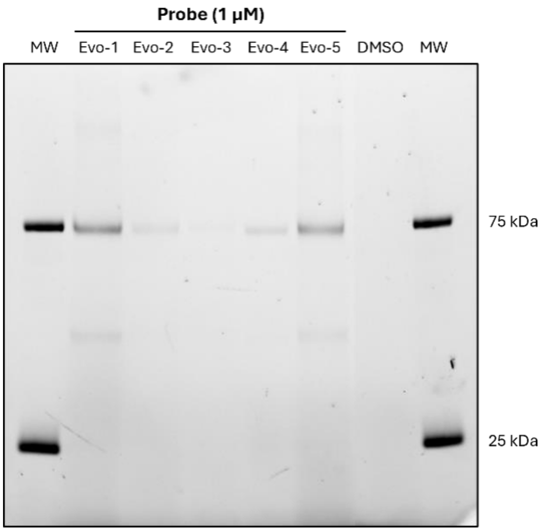

Figure S9. In-gel fluorescence scanning shows labeling of recombinant BTK by BODIPY-functionalized probes. Recombinant BTK (100 ng) was incubated with 1 μM of **Evo-1** to **Evo-5** for 1 h at room temperature and separated by SDS-PAGE. The gel was imaged with ChemiDoc imaging system (blue LED, 530/28 filter). The molecular weights (MWs) of the fluorescent markers are shown to the right of the gel.

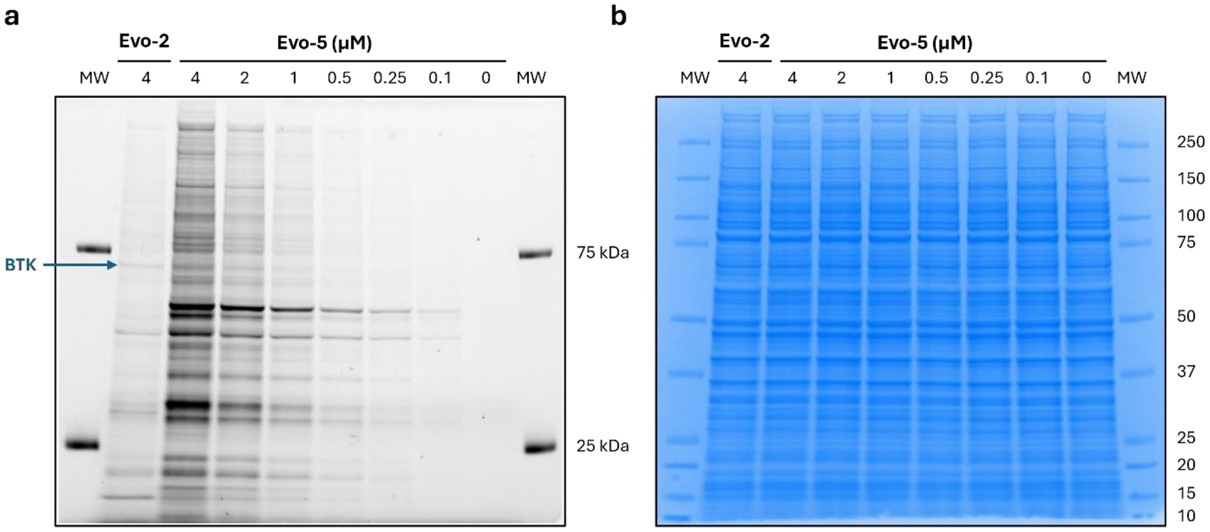

Figure S10. Cellular protein labeling profile of **Evo-5** shows labeling of BTK with low selectivity. (a) Ramos cells were incubated with 0.1-4 μM of **Evo-5** or 4 μM **Evo-2** as reference for 2 h. The cells were washed, lysed, and cellular proteins were separated by SDS-PAGE under denatured and reduced conditions. The gel was imaged with ChemiDoc imaging system (blue LED, 530/28 filter) to detect fluorescently labeled proteins. The blue arrow indicates the BTK band. The molecular weights (MWs) of the fluorescent markers are shown to the right of the gel. (b) Total protein staining of the same gel with SimplyBlue. The MWs of the protein markers are shown to the right of the gel.

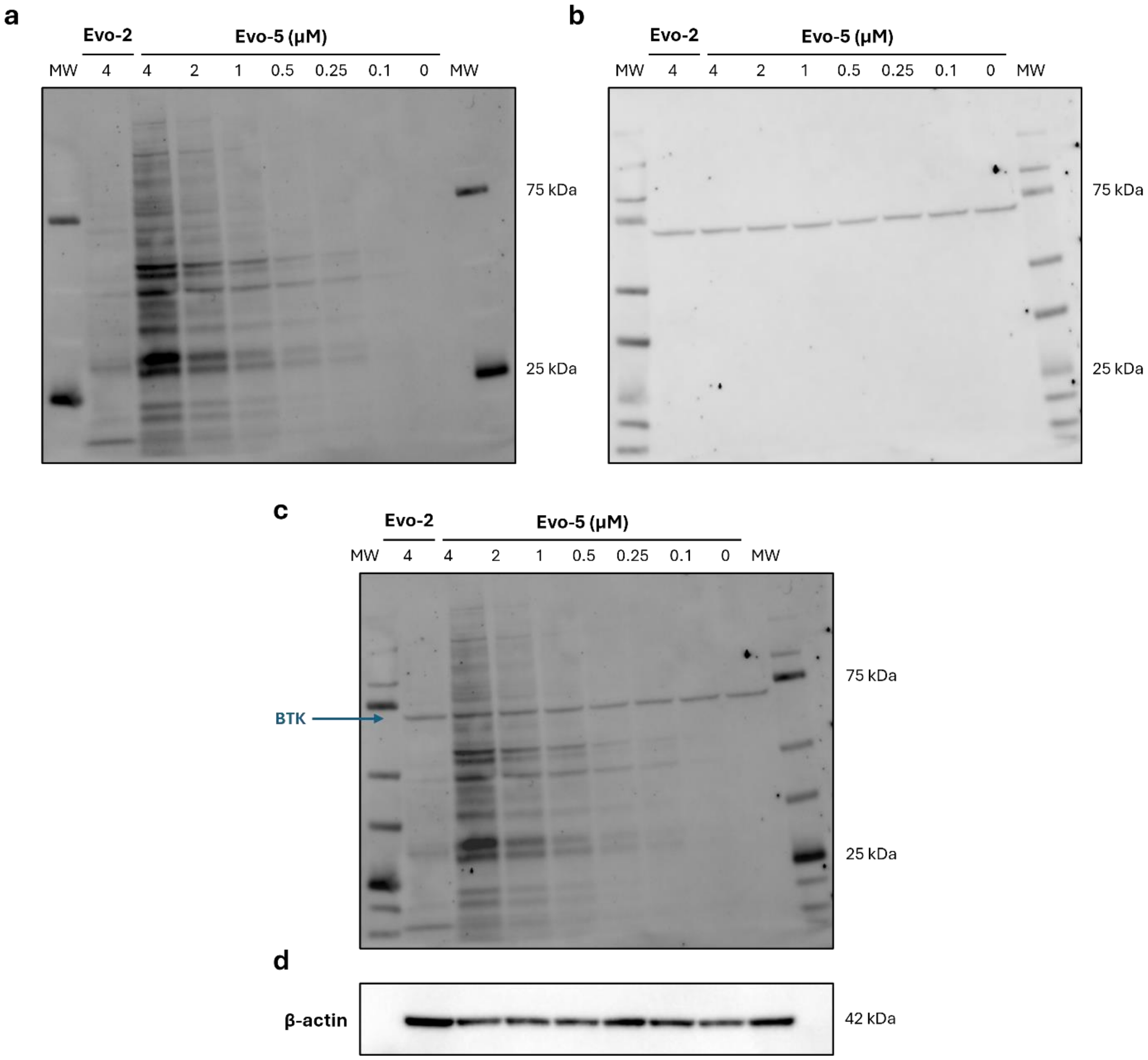

Figure S11. Identification of the BTK band in Ramos cells treated with **Evo-5** using fluorescent Western blot. An identical gel as in Figure S6 was transferred to a nitrocellulose membrane for immunoblotting. (a) Fluorescent signal on membrane corresponding to BODIPY-labeled proteins imaged with ChemiDoc imaging system (blue LED, 530/28 filter). (b) Immunoblotting of BTK with mouse anti-BTK primary antibody followed by goat anti-mouse Alexa633 secondary antibody. Fluorescent signal on membrane corresponding to BTK cross-coupled with Alexa633 imaged with ChemiDoc imaging system (red LED, 695/50 filter). (c) Merged picture of the two channels confirm binding of **Evo-2** and **Evo-5** to BTK. The blue arrow indicates the BTK band. The molecular weights (MWs) of the fluorescent markers are shown to the right of the gel. (d) Membrane was stripped and re-probed against β-actin using mouse anti-β-actin HRP-conjugated antibody and chemiluminescence detection.

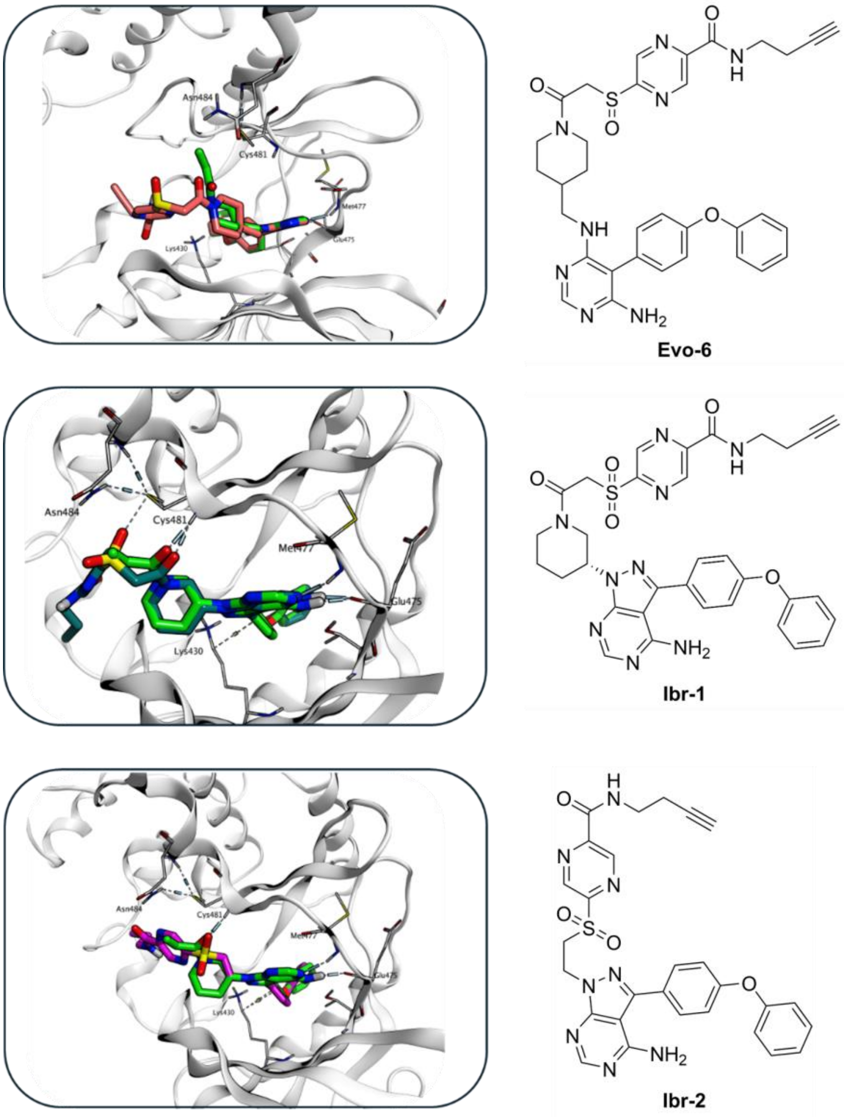

Figure S12. Docking poses of **Evo-6** (salmon-pink carbons), **Ibr-1** (petrol carbons), and **Ibr-2** (fuchsia carbons) bound to the kinase domain of BTK, superimposed on the parent ligand (light-green carbons). For **Evo-6**, the crystal structure of evobrutinib (PDB ID: 6OMU) was used, whereas for **Ibr-1** and **Ibr-2** the crystal structure of the non-covalent ibrutinib analogue **Ibr-NH** was used (PDB ID: 5P9I).

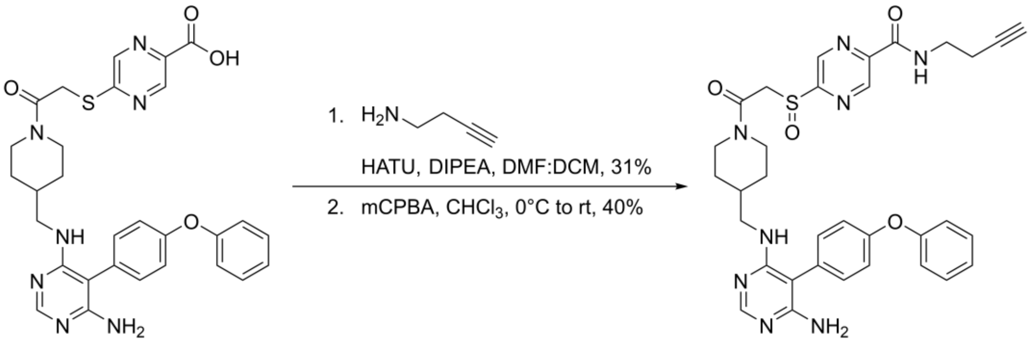

33 **Evo-6**

Scheme S10. Synthesis of BTK probe **Evo-6**.

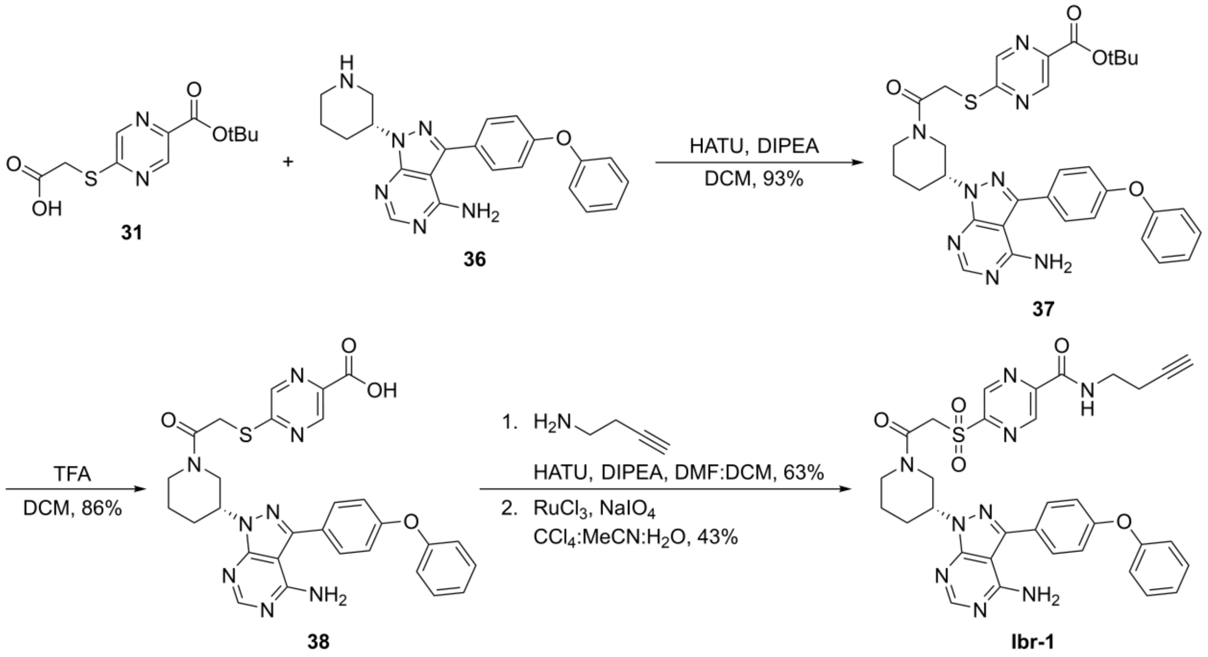

Scheme S11. Synthesis of BTK probe **Ibr-1**.

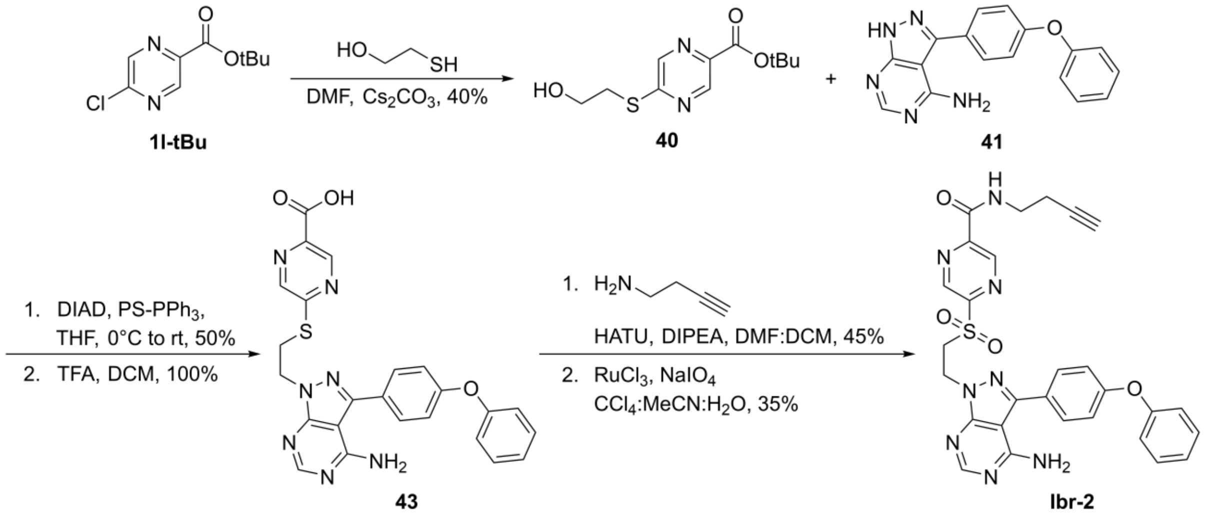

Scheme S12. Synthesis of BTK probe **Ibr-2**.

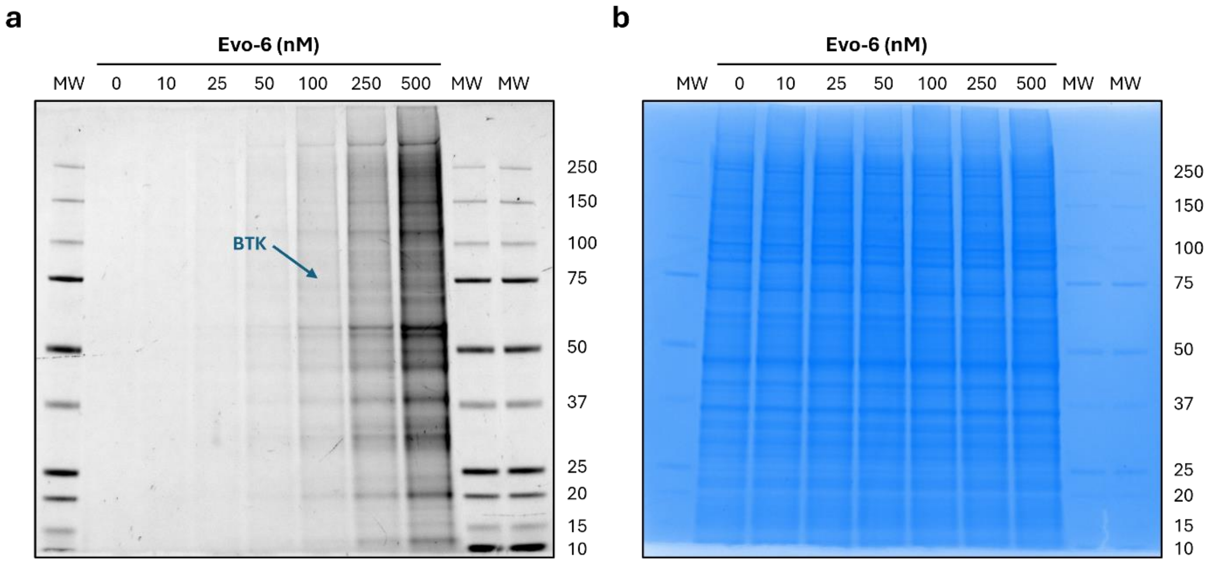

Figure S13. Cellular protein labeling profile after incubation with different concentrations of **Evo-6**. (a) Ramos cells were incubated with 10-500 nM of **Evo-6** for 1 h. The cells were washed, lysed, and the resulting lysate was modified by click reaction with TAMRA-N_3 followed by precipitation with acetone. Proteins were separated by SDS-PAGE under denatured and reduced conditions. The gel was imaged with ChemiDoc imaging system at two channels (green LED, 605/50 filter for TAMRA, red LED 695/50 filter for MW markers). Images were merged to generate the composite image. The blue arrow indicates the BTK band. The molecular weights (MWs) of the fluorescent markers are shown to the right of the gel. (b) Total protein staining of the same gel with SimplyBlue. The MWs of the protein markers are shown to the right of the gel.

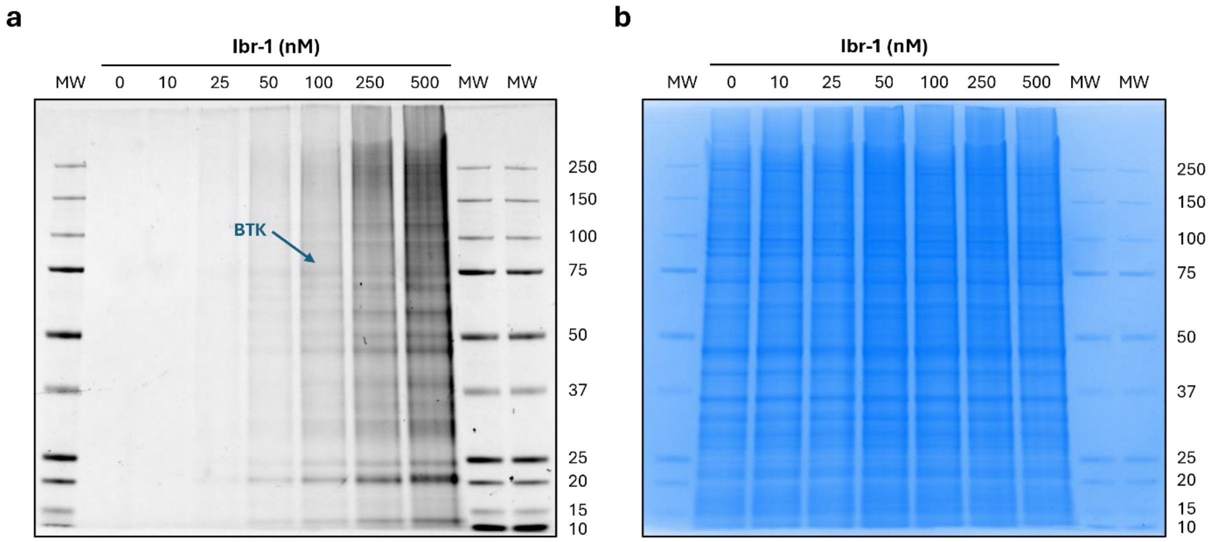

Figure S14. Cellular protein labeling profile after incubation with different concentrations of **Ibr-1**. (a) Ramos cells were incubated with 10-500 nM of **Ibr-1** for 1 h. The cells were washed, lysed, and the resulting lysate was modified by click reaction with TAMRA-N_3 followed by precipitation with acetone. Proteins were separated by SDS-PAGE under denatured and reduced conditions. The gel was imaged with ChemiDoc imaging system at two channels (green LED, 605/50 filter for TAMRA, red LED 695/50 filter for MW markers). Images were merged to generate the composite image. The blue arrow indicates the BTK band. The molecular weights (MWs) of the fluorescent markers are shown to the right of the gel. (b) Total protein staining of the same gel with SimplyBlue. The MWs of the protein markers are shown to the right of the gel.

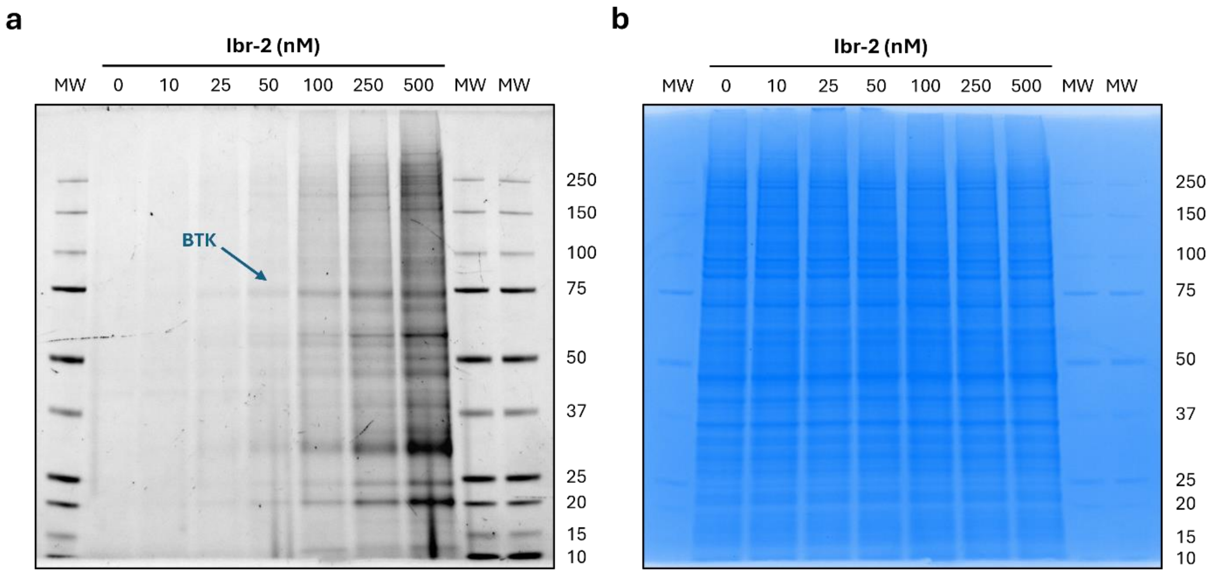

Figure S15. Cellular protein labeling profile after incubation with different concentrations of **Ibr-2**. (a) Ramos cells were incubated with 10-500 nM of **Ibr-2** for 1 h. The cells were washed, lysed, and the resulting lysate was modified by click reaction with TAMRA-N_3 followed by precipitation with acetone. Proteins were separated by SDS-PAGE under denatured and reduced conditions. The gel was imaged with ChemiDoc imaging system at two channels (green LED, 605/50 filter for TAMRA, red LED 695/50 filter for MW markers). Images were merged to generate the composite image. The blue arrow indicates the BTK band. The molecular weights (MWs) of the fluorescent markers are shown to the right of the gel. (b) Total protein staining of the same gel with SimplyBlue. The MWs of the protein markers are shown to the right of the gel.

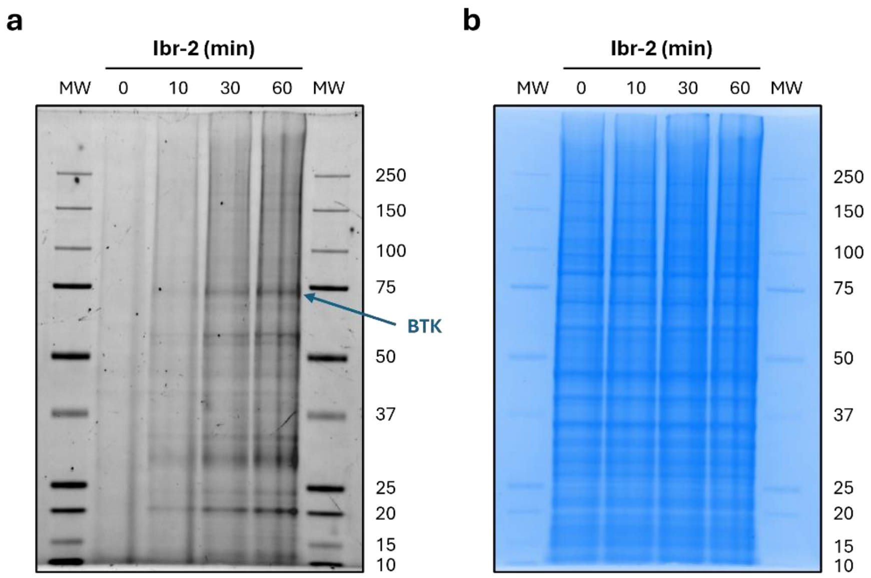

Figure S16. Time-dependent labeling profile of **Ibr-2** using different incubation times. Ramos cells were incubated with 100 nM of **Ibr-2** for 10, 30, and 60 min. The cells were washed, lysed, and the resulting lysate was modified by click reaction with TAMRA-N_3 followed by precipitation with acetone. Proteins were separated by SDS-PAGE under denatured and reduced conditions. The gel was imaged with ChemiDoc imaging system at two channels (green LED, 605/50 filter for TAMRA, red LED 695/50 filter for MW markers). Images were merged to generate the composite image. The blue arrow indicates the BTK band. The molecular weights (MWs) of the fluorescent markers are shown to the right of the gel. (b) Total protein staining of the same gel with SimplyBlue. The MWs of the protein markers are shown to the right of the gel.

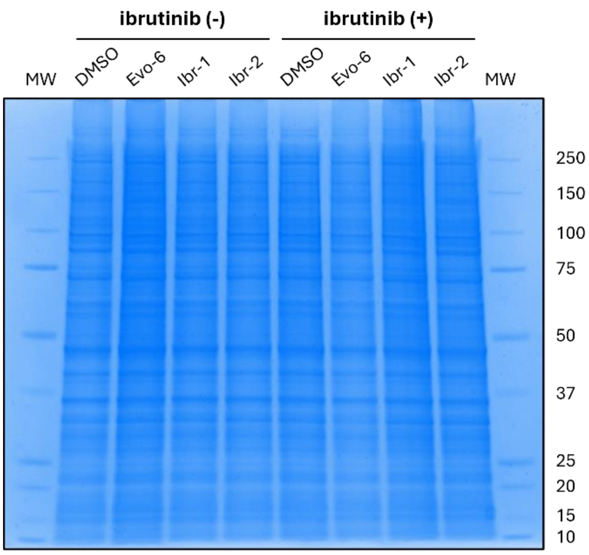

Figure S17. Competition experiment of **Evo-6**, **Ibr-1**, and **Ibr-2** with ibrutinib. Total protein staining of the gel shown in Figure 8b with SimplyBlue. The MWs of the protein markers are shown to the right of the gel.

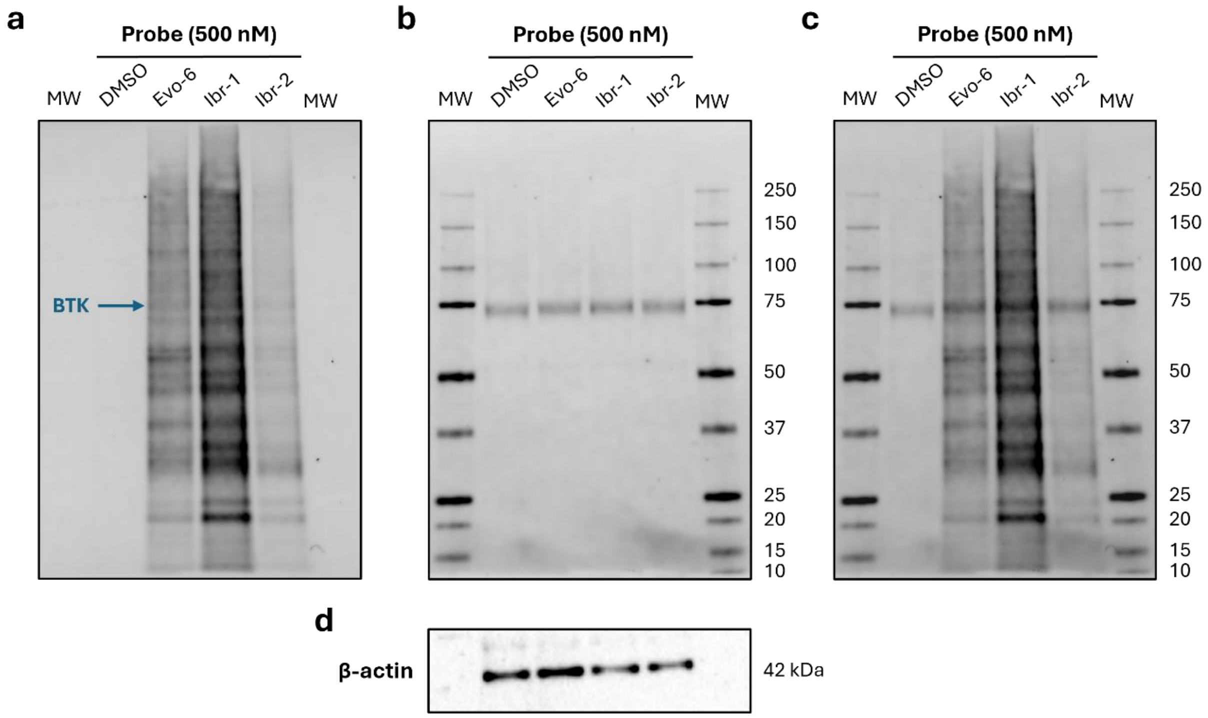

Figure S18. Evo-6, **Ibr-1** and **Ibr-2** binds to cellular BTK as shown by fluorescent Western blot. Ramos cells were treated with 500 nM probe for 1 h and modified with TAMRA-N_3. Proteins were separated by SDS-PAGE and transferred to a nitrocellulose membrane for immunoblotting. (a) Fluorescent signal on membrane corresponding to TAMRA-labeled proteins imaged with ChemiDoc imaging system (green LED, 605/50 filter). The blue arrow indicates the BTK band. (b) Immunoblotting of BTK with mouse anti-BTK primary antibody followed by goat anti-mouse Alexa633 secondary antibody. Fluorescent signal on membrane corresponding to BTK imaged with ChemiDoc imaging system (red LED, 695/50 filter). (c) Merged picture of the two channels confirm binding of the probes to BTK. The molecular weights (MWs) of the fluorescent markers are shown to the right of the gel. (d) Membrane was stripped and re-probed against β-actin using mouse anti-β-actin HRP-conjugated antibody and chemiluminescence detection.

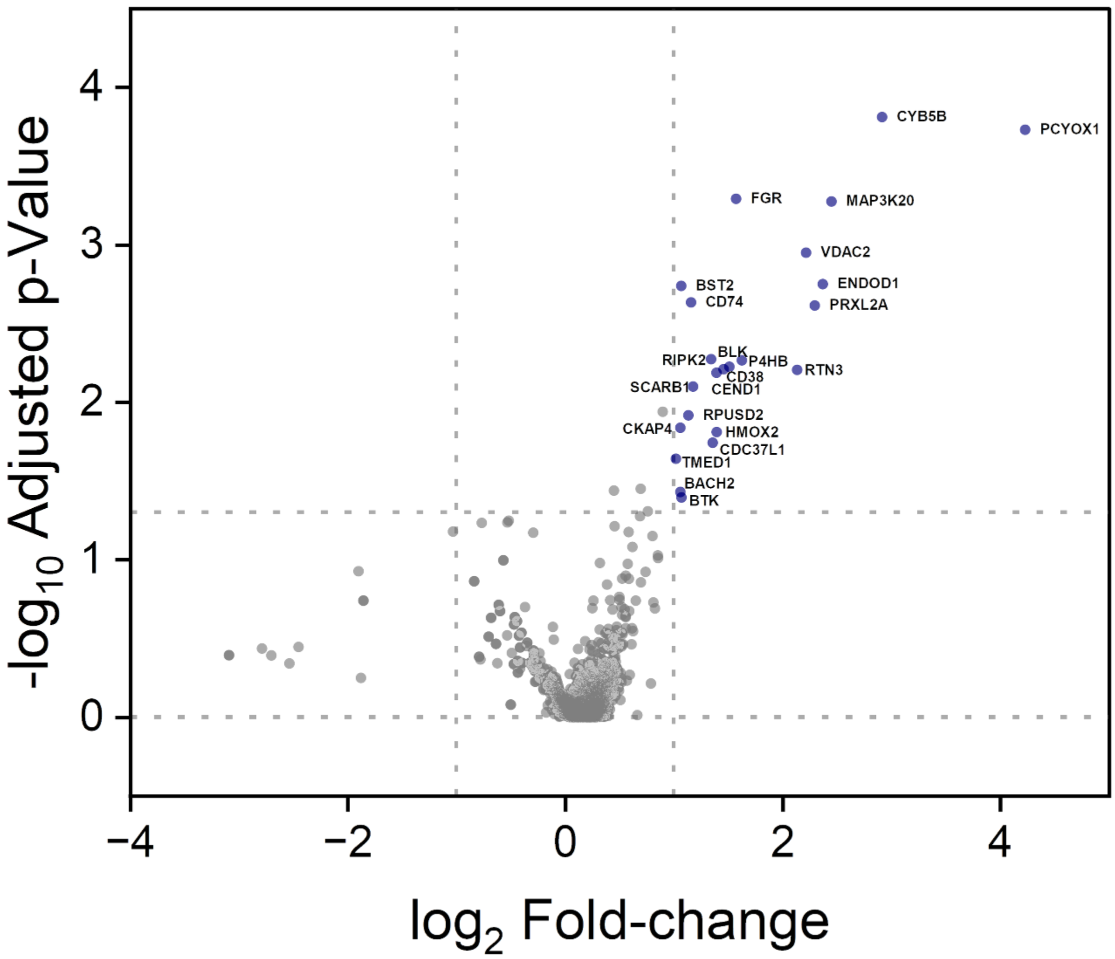

Figure S19. Enlarged version of the volcano plot presented in Figure 6c with annotation of all significantly enriched proteins. Ramos cells were treated with 250 nM **Ibr-2** or DMSO, followed by conjugation to biotin-N_3 and pull-down. Proteins with a $\log_2$ fold-change > 1 compared to the DMSO control and an adjusted p-value < 0.05 were considered significantly enriched (highlighted in blue and labeled).

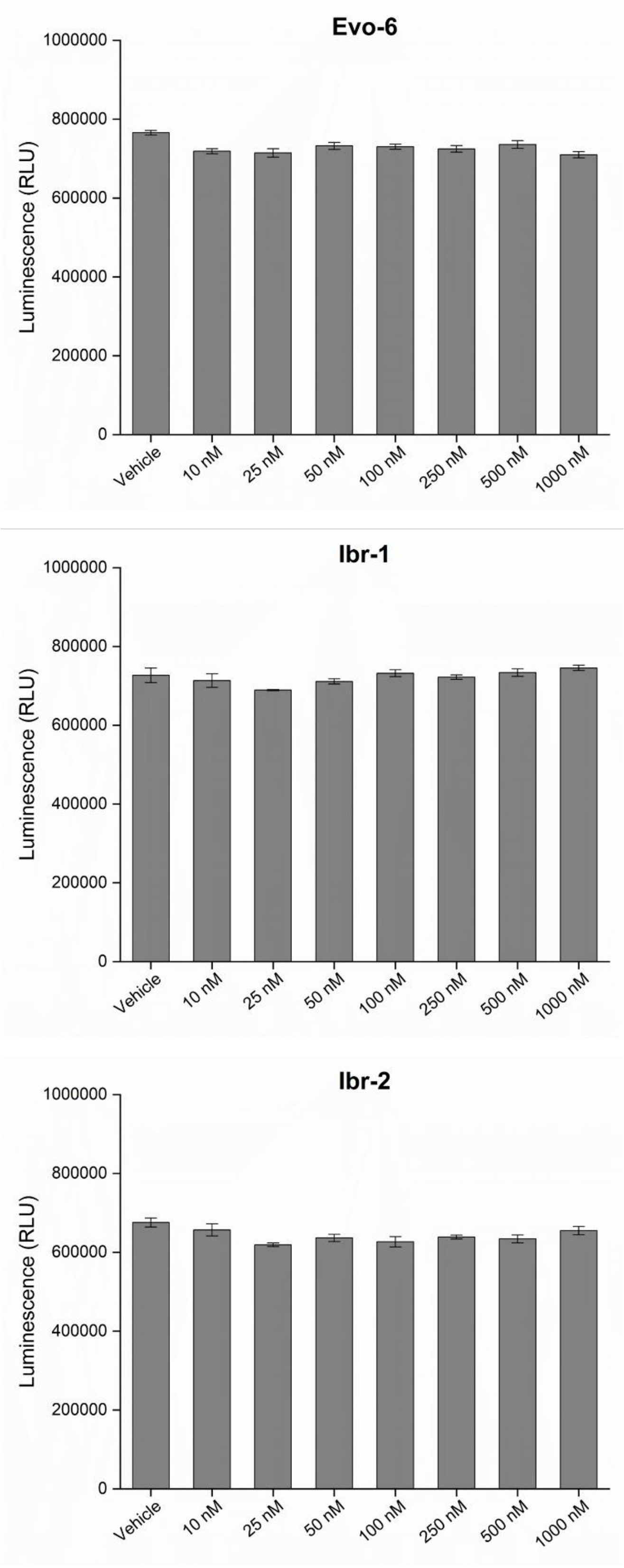

Figure S20. Evo-6, **Ibr-1** and **Ibr-2** showed no significant effect on cell viability up to 1 μM. Ramos cell suspension was seeded in a 96-well plate and treated with DMSO or 10-1000 nM probe for 1 h at 37°C. Next, the CellTiter-Glo assay was used to determine the viability. Luminescence intensity (RLU) correlates with the amount of ATP in the cell lysate which correlates with the number of metabolically active cells. Data are presented as mean ± SD (n = 4).

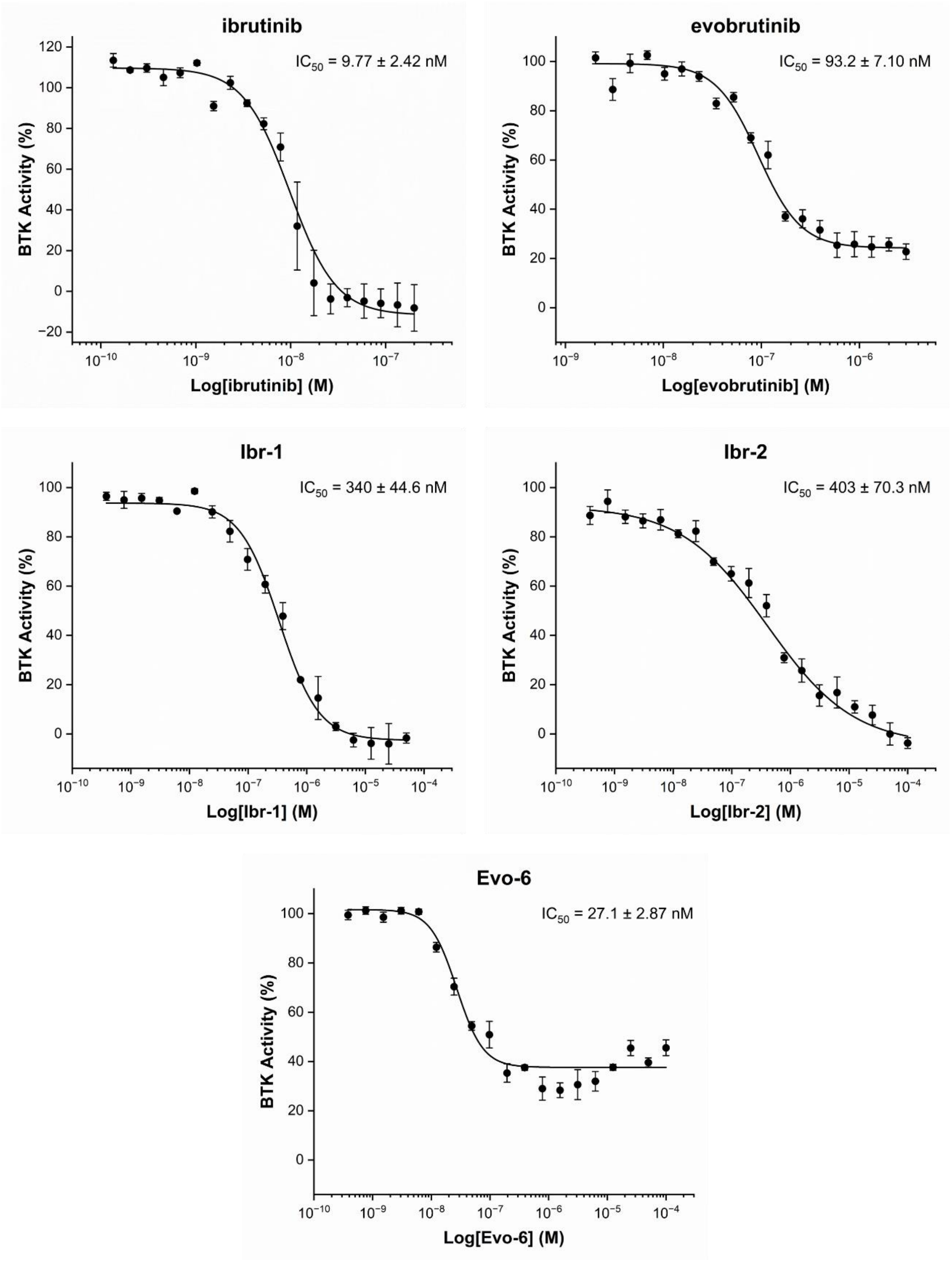

Figure S21. IC_{50} values of ibrutinib, evobrutinib, **Ibr-1**, **Ibr-2**, and **Evo-6** against full-length recombinant BTK. An ADP-Glo Kinase Assay was performed with 10 ng BTK, 50 µM ATP, 0.2 µg/µL Poly(Glu_4Tyr_1), test compounds (0-100 µM), and 1% DMSO. Data are presented as the mean ± SD (*n* = 4).

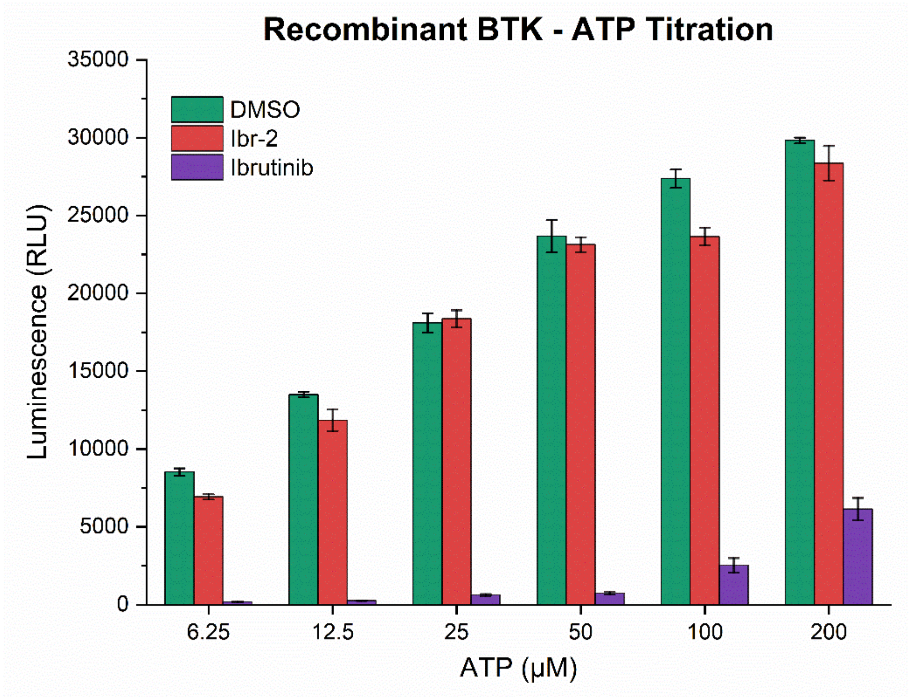

Figure S22. Ibr-2 does not interfere with the ATP-binding in recombinant BTK. An ADP-Glo Kinase Assay was performed where BTK was treated with DMSO (green), **Ibr-2** (red), or ibrutinib (purple) under increasing concentrations of ATP. Assay conditions: 10 ng BTK, 6.25-200 μM ATP, 0.2 μg/μL Poly(Glu_4Tyr_1), 500 nM test compound, and 1% DMSO. Luminescence is presented as relative luminescence units (RLU). Data are presented as the mean ± SD (n = 4).

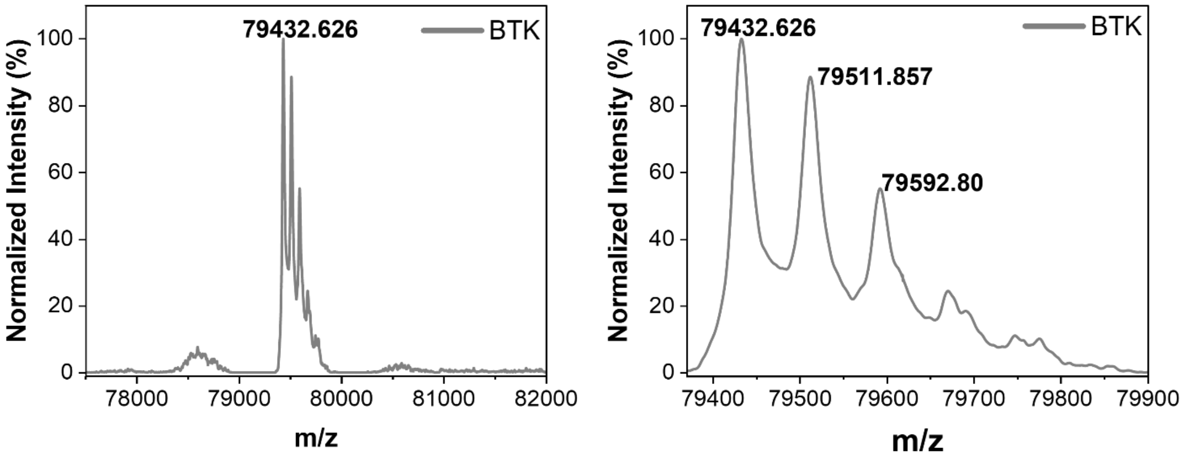

Figure S23. Intact protein mass spectrometry analysis of full-length recombinant BTK. Deconvoluted MS spectrum of BTK (grey, m/z = 79432.626 Da) and its enlarged version with the mono-and di-phosphorylated peaks annotated.

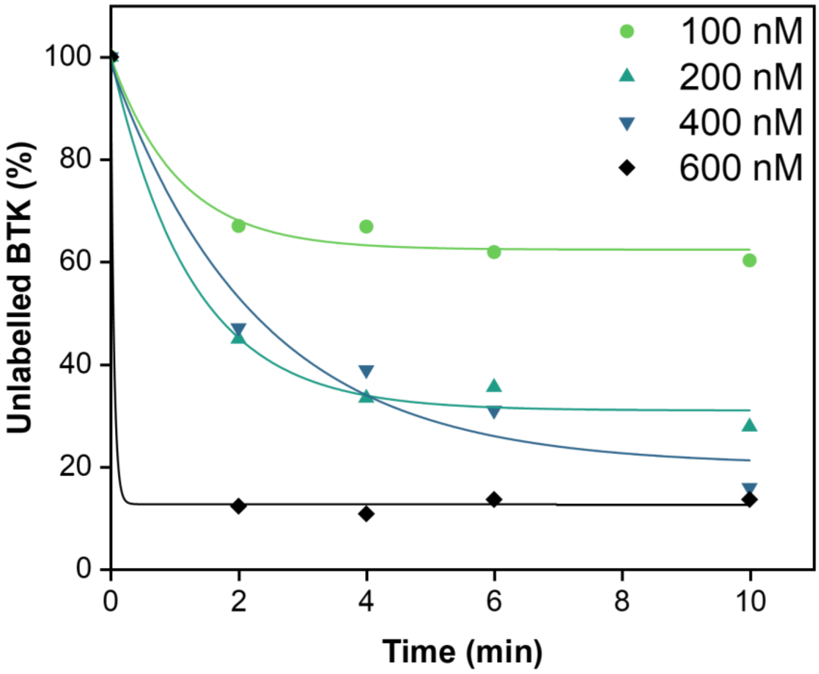

Figure S24. Time-course labeling experiment of recombinant BTK with different concentrations of **Ibr-2**. Depletion of unlabeled BTK (200 nM) when incubated with **Ibr-2** (100, 200, 400, and 600 nM) in 50 mM ABC buffer pH 8, 22 °C, over time. Following protein denaturation, alkylation with iodoacetamide, and trypsin digestion overnight, samples were analyzed by LC-MS and quantification was performed by measuring the relative peak areas of the unlabeled and **Ibr-2**-labeled Cys-481 containing peptides.

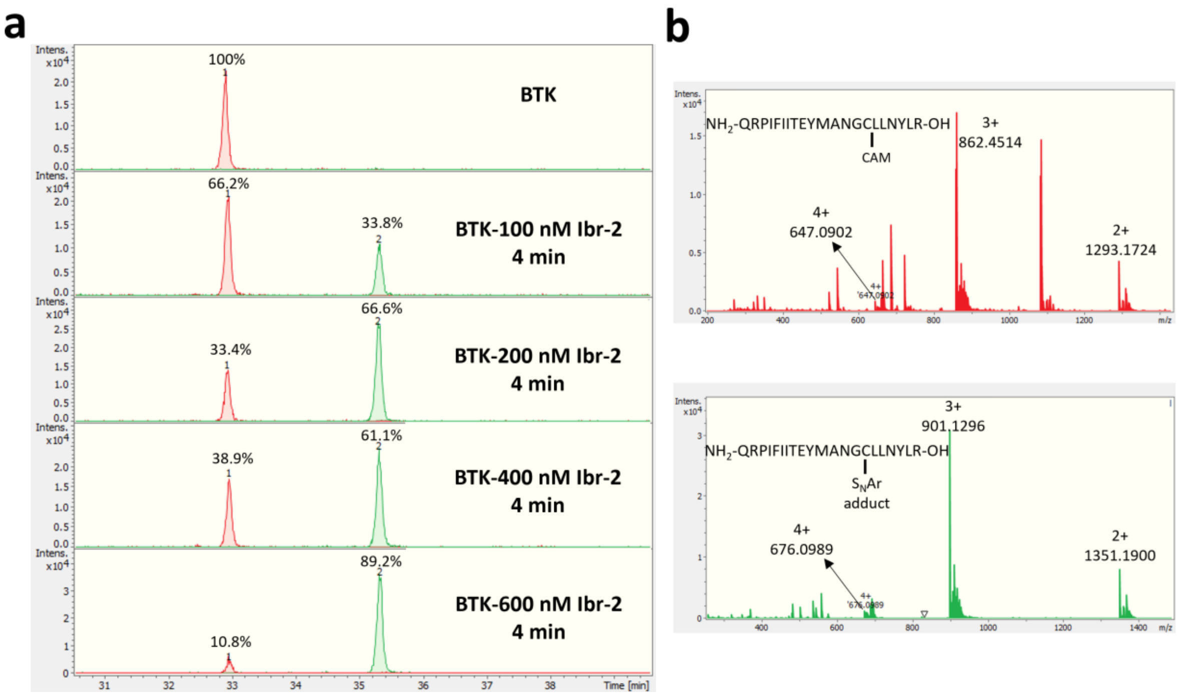

Figure S25. Ibr-2 demonstrated concentration-dependent BTK labeling. a) Extracted ion chromatogram (EIC) of labeled peptides at different concentrations of probe **Ibr-2** after 4 min of incubation time. The carbamidomethylated (CAM) peptide is shown in red and the **Ibr-2**-modified peptide in green. b) The mass spectrum of the CAM-modified Q_{467}-R_{487} peptide is shown in red and the mass spectrum of the **Ibr-2**-modified Q_{467}-R_{487} peptide in green. The relevant *m/z* values ($[M+2H]^{2+}$, $[M+3H]^{3+}$, and $[M+4H]^{4+}$) for the corresponding peptides are annotated in the spectrum.

2 Supplementary Methods

2.1 Molecular Modeling

Molecular Docking

The crystal structures of BTK in complex with evobrutinib (PDB ID: 6OMU), ibrutinib (PDB ID: 5P9J), and a non-covalent ibrutinib analogue (PDB ID: 5P9I) were prepared (Schrödinger) using the Protein Preparation Wizard from Schrödinger.s23 The ligands were imported to Maestro as SDF files prepared with the LigPrep utility function set to default settings. A Glide grid was generated with 22 Å dimensions set to the centroid of Cys481, without any restraints or restricted volumes. The prepared ligand structures were docked against the generated Glide grid with the following modification made to the default settings: Precision = XP; write out at most 2 poses per ligand. The docking results were exported as PDB structures and were analyzed in Molecular Operating Environment (MOE, version 2024.0601).

Molecular Dynamics Simulations

All molecular dynamics simulations were conducted using Desmond within the Schrödinger Suite.[1] Five systems were simulated including, BTK in complex with evobrutinib (PDB ID: 6OMU), BTK in complex with a non-covalent ibrutinib analogue (PDB ID: 5P9I), and the docked structures of BTK with **Evo-6**, **Ibr-1** and **Ibr-2**. The systems were prepared using the Desmond system builder,[2] solvating each system in a 20 Å buffered orthorhombic solvent box of SPC water molecules.[3] Net positive charges were neutralized by Cl^- ions, and 150 mM of KCl was added. Both compounds and the proteins were parametrized using the OPLS4 force field.[4] After energy minimization to alleviate steric clashes, the systems underwent gradual heating to 300 K under harmonic restraints imposed on backbone atoms. Following this, the restraints were gradually released, and an NPT ensemble was sampled for 100 ns with 1.2 ps integration time steps. Employing the Nose-Hoover thermostat and Martyna–Tobias–Klein barostat with isotropic coupling, the temperature and pressure of the system were kept constant at 300 K and 1.01325 bar atmospheric pressure, respectively.[5,6] Using the simulation interaction diagram (SID) panel as implemented in Schrödinger,[1] the simulation trajectories were analyzed for root-mean-square deviations (RMSD), protein-ligand interactions and root-mean-square fluctuations (RMSF) of both proteins and ligands. The obtained trajectories were clustered according to RMSD using the “Desmond Trajectory Clustering” module, setting up a frequency value of 10 (every 10th ns) and up to a maximum of 2 clusters. Regarding the MD simulations of co-crystallized complexes, the obtained cluster resembling the crystallographic pose was used as a representative structure, while with respect to the MD simulations of docked complexes, the most populated cluster was used as a representative structure.

Hydrogen-Bond Analysis

Hydrogen-bond interactions were quantified using Maestro’s *analyze_hbonds_trajectory.py* script, which processes entire trajectory files to identify donor-acceptor pairs across simulation frames. For each system, detailed temporal maps of hydrogen bonds were generated, and their occupancy percentages calculated to distinguish persistent from transient interactions. These occupancy statistics facilitated comparative analysis across **Evo-6**, **Ibr-1**, and **Ibr-2** systems.

Specifically, conserved hydrogen bonds were determined by high occupancy values, whereas low occupancy corresponded to transient or context-dependent interactions.

2.2 Pull-Down Proteomics Analysis

LC-MS3 Analysis

The fractions (5 µL) were analyzed on an Orbitrap Eclipse Tribrid mass spectrometer equipped with a FAIMS Pro ion mobility system and interfaced with an Easy-nLC1200 liquid chromatography system (all Thermo Fisher Scientific). Peptides were trapped on an Acclaim Pepmap 100 C18 trap column (100 µm x 2 cm, particle size 5 µm, Thermo Fisher Scientific) and separated on an in-house packed analytical column (35 cm x 75 µm, particle size 3 µm, Reprosil-Pur C18, Dr. Maisch) using a stepped gradient from 5% to 35% acetonitrile in 0.2% formic acid over 77 min at a flow of 300 nL/min. FAIMS Pro was alternating between the compensation voltages (CV) of -50 and -70, and the same data-dependent settings were used for all CVs. The precursor ion mass spectra were acquired at a resolution of 120,000 and an m/z range of 375-1375. Using a cycle time of 1 seconds the most abundant precursors with charges 2–7 were isolated with an *m/z* window of 0.7 and fragmented by collision induced dissociation (CID) at 35%. Fragment spectra were recorded in the ion trap at Rapid scan rate. Dynamic exclusion was set to 60 s. The ten most abundant MS2 fragment ions were isolated using multi-notch isolation for further MS3 fragmentation. MS3 fragmentation was performed using higher-energy collision dissociation (HCD) at 55% and the MS3 spectra were recorded in the Orbitrap at 50,000 resolution and an *m/z* range of 100–500.

Proteomic Data Analysis

Raw files were processed and analyzed with Proteome Discoverer (ver 3.0, Thermo Scientific). The data was matched against SwissProt *Human* (Jan 2025, 20,421 entries) together with a contaminant and streptavidin database (ThermoFisher, 248 entries) with Sequest as the search engine. Precursor and fragment ion tolerance were set to 10 ppm and 0.6 Da, and tryptic peptides were accepted with 1 missed cleavage. Methionine oxidation was set as a variable modification and cysteine carbamidomethylation, TMTpro on lysine and peptide N-termini were set as fixed modifications. Inferys rescoring was applied. Percolator was used for PSM validation with a strict FDR threshold of 1%. For quantification, TMT reporter ions were identified in the MS3 HCD spectra with 3 mmu mass tolerance. No normalization was applied. The SPS threshold was set to 65%, a Sequest HT threshold score of 2 was chosen. Only unique peptides were used for relative quantification and proteins were required to pass a protein FDR of 5%.

3 Chemical Synthesis of Starting Materials and Intermediates

3.1 General Procedures

General Procedure 1 (GP1): Preparation of Aryl Methyl Esters

A round-bottom flask was charged with the respective carboxylic acid (1.0 eq.) and purged with N_2. Then dry DCM and oxalyl chloride (1.5 eq.) were added followed by 3 drops of catalytic dry DMF. Upon addition of DMF, there was gas formation, and the solution was stirred at room temperature until LC-MS analysis indicated full conversion to the acid chloride (quenching of an aliquot with MeOH to detect the methyl ester). The reaction mixture was then cooled to 0°C and a pre-mixed solution of MeOH (20 eq.) and Et_3N (5.0 eq.) was added dropwise. White fumes of gaseous HCl formed and the reaction mixture was stirred at room temperature until LC-MS indicated full consumption of the acid chloride. Solvent was then evaporated under reduced pressure, and the residue was diluted in EtOAc and washed with water, saturated aqueous NH_4Cl, and saturated aqueous Na_2CO_3. The organic layer was dried over Na_2SO_4 and concentrated under reduced pressure. The crude was then charged on silica and purified by column chromatography to afford the respective aryl methyl esters.

General Procedure 2 (GP2): Thiolation of Aryl Halides for the Preparation of Aryl Thioethers

A microwave vial was charged with the respective aryl halide (1.0 eq.) and Cs_2CO_3 (1.2 eq.). The vial was capped and purged with N_2. Then DMF and the thiol (1.05-1.1 eq.) were added, and the reaction mixture was stirred at room temperature until TLC and LC-MS analysis indicated complete reaction. The reaction mixture was poured into water and extracted twice with EtOAc. The combined organic layers were washed twice with saturated aqueous Na_2CO_3, washed once with brine, dried over Na_2SO_4, and concentrated under reduced pressure. The crude was then charged on silica and purified by column chromatography to afford the respective aryl thioethers. In some cases, the crude product was of high purity and no column purification was required. *Note: In some cases, the residual thiol co-eluted with the desired product and is present in the aliphatic region of the NMR spectra. This impurity was successfully removed in the next step.*

3.2 Synthesis and Characterization of Starting Materials

***tert*-butyl 4-fluoro-3-(trifluoromethyl)benzoate (1a)**

An oven-dried 100 ml round-bottom flask was charged with 4-fluoro-3-(trifluoromethyl)benzoic acid (1.51 g, 7.24 mmol, 1.0 eq.) and purged with N_2. Then, *tert*-butyl 2,2,2-trichloroacetimidate (1.94 ml, 10.9 mmol, 1.5 eq.) and Et_2O (15 ml) were added, followed by dropwise addition of BF_3-OEt_2 (91 μL, 0.72 mmol, 10 mol%). The colorless solution was stirred at room temperature. The reaction was monitored by LC-MS and if required additional *tert*-butyl 2,2,2-trichloroacetimidate and BF_3-OEt_2 were added to consume all starting material. After 40 h, solid $NaHCO_3$ (500 mg) was added, and the reaction mixture was concentrated under reduced pressure. The residue was purified by column chromatography (silica 25 g, 0-20% EtOAc:pentane) to afford **1a** (eluted at 15% EtOAc) as a clear oil (1.54 g, 80%). Rf = 0.89 (EtOAc:pentane 1:9). ^1H NMR (600 MHz, $CDCl_3$) δ 8.24 (dd, J = 7.0, 2.3 Hz, 1H), 8.17 (ddd, J = 8.6, 4.8, 2.2 Hz, 1H), 7.22 (t, J = 9.3 Hz, 1H), 1.59 (s, 9H); ^{13}C NMR (151 MHz, $CDCl_3$) δ 163.6, 162.3 (d, $^1J_{CF}$ = 263 Hz), 135.5 (d, $^3J_{CF}$ = 9.8 Hz), 129.1, 128.7, 122.3 (q, $^1J_{CF}$ = 272 Hz), 118.6 (qd, $^2J_{CF}$ = 33.5, 13.0 Hz), 117.0 (d, $^2J_{CF}$ = 21 Hz), 82.4, 28.2;

^{19}F NMR (564 MHz, $CDCl_3$) δ -61.75 (d, J = 12.7 Hz), -108.56 (ddt, J = 22.9, 12.0, 6.6 Hz); LC-MS (ESI) *m/z*: compound did not ionize.

methyl 4-fluoro-3-nitrobenzoate (1b-Me)

Following GP1 using 4-fluoro-3-nitrobenzoic acid (1.01 g, 5.46 mmol, 1.0 eq.), ester **1b-Me** was obtained as a yellow solid (800 mg, 74%) after purification by column chromatography (silica 25 g, 0-15% EtOAc:pentane) with the product eluting at 15% EtOAc. ^1H NMR (600 MHz, $CDCl_3$) δ 8.74 (dd, J = 7.2, 2.1 Hz, 1H), 8.32 (ddd, J = 8.7, 4.2, 2.2 Hz, 1H), 7.42-7.35 (m, 1H), 3.98 (s, 3H); ^{13}C NMR (151 MHz, $CDCl_3$) δ 164.2, 158.2 (d, $^1J_{CF}$ = 272 Hz), 137.5, 136.7 (d, $^3J_{CF}$ = 9.8 Hz), 128.0, 127.4 (d, $^4J_{CF}$ = 4.1 Hz), 118.9 (d, $^2J_{CF}$ = 21 Hz), 53.1; ^{19}F NMR (564 MHz, $CDCl_3$) δ -110.50 (dddd, J = 10.2, 7.2, 4.2, 0.8 Hz); LC-MS (ESI) *m/z*: compound did not ionize.

***tert*-butyl 4-fluoro-3-nitrobenzoate (1b-tBu)**

An oven-dried 100 ml round-bottom flask was charged with 4-fluoro-3-nitrobenzoic acid (1.00 g, 5.40 mmol, 1.0 eq.) and purged with N_2. Then, *tert*-butyl 2,2,2-trichloroacetimidate (1.16 ml, 6.48 mmol, 1.2 eq.) and Et_2O (12 ml) were added, followed by dropwise addition of BF_3-OEt_2 (34 μL, 0.27 mmol, 5 mol%). The colorless solution was stirred at room temperature. The reaction was monitored by LC-MS and if required additional *tert*-butyl 2,2,2-trichloroacetimidate and BF_3-OEt_2 were added to consume all starting material. After 30 h, solid $NaHCO_3$ (300 mg) was added, and the reaction mixture was concentrated under reduced pressure. The residue was purified by column chromatography (silica 25 g, 0-20% EtOAc:pentane) to afford **1b-tBu** (eluted at 15% EtOAc) as a white crystalline solid (1.12 g, 86%). Rf = 0.88 (EtOAc:pentane 1:4). ^1H NMR (600 MHz, $CDCl_3$) δ 8.64 (dd, J = 7.2, 2.2 Hz, 1H), 8.26 (ddd, J = 8.7, 4.3, 2.2 Hz, 1H), 7.34 (dd, J = 10.2, 8.7 Hz, 1H), 1.61 (s, 9H); ^{13}C NMR (151 MHz, $CDCl_3$) δ 162.8, 157.9 (d, $^1J_{CF}$ = 271 Hz), 137.4, 136.5 (d, $^3J_{CF}$ = 9.8 Hz), 129.2 d, $^4J_{CF}$ = 2.9 Hz), 127.7, 118.6 (d, $^2J_{CF}$ = 21 Hz), 83.1, 28.2; ^{19}F NMR (564 MHz, $CDCl_3$) δ -111.70 (ddd, J = 10.3, 7.2, 4.3 Hz); LC-MS (ESI) *m/z*: compound did not ionize.

***tert*-butyl 4-((3-cyano-4-fluorophenyl)sulfonyl)piperazine-1-carboxylate (1c)**

In a round-bottom flask, 4-fluoro-3-cyanobenzenesulfonyl chloride (240 mg, 1.09 mmol, 1.0 eq.) was dissolved in DCM (2 ml) and cooled to 0°C. Then a solution of 1-Boc-piperazine (204 mg, 1.09 mmol, 1.0 eq.) and Et_3N (0.46 ml, 3.28 mmol, 3.0 eq.) in DCM (2 mL) was added in one portion. The reaction mixture was stirred at room temperature until TLC showed full consumption of the starting material. After 20 min, the solution was diluted with DCM and washed twice with saturated aqueous NH_4Cl. The organic layer was dried over Na_2SO_4 and concentrated under reduced pressure to afford **1c** as a white solid (230 mg, 57%), that was used in the next step without further purification. ^1H NMR (600 MHz, $CDCl_3$) δ 8.04 (dd, J = 5.8, 2.3 Hz, 1H), 7.98 (ddd, J = 8.8, 4.7, 2.3 Hz, 1H), 7.42 (t, J = 8.5 Hz, 1H), 3.53 (t, J = 5.1 Hz, 4H), 3.00 (t, J = 5.0 Hz, 4H), 1.41 (s, 9H); ^{13}C NMR (151 MHz, $CDCl_3$) δ 165.4 (d, $^1J_{CF}$ = 268 Hz), 154.2, 134.5 (d, $^3J_{CF}$ = 9.5 Hz), 133.9, 133.6, 117.9 (d, $^2J_{CF}$ = 21 Hz), 112.3, 103.3 (d, $^3J_{CF}$ = 16 Hz), 80.9, 46.0, 28.4; ^{19}F NMR (564 MHz, $CDCl_3$) δ -98.29 (ddd, J = 8.2, 5.8, 4.8 Hz); LC-MS (ESI) *m/z*: $[M+H-Boc]^+$ calcd. for $C_{11}H_{13}FN_3O_2S$: 270.07, found: 270.10 (fragmentation of Boc group).

2-fluoro-5-(pyrrolidin-1-ylsulfonyl)benzonitrile (1d)

In a round-bottom flask, 4-fluoro-3-cyanobenzenesulfonyl chloride (314 mg, 1.43 mmol, 1.0 eq.) was dissolved in DCM (2 ml) and cooled to -20°C. Then a solution of pyrrolidine (0.12 ml, 1.43 mmol, 1.0 eq.) and Et_3N (0.40 ml, 2.86 mmol, 2.0 eq.) in DCM (2 mL) was added in one portion. The reaction mixture was stirred at room temperature until TLC showed full consumption of the starting material. After 15 min, the solution was diluted with DCM and washed with water followed by 1 M HCl and brine. The organic layer was dried over Na_2SO_4 and concentrated under reduced pressure. The crude was purified by column chromatography (silica 10 g, 0-100% DCM:pentane) to afford **1d** (eluted at 70% DCM) as a beige crystalline solid (113 mg, 31%). ^{1}H NMR (600 MHz, $CDCl_3$) δ 8.09 (dd, J = 5.9, 2.3 Hz, 1H), 8.05 (ddd, J = 8.8, 4.9, 2.3 Hz, 1H), 7.40 (t, J = 8.6 Hz, 1H), 3.25-3.20 (m, 4H), 1.83-1.77 (m, 4H); ^{13}C NMR (151 MHz, $CDCl_3$) δ 165.0 (d, $^1J_{CF}$ = 266 Hz), 135.1 (d, $^4J_{CF}$ = 3.8 Hz), 134.2 (d, $^3J_{CF}$ = 9.6 Hz), 133.1, 117.7 (d, $^2J_{CF}$ = 21 Hz), 112.5, 102.7 (d, $^3J_{CF}$ = 16 Hz), 48.1, 25.3; ^{19}F NMR (564 MHz, $CDCl_3$) δ -99.76 (ddd, J = 8.4, 5.9, 4.8 Hz); LC-MS (ESI) *m/z*: $[M+H]^+$ calcd. for $C_{11}H_{12}FN_2O_2S$: 255.06, found: 255.10.

6-((2-(*tert*-butoxy)-2-oxoethyl)thio)nicotinic acid (9)

In a 25 ml round-bottom flask, *tert*-butyl bromoacetate (0.21 ml, 1.44 mmol, 1.1 eq.) and Cs_2CO_3 (561 mg, 1.72 mmol, 1.3 eq.) were suspended in dry DMF (2 ml). The flask was purged with N_2 and cooled to 0°C. Then, 6-mercaptopyridine-3-carboxylic acid (200 mg, 1.29 mmol, 1.0 eq.) in dry DMF (2 ml) was added dropwise and the mixture was stirred at 0°C until HPLC showed full consumption of the starting material. After 30 min, the reaction mixture was poured into ice-water (20 ml) and washed with EtOAc (20 ml). The aqueous layer was concentrated and dried under reduced pressure to afford **9** as a yellow solid. The crude was used directly in the next step. *Note: Compound **9** is soluble in the aqueous layer upon extraction, which is concentrated and used in the sequential esterification. Crude product contains residual salts. Washing with EtOAc was to remove a dialkylation side product where the carboxylic acid was alkylated.*

***tert*-butyl 6-chloro-5-(trifluoromethyl)nicotinate (1f)**

A 50 ml round-bottom flask was charged with 6-chloro-5-(trifluoromethyl)nicotinic acid (401 mg, 1.78 mmol, 1.0 eq.) and DMAP (43 mg, 0.36 mmol, 0.2 eq.), and purged with N_2. Then Boc_2O (0.82 ml, 3.55 mmol, 2.0 eq.) and anhydrous *t*-BuOH (8 ml) were added, and the yellow solution was heated at 40°C until LC-MS showed full consumption of the starting material. After 21 h, the reaction mixture was diluted with water (15 ml) and extracted with EtOAc (3x15 ml). The combined organic layers were washed with NH_4Cl (2x25 ml) and concentrated under reduced pressure. The crude was purified by reverse-phase column chromatography (C18 silica 30 g, 40-100% MeCN:water with 0.1% TFA) to afford **1f** (eluted at 60% MeCN) as a clear oil (115 mg, 23%). Rf = 0.37 (MeCN:water 4:1 with 0.1% TFA, reverse-phase TLC). ^{1}H NMR (600 MHz, $CDCl_3$) δ 9.06 (d, J = 2.1 Hz, 1H), 8.51 (d, J = 1.9 Hz, 1H), 1.61 (s, 9H); ^{13}C NMR (151 MHz, $CDCl_3$) δ 162.4, 153.3, 152.5, 137.6 (q, $^3J_{CF}$ = 5.1 Hz), 126.8, 125.3 (q, $^2J_{CF}$ = 34 Hz), 121.9 (q, $^1J_{CF}$ = 273 Hz), 83.7, 28.2. ^{19}F NMR (564 MHz, $CDCl_3$) δ -63.84; LC-MS (ESI) *m/z*: $[M+H]^+$ calcd. for $C_{11}H_{12}ClF_3NO_2$: 282.05, found: 282.04.

***tert*-butyl 6-chloro-5-nitronicotinate (1g)**

An oven-dried 2-necked 100 ml round-bottom flask equipped with a condenser was evacuated and refilled with N_2 three times. The flask was charged with 6-hydroxy-5-nitronicotinic acid (1.00 g, 5.43 mmol, 1.0 eq.), PCl_5 (2.26 g, 10.9 mmol, 2.0 eq.) and $POCl_3$ (2.53 ml, 27.2 mmol, 5.0 eq.).

The reaction mixture was heated at 115°C for 2 h. After this time, the red solution was cooled to 0°C and a pre-mixed solution of anhydrous *t*-BuOH (5.19 ml, 54.3 mmol, 10 eq.) and pyridine (4.37 ml, 54.3 mmol, 10 eq.) was added slowly. White fumes of gaseous HCl formed and the black solution was allowed to warm to room temperature and was stirred until LC-MS indicated full consumption of the intermediate acid chloride. After 20 h, the reaction mixture was cooled to 0°C and diluted with water (100 ml) and EtOAc (100 ml). The black suspension was filtered to remove solids and the layers were separated. The organic layer was dried over Na_2SO_4 and concentrated under reduced pressure. The crude was purified by column chromatography (silica 25 g, 0-15% EtOAc:pentane) to afford **1g** (eluted at 11% EtOAc) as an off-white crystalline solid (342 mg, 24%). Rf = 0.40 (EtOAc:pentane 1:19). ^{1}H NMR (600 MHz, $CDCl_3$) δ 9.10 (d, *J* = 2.1 Hz, 1H), 8.66 (d, *J* = 2.0 Hz, 1H), 1.62 (s, 9H); ^{13}C NMR (151 MHz, $CDCl_3$) δ 161.5, 153.0, 146.7, 144.6, 134.9, 127.9, 84.3, 28.2; LC-MS (ESI) *m/z*: $[M+H]^+$ calcd. for $C_{10}H_{12}ClN_2O_4$: 259.05, found: 259.06.

2-mercaptopyrimidine-5-carbaldehyde (10)

Compound **10** was synthesized according to a literature procedure by Maltsev, O. V. et al.[7] in three steps from bromoacetic acid (Scheme S1).

***tert*-butyl 2-((5-formylpyrimidin-2-yl)thio)acetate (11)**

In a 50 ml round-bottom flask, *tert*-butyl bromoacetate (0.38 ml, 2.55 mmol, 1.1 eq.) and Cs_2CO_3 (907 mg, 2.78 mmol, 1.2 eq.) were suspended in dry DMF (4 ml) and the flask was purged with N_2. Then, **10** (325 mg, 2.31 mmol, 1.0 eq.) in dry DMF (4 ml) was added dropwise and the orange suspension was stirred at room temperature until LC-MS showed full consumption of the starting material. After 20 min, the reaction mixture was poured into ice-water (50 ml) and extracted with EtOAc (3x40 ml). The combined organic layers were concentrated under reduced pressure and residual DMF was removed by freeze-drying to afford **11** as an orange solid (589 mg, 100%). ^{1}H NMR (600 MHz, $CDCl_3$) δ 10.02 (s, 1H), 8.91 (s, 2H), 3.92 (s, 2H), 1.47 (s, 9H); ^{13}C NMR (151 MHz, $CDCl_3$) δ 188.4, 177.3, 167.5, 158.3, 125.1, 82.6, 35.0, 28.1; LC-MS (ESI) *m/z*: $[M+H\text{-}tBu]^+$ calcd. for $C_7H_7N_2O_3S$: 199.02, found: 199.05 (fragmentation of *t*-Bu group).

2-((2-(*tert*-butoxy)-2-oxoethyl)thio)pyrimidine-5-carboxylic acid (11a)

In a 100 ml round-bottom flask, **11** (1.36 g, 5.35 mmol, 1.0 eq.) was dissolved in MeCN (6 ml). Then $NaH_2PO_4 \cdot 2\,H_2O$ (0.92 g, 5.89 mmol, 1.1 eq.) was added, followed by 50% H_2O_2 solution (0.46 ml, 8.03 mmol, 1.5 eq.). The yellow suspension was cooled to 0°C, followed by slow addition of $NaClO_2$ (0.67 g, 5.89 mmol, 1.1 eq.) as a solution in water (6 ml). The yellow solution was stirred at 0°C until LC-MS showed full consumption of the starting material. After 20 min, the reaction was quenched by addition of saturated aqueous $Na_2S_2O_3$ (6 ml). The MeCN was removed under reduced pressure and the aqueous residue was acidified to pH 1 using 1 M HCl. Upon acidification, a white precipitate formed. The solids were filtered, washed with 1 M HCl followed by pentane, and dried under vacuum to afford **11a** as an off-white solid (1.29 g, 89%). ^{1}H NMR (600 MHz, $(CD_3)_2SO$) δ 8.99 (s, 2H), 3.99 (s, 2H), 1.40 (s, 9H); ^{13}C NMR (151 MHz, $(CD_3)_2SO$) δ 174.3, 167.4, 164.8, 158.1, 120.5, 81.3, 34.1, 27.6; LC-MS (ESI) *m/z*: $[M-H]^-$ calcd. for $C_{11}H_{13}N_2O_4S$: 269.06, found: 269.07.

2-(methylthio)pyrimidine-4-carbaldehyde (1i-1)

Compound **1i-1** was synthesized according to a literature procedure by Divakaran, A. et al.[8] in three steps from 1,1-dimethoxyacetone (Scheme S3).

2-(methylsulfonyl)pyrimidine-4-carboxylic acid (1i-2)

In a 100 ml round-bottom flask, **1i-1** (584 mg, 3.79 mmol, 1.0 eq.) was suspended in MeCN (10 ml). Then $NaH_2PO_4 \cdot 2\ H_2O$ (2.95 g, 18.9 mmol, 5.0 eq.) was added, followed by 50% H_2O_2 solution (2.15 ml, 37.9 mmol, 10 eq.). The yellow suspension was cooled to 0°C, followed by slow addition of $NaClO_2$ (2.14 g, 18.9 mmol, 5.0 eq.) as a solution in water (10 ml). The reaction mixture was warmed to room temperature and stirred until LC-MS indicated complete reaction. After 22 h, the reaction was quenched by addition of solid $Na_2S_2O_3$. The solvent was removed under reduced pressure to yield a crude solid. The solids were transferred to a filter funnel and washed three times with acetone to dissolve the product but not salts from the reaction. The filtrate was concentrated and dried under vacuum to afford **1i-2** as a white solid (492 mg, 64%). ^{1}H NMR (600 MHz, $(CD_3)_2SO$) δ 9.29 (d, *J* = 5.0 Hz, 1H), 8.23 (d, *J* = 5.0 Hz, 1H), 3.44 (s, 3H); LC-MS (ESI) *m/z*: $[M-H]^-$ calcd. for $C_6H_5N_2O_4S$: 201.00, found: 201.09. Characterization data was in agreement with literature.[9]

methyl 2-(methylsulfonyl)pyrimidine-4-carboxylate (1i)

A 50 ml round-bottom flask was charged with **1i-2** (195 mg, 0.97 mmol, 1.0 eq.) and purged with N_2. Dry DCM (5 ml) and MeOH (1 ml) were added, and the mixture was cooled to 0°C. Then (trimethylsilyl)diazomethane (2.0 M solution in hexanes, 0.97 ml, 1.93 mmol, 2.0 eq.) was added dropwise. The white suspension was stirred at 0°C until TLC showed full consumption of the starting material. After 40 min, the reaction mixture was quenched by addition of AcOH (0.2 ml) and concentrated under reduced pressure. The crude was purified by column chromatography (silica 10 g, 50-100% EtOAc:pentane) to afford **1i** (eluted at 78% EtOAc) as a white solid (48 mg, 23%). Rf = 0.54 (EtOAc). ^{1}H NMR (600 MHz, $CDCl_3$) δ 9.18 (d, *J* = 4.9 Hz, 1H), 8.20 (d, *J* = 4.9 Hz, 1H), 4.03 (s, 3H), 3.40 (s, 3H); ^{13}C NMR (151 MHz, $CDCl_3$) δ 166.7, 163.2, 161.1, 156.6, 123.4, 54.0, 39.4; LC-MS (ESI) *m/z*: $[M+H]^+$ calcd. for $C_7H_9N_2O_4S$: 217.03, found: 217.06.

methyl (*E*)-3-(2-((2-(*tert*-butoxy)-2-oxoethyl)thio)pyrimidin-5-yl)acrylate (11b)

An oven-dried 50 ml round-bottom flask was charged with **11** (471 mg, 1.85 mmol, 1.0 eq.). The flask was evacuated and refilled with N_2 three times. Then dry DCM (6 ml) followed by methyl (triphenylphosphoranylidene)acetate (1.24 mg, 3.71 mmol, 2.0 eq.) as a solution in dry DCM (6 ml) were added, and the orange solution was stirred at room temperature until LC-MS indicated complete reaction. After 3 h, the reaction mixture was concentrated under reduced pressure and the crude was purified by column chromatography (silica 25 g, 0-50% EtOAc:pentane) to afford **11b** (eluted at 30% EtOAc) as a white solid (489 mg, 85%). Rf = 0.25 (EtOAc:pentane 1:4). ^{1}H NMR (600 MHz, $CDCl_3$) δ 8.63 (s, 2H), 7.54 (d, *J* = 16.2 Hz, 1H), 6.49 (d, *J* = 16.1 Hz, 1H), 3.88 (s, 2H), 3.82 (s, 3H), 1.46 (s, 9H); ^{13}C NMR (151 MHz, $CDCl_3$) δ 172.6, 167.9, 166.5, 156.0, 137.6, 123.9, 120.1, 82.3, 52.2, 34.9, 28.1; LC-MS (ESI) *m/z*: $[M+Na]^+$ calcd. for $C_{14}H_{18}N_2NaO_4S$: 333.09, found: 333.18.

methyl 6-chloropyridazine-3-carboxylate (1k)

Following GP1 using 6-chloropyridazine-3-carboxylic acid (502 mg, 3.17 mmol, 1.0 eq.), ester **1k** was obtained as a white solid (358 mg, 66%). The crude did not require further purification. ^{1}H

NMR (600 MHz, $CDCl_3$) δ 8.17 (d, *J* = 8.8 Hz, 1H), 7.68 (d, *J* = 8.8 Hz, 1H), 4.09 (s, 3H); ^{13}C NMR (151 MHz, $CDCl_3$) δ 164.0, 159.5, 150.8, 129.8, 128.9, 53.7; LC-MS (ESI) *m/z*: $[M+H]^+$ calcd. for $C_6H_6ClN_2O_2$: 173.01, found: 173.04.

methyl 5-chloropyrazine-2-carboxylate (1l-Me)

Following GP1 using 5-chloropyrazine-3-carboxylic acid (498 mg, 3.14 mmol, 1.0 eq.), ester **1l-Me** was obtained as a white solid (343 mg, 63%) after purification by column chromatography (silica 10 g, 0-35% EtOAc:pentane) with the product eluting at 30% EtOAc. Rf = 0.40 (EtOAc:pentane 1:4). ^{1}H NMR (600 MHz, $CDCl_3$) δ 9.05 (d, *J* = 1.4 Hz, 1H), 8.67 (d, *J* = 1.4 Hz, 1H), 4.01 (s, 3H); ^{13}C NMR (151 MHz, $CDCl_3$) δ 163.7, 152.8, 145.8, 144.5, 141.3, 53.4; LC-MS (ESI) *m/z*: $[M+H]^+$ calcd. for $C_6H_6ClN_2O_2$: 173.01, found: 173.04.

***tert*-butyl 5-chloropyrazine-2-carboxylate (1l-tBu)**

In an oven-dried 100 ml round-bottom flask, 5-chloropyrazine-3-carboxylic acid (1.02 g, 6.41 mmol, 1.0 eq.) in THF (9 ml) was treated with a solution of *tert*-butyl 2,2,2-trichloroacetimidate (2.30 ml, 12.8 mmol, 2.0 eq.) in cyclohexane (5 ml) under N_2 atmosphere. The reaction was stirred at room temperature for 5 min followed by addition of BF_3-OEt_2 (0.81 ml, 6.41 mmol, 1.0 eq.). The orange solution was stirred at room temperature until LC-MS indicated complete reaction. After 4 h, the reaction mixture was diluted with EtOAc (10 ml), washed with saturated aqueous $NaHCO_3$ (15 ml) and water (15 ml), dried over Na_2SO_4, and concentrated under reduced pressure. The crude was purified by column chromatography (silica 25 g, 0-10% EtOAc:pentane) to afford **1l-tBu** (eluted at 10% EtOAc) as a clear oil (1.17 g, 85%). ^{1}H NMR (600 MHz, $CDCl_3$) δ 8.99 (d, *J* = 1.2 Hz, 1H), 8.67 (d, *J* = 1.3 Hz, 1H), 1.64 (s, 9H); ^{13}C NMR (151 MHz, $CDCl_3$) δ 162.1, 152.0, 145.4, 144.3, 142.9, 83.7, 28.1. LC-MS (ESI) *m/z*: $[M+H\text{-}tBu]^+$ calcd. for $C_5H_4ClN_2O_2$: 159.00, found: 159.06 (fragmentation of *t*-Bu group).

2-cyano-3-(1*H*-indol-3-yl)prop-2-enethioamide (12a)

Compound **12a** was synthesized according to a literature procedure by Dyachenko, V. D. et al.[10] in one step from indole-3-carboxaldehyde and 2-cyanothioacetamide (Scheme S2).

ethyl 5-cyano-6-mercapto-2-methylnicotinate (12)

Compound **12** was synthesized according to a literature procedure by Dyachenko, V. D. et al.[11] in two steps from **12a** (Scheme S2).

***tert*-butyl 4-((4-chloro-2-(trifluoromethyl)phenyl)sulfonyl)piperazine-1-carboxylate (1o)**

In a round-bottom flask, 4-chloro-2-(trifluoromethyl)benzenesulfonyl chloride (161 mg, 0.58 mmol, 1.0 eq.) was dissolved in DCM (2 ml) and cooled to 0°C. Then a solution of 1-Boc-piperazine (108 mg, 0.58 mmol, 1.0 eq.) and Et_3N (0.24 ml, 1.73 mmol, 3.0 eq.) in DCM (2 mL) was added in one portion. The reaction mixture was stirred at room temperature until TLC showed full consumption of the starting material. After 1 h, the solution was diluted with DCM and washed with water followed by 1 M HCl and brine. The organic layer was dried over Na_2SO_4 and concentrated under reduced pressure to afford **1o** as a white solid (70 mg, 28%), that was used in the next step without further purification. ^{1}H NMR (600 MHz, $CDCl_3$) δ 8.02 (d, *J* = 2.1 Hz, 1H), 7.97 (d, *J* = 8.5 Hz, 1H), 7.84 (dd, *J* = 8.5, 2.1 Hz, 1H), 3.49 (t, *J* = 5.1 Hz, 4H), 3.20 (t, *J* = 5.1 Hz, 4H), 1.43 (s, 9H); ^{13}C NMR (201 MHz, $CDCl_3$) δ 154.3, 136.4, 135.4, 133.7, 131.9 (q, $^3J_{CF}$ = 7 Hz), 129.6

(q, $^2J_{CF}$ = 34 Hz), 127.9, 121.6 (q, $^1J_{CF}$ = 275 Hz), 80.6, 45.6, 44.1, 42.8, 28.4; ^{19}F NMR (564 MHz, $CDCl_3$) δ -57.74; LC-MS (ESI) *m/z*: $[M+H\text{-}tBu]^+$ calcd. for $C_{12}H_{13}ClF_3N_2O_4S$: 373.02, found: 373.07 (fragmentation of *t*-Bu group).

***tert*-butyl 4-((chloromethyl)thio)-3-nitrobenzoate (17)**

In a 100 ml round-bottom flask, sodium hydrosulfide hydrate (300 mg, 4.05 mmol, 2.0 eq.) was dissolved in water (10 ml) and cooled to 0°C. A solution of **1b-tBu** (488 mg, 2.02 mmol, 1.0 eq.) in acetone (10 ml) was added dropwise over 10 min and the dark-red suspension was stirred at 0°C until LC-MS indicated full conversion to the intermediate thiol. After 30 min, chloroiodomethane (0.44 ml, 6.07 mmol, 3.0 eq.) was added dropwise at 0°C and the reaction mixture was left to stir at room temperature until LC-MS indicated complete reaction. The color lightened from black to yellow over time. After 18 h, the acetone was removed under reduced pressure. The aqueous residue was diluted with water (25 ml) and extracted with EtOAc (3x25 ml). The combined organic layers were washed with brine (50 ml), dried over Na_2SO_4, and concentrated under reduced pressure. The crude was purified by column chromatography (silica 25 g, 0-15% EtOAc:pentane) to afford **17** (eluted at 10% EtOAc) as a yellow oil (370 mg, 60%). Rf = 0.49 (EtOAc:pentane 1:9). ^{1}H NMR (800 MHz, $CDCl_3$) δ 8.79 (d, *J* = 1.9 Hz, 1H), 8.24 (dd, *J* = 8.5, 1.8 Hz, 1H), 7.77 (d, *J* = 8.4 Hz, 1H), 5.02 (s, 2H), 1.61 (s, 9H); ^{13}C NMR (201 MHz, $CDCl_3$) δ 163.3, 145.6, 139.0, 134.2, 130.3, 127.2, 126.4, 82.8, 46.1, 28.2; GC-MS (EI) *m/z*: $[M]^+$ calcd. for $C_{12}H_{14}ClNO_4S$: 303.03, found: 303.0.

***tert*-butyl 3-nitro-4-(prop-2-yn-1-ylthio)benzoate (19)**

In a 100 ml round-bottom flask, sodium hydrosulfide hydrate (462 mg, 6.24 mmol, 2.0 eq.) was dissolved in water (10 ml) and cooled to 0°C. A solution of **1b-tBu** (752 mg, 3.12 mmol, 1.0 eq.) in acetone (10 ml) was added dropwise over 10 min and the dark-red suspension was stirred at 0°C until LC-MS indicated full conversion to the intermediate thiol. After 30 min, 80% propargyl bromide in toluene (0.70 ml, 6.24 mmol, 2.0 eq.) was added dropwise at 0°C and the reaction mixture was left to stir at room temperature until LC-MS indicated complete reaction. The color lightened from black to yellow over time. After 1 h, the acetone was removed under reduced pressure. The aqueous residue was diluted with water (25 ml) and extracted with EtOAc (3x25 ml). The combined organic layers were washed with brine (50 ml), dried over Na_2SO_4, and concentrated under reduced pressure. The crude was purified by column chromatography (silica 25 g, 0-15% EtOAc:pentane) to afford **19** (eluted at 10% EtOAc) as a yellow solid (889 mg, 97%). Rf = 0.35 (EtOAc:pentane 1:9). ^{1}H NMR (600 MHz, $CDCl_3$) δ 8.77 (d, *J* = 1.7 Hz, 1H), 8.16 (dd, *J* = 8.5, 1.8 Hz, 1H), 7.61 (d, *J* = 8.6 Hz, 1H), 3.72 (d, *J* = 2.4 Hz, 2H), 2.27 (t, *J* = 2.5 Hz, 1H), 1.59 (s, 9H); ^{13}C NMR (151 MHz, $CDCl_3$) δ 163.5, 145.2, 141.3, 133.8, 129.3, 127.1, 126.4, 82.5, 77.9, 72.8, 28.2, 21.1; GC-MS (EI) *m/z*: $[M]^+$ calcd. for $C_{14}H_{15}NO_4S$: 293.07, found: 293.1.

(9*H*-fluoren-9-yl)methyl carbonazidate (Fmoc-N_3)

Fmoc-N_3 was synthesized from Fmoc-Cl according to a literature procedure by Meyer, A. R. et al.[12] Characterization data was in agreement with the reference.

3.3 Synthesis and Characterization of Aryl Thioethers (2a-2o, 3a-3o, 18, 20)

***tert*-butyl 4-((2-methoxy-2-oxoethyl)thio)-3-(trifluoromethyl)benzoate (2a)**

Following GP2, aryl halide **1a** (313 mg, 1.18 mmol, 1.0 eq.), Cs_2CO_3 (463 mg, 1.42 mmol, 1.2 eq.), and methyl thioglycolate (0.12 ml, 1.30 mmol, 1.1 eq.) were stirred for 3 h to afford the title compound (279 mg, 67%) as a clear oil. The crude was purified by column chromatography (silica 10 g, 0-20% EtOAc:pentane) with the product eluting at 15% EtOAc. Rf = 0.35 (EtOAc:pentane 1:9). ^{1}H NMR (600 MHz, $CDCl_3$) δ 8.24 (d, *J* = 1.9 Hz, 1H), 8.07 (dd, *J* = 8.3, 1.9 Hz, 1H), 7.55 (d, *J* = 8.3 Hz, 1H), 3.76 (s, 2H), 3.74 (s, 3H), 1.59 (s, 9H); ^{13}C NMR (151 MHz, $CDCl_3$) δ 169.2, 164.2, 140.5, 132.9, 130.1, 129.5, 129.1 (q, $^2J_{CF}$ = 31 Hz), 128.0 (q, $^3J_{CF}$ = 6 Hz), 123.4 (q, $^1J_{CF}$ = 274 Hz), 82.2, 53.0, 35.8, 28.3; ^{19}F NMR (564 MHz, $CDCl_3$) δ -61.32; LC-MS (ESI) *m/z*: compound did not ionize.

***tert*-butyl 4-((2-methoxy-2-oxoethyl)thio)-3-nitrobenzoate (2b)**

Following GP2, aryl halide **1b-tBu** (340 mg, 1.41 mmol, 1.0 eq.), Cs_2CO_3 (551 mg, 1.69 mmol, 1.2 eq.), and methyl thioglycolate (0.14 ml, 1.55 mmol, 1.1 eq.) were stirred for 1 h to afford the title compound (432 mg, 94%) as a yellow crystalline solid. The crude did not require further purification. Rf = 0.40 (EtOAc:pentane 1:4). ^{1}H NMR (600 MHz, $CDCl_3$) δ 8.76 (d, *J* = 1.9 Hz, 1H), 8.13 (dd, *J* = 8.5, 1.9 Hz, 1H), 7.51 (d, *J* = 8.5 Hz, 1H), 3.80 (s, 2H), 3.76 (s, 3H), 1.59 (s, 9H); ^{13}C NMR (151 MHz, $CDCl_3$) δ 168.9, 163.4, 145.4, 141.3, 134.0, 129.5, 127.2, 126.3, 82.6, 53.1, 34.8, 28.2; LC-MS (ESI) *m/z*: compound did not ionize.

methyl 6-((2-(*tert*-butoxy)-2-oxoethyl)thio)nicotinate (2e)

A 50 ml round-bottom flask was charged with crude **9** (347 mg, 1.29 mmol, 1.0 eq.) and EDC HCl (296 mg, 1.55 mmol, 1.2 eq.) and purged with N_2. Then, dry DCM (5 ml), MeOH (0.26 ml, 6.44 mmol, 5.0 eq.), and DMAP (31 mg, 0.26 mmol, 0.2 eq.) as a solution in dry DCM (1 ml) were added. The yellow mixture was stirred at room temperature until LC-MS showed full consumption of the starting material. After 16 h, the solvent was removed under reduced pressure. The residue was dissolved in EtOAc (25 ml) and washed with saturated aqueous NH_4Cl (3x25 ml) followed by water (25 ml). The organic layer was concentrated under reduced pressure and the crude was purified by column chromatography (silica 10 g, 0-50% EtOAc:pentane) to afford **2e** (eluted at 30% EtOAc) as a yellow oil (206 mg, 56% over 2 steps). Rf = 0.89 (EtOAc). ^{1}H NMR (700 MHz, $CDCl_3$) δ 8.92 (d, *J* = 2.2 Hz, 1H), 8.00 (dd, *J* = 8.4, 2.2 Hz, 1H), 7.23 (d, *J* = 8.4 Hz, 1H), 3.87 (s, 2H), 3.86 (s, 3H), 1.41 (s, 9H); ^{13}C NMR (176 MHz, $CDCl_3$) δ 168.1, 165.8, 163.1, 150.5, 136.4, 122.0, 121.1, 82.0, 52.2, 33.6, 28.0; LC-MS (ESI) *m/z*: $[M+H\text{-}t\text{Bu}]^+$ calcd. for $C_9H_{10}NO_4S$: 228.03, found: 228.11 (fragmentation of *t*-Bu group).

***tert*-butyl 6-((2-methoxy-2-oxoethyl)thio)-5-(trifluoromethyl)nicotinate (2f)**

Following GP2, aryl halide **1f** (65 mg, 0.23 mmol, 1.0 eq.), Cs_2CO_3 (90 mg, 0.28 mmol, 1.2 eq.), and methyl thioglycolate (23 µL, 0.25 mmol, 1.1 eq.) were stirred for 45 min to afford the title compound (63 mg, 78%) as a clear oil. The crude was purified by column chromatography (silica 5 g, 0-15% EtOAc:pentane) with the product eluting at 12% EtOAc. Rf = 0.54 (EtOAc:pentane 1:9). ^{1}H NMR (600 MHz, $CDCl_3$) δ 9.02 (d, *J* = 2.0 Hz, 1H), 8.32 (d, *J* = 2.1 Hz, 1H), 4.03 (s, 2H), 3.74 (s, 3H), 1.59 (s, 9H); ^{13}C NMR (151 MHz, $CDCl_3$) δ 169.3, 163.3, 160.8, 152.2, 135.3 (q, $^3J_{CF}$ = 5 Hz), 123.4, 123.2 (q, $^2J_{CF}$ = 34 Hz), 123.0 (q, $^1J_{CF}$ = 273 Hz), 82.8, 52.9, 32.6, 28.2; ^{19}F NMR (564 MHz, $CDCl_3$) δ -63.28; LC-MS (ESI) *m/z*: $[M+H]^+$ calcd. for $C_{14}H_{17}F_3NO_4S$: 352.08, found: 352.15.

***tert*-butyl 6-((2-methoxy-2-oxoethyl)thio)-5-nitronicotinate (2g)**

Following GP2, aryl halide **1g** (101 mg, 0.39 mmol, 1.0 eq.), Cs_2CO_3 (153 mg, 0.47 mmol, 1.2 eq.), and methyl thioglycolate (38 µL, 0.43 mmol, 1.1 eq.) were stirred for 45 min to afford the title compound (118 mg, 92%) as a black crystalline solid. The crude did not require further purification. Rf = 0.52 (EtOAc:pentane 1:4). ^{1}H NMR (600 MHz, $CDCl_3$) δ 9.13 (d, *J* = 1.9 Hz, 1H), 8.96 (d, *J* = 2.0 Hz, 1H), 4.00 (s, 2H), 3.74 (s, 3H), 1.61 (s, 9H); ^{13}C NMR (151 MHz, $CDCl_3$) δ 169.3, 162.5, 160.3, 153.4, 141.4, 134.5, 124.2, 83.5, 52.9, 33.6, 28.2; LC-MS (ESI) *m/z*: $[M+H]^+$ calcd. for $C_{13}H_{17}N_2O_6S$: 329.08, found: 329.13.

methyl 2-((2-(*tert*-butoxy)-2-oxoethyl)thio)pyrimidine-5-carboxylate (2h)

Following GP1 using **11a** (290 mg, 1.07 mmol, 1.0 eq.), ester **2h** was obtained as a white crystalline solid (216 mg, 71%) after purification by column chromatography (silica 10 g, 0-50% EtOAc:pentane) with the product eluting at 30% EtOAc. ^{1}H NMR (600 MHz, $CDCl_3$) δ 8.94 (s, 2H), 3.88 (s, 3H), 3.83 (s, 2H), 1.40 (s, 9H); ^{13}C NMR (151 MHz, $CDCl_3$) δ 175.7, 167.6, 164.2, 158.0, 119.5, 82.2, 52.5, 34.8, 27.9; LC-MS (ESI) *m/z*: $[M+H\text{-}tBu]^+$ calcd. for $C_8H_9N_2O_4S$: 229.03, found: 229.09 (fragmentation of *t*-Bu group).

methyl 2-((2-methoxy-2-oxoethyl)thio)pyrimidine-4-carboxylate (2i)

Following GP2, aryl methanesulfonyl **1i** (47 mg, 0.22 mmol, 1.0 eq.), Cs_2CO_3 (84 mg, 0.26 mmol, 1.2 eq.), and methyl thioglycolate (21 µL, 0.24 mmol, 1.1 eq.) were stirred for 75 min to afford the title compound (29 mg, 55%) as a clear oil. The crude was purified by column chromatography (high-capacity silica 5 g, 10-70% EtOAc:pentane) with the product eluting at 40% EtOAc. ^{1}H NMR (600 MHz, $CDCl_3$) δ 8.72 (d, *J* = 4.9 Hz, 1H), 7.63 (d, *J* = 4.9 Hz, 1H), 3.99-3.96 (m, 5H), 3.75 (s, 3H); ^{13}C NMR (151 MHz, $CDCl_3$) δ 171.9, 169.5, 164.3, 159.4, 155.3, 116.3, 53.4, 52.8, 33.5; LC-MS (ESI) *m/z*: $[M+H]^+$ calcd. for $C_9H_{11}N_2O_4S$: 243.04, found: 243.08.

methyl 3-(2-((2-(*tert*-butoxy)-2-oxoethyl)thio)pyrimidin-5-yl)propanoate (2j)

In a 5 ml microwave vial, **11b** (288 mg, 0.93 mmol, 1.0 eq.) was dissolved in MeOH (4 ml) and purged with N_2. The vial was cooled to 0°C and $CoCl_2 \cdot 6\,H_2O$ (55 mg, 0. 23 mmol, 0.25 eq.) was added. Then $NaBH_4$ (141 mg, 3.71 mmol, 4.0 eq.) was added in portions of 1 eq. each hour and the brown reaction mixture was stirred at 0°C, until TLC indicated full consumption of the starting material. After 3.5 h, water was added, and the product was extracted three times with EtOAc. The combined organic layers were washed with brine, dried over $MgSO_4$, and concentrated under reduced pressure. The crude was purified by column chromatography (silica 25 g, 0-50% EtOAc:pentane) to afford **2j** (eluted at 20% EtOAc) as a white solid (130 mg, 45%). Rf = 0.58 (EtOAc:pentane 2:3). ^{1}H NMR (600 MHz, $CDCl_3$) δ 8.32 (s, 2H), 3.76 (s, 2H), 3.59 (s, 3H), 2.79 (t, *J* = 7.4 Hz, 2H), 2.55 (t, *J* = 7.4 Hz, 2H), 1.38 (s, 9H); ^{13}C NMR (151 MHz, $CDCl_3$) δ 172.2, 168.8, 168.2, 157.2, 128.8, 81.8, 51.8, 34.6, 34.5, 27.9, 24.8; LC-MS (ESI) *m/z*: $[M+Na]^+$ calcd. for $C_{14}H_{20}N_2NaO_4S$: 335.10, found: 335.20.

methyl 6-((2-methoxy-2-oxoethyl)thio)pyridazine-3-carboxylate (2k)

Following GP2, aryl halide **1k** (140 mg, 0.81 mmol, 1.0 eq.), Cs_2CO_3 (318 mg, 0.98 mmol, 1.2 eq.), and methyl thioglycolate (80 µL, 0.89 mmol, 1.1 eq.) were stirred for 1 h to afford the title compound (116 mg, 59%) as a white crystalline solid. The crude was purified by column chromatography (silica 10 g, 0-70% EtOAc:pentane) with the product eluting at 50% EtOAc. Rf = 0.30 (EtOAc:pentane 1:1). ^{1}H NMR (600 MHz, $CDCl_3$) δ 7.96 (d, *J* = 8.9 Hz, 1H), 7.51 (d, *J* = 8.9 Hz, 1H), 4.21 (s, 2H), 4.05 (s, 3H), 3.77 (s, 3H); ^{13}C NMR (151 MHz, $CDCl_3$) δ 169.0, 164.7, 163.7, 148.8,

126.6, 125.9, 53.4, 53.1, 32.5; LC-MS (ESI) *m/z*: $[M+H]^+$ calcd. for $C_9H_{11}N_2O_4S$: 243.04, found: 243.10.

methyl 5-((2-methoxy-2-oxoethyl)thio)pyrazine-2-carboxylate (2l)

Following GP2, aryl halide **1l-Me** (92 mg, 0.54 mmol, 1.0 eq.), Cs_2CO_3 (340 mg, 1.04 mmol, 1.95 eq.), and methyl thioglycolate (53 µL, 0.59 mmol, 1.1 eq.) were stirred for 45 min to afford the title compound (104 mg, 80%) as a yellow crystalline solid. The crude was purified by column chromatography (silica 5 g, 0-50% EtOAc:pentane) with the product eluting at 40% EtOAc. Rf = 0.11 (EtOAc:pentane 1:4). ^{1}H NMR (600 MHz, $CDCl_3$) δ 9.02 (d, *J* = 1.5 Hz, 1H), 8.57 (d, *J* = 1.4 Hz, 1H), 4.03 (s, 2H), 3.99 (s, 3H), 3.74 (s, 3H); ^{13}C NMR (151 MHz, $CDCl_3$) δ 169.0, 164.7, 159.7, 145.5, 142.7, 138.5, 53.1, 31.7; LC-MS (ESI) *m/z*: $[M+H]^+$ calcd. for $C_9H_{11}N_2O_4S$: 243.04, found: 243.11.

methyl 2-((2-methoxy-2-oxoethyl)thio)thiazole-5-carboxylate (2m)

Following GP2, methyl 2-bromothiazole-5-carboxylate (100 mg, 0.45 mmol, 1.0 eq.), Cs_2CO_3 (176 mg, 0.54 mmol, 1.2 eq.), and methyl thioglycolate (44 µl, 0.50 mmol, 1.1 eq.) were stirred for 2 h to afford the title compound (60 mg, 53%) as a white crystalline solid. The crude was purified by column chromatography (silica 5 g, 0-30% EtOAc:pentane) with the product eluting at 30% EtOAc. Rf = 0.47 (EtOAc:pentane 1:4). ^{1}H NMR (600 MHz, $CDCl_3$) δ 8.16 (s, 1H), 4.06 (s, 2H), 3.86 (s, 3H), 3.75 (s, 3H); ^{13}C NMR (151 MHz, $CDCl_3$) δ 170.1, 168.5, 161.3, 148.1, 128.8, 53.1, 52.6, 35.1; LC-MS (ESI) *m/z*: $[M+H]^+$ calcd. for $C_8H_{10}NO_4S_2$: 248.00, found: 248.02.

ethyl 6-((2-(tert-butoxy)-2-oxoethyl)thio)-5-cyano-2-methylnicotinate (2n)

In a 25 ml round-bottom flask, *tert*-butyl bromoacetate (40 µl, 0.27 mmol, 1.1 eq.) and K_2CO_3 (34 mg, 0.25 mmol, 1.0 eq.) were suspended in dry DMF (1.5 ml) and the flask was purged with N_2. Then, **12** (54 mg, 0.25 mmol, 1.0 eq.) in dry DMF (1.5 ml) was added dropwise and the yellow suspension was stirred at room temperature until LC-MS showed full consumption of the starting material. After 15 min, the reaction mixture was poured into ice-water (20 ml) and extracted with EtOAc (3x20 ml). The combined organic layers were concentrated under reduced pressure. The residue was purified by column chromatography (high-capacity silica 5 g, 0-20% EtOAc:pentane) to afford **2n** (eluted at 20% EtOAc) as a light-pink crystalline solid (44 mg, 54%). Rf = 0.72 (EtOAc:pentane 3:17). ^{1}H NMR (600 MHz, $CDCl_3$) δ 8.32 (s, 1H), 4.36 (q, *J* = 7.1 Hz, 2H), 3.94 (s, 2H), 2.83 (s, 3H), 1.45 (s, 9H), 1.39 (t, *J* = 7.1 Hz, 3H); ^{13}C NMR (151 MHz, $CDCl_3$) δ 167.3, 164.6, 163.8, 163.8, 143.0, 121.1, 114.8, 104.5, 82.5, 61.8, 33.9, 28.1, 25.6, 14.3; LC-MS (ESI) *m/z*: [M+H-*t*Bu]$^+$ calcd. *for $C_{12}H_{13}N_2O_4S$: 281.06, found: 281.05 (fragmentation of t-Bu group).*

***tert*-butyl 4-((4-((2-methoxy-2-oxoethyl)thio)-2-(trifluoromethyl)phenyl)sulfonyl) piperazine-1-carboxylate (2o)**

Following GP2, aryl halide **1o** (37 mg, 0.086 mmol, 1.0 eq.), Cs_2CO_3 (34 mg, 0.10 mmol, 1.2 eq.), and methyl thioglycolate (8.5 µL, 0.095 mmol, 1.1 eq.) were stirred for 2.5 h to afford the title compound (17 mg, 39%) as a clear oil. The crude was purified by column chromatography (high-capacity silica 5 g, 0-30% EtOAc:pentane) with the product eluting at 27% EtOAc. Rf = 0.18 (EtOAc:pentane 1:3). ^{1}H NMR (600 MHz, $CDCl_3$) δ 8.00 (d, *J* = 8.5 Hz, 1H), 7.77 (d, *J* = 2.1 Hz, 1H), 7.57 (dd, *J* = 8.5, 2.1 Hz, 1H), 3.80-3.76 (m, 5H), 3.51-3.46 (m, 4H), 3.21-3.16 (m, 4H), 1.44 (s, 9H); ^{13}C NMR (151 MHz, $CDCl_3$) δ 169.1, 154.4, 143.8, 134.0, 132.9, 129.2, 128.7 (q, $^2J_{CF}$ = 33 Hz), 126.6

(q, $^3J_{CF}$ = 6 Hz), 122.2 (q, $^1J_{CF}$ = 275 Hz), 80.6, 53.2, 45.6, 44.0, 42.8, 34.3, 28.5; ^{19}F NMR (564 MHz, $CDCl_3$) δ -57.72; LC-MS (ESI) *m/z*: $[M+Na]^+$ calcd. for $C_{19}H_{25}F_3N_2NaO_6S_2$: 521.10, found: 521.18.

***tert*-butyl 4-((2-((tert-butoxycarbonyl)amino)ethyl)thio)-3-(trifluoromethyl)benzoate (3a)**

Following GP2, aryl halide **1a** (311 mg, 1.77 mmol, 1.0 eq.), Cs_2CO_3 (460 mg, 1.41 mmol, 1.2 eq.), and 2-(Boc-amino)ethanethiol (0.21 ml, 1.24 mmol, 1.05 eq.) were stirred for 1 h to afford the title compound (311 mg, 63%) as a clear oil. The crude was purified by column chromatography (silica 10 g, 0-40% DCM:pentane) with the product eluting at 40% DCM. ^{1}H NMR (600 MHz, $CDCl_3$) δ 8.22 (d, *J* = 1.9 Hz, 1H), 8.06 (dd, *J* = 8.3, 1.9 Hz, 1H), 7.56 (d, *J* = 8.3 Hz, 1H), 4.89 (t, *J* = 6.4 Hz, 1H), 3.38 (q, *J* = 6.4 Hz, 2H), 3.18 (t, *J* = 6.5 Hz, 2H), 1.59 (s, 9H), 1.43 (s, 9H); ^{13}C NMR (151 MHz, $CDCl_3$) δ 164.2, 155.8, 141.6, 132.7, 129.1, 128.6 (q, $^2J_{CF}$ = 31 Hz), 128.4, 127.8 (q, $^3J_{CF}$ = 6 Hz), 123.4 (q, $^1J_{CF}$ = 274 Hz), 81.9, 79.6, 39.5, 33.2, 28.3, 28.1; ^{19}F NMR (564 MHz, $CDCl_3$) δ -61.63; LC-MS (ESI) *m/z*: $[M+H-Boc]^+$ calcd. for $C_{14}H_{19}F_3NO_2S$: 322.11, found: 322.22 (fragmentation of Boc group).

methyl 4-((2-((tert-butoxycarbonyl)amino)ethyl)thio)-3-nitrobenzoate (3b)

Following GP2, aryl halide **1b-Me** (203 mg, 1.02 mmol, 1.0 eq.), Cs_2CO_3 (399 mg, 1.22 mmol, 1.2 eq.), and 2-(Boc-amino)ethanethiol (0.19 ml, 1.12 mmol, 1.1 eq.) were stirred for 2 h to afford the title compound (351 mg, 97%) as a yellow solid. The crude did not require further purification. ^{1}H NMR (600 MHz, $CDCl_3$) δ 8.84 (d, *J* = 1.9 Hz, 1H), 8.20 (dd, *J* = 8.5, 1.9 Hz, 1H), 7.66 (d, *J* = 8.5 Hz, 1H), 4.95 (t, *J* = 6.1 Hz, 1H), 3.96 (s, 3H), 3.46 (q, *J* = 6.7 Hz, 2H), 3.19 (t, *J* = 6.9 Hz, 2H), 1.45 (s, 9H); ^{13}C NMR (151 MHz, $CDCl_3$) δ 165.0, 155.9, 145.8, 142.8, 133.9, 127.5, 127.0, 126.6, 80.1, 52.8, 39.0, 32.4, 28.5; LC-MS (ESI) *m/z*: $[M+Na]^+$ calcd. for $C_{15}H_{20}N_2NaO_6S$: 379.09, found: 379.16.

***tert*-butyl 4-((4-((2-((tert-butoxycarbonyl)amino)ethyl)thio)-3-cyanophenyl)sulfonyl) piperazine-1-carboxylate (3c)**

Following GP2, aryl halide **1c** (115 mg, 0.31 mmol, 1.0 eq.), Cs_2CO_3 (122 mg, 0.37 mmol, 1.2 eq.), and 2-(Boc-amino)ethanethiol (58 µl, 0.34 mmol, 1.1 eq.) were stirred for 2 h to afford the title compound (83 mg, 51%) as a white solid. The crude was purified by column chromatography (silica 5 g, 0-60% EtOAc:pentane) with the product eluting at 60% EtOAc. ^{1}H NMR (600 MHz, $CDCl_3$) δ 7.92 (d, *J* = 2.0 Hz, 1H), 7.84 (dd, *J* = 8.5, 2.0 Hz, 1H), 7.71 (d, *J* = 8.5 Hz, 1H), 4.99 (s, 1H), 3.52 (t, *J* = 5.0 Hz, 4H), 3.41 (q, *J* = 6.7 Hz, 2H), 3.25 (t, *J* = 7.0 Hz, 2H), 2.99 (t, *J* = 5.0 Hz, 4H), 1.44 (s, 9H), 1.41 (s, 9H); ^{13}C NMR (151 MHz, $CDCl_3$) δ 156.0, 154.2, 148.8, 133.0, 132.7, 131.7, 126.6, 115.4, 112.2, 80.7, 80.2, 46.0, 43.5, 42.5, 39.6, 31.9, 28.5, 28.4.

***tert*-butyl (2-((2-cyano-4-(pyrrolidin-1-ylsulfonyl)phenyl)thio)ethyl)carbamate (3d)**

Following GP2, aryl halide **1d** (26 mg, 0.10 mmol, 1.0 eq.), Cs_2CO_3 (40 mg, 0.12 mmol, 1.2 eq.), and 2-(Boc-amino)ethanethiol (18 µl, 0.11 mmol, 1.05 eq.) were stirred for 1 h to afford the title compound (29 mg, 69%) as a white solid. The crude was purified by column chromatography (silica 5 g, 0-65% EtOAc:pentane) with the product eluting at 65% EtOAc. ^{1}H NMR (600 MHz, $CDCl_3$) δ 8.01 (d, *J* = 2.0 Hz, 1H), 7.93 (dd, *J* = 8.5, 2.0 Hz, 1H), 7.67 (d, *J* = 8.5 Hz, 1H), 4.98 (t, *J* = 6.2 Hz, 1H), 3.41 (q, *J* = 6.6 Hz, 2H), 3.25 (t, *J* = 6.7 Hz, 6H), 1.85-1.78 (m, 4H), 1.44 (s, 9H); ^{13}C NMR (151 MHz, $CDCl_3$) δ 156.0, 147.8, 134.8, 132.4, 131.5, 126.8, 115.7, 112.2, 80.2, 48.2, 39.6, 32.1, 28.5, 25.4; LC-MS (ESI) *m/z*: $[M+Na]^+$ calcd. for $C_{18}H_{25}N_3NaO_4S_2$: 434.12, found: 434.26.

***tert*-butyl 6-((2-((tert-butoxycarbonyl)amino)ethyl)thio)-5-(trifluoromethyl)nicotinate (3f)**

Following GP2, aryl halide **1f** (60 mg, 0.21 mmol, 1.0 eq.), Cs_2CO_3 (83 mg, 0.26 mmol, 1.2 eq.), and 2-(Boc-amino)ethanethiol (38 µl, 0.22 mmol, 1.05 eq.) were stirred for 1 h to afford the title compound (38 mg, 42%) as a pale-yellow solid. The crude was purified by column chromatography (silica 5 g, 0-25% EtOAc:pentane) with the product eluting at 25% EtOAc. ^{1}H NMR (600 MHz, $CDCl_3$) δ 9.05 (d, J = 2.0 Hz, 1H), 8.31 (d, J = 2.1 Hz, 1H), 4.97 (t, J = 5.6 Hz, 1H), 3.51-3.38 (m, 4H), 1.60 (s, 9H), 1.43 (s, 9H); ^{13}C NMR (151 MHz, $CDCl_3$) δ 163.5, 162.3, 155.9, 152.3, 135.2 (q, $^3J_{CF}$ = 5 Hz), 123.4 (q, $^2J_{CF}$ = 33 Hz), 123.2 (q, $^1J_{CF}$ = 273 Hz), 123.1, 82.8, 79.6, 40.1, 30.7, 28.5, 28.3; ^{19}F NMR (564 MHz, $CDCl_3$) δ -63.54; LC-MS (ESI) *m/z*: $[M+H]^+$ calcd. for $C_{18}H_{26}F_3N_2O_4S$: 423.16, found: 423.30.

***tert*-butyl 6-((2-((tert-butoxycarbonyl)amino)ethyl)thio)-5-nitronicotinate (3g)**

Following GP2, aryl halide **1g** (138 mg, 0.53 mmol, 1.0 eq.), Cs_2CO_3 (209 mg, 0.64 mmol, 1.2 eq.), and 2-(Boc-amino)ethanethiol (95 µl, 0.56 mmol, 1.05 eq.) were stirred for 45 min to afford the title compound (138 mg, 65%) as a yellow oil. The crude was purified by column chromatography (silica 5 g, 0-25% EtOAc:pentane) with the product eluting at 25% EtOAc. ^{1}H NMR (600 MHz, $CDCl_3$) δ 9.13 (d, J = 2.0 Hz, 1H), 8.88 (d, J = 2.0 Hz, 1H), 5.00 (t, J = 6.0 Hz, 1H), 3.48-3.35 (m, 4H), 1.58 (s, 9H), 1.39 (s, 9H); LC-MS (ESI) *m/z*: $[M+Na]^+$ calcd. for $C_{17}H_{25}N_3NaO_6S$: 422.14, found: 422.25.

methyl 6-((2-((*tert*-butoxycarbonyl)amino)ethyl)thio)pyridazine-3-carboxylate (3k)

Following GP2, aryl halide **1k** (146 mg, 0.85 mmol, 1.0 eq.), Cs_2CO_3 (331 mg, 1.02 mmol, 1.2 eq.), and 2-(Boc-amino)ethanethiol (0.15 ml, 0.89 mmol, 1.05 eq.) were stirred for 1 h to afford the title compound (198 mg, 75%) as a white solid. The crude did not require further purification. ^{1}H NMR (600 MHz, $CDCl_3$) δ 7.93 (d, J = 8.9 Hz, 1H), 7.46 (d, J = 9.0 Hz, 1H), 5.00 (s, 1H), 4.05 (s, 3H), 3.58-3.49 (m, 4H), 1.43 (s, 9H); ^{13}C NMR (151 MHz, $CDCl_3$) δ 165.4, 164.7, 156.0, 148.5, 126.5, 126.4, 79.7, 53.3, 40.1, 30.5, 28.5; LC-MS (ESI) *m/z*: $[M+Na]^+$ calcd. for $C_{13}H_{19}N_3NaO_4S$: 336.10, found: 336.16.

methyl 5-((2-((*tert*-butoxycarbonyl)amino)ethyl)thio)pyrazine-2-carboxylate (3l)

Following GP2, aryl halide **1l-Me** (165 mg, 0.96 mmol, 1.0 eq.), Cs_2CO_3 (374 mg, 1.15 mmol, 1.2 eq.), and 2-(Boc-amino)ethanethiol (0.17 ml, 1.00 mmol, 1.05 eq.) were stirred for 1 h to afford the title compound (146 mg, 49%) as a pale-yellow solid. The crude was purified by column chromatography (silica 5 g, 0-25% EtOAc:pentane) with the product eluting at 25% EtOAc. ^{1}H NMR (600 MHz, $CDCl_3$) δ 9.02 (d, J = 1.5 Hz, 1H), 8.50 (d, J = 1.5 Hz, 1H), 4.97 (t, J = 6.4 Hz, 1H), 3.99 (s, 3H), 3.45 (q, J = 6.3 Hz, 2H), 3.37 (t, J = 6.4 Hz, 2H), 1.41 (s, 9H); ^{13}C NMR (151 MHz, $CDCl_3$) δ 164.8, 161.3, 155.9, 145.5, 143.2, 138.1, 79.7, 53.0, 39.9, 30.0, 28.5; LC-MS (ESI) *m/z*: $[M+Na]^+$ calcd. for $C_{13}H_{19}N_3NaO_4S$: 336.10, found: 336.14.

methyl 2-((2-((*tert*-butoxycarbonyl)amino)ethyl)thio)thiazole-5-carboxylate (3m)

Following GP2, methyl 2-bromothiazole-5-carboxylate (226 mg, 1.02 mmol, 1.0 eq.), Cs_2CO_3 (397 mg, 1.22 mmol, 1.2 eq.), and 2-(Boc-amino)ethanethiol (0.19 ml, 1.12 mmol, 1.1 eq.) were stirred for 2 h to afford the title compound (134 mg, 41%) as a white gummy solid. The crude was purified by column chromatography (silica 5 g, 0-40% EtOAc:pentane) with the product eluting at 40% EtOAc. ^{1}H NMR (600 MHz, $CDCl_3$) δ 8.12 (s, 1H), 5.22 (t, J = 6.5 Hz, 1H), 3.82 (s, 3H), 3.46 (q, J = 6.3 Hz, 2H), 3.35 (t, J = 6.4 Hz, 2H), 1.37 (s, 9H); ^{13}C NMR (151 MHz, $CDCl_3$) δ 171.9, 161.2, 155.8,

148.1, 128.1, 79.6, 52.4, 40.0, 33.9, 28.4; LC-MS (ESI) *m/z*: $[M+Na]^+$ calcd. for $C_{12}H_{18}N_2NaO_4S_2$: 341.06, found: 341.13.

tert-butyl 4-((4-((2-((_tert_-butoxycarbonyl)amino)ethyl)thio)-2-(trifluoromethyl)phenyl) sulfonyl)piperazine-1-carboxylate (3o)

Following GP2, aryl halide **1o** (38 mg, 0.090 mmol, 1.0 eq.), Cs_2CO_3 (35 mg, 0.11 mmol, 1.2 eq.), and 2-(Boc-amino)ethanethiol (17 µl, 0.98 mmol, 1.1 eq.) were stirred for 2 h to afford the title compound (24 mg, 48%) as a white solid. The crude was purified by column chromatography (silica 5 g, 0-33% EtOAc:pentane) with the product eluting at 33% EtOAc. ^{1}H NMR (600 MHz, $CDCl_3$) δ 8.00 (d, *J* = 8.4 Hz, 1H), 7.71 (d, *J* = 2.1 Hz, 1H), 7.60 (d, *J* = 8.4 Hz, 1H), 4.90 (t, *J* = 6.2 Hz, 1H), 3.48 (t, *J* = 5.3 Hz, 4H), 3.40 (q, *J* = 6.6 Hz, 2H), 3.22-3.15 (m, 6H), 1.47-1.42 (m, 18H); ^{13}C NMR (151 MHz, $CDCl_3$) δ 155.9, 154.4, 144.9, 133.1, 133.0, 128.7, 128.6 (q, $^2J_{CF}$ = 33 Hz),126.6 (q, $^3J_{CF}$ = 7 Hz), 122.2 (q, $^1J_{CF}$ = 275 Hz), 80.6, 80.1, 45.5, 44.0, 42.8, 39.6, 31.9, 28.5, 28.5; ^{19}F NMR (564 MHz, $CDCl_3$) δ -57.66; LC-MS (ESI) *m/z*: $[M+Na]^+$ calcd. for $C_{23}H_{34}F_3N_3NaO_6S_2$: 592.17, found: 592.33.

tert-butyl 4-(((1_H_-1,2,3-triazol-1-yl)methyl)thio)-3-nitrobenzoate (18)

A 5 ml MW vial was charged with 1*H*-1,2,3-triazole (38 µl, 0.66 mmol, 1.2 eq.), 1,5,7-triazabicyclo[4.4.0]dec-5-ene (TBD) (91 mg, 0.66 mmol, 1.2 eq.), and TBAI (40 mg, 0.11 mmol, 0.2 eq.). The vial was purged with N_2, and dry DCM (2 ml) was added. The mixture was stirred at room temperature for 15 min followed by addition of **17** (166 mg, 0.55 mmol, 1.0 eq.) as a solution in dry DCM (2 ml). The pale-yellow solution was heated at 40°C until LC-MS indicated complete reaction. If required, additional triazole and TBD were added to consume all starting material. After 48 h, the reaction mixture was cooled to room temperature and diluted with DCM (50 ml). The organic layer was washed with NH_4Cl (2x50 ml) followed by brine (50 ml), dried over Na_2SO_4, and concentrated under reduced pressure. The crude was purified by column chromatography (high-capacity silica 10 g, 0-70% EtOAc:pentane) to afford **18** (eluted at 50% EtOAc) as a yellow crystalline solid (63 mg, 34%). Rf = 0.31 (EtOAc:pentane 1:1). ^{1}H NMR (600 MHz, $CDCl_3$) δ 8.69 (d, *J* = 1.9 Hz, 1H), 8.10 (dd, *J* = 8.4, 1.9 Hz, 1H), 7.85 (d, *J* = 1.1 Hz, 1H), 7.76 (d, *J* = 8.5 Hz, 1H), 7.70 (d, *J* = 1.2 Hz, 1H), 5.90 (s, 2H), 1.56 (s, 9H); ^{13}C NMR (151 MHz, $CDCl_3$) δ 163.0, 146.1, 137.2, 135.0, 134.4, 130.9, 127.3, 127.1, 123.4, 82.8, 50.2, 28.1; LC-MS (ESI) *m/z*: $[M+H]^+$ calcd. for $C_{14}H_{16}N_4O_4S$: 337.10, found: 337.19. *Note: The procedure was adapted from Dale, H. J. A. et al.*[13] *to favor alkylation of the 1-position of the triazole. The regioselectivity of N1:N2 was ca 80:20.*

tert-butyl 4-(((1_H_-1,2,3-triazol-4-yl)methyl)thio)-3-nitrobenzoate (20)

A 5 ml MW vial was charged with **19** (262 mg, 0.89 mmol, 1.0 eq.), **Fmoc-N$_3$** (237 mg, 0.89 mmol, 1.0 eq.), $[Cu(CH_3CN)_4]PF_6$ (34 mg, 0.089 mmol, 10 mol%) and $CHCl_3$ (4 ml). The vial was sealed, and the yellow solution was heated at 60°C until LC-MS indicated full conversion to the intermediate Fmoc-protected triazole. After 8 h, the reaction mixture was cooled to room temperature, the cap was removed, and piperidine (0.44 ml, 4.47 mmol, 5.0 eq.) was added dropwise. The solution changed color from orange to dark-green and there was gas formation. The reaction mixture was stirred at room temperature until LC-MS indicated complete reaction. After 45 min, the solution was diluted with DCM (20 ml) and washed with water (25 ml) followed by brine (25 ml). The organic layer was dried over Na_2SO_4 and concentrated under reduced pressure. The crude was purified by reverse-phase column chromatography (C18 silica 12 g, 0-

100% MeCN:water) to afford **20** (eluted at 50% MeCN) as a yellow crystalline solid (149 mg, 49%). Rf = 0.35 (EtOAc:DCM 1:4). ^{1}H NMR (600 MHz, $CDCl_3$) δ 8.74 (d, *J* = 1.9 Hz, 1H), 8.09 (dd, *J* = 8.5, 1.9 Hz, 1H), 7.74 (s, 1H), 7.59 (d, *J* = 8.5 Hz, 1H), 4.38 (s, 2H), 1.59 (s, 9H); ^{13}C NMR (201 MHz, $CDCl_3$) δ 163.6, 145.3, 142.6, 141.7, 133.9, 129.2, 127.2, 126.5, 82.7, 28.2, 27.4; LC-MS (ESI) *m/z*: $[M+H]^+$ calcd. for $C_{14}H_{16}N_4O_4S$: 337.10, found: 337.25. *Note: The click procedure with azidoformates was adapted from Lee, H. et al.*[14]

3.4 Synthesis and Characterization of Intermediates for BTK Labeling Probes

2-((5-(methoxycarbonyl)pyridin-2-yl)sulfonyl)acetic acid (21)

In a 25 ml round-bottom flask, **6e** (225 mg, 0.71 mmol, 1.0 eq.) was dissolved in dry DCM (6 ml), cooled to 0°C, and purged with N_2. Then TFA (2 ml, 26 mmol, 37 eq.) was added dropwise and the pale-yellow solution was stirred at room temperature until LC-MS indicated complete reaction. After 16 h, the mixture was concentrated under reduced pressure to afford **21** as a yellow solid (185 mg, 100%), that was used in the next step without further purification. ^{1}H NMR (600 MHz, $(CD_3)_2SO$) δ 9.23 (dd, *J* = 2.1, 0.8 Hz, 1H), 8.63 (dd, *J* = 8.2, 2.1 Hz, 1H), 8.21 (dd, *J* = 8.2, 0.8 Hz, 1H), 4.71 (s, 2H), 3.94 (s, 3H); ^{13}C NMR (151 MHz, $(CD_3)_2SO$) δ 164.0, 163.8, 159.5, 150.4, 139.9, 129.1, 122.0, 56.1, 53.0; LC-MS (ESI) *m/z*: $[M+H]^+$ calcd. for $C_9H_{10}NO_6S$: 260.02, found: 260.00.

methyl 6-((2-(4-(((6-amino-5-(4-phenoxyphenyl)pyrimidin-4-yl)amino)methyl)piperidin-1-yl)-2-oxoethyl)sulfonyl)nicotinate (23)

In a 25 ml round-bottom flask purged with N_2, **22** (133 mg, 0.32 mmol, 1.2 eq.) was dissolved in dry DCM (3 ml) and basified by addition of DIPEA (56 µL, 0.32 mmol, 1.2 eq.). The colorless solution was stirred at room temperature for 10 min. A second 25 ml round-bottom flask was charged with **21** (70 mg, 0.27 mmol, 1.0 eq.) and HATU (123 mg, 0.32 mmol, 1.2 eq.), and the flask was purged with N_2. Then, dry DCM (5 ml), DIPEA (56 µL, 0.32 mmol, 1.2 eq.), and the amine solution in the other flask, were all added in rapid succession. The yellow solution was stirred at room temperature until LC-MS indicated complete reaction. After 40 min, the reaction mixture was concentrated under reduced pressure, and the crude was purified by reverse-phase column chromatography (C18 silica 12 g, 0-100% MeCN:water) to afford **23** (eluted at 50% MeCN) as a white crystalline solid (103 mg, 62%). ^{1}H NMR (600 MHz, $(CD_3)_2CO$) δ 9.21 (dd, *J* = 2.1, 0.8 Hz, 1H), 8.62 (dd, *J* = 8.1, 2.1 Hz, 1H), 8.25 (s, 1H), 8.17 (dd, *J* = 8.1, 0.8 Hz, 1H), 7.42 (dd, *J* = 8.6, 7.4 Hz, 2H), 7.38 (d, *J* = 8.7 Hz, 2H), 7.18 (tt, *J* = 7.4, 1.1 Hz, 1H), 7.14 (d, *J* = 8.7 Hz, 2H), 7.08 (dd, *J* = 8.7, 1.1 Hz, 2H), 6.12 (t, *J* = 6.2 Hz, 1H), 5.95 (br s, 2H), 4.79 (d, *J* = 15.0 Hz, 1H), 4.73 (d, *J* = 15.1 Hz, 1H), 4.35-4.28 (m, 1H), 4.15-4.07 (m, 1H), 3.99 (s, 3H), 3.39 (td, *J* = 6.7, 1.8 Hz, 2H), 3.12 (td, *J* = 13.0, 2.8 Hz, 1H), 2.54 (td, *J* = 12.8, 2.9 Hz, 1H), 1.97-1.88 (m, 1H), 1.81-1.74 (m, 1H), 1.71-1.65 (m, 1H), 1.29 (qd, *J* = 12.4, 4.3 Hz, 1H), 1.01 (qd, *J* = 12.4, 4.3 Hz, 1H); ^{13}C NMR (151 MHz, $(CD_3)_2CO$) δ 165.0, 161.7, 161.1, 160.4, 158.7, 157.5, 157.1, 153.3, 151.3, 140.2, 133.3, 130.9, 130.0, 126.4, 124.7, 122.6, 120.9, 120.0, 97.2, 55.6, 53.2, 47.3, 46.8, 42.5, 37.0, 31.0; LC-MS (ESI) *m/z*: $[M+H]^+$ calcd. for $C_{31}H_{33}N_6O_6S$: 617.22, found: 617.39.

6-((2-(4-(((6-amino-5-(4-phenoxyphenyl)pyrimidin-4-yl)amino)methyl)piperidin-1-yl)-2-oxoethyl)sulfonyl)nicotinic acid (24)

In a 25 ml round-bottom flask, **23** (50 mg, 0.081 mmol, 1.0 eq.) was dissolved in 1,4-dioxane (3 ml) and MeOH (2 ml), and cooled to 0°C. Then 1 M NaOH (1 ml, 1 mmol, 12.3 eq.) was added dropwise over 5 min and the solution instantly turned yellow upon addition of the base. The reaction mixture was stirred at 0°C until LC-MS indicated complete reaction. After 40 min, the mixture was concentrated under reduced pressure to remove the organic solvents. The residual yellow oil was dissolved in water (5 ml) and acidified to pH 1 using 1 M HCl, to afford a white precipitate. The solids were filtered, washed with water followed by Et_2O, and dried under high vacuum to afford **24** as a white solid (31 mg, 64%). ^1H NMR (600 MHz, $(CD_3)_2SO$) δ 9.18 (d, *J* = 2.1 Hz, 1H), 8.56 (dd, *J* = 8.2, 2.1 Hz, 1H), 8.18 (s, 1H), 8.11 (d, *J* = 8.1 Hz, 1H), 7.43 (t, *J* = 7.7 Hz, 2H), 7.25 (d, *J* = 8.5 Hz, 2H), 7.18 (t, *J* = 7.4 Hz, 1H), 7.16-7.09 (m, 4H), 6.41 (br s, 1H), 6.30 (br s, 2H), 4.92 (d, *J* = 15.4 Hz, 1H), 4.86 (d, *J* = 15.3 Hz, 1H), 4.14 (d, *J* = 13.1 Hz, 1H), 3.95 (d, *J* = 13.5 Hz, 1H), 3.18 (t, *J* = 6.9 Hz, 2H), 2.97 (t, *J* = 11.7 Hz, 1H), 1.85-1.73 (m, 1H), 1.65-1.48 (m, 2H), 1.10 (qd, *J* = 12.5, 3.9 Hz, 1H), 0.86 (qd, *J* = 12.6, 4.4 Hz, 1H); ^{13}C NMR (151 MHz, $(CD_3)_2SO$) δ 165.1, 160.0, 159.6, 156.8, 156.3, 150.4, 139.7, 132.5, 130.1, 129.9, 125.5, 123.7, 121.7, 119.9, 119.0, 95.3, 54.8, 45.8, 45.6, 41.3, 35.4, 29.8, 29.0; LC-MS (ESI) *m/z*: $[M+H]^+$ calcd. for $C_{30}H_{31}N_6O_6S$: 603.20, found: 603.33.

2-((4-(*tert*-butoxycarbonyl)-2-nitrophenyl)thio)acetic acid (26)

1 M NaOH (0.89 ml, 0.89 mmol, 1.2 eq.) was added dropwise at room temperature to a solution of **2b** (243 mg, 0.74 mmol, 1.0 eq.) in THF (5 ml). The solution changed color from orange to black upon addition of base and was stirred at room temperature until LC-MS indicated complete reaction. After 2.5 h, the reaction mixture was concentrated under reduced pressure. The residue was diluted in water (10 ml), and the aqueous phase was washed with Et_2O (20 ml). The aqueous phase was acidified to pH 2-3 using 1 M HCl to afford an orange precipitate. The solids were filtered, washed with 1 M HCl followed by pentane, and dried under high vacuum to afford **26** as an orange solid (194 mg, 83%). ^1H NMR (600 MHz, $(CD_3)_2SO$) δ 8.57 (d, *J* = 1.9 Hz, 1H), 8.11 (dd, *J* = 8.5, 2.0 Hz, 1H), 7.69 (d, *J* = 8.6 Hz, 1H), 4.06 (s, 2H), 1.56 (s, 9H); ^{13}C NMR (151 MHz, $(CD_3)_2SO$) δ 169.5, 163.0, 144.8, 142.0, 133.4, 127.9, 127.7, 126.1, 82.0, 34.8, 27.7; LC-MS (ESI) *m/z*: $[M-H]^-$ calcd. for $C_{13}H_{14}NO_6S$: 312.05, found: 312.09.

***tert*-butyl 4-((2-(4-(((6-amino-5-(4-phenoxyphenyl)pyrimidin-4-yl)amino)methyl)piperidin-1-yl)-2-oxoethyl)thio)-3-nitrobenzoate (27)**

In a 50 ml round-bottom flask purged with N_2, **22** (251 mg, 0.61 mmol, 1.2 eq.) was suspended in dry DCM (4 ml) and basified by addition of DIPEA (0.11 ml, 0.61 mmol, 1.2 eq.). The brown suspension was stirred at room temperature for 10 min. A second 50 ml round-bottom flask was charged with **26** (159 mg, 0.51 mmol, 1.0 eq.) and HATU (232 mg, 0.61 mmol, 1.2 eq.), and the flask was purged with N_2. Then, dry DCM (6 ml), DIPEA (0.11 ml, 0.61 mmol, 1.2 eq.), and the amine solution in the other flask, were all added in rapid succession. The orange solution was stirred at room temperature until LC-MS indicated complete reaction. After 3 h, the reaction mixture was concentrated under reduced pressure, and the crude was purified by reverse-phase column chromatography (C18 silica 12 g, 5-95% MeOH:water) to afford **27** (eluted at 80% MeOH) as a yellow solid (144 mg, 42%). ^1H NMR (600 MHz, $(CD_3)_2CO$) δ 8.67 (d, *J* = 1.9 Hz, 1H), 8.12 (dd, *J* = 8.5, 1.9 Hz, 1H), 8.02 (s, 1H), 7.92 (d, *J* = 8.6 Hz, 1H), 7.41 (dd, *J* = 8.7, 7.4 Hz, 2H), 7.31 (d, *J* = 8.6 Hz, 2H), 7.16 (tt, *J* = 7.4, 1.1 Hz, 1H), 7.12 (d, *J* = 8.6 Hz, 2H), 7.08 (dd, *J* = 8.7, 1.1 Hz, 2H), 5.23 (t, *J* = 6.1 Hz, 1H), 5.06 (br s, 2H), 4.50-4.42 (m, 1H), 4.23-4.15 (m, 2H), 4.14-4.07 (m, 1H), 3.31 (t,

J = 6.6 Hz, 2H), 3.15 (td, *J* = 13.6, 2.9 Hz, 1H), 2.61 (td, *J* = 12.7, 3.0 Hz, 1H), 1.96-1.86 (m, 1H), 1.78 (d, *J* = 13.3 Hz, 1H), 1.73-1.67 (m, 1H), 1.61 (s, 9H), 1.23 (qd, *J* = 12.2, 4.2 Hz, 1H), 1.03 (qd, *J* = 12.3, 4.3 Hz, 1H); ^{13}C NMR (151 MHz, $(CD_3)_2CO$) δ 165.6, 164.1, 161.1, 158.0, 157.9, 157.5, 146.4, 143.6, 134.1, 133.1, 130.8, 129.6, 129.1, 129.0, 127.1, 124.5, 120.8, 119.8, 97.7, 82.7, 46.8, 46.6, 42.8, 37.2, 36.4, 31.3, 28.2; LC-MS (ESI) *m/z*: $[M+H]^+$ calcd. for $C_{35}H_{39}N_6O_6S$: 671.26, found: 671.43.

4-((2-(4-(((6-amino-5-(4-phenoxyphenyl)pyrimidin-4-yl)amino)methyl)piperidin-1-yl)-2-oxoethyl)thio)-3-nitrobenzoic acid (28)

In a round-bottom flask, **27** (140 mg, 0.21 mmol, 1.0 eq.) was dissolved in dry DCM (1.5 ml) and TFA (0.40 ml, 5.22 mmol, 25 eq.) was added dropwise. The reaction mixture was stirred at room temperature until LC-MS indicated complete reaction. After 2.5 h, the mixture was concentrated under reduced pressure, and the residual yellow oil was precipitated with 1 M HCl. The solids were filtered, washed with water followed by hexane, and dried under high vacuum to afford **28** as a yellow solid (128 mg, 100%). ^{1}H NMR (600 MHz, $(CD_3)_2SO$) δ 8.61 (d, *J* = 1.9 Hz, 1H), 8.36 (s, 1H), 8.11 (dd, *J* = 8.5, 1.9 Hz, 1H), 7.81 (d, *J* = 8.6 Hz, 1H), 7.44 (t, *J* = 7.9 Hz, 2H), 7.27 (d, *J* = 8.2 Hz, 2H), 7.20 (t, *J* = 7.4 Hz, 1H), 7.15 (d, *J* = 8.1 Hz, 2H), 7.13 (d, *J* = 8.0 Hz, 2H), 7.09 (t, *J* = 6.2 Hz, 1H), 7.00 (br s, 2H), 4.33-4.20 (m, 3H), 4.01 (d, *J* = 13.5 Hz, 1H), 3.29-3.20 (m, 2H), 3.08-3.00 (m, 1H), 2.58 (td, *J* = 12.8, 2.9 Hz, 1H), 1.88-1.79 (m, 1H), 1.68-1.57 (m, 2H), 1.13 (qd, *J* = 12.3, 4.1 Hz, 1H), 0.94 (qd, *J* = 12.4, 4.3 Hz, 1H); ^{13}C NMR (151 MHz, $(CD_3)_2SO$) δ 165.4, 164.8, 157.3, 156.1, 148.7, 144.9, 142.3, 133.5, 132.6, 130.1, 128.4, 127.5, 126.3, 123.8, 123.7, 119.9, 119.1, 94.7, 45.9, 45.3, 41.5, 35.5, 35.4, 29.8, 29.0; LC-MS (ESI) *m/z*: $[M-H]^-$ calcd. for $C_{31}H_{29}N_6O_6S$: 613.19, found: 613.32.

4-((2-(4-(((6-amino-5-(4-phenoxyphenyl)pyrimidin-4-yl)amino)methyl)piperidin-1-yl)-2-oxoethyl)thio)-*N*-(2-(3-(5,5-difluoro-7,9-dimethyl-5H-4λ^4,5λ^4-dipyrrolo[1,2-*c*:2',1'-*f*][1,3,2]diazaborinin-3-yl)propanamido)ethyl)-3-nitrobenzamide (29)

In a 2 ml microwave vial purged with N_2, **25** (25 mg, 0.067 mmol, 1.2 eq.) was dissolved in dry DCM:DMF 3:1 (1.5 ml) and basified by addition of DIPEA (12 µL, 0.067 mmol, 1.2 eq.). The red solution was stirred at room temperature for 10 min. A 5 ml microwave vial was charged with **28** (34 mg, 0.056 mmol, 1.0 eq.) and HATU (25 mg, 0.067 mmol, 1.2 eq.), and the flask was purged with N_2. Then, dry DCM:DMF 3:1 (1.5 ml), DIPEA (12 µL, 0.067 mmol, 1.2 eq.), and the amine solution in the other vial, were all added in rapid succession. The red solution was stirred at room temperature until HPLC indicated complete reaction. After 40 min, the reaction mixture was diluted with DCM (15 ml) and NH_4Cl (15 ml). The layers were separated, and the aqueous layer was extracted with DCM (2x10 ml). The combined organic layers were washed with NH_4Cl (2x25 ml) followed by brine (25 ml), and concentrated under reduced pressure. The crude was purified by column chromatography (high-capacity silica 10 g, 0-10% MeOH:DCM) to afford **29** (eluted at 10% MeOH) as an orange solid (21 mg, 41%). Rf = 0.46 (MeOH:DCM 1:9). ^{1}H NMR (600 MHz, $CDCl_3$) δ 8.65 (d, *J* = 2.0 Hz, 1H), 8.13 (s, 1H), 7.89 (dd, *J* = 8.5, 2.0 Hz, 1H), 7.79 (t, *J* = 4.2 Hz, 1H), 7.69 (d, *J* = 8.6 Hz, 1H), 7.39 (dd, *J* = 8.6, 7.4 Hz, 2H), 7.23 (d, *J* = 8.6 Hz, 2H), 7.18 (tt, *J* = 7.4, 1.1 Hz, 1H), 7.12 (d, *J* = 8.7 Hz, 2H), 7.09 (dd, *J* = 8.7, 1.1 Hz, 2H), 6.99 (s, 1H), 6.73 (d, *J* = 4.0 Hz, 1H), 6.53 (t, *J* = 5.6 Hz, 1H), 6.17 (d, *J* = 4.0 Hz, 1H), 6.10 (s, 1H), 4.80 (br s, 2H), 4.54 (d, *J* = 13.3 Hz, 1H), 4.48 (t, *J* = 6.2 Hz, 1H), 3.90 (d, *J* = 13.9 Hz, 1H), 3.83–3.77 (m, 2H), 3.49-3.42 (m, 4H), 3.33-3.22 (m, 4H), 3.15-3.08 (m, 1H), 2.69 (t, *J* = 7.2 Hz, 2H), 2.61 (td, *J* = 13.0, 3.0 Hz, 1H), 2.52 (s, 3H), 2.22

(s, 3H), 1.91-1.82 (m, 1H), 1.78 (d, *J* = 12.8 Hz, 1H), 1.69 (d, *J* = 13.2 Hz, 1H), 1.20 (qd, *J* = 12.6, 4.3 Hz, 1H), 1.07 (qd, *J* = 12.4, 4.3 Hz, 1H); ^{13}C NMR (151 MHz, $CDCl_3$) δ 174.5, 165.5, 164.8, 160.8, 160.2, 159.3, 158.4, 156.4, 156.3, 156.1, 145.5, 144.6, 140.2, 135.4, 133.4, 131.9, 131.7, 131.4, 130.2, 128.2, 127.6, 126.1, 125.2, 124.4, 124.0, 120.8, 120.0, 119.9, 117.0, 97.5, 46.7, 46.3, 42.6, 42.5, 39.2, 36.3, 35.5, 30.5, 29.5, 24.9, 15.1, 11.5; ^{19}F NMR (564 MHz, $CDCl_3$) δ -144.31 (dd, *J* = 66.9, 32.8 Hz); LC-MS (ESI) *m/z*: $[M+H]^+$ calcd. for $C_{47}H_{49}BF_2N_{10}O_6S$: 931.37, found: 931.70.

***tert*-butyl 5-((2-methoxy-2-oxoethyl)thio)pyrazine-2-carboxylate (30)**

Following GP2, aryl halide **1l-tBu** (450 mg, 2.09 mmol, 1.0 eq.), Cs_2CO_3 (819 mg, 2.51 mmol, 1.2 eq.), and methyl thioglycolate (0.21ml, 2.30 mmol, 1.1 eq.) were stirred for 1 h 40 min to afford the title compound (407 mg, 68%) as a white crystalline solid. The crude was purified by column chromatography (high-capacity silica 10 g, 0-10% MeOH:DCM) with the product eluting at 6% MeOH. ^{1}H NMR (600 MHz, $CDCl_3$) δ 8.95 (d, J = 1.5 Hz, 1H), 8.56 (d, J = 1.5 Hz, 1H), 4.01 (s, 2H), 3.74 (s, 3H), 1.61 (s, 9H); ^{13}C NMR (151 MHz, $CDCl_3$) δ 169.1, 163.2, 158.7, 145.2, 142.7, 140.1, 83.0, 53.0, 31.7, 28.2; LC-MS (ESI) m/z: $[M+Na]^+$ calcd. for $C_{12}H_{16}N_2NaO_4S$: 307.07, found: 307.19.

2-((5-(*tert*-butoxycarbonyl)pyrazin-2-yl)thio)acetic acid (31)

1 M NaOH (2.47 ml, 2.47 mmol, 1.2 eq.) was added dropwise at room temperature to a solution of **30** (586 mg, 2.06 mmol, 1.0 eq.) in THF (4 ml). The solution changed from colorless to orange upon addition of base and was stirred at room temperature until LC-MS indicated complete reaction. After 30 min, the reaction mixture was concentrated under reduced pressure. The residue was diluted in water (5 ml), and the aqueous phase was washed with Et_2O (5 ml). The aqueous phase was acidified to pH 2-3 using 1 M HCl to afford a yellow precipitate. The solids were filtered, washed with 1 M HCl followed by pentane, and dried under high vacuum to afford **31** as a yellow solid (354 mg, 64%). Rf = 0.11 (MeCN:water 2:3, reverse-phase TLC). ^{1}H NMR (600 MHz, $(CD_3)_2SO$) δ 8.85 (d, *J* = 1.5 Hz, 1H), 8.58 (d, *J* = 1.5 Hz, 1H), 3.70 (s, 2H), 1.55 (s, 9H); ^{13}C NMR (151 MHz, $(CD_3)_2SO$) δ 169.0, 163.8, 163.5, 144.8, 142.5, 138.5, 82.2, 37.5, 28.2; LC-MS (ESI) *m/z*: $[M-H]^-$ calcd. for $C_{11}H_{13}N_2O_4S$: 269.06, found: 269.11.

***tert*-butyl 5-((2-(4-(((6-amino-5-(4-phenoxyphenyl)pyrimidin-4-yl)amino)methyl)piperidin-1-yl)-2-oxoethyl)thio)pyrazine-2-carboxylate (32)**

In a 25 ml round-bottom flask purged with N_2, **22** (223 mg, 0.55 mmol, 1.0 eq.) was suspended in dry DCM (3 ml) and basified by addition of DIPEA (0.13 ml, 0.78 mmol, 1.4 eq.). The brown suspension was stirred at room temperature for 10 min. A 50 ml round-bottom flask was charged with **31** (164 mg, 0.61 mmol, 1.1 eq.) and HATU (294 mg, 0.77 mmol, 1.4 eq.), and the flask was purged with N_2. Then, dry DCM (5 ml) and DIPEA (0.13 ml, 0.78 mmol, 1.4 eq.) were added and the mixture was pre-stirred for 15 min before addition of the amine solution in the other flask. The orange solution was stirred at room temperature until LC-MS indicated complete reaction. After 2 h, the reaction mixture was concentrated under reduced pressure, and the crude was purified by reverse-phase column chromatography (C18 silica 12 g, 30-100% MeCN:water) to afford **32** (eluted at 50% MeCN) as a white solid (167 mg, 48%). ^{1}H NMR (600 MHz, $(CD_3)_2CO$) δ 8.90 (d, *J* = 1.5 Hz, 1H), 8.61 (d, *J* = 1.5 Hz, 1H), 8.02 (s, 1H), 7.40 (dd, *J* = 8.6, 7.3 Hz, 2H), 7.31 (d, *J* = 8.6 Hz, 2H), 7.16 (t, *J* = 7.5 Hz, 1H), 7.11 (d, *J* = 8.6 Hz, 2H), 7.07 (d, *J* = 7.5 Hz, 2H), 5.27 (t, *J* = 6.1 Hz, 1H), 5.19 (br s, 2H), 4.43 (d, *J* = 12.5 Hz, 1H), 4.32 (d, *J* = 15.2 Hz, 1H), 4.26 (d, *J* = 15.2 Hz, 1H), 4.05 (d, *J* = 12.6 Hz, 1H), 3.37-3.26 (m, 2H), 3.16-3.08 (m, 1H), 2.63-2.55 (m, 1H), 1.95-1.85 (m, 1H), 1.78

(d, J = 13.3 Hz, 1H), 1.69 (d, J = 13.4 Hz, 1H), 1.59 (s, 9H), 1.23 (qd, J = 12.1, 4.1 Hz, 1H), 1.03 (qd, J = 12.2, 4.3 Hz, 1H); ^{13}C NMR (151 MHz, $(CD_3)_2CO$) δ 165.6, 163.8, 161.3, 161.0, 157.9, 157.8, 157.4, 145.5, 143.2, 140.6, 133.1, 130.8, 128.9, 124.5, 120.8, 119.8, 97.7, 82.6, 46.8, 46.7, 42.8, 37.1, 34.0, 31.1, 30.4, 28.3; LC-MS (ESI) *m/z*: $[M+H]^+$ calcd. for $C_{33}H_{38}N_7O_4S$: 628.27, found: 628.51.

5-((2-(4-(((6-amino-5-(4-phenoxyphenyl)pyrimidin-4-yl)amino)methyl)piperidin-1-yl)-2-oxoethyl)thio)pyrazine-2-carboxylic acid (33)

In a round-bottom flask, **32** (167 mg, 0.27 mmol, 1.0 eq.) was dissolved in dry DCM (3 ml) and TFA (0.51 ml, 6.64 mmol, 25 eq.) was added dropwise. The reaction mixture was stirred at room temperature until LC-MS indicated complete reaction. After 2.5 h, the mixture was concentrated under reduced pressure, and the residual yellow oil was precipitated with 1 M HCl. The solids were filtered, washed with water followed by hexane, and dried under high vacuum to afford **33** as a beige solid (152 mg, 100%). ^{1}H NMR (600 MHz, CD_3OD) δ 9.01 (br s, 1H), 8.60 (br s, 1H), 8.26 (s, 1H), 7.40 (dd, J = 8.5, 7.3 Hz, 2H), 7.30 (d, J = 8.2 Hz, 2H), 7.19-7.15 (m, 3H), 7.11 (d, J = 8.5 Hz, 2H), 4.46 (d, J = 12.9 Hz, 1H), 4.36 (d, J = 15.5 Hz, 1H), 4.22 (d, J = 15.5 Hz, 1H), 4.12 (d, J = 13.1 Hz, 1H), 3.44-3.34 (m, 2H), 3.20-3.13 (m, 1H), 2.71-2.63 (m, 1H), 1.98-1.89 (m, 1H), 1.78 (d, J = 13.1 Hz, 1H), 1.69 (d, J = 13.2 Hz, 1H), 1.29 (qd, J = 12.3, 4.1 Hz, 1H), 1.10 (qd, J = 12.3, 4.1 Hz, 1H); ^{13}C NMR (151 MHz, CD_3OD) δ 167.8, 166.8, 162.1, 161.6, 160.2, 157.8, 153.6, 149.2, 146.4, 143.6, 139.6, 133.5, 131.1, 125.2, 124.3, 121.2, 120.6, 96.7, 47.4, 47.3, 43.8, 37.5, 33.4, 31.2, 30.5; LC-MS (ESI) *m/z*: $[M-H]^-$ calcd. for $C_{29}H_{28}N_7O_4S$: 570.19, found: 570.34.

5-((2-(4-(((6-amino-5-(4-phenoxyphenyl)pyrimidin-4-yl)amino)methyl)piperidin-1-yl)-2-oxoethyl)thio)-*N*-(2-(3-(5,5-difluoro-7,9-dimethyl-5*H*-4λ^4,5λ^4-dipyrrolo[1,2-*c*:2',1'-*f*][1,3,2]diazaborinin-3-yl)propanamido)ethyl)pyrazine-2-carboxamide (34)

In a 10 ml round-bottom flask purged with N_2, **25** (24 mg, 0.065 mmol, 1.2 eq.) was dissolved in dry DCM:DMF 3:1 (1.5 ml) and basified by addition of DIPEA (18 µL, 0.11 mmol, 2.0 eq.). The red solution was stirred at room temperature for 10 min. A second 10 ml round-bottom flask was charged with **33** (31 mg, 0.054 mmol, 1.0 eq.) and HATU (25 mg, 0.065 mmol, 1.2 eq.), and the flask was purged with N_2. Then, dry DCM:DMF 3:1 (1.5 ml) and DIPEA (18 µL, 0.11 mmol, 2.0 eq.) were added and the mixture was pre-stirred for 15 min before addition of the amine solution in the other flask. The dark-red solution was stirred at room temperature until LC-MS indicated complete reaction. After 45 min, the reaction mixture was diluted with DCM (20 ml) and NH_4Cl (20 ml). The layers were separated, and the aqueous layer was extracted with DCM (2x20 ml). The combined organic layers were washed with NH_4Cl (2x40 ml) followed by brine (50 ml), and concentrated under reduced pressure. The crude was purified by column chromatography (high-capacity silica 5 g, 0-20% MeOH:DCM) to elute the compound at 10% MeOH. Further purification by preparative TLC (MeOH:DCM 1:19) was performed to afford **34** as a red solid (17 mg, 35%). Rf = 0.40 (MeOH:DCM 1:19). ^{1}H NMR (600 MHz, $CDCl_3$) δ 9.01 (d, J = 1.5 Hz, 1H), 8.36 (d, J = 1.5 Hz, 1H), 8.17 (s, 1H), 8.01 (t, J = 5.7 Hz, 1H), 7.40 (dd, J = 8.5, 7.5 Hz, 2H), 7.23 (d, J = 8.7 Hz, 2H), 7.19 (tt, J = 7.5, 1.0 Hz, 1H), 7.13 (d, J = 8.7 Hz, 2H), 7.09 (dd, J = 8.7, 1.0 Hz, 2H), 7.05 (s, 1H), 6.80 (d, J = 4.0 Hz, 1H), 6.35 (t, J = 5.0 Hz, 1H), 6.25 (d, J = 4.0 Hz, 1H), 6.11 (s, 1H), 4.80 (br s, 2H), 4.60-4.53 (m, 2H), 4.22 (d, J = 15.0 Hz, 1H), 4.10 (d, J = 15.0 Hz, 1H), 3.99 (d, J = 13.5 Hz, 1H), 3.53-3.41 (m, 4H), 3.39-3.32 (m, 1H), 3.30-3.22 (m, 3H), 3.13-3.06 (m, 1H), 2.64 (t, J = 7.4 Hz, 2H), 2.63-2.57 (m, 1H), 2.53 (s, 3H), 2.24 (s, 3H), 1.89-1.80 (m, 1H), 1.74 (d, J = 12.7 Hz, 1H), 1.67 (d, J = 12.6 Hz, 1H),

1.18 (qd, J = 12.6, 4.1 Hz, 1H), 1.09 (qd, J = 12.6, 4.2 Hz, 1H); ^{13}C NMR (151 MHz, $CDCl_3$) δ 172.7, 165.7, 164.1, 160.5, 160.2, 159.4, 158.6, 158.3, 157.3, 156.0, 155.3, 144.1, 143.2, 141.2, 139.8, 135.3, 133.5, 131.9, 130.2, 128.3, 125.5, 124.5, 124.0, 120.6, 120.1, 119.9, 117.5, 97.3, 46.4, 42.6, 39.9, 39.7, 36.5, 35.8, 33.0, 30.5, 29.5, 24.9, 15.1, 11.5; ^{19}F NMR (564 MHz, $CDCl_3$) δ -144.52 (dd, J = 66.7, 33.1 Hz); LC-MS (ESI) *m/z*: $[M+H]^+$ calcd. for $C_{45}H_{49}BF_2N_{11}O_4S$: 888.37, found: 888.67.

5-((2-(4-(((6-amino-5-(4-phenoxyphenyl)pyrimidin-4-yl)amino)methyl)piperidin-1-yl)-2-oxoethyl)thio)-*N*-(but-3-yn-1-yl)pyrazine-2-carboxamide (35)

A 25 ml round-bottom flask was charged with **33** (77 mg, 0.14 mmol, 1.0 eq.) and HATU (62 mg, 0.16 mmol, 1.2 eq.), and the flask was purged with N_2. Then, dry DCM (3 ml) and DIPEA (28 µL, 0.16 mmol, 1.2 eq.) were added and the mixture was pre-stirred for 15 min before addition of 1-amino-3-butyne (13 µL, 0.16 mmol, 1.2 eq.) as a solution in dry DCM (1 ml). The reaction mixture was stirred at room temperature until LC-MS indicated complete reaction. After 1.5 h, the reaction mixture was concentrated under reduced pressure, and the crude was purified by reverse-phase column chromatography (C18 silica 6 g, 20-100% MeCN:water) to afford **35** (eluted at 50% MeCN) as a white crystalline solid (26 mg, 31%). ^{1}H NMR (600 MHz, $CDCl_3$) δ 9.05 (s, 1H), 8.40 (s, 1H), 8.21 (br s, 1H), 7.97 (t, J = 6.4 Hz, 1H), 7.39 (t, J = 7.6 Hz, 2H), 7.23 (d, J = 8.1 Hz, 2H), 7.18 (t, J = 7.5 Hz, 1H), 7.12 (d, J = 8.0 Hz, 2H), 7.08 (d, J = 8.0 Hz, 2H), 4.87 (br s, 2H), 4.61 (t, J = 6.1 Hz, 1H), 4.55 (d, J = 13.4 Hz, 1H), 4.21 (d, J = 15.1 Hz, 1H), 4.12 (d, J = 15.1 Hz, 1H), 3.99 (d, J = 13.6 Hz, 1H), 3.61 (q, J = 6.6 Hz, 2H), 3.39-3.31 (m, 1H), 3.30-3.22 (m, 1H), 3.10 (t, J = 12.9 Hz, 1H), 2.60 (t, J = 12.7 Hz, 1H), 2.50 (t, J = 7.0 Hz, 2H), 2.03 (s, 1H), 1.90-1.80 (m, 1H), 1.76 (d, J = 13.2 Hz, 1H), 1.67 (d, J = 13.3 Hz, 1H), 1.20 (q, J = 11.3 Hz, 1H), 1.08 (q, J = 12.6 Hz, 1H); ^{13}C NMR (151 MHz, $CDCl_3$) δ 165.6, 163.4, 160.2, 159.6, 158.6, 158.2, 155.9, 155.0, 143.2, 141.2, 139.7, 131.9, 130.1, 125.5, 124.5, 120.0, 119.9, 81.3, 70.3, 46.4, 46.3, 42.5, 38.1, 36.4, 33.1, 30.4, 29.5, 19.6; LC-MS (ESI) *m/z*: $[M+H]^+$ calcd. for $C_{33}H_{35}N_8O_3S$: 623.25, found: 623.57.

***tert*-butyl (*R*)-5-((2-(3-(4-amino-3-(4-phenoxyphenyl)-1*H*-pyrazolo[3,4-*d*]pyrimidin-1-yl)piperidin-1-yl)-2-oxoethyl)thio)pyrazine-2-carboxylate (37)**

A 50 ml round-bottom flask was charged with **31** (187 mg, 0.69 mmol, 1.0 eq.) and HATU (315 mg, 0.83 mmol, 1.2 eq.), and the flask was purged with N_2. Dry DCM (4 ml) and DIPEA (0.14 ml, 0.83 mmol, 1.2 eq.) were added, and the yellow suspension was pre-stirred at room temperature for 10 min. Then (*R*)-3-(4-phenoxyphenyl)-1-(piperidin-3-yl)-1*H*-pyrazolo[3,4-*d*]pyrimidin-4-amine (**36**) (320 mg, 0.83 mmol, 1.2 eq.) in dry DCM (4 ml) was added, and the orange solution was stirred at room temperature until LC-MS indicated complete reaction. After 1.5 h, the reaction mixture was concentrated under reduced pressure. The residue was dissolved in EtOAc (60 ml) and washed with NH_4Cl (3x40 ml) followed by brine (40 ml). The organic layer was dried over Na_2SO_4 and concentrated under reduced pressure. The crude was purified by column chromatography (silica 25 g, 0-10% MeOH:DCM) to afford **37** (eluted at 6% MeOH) as a pale-yellow crystalline solid (412 mg, 93%). Rf = 0.18 (MeOH:DCM 1:19). ^{1}H NMR (600 MHz, $CDCl_3$) δ 8.98 (d, J = 1.5 Hz, 1H), 8.91 (d, J = 1.5 Hz, 1H), 8.58 (d, J = 1.5 Hz, 1H), 8.54 (d, J = 1.5 Hz, 1H), 8.36-8.33 (m, 2H), 7.66-7.61 (m, 4H), 7.41-7.36 (m, 4H), 7.20-7.12 (m, 6H), 7.10-7.06 (m, 4H), 5.61 (br s, 4H), 4.99-4.93 (m, 1H), 4.91-4.84 (m, 1H), 4.79 (dd, J = 12.9, 4.6 Hz, 1H), 4.48 (d, J = 13.3 Hz, 1H), 4.36-4.27 (m, 2H), 4.26-4.17 (m, 2H), 4.10-4.00 (m, 2H), 3.89 (dd, J = 13.3, 10.2 Hz, 1H), 3.43 (t, J = 11.8 Hz, 1H), 3.29 (t, J = 11.5 Hz, 1H), 2.95 (t, J = 10.9 Hz, 1H), 2.45-2.30 (m, 2H), 2.29-2.22 (m, 2H), 2.07-1.94 (m, 2H), 1.86-1.67 (m, 4H), 1.63 (s, 9H), 1.61 (s, 9H); ^{13}C NMR (151 MHz, $CDCl_3$)

δ 166.3, 166.0, 163.3, 159.5, 159.4, 158.8, 158.7, 158.0, 156.5, 156.4, 156.1, 156.0, 154.5, 154.4, 145.2, 145.1, 144.2, 144.1, 142.9, 142.8, 139.9, 139.8, 130.1, 130.1, 127.9, 127.7, 124.3, 124.2, 119.7, 119.7, 119.2, 98.7, 83.0, 82.9, 53.3, 52.5, 50.5, 46.5, 42.8, 32.9, 32.6, 30.3, 30.1, 28.3, 25.2, 23.9; LC-MS (ESI) *m/z*: $[M+H]^+$ calcd. for $C_{33}H_{35}N_8O_4S$: 639.25, found: 639.53. *Note: The $^1H/^{13}C$ NMR show two sets of peaks indicating two species in solution. This is likely due to cis-trans rotamers of the piperidine tertiary amide in proximity of the stereocenter. The two species were present at roughly a 1:1 ratio. All signals for the pair are listed above.*

(*R*)-5-((2-(3-(4-amino-3-(4-phenoxyphenyl)-1*H*-pyrazolo[3,4-d]pyrimidin-1-yl)piperidin-1-yl)-2-oxoethyl)thio)pyrazine-2-carboxylic acid (38)

In a 50 ml round-bottom flask, **37** (412 mg, 0.65 mmol, 1.0 eq.) was dissolved in dry DCM (6 ml) and cooled to 0°C. Then TFA (2 ml, 26 mmol, 40 eq.) was added dropwise and the yellow solution was stirred at room temperature until LC-MS indicated complete reaction. After 3 h, the mixture was concentrated under reduced pressure, and the crude was purified by reverse-phase column chromatography (C18 silica 30 g, 0-100% MeCN:water) to afford **38** (eluted at 60% MeCN) as an orange crystalline solid (323 mg, 86%). ^1H NMR (600 MHz, $(CD_3)_2SO$) δ 8.90 (br s, 2H), 8.45 (br s, 2H), 8.30-8.20 (m, 2H), 7.66 (d, *J* = 8.1 Hz, 4H), 7.43 (t, *J* = 7.6 Hz, 4H), 7.22-7.08 (m, 10H), 4.93 (t, *J* = 10.5 Hz, 1H), 4.67 (t, *J* = 10.8 Hz, 1H), 4.49 (d, *J* = 12.4 Hz, 1H), 4.42-3.99 (m, 6H), 3.82 (t, *J* = 11.2 Hz, 1H), 3.29 -3.16 (m, 2H), 2.99 (t, *J* = 11.9 Hz, 1H), 2.31-1.49 (m, 9H); ^{13}C NMR (151 MHz, $(CD_3)_2SO$) δ 166.0, 165.9, 158.2, 157.1, 156.3, 155.7, 154.1, 153.9, 144.4, 143.3, 143.2, 130.1, 130.1, 127.9, 123.8, 119.0, 97.5, 97.4, 52.4, 52.0, 49.5, 45.9, 45.7, 41.9, 32.6, 30.4, 29.5, 24.5, 23.3; LC-MS (ESI) *m/z*: $[M+H]^+$ calcd. for $C_{29}H_{27}N_8O_4S$: 583.19, found: 583.40. *Note: The $^1H/^{13}C$ NMR show two sets of peaks indicating two species in solution. This is likely due to cis-trans rotamers of the piperidine tertiary amide in proximity of the stereocenter. The two species were present at roughly a 1:1 ratio. All signals for the pair are listed above.*

(*R*)-5-((2-(3-(4-amino-3-(4-phenoxyphenyl)-1*H*-pyrazolo[3,4-d]pyrimidin-1-yl)piperidin-1-yl)-2-oxoethyl)thio)-*N*-(but-3-yn-1-yl)pyrazine-2-carboxamide (39)

A 50 ml round-bottom flask was charged with **38** (98 mg, 0.17 mmol, 1.0 eq.) and HATU (77 mg, 0.20 mmol, 1.2 eq.), and the flask was purged with N_2. Dry DCM:DMF 3:1 (4 ml) and DIPEA (57 µL, 0.34 mmol, 2.0 eq.) were added, and the orange suspension was pre-stirred at room temperature for 10 min. Then 1-amino-3-butyne (28 µL, 0.34 mmol, 2.0 eq.) in dry DCM:DMF 3:1 (2 ml) was added, and the orange solution was stirred at room temperature until LC-MS indicated complete reaction. After 2 h, the reaction mixture was concentrated under reduced pressure. The residue was dissolved in EtOAc (25 ml) and washed with NH_4Cl (3x25 ml) followed by brine (25 ml). The organic layer was dried over Na_2SO_4 and concentrated under reduced pressure. The crude was purified by column chromatography (high-capacity silica 10 g, 0-10% MeOH:DCM) to afford **39** (eluted at 8% MeOH) as a beige crystalline solid (68 mg, 63%). Rf = 0.24 (MeOH:DCM 1:19). ^1H NMR (600 MHz, $CDCl_3$) δ 9.12 (d, *J* = 1.5 Hz, 1H), 8.99 (d, *J* = 1.5 Hz, 1H), 8.43 (d, *J* = 1.5 Hz, 1H), 8.39 (d, *J* = 1.5 Hz, 1H), 8.35-8.32 (m, 2H), 7.98 (t, *J* = 6.2 Hz, 1H), 7.92 (t, *J* = 6.2 Hz, 1H), 7.64 (t, *J* = 8.3 Hz, 4H), 7.42-7.36 (m, 4H), 7.19-7.12 (m, 6H), 7.10-7.06 (m, 4H), 5.60 (br s, 4H), 4.96-4.83 (m, 2H), 4.78 (dd, *J* = 13.2, 4.2 Hz, 1H), 4.49 (d, *J* = 13.3 Hz, 1H), 4.42-4.18 (m, 4H), 4.09-4.02 (m, 2H), 3.89 (dd, *J* = 13.3, 10.3 Hz, 1H), 3.67-3.58 (m, 4H), 3.45-3.40 (m, 1H), 3.33-3.27 (m, 1H), 2.98-2.90 (m, 1H), 2.52 (qd, *J* = 6.4, 2.6 Hz, 4H), 2.42-2.30 (m, 2H), 2.29-2.21 (m, 2H), 2.06-2.03 (m, 2H), 1.99-1.93 (m, 1H), 1.86-1.65 (m, 4H); ^{13}C NMR (151 MHz, $CDCl_3$) δ 166.5, 166.1, 163.5, 163.4,

159.5, 159.2, 158.8, 158.7, 158.0, 156.5, 156.4, 156.1, 155.9, 154.4, 144.2, 144.1, 143.4, 143.2, 141.3, 141.2, 139.8, 139.7, 130.1, 127.9, 127.7, 124.2, 124.2, 119.7, 119.7, 119.3, 98.7, 81.4, 81.3, 70.4, 53.2, 52.5, 50.5, 46.5, 42.8, 38.1, 33.0, 32.4, 30.3, 30.2, 25.2, 23.9, 19.7; LC-MS (ESI) *m/z*: $[M+H]^+$ calcd. for $C_{33}H_{31}N_9O_3S$: 634.23, found: 634.50. *Note: The $^1H/^{13}C$ NMR show two sets of peaks indicating two species in solution. This is likely due to cis-trans rotamers of the piperidine tertiary amide in proximity of the stereocenter. The two species were present at roughly a 1:1 ratio. All signals for the pair are listed above.*

tert-butyl 5-((2-hydroxyethyl)thio)pyrazine-2-carboxylate (40)

A 5 ml microwave vial was charged with **1l-tBu** (361 mg, 1.68 mmol, 1.0 eq.) and Cs_2CO_3 (657 mg, 2.02 mmol, 1.2 eq.). The vial was purged with N_2 and dry DMF (4 ml) was added. Then 2-mercaptoethanol (0.13 ml, 1.85 mmol, 1.1 eq.) was added and the orange suspension was stirred at room temperature until LC-MS indicated complete reaction. After 2 h, the reaction mixture was poured into water (20 ml), and the vial was rinsed with EtOAc (20 ml). The layers were separated, and the aqueous layer was extracted with EtOAc (2x20 ml). The combined organic layers were washed with water (50 ml) followed by brine (50 ml), dried over Na_2SO_4, and concentrated under reduced pressure. The crude was purified by reverse-phase column chromatography (C18 silica 12 g, 0-100% MeCN:water) to afford **40** (eluted at 40% MeCN) as a white crystalline solid (174 mg, 40%). Rf = 0.54 (MeCN:water 3:2, reverse-phase TLC). ^1H NMR (600 MHz, $CDCl_3$) δ 8.95 (d, *J* = 1.5 Hz, 1H), 8.57 (d, *J* = 1.5 Hz, 1H), 3.94 (t, *J* = 5.7 Hz, 2H), 3.44 (t, *J* = 5.8 Hz, 2H), 2.71 (br s, 1H), 1.63 (s, 9H); ^{13}C NMR (151 MHz, $CDCl_3$) δ 163.2, 160.4, 145.0, 143.4, 140.1, 83.1, 62.2, 33.1, 28.3; LC-MS (ESI) *m/z*: $[M+H]^+$ calcd. for $C_{11}H_{17}N_2O_3S$: 257.10, found: 257.24.

tert-butyl 5-((2-(4-amino-3-(4-phenoxyphenyl)-1*H*-pyrazolo[3,4-d]pyrimidin-1-yl)ethyl)thio)pyrazine-2-carboxylate (42)

A 50 ml round-bottom flask was charged with solid-supported PS-PPh_3 (1.85 mmol/g loading, 733 mg, 1.36 mmol, 2.0 eq.). The flask was evacuated and refilled with N_2 three times. Dry THF (5 ml) was added, and the flask was cooled to 0°C. Then DIAD (0.27 ml, 1.36 mmol, 2.0 eq.) was added dropwise and the yellow suspension was stirred at 0°C for 20 min. Next, a suspension of **40** (174 mg, 0.68 mmol, 1.0 eq.) and 3-(4-phenoxyphenyl)-1*H*-pyrazolo[3,4-*d*]pyrimidin-4-amine (**41**) (267 mg, 0.89 mmol, 1.3 eq.) in dry THF (5 ml) was added dropwise at 0°C. The yellow suspension was stirred at room temperature until LC-MS indicated complete reaction. After 2 h, the reaction mixture was filtered over celite, and the flask was rinsed with EtOAc and DCM. The filtrate was concentrated under reduced pressure, and the crude was purified by column chromatography (silica 25 g, 0-10% MeOH:DCM) to afford **42** (eluted at 5% MeOH) as a white solid (184 mg, 50%). Rf = 0.23 (MeOH:DCM 1:19). ^1H NMR (600 MHz, $CDCl_3$) δ 8.97 (d, *J* = 1.5 Hz, 1H), 8.45 (d, *J* = 1.5 Hz, 1H), 8.32 (s, 1H), 7.63 (d, *J* = 8.6 Hz, 2H), 7.38 (dd, *J* = 8.5, 7.3 Hz, 2H), 7.18-7.13 (m, 3H), 7.08 (d, *J* = 7.5 Hz, 2H), 5.75 (br s, 2H), 4.78 (t, *J* = 6.6 Hz, 2H), 3.82 (t, *J* = 6.6 Hz, 2H), 1.62 (s, 9H); ^{13}C NMR (151 MHz, $CDCl_3$) δ 163.3, 159.7, 158.7, 158.0, 156.4, 156.2, 155.1, 145.3, 144.4, 143.1, 139.8, 130.1, 130.0, 127.7, 124.2, 119.7, 119.2, 98.6, 82.9, 46.0, 29.2, 28.3; LC-MS (ESI) *m/z*: $[M+H]^+$ calcd. for $C_{28}H_{28}N_7O_3S$: 542.20, found: 542.41.

5-((2-(4-amino-3-(4-phenoxyphenyl)-1*H*-pyrazolo[3,4-d]pyrimidin-1-yl)ethyl)thio)pyrazine-2-carboxylic acid (43)

In a 50 ml round-bottom flask, **42** (184 mg, 0.34 mmol, 1.0 eq.) was dissolved in dry DCM (4.5 ml) and cooled to 0°C. Then TFA (1.5 ml, 20 mmol, 58 eq.) was added dropwise and the pale-yellow solution was stirred at room temperature until LC-MS indicated complete reaction. After 3 h, the mixture was concentrated under reduced pressure. DCM and MeCN were added to the residue, and upon sonication a white solid crashed out. The solid was filtered, washed with DCM, and dried under high vacuum to afford **43** as a white solid (164 mg, 100%). ^{1}H NMR (600 MHz, $(CD_3)_2SO$) δ 8.88 (d, *J* = 1.5 Hz, 1H), 8.62 (d, *J* = 1.5 Hz, 1H), 8.29 (s, 1H), 7.61 (d, *J* = 8.6 Hz, 2H), 7.44 (dd, *J* = 8.6, 7.3 Hz, 2H), 7.20 (tt, *J* = 7.4, 1.1 Hz, 1H), 7.16-7.11 (m, 4H), 4.72 (t, *J* = 6.2 Hz, 2H), 3.81 (t, *J* = 6.2 Hz, 2H); ^{13}C NMR (151 MHz, $(CD_3)_2SO$) δ 165.1, 159.3, 157.4, 156.1, 153.9, 144.7, 144.4, 142.9, 139.0, 130.2, 130.0, 127.1, 123.9, 119.1, 118.9, 96.9, 45.7, 40.1, 28.7; LC-MS (ESI) *m/z*: $[M+H]^+$ calcd. for $C_{24}H_{20}N_7O_3S$: 486.13, found: 486.33.

5-((2-(4-amino-3-(4-phenoxyphenyl)-1*H*-pyrazolo[3,4-*d*]pyrimidin-1-yl)ethyl)thio)-*N*-(but-3-yn-1-yl)pyrazine-2-carboxamide (44)

A 50 ml round-bottom flask was charged with **43** (113 mg, 0.23 mmol, 1.0 eq.) and HATU (106 mg, 0.28 mmol, 1.2 eq.), and the flask was purged with N_2. Dry DCM:DMF 3:1 (4 ml) and DIPEA (79 µL, 0.47 mmol, 2.0 eq.) were added, and the pale-yellow solution was pre-stirred at room temperature for 10 min. Then 1-amino-3-butyne (38 µL, 0.47 mmol, 2.0 eq.) in dry DCM:DMF 3:1 (2 ml) was added, and the mixture was stirred at room temperature until LC-MS indicated complete reaction. After 2 h, the reaction mixture was concentrated under reduced pressure. The residue was dissolved in EtOAc (25 ml) and washed with NH_4Cl (3x25 ml) followed by brine (25 ml). The organic layer was dried over Na_2SO_4 and concentrated under reduced pressure. The crude was purified by column chromatography (high-capacity silica 10 g, 50-100% EtOAc:pentane, then 0-20% MeOH:EtOAc) to afford **44** (eluted at 100% EtOAc) as a white solid (56 mg, 45%). ^{1}H NMR (600 MHz, $(CD_3)_2SO$) δ 8.87-8.82 (m, 2H), 8.55 (d, *J* = 1.5 Hz, 1H), 8.19 (s, 1H), 7.59 (d, *J* = 8.8 Hz, 2H), 7.44 (dd, *J* = 8.6, 7.4 Hz, 2H), 7.19 (tt, *J* = 7.7, 1.4 Hz, 1H), 7.15-7.11 (m, 4H), 4.69 (t, *J* = 6.2 Hz, 2H), 3.80 (t, *J* = 6.2 Hz, 2H), 3.41 (q, *J* = 7.0 Hz, 2H), 2.82 (t, *J* = 2.6 Hz, 1H), 2.42 (td, *J* = 7.2, 2.7 Hz, 2H); ^{13}C NMR (151 MHz, $(CD_3)_2SO$) δ 162.8, 158.7, 158.1, 157.2, 156.2, 155.8, 154.7, 143.4, 142.5, 141.6, 140.0, 130.1, 130.0, 127.7, 123.8, 119.0, 118.9, 97.2, 82.0, 72.2, 45.4, 40.1, 37.9, 28.9, 18.6; LC-MS (ESI) *m/z*: $[M+H]^+$ calcd. for $C_{28}H_{25}N_8O_2S$: 537.18, found: 537.38.

4 References

1. In Schrödinger Release 2025-2: Maestro, Schrödinger, LLC, New York, NY, 2025., Schrödinger Release 2025-2: Maestro, Schrödinger, LLC, New York, NY, 2025.
2. In Desmond 2.2 User Manual, Schrödinger, LLC, New York, NY, 2009., Desmond 2.2 User Manual, Schrödinger, LLC, New York, NY, 2009.
3. Zielkiewicz, J. Structural Properties of Water: Comparison of the SPC, SPCE, TIP4P, and TIP5P Models of Water. J. Chem. Phys. 2005, 123, 104501.
4. Lu, C.; Wu, C.; Ghoreishi, D.; Chen, W.; Wang, L.; Damm, W.; Ross, G. A.; Dahlgren, M. K.; Russell, E.; Von Bargen, C. D.; Abel, R.; Friesner, R. A.; Harder, E. D. OPLS4: Improving Force Field Accuracy on Challenging Regimes of Chemical Space. J. Chem. Theory Comput. 2021, 17, 4291-4300.
5. Martyna, G. J.; Tobias, D. J.; Klein, M. L. Constant Pressure Molecular Dynamics Algorithms. J. Chem. Phys. 1994, 101, 4177-4189.
6. Sastry, G. M.; Adzhigirey, M.; Day, T.; Annabhimoju, R.; Sherman, W. Protein and Ligand Preparation: Parameters, Protocols, and Influence on Virtual Screening Enrichments. J. Comput. Aided Mol. Des. 2013, 27, 221-234.
7. Maltsev, O. V.; Pöthig, A.; Hintermann, L. Synthesis of Soai Aldehydes for Asymmetric Autocatalysis by Desulfurative Cross-Coupling. Org. Lett. 2014, 16, 1282–1285.
8. Divakaran, A.; Talluri, S. K.; Ayoub, A. M.; Mishra, N. K.; Cui, H.; Widen, J. C.; Berndt, N.; Zhu, J.-Y.; Carlson, A. S.; Topczewski, J. J.; Schonbrunn, E. K.; Harki, D. A.; Pomerantz, W. C. K. Molecular Basis for the N-Terminal Bromodomain-and-Extra-Terminal-Family Selectivity of a Dual Kinase−Bromodomain Inhibitor. J. Med. Chem. 2018, 61, 9316-9334.
9. Pichon, M. M.; Drelinkiewicz, D.; Lozano, D.; Moraru, R.; Hayward, L. J.; Jones, M.; McCoy, M. A.; Allstrum-Graves, S.; Balourdas, D.-I.; Joerger, A. C.; Whitby, R. J.; Goldup, S. M.; Wells, N.; Langley, G. J.; Herniman, J. M.; Baud, M. G. J. Structure−Reactivity Studies of 2-Sulfonylpyrimidines Allow Selective Protein Arylation. Bioconjugate Chem. 2023, 34, 1679-1687.
10. Dyachenko, V. D.; Matusov, I. O.; Dyachenko, I. V.; Nenajdenko, V. G. Knoevenagel Reactions of Indole-3-carbaldehyde. Synthesis of 3-Substituted Indole Derivatives. Russ. J. Org. Chem. 2018, 54, 1777–1784.
11. Dyachenko, V. D.; Dyachenko, A. D. Synthesis and Unusual Reaction of Piperidinium 3-Cyano-5-ethoxycarbonyl-4-(1H-indol-3-yl)-6-methyl-1,4-dihydro-pyridine-2-thiolate with Glacial Acetic Acid. Russ. J. Org. Chem. 2006, 42, 1091–1092.
12. Meyer, A. R.; Popescu, M. V.; Sau, A.; Damrauer, N. H.; Paton, R. S.; Yoon, T. P. Combined Synthetic, Spectroscopic, and Computational Insights Into a General Method for Photosensitized Alkene Aziridination. ACS Catal. 2024, 14, 12310-12317.
13. Dale, H. J. A.; Hodges, G. R.; Lloyd-Jones, G. C. Taming Ambident Triazole Anions: Regioselective Ion Pairing Catalyzes Direct N-Alkylation with Atypical Regioselectivity. J. Am. Chem. Soc. 2019, 141, 7181-7193.
14. Lee, H.; Lee, J. K.; Min, S.-J.; Seo, H.; Lee, Y.; Rhee, H. Copper(I)-Catalyzed Synthesis of 1,4-Disubstituted 1,2,3-Triazoles from Azidoformates and Aryl Terminal Alkynes. J. Org. Chem. 2018, 83, 4805-4811.

5 NMR Spectra

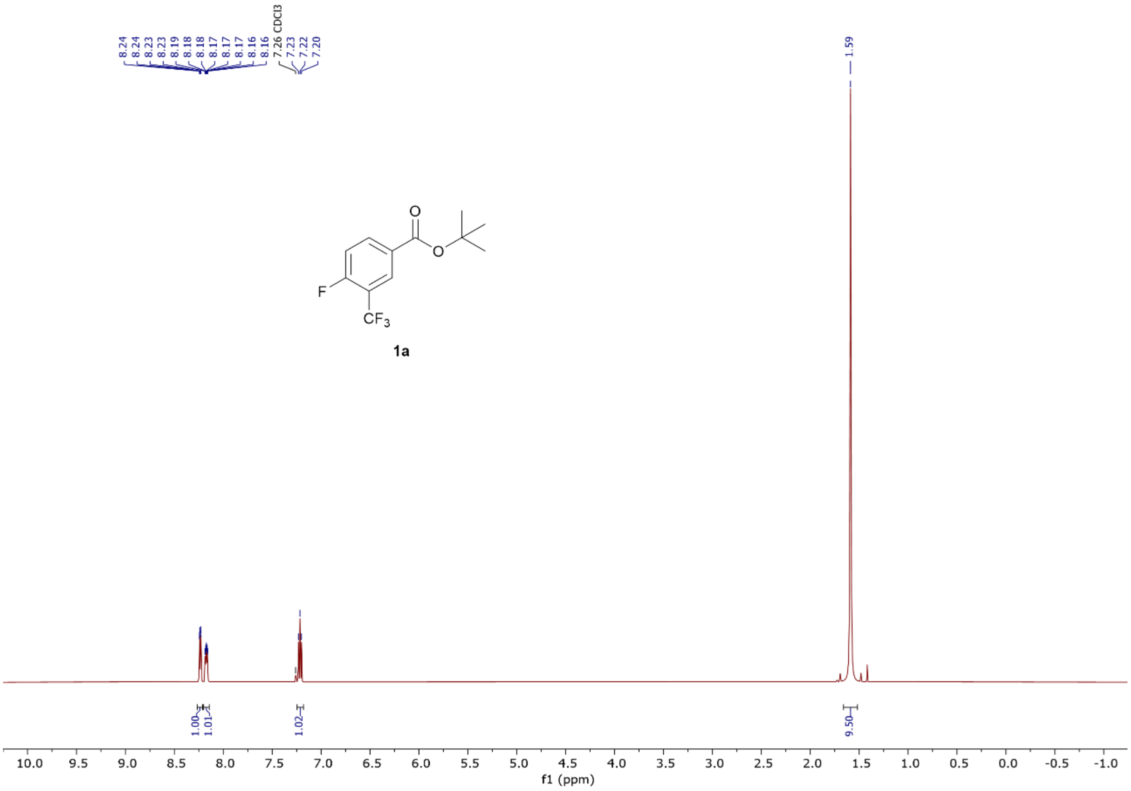

Figure S26. ^{1}H NMR spectrum of compound **1a** (600 MHz, $CDCl_3$).

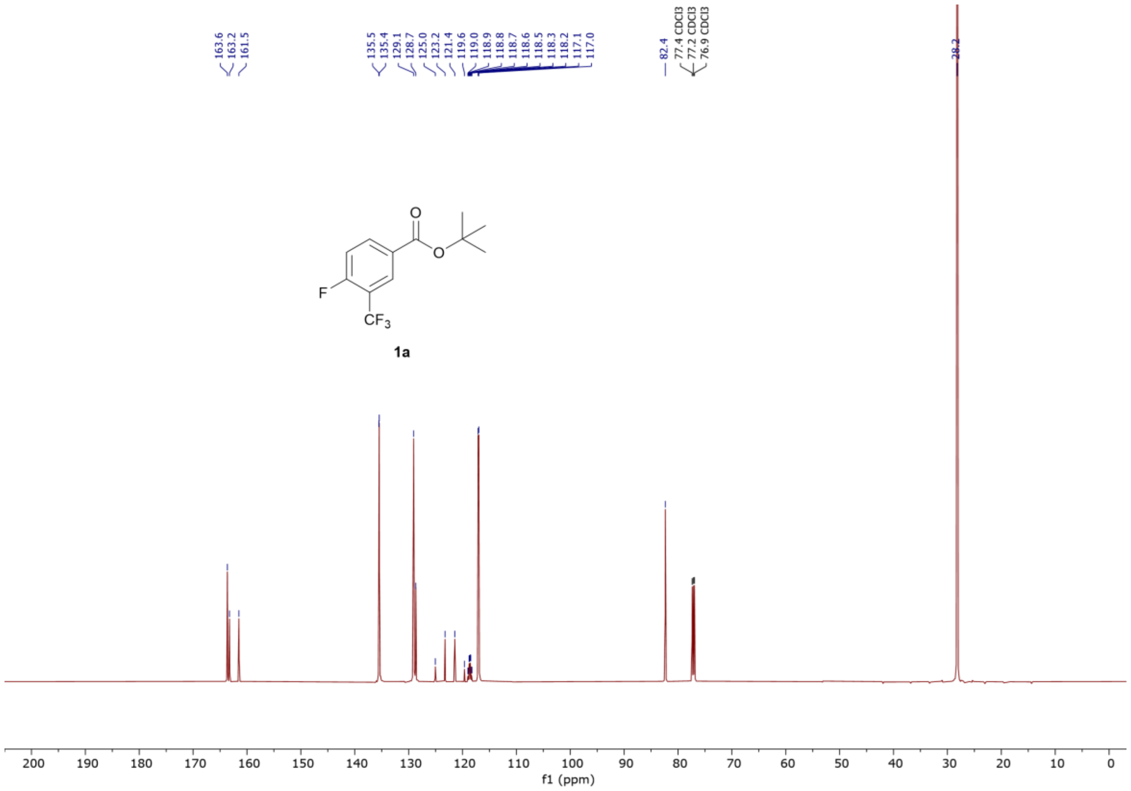

Figure S27. ^{13}C NMR spectrum of compound **1a** (151 MHz, $CDCl_3$).

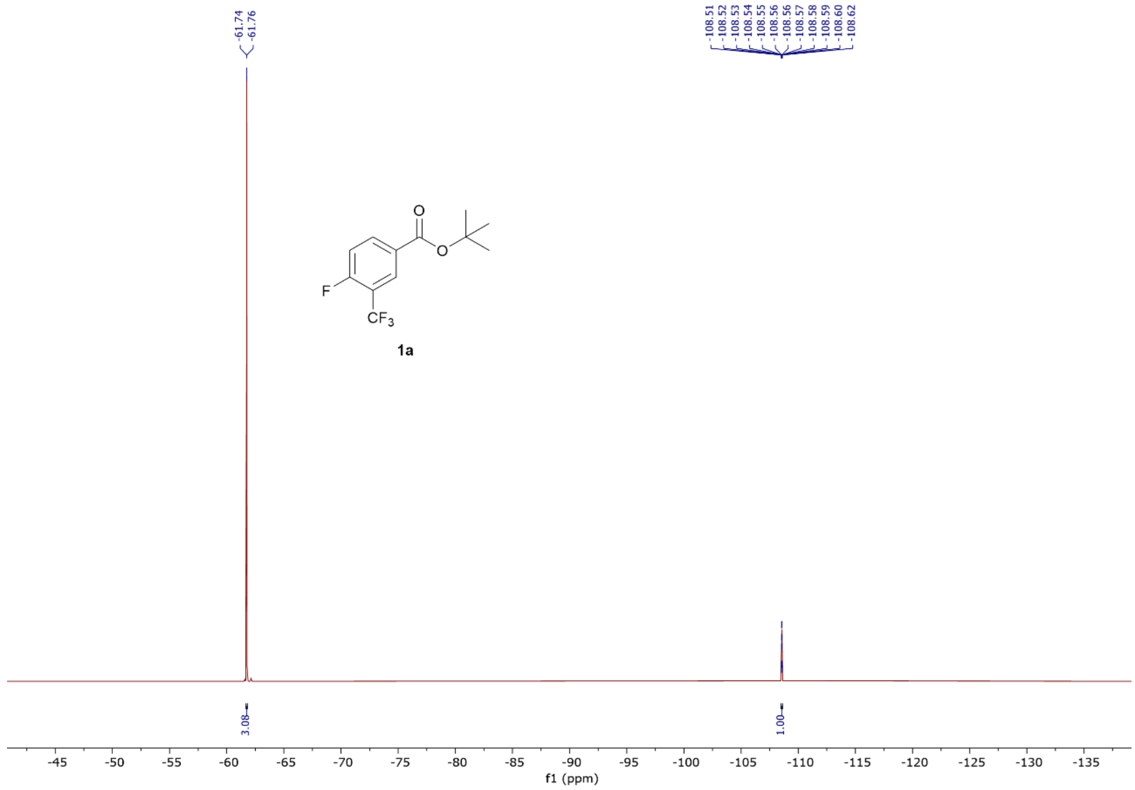

Figure S28. ^{19}F NMR spectrum of compound **1a** (564 MHz, $CDCl_3$).

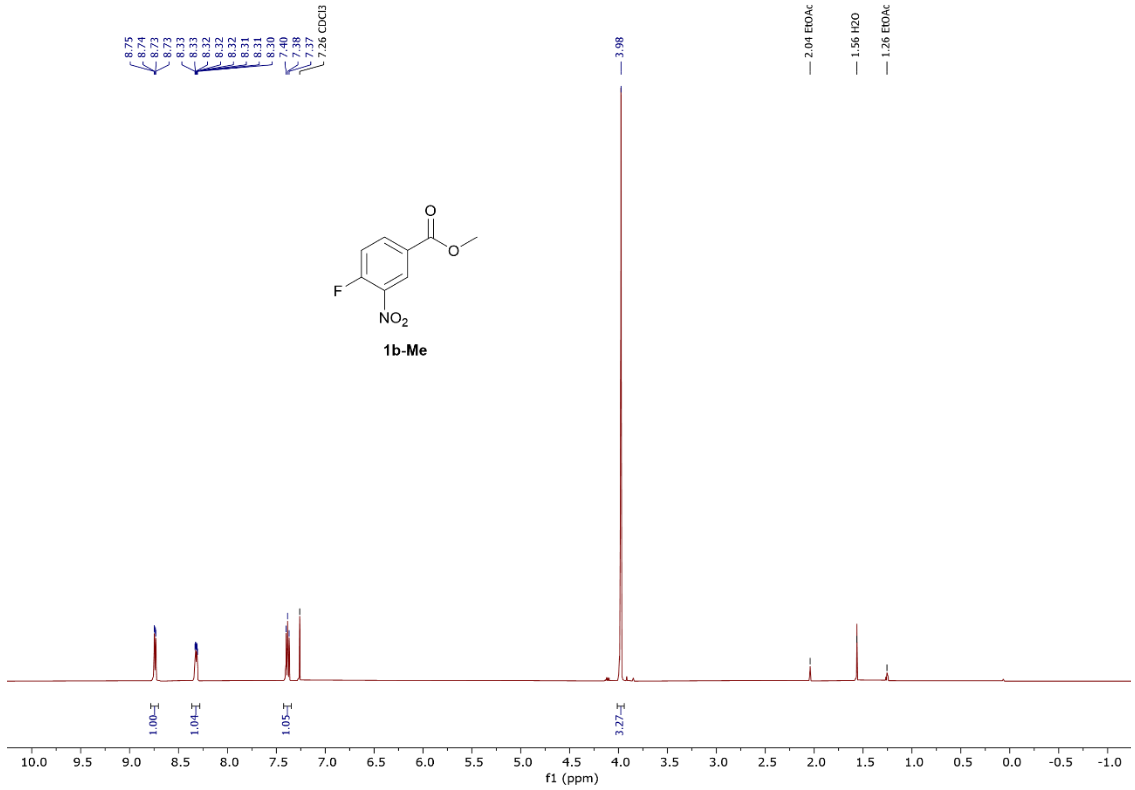

Figure S29. ^{1}H NMR spectrum of compound **1b-Me** (600 MHz, $CDCl_3$).

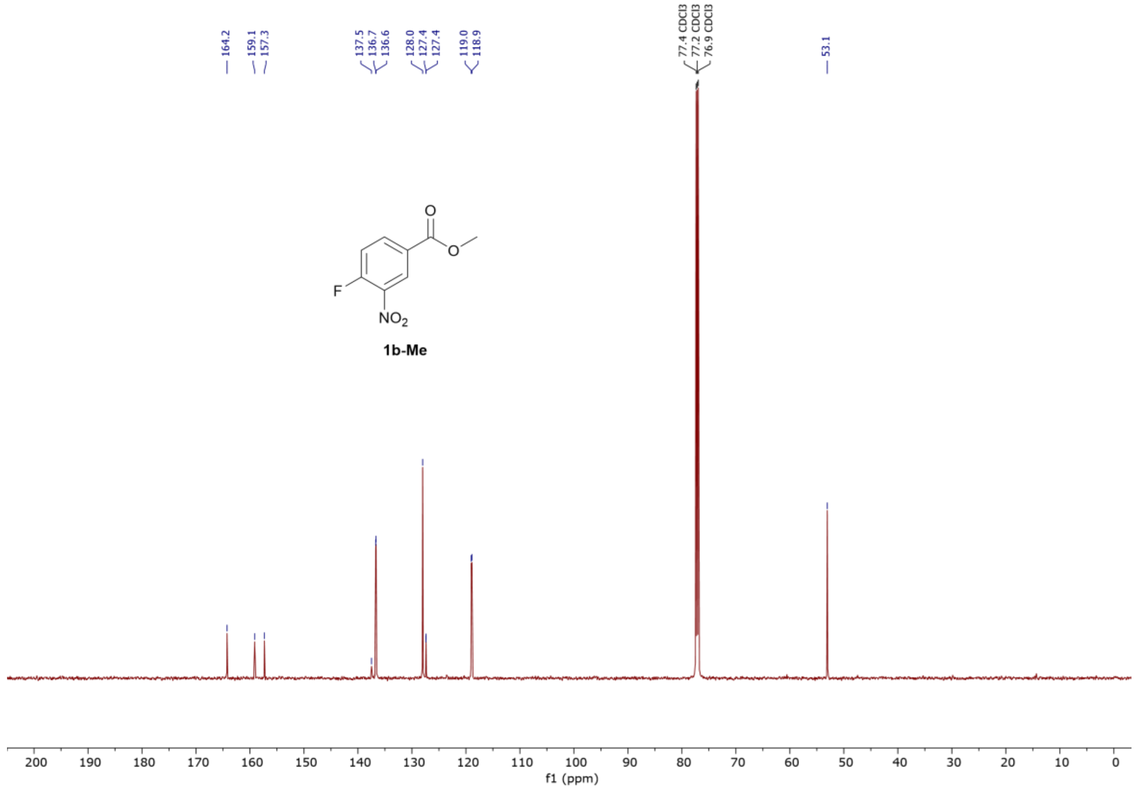

Figure S30. ^{13}C NMR spectrum of compound **1b-Me** (151 MHz, $CDCl_3$).

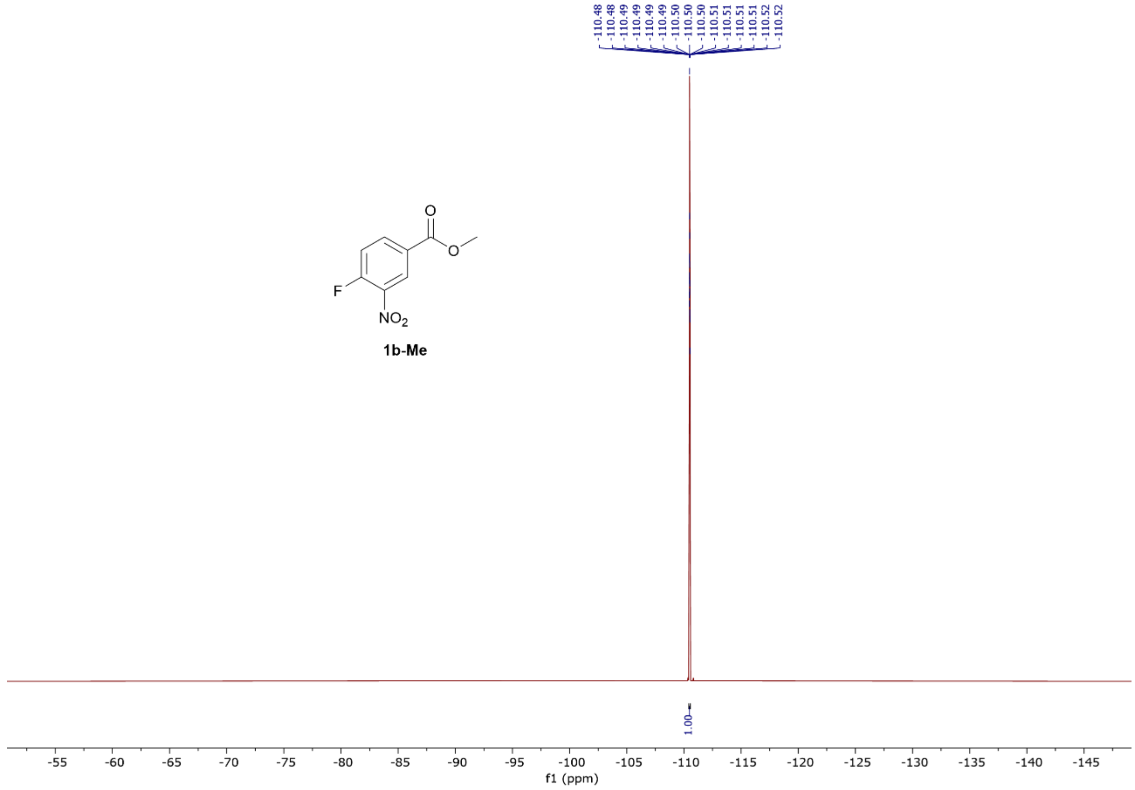

Figure S31. ^{19}F NMR spectrum of compound **1b-Me** (564 MHz, $CDCl_3$).

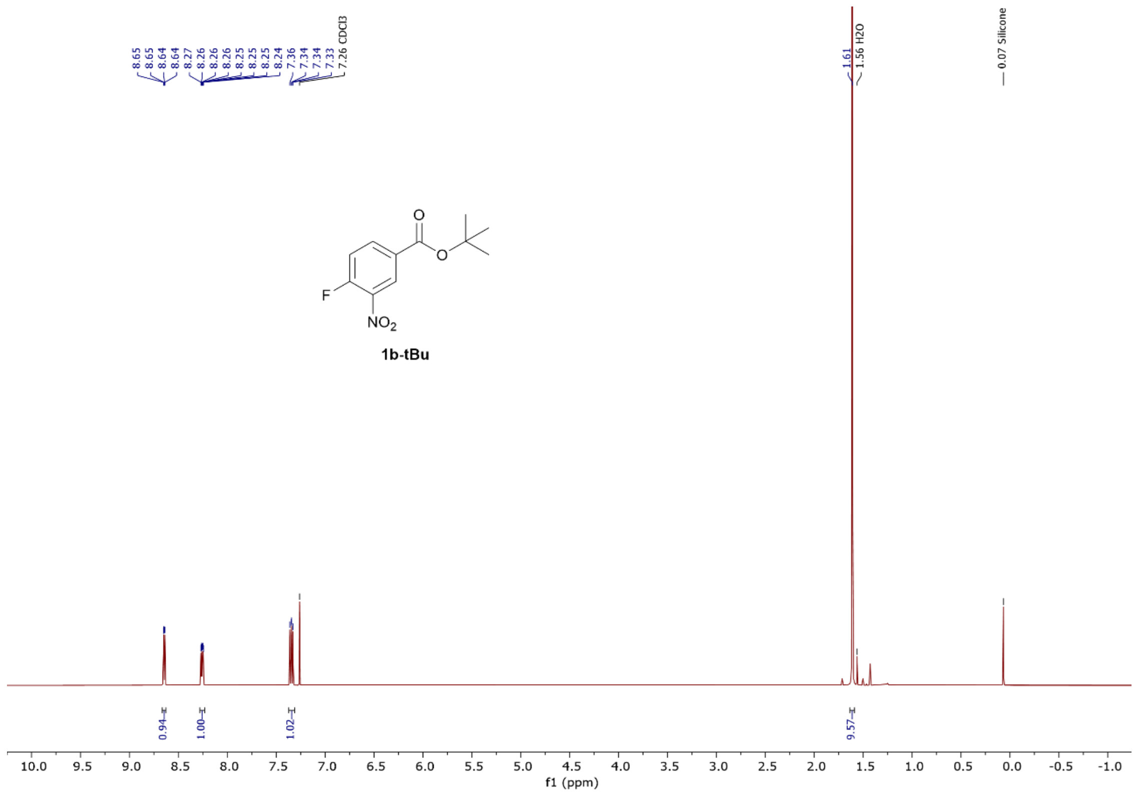

Figure S32. ^{1}H NMR spectrum of compound **1b-tBu** (600 MHz, $CDCl_3$).

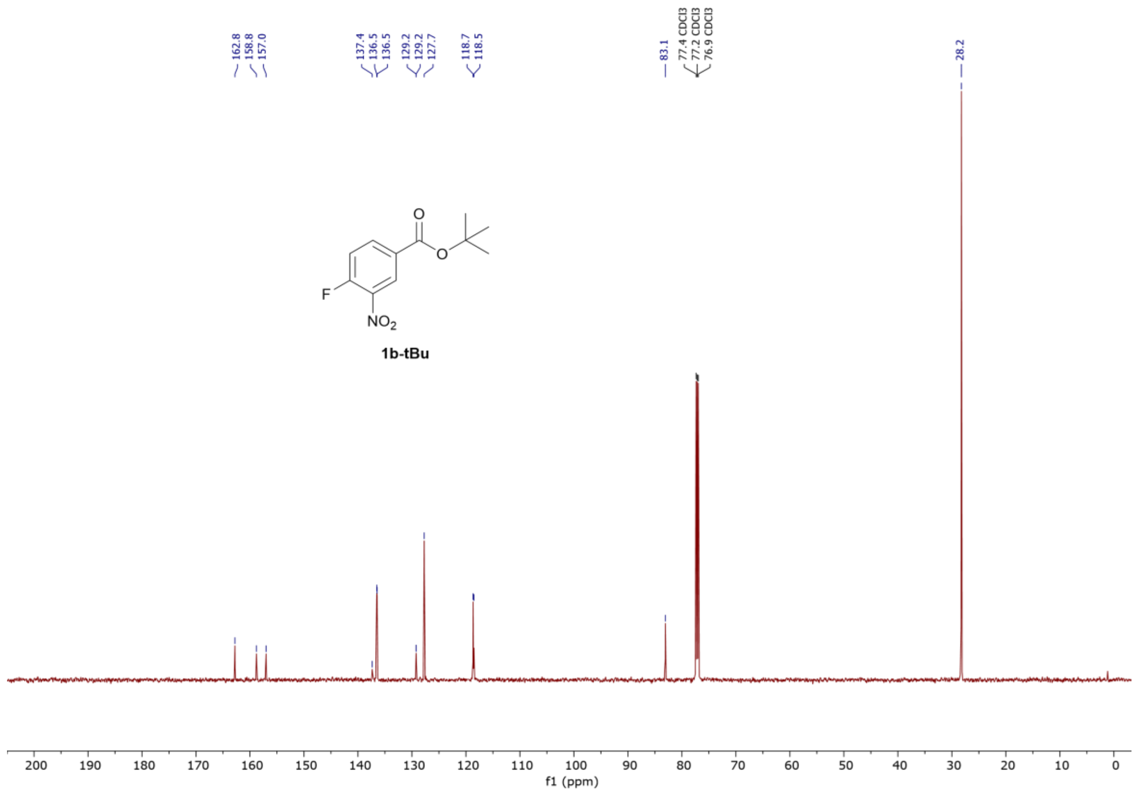

Figure S33. ^{13}C NMR spectrum of compound **1b-tBu** (151 MHz, $CDCl_3$).

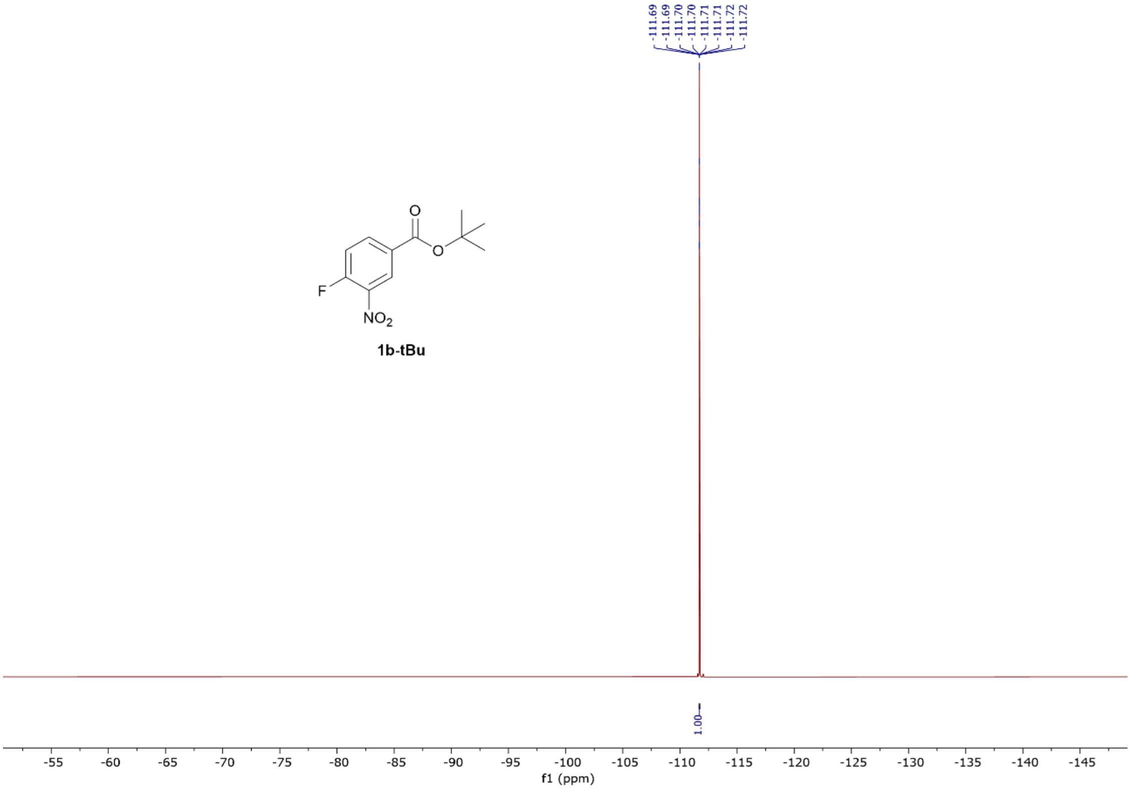

Figure S34. ^{19}F NMR spectrum of compound **1b-tBu** (564 MHz, $CDCl_3$).

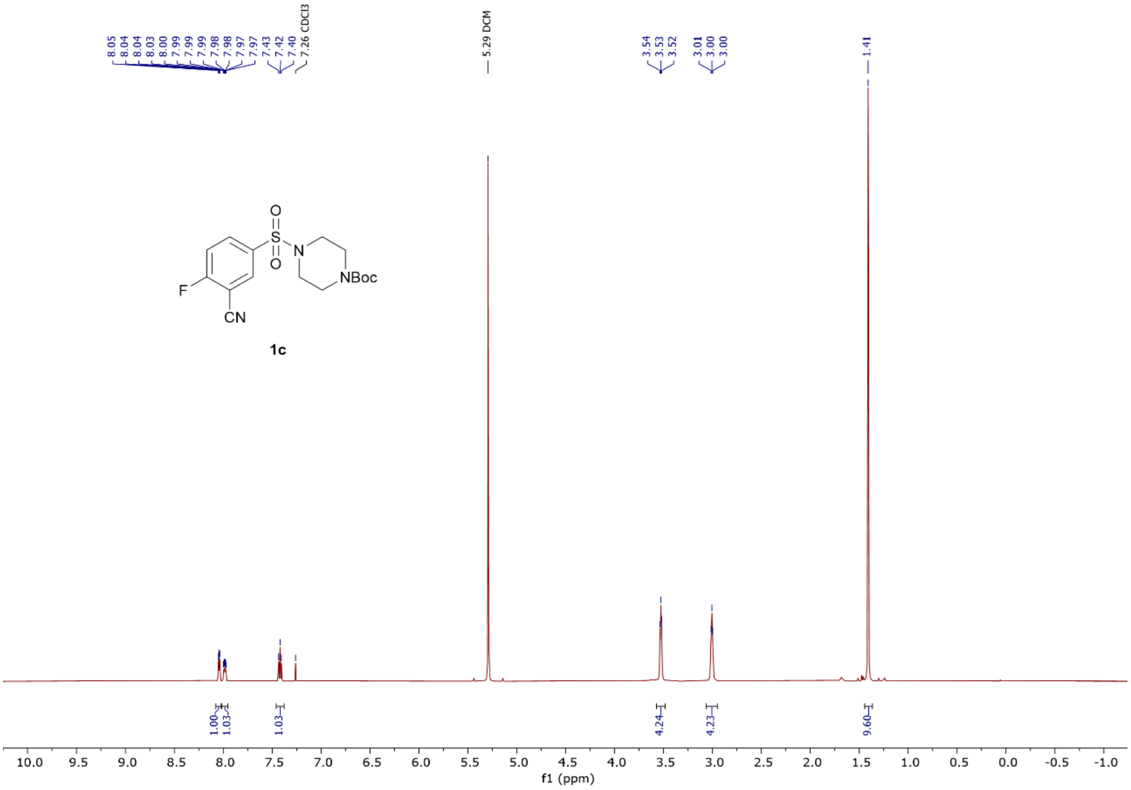

Figure S35. ^{1}H NMR spectrum of compound **1c** (600 MHz, $CDCl_3$).

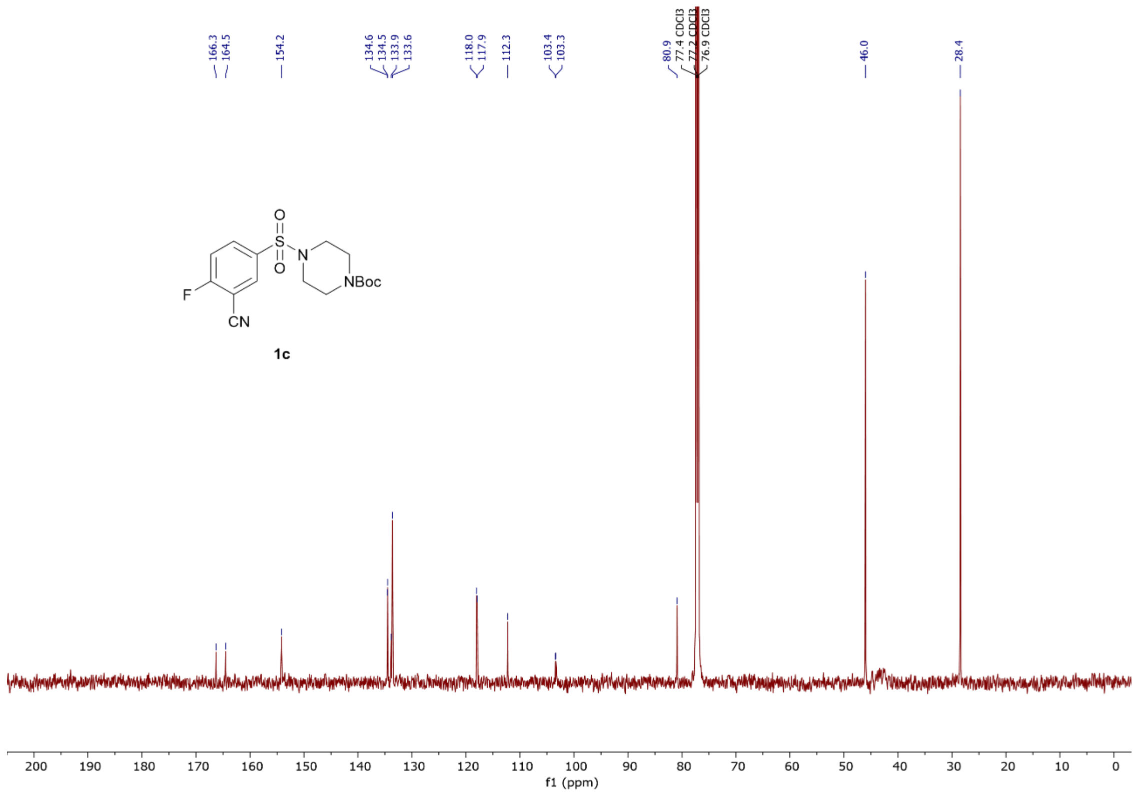

Figure S36. ^{13}C NMR spectrum of compound **1c** (151 MHz, $CDCl_3$).

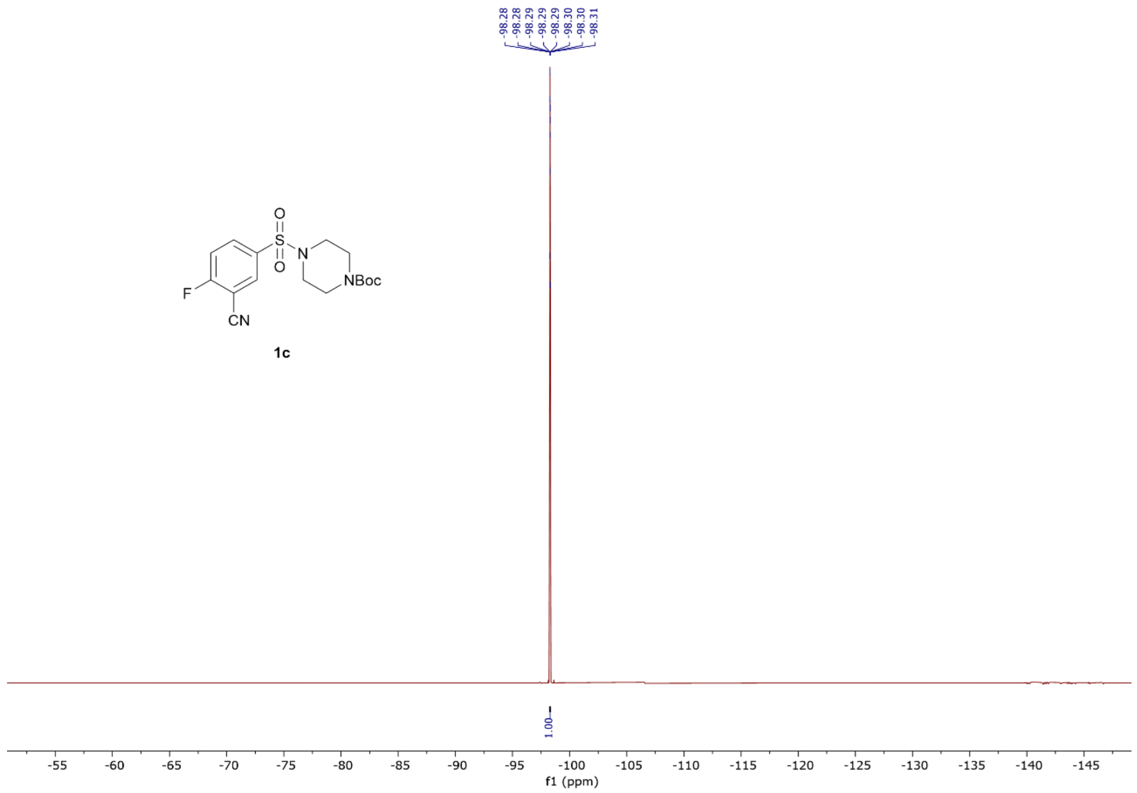

Figure S37. ^{19}F NMR spectrum of compound **1c** (564 MHz, $CDCl_3$).

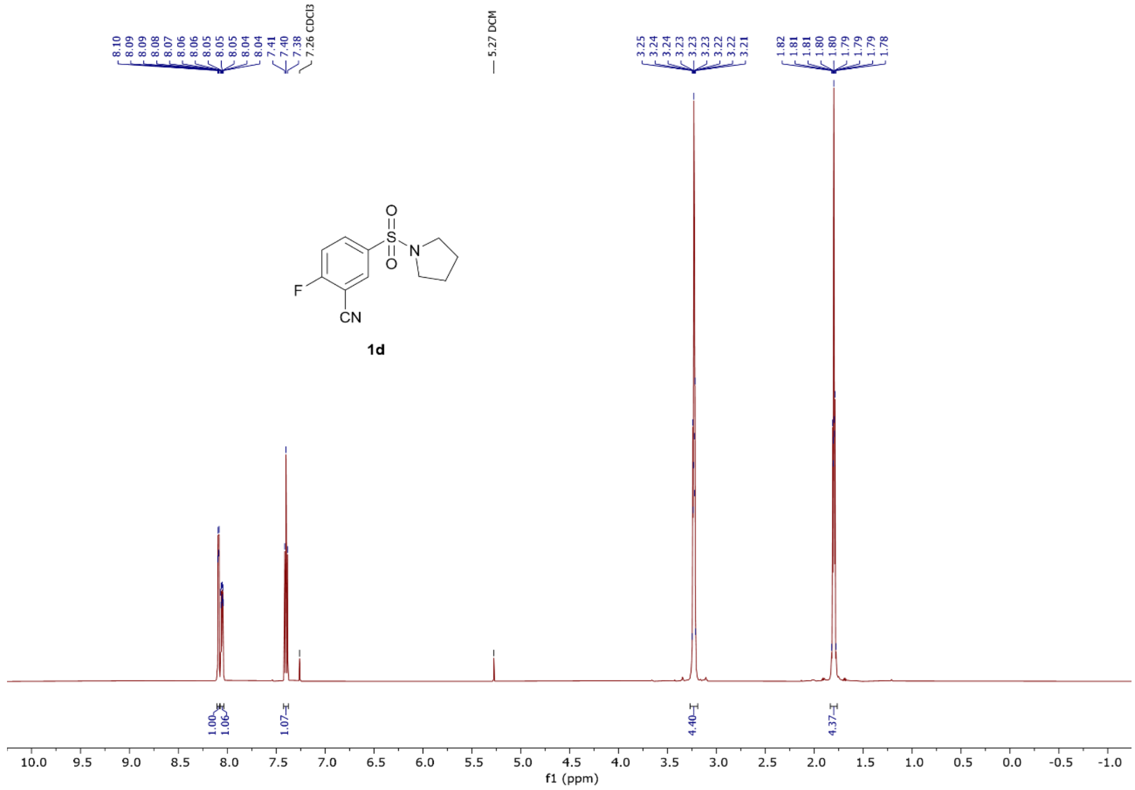

Figure S38. ^{1}H NMR spectrum of compound **1d** (600 MHz, $CDCl_3$).

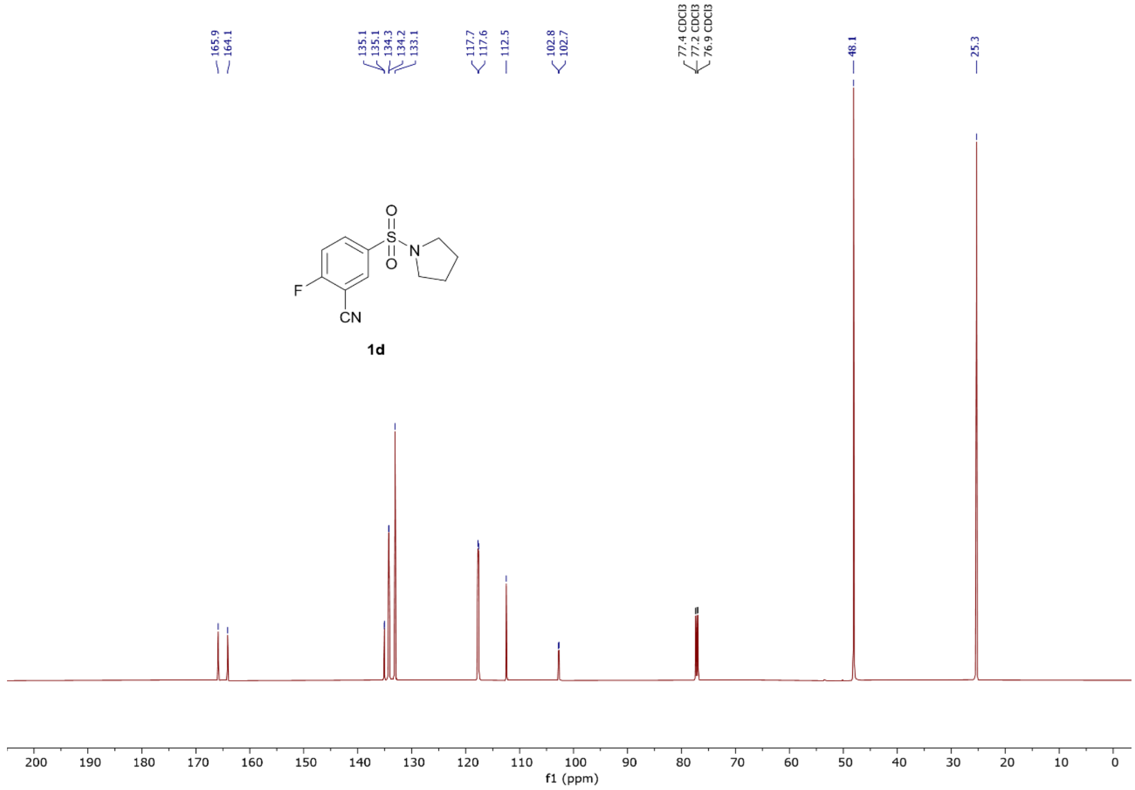

Figure S39. ^{13}C NMR spectrum of compound **1d** (151 MHz, $CDCl_3$).

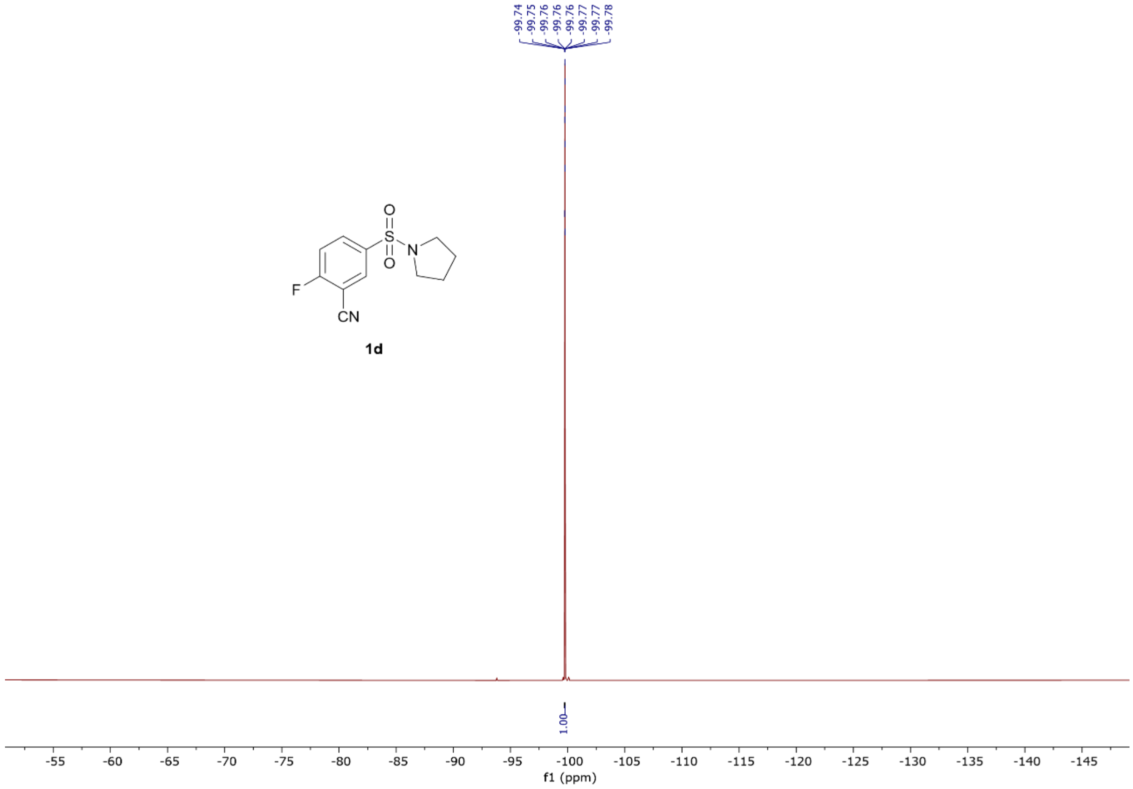

Figure S40. ^{19}F NMR spectrum of compound **1d** (564 MHz, $CDCl_3$).

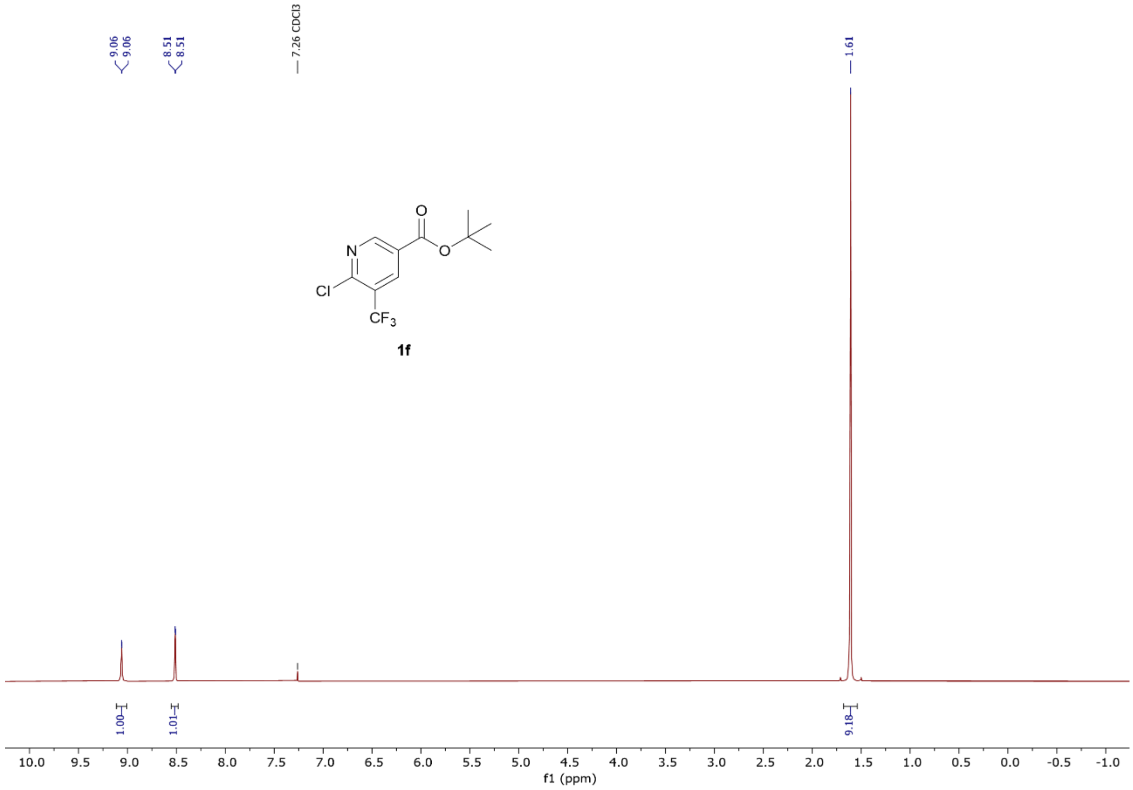

Figure S41. ^{1}H NMR spectrum of compound **1f** (600 MHz, $CDCl_3$).

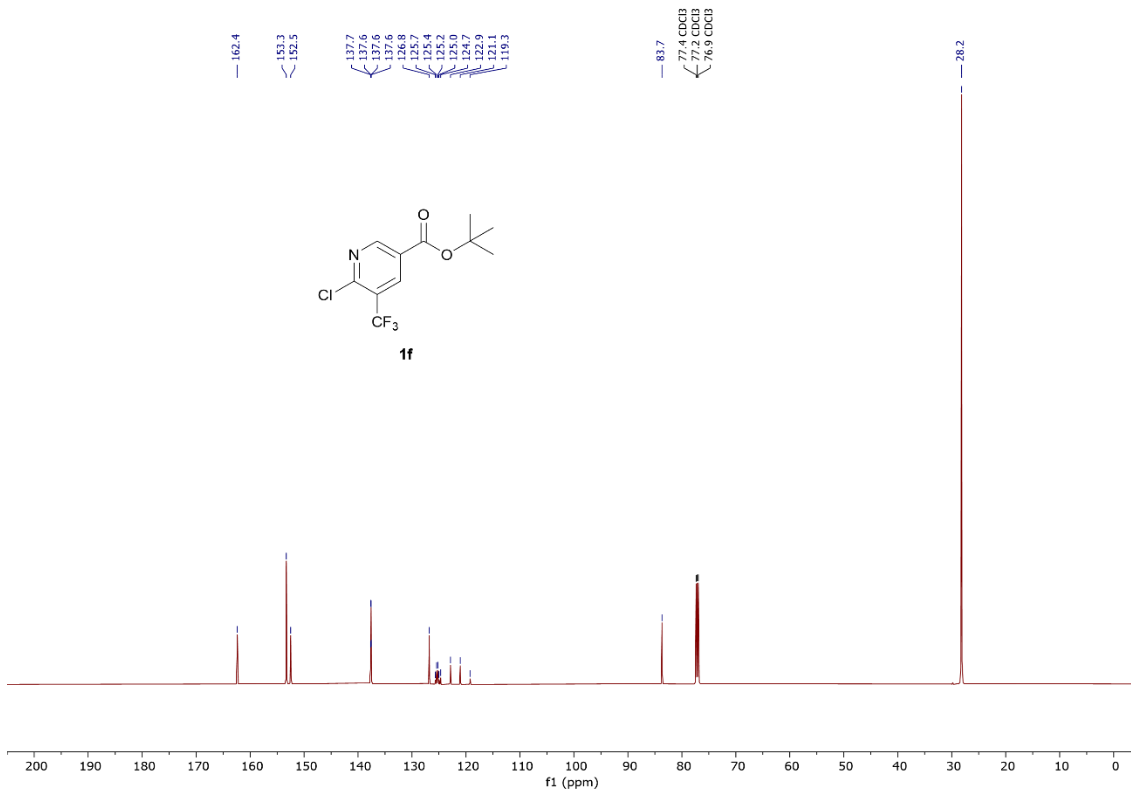

Figure S42. ^{13}C NMR spectrum of compound **1f** (151 MHz, $CDCl_3$).

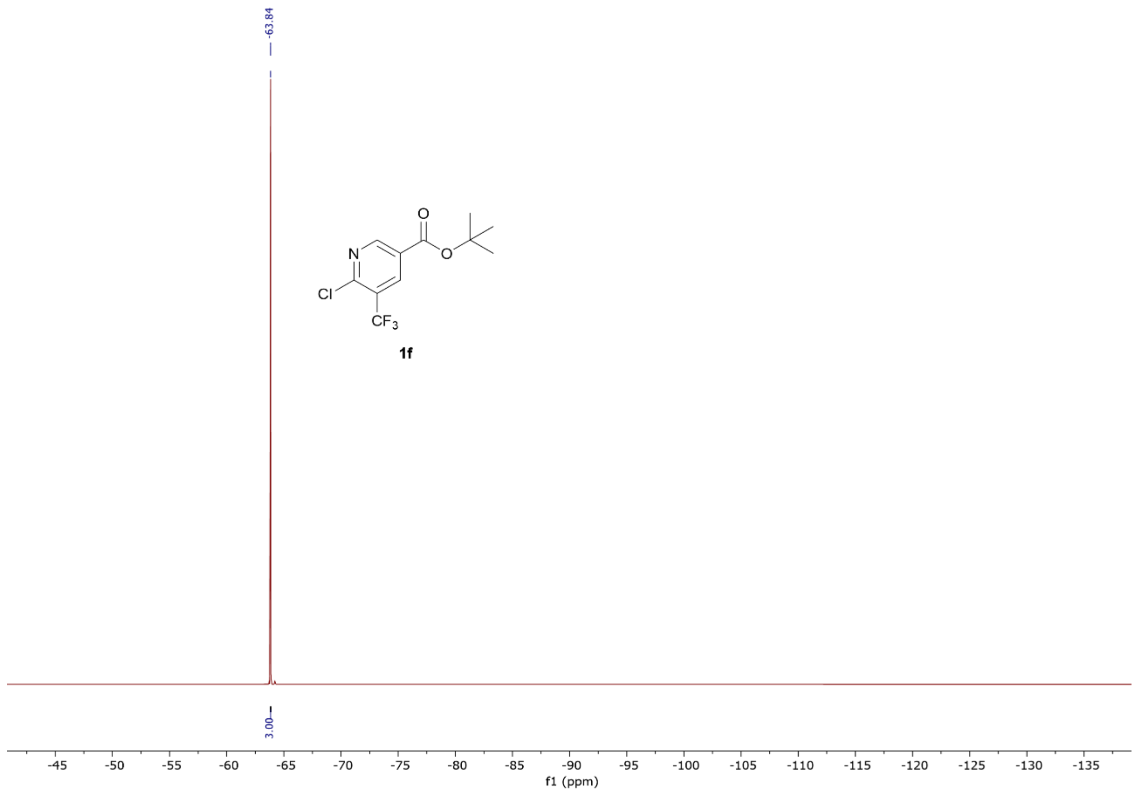

Figure S43. ^{19}F NMR spectrum of compound **1f** (564 MHz, $CDCl_3$).

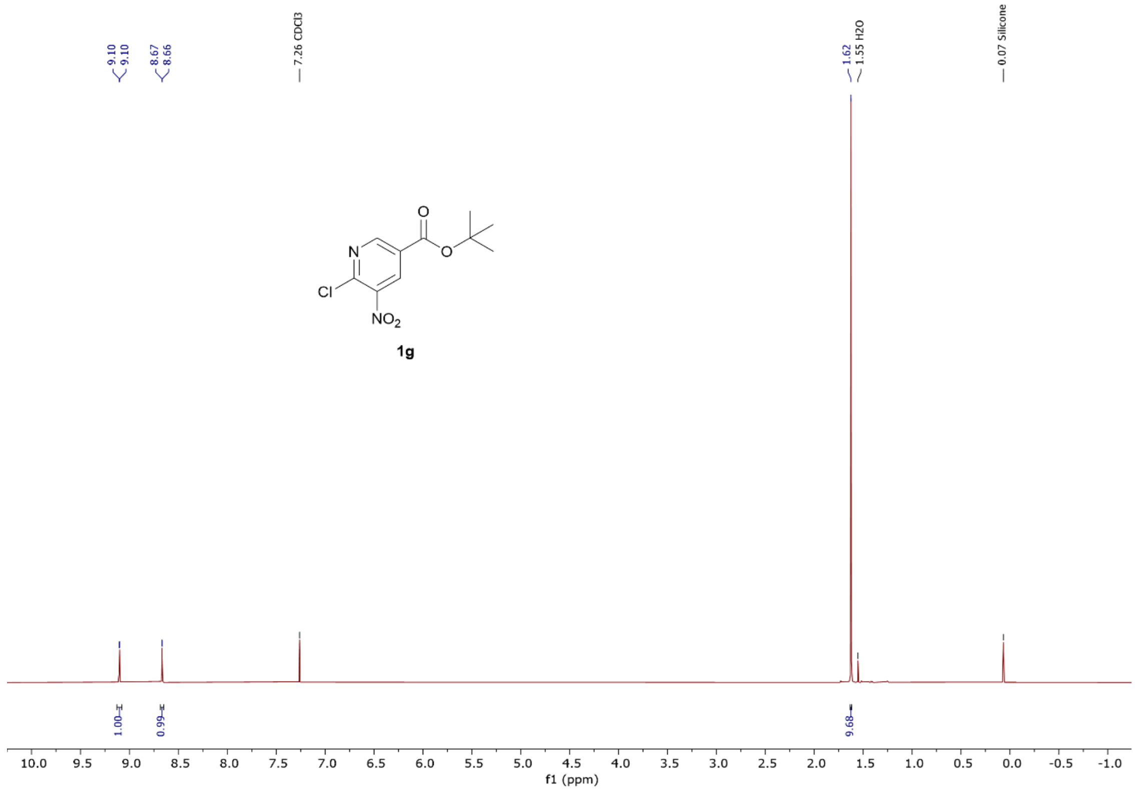

Figure S44. ^{1}H NMR spectrum of compound **1g** (600 MHz, $CDCl_3$).

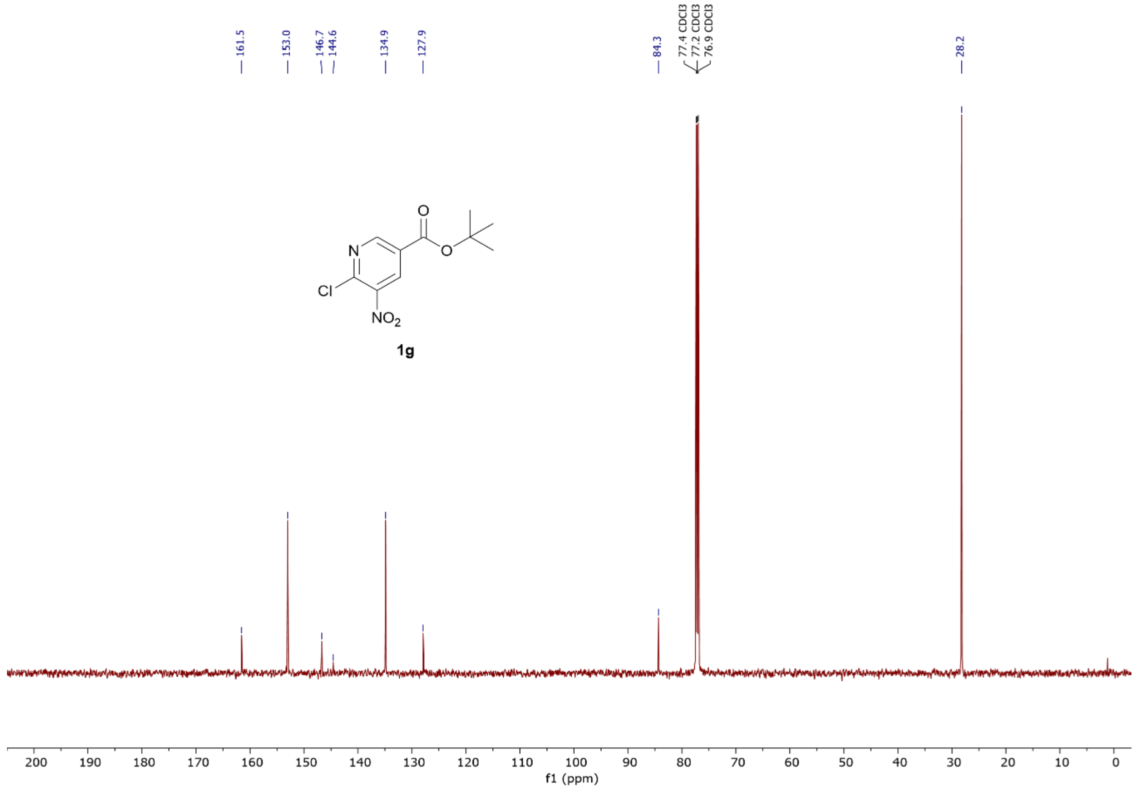

Figure S45. ^{13}C NMR spectrum of compound **1g** (151 MHz, $CDCl_3$).

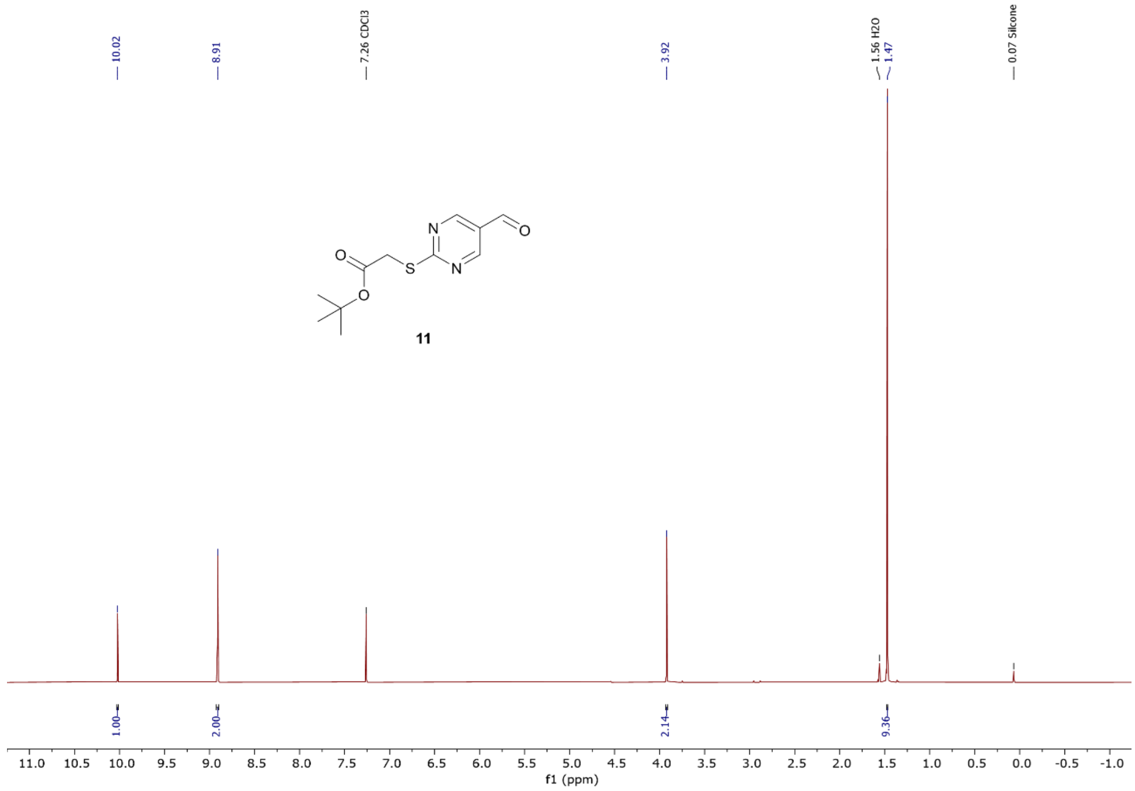

Figure S46. ^{1}H NMR spectrum of compound **11** (600 MHz, $CDCl_3$).

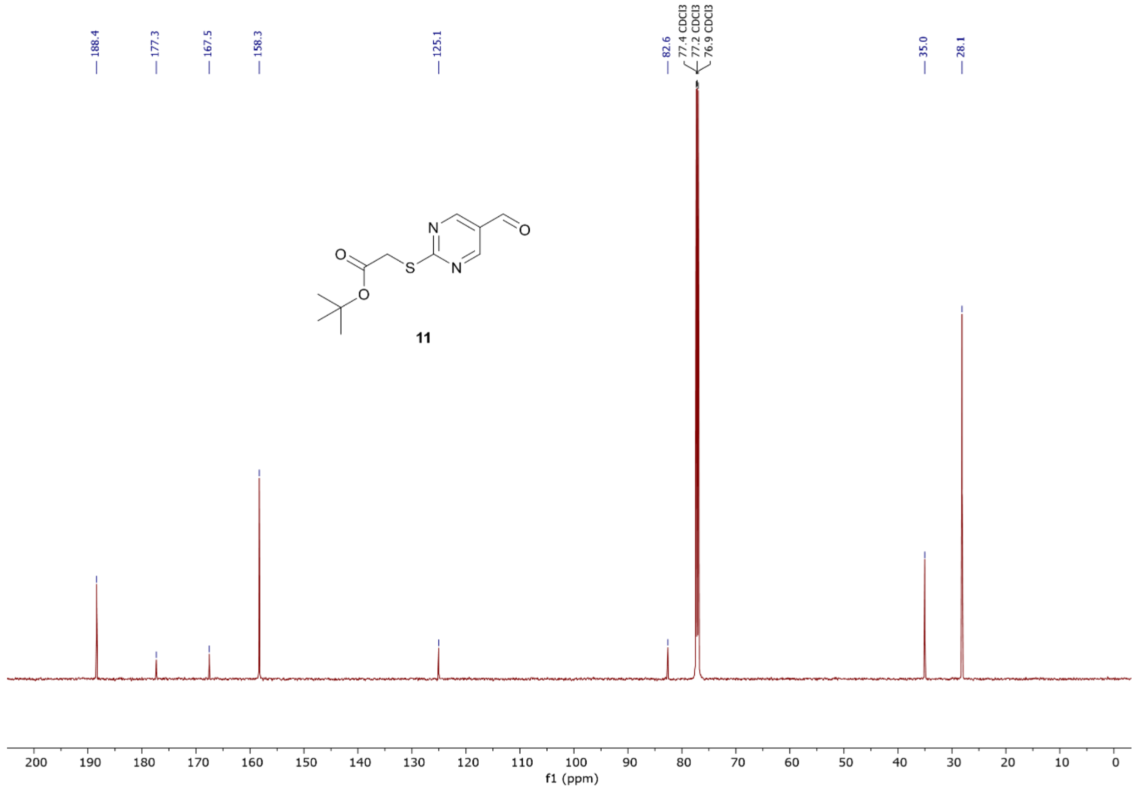

Figure S47. ^{13}C NMR spectrum of compound **11** (151 MHz, $CDCl_3$).

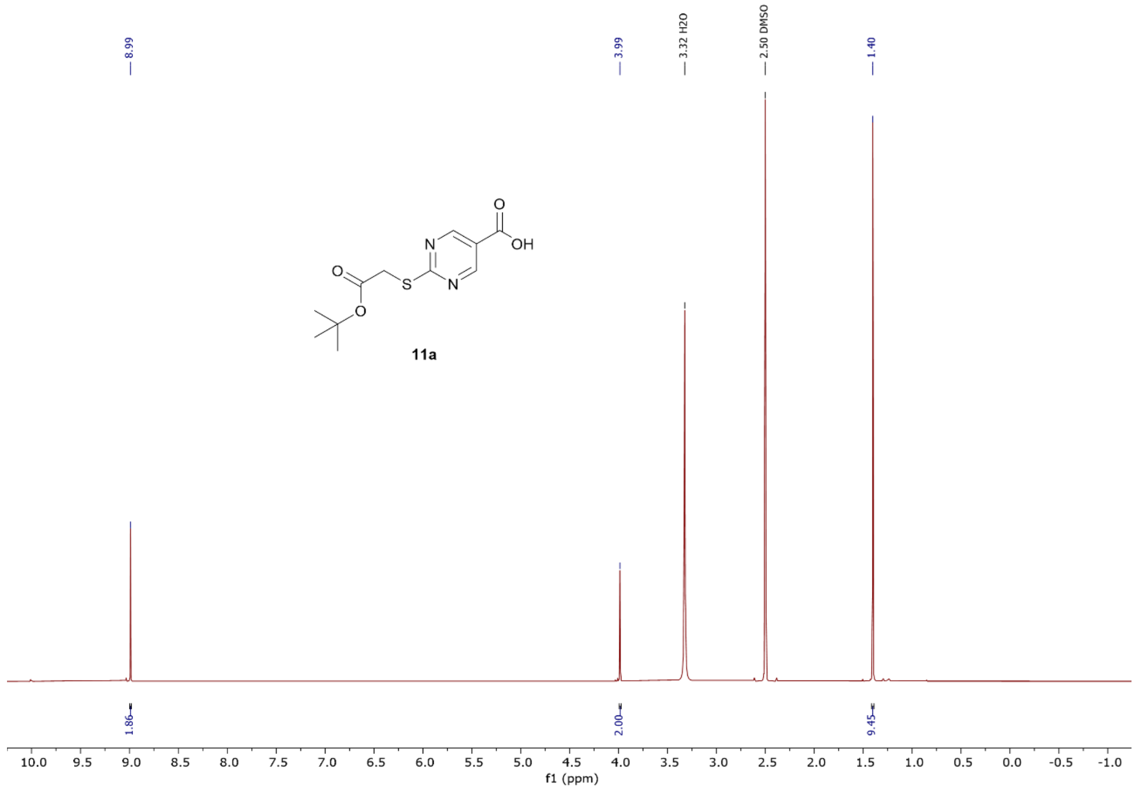

Figure S48. ^{1}H NMR spectrum of compound **11a** (600 MHz, $(CD_3)_2SO$).

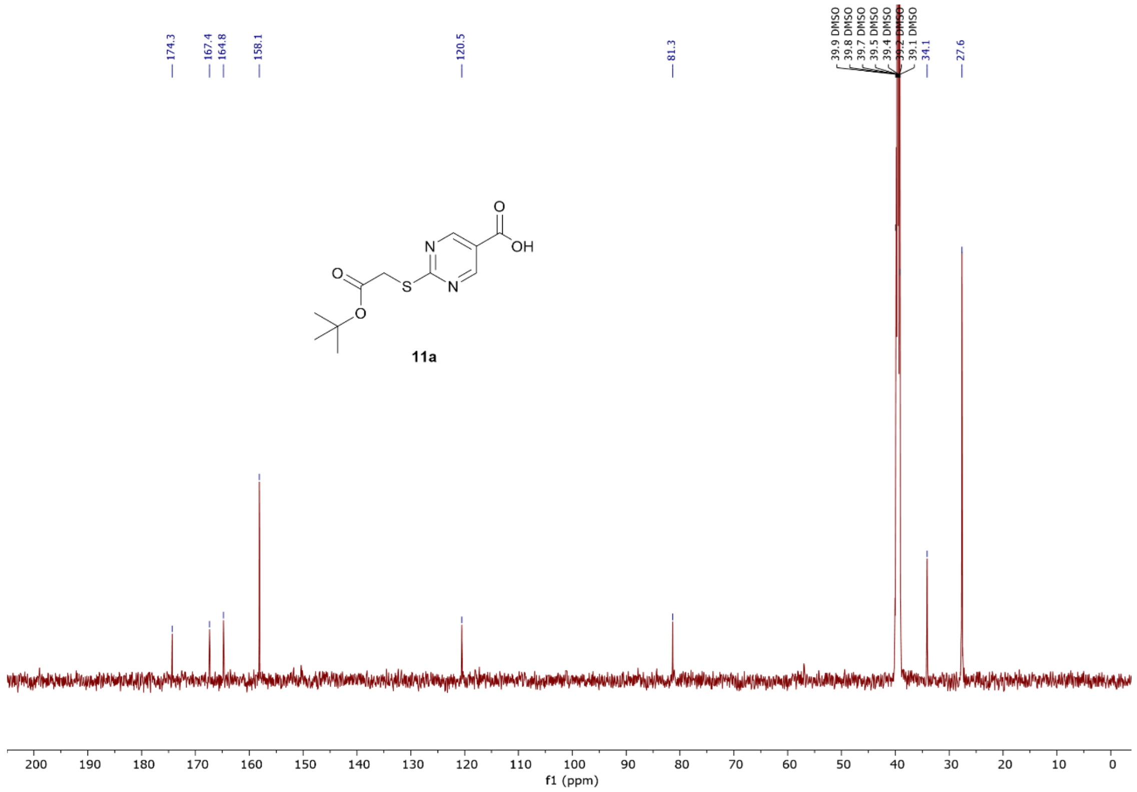

Figure S49. ^{13}C NMR spectrum of compound **11a** (151 MHz, $(CD_3)_2SO$).

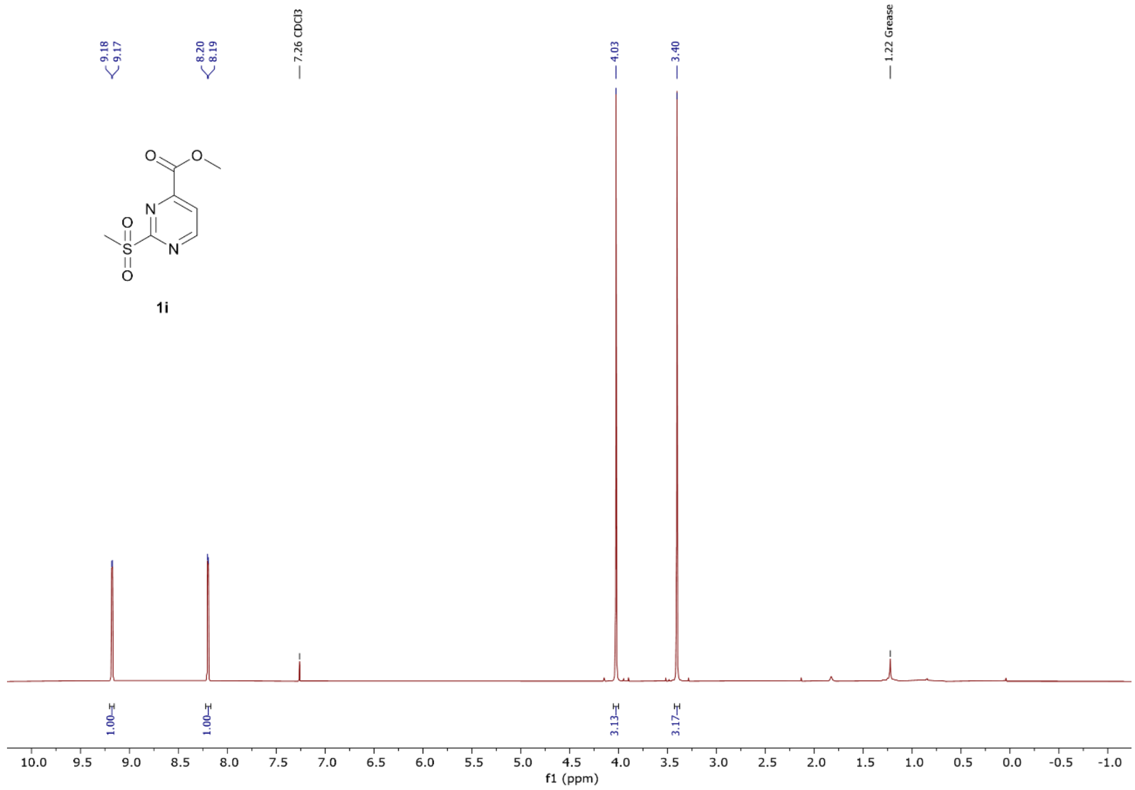

Figure S50. ^{1}H NMR spectrum of compound **1i** (600 MHz, $CDCl_3$).

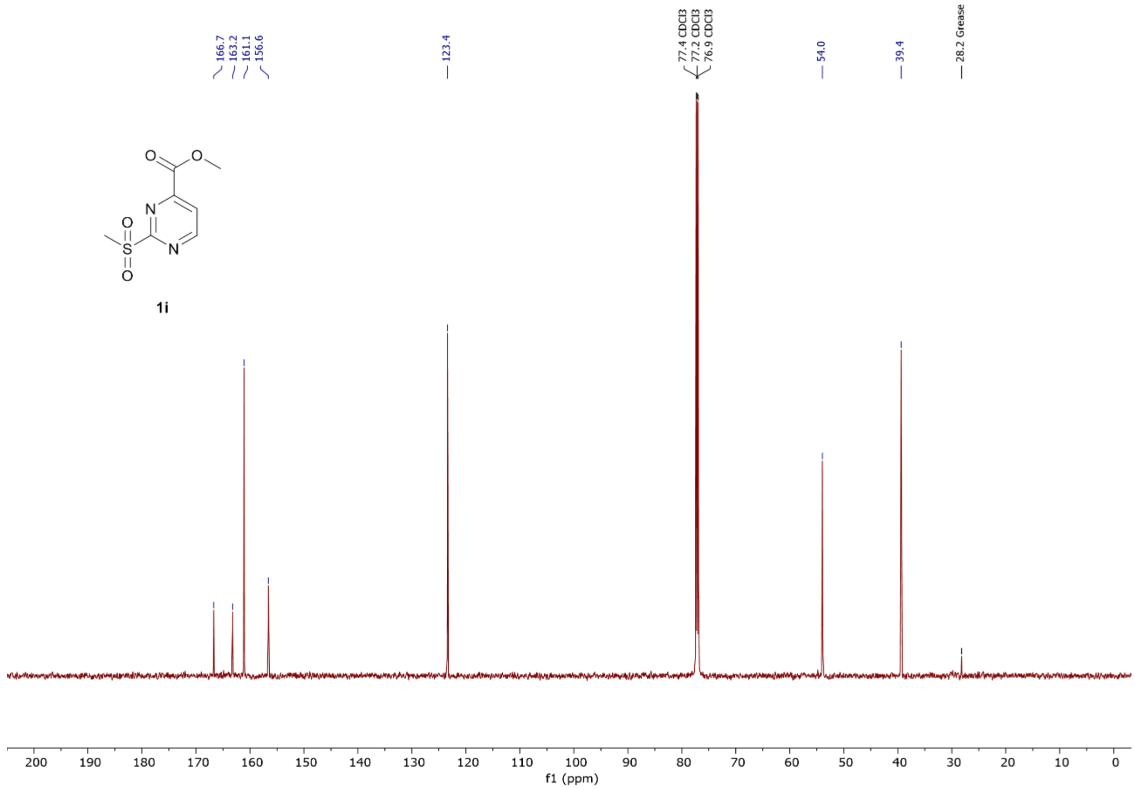

Figure S51. ^{13}C NMR spectrum of compound **1i** (151 MHz, $CDCl_3$).

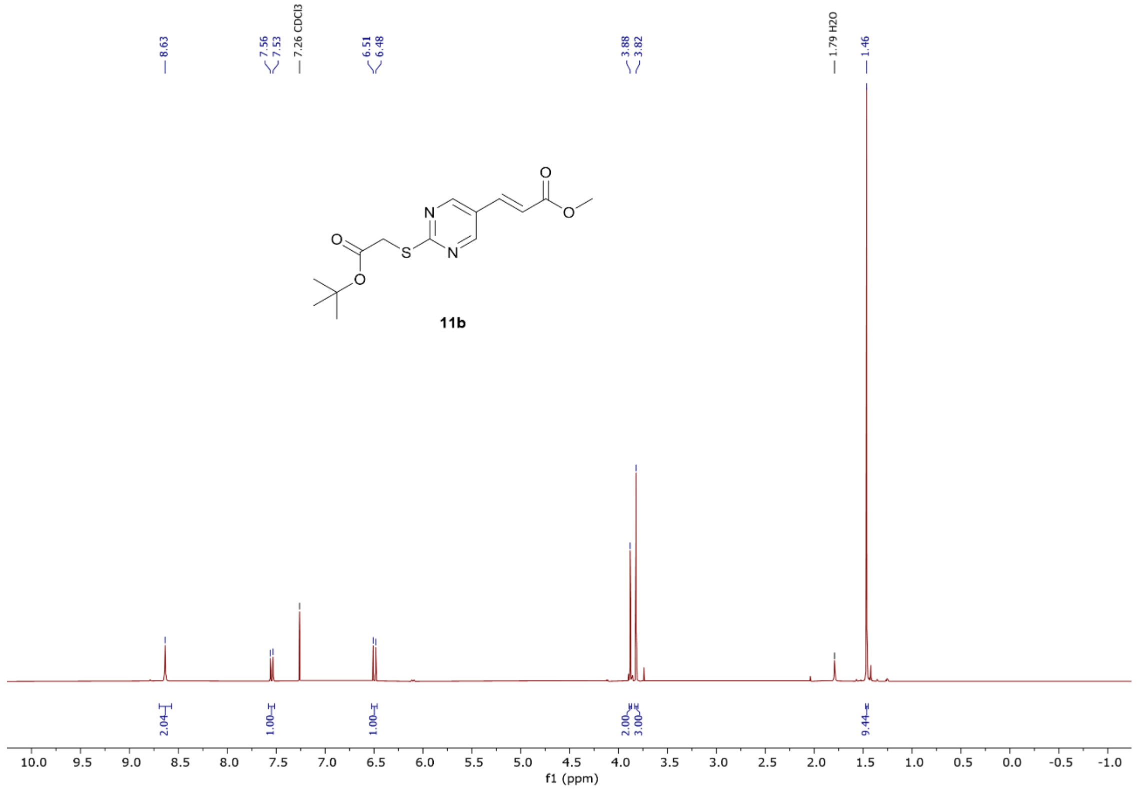

Figure S52. ^{1}H NMR spectrum of compound **11b** (600 MHz, $CDCl_3$).

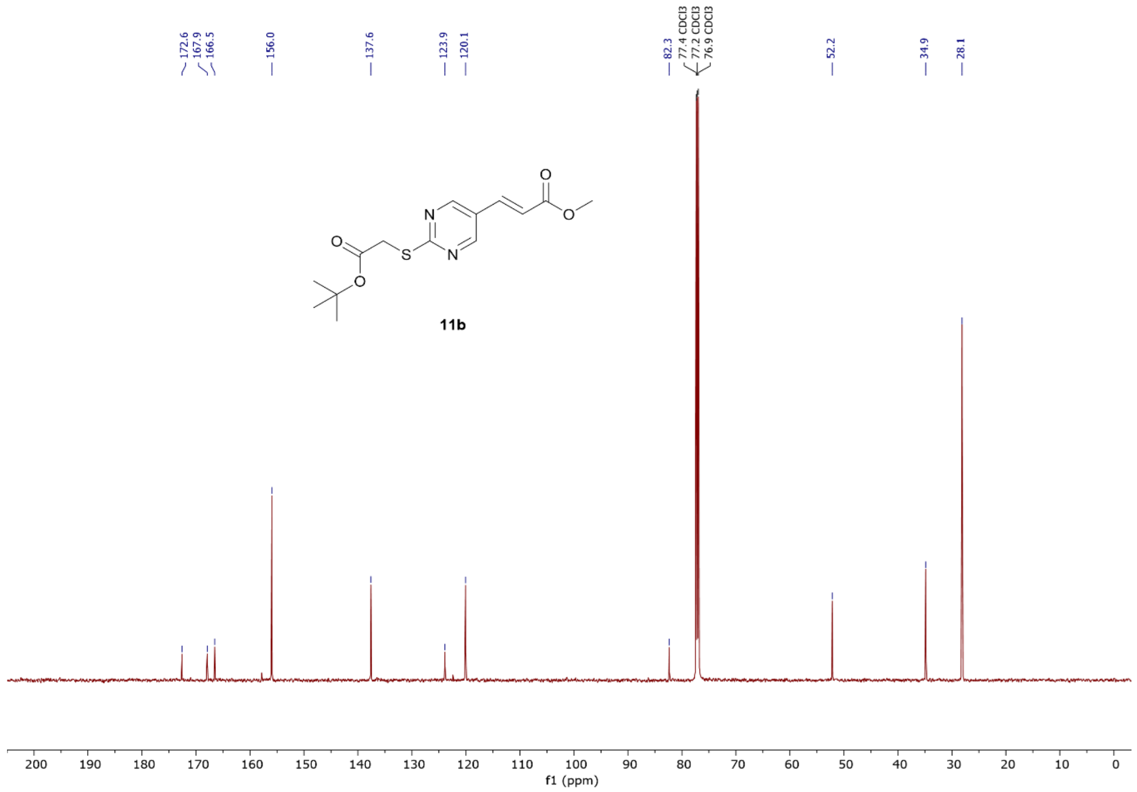

Figure S53. ^{13}C NMR spectrum of compound **11b** (151 MHz, $CDCl_3$).

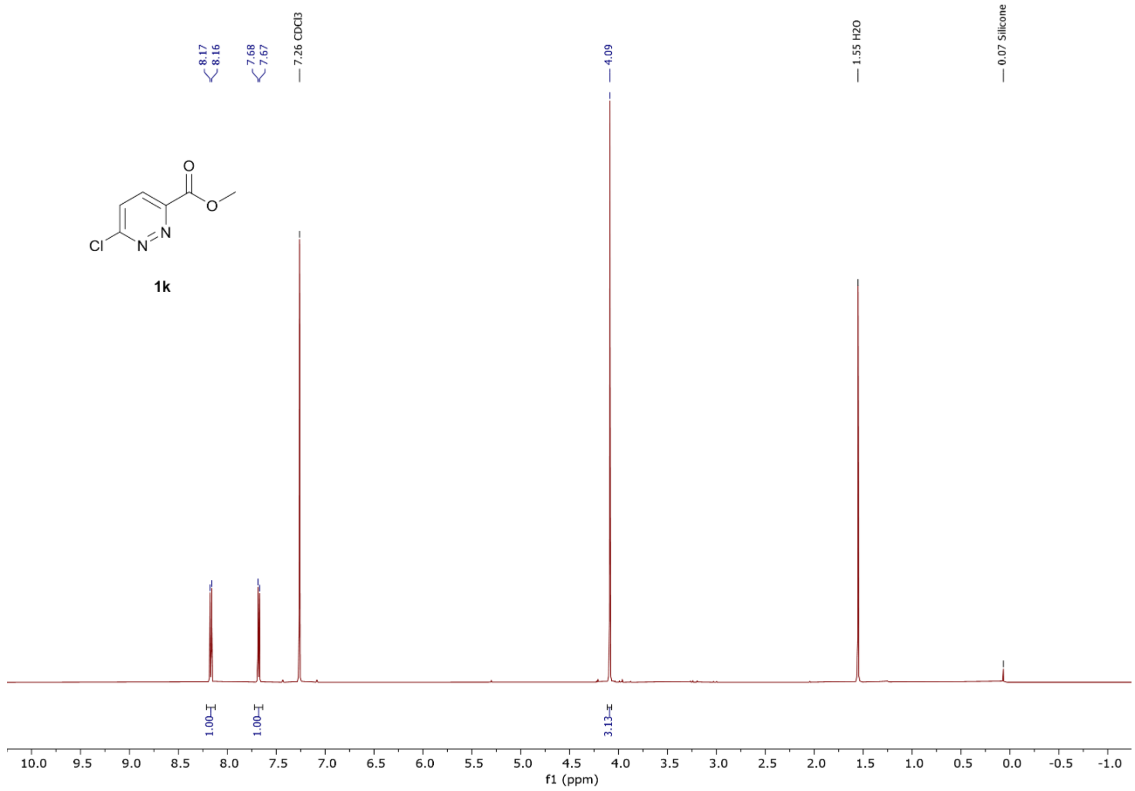

Figure S54. ^{1}H NMR spectrum of compound **1k** (600 MHz, $CDCl_3$).

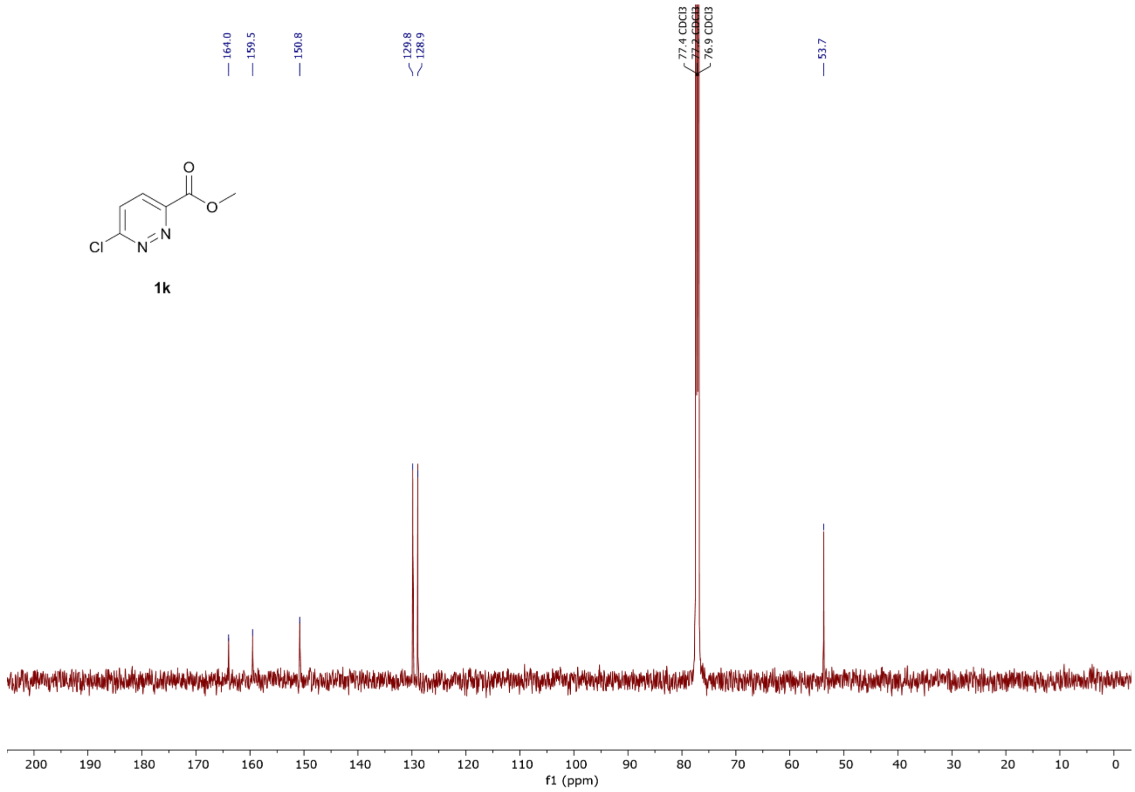

Figure S55. ^{13}C NMR spectrum of compound **1k** (151 MHz, $CDCl_3$).

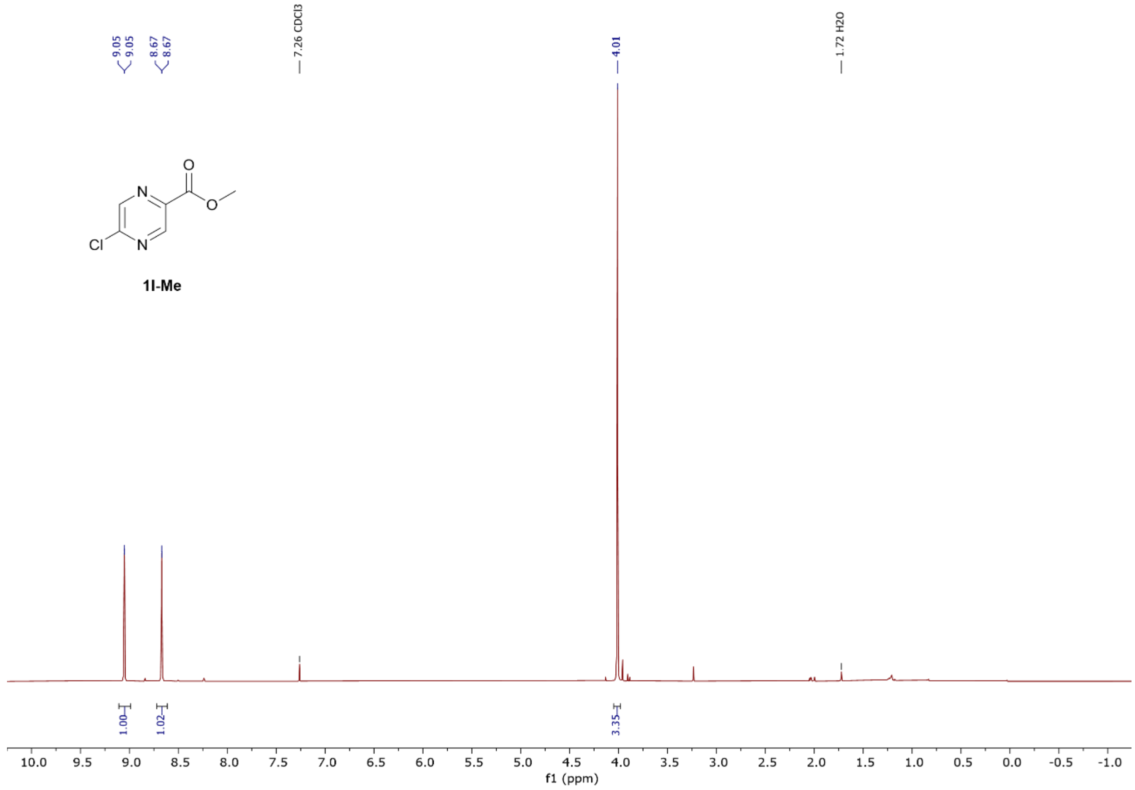

Figure S56. ^{1}H NMR spectrum of compound **1l-Me** (600 MHz, $CDCl_3$).

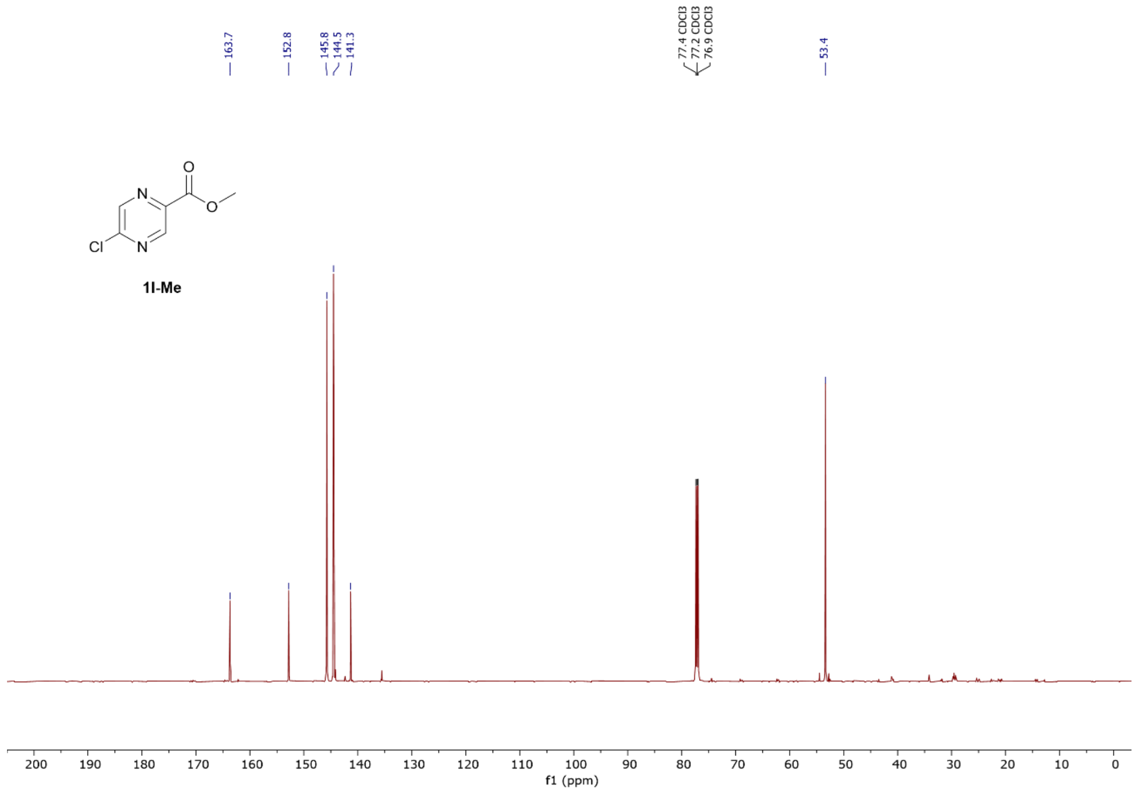

Figure S57. ^{13}C NMR spectrum of compound **1l-Me** (151 MHz, $CDCl_3$).

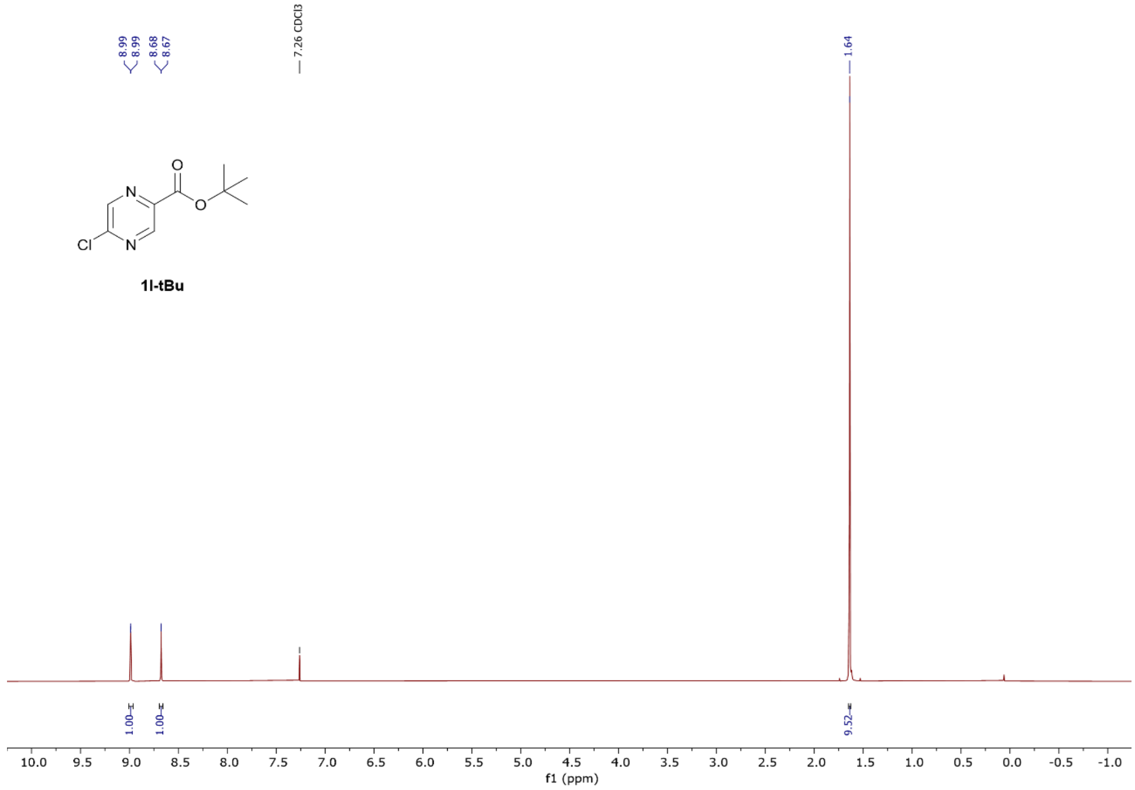

1l-tBu

Figure S58. ^{1}H NMR spectrum of compound **1l-tBu** (600 MHz, $CDCl_3$).

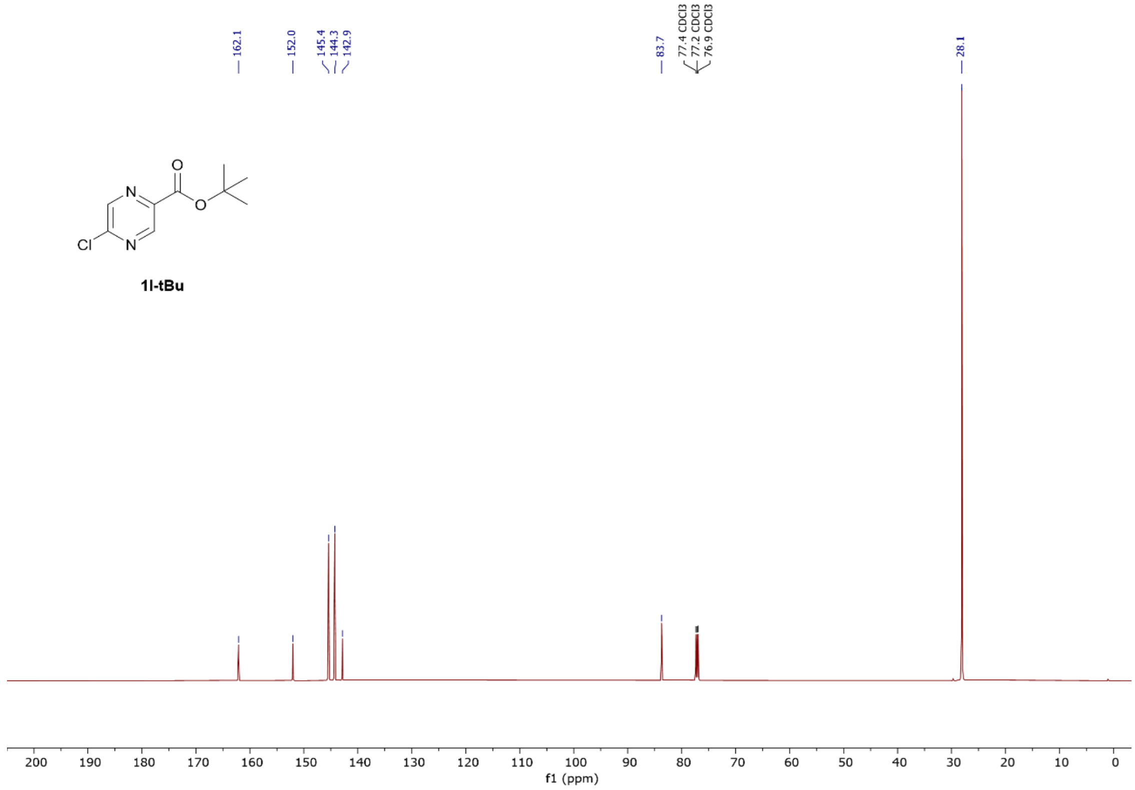

1l-tBu

Figure S59. ^{13}C NMR spectrum of compound **1l-tBu** (151 MHz, $CDCl_3$).

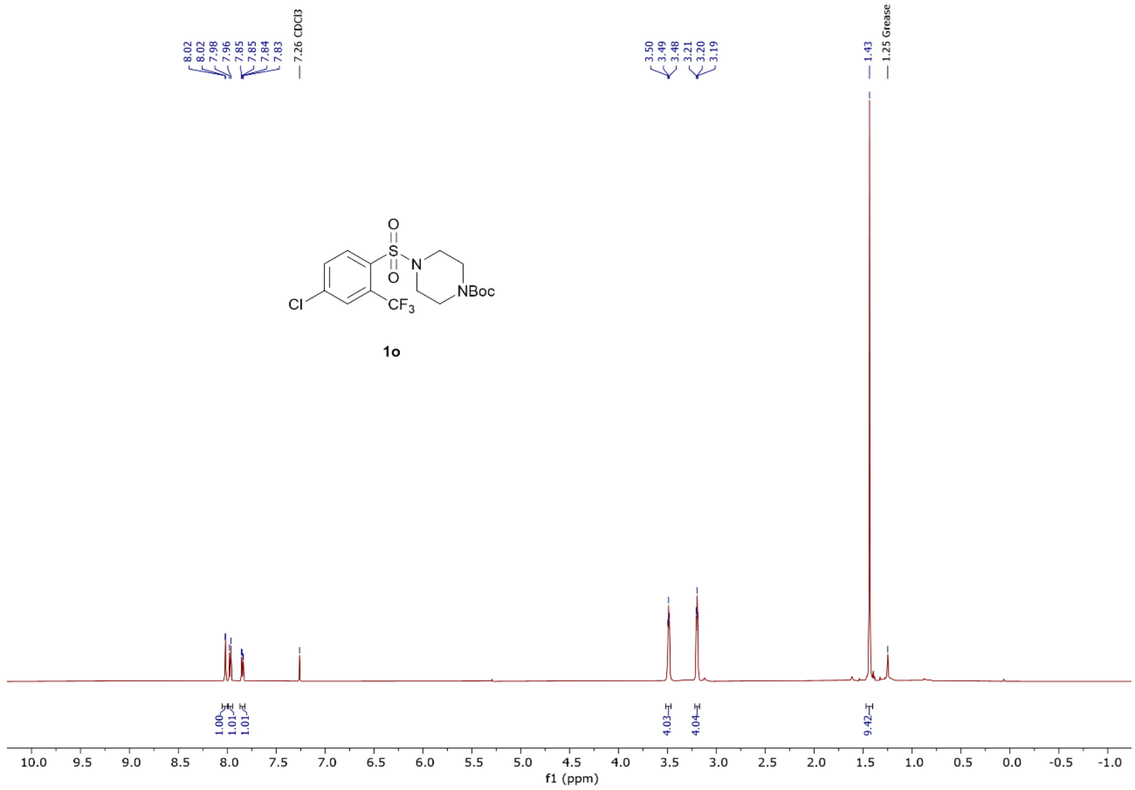

Figure S60. ^{1}H NMR spectrum of compound **1o** (600 MHz, $CDCl_3$).

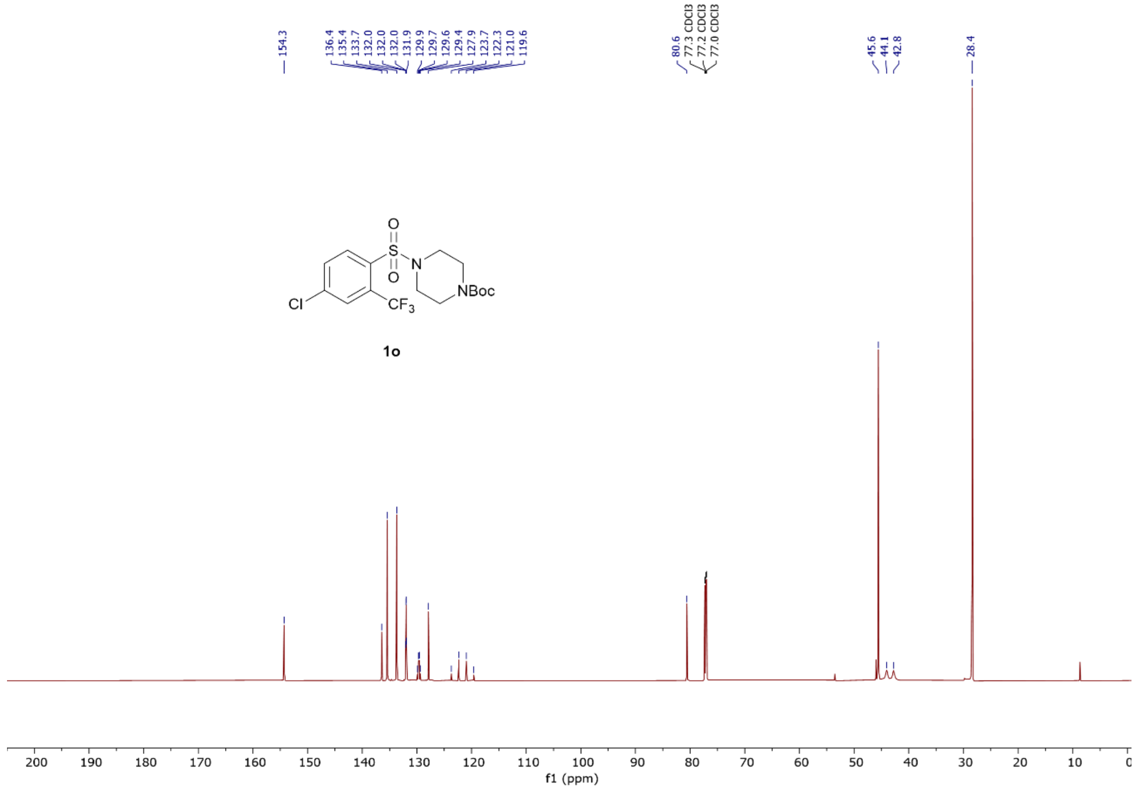

Figure S61. ^{13}C NMR spectrum of compound **1o** (201 MHz, $CDCl_3$).

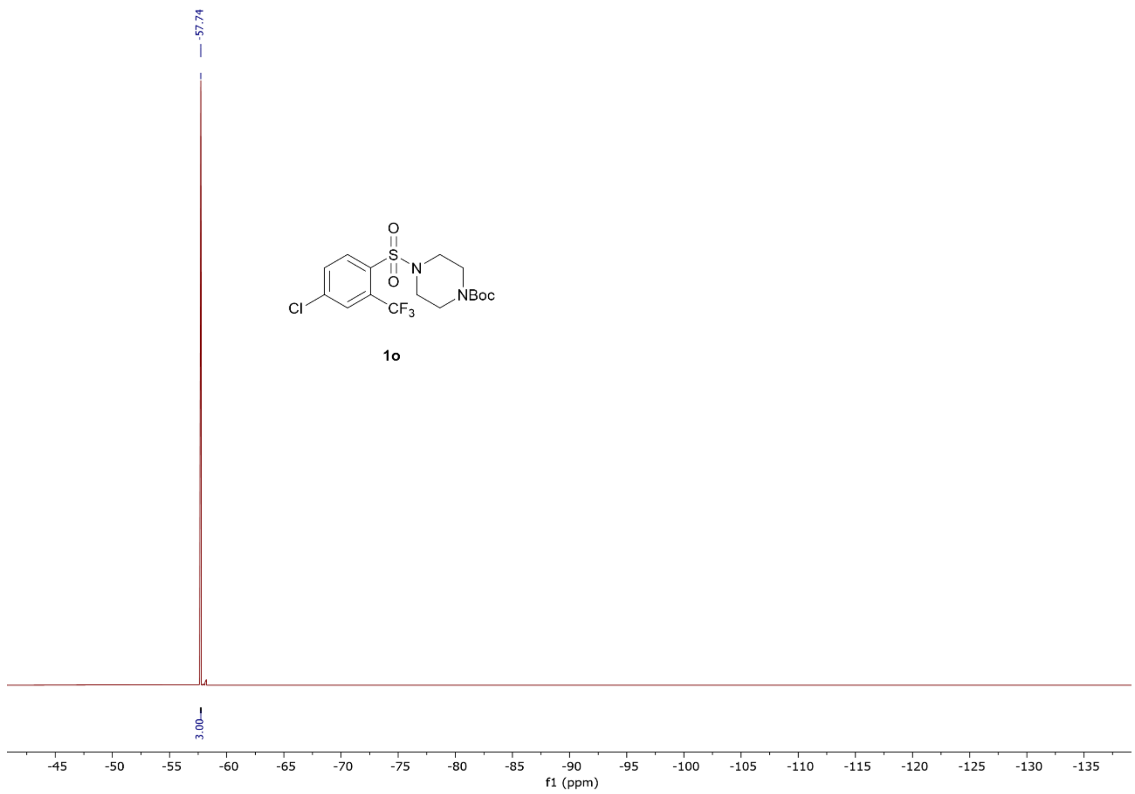

Figure S62. ^{19}F NMR spectrum of compound **1o** (564 MHz, $CDCl_3$).

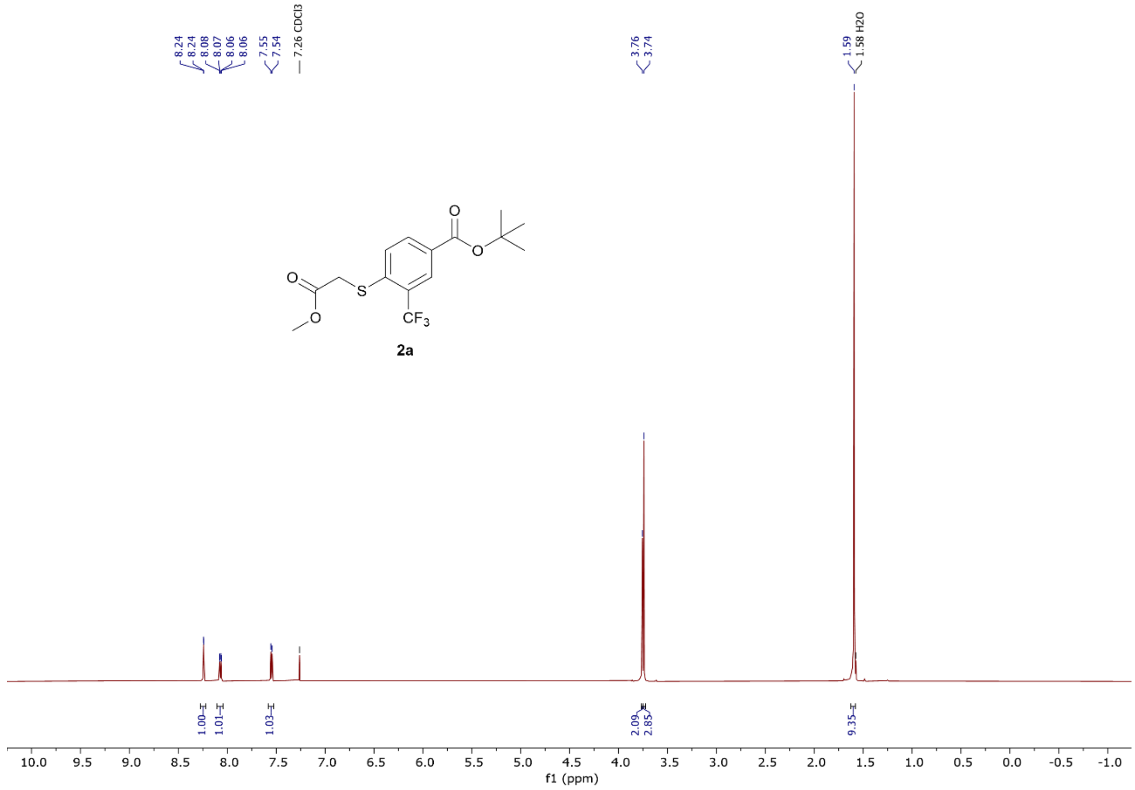

Figure S63. ^{1}H NMR spectrum of compound **2a** (600 MHz, $CDCl_3$).

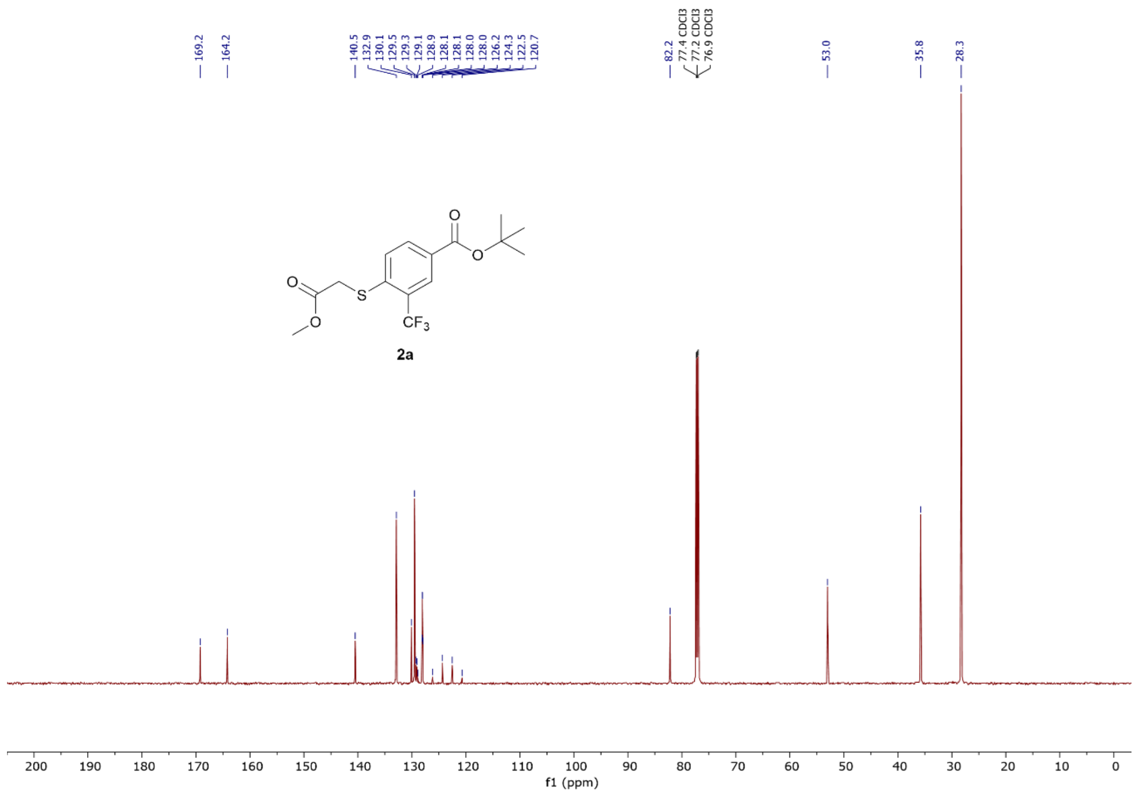

Figure S64. ^{13}C NMR spectrum of compound **2a** (151 MHz, $CDCl_3$).

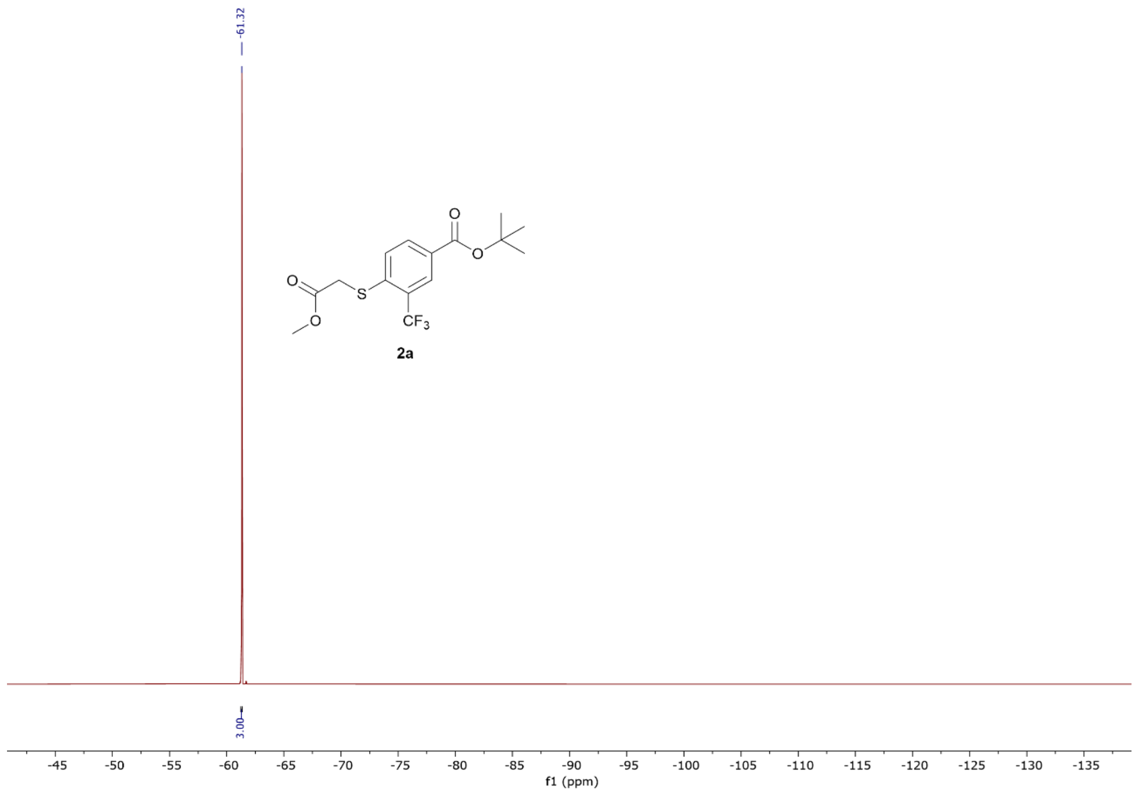

Figure S65. ^{19}F NMR spectrum of compound **2a** (564 MHz, $CDCl_3$).

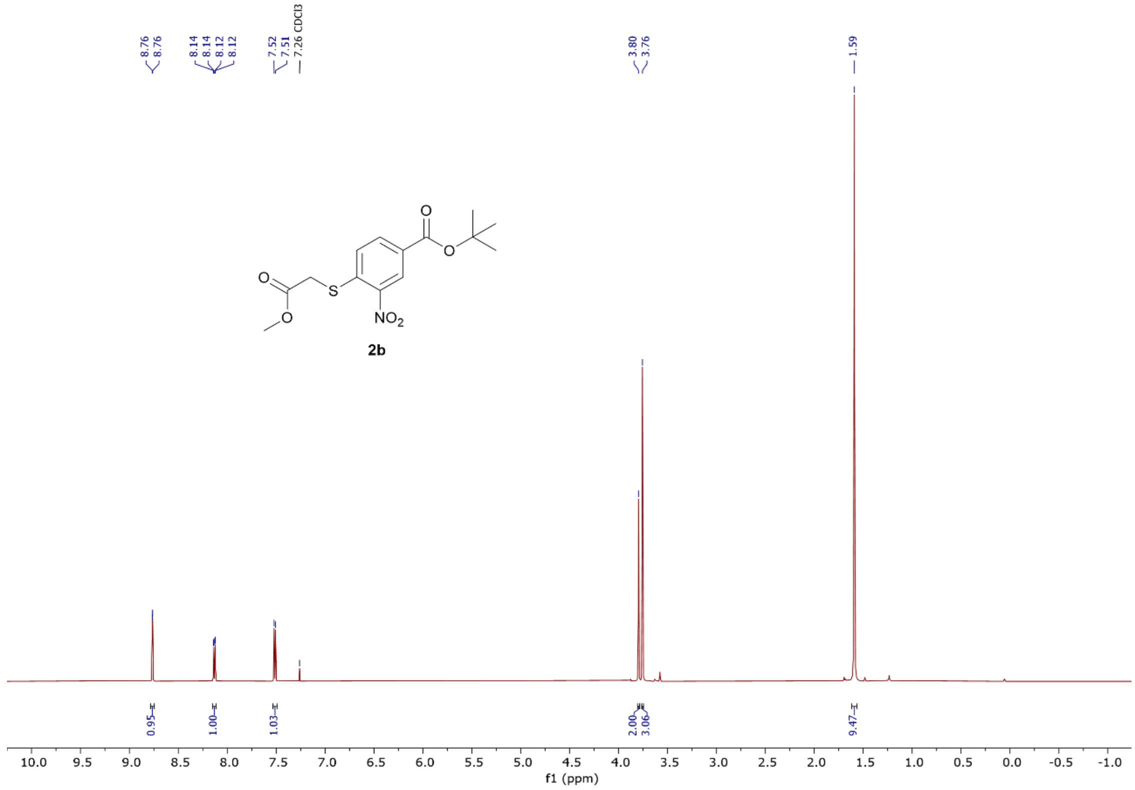

Figure S66. ^{1}H NMR spectrum of compound **2b** (600 MHz, $CDCl_3$).

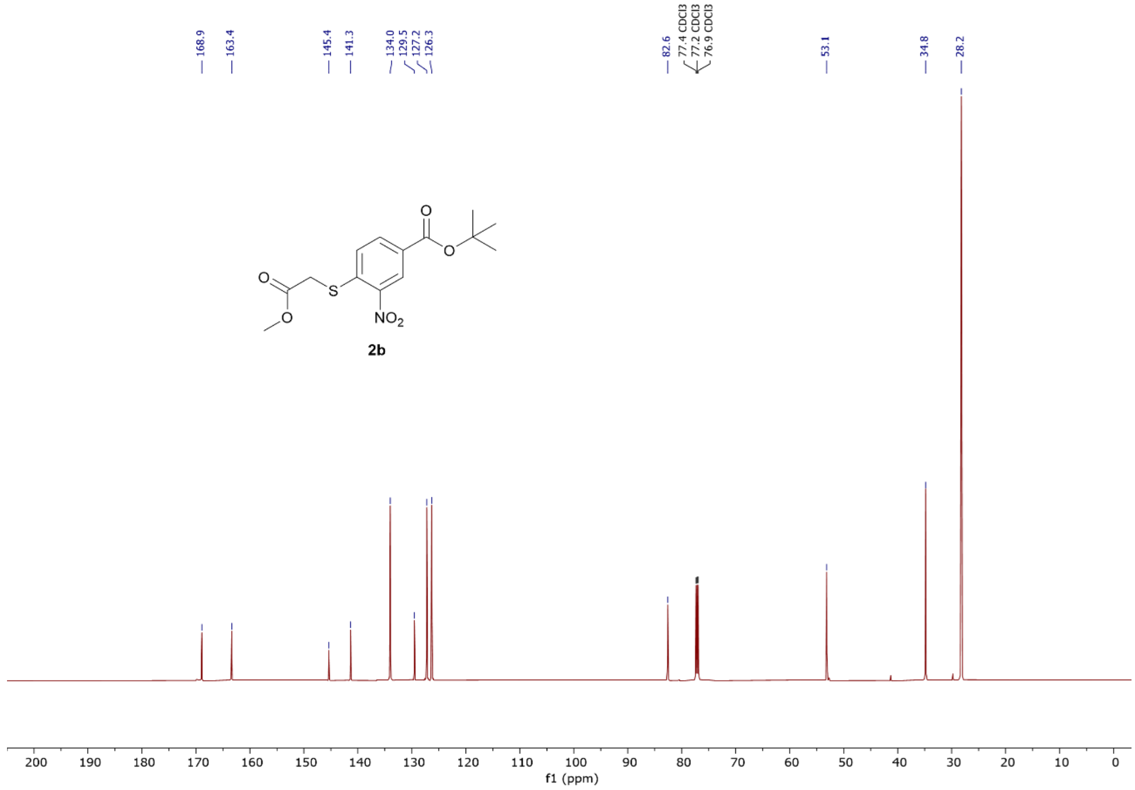

Figure S67. ^{13}C NMR spectrum of compound **2b** (151 MHz, $CDCl_3$).

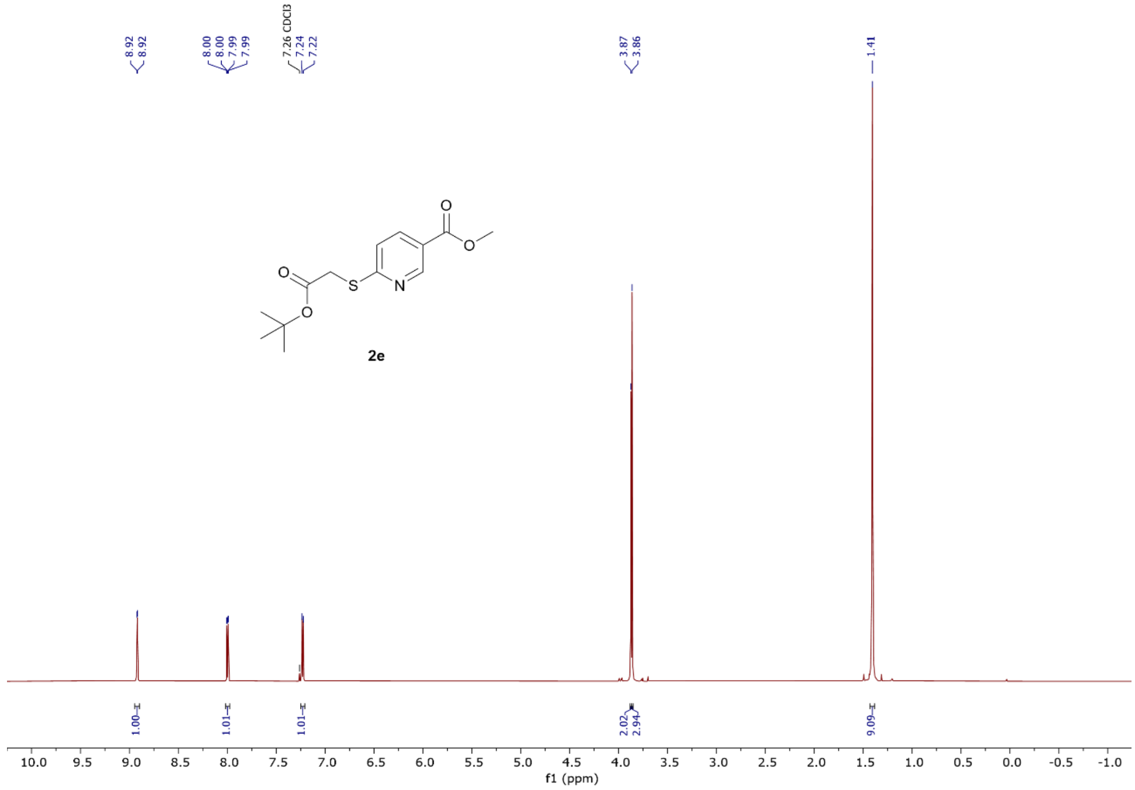

Figure S68. ^{1}H NMR spectrum of compound **2e** (700 MHz, $CDCl_3$).

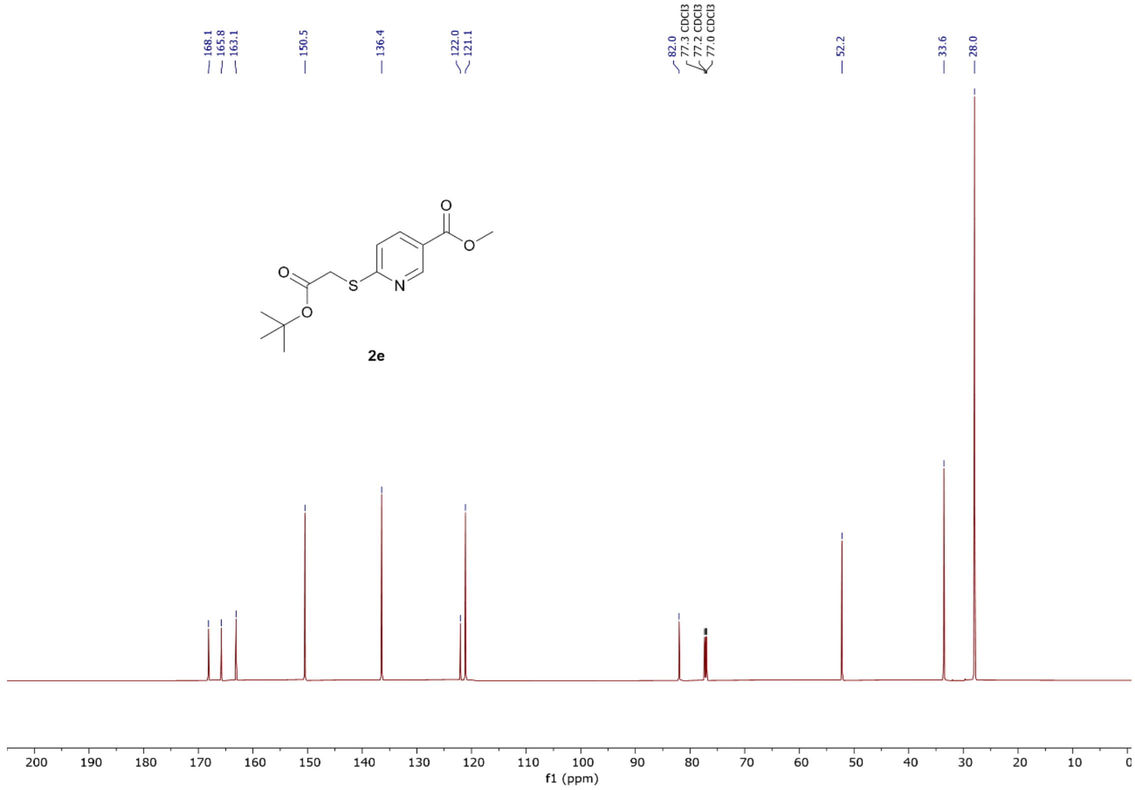

Figure S69. ^{13}C NMR spectrum of compound **2e** (176 MHz, $CDCl_3$).

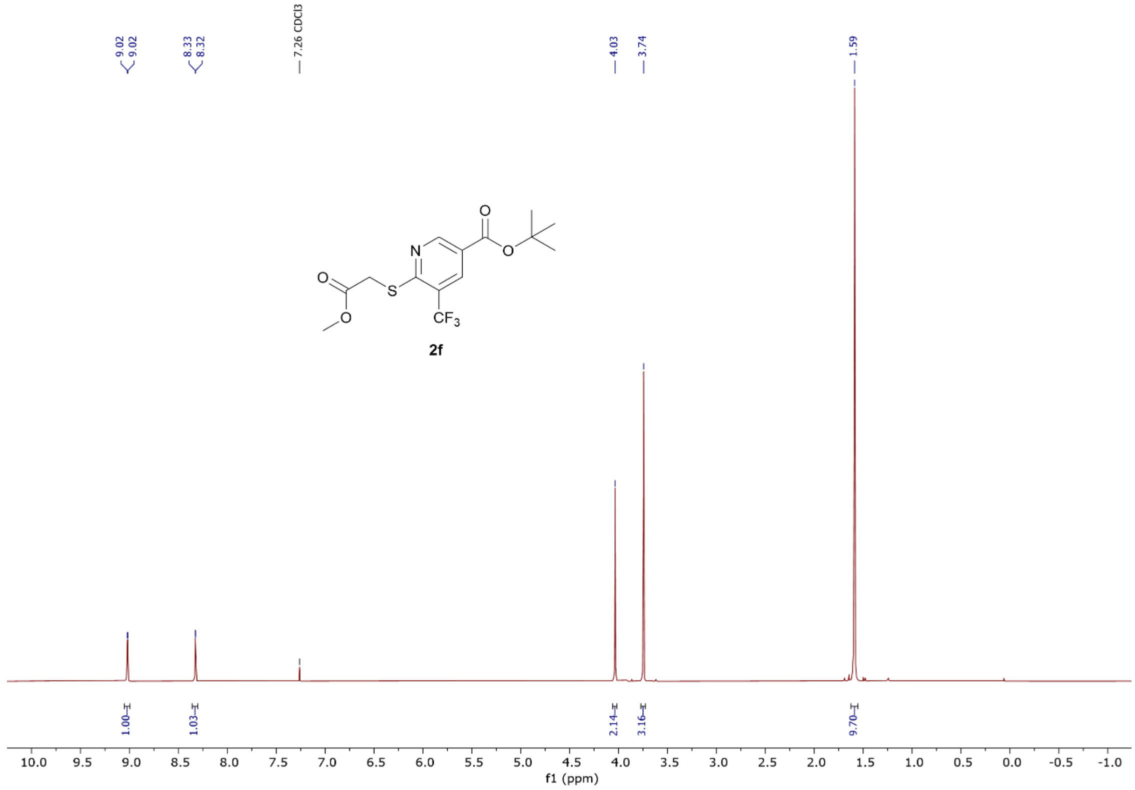

Figure S70. ^{1}H NMR spectrum of compound **2f** (600 MHz, $CDCl_3$).

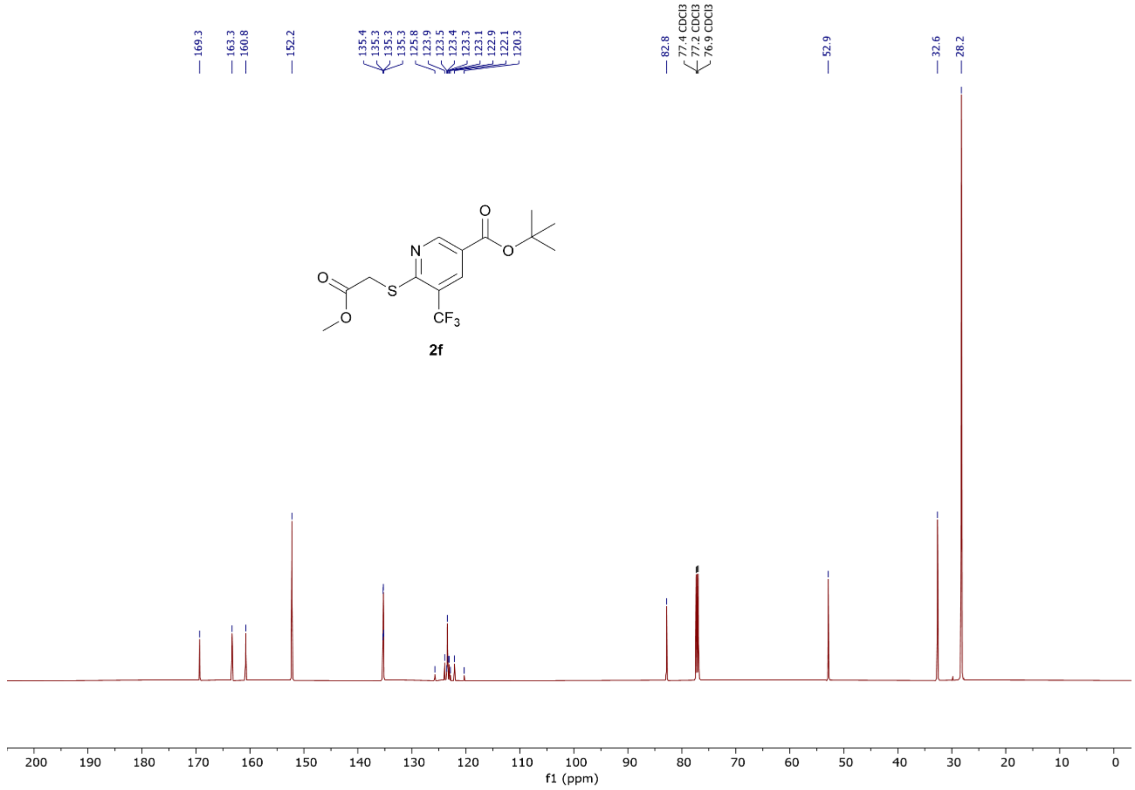

Figure S71. ^{13}C NMR spectrum of compound **2f** (151 MHz, $CDCl_3$).

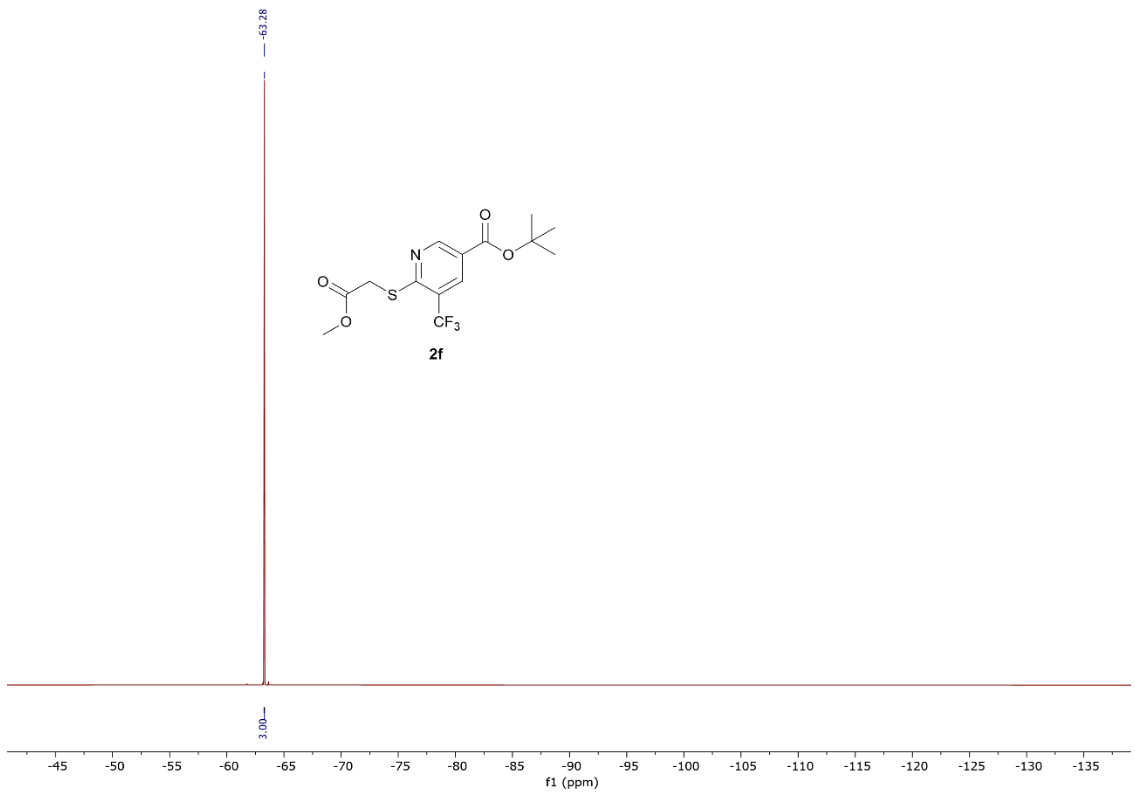

Figure S72. ^{19}F NMR spectrum of compound **2f** (564 MHz, $CDCl_3$).

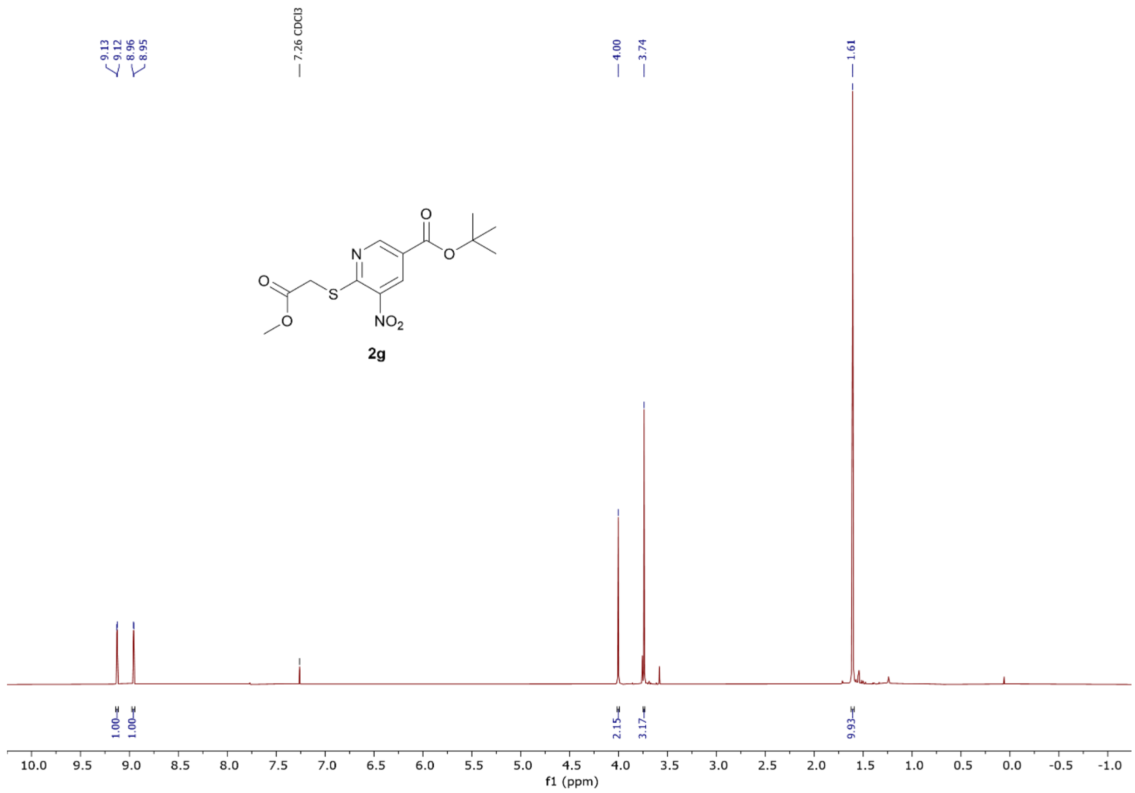

Figure S73. ^{1}H NMR spectrum of compound **2g** (600 MHz, $CDCl_3$).

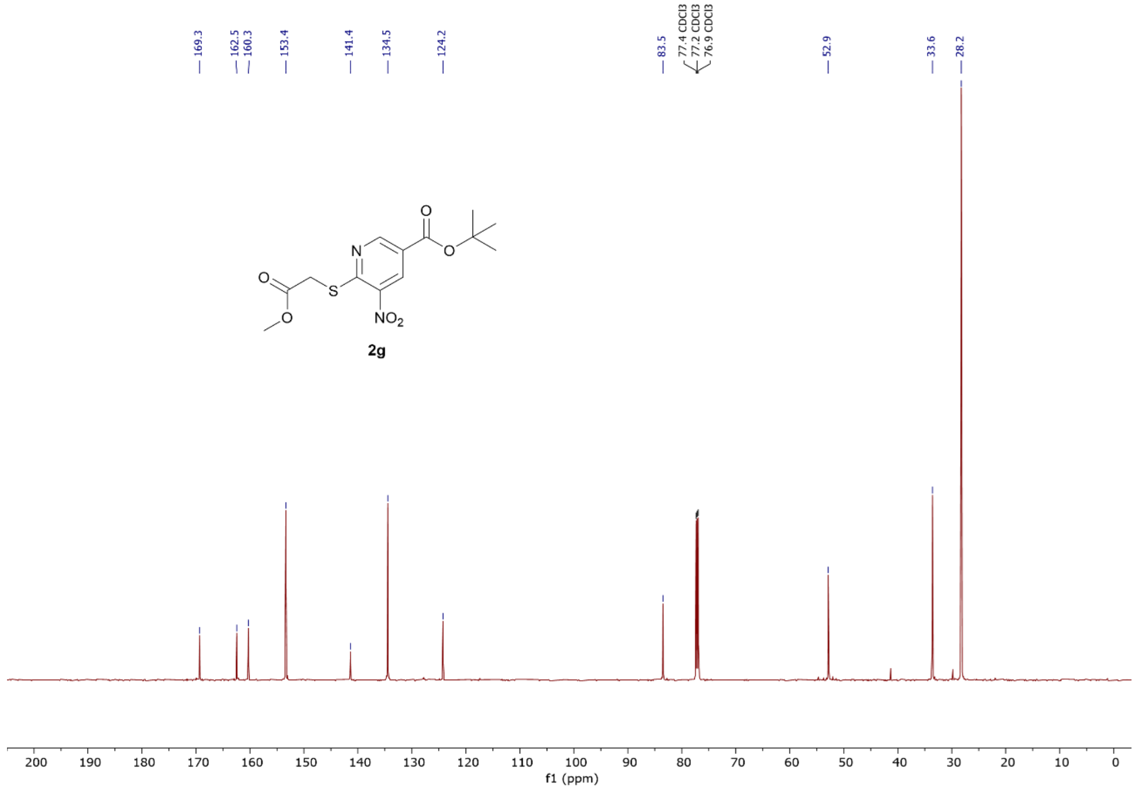

Figure S74. ^{13}C NMR spectrum of compound **2g** (151 MHz, $CDCl_3$).

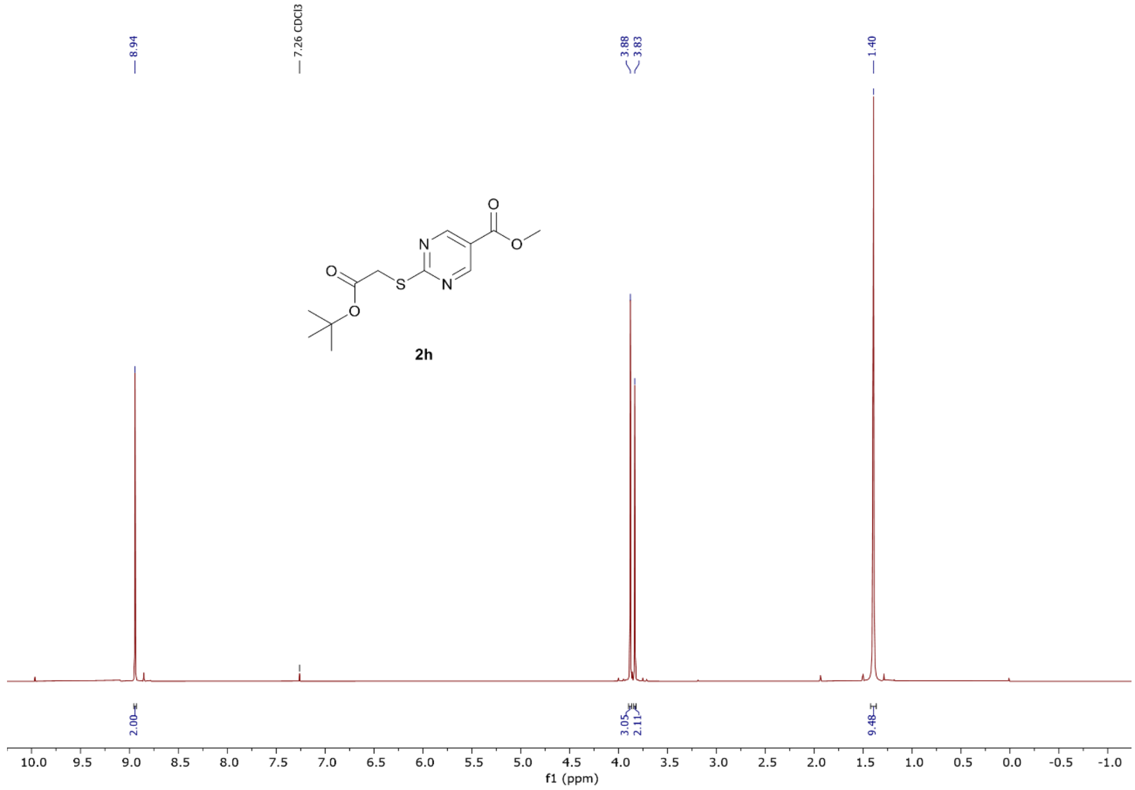

Figure S75. ^{1}H NMR spectrum of compound **2h** (600 MHz, $CDCl_3$).

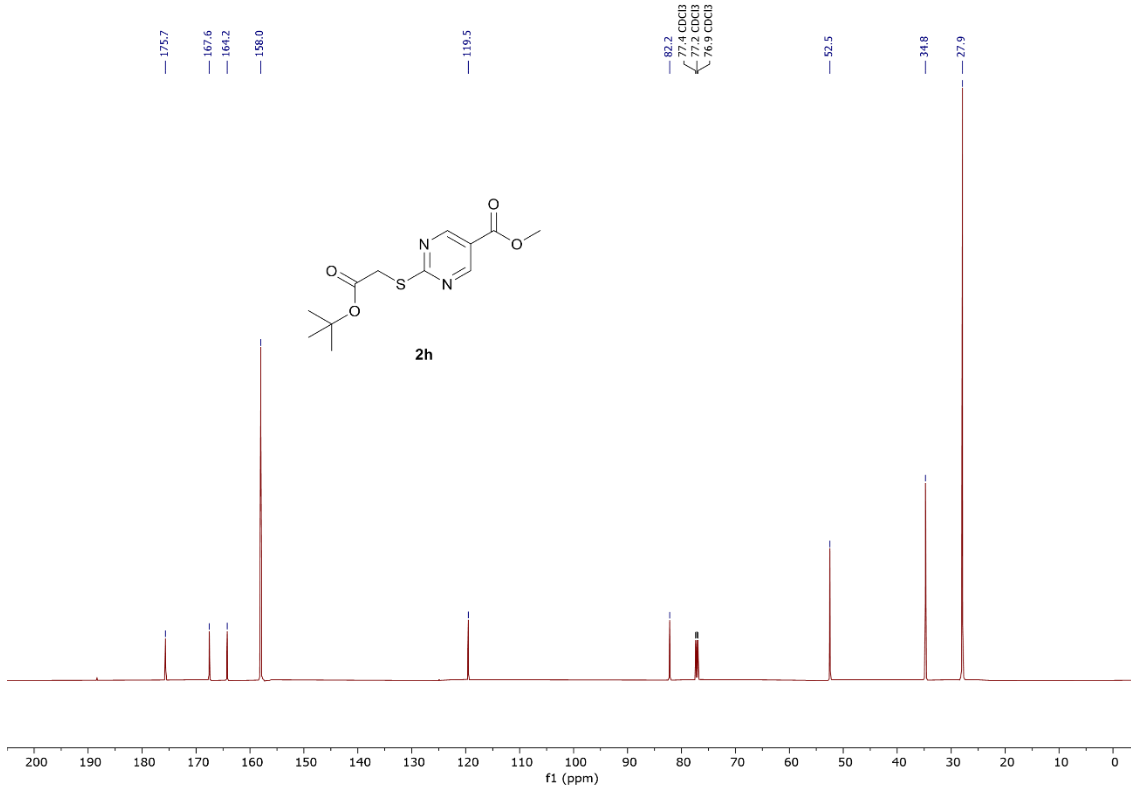

Figure S76. ^{13}C NMR spectrum of compound **2h** (151 MHz, $CDCl_3$).

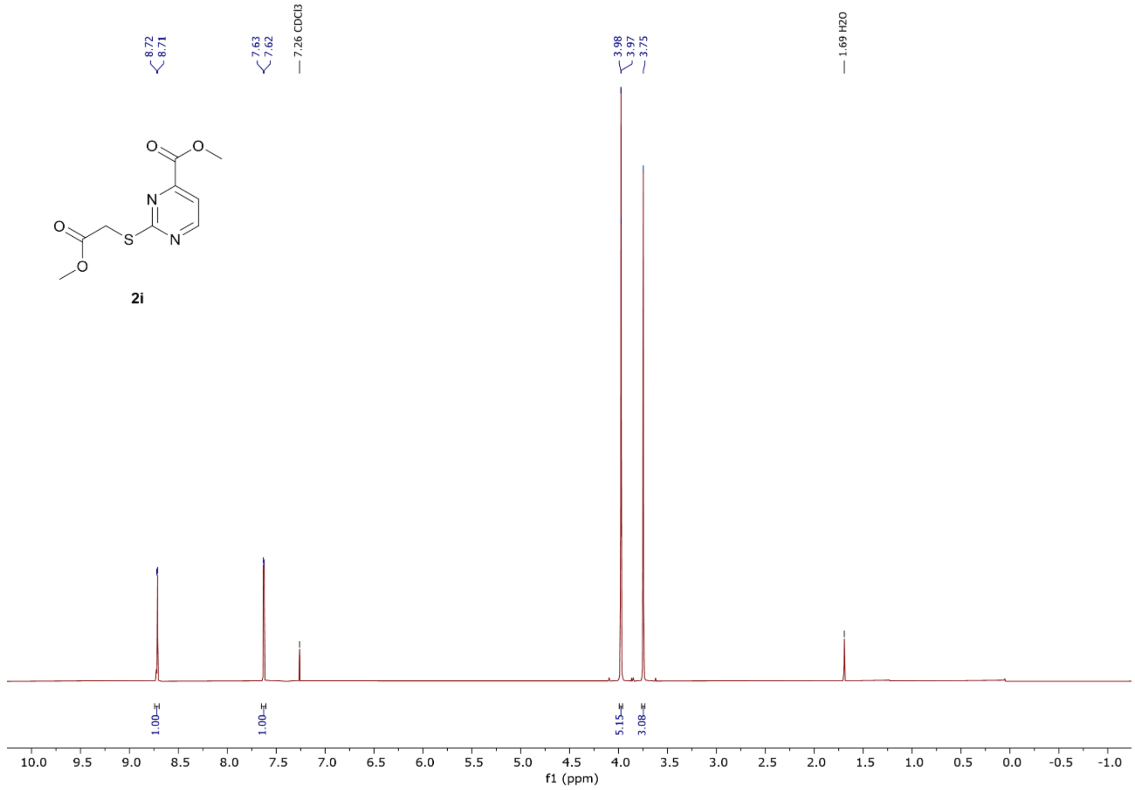

Figure S77. ^{1}H NMR spectrum of compound **2i** (600 MHz, $CDCl_3$).

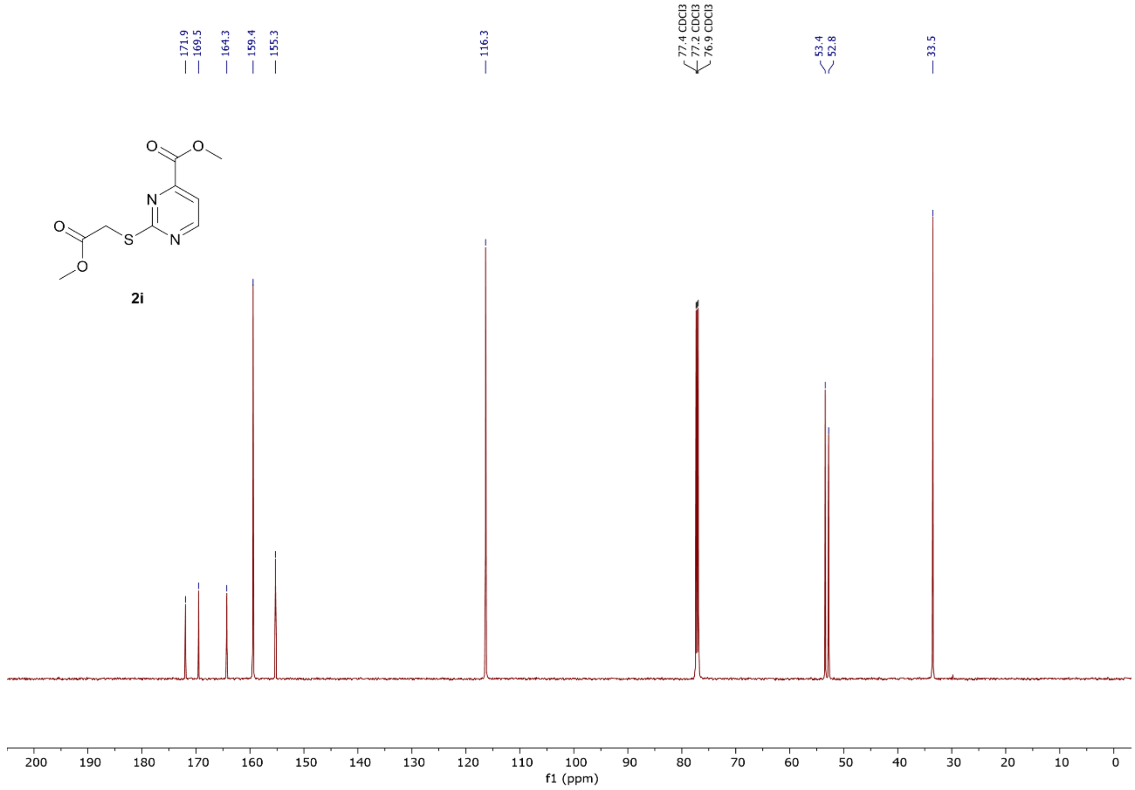

Figure S78. ^{13}C NMR spectrum of compound **2i** (151 MHz, $CDCl_3$).

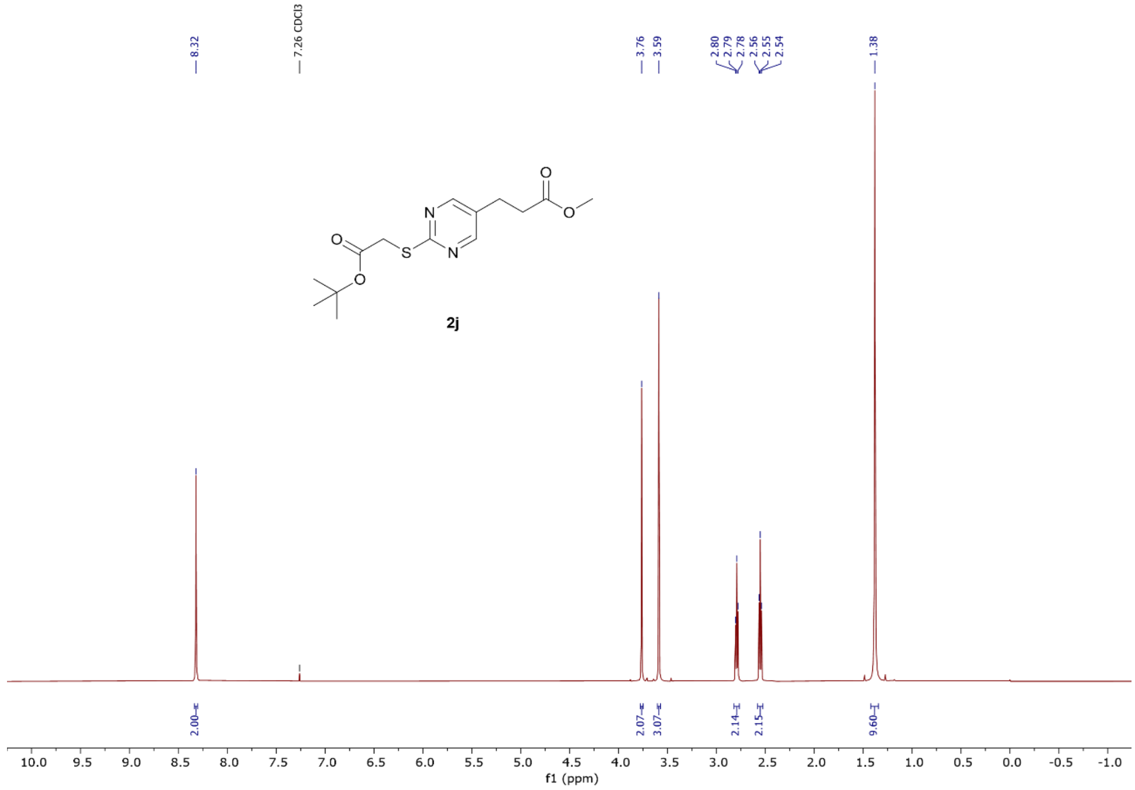

Figure S79. ^{1}H NMR spectrum of compound **2j** (600 MHz, $CDCl_3$).

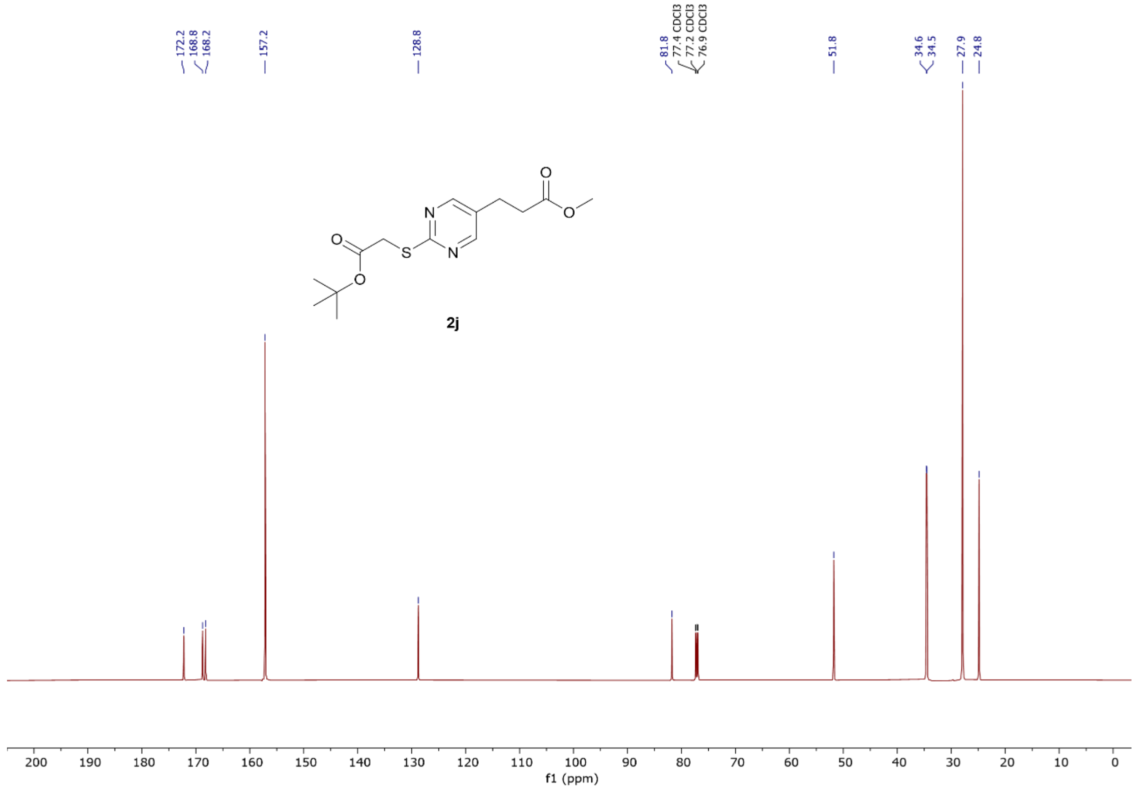

Figure S80. ^{13}C NMR spectrum of compound **2j** (151 MHz, $CDCl_3$).

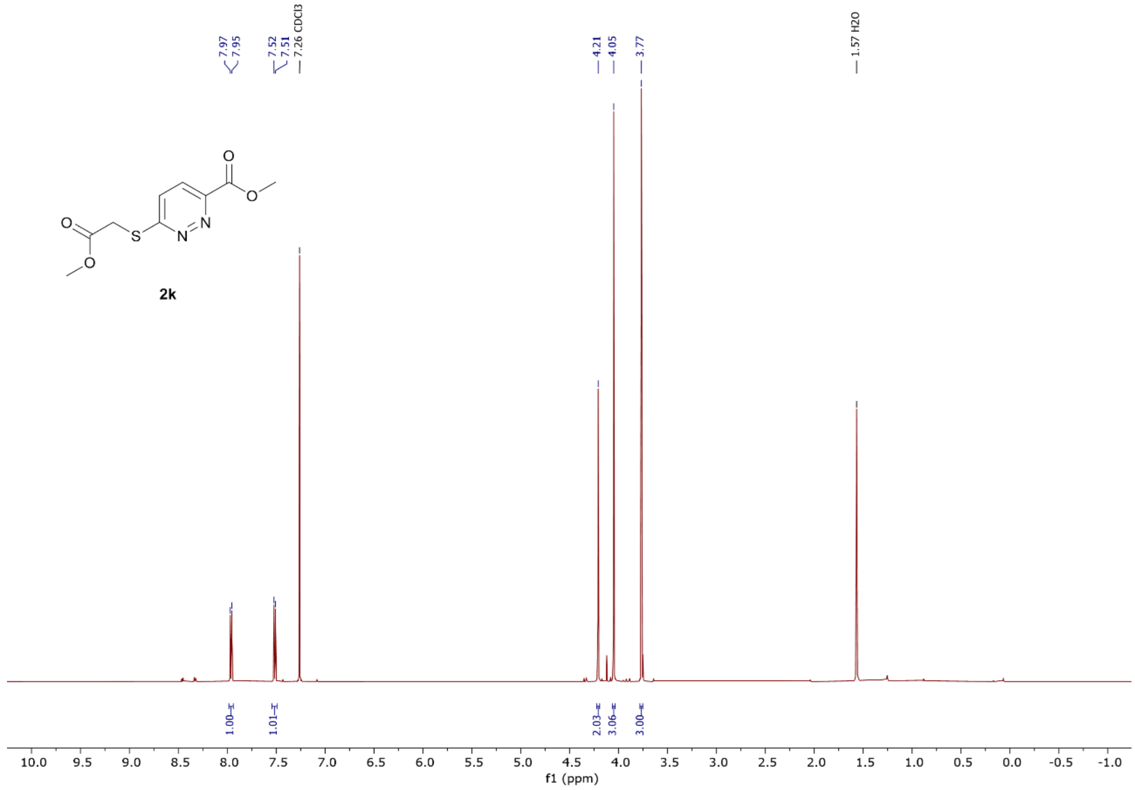

Figure S81. ^{1}H NMR spectrum of compound **2k** (600 MHz, $CDCl_3$).

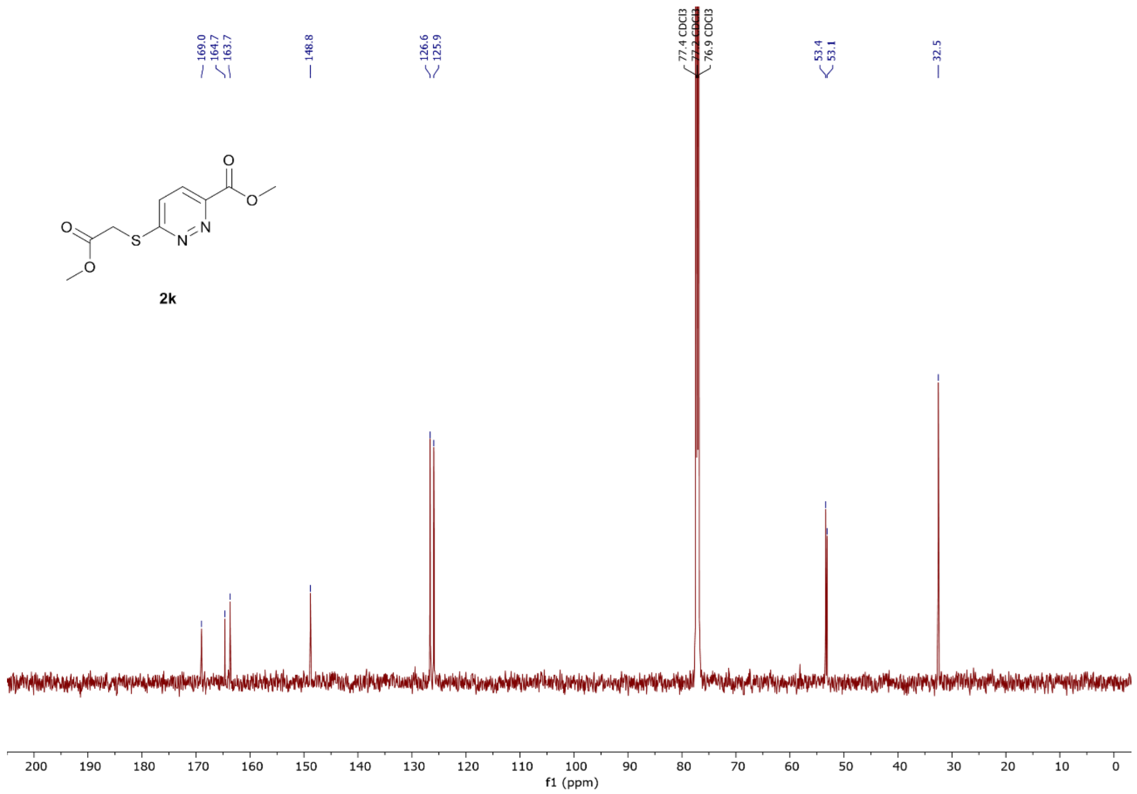

Figure S82. ^{13}C NMR spectrum of compound **2k** (151 MHz, $CDCl_3$).

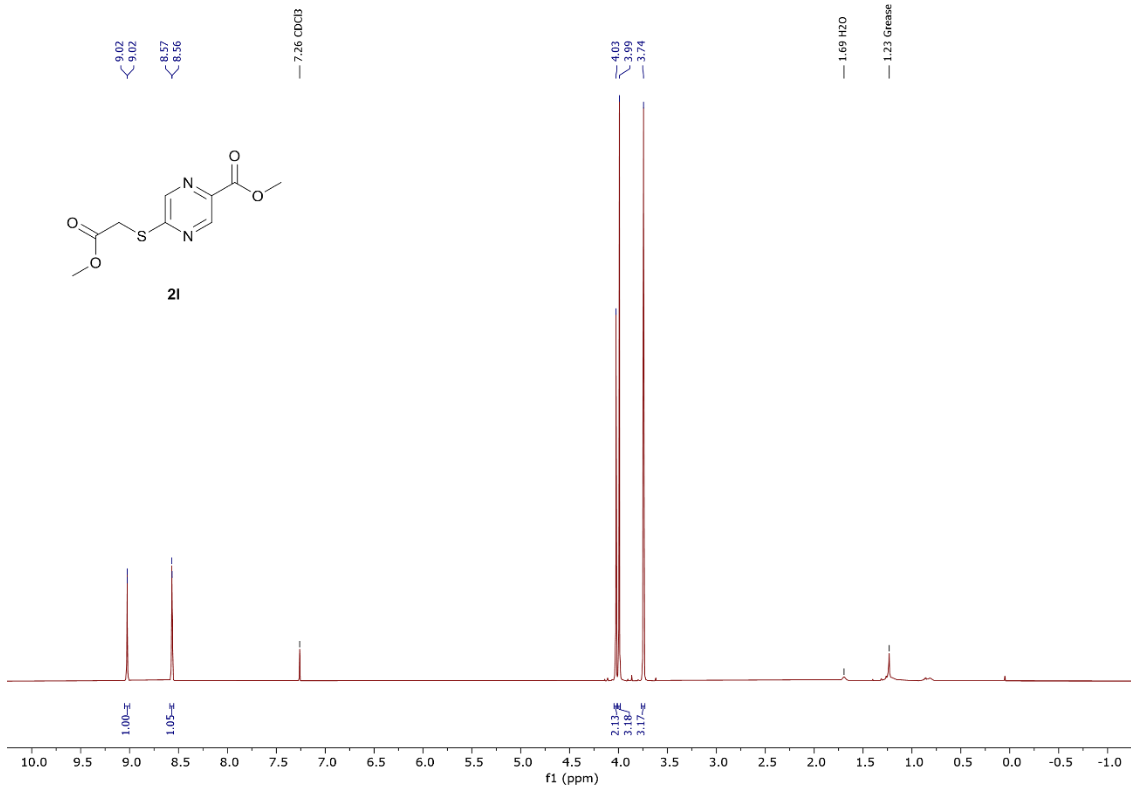

Figure S83. ^{1}H NMR spectrum of compound **2l** (600 MHz, $CDCl_3$).

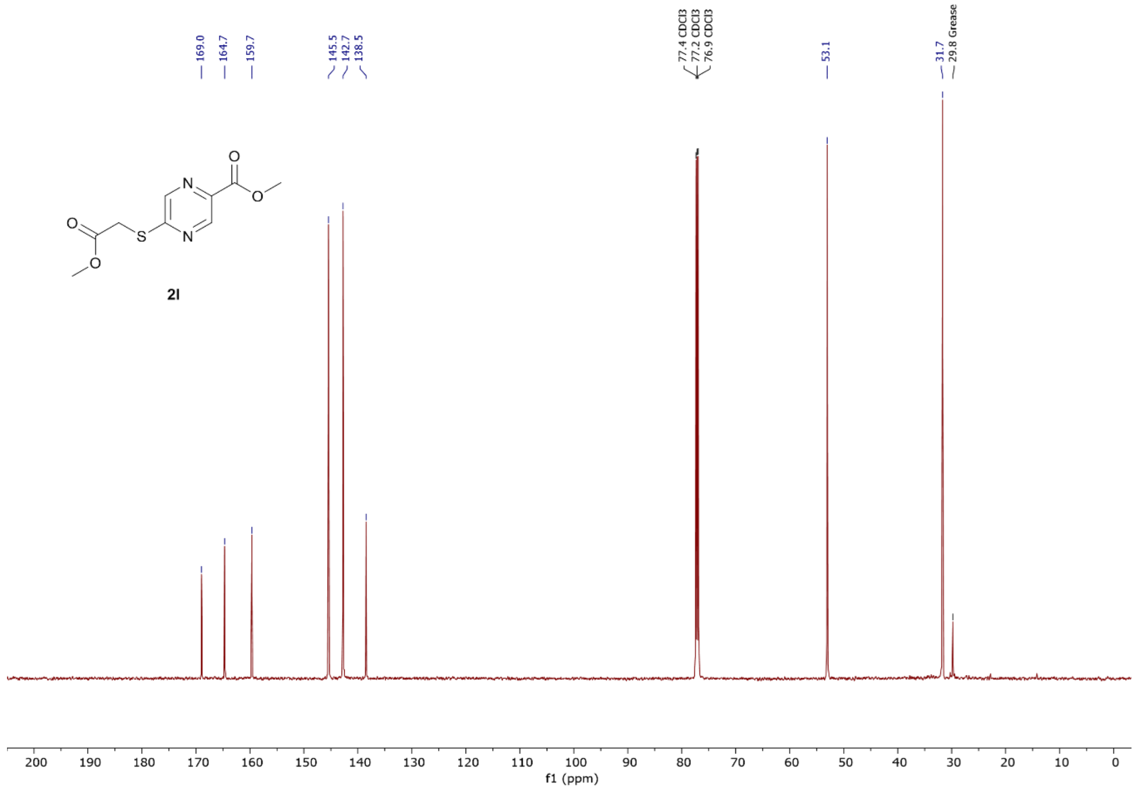

Figure S84. ^{13}C NMR spectrum of compound **2l** (151 MHz, $CDCl_3$).

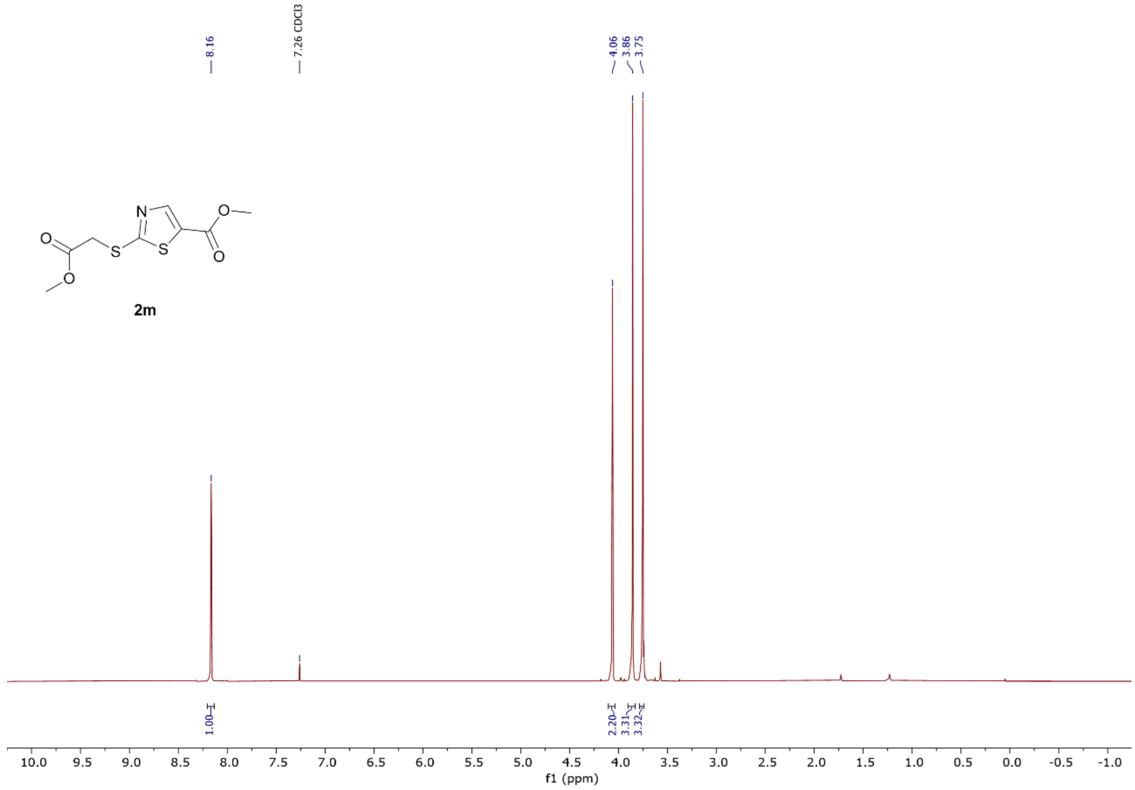

Figure S85. ^{1}H NMR spectrum of compound **2m** (600 MHz, $CDCl_3$).

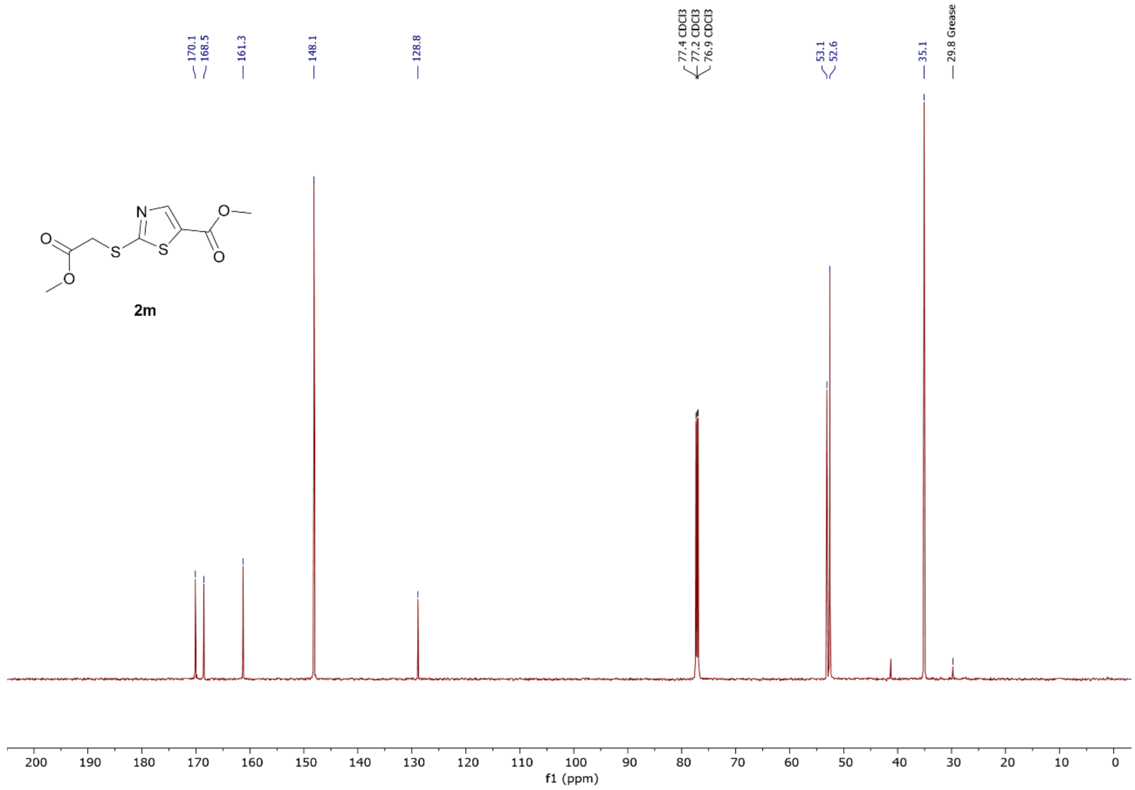

Figure S86. ^{13}C NMR spectrum of compound **2m** (151 MHz, $CDCl_3$).

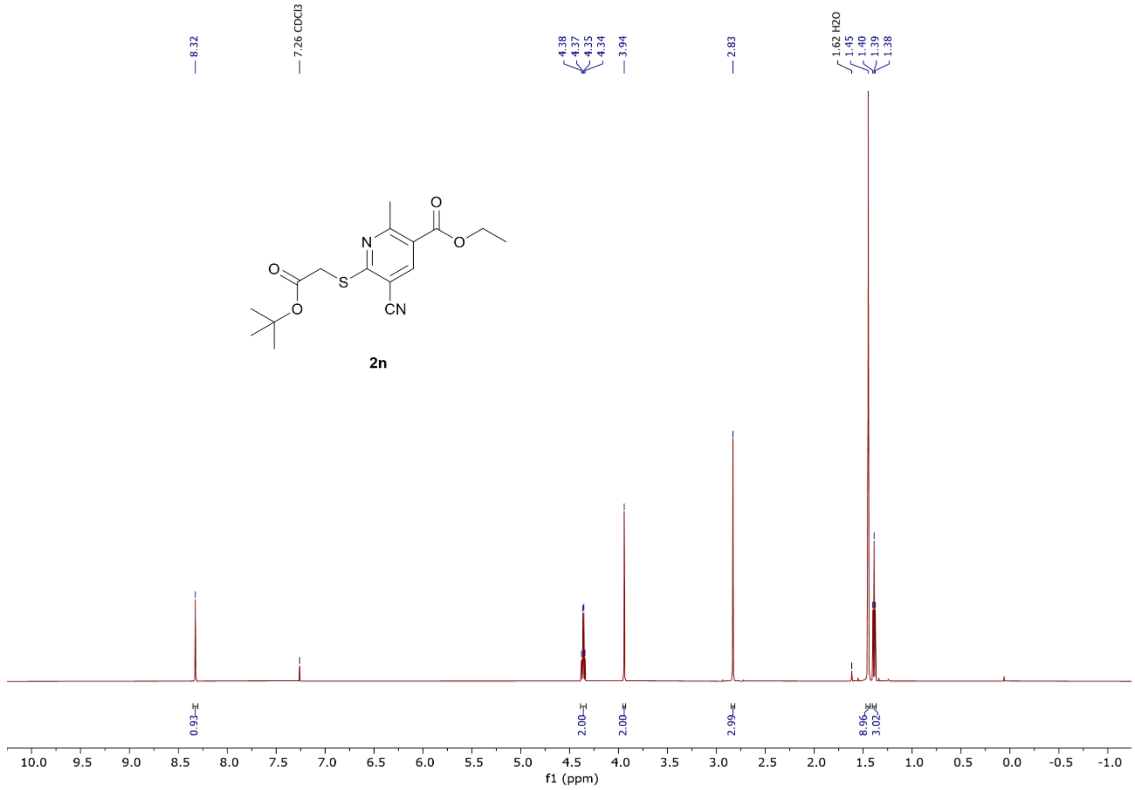

Figure S87. ^{1}H NMR spectrum of compound **2n** (600 MHz, $CDCl_3$).

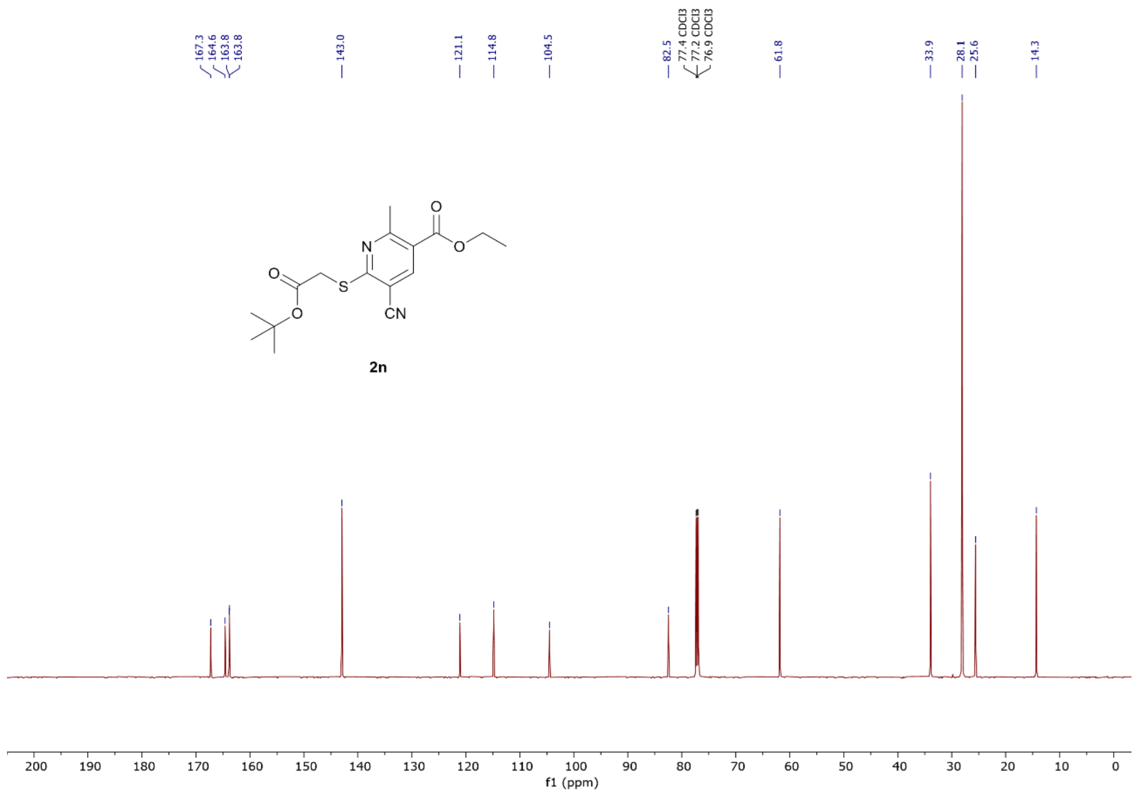

Figure S88. ^{13}C NMR spectrum of compound **2n** (151 MHz, $CDCl_3$).

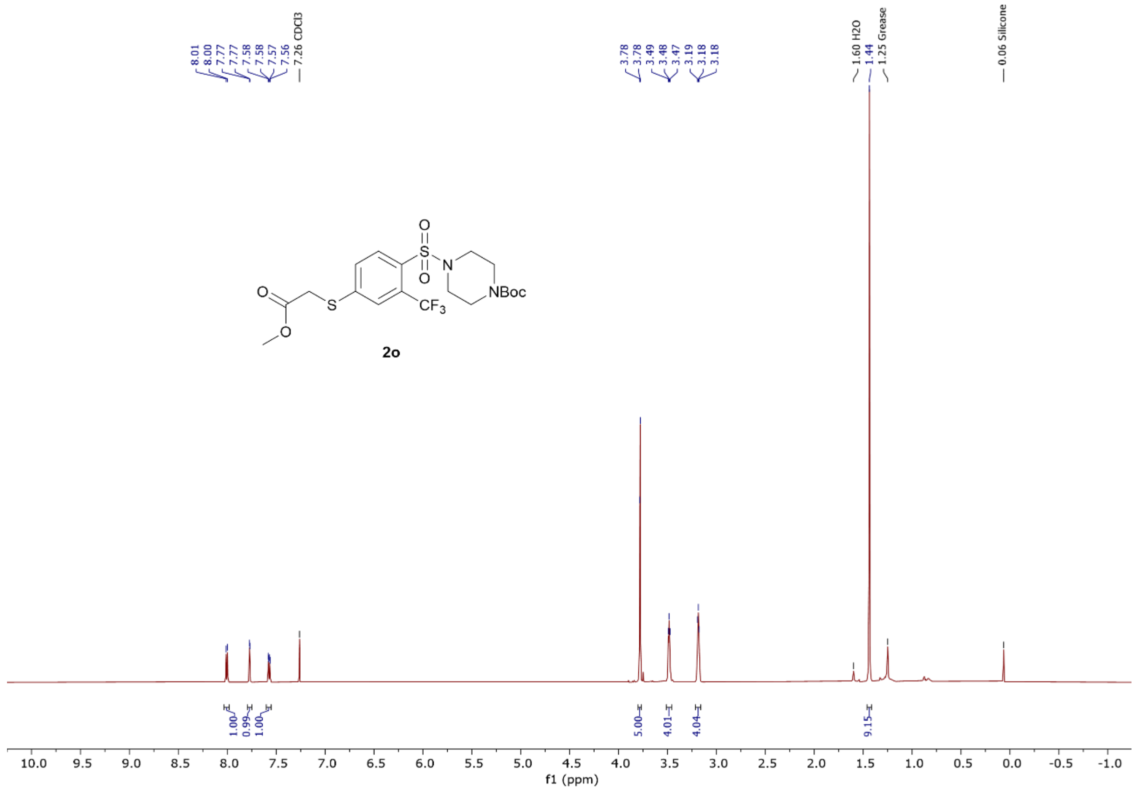

Figure S89. ^{1}H NMR spectrum of compound **2o** (600 MHz, $CDCl_3$).

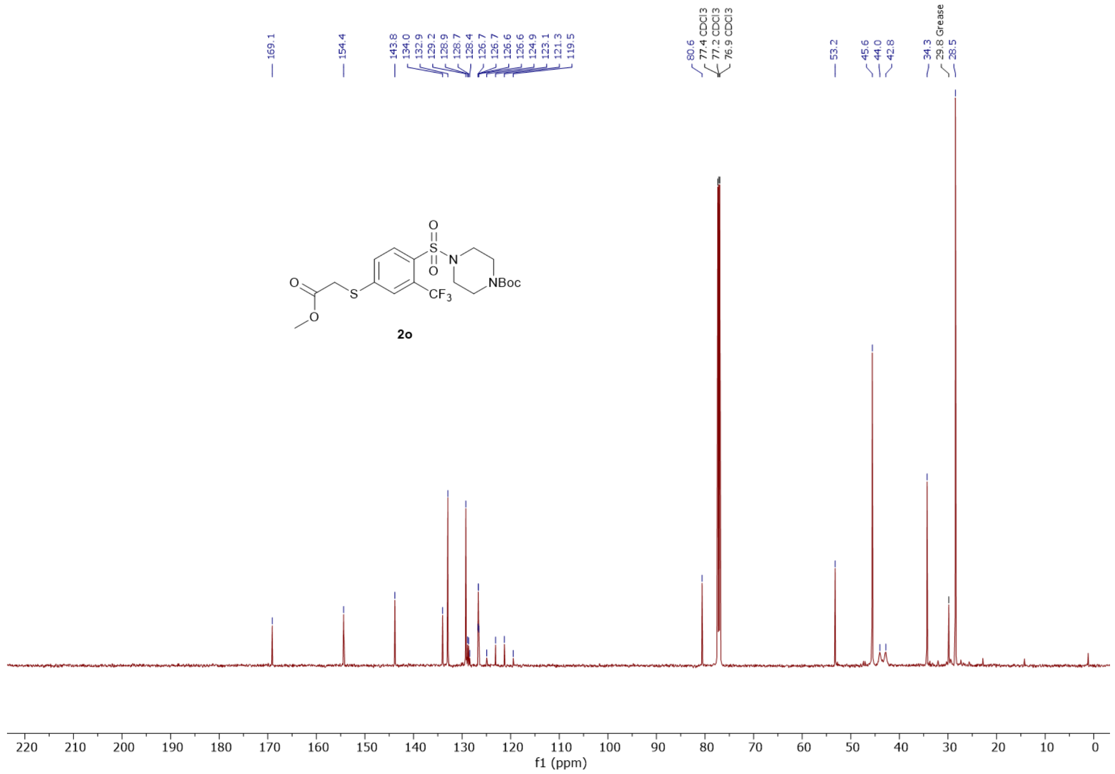

Figure S90. ^{13}C NMR spectrum of compound **2o** (151 MHz, $CDCl_3$).

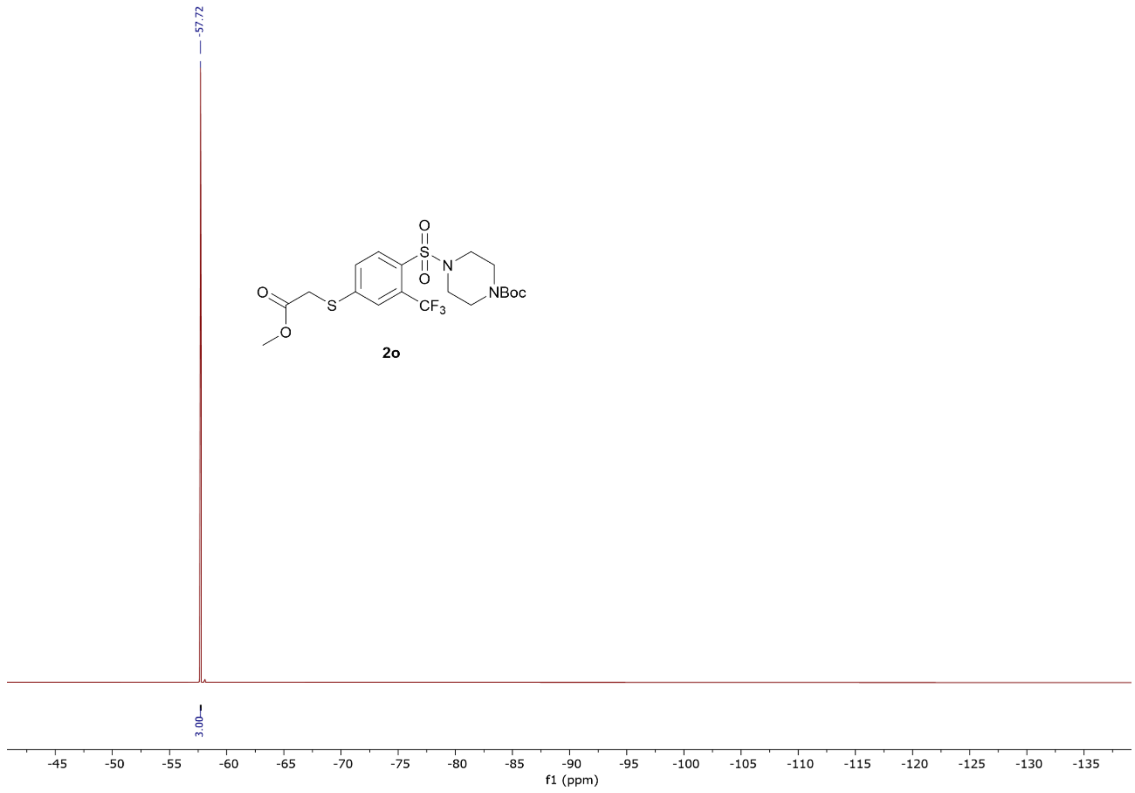

Figure S91. ^{19}F NMR spectrum of compound **2o** (564 MHz, $CDCl_3$).

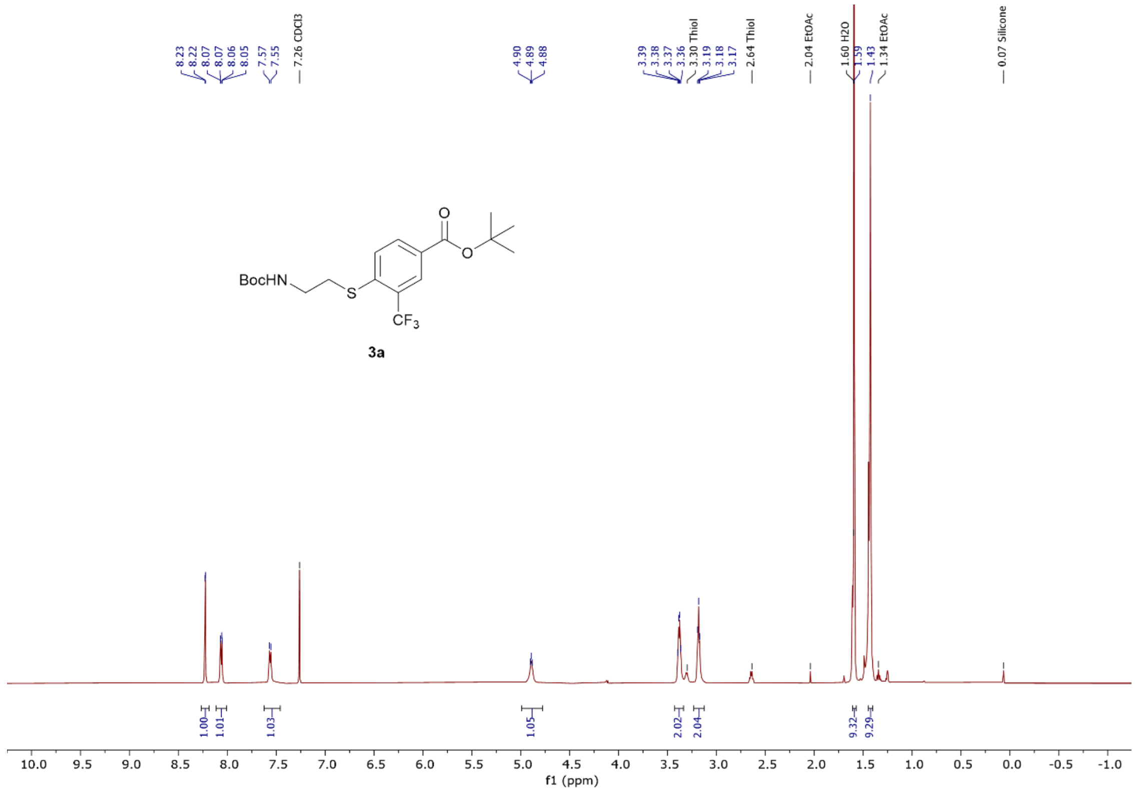

Figure S92. ^{1}H NMR spectrum of compound **3a** (600 MHz, $CDCl_3$).

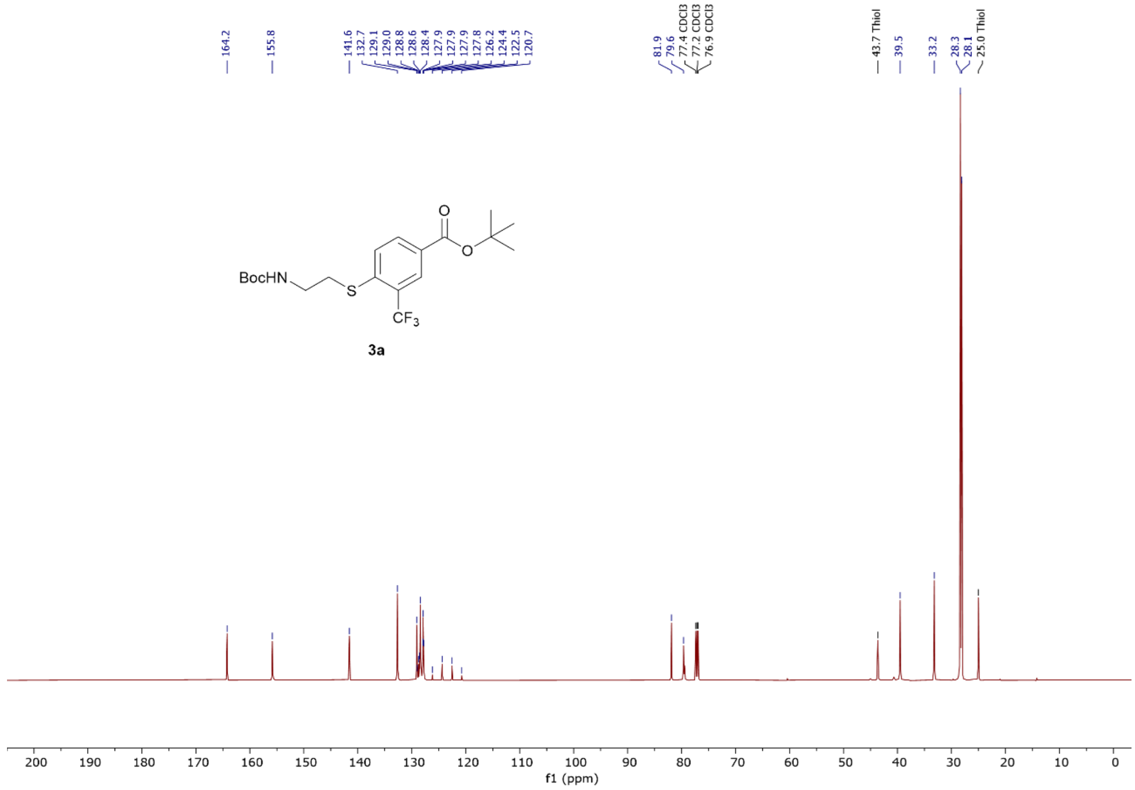

Figure S93. ^{13}C NMR spectrum of compound **3a** (151 MHz, $CDCl_3$).

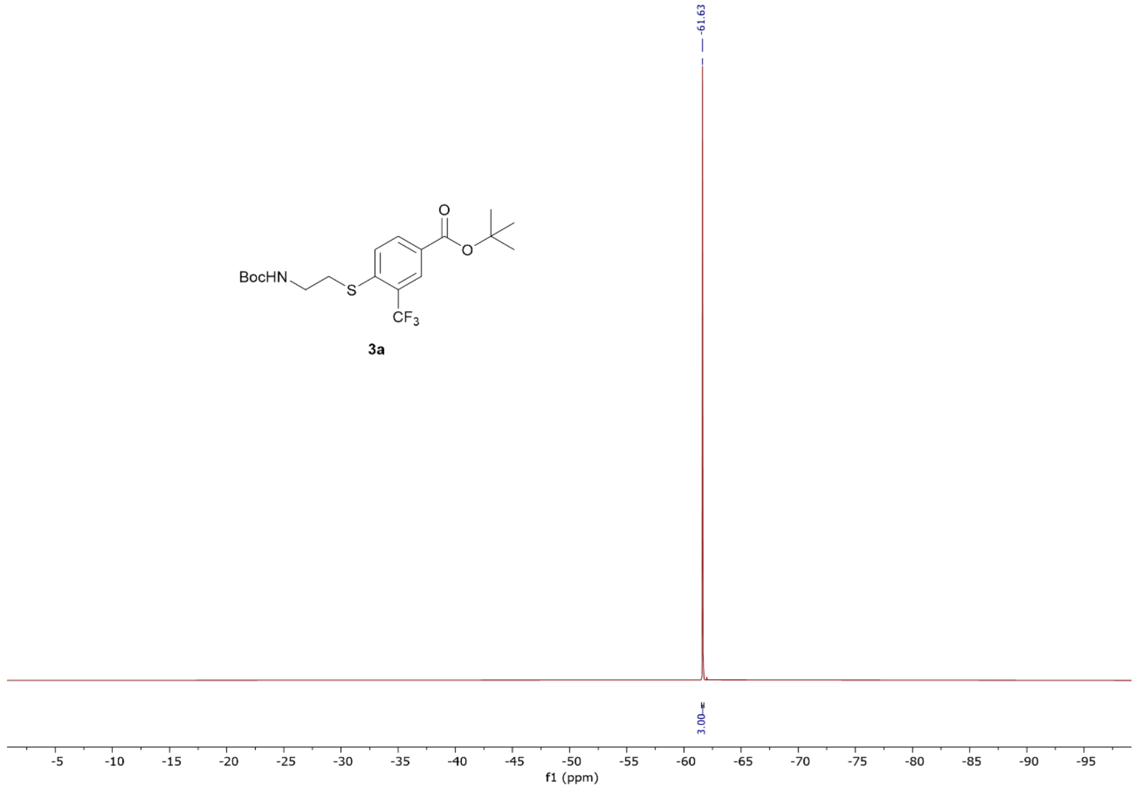

Figure S94. ^{19}F NMR spectrum of compound **3a** (564 MHz, $CDCl_3$).

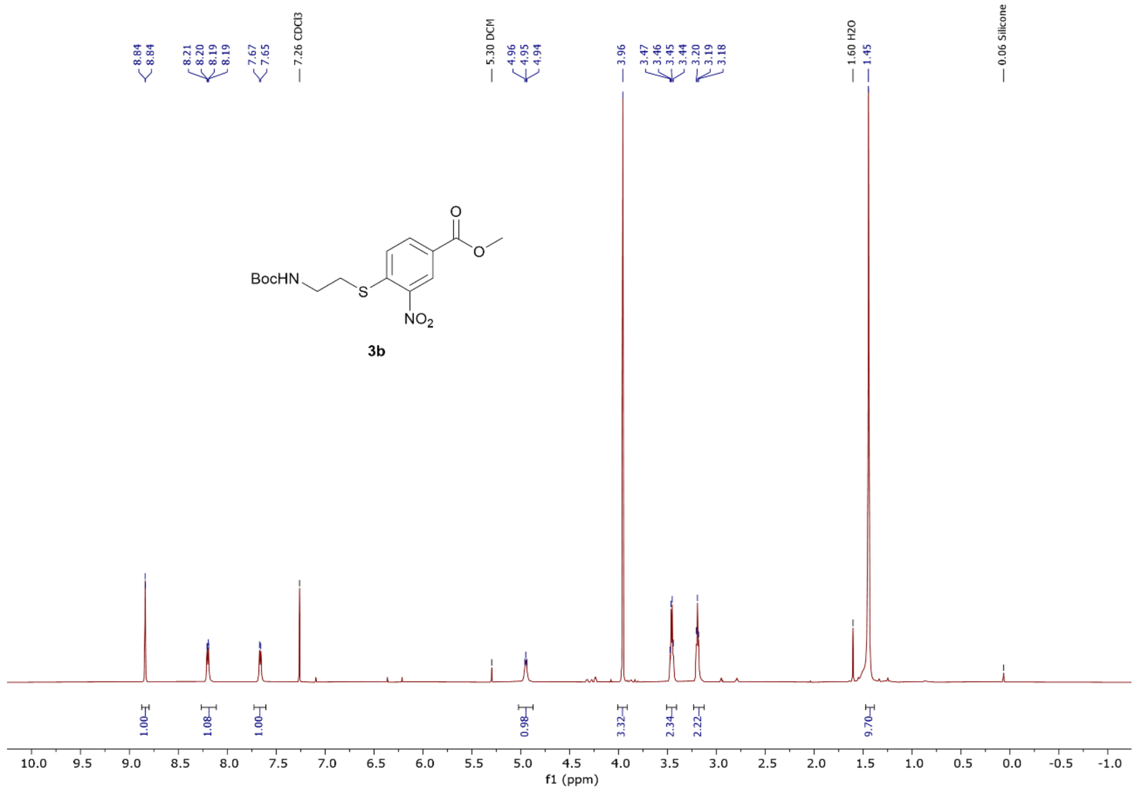

Figure S95. ^{1}H NMR spectrum of compound **3b** (600 MHz, $CDCl_3$).

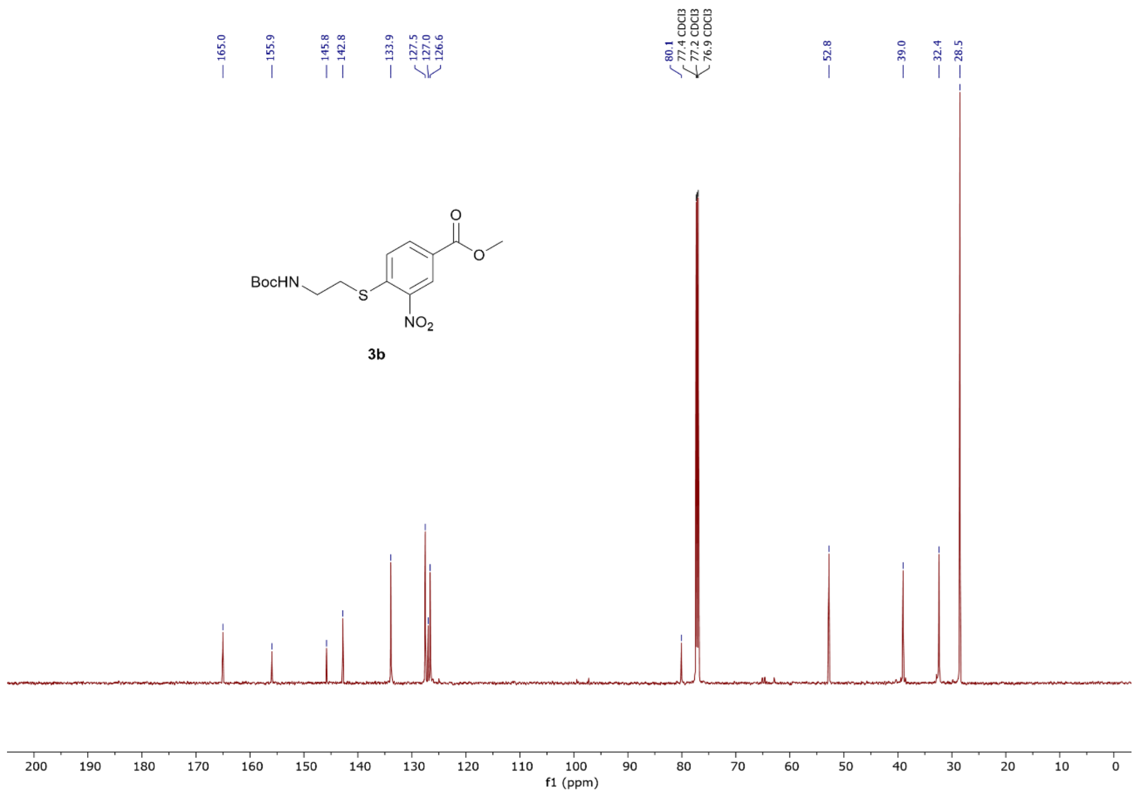

Figure S96. ^{13}C NMR spectrum of compound **3b** (151 MHz, $CDCl_3$).

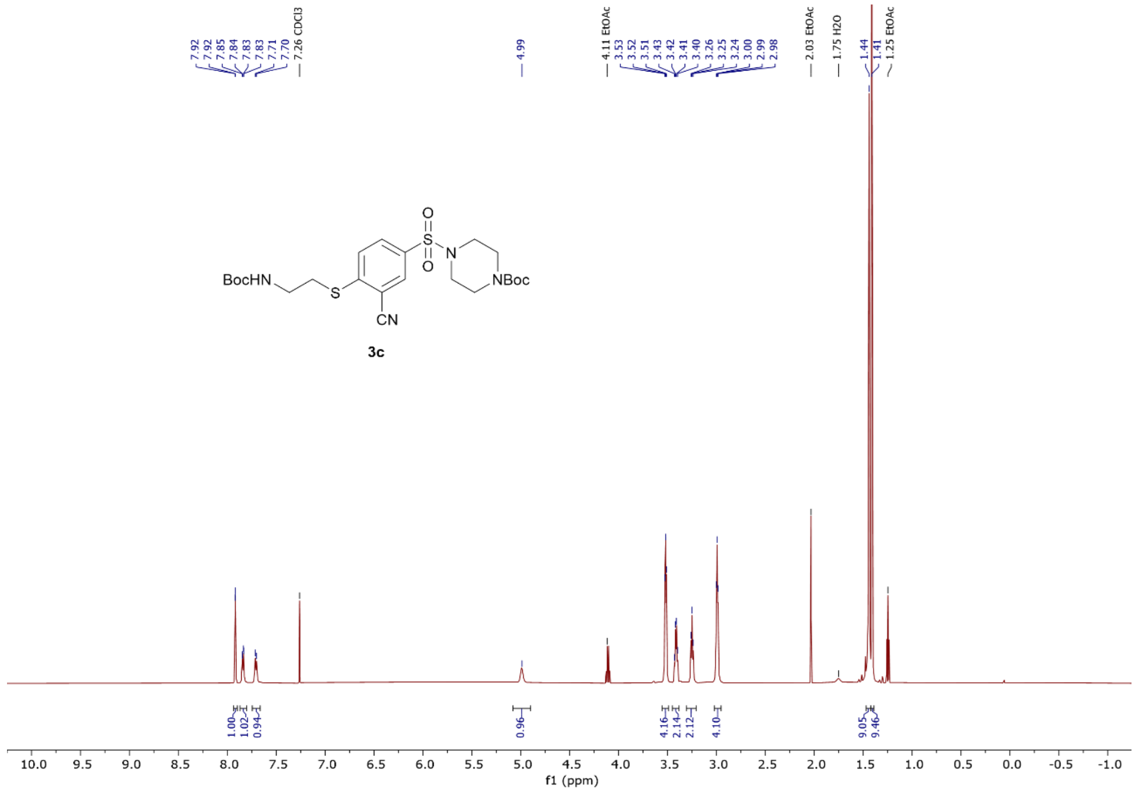

Figure S97. ^{1}H NMR spectrum of compound **3c** (600 MHz, $CDCl_3$).

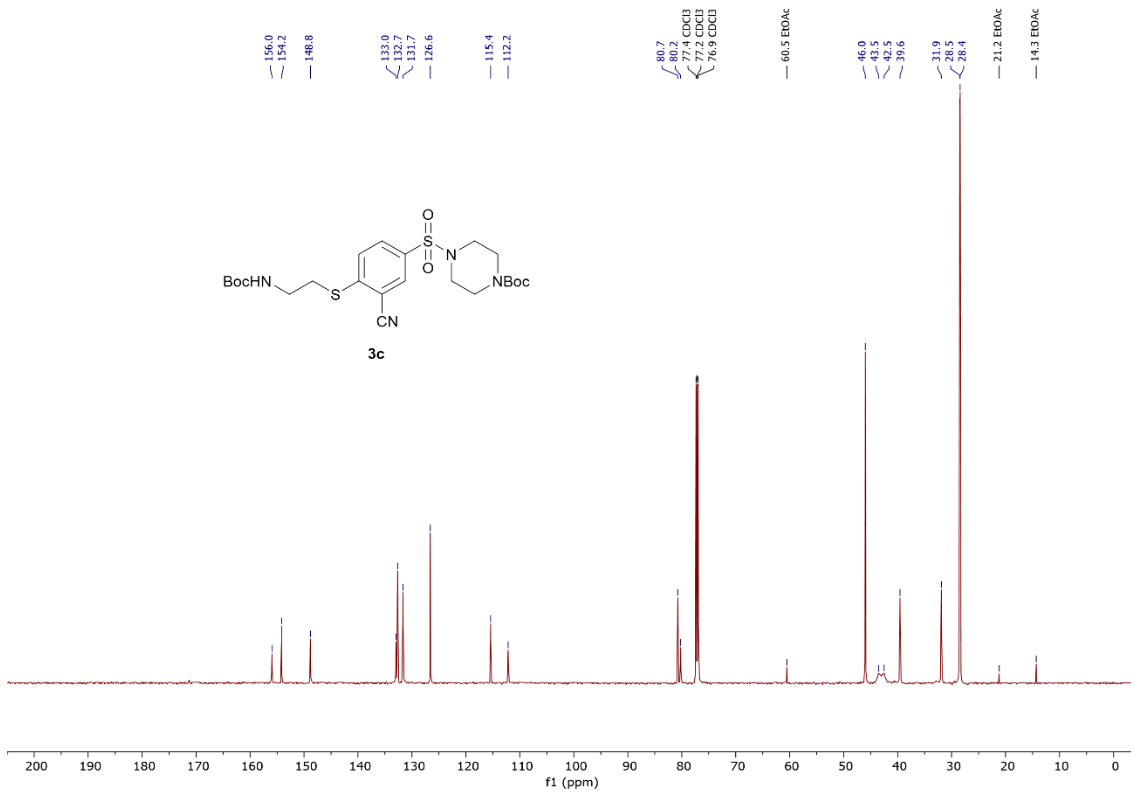

Figure S98. ^{13}C NMR spectrum of compound **3c** (151 MHz, $CDCl_3$).

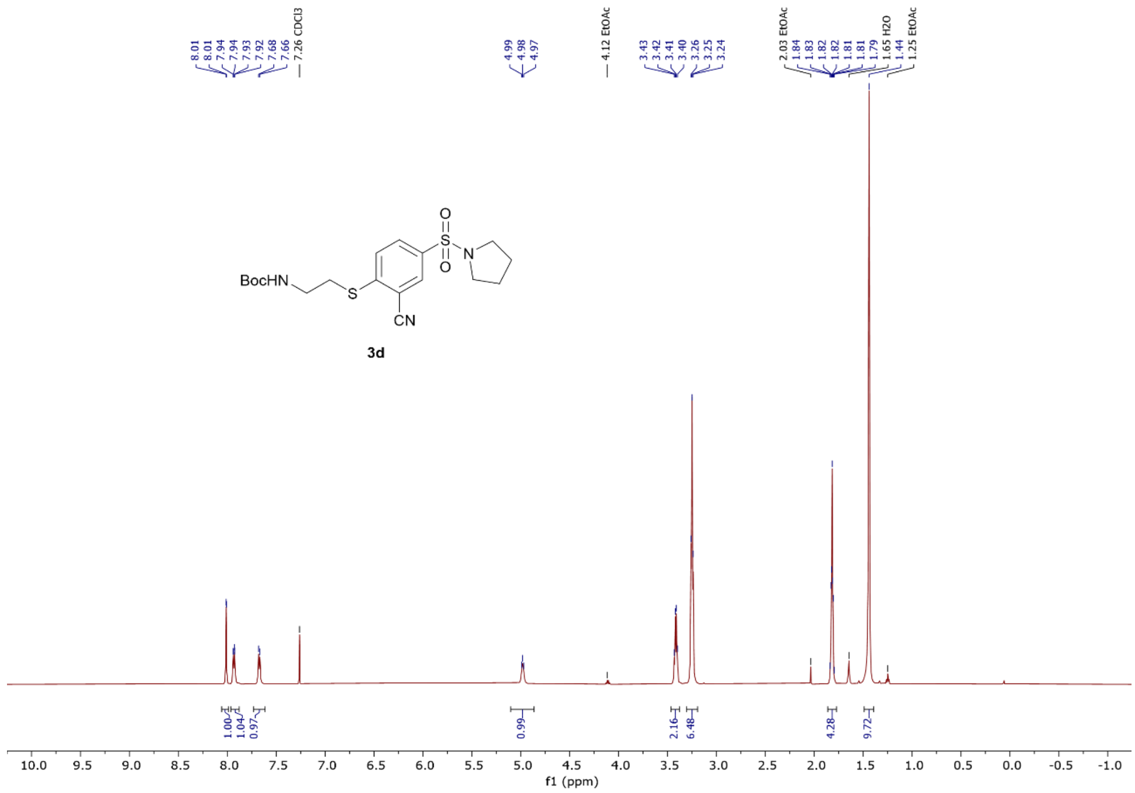

Figure S99. ^{1}H NMR spectrum of compound **3d** (600 MHz, $CDCl_3$).

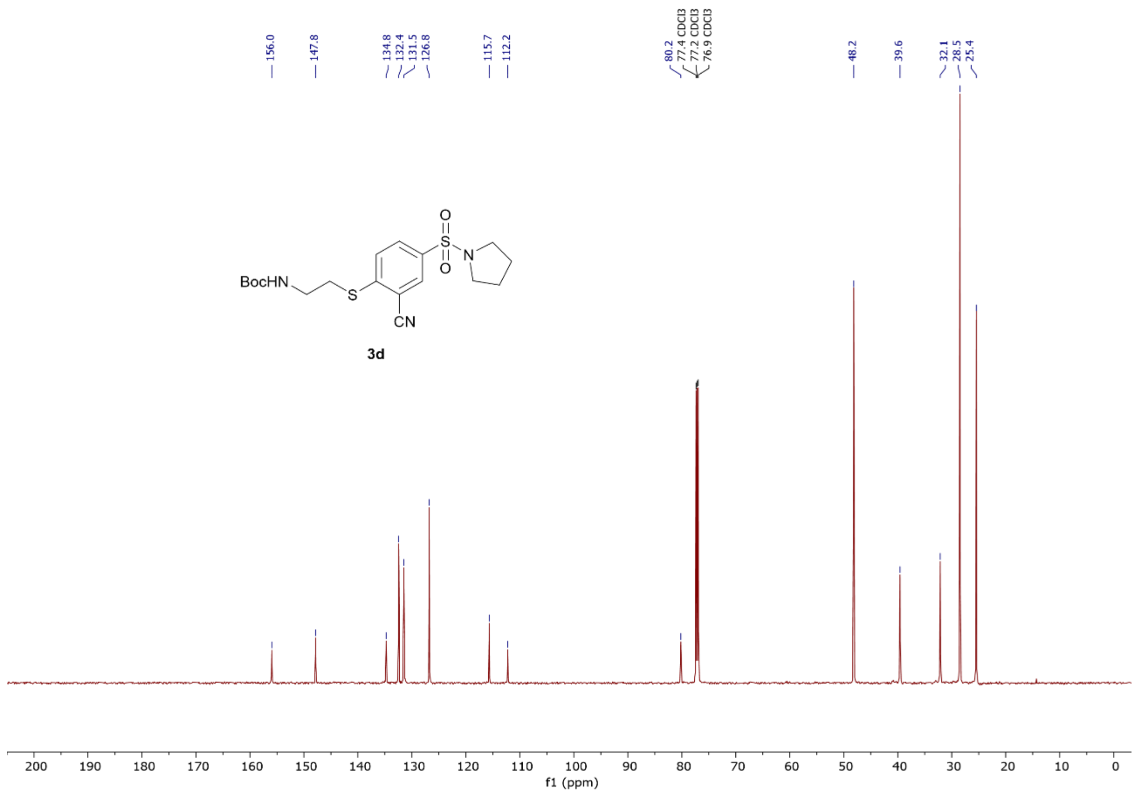

Figure S100. ^{13}C NMR spectrum of compound **3d** (151 MHz, $CDCl_3$).

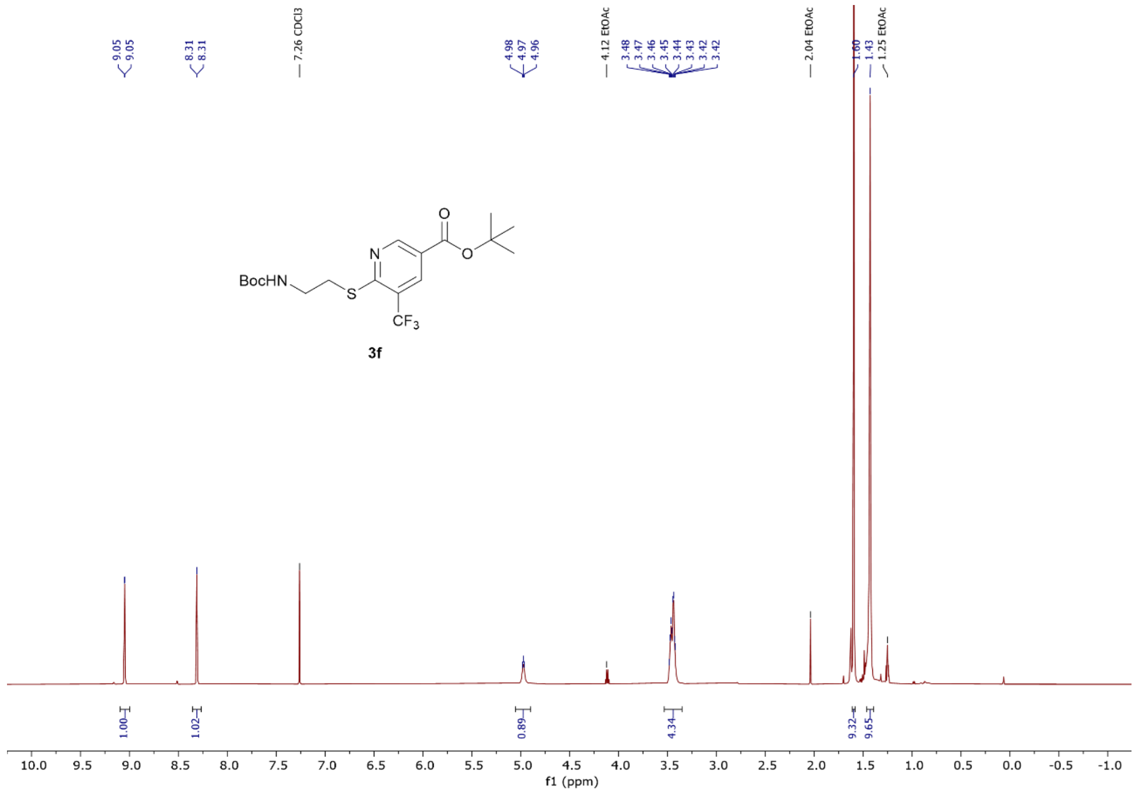

Figure S101. ^{1}H NMR spectrum of compound **3f** (600 MHz, $CDCl_3$).

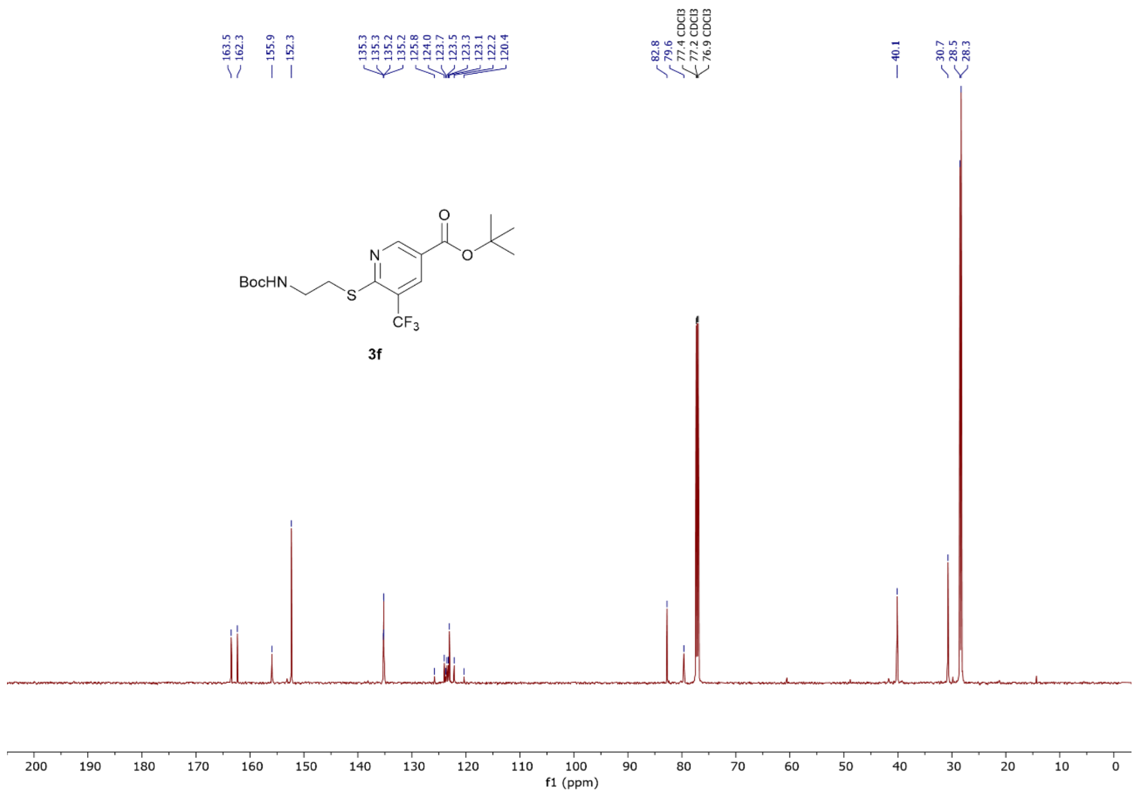

Figure S102. ^{13}C NMR spectrum of compound **3f** (151 MHz, $CDCl_3$).

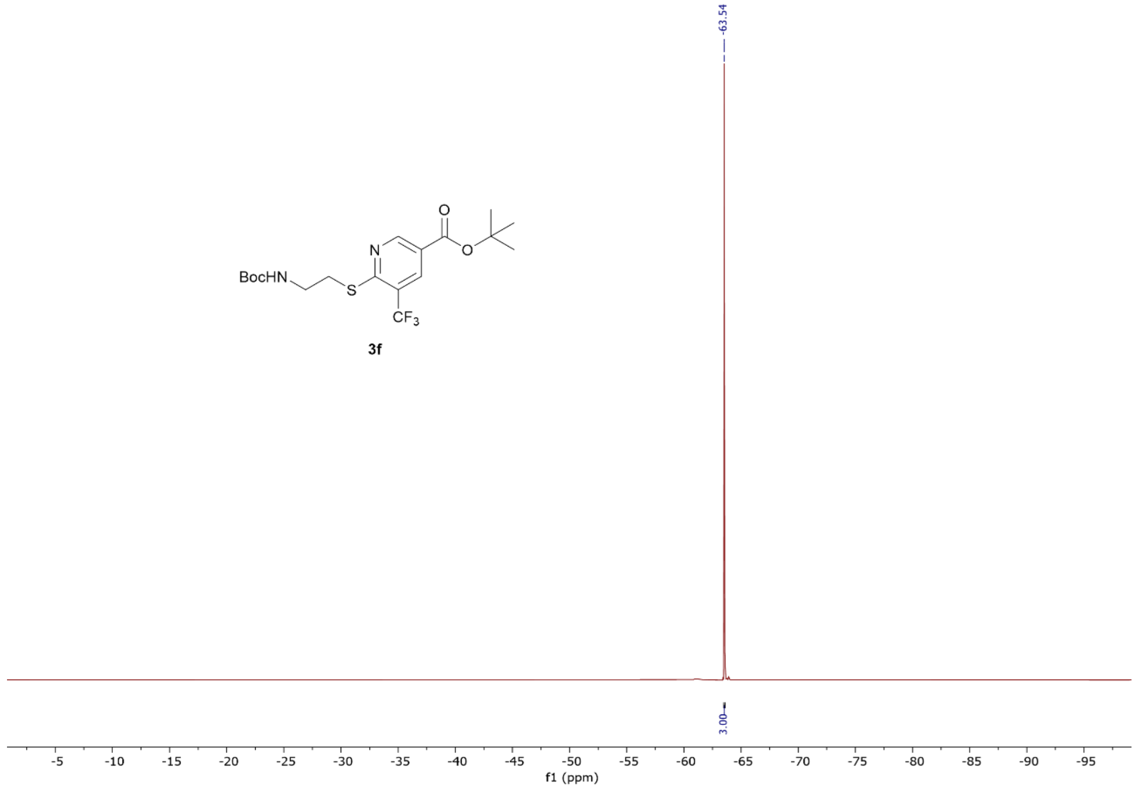

Figure S103. ^{19}F NMR spectrum of compound **3f** (564 MHz, $CDCl_3$).

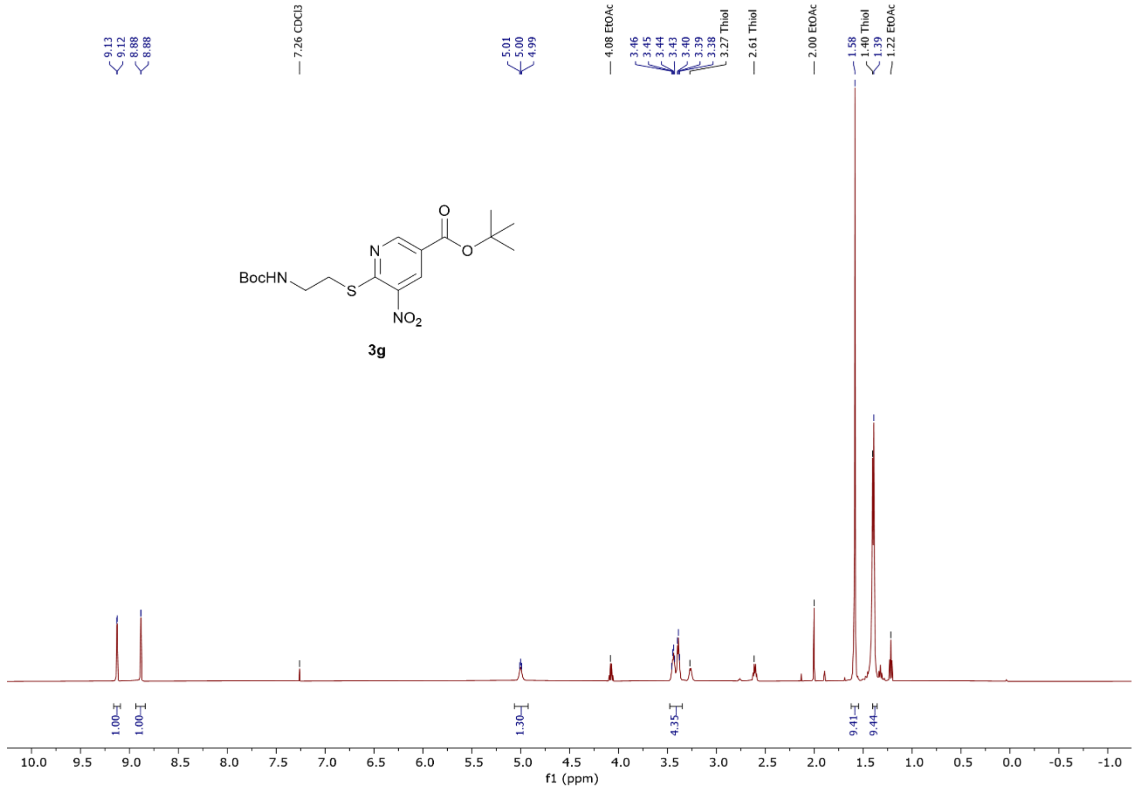

Figure S104. ^{1}H NMR spectrum of compound **3g** (600 MHz, $CDCl_3$).

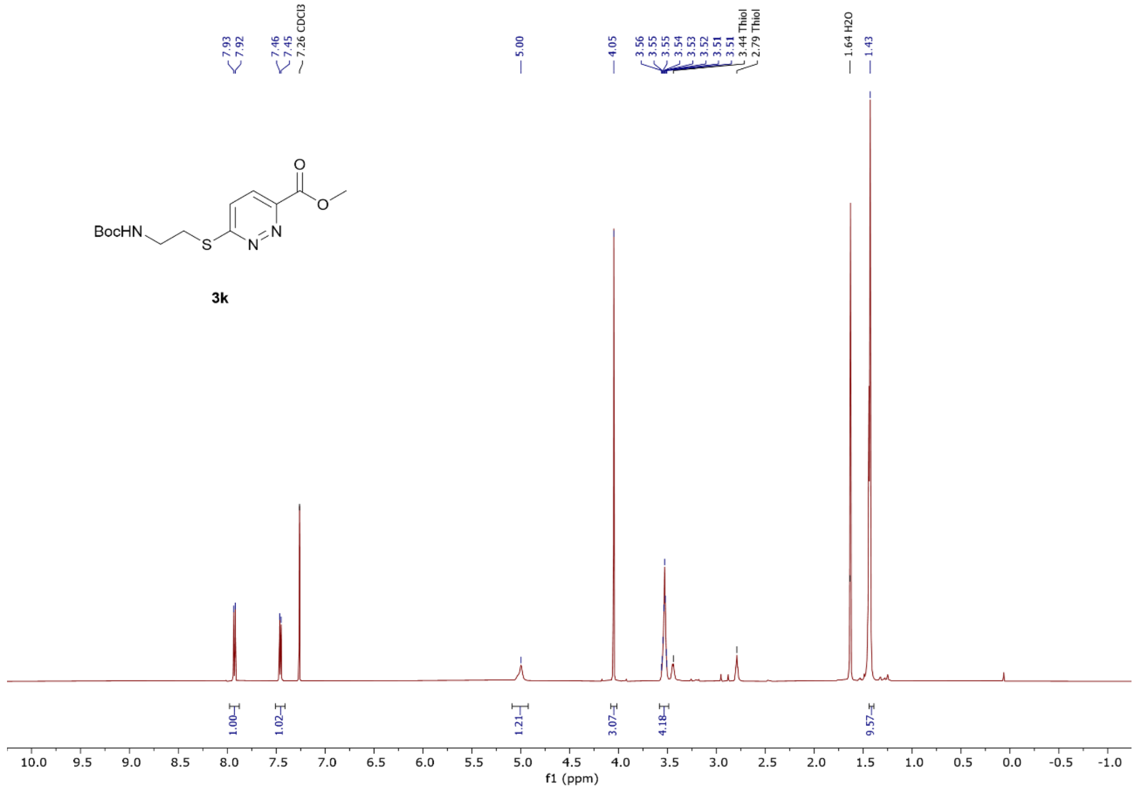

Figure S105. ^{1}H NMR spectrum of compound **3k** (600 MHz, $CDCl_3$).

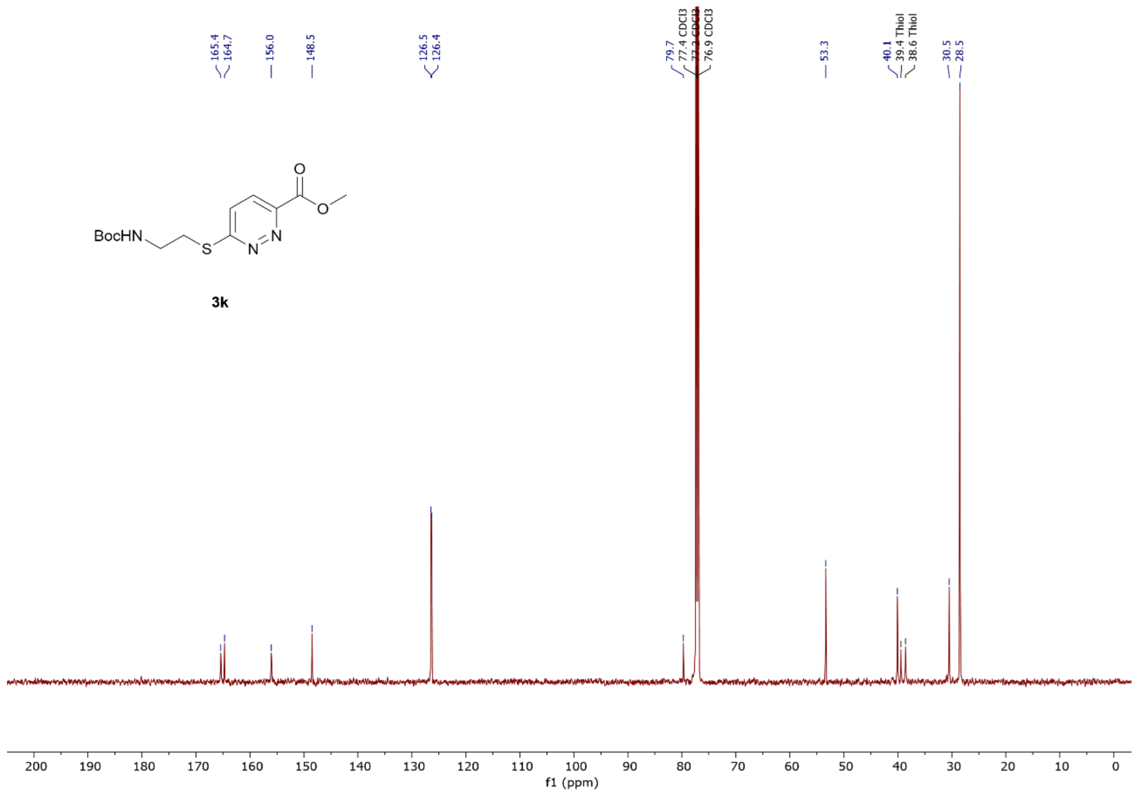

Figure S106. ^{13}C NMR spectrum of compound **3k** (151 MHz, $CDCl_3$).

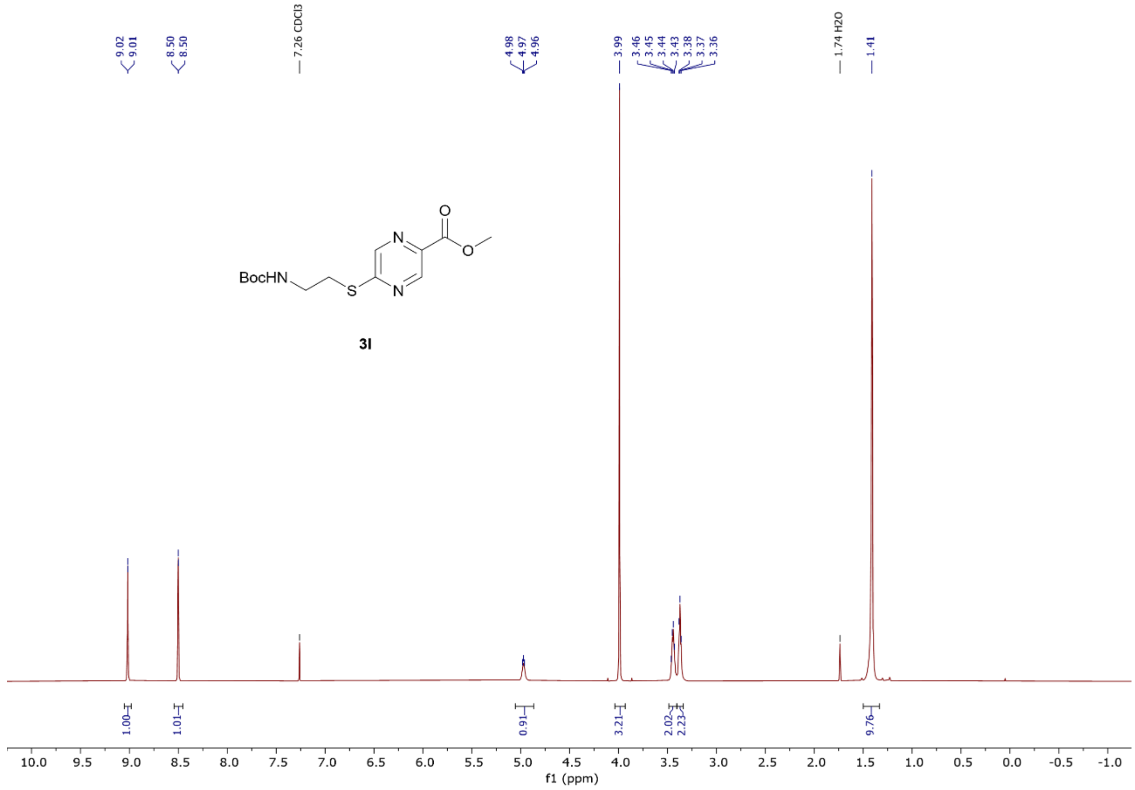

Figure S107. ^{1}H NMR spectrum of compound **3l** (600 MHz, $CDCl_3$).

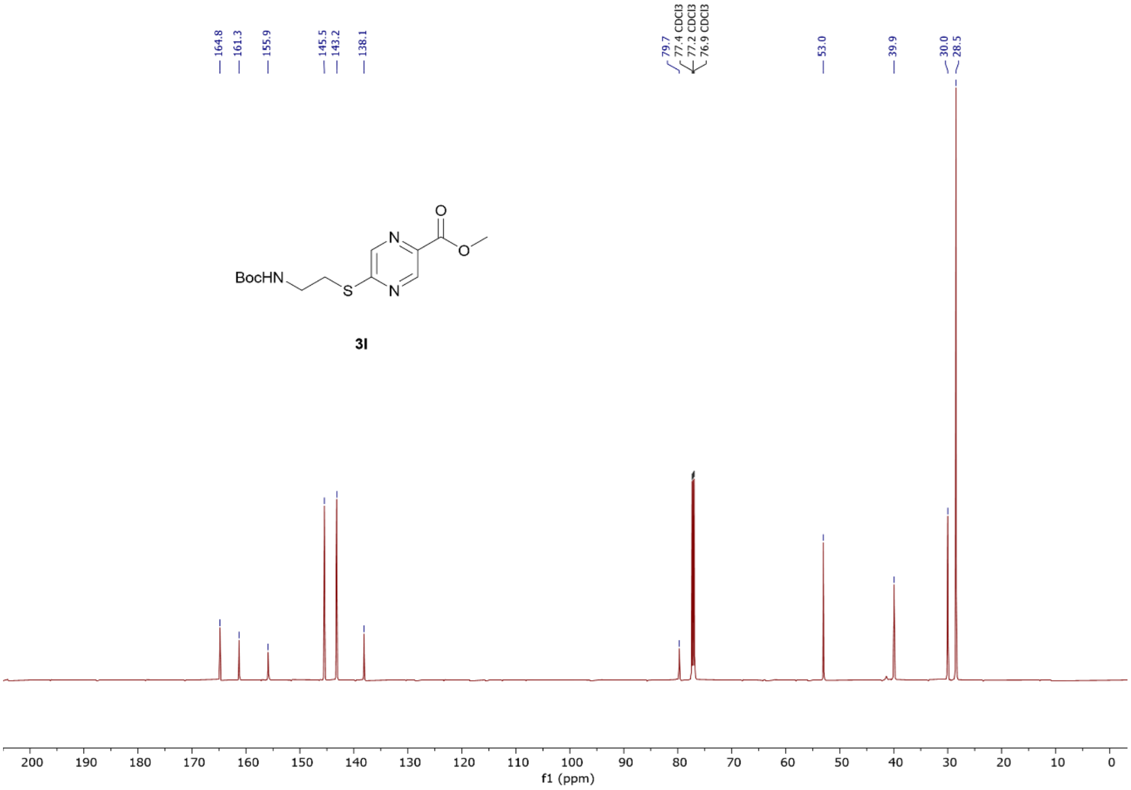

Figure S108. ^{13}C NMR spectrum of compound **3l** (151 MHz, $CDCl_3$).

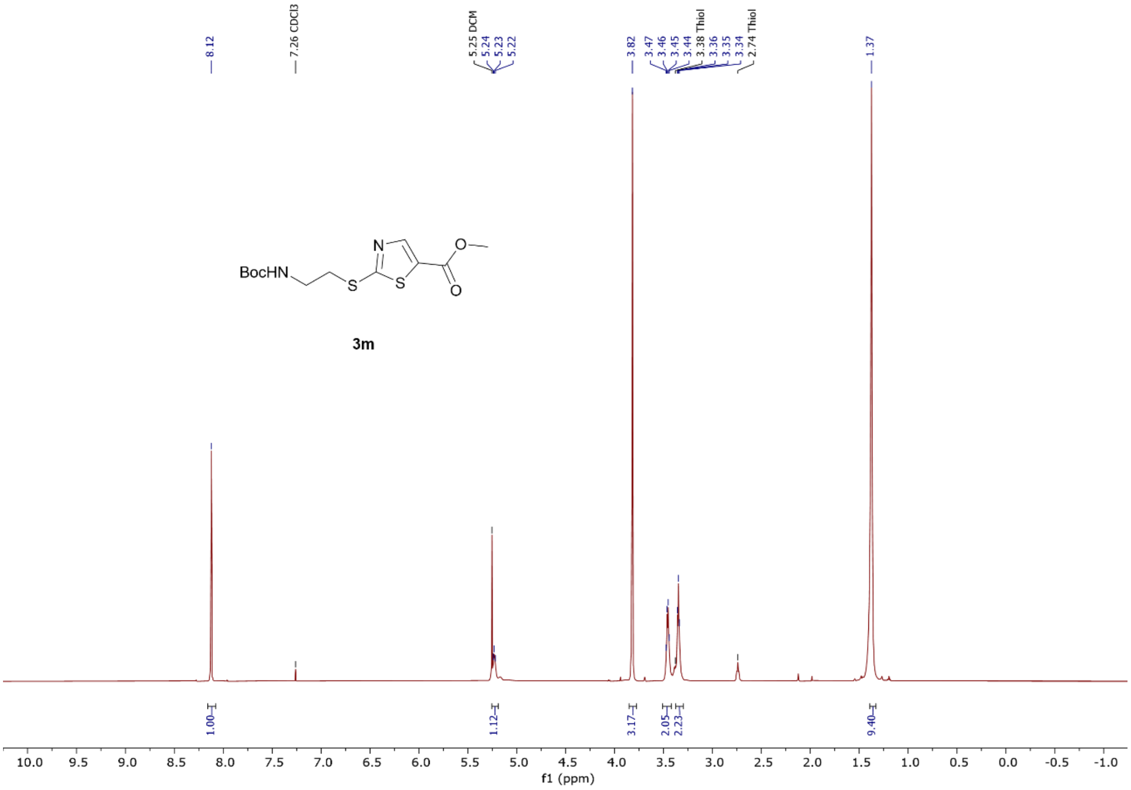

Figure S109. ^{1}H NMR spectrum of compound **3m** (600 MHz, $CDCl_3$).

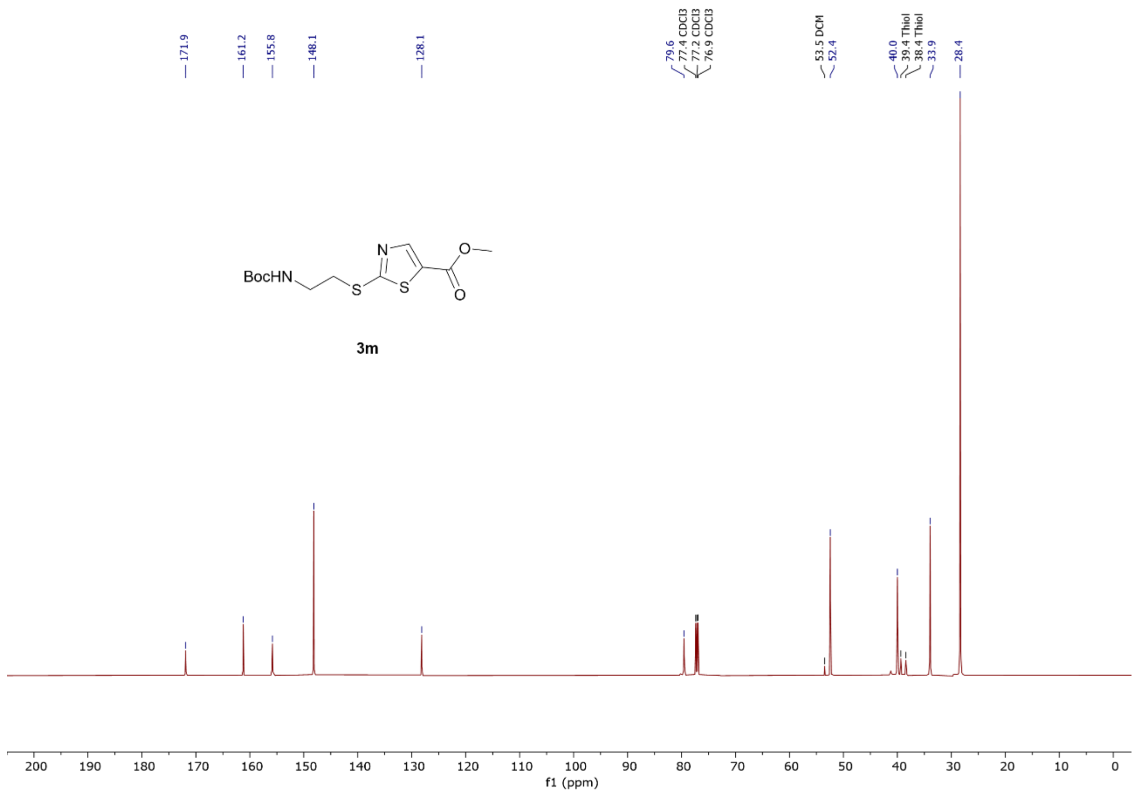

Figure S110. ^{13}C NMR spectrum of compound **3m** (151 MHz, $CDCl_3$).

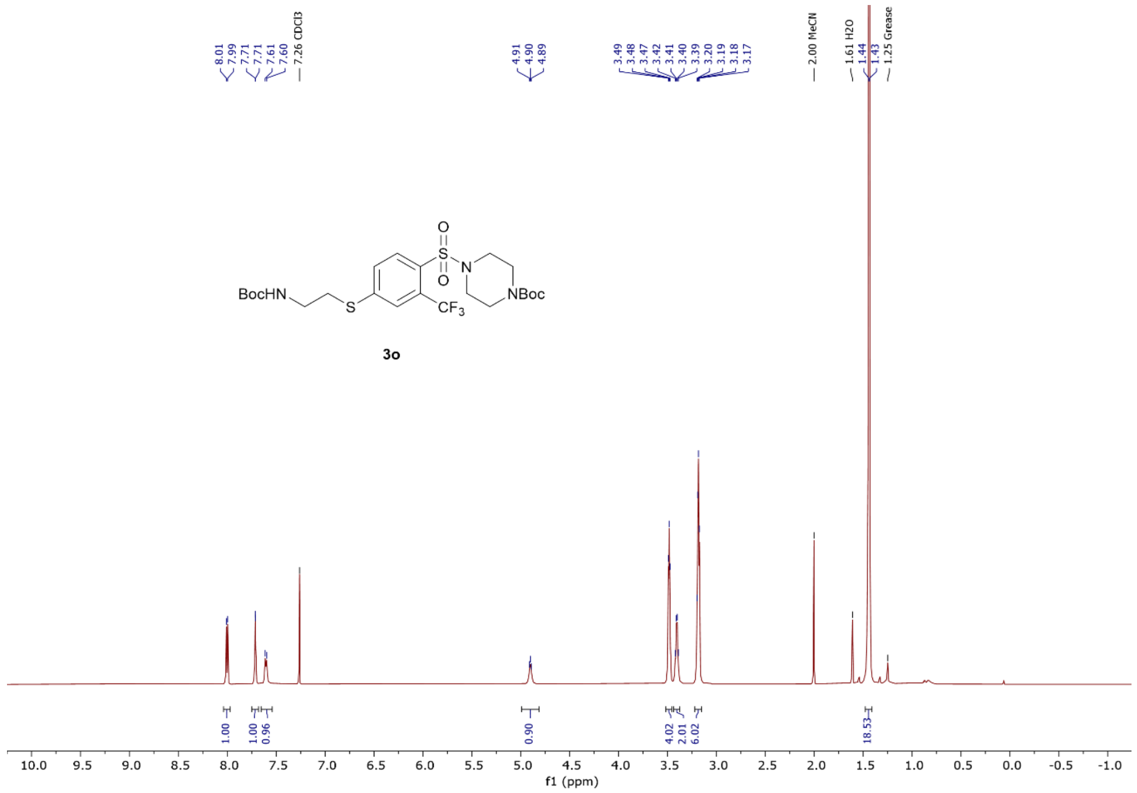

Figure S111. ^{1}H NMR spectrum of compound **3o** (600 MHz, $CDCl_3$).

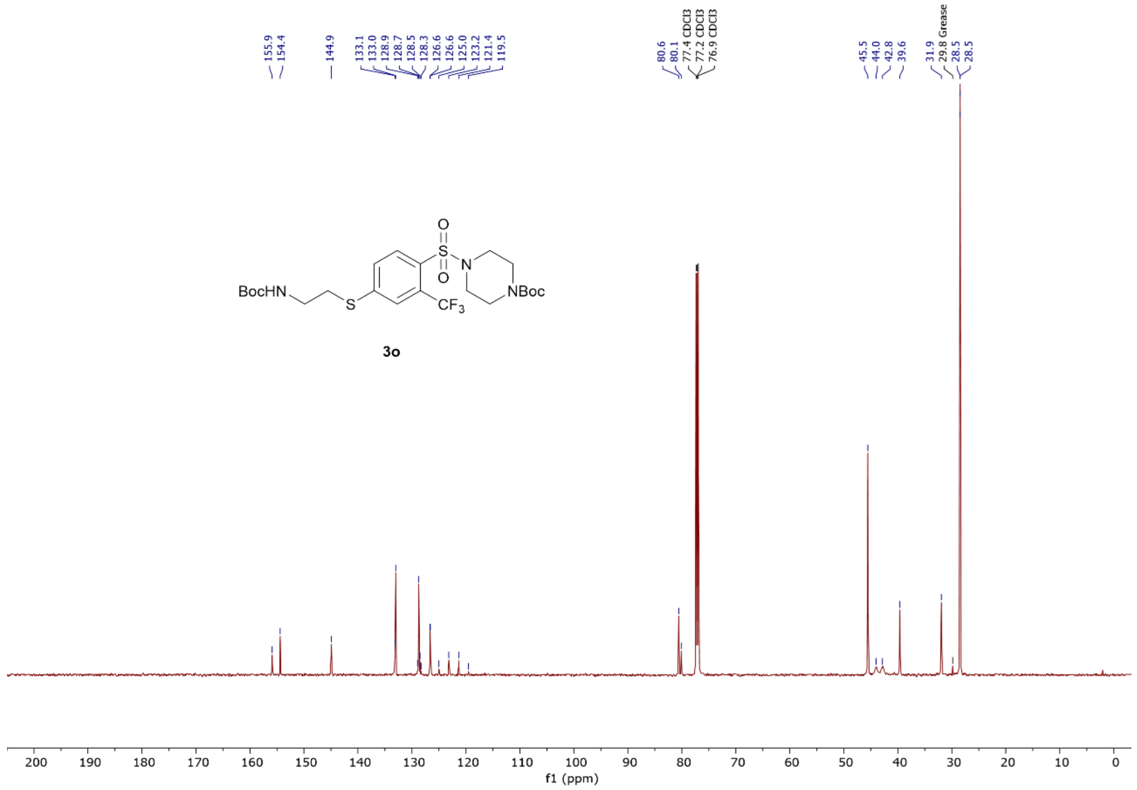

Figure S112. ^{13}C NMR spectrum of compound **3o** (151 MHz, $CDCl_3$).

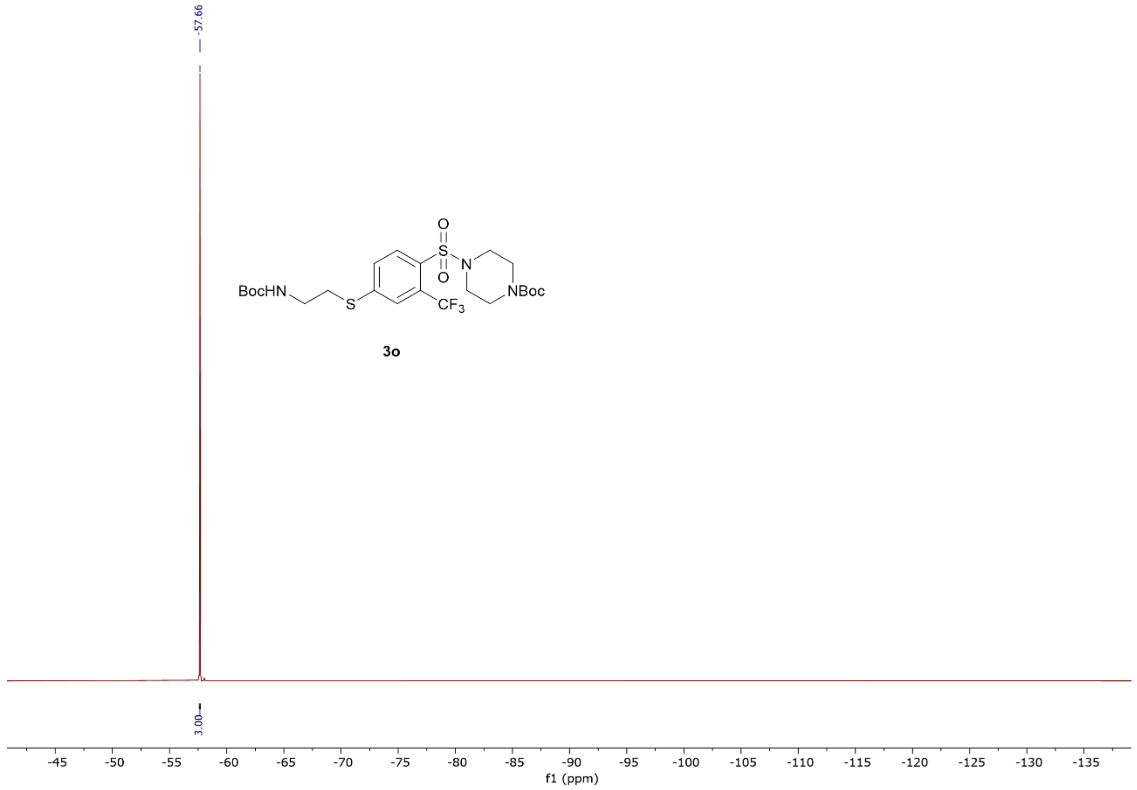

Figure S113. ^{19}F NMR spectrum of compound **3o** (564 MHz, $CDCl_3$).

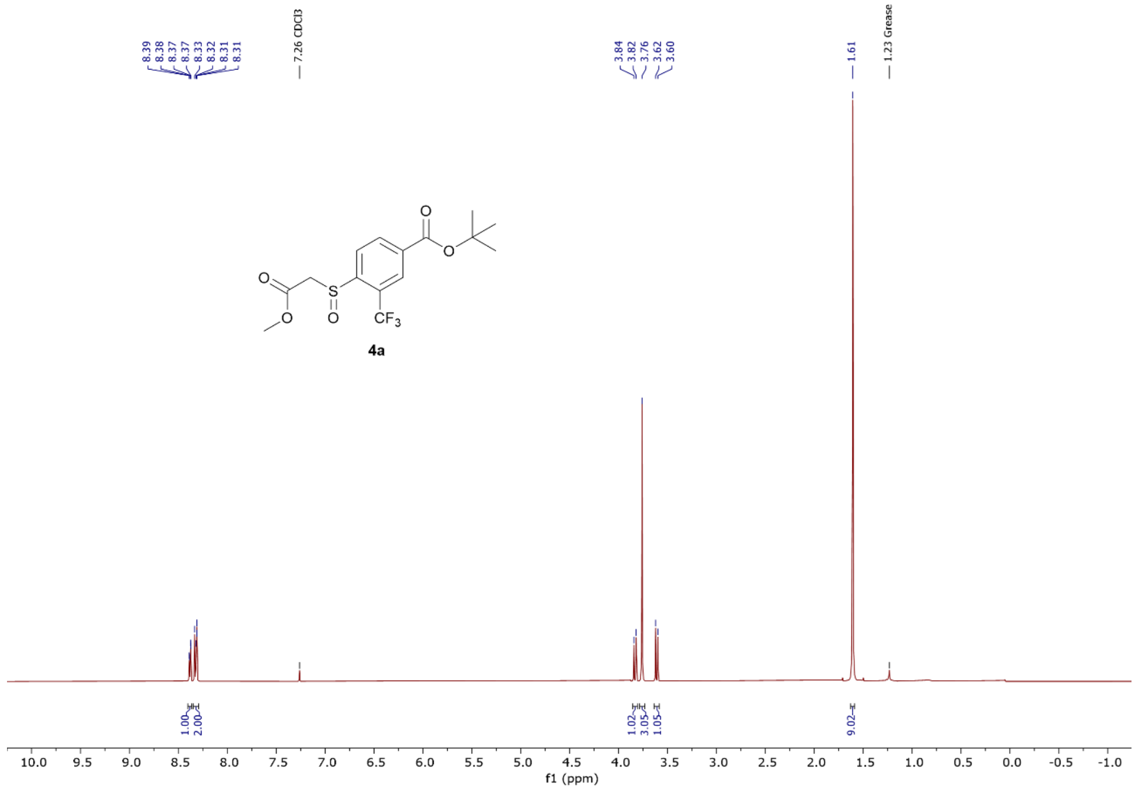

Figure S114. ^{1}H NMR spectrum of compound **4a** (600 MHz, $CDCl_3$).

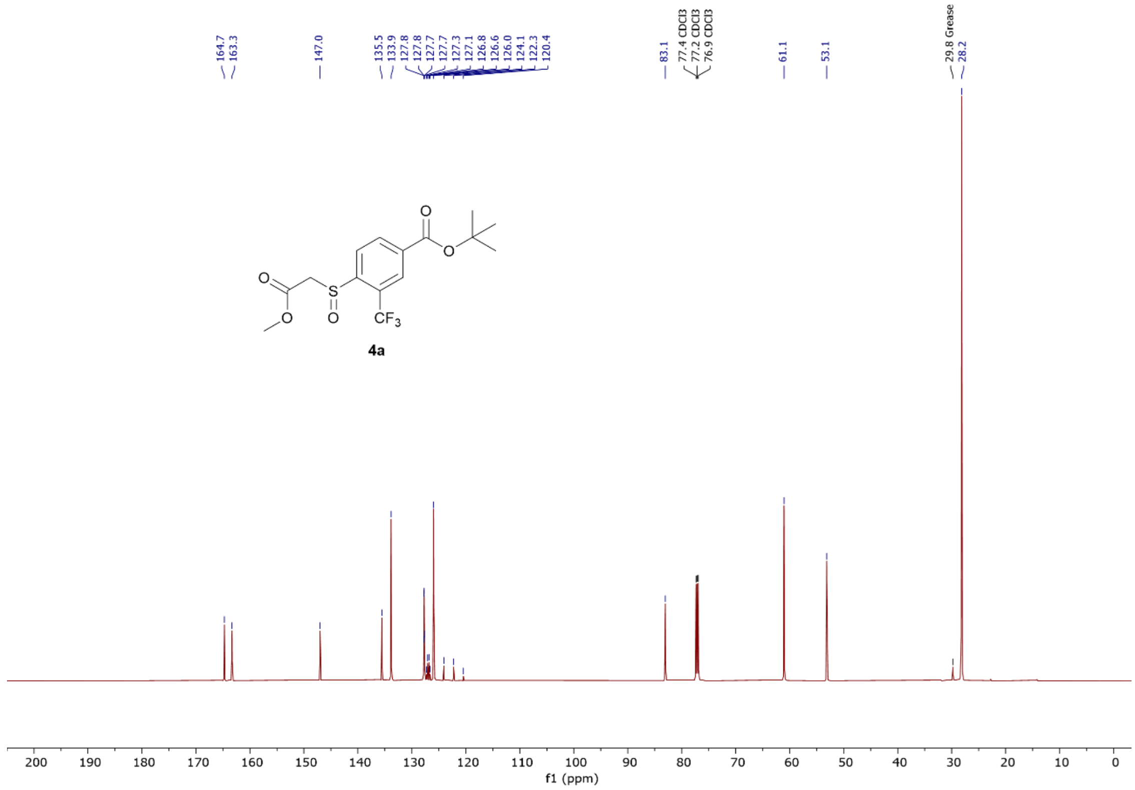

Figure S115. ^{13}C NMR spectrum of compound **4a** (151 MHz, $CDCl_3$).

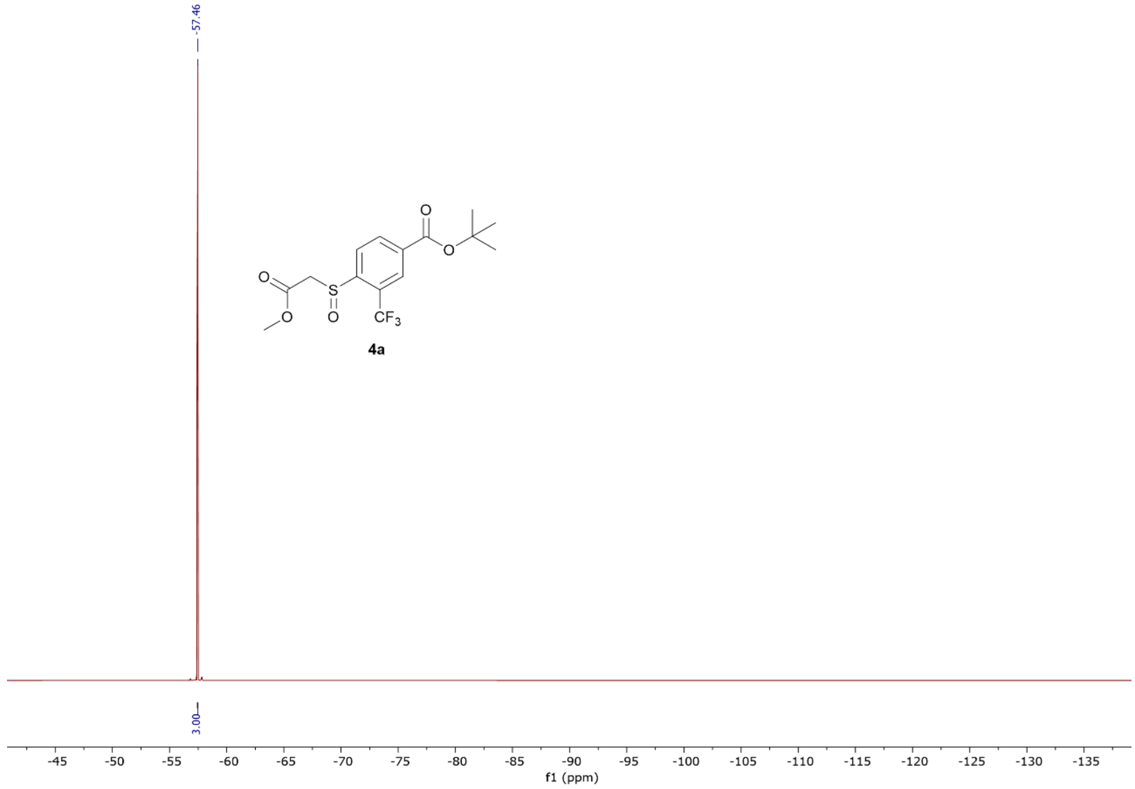

Figure S116. ^{19}F NMR spectrum of compound **4a** (564 MHz, $CDCl_3$).

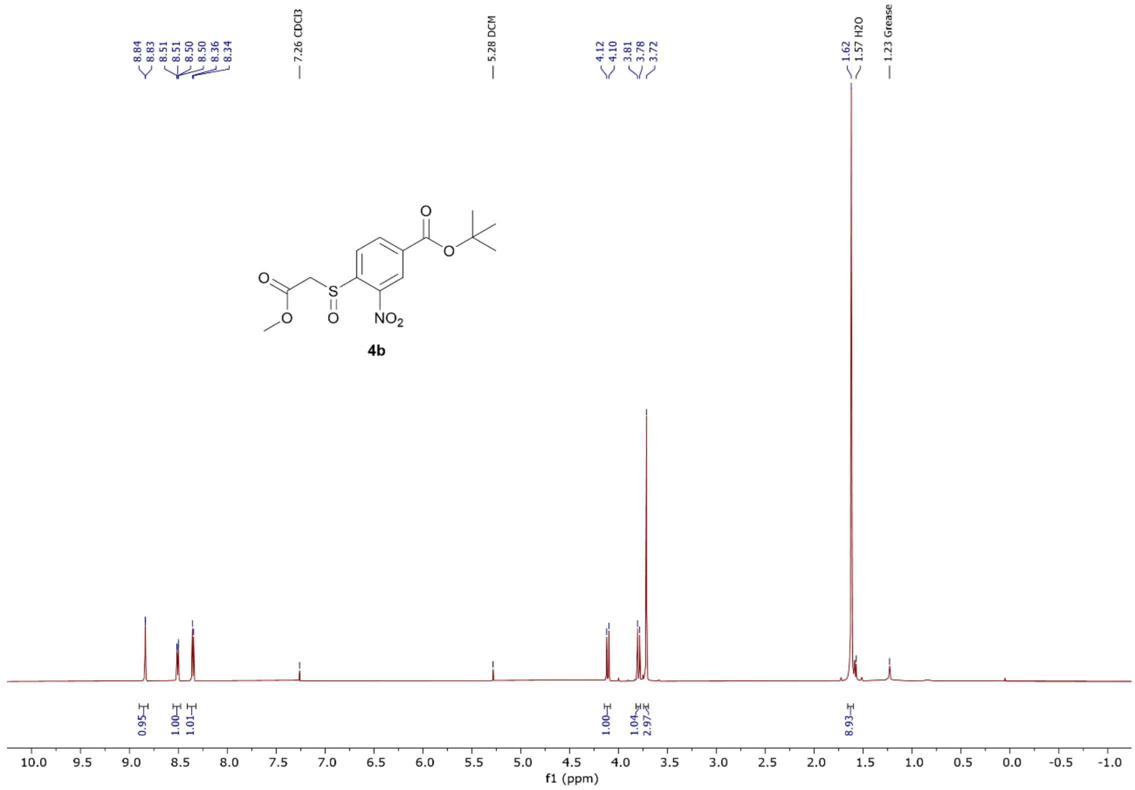

Figure S117. ^{1}H NMR spectrum of compound **4b** (600 MHz, $CDCl_3$).

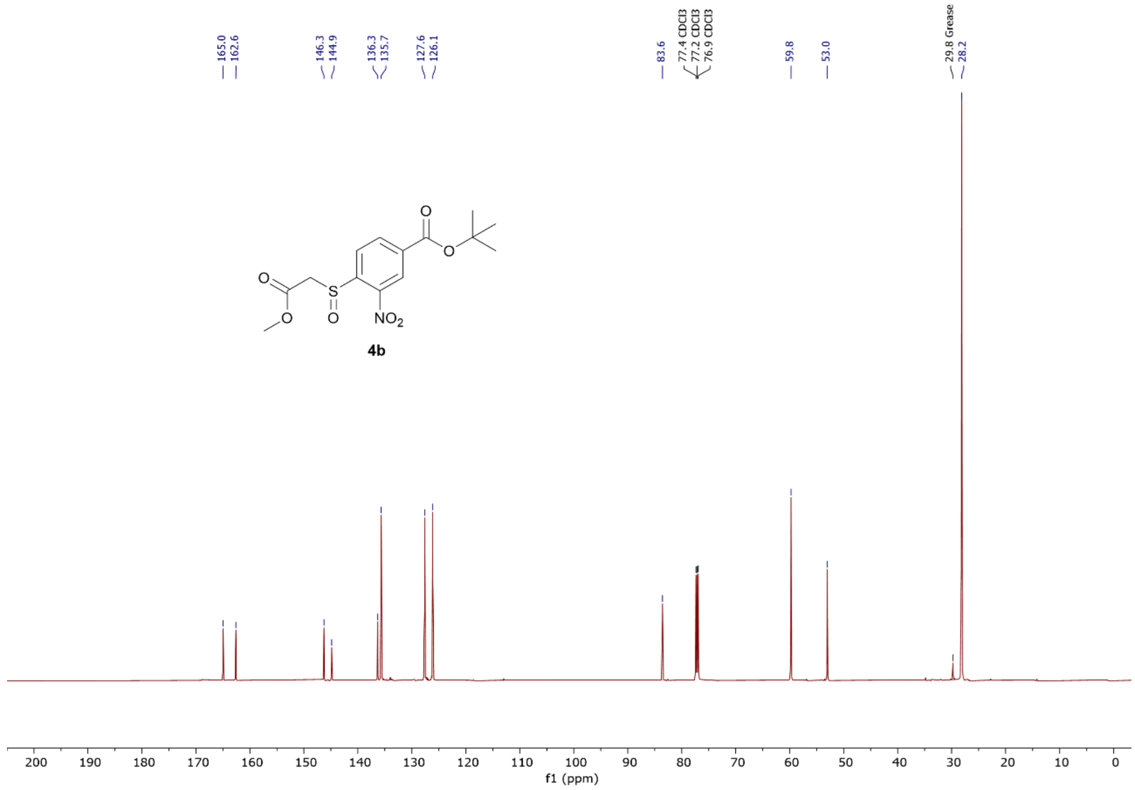

Figure S118. ^{13}C NMR spectrum of compound **4b** (151 MHz, $CDCl_3$).

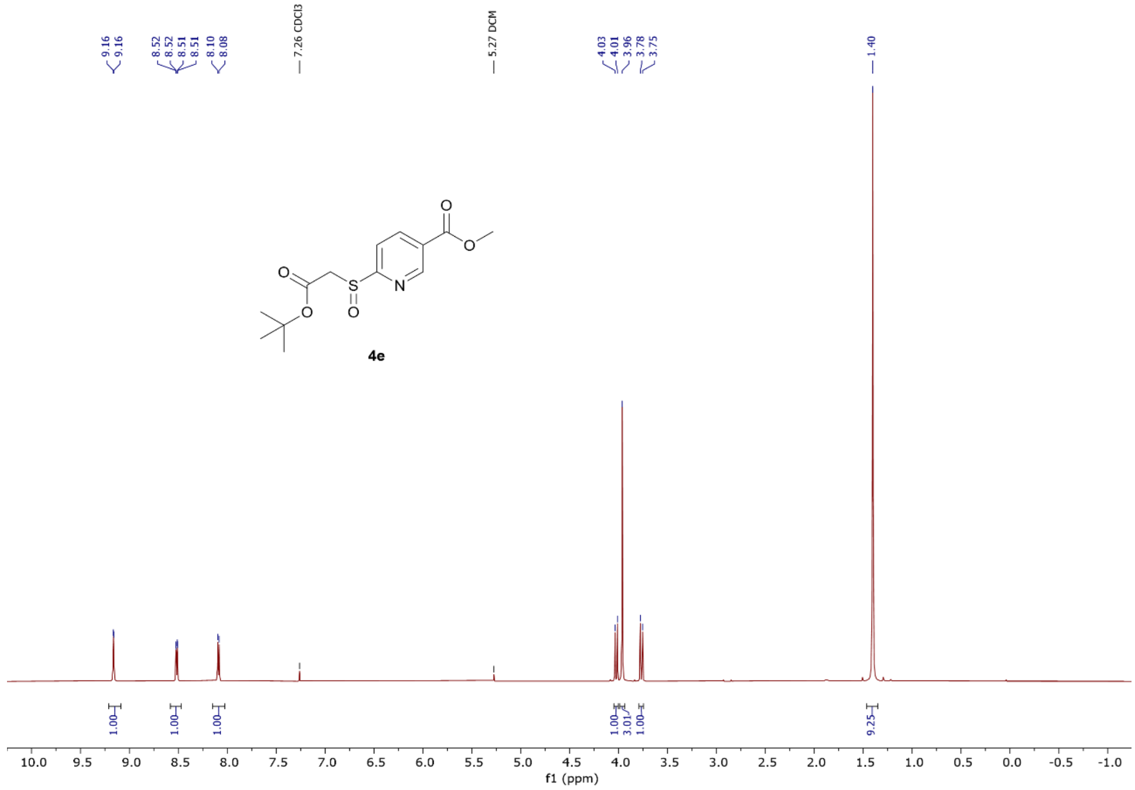

Figure S119. ^{1}H NMR spectrum of compound **4e** (600 MHz, $CDCl_3$).

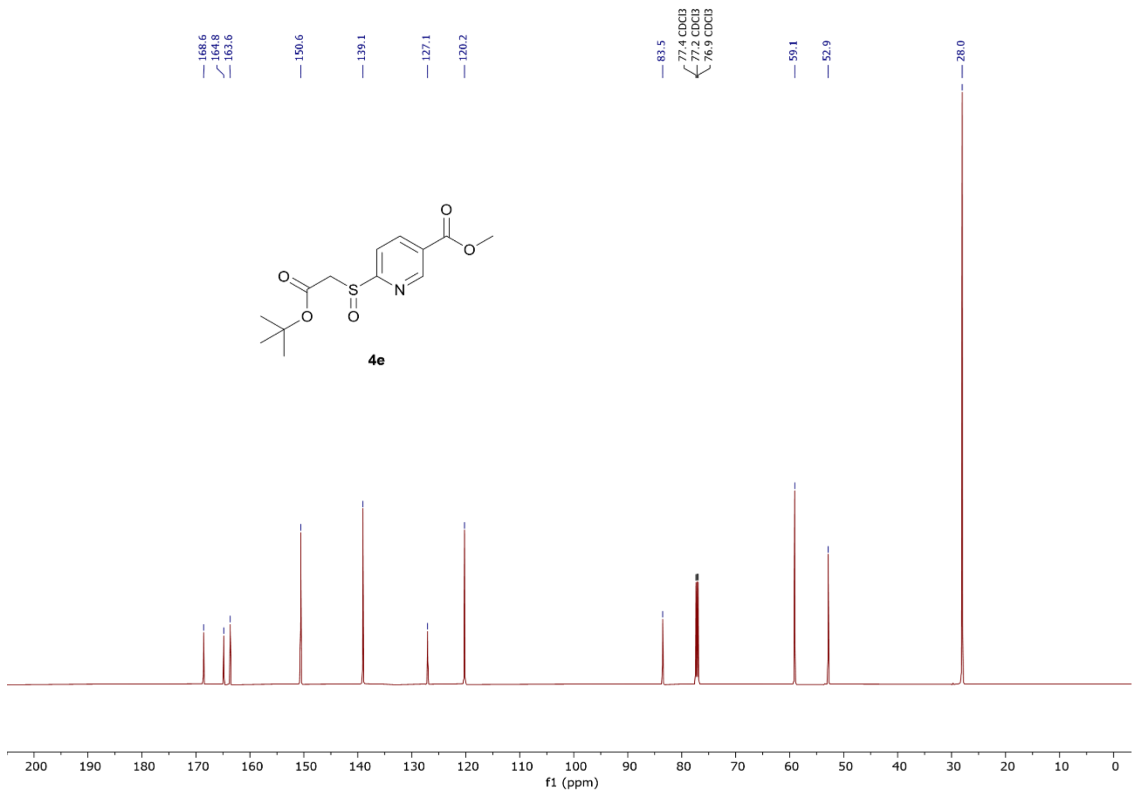

Figure S120. ^{13}C NMR spectrum of compound **4e** (151 MHz, $CDCl_3$).

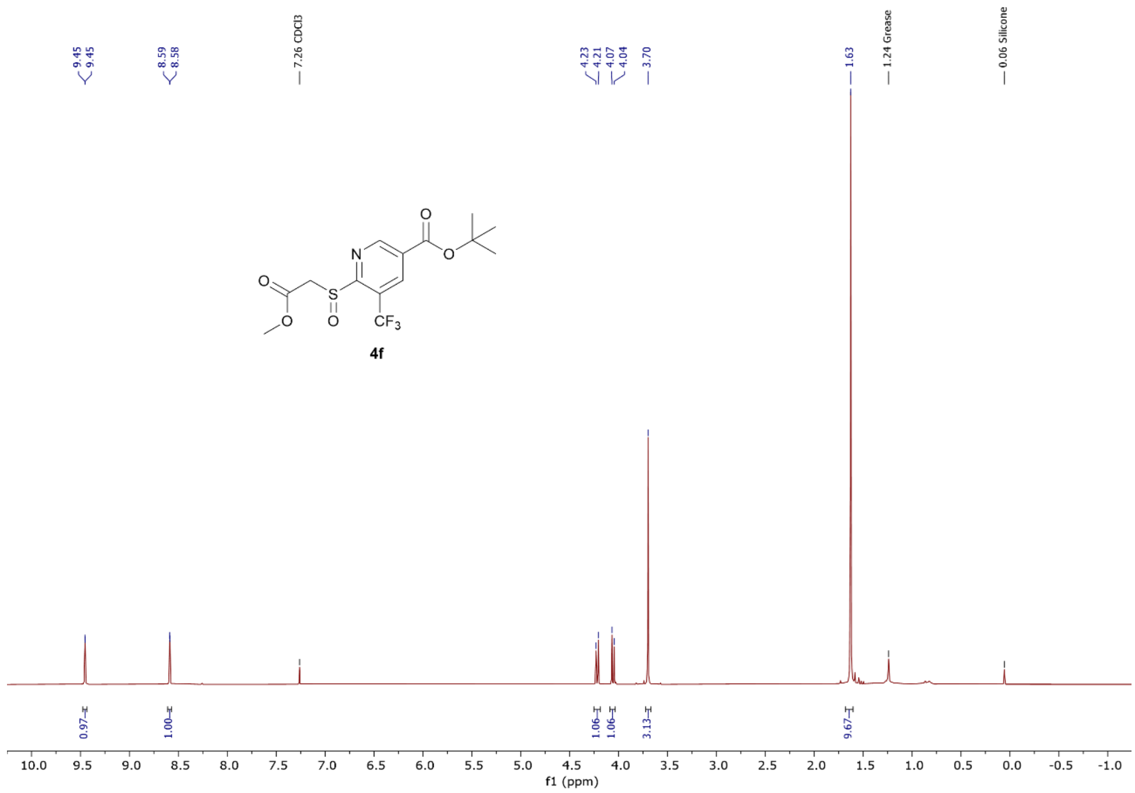

Figure S121. ^{1}H NMR spectrum of compound **4f** (600 MHz, $CDCl_3$).

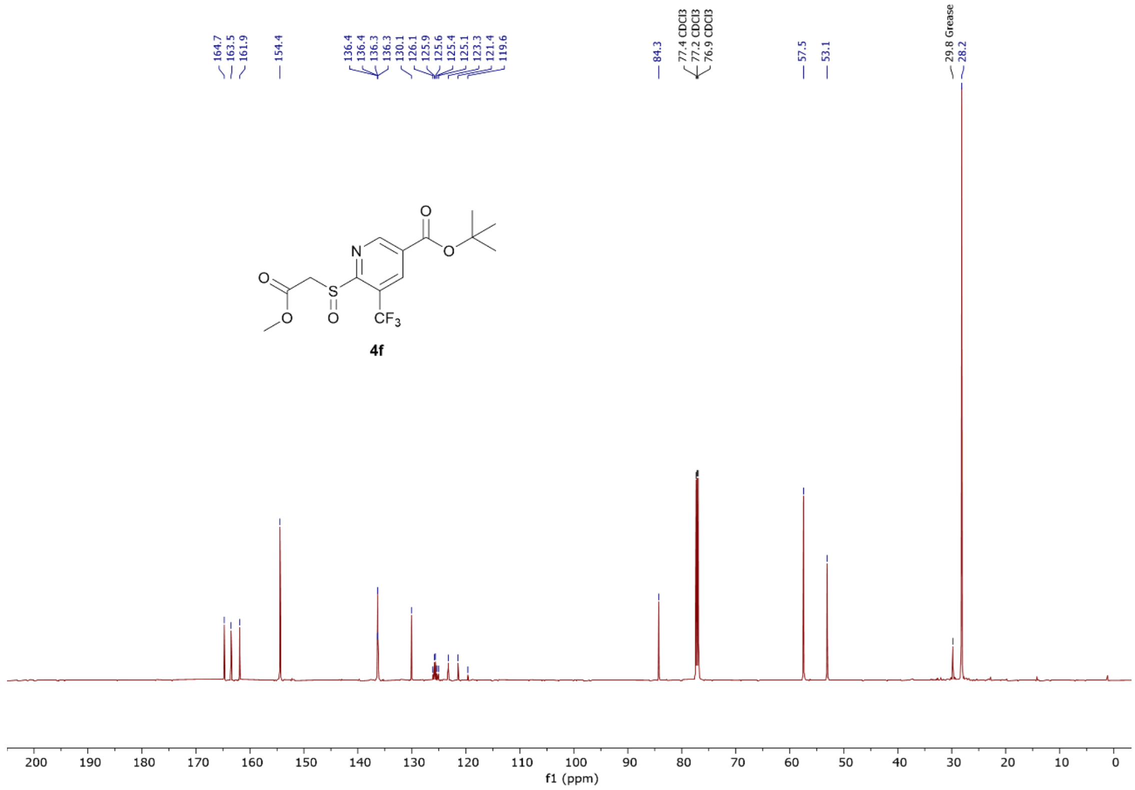

Figure S122. ^{13}C NMR spectrum of compound **4f** (151 MHz, $CDCl_3$).

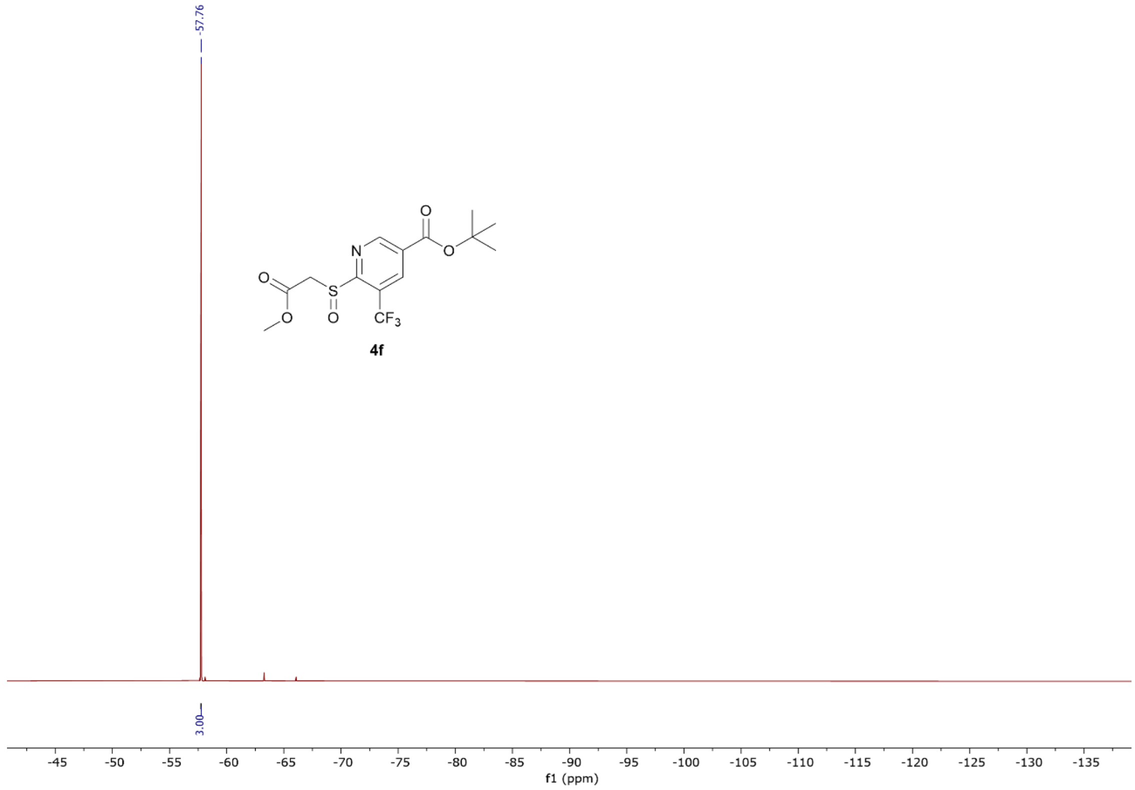

Figure S123. ^{19}F NMR spectrum of compound **4f** (564 MHz, $CDCl_3$).

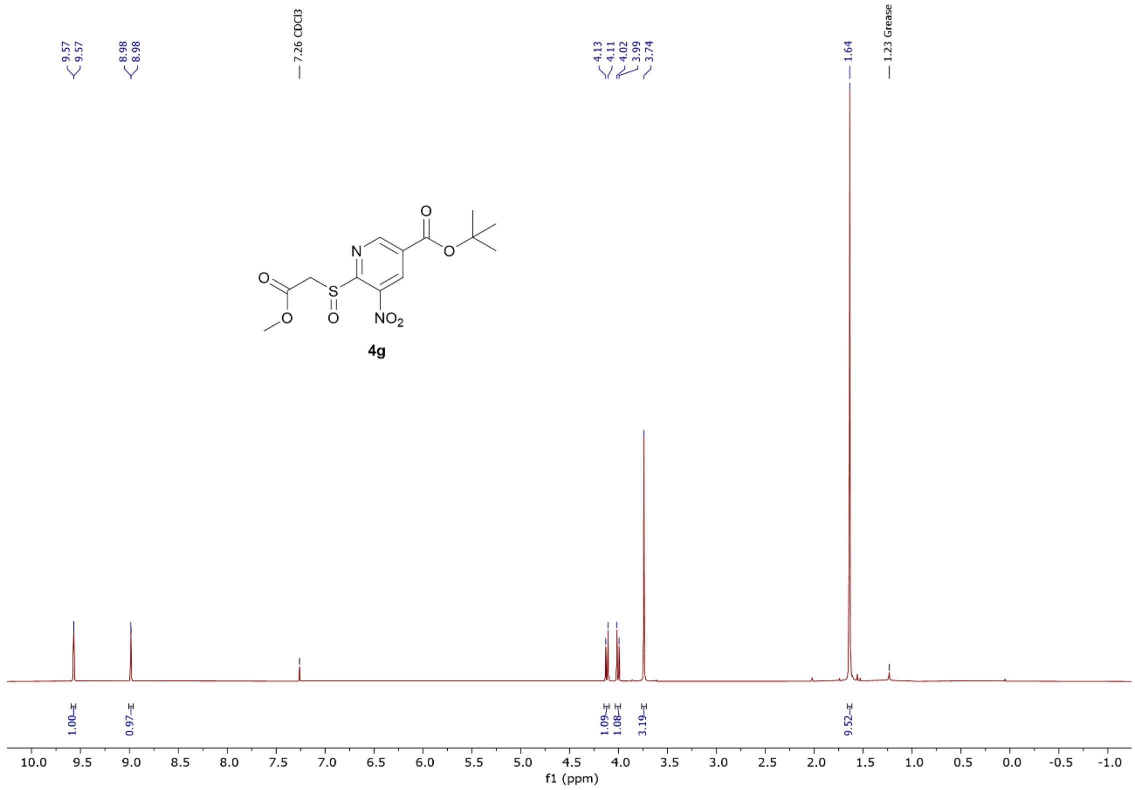

Figure S124. ^{1}H NMR spectrum of compound **4g** (600 MHz, $CDCl_3$).

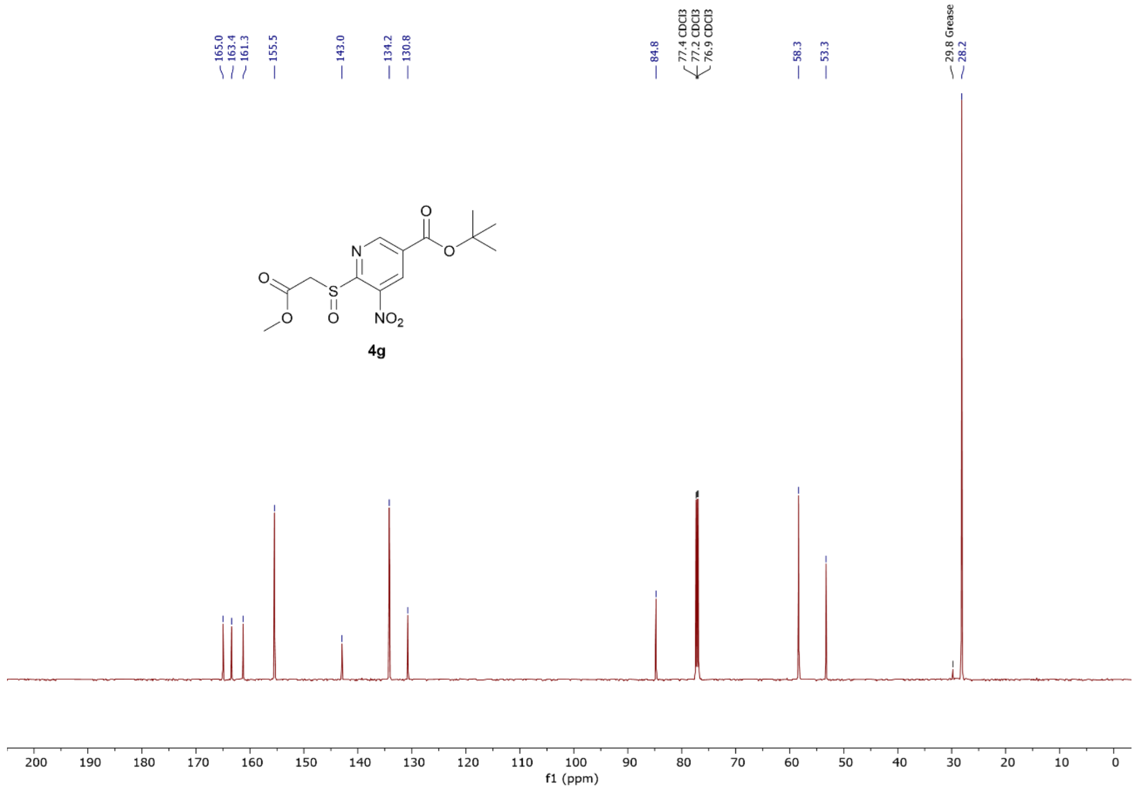

Figure S125. ^{13}C NMR spectrum of compound **4g** (151 MHz, $CDCl_3$).

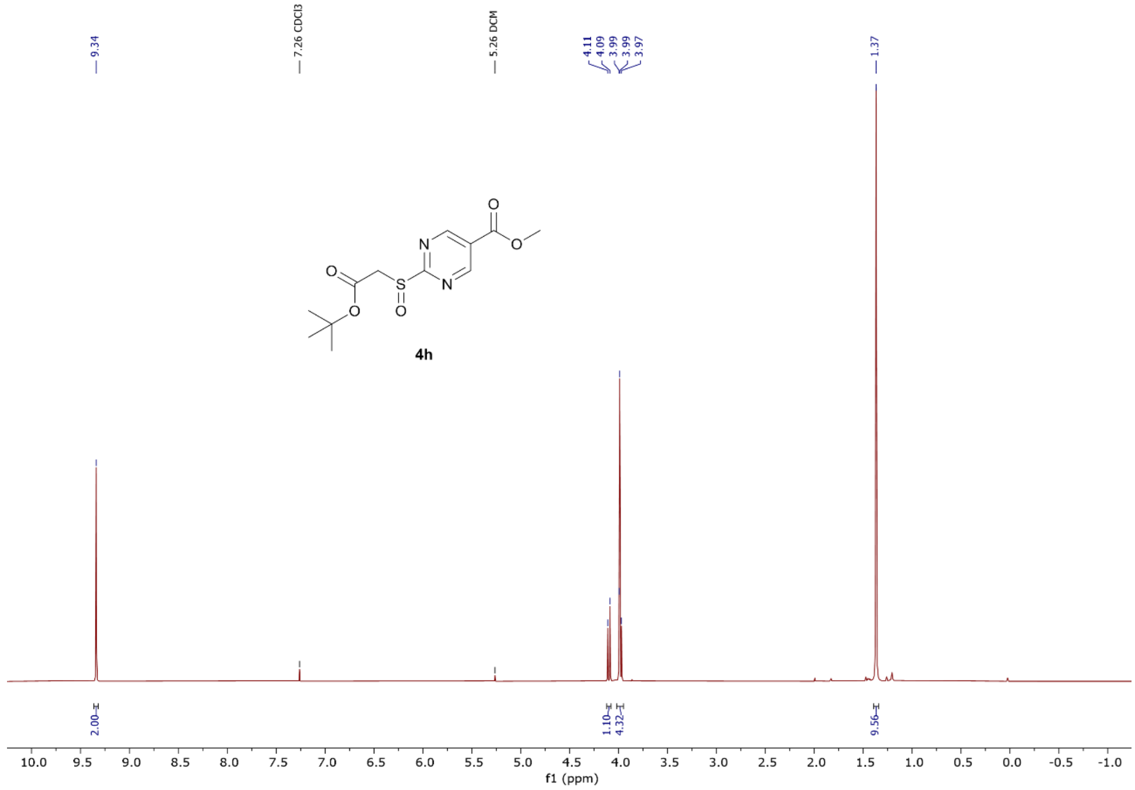

Figure S126. ^{1}H NMR spectrum of compound **4h** (600 MHz, $CDCl_3$).

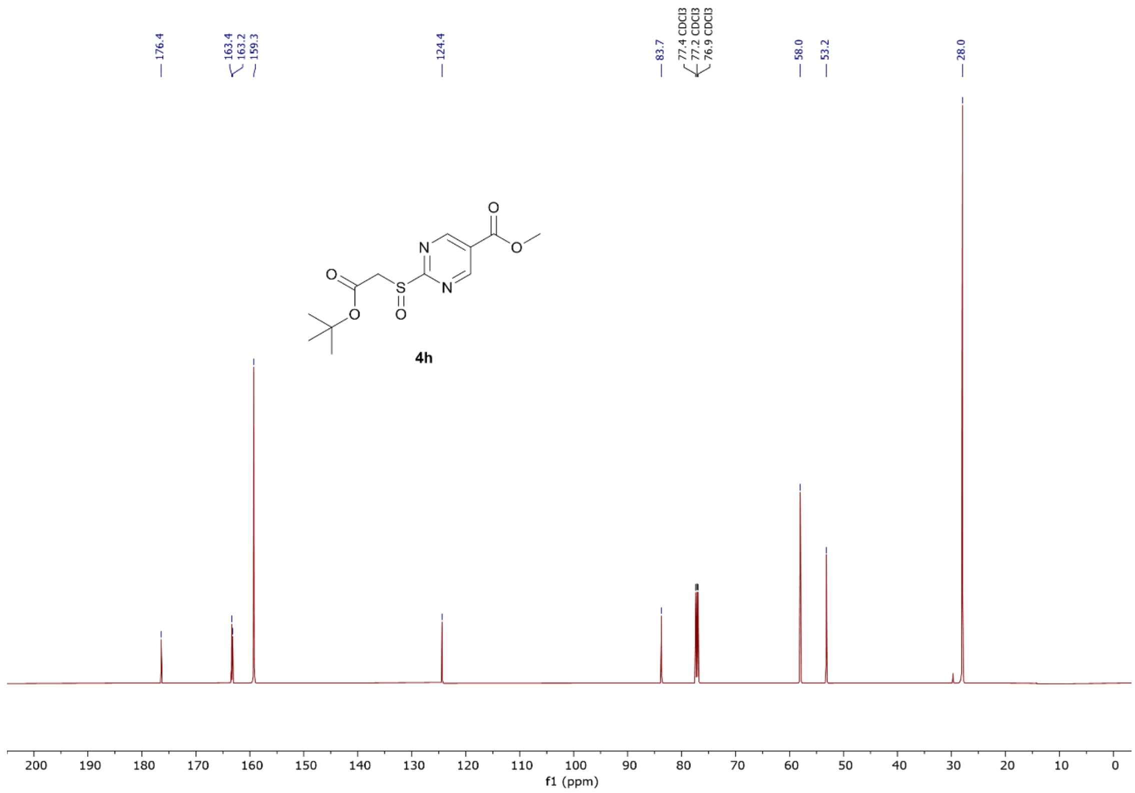

Figure S127. ^{13}C NMR spectrum of compound **4h** (151 MHz, $CDCl_3$).

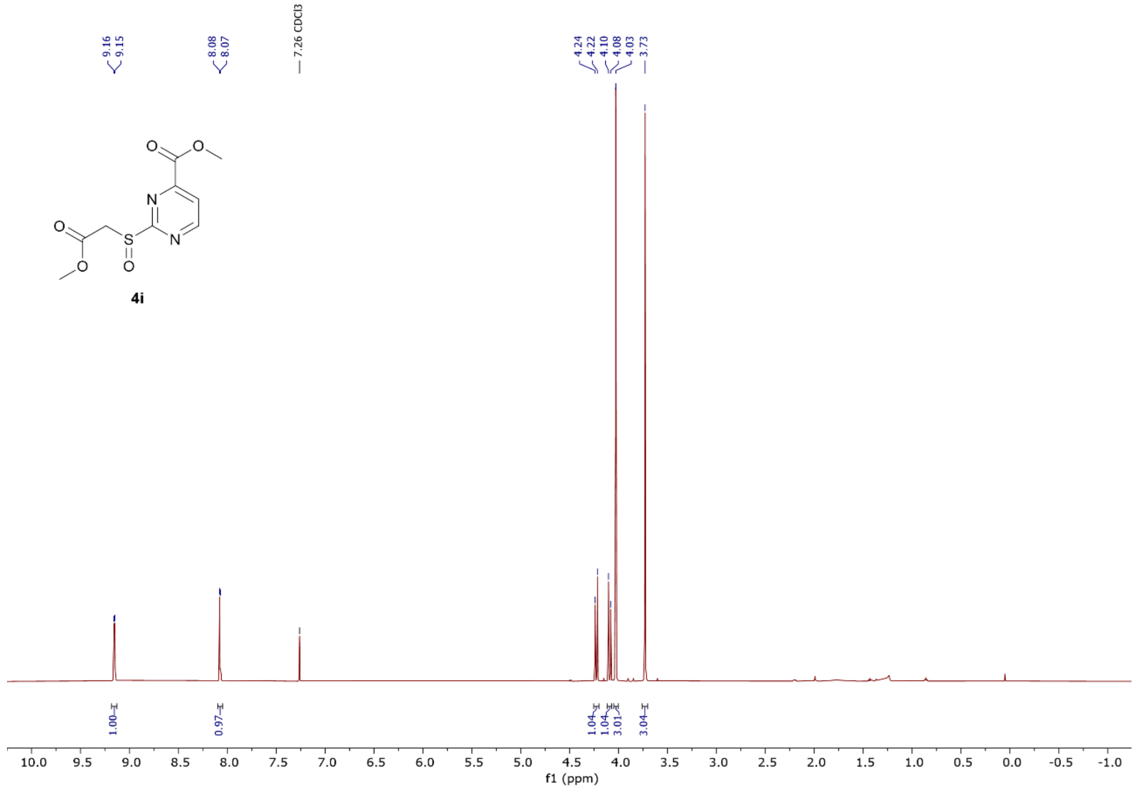

Figure S128. ^{1}H NMR spectrum of compound **4i** (600 MHz, $CDCl_3$).

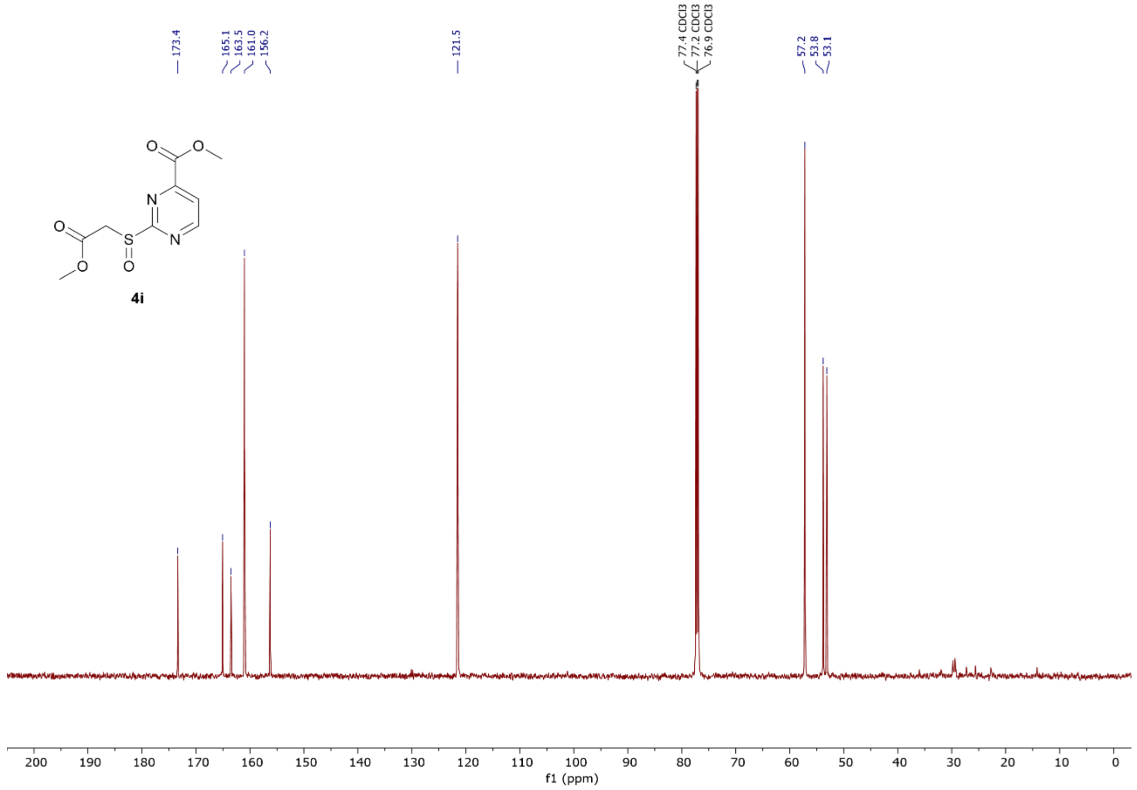

Figure S129. ^{13}C NMR spectrum of compound **4i** (151 MHz, $CDCl_3$).

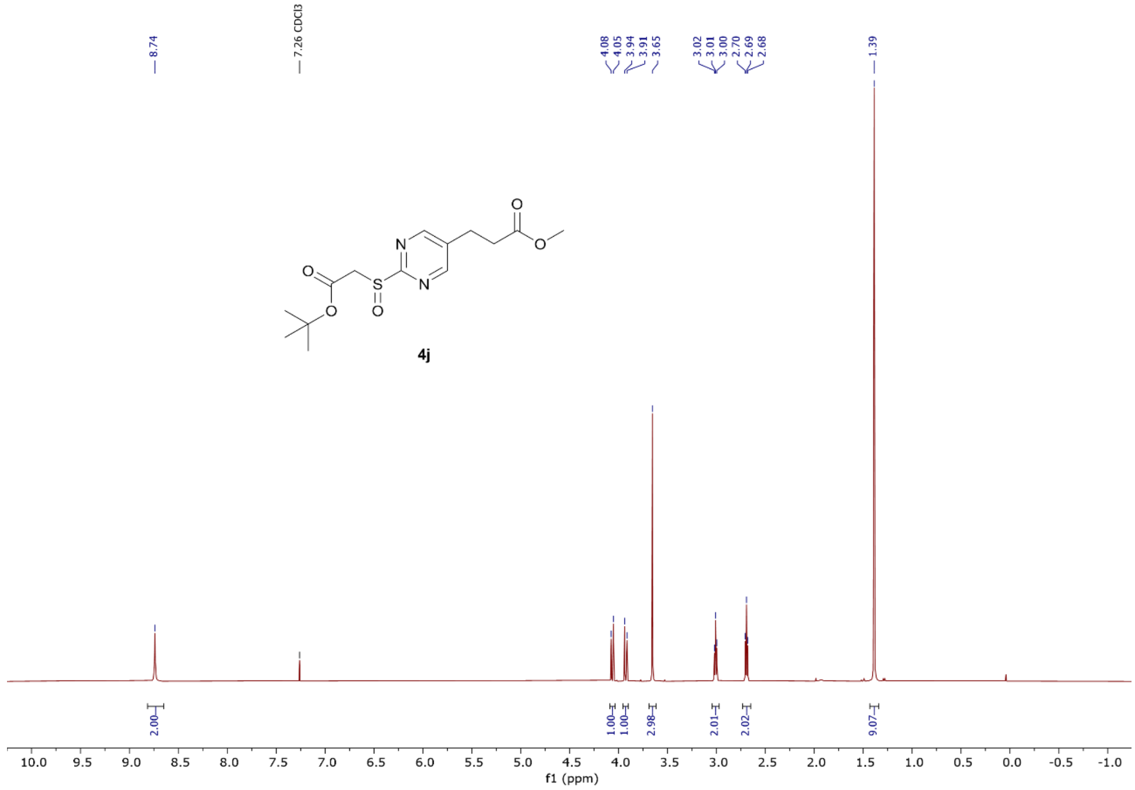

Figure S130. ^{1}H NMR spectrum of compound **4j** (600 MHz, $CDCl_3$).

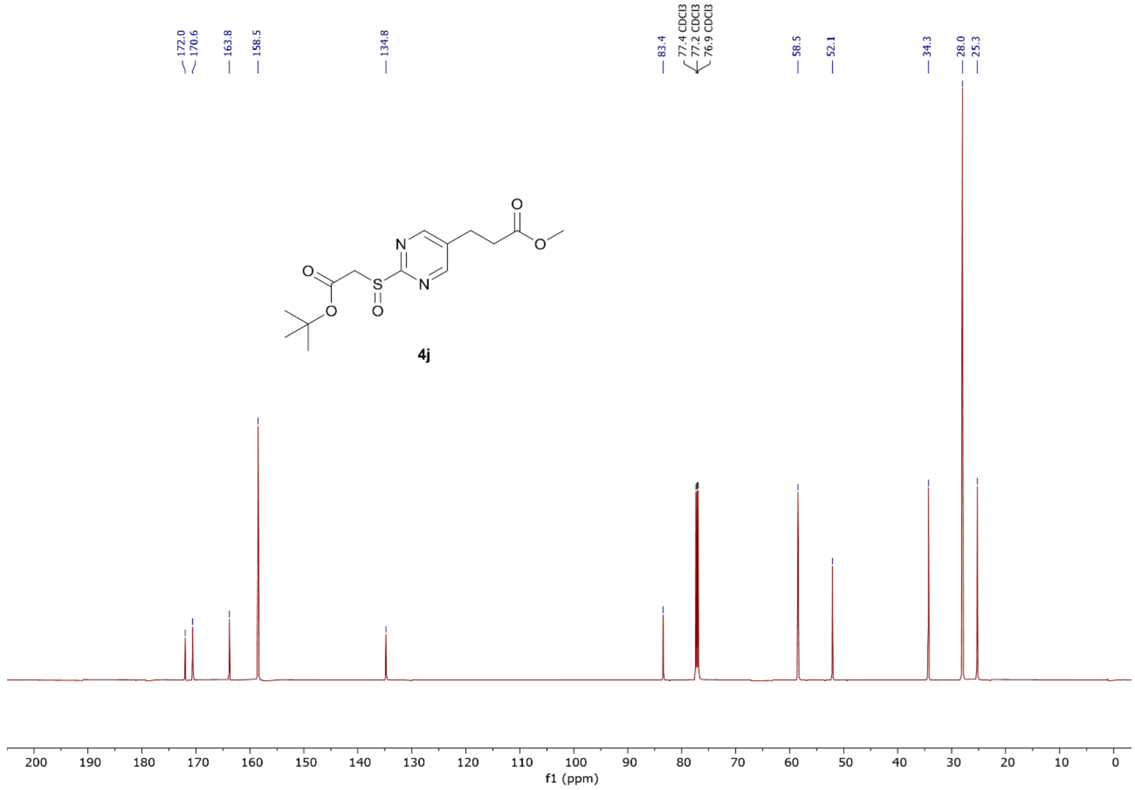

Figure S131. ^{13}C NMR spectrum of compound **4j** (151 MHz, $CDCl_3$).

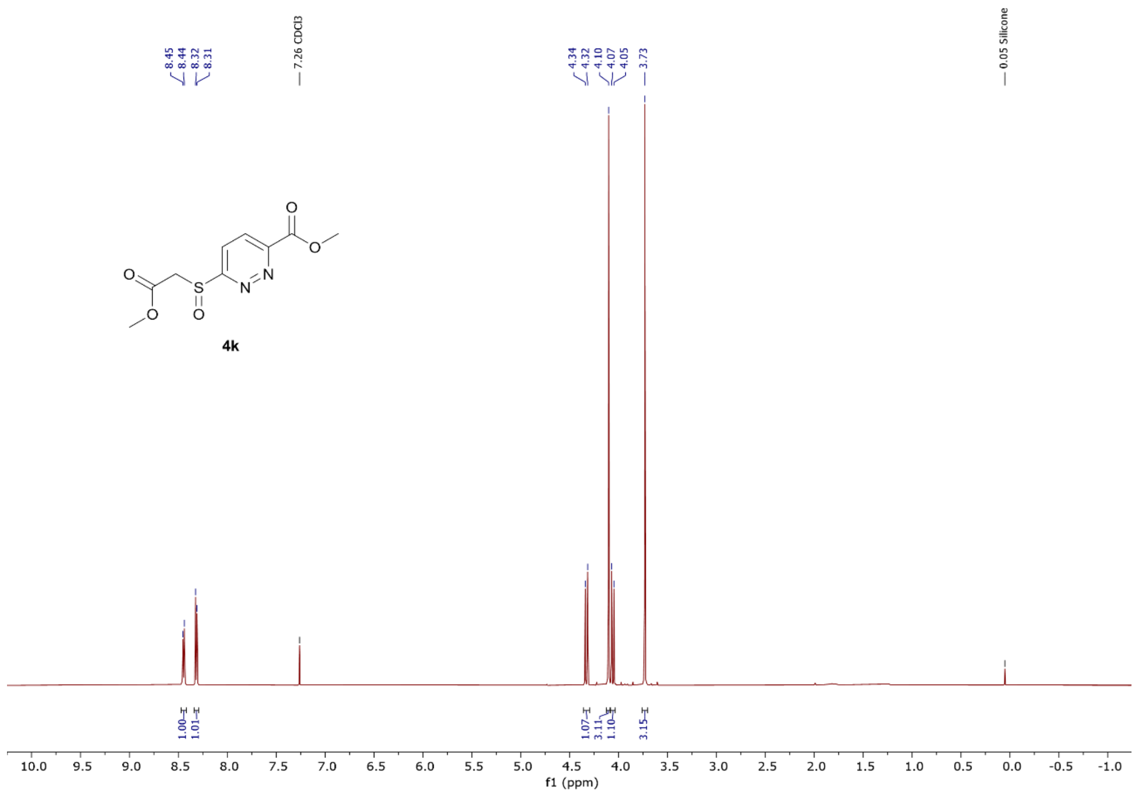

Figure S132. ^{1}H NMR spectrum of compound **4k** (600 MHz, $CDCl_3$).

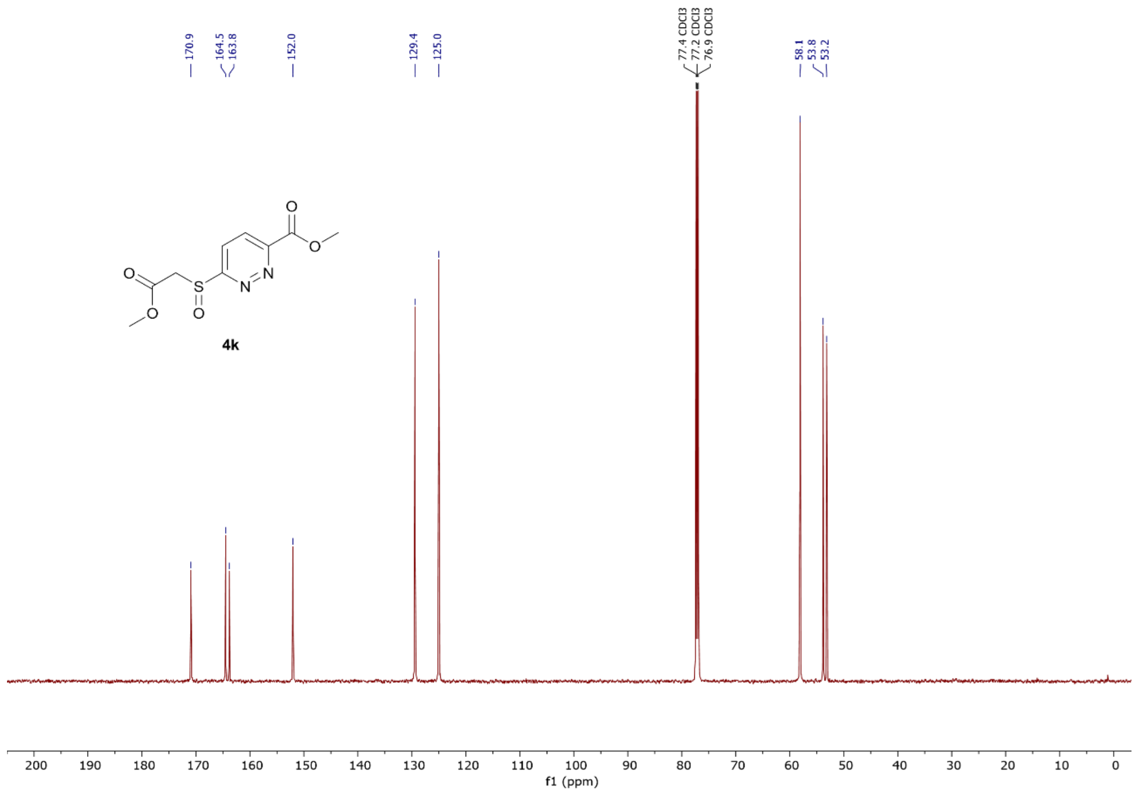

Figure S133. ^{13}C NMR spectrum of compound **4k** (151 MHz, $CDCl_3$).

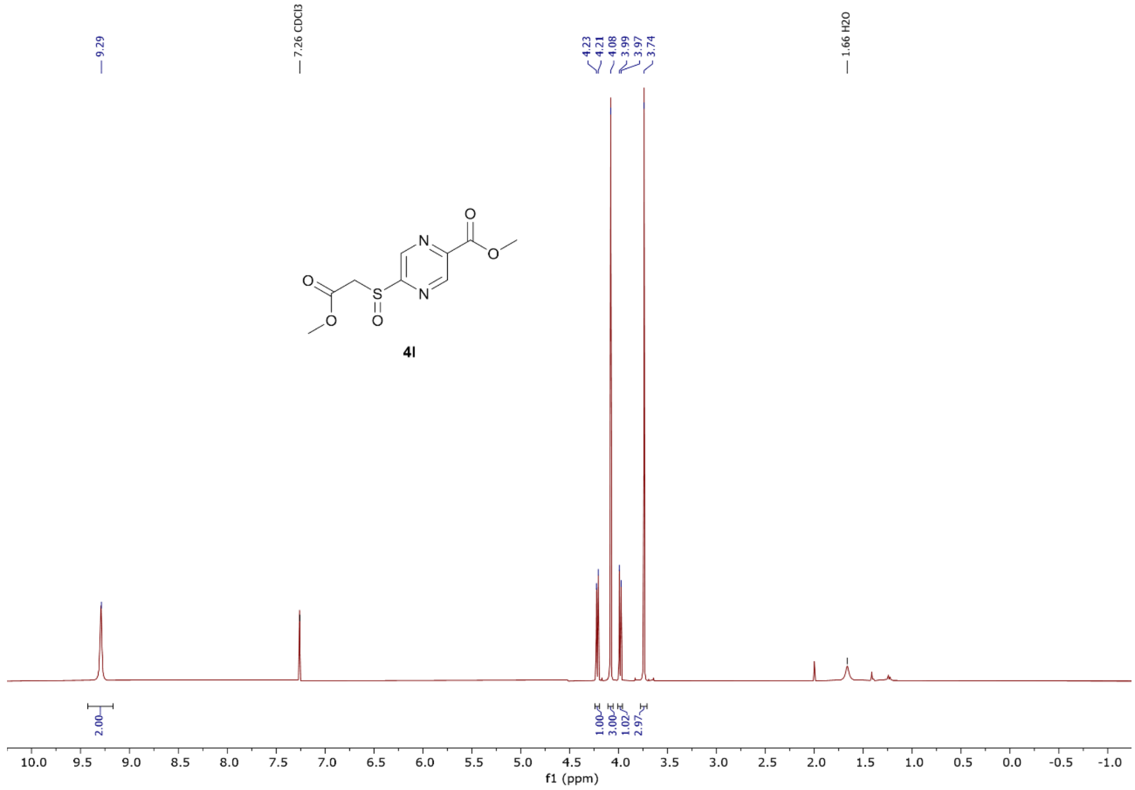

Figure S134. ^{1}H NMR spectrum of compound **4l** (800 MHz, $CDCl_3$).

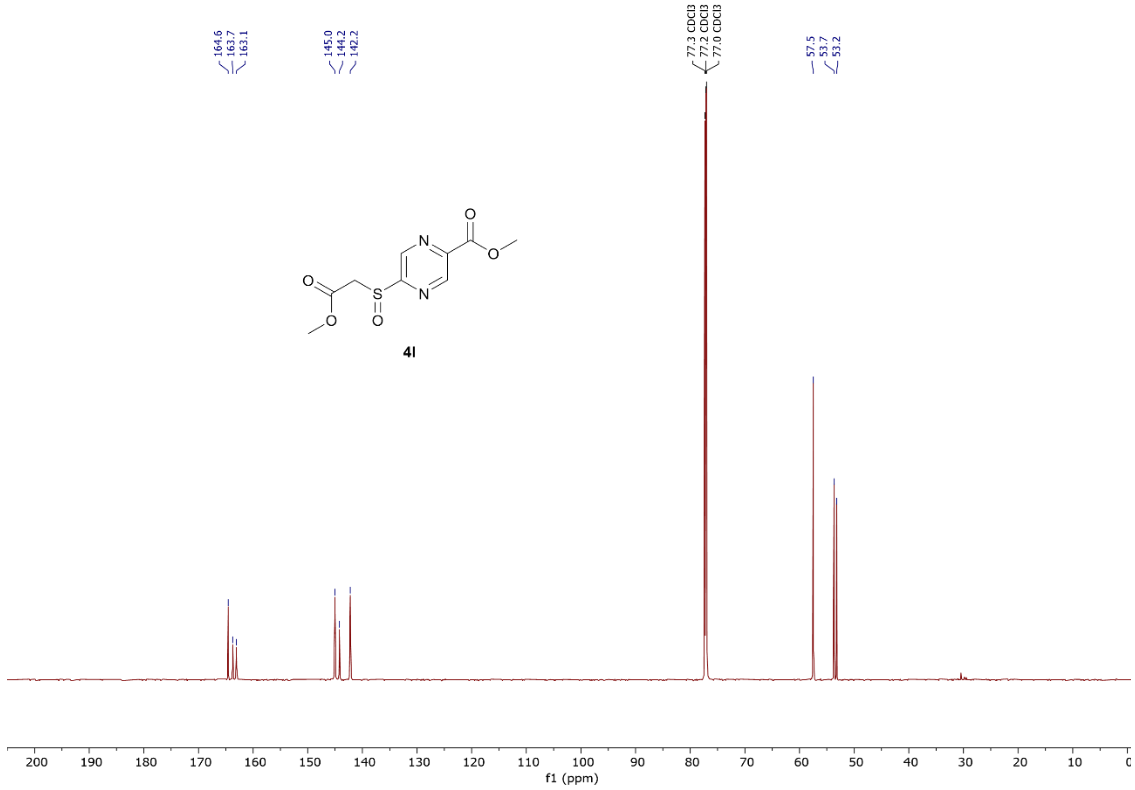

Figure S135. ^{13}C NMR spectrum of compound **4l** (201 MHz, $CDCl_3$).

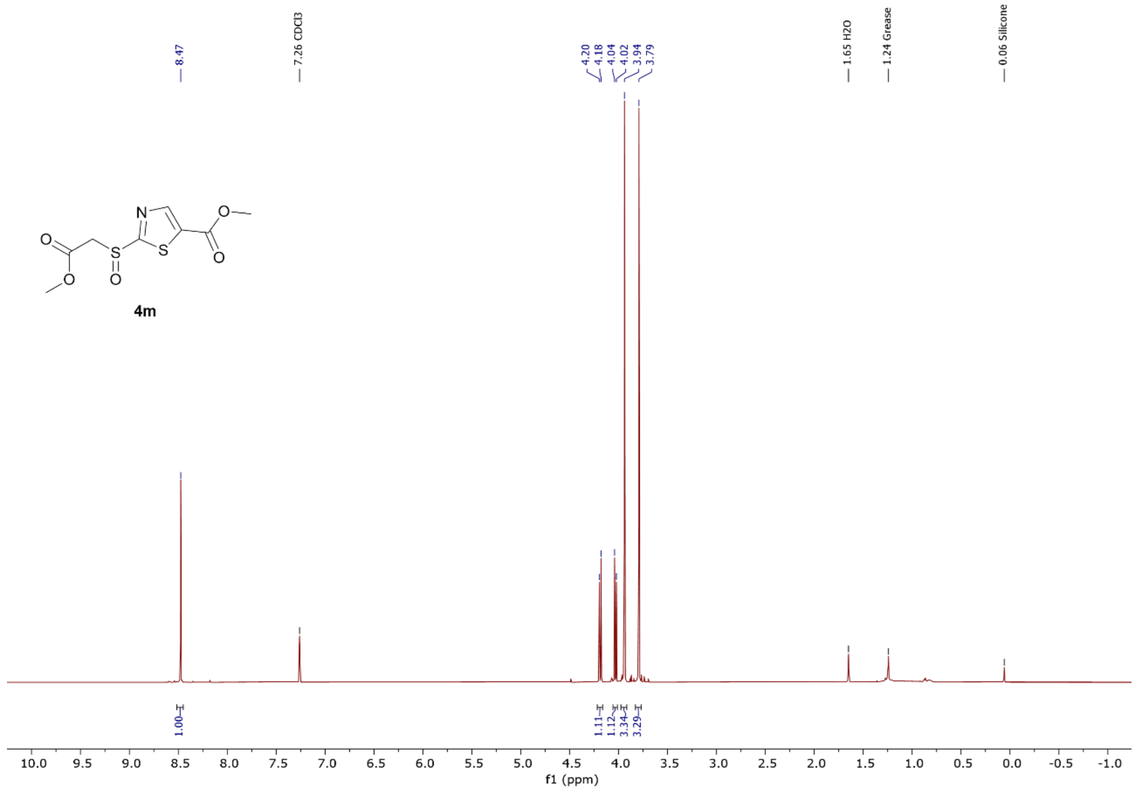

Figure S136. ^{1}H NMR spectrum of compound **4m** (800 MHz, $CDCl_3$).

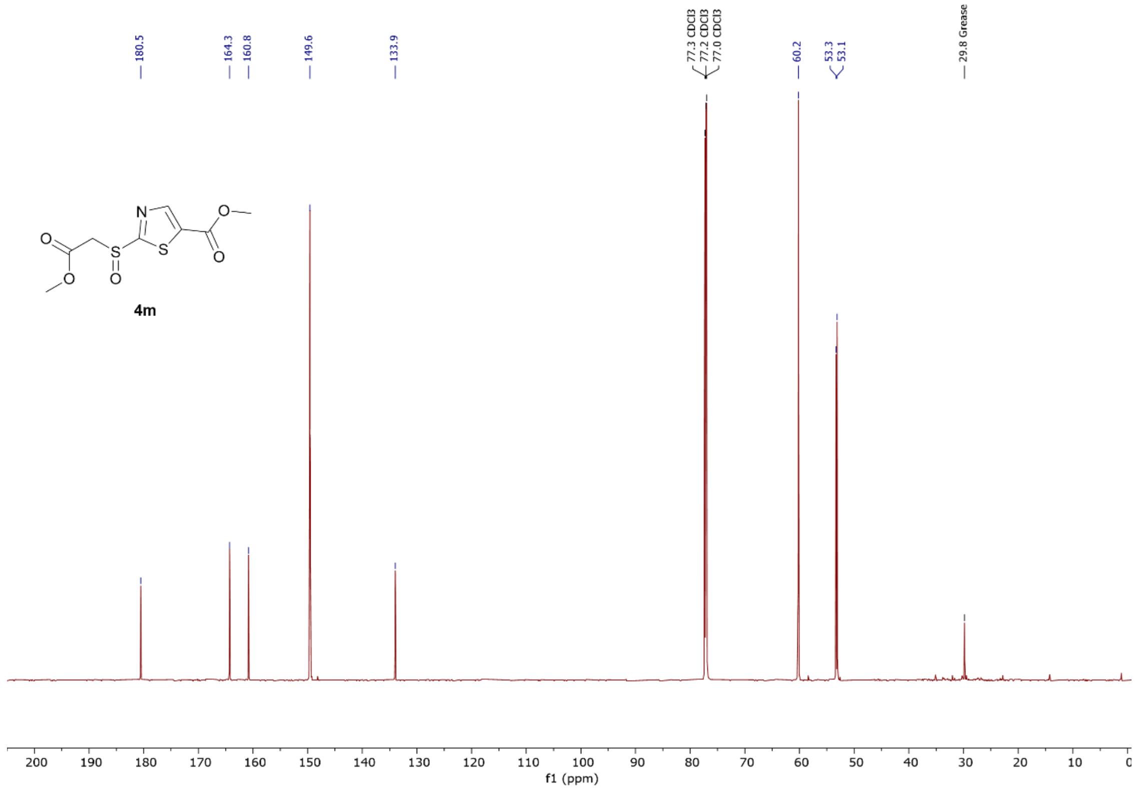

Figure S137. ^{13}C NMR spectrum of compound **4m** (201 MHz, $CDCl_3$).

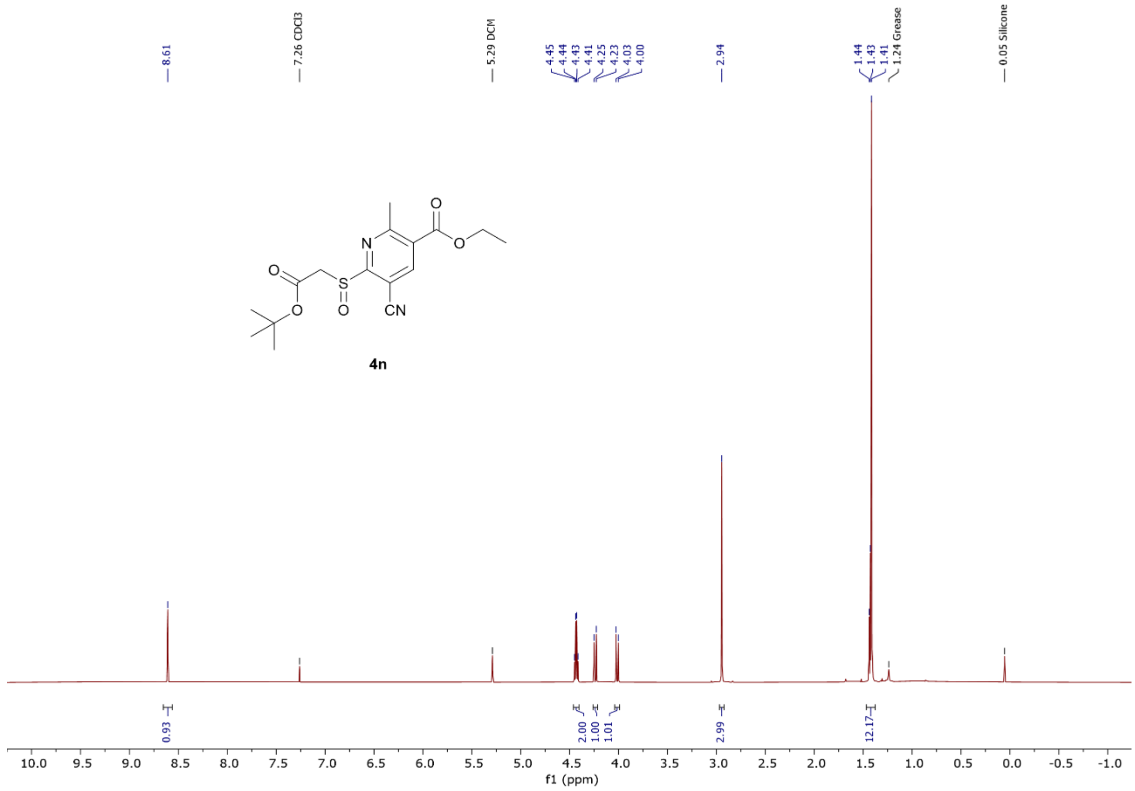

Figure S138. ^{1}H NMR spectrum of compound **4n** (600 MHz, $CDCl_3$).

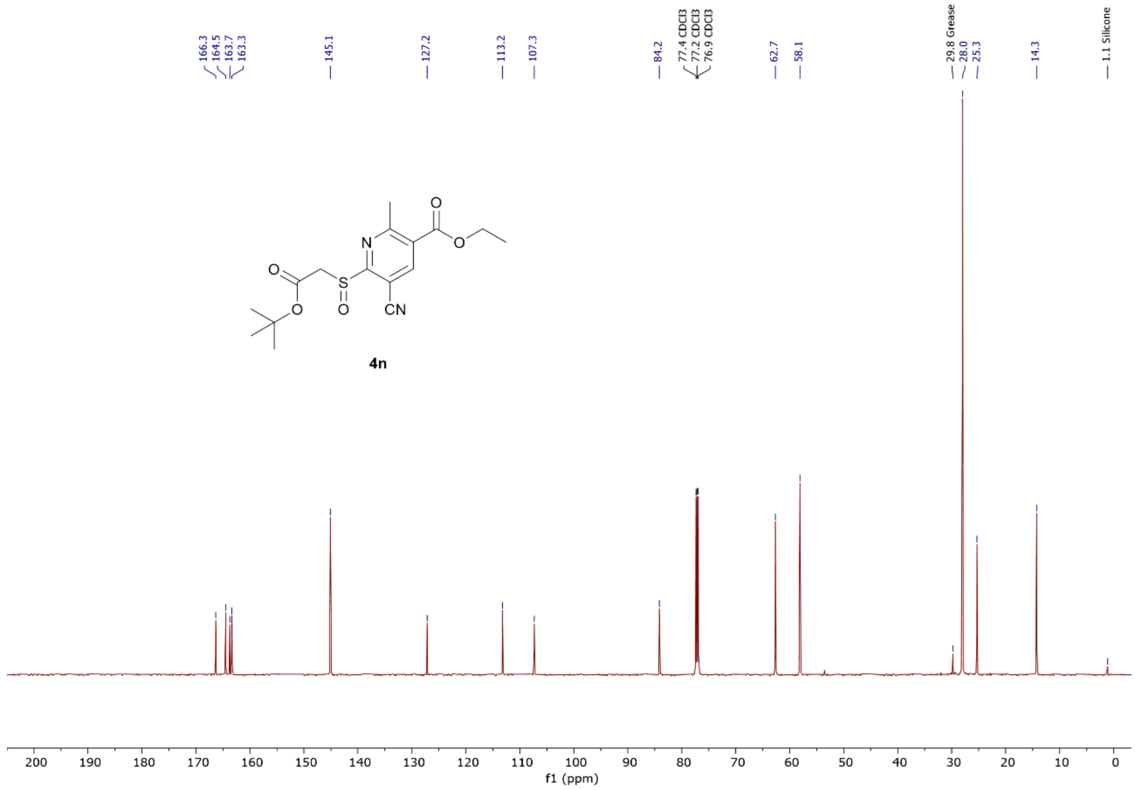

Figure S139. ^{13}C NMR spectrum of compound **4n** (151 MHz, $CDCl_3$).

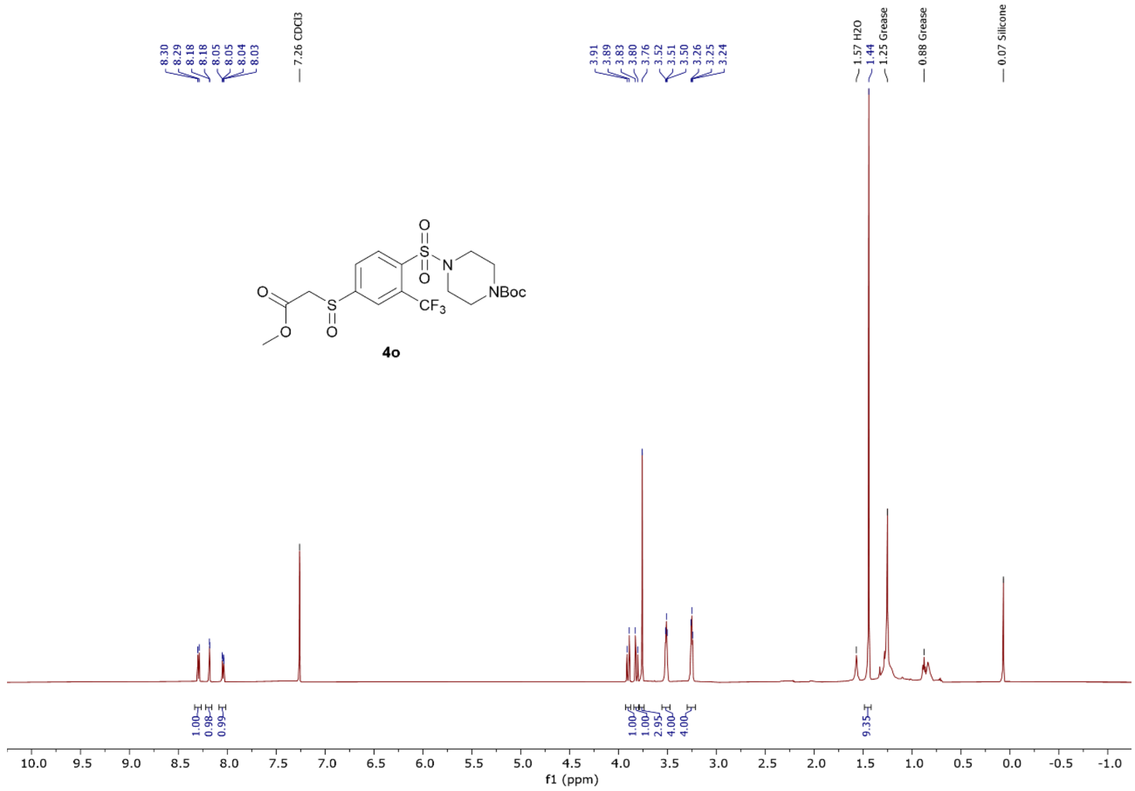

Figure S140. ^{1}H NMR spectrum of compound **4o** (600 MHz, $CDCl_3$).

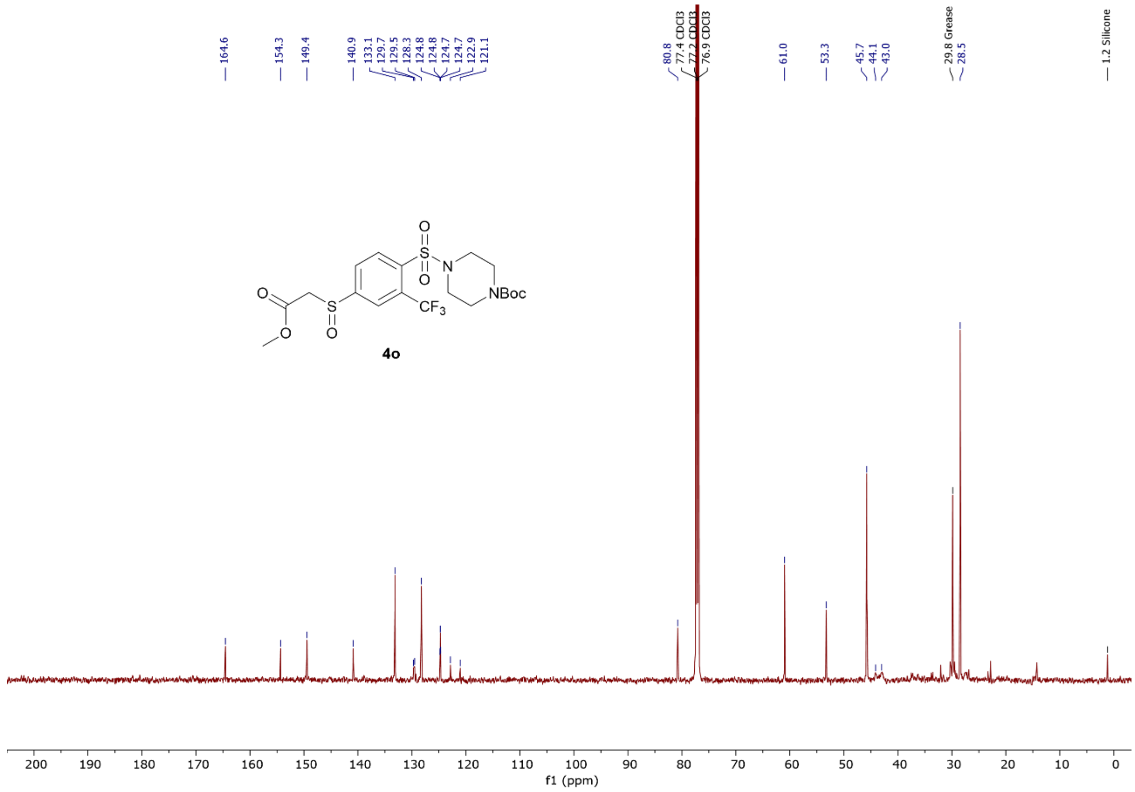

Figure S141. ^{13}C NMR spectrum of compound **4o** (151 MHz, $CDCl_3$).

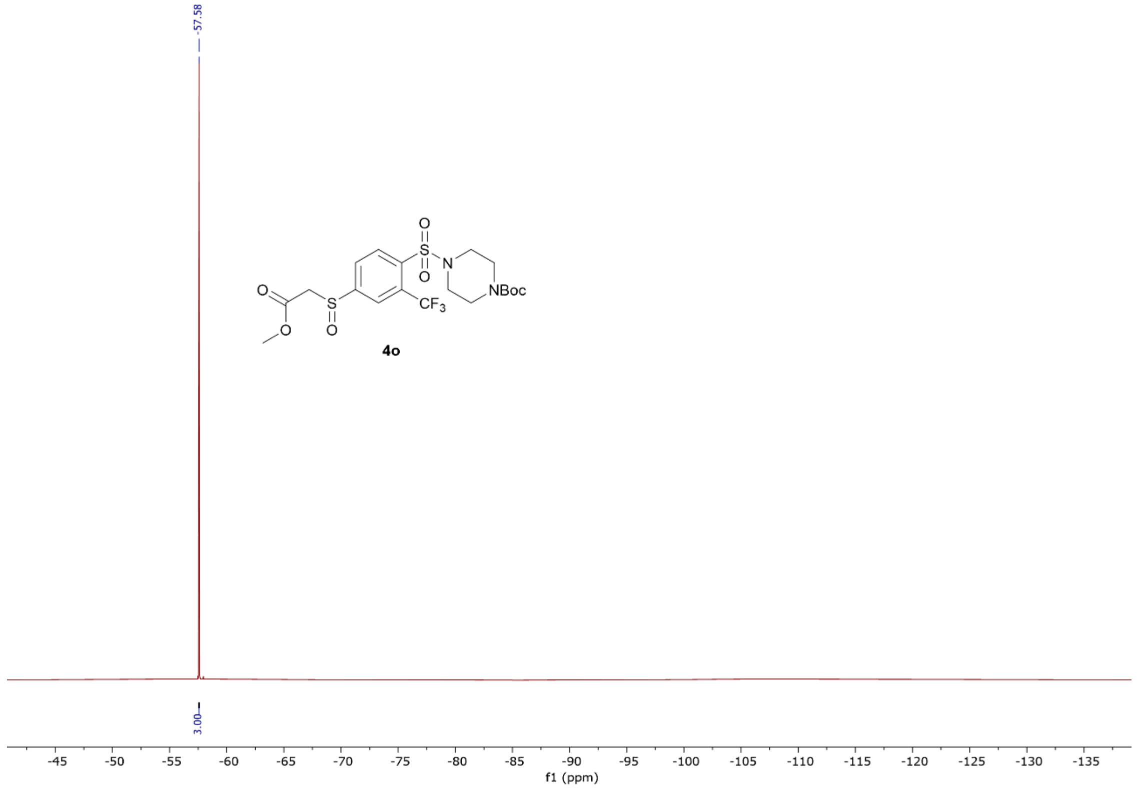

Figure S142. ^{19}F NMR spectrum of compound **4o** (564 MHz, $CDCl_3$).

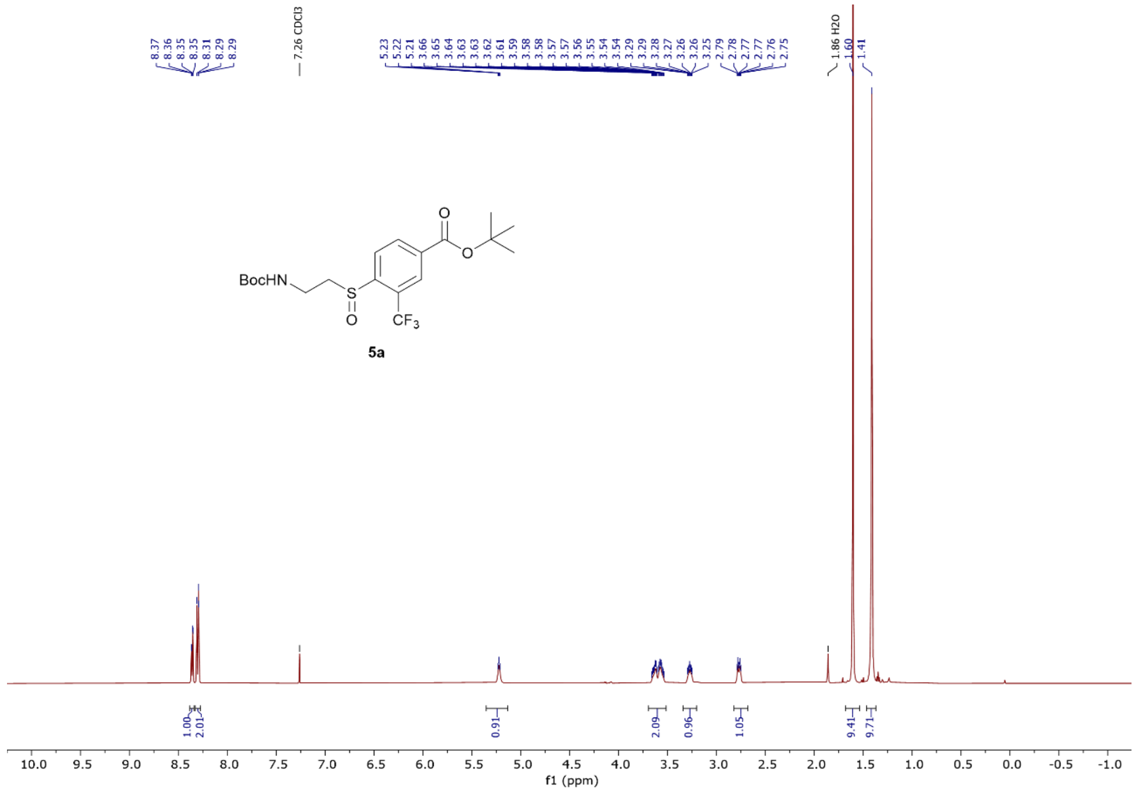

Figure S143. ^{1}H NMR spectrum of compound **5a** (600 MHz, $CDCl_3$).

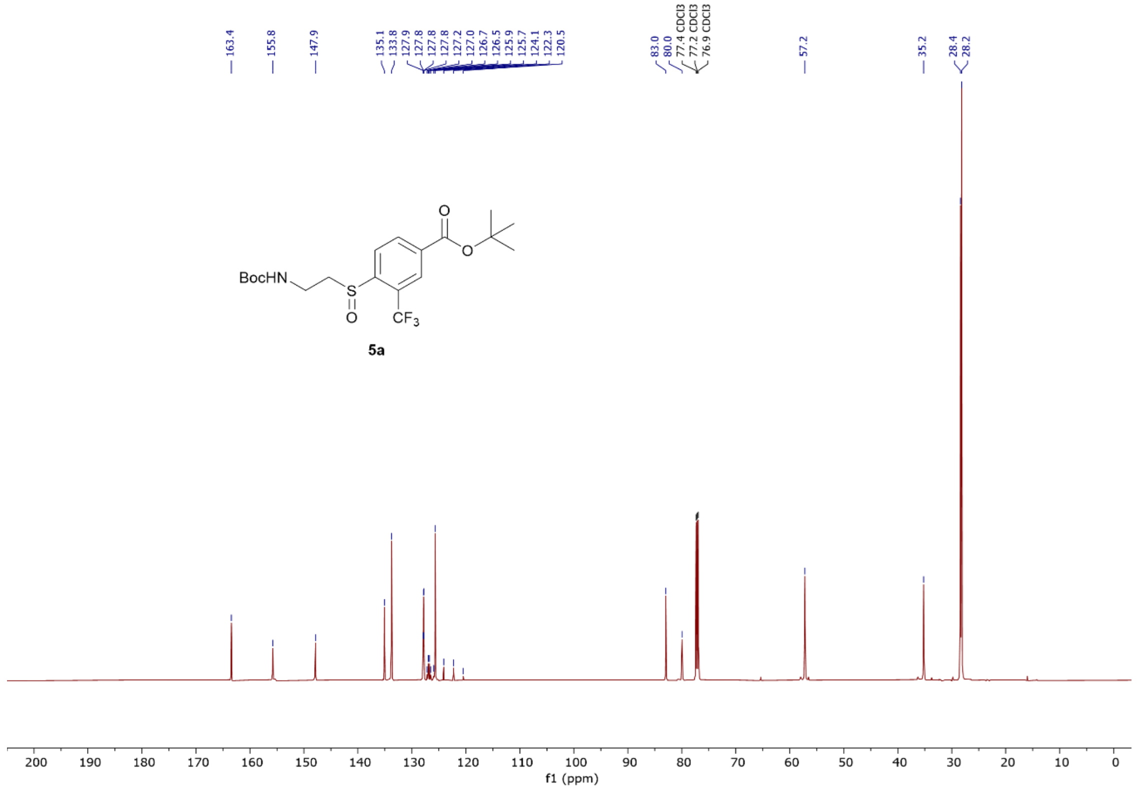

Figure S144. ^{13}C NMR spectrum of compound **5a** (151 MHz, $CDCl_3$).

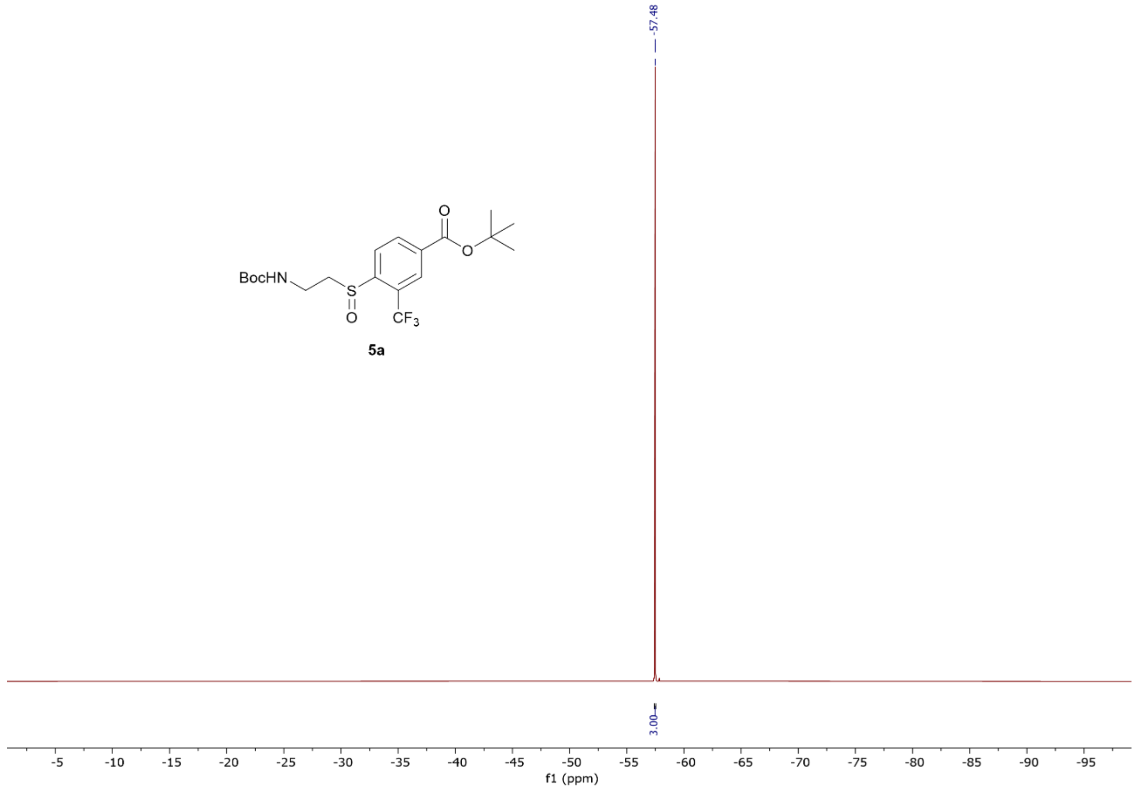

Figure S145. ^{19}F NMR spectrum of compound **5a** (564 MHz, $CDCl_3$).

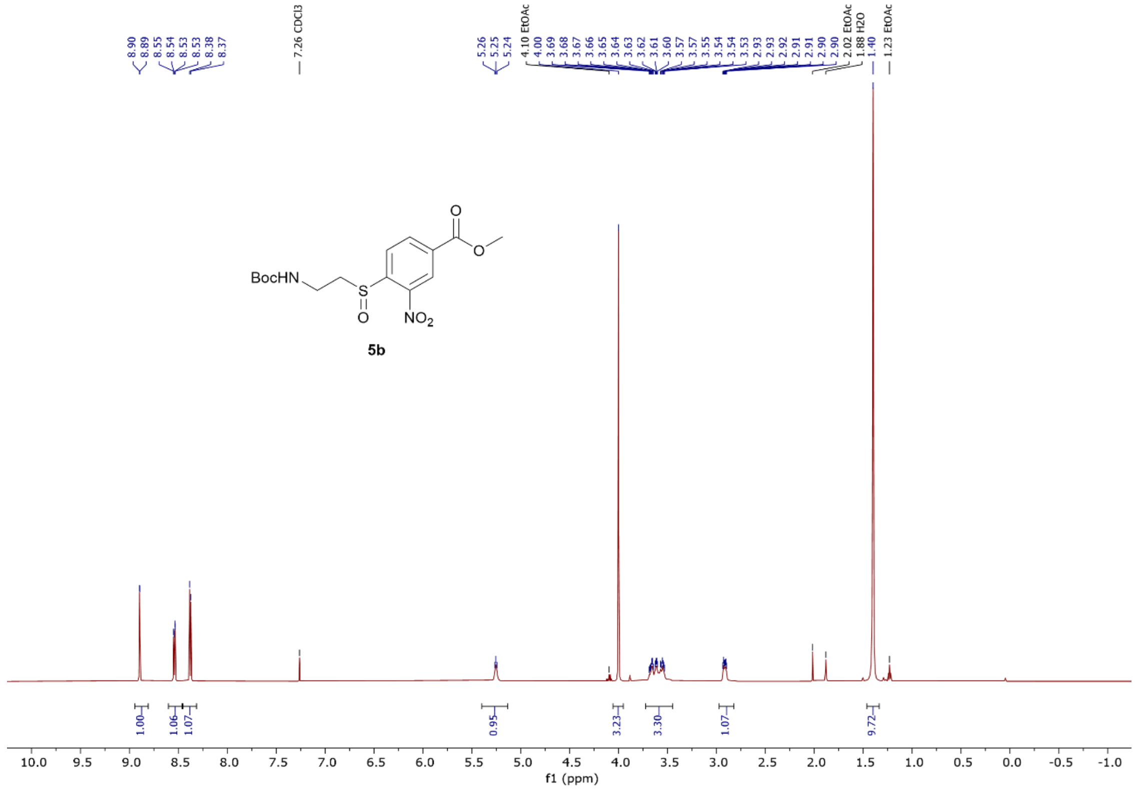

Figure S146. ^1H NMR spectrum of compound **5b** (600 MHz, $CDCl_3$).

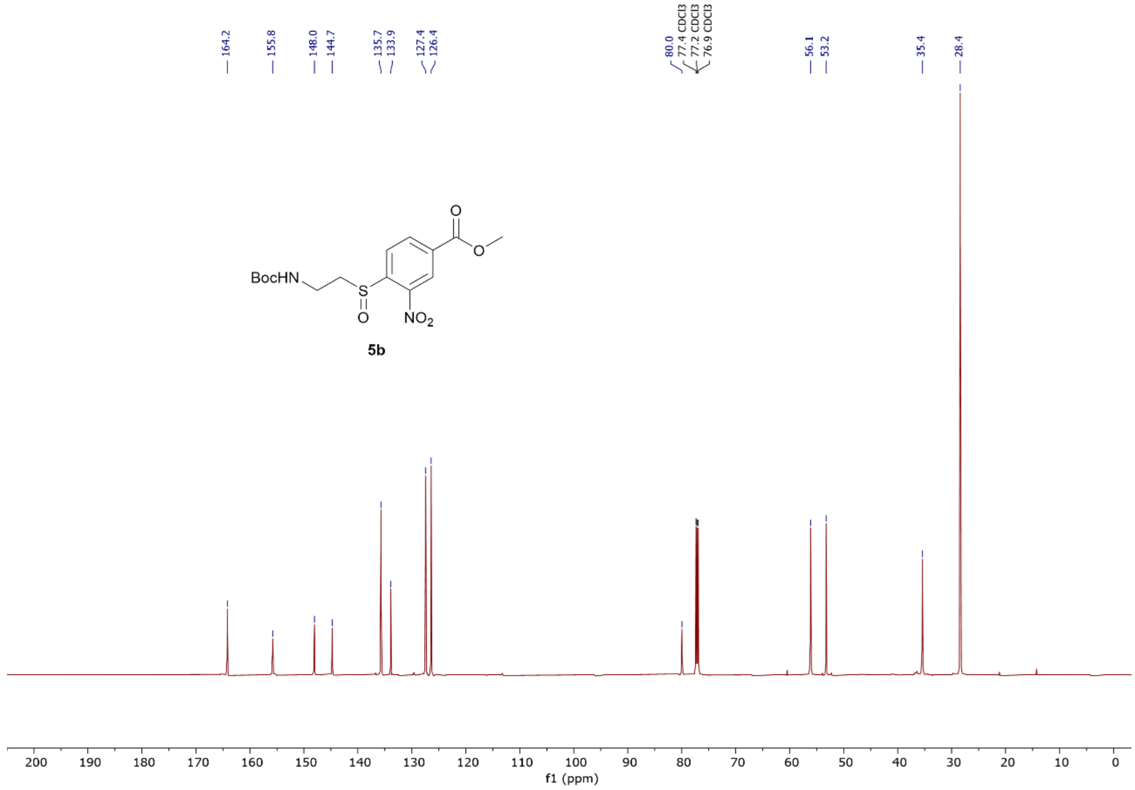

Figure S147. ^{13}C NMR spectrum of compound **5b** (151 MHz, $CDCl_3$).

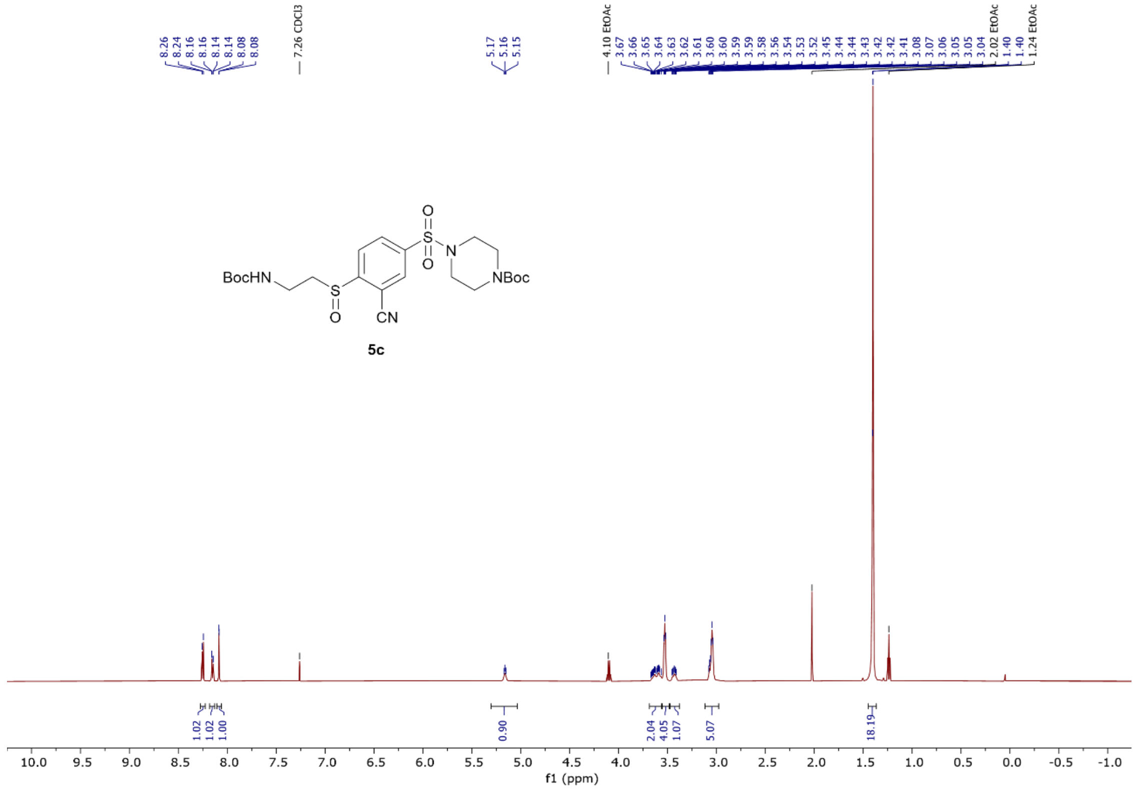

Figure S148. ^{1}H NMR spectrum of compound **5c** (600 MHz, $CDCl_3$).

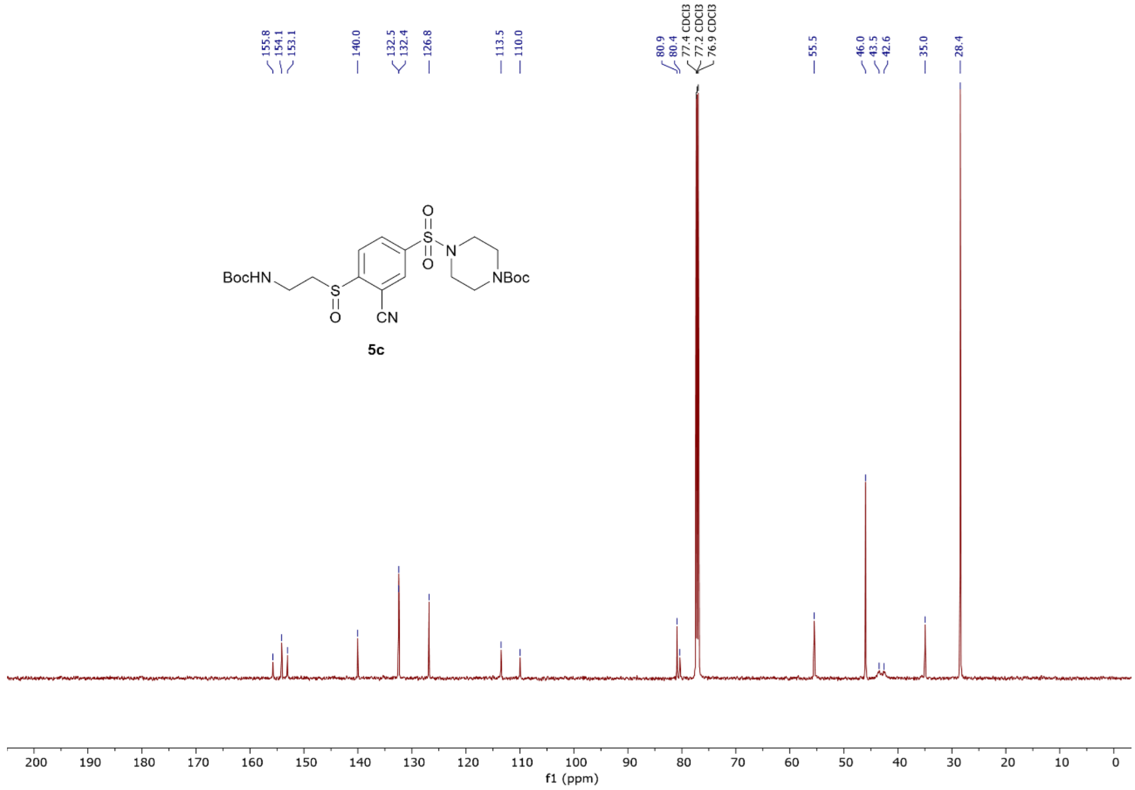

Figure S149. ^{13}C NMR spectrum of compound **5c** (151 MHz, $CDCl_3$).

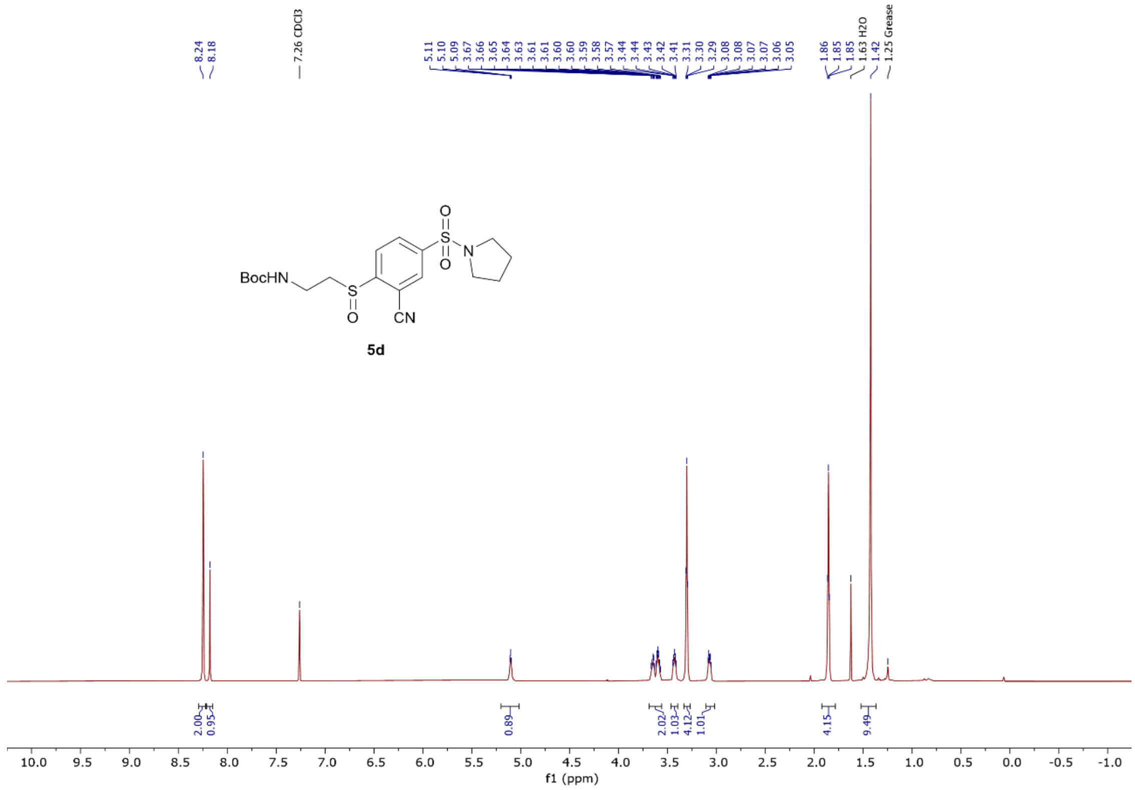

Figure S150. ^{1}H NMR spectrum of compound **5d** (800 MHz, $CDCl_3$).

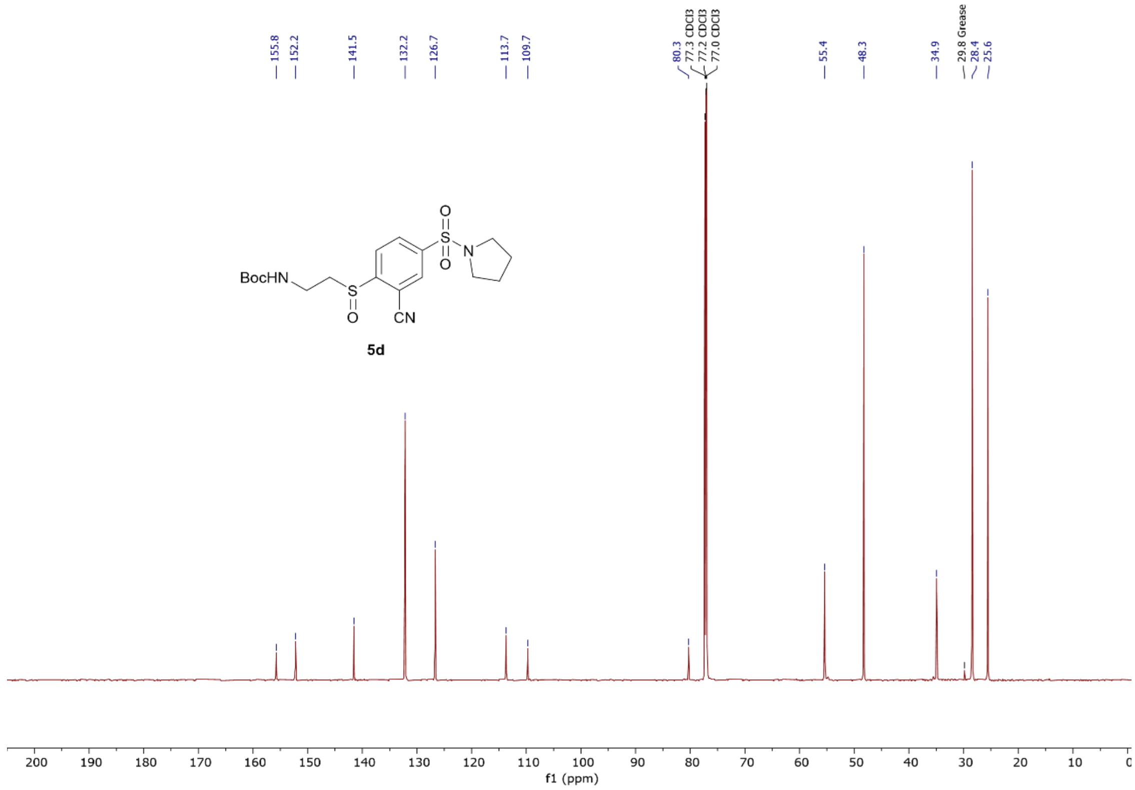

Figure S151. ^{13}C NMR spectrum of compound **5d** (201 MHz, $CDCl_3$).

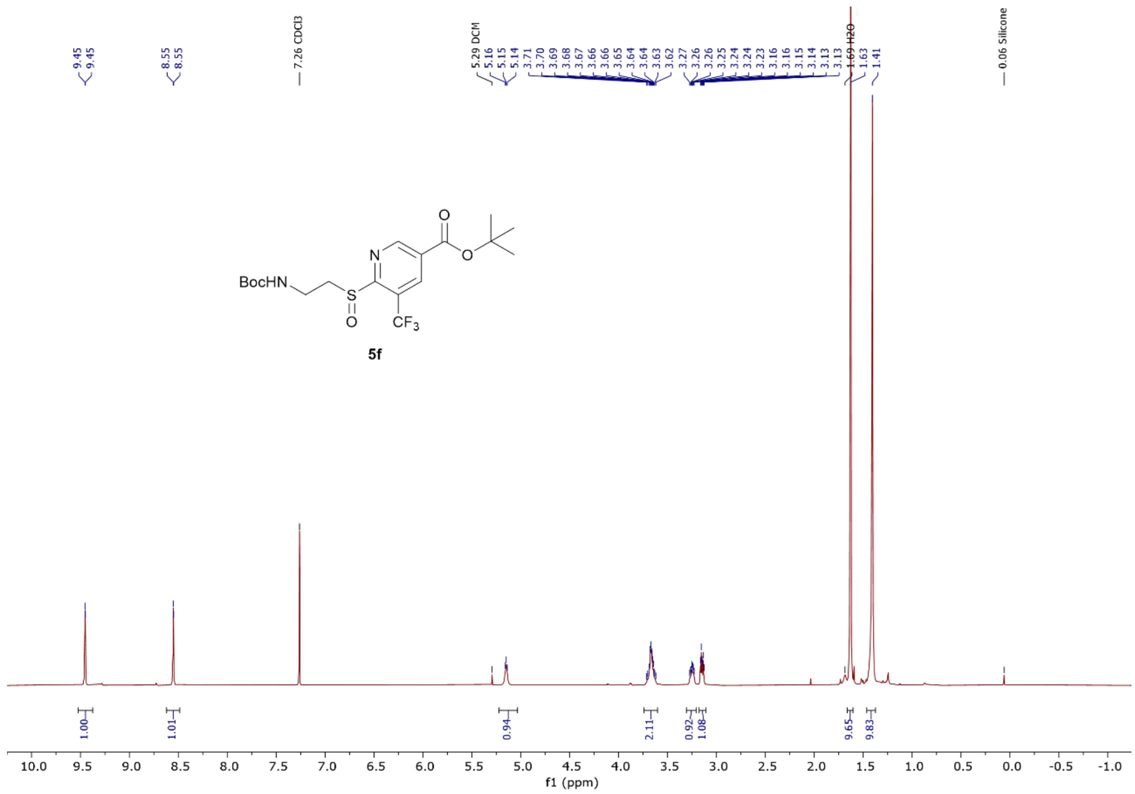

Figure S152. ^{1}H NMR spectrum of compound **5f** (600 MHz, $CDCl_3$).

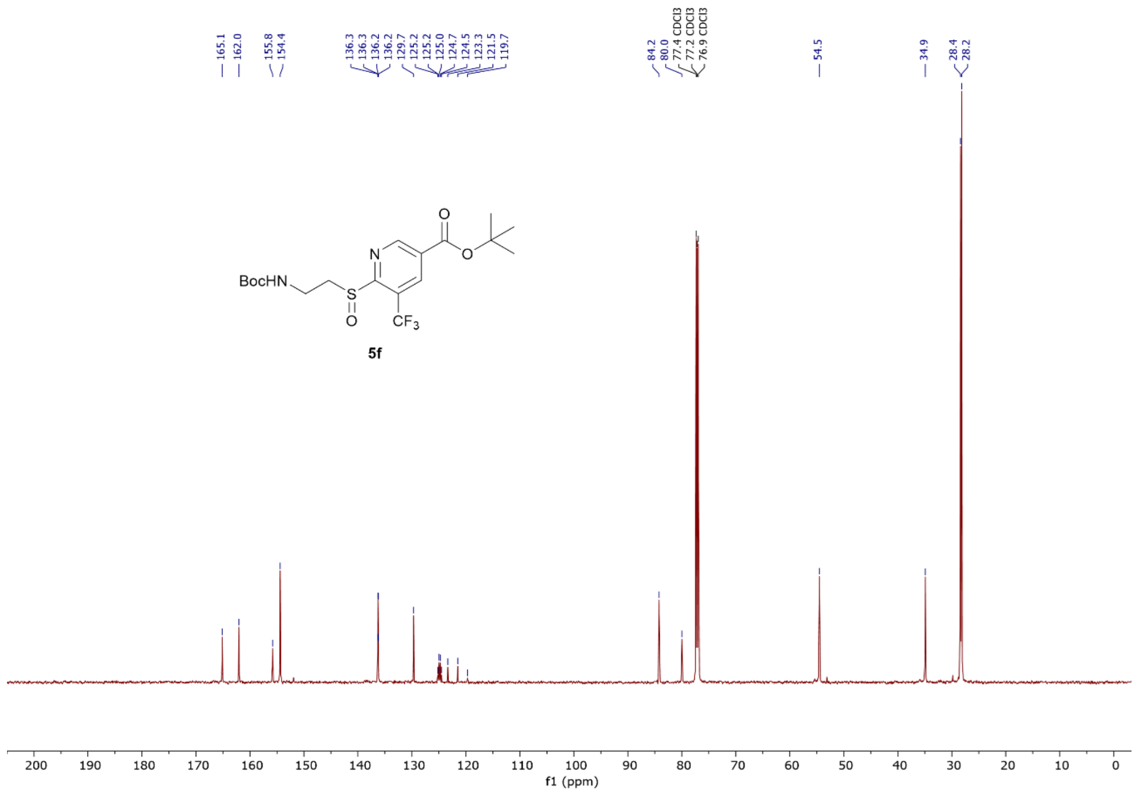

Figure S153. ^{13}C NMR spectrum of compound **5f** (151 MHz, $CDCl_3$).

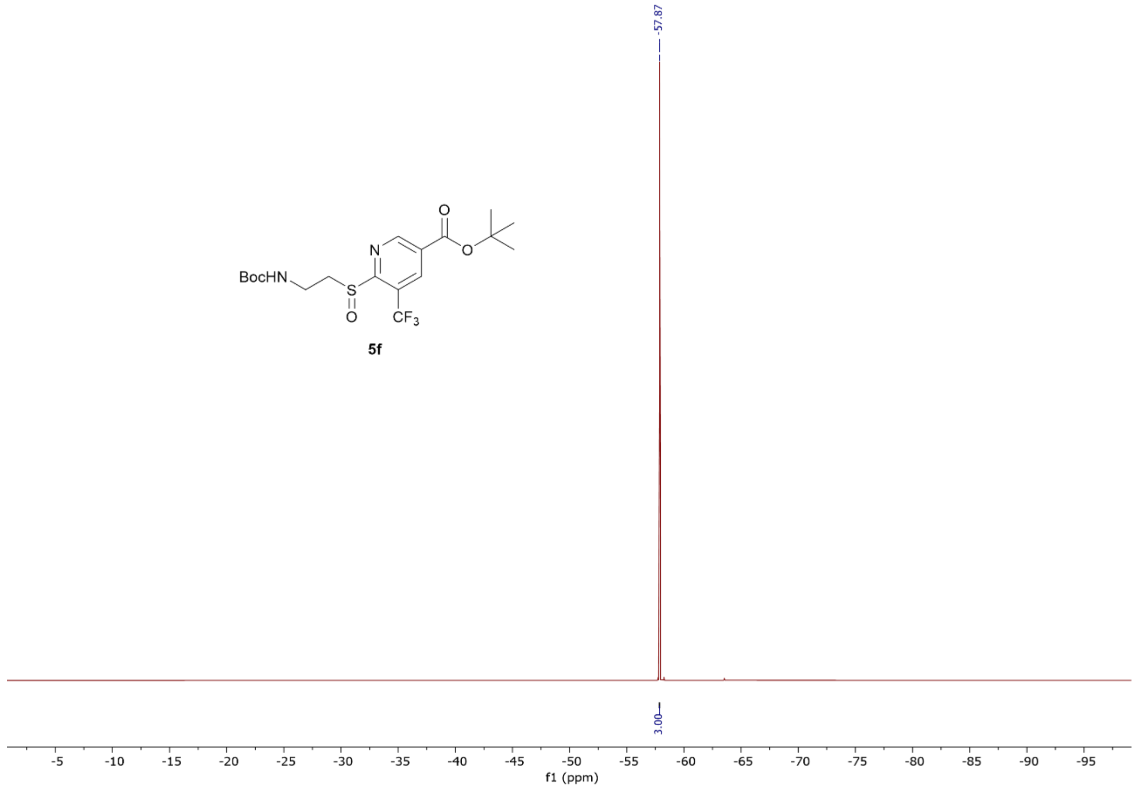

Figure S154. ^{19}F NMR spectrum of compound **5f** (564 MHz, $CDCl_3$).

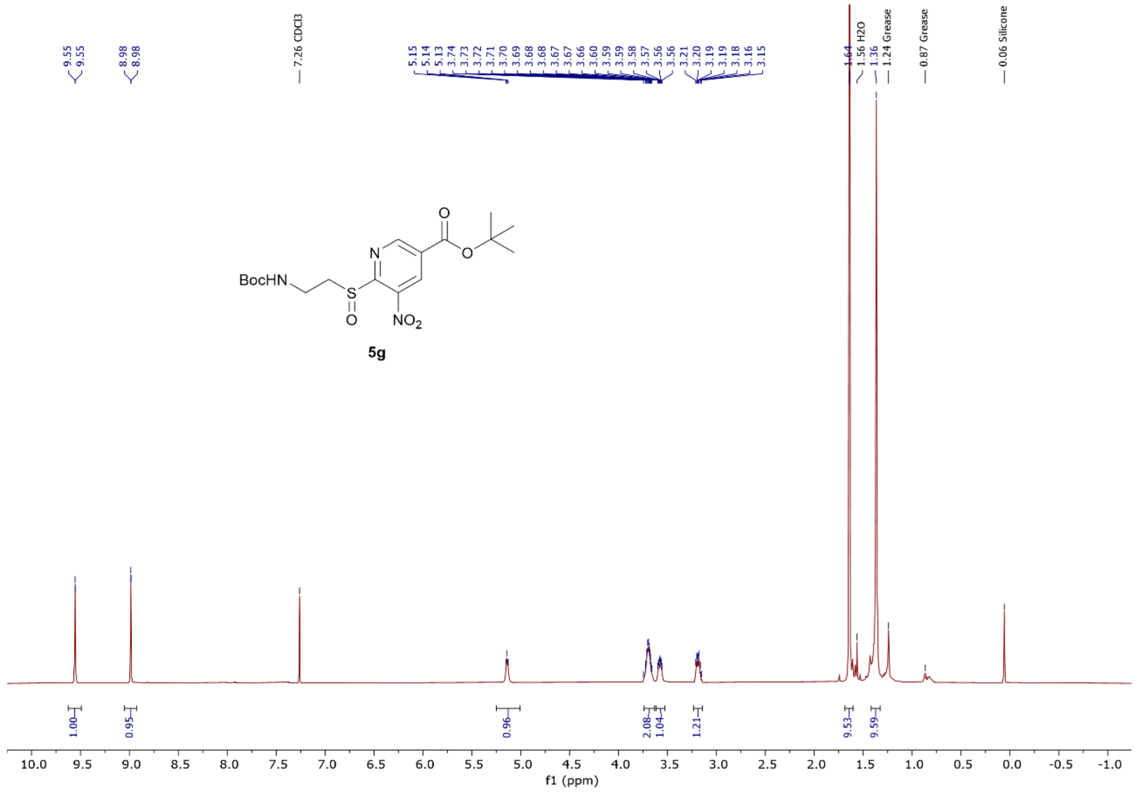

Figure S155. ^{1}H NMR spectrum of compound **5g** (600 MHz, $CDCl_3$).

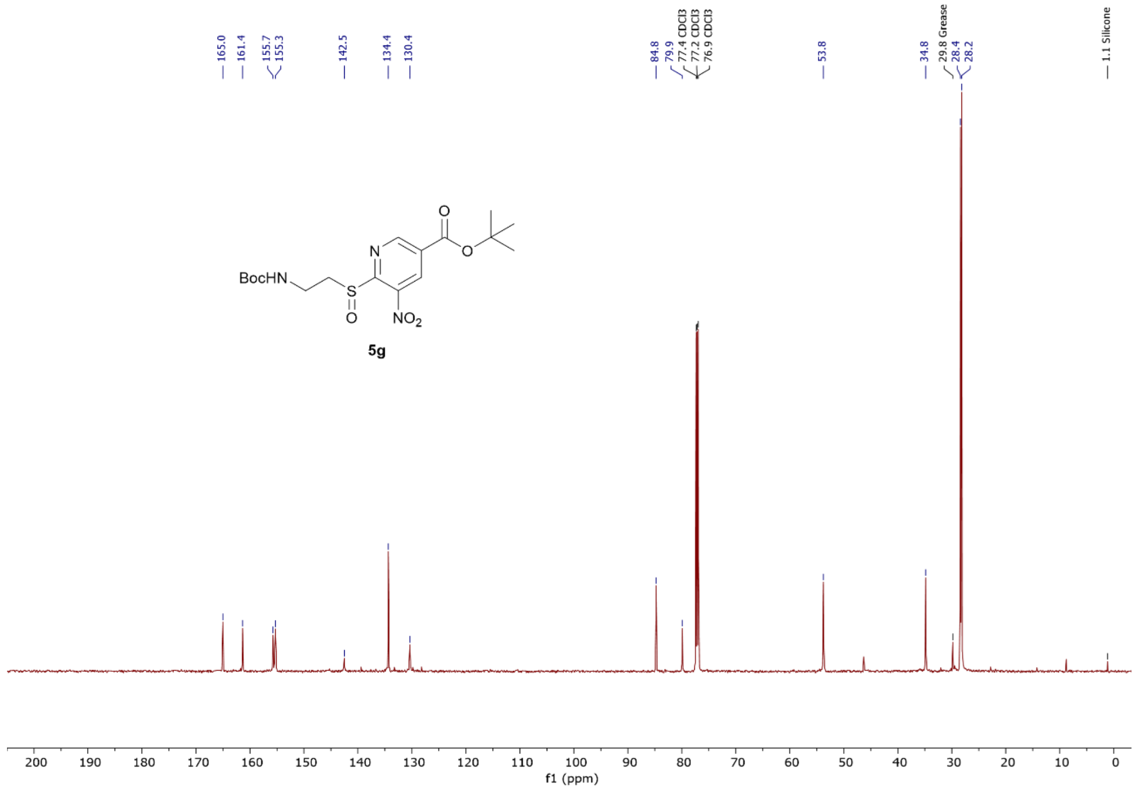

Figure S156. ^{13}C NMR spectrum of compound **5g** (151 MHz, $CDCl_3$).

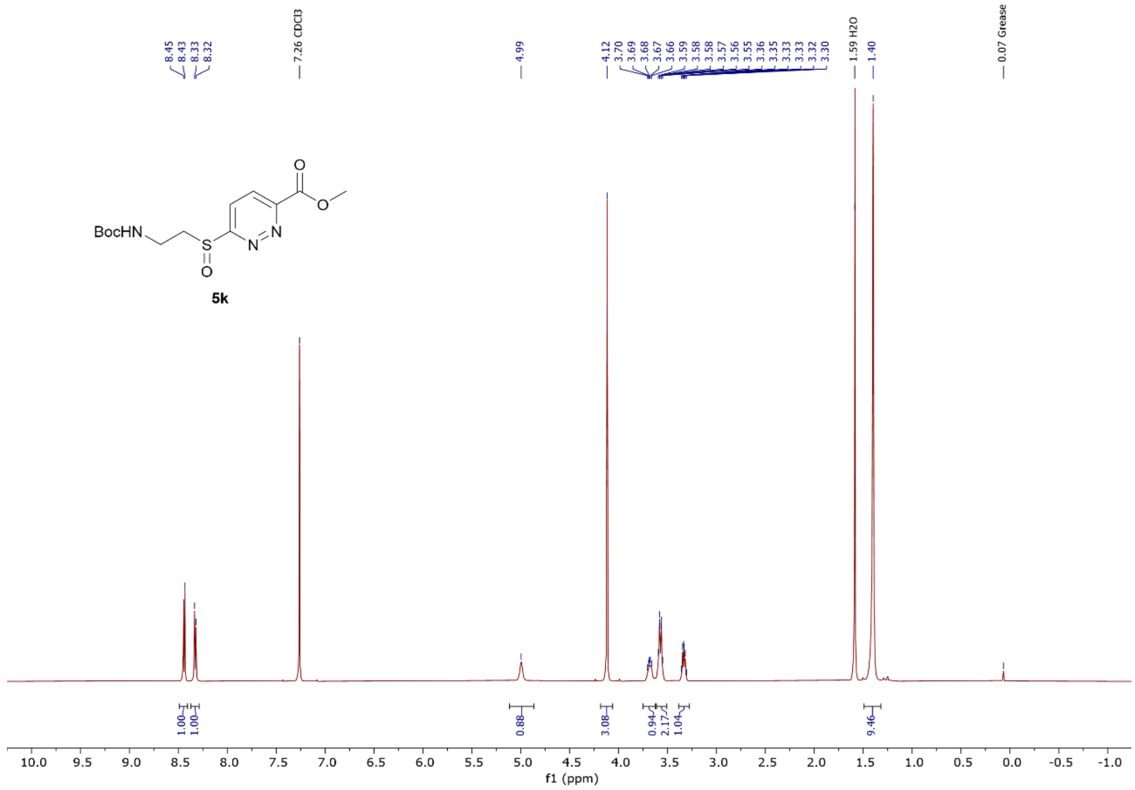

Figure S157. ^{1}H NMR spectrum of compound **5k** (600 MHz, $CDCl_3$).

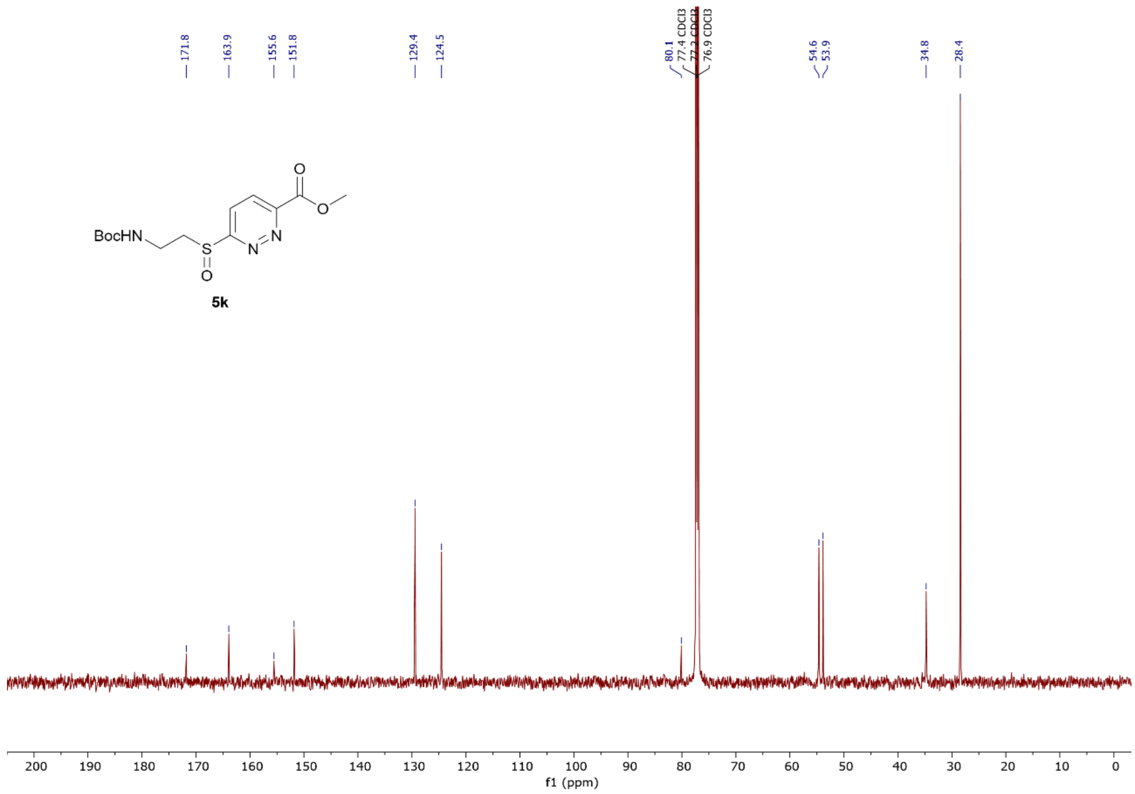

Figure S158. ^{13}C NMR spectrum of compound **5k** (151 MHz, $CDCl_3$).

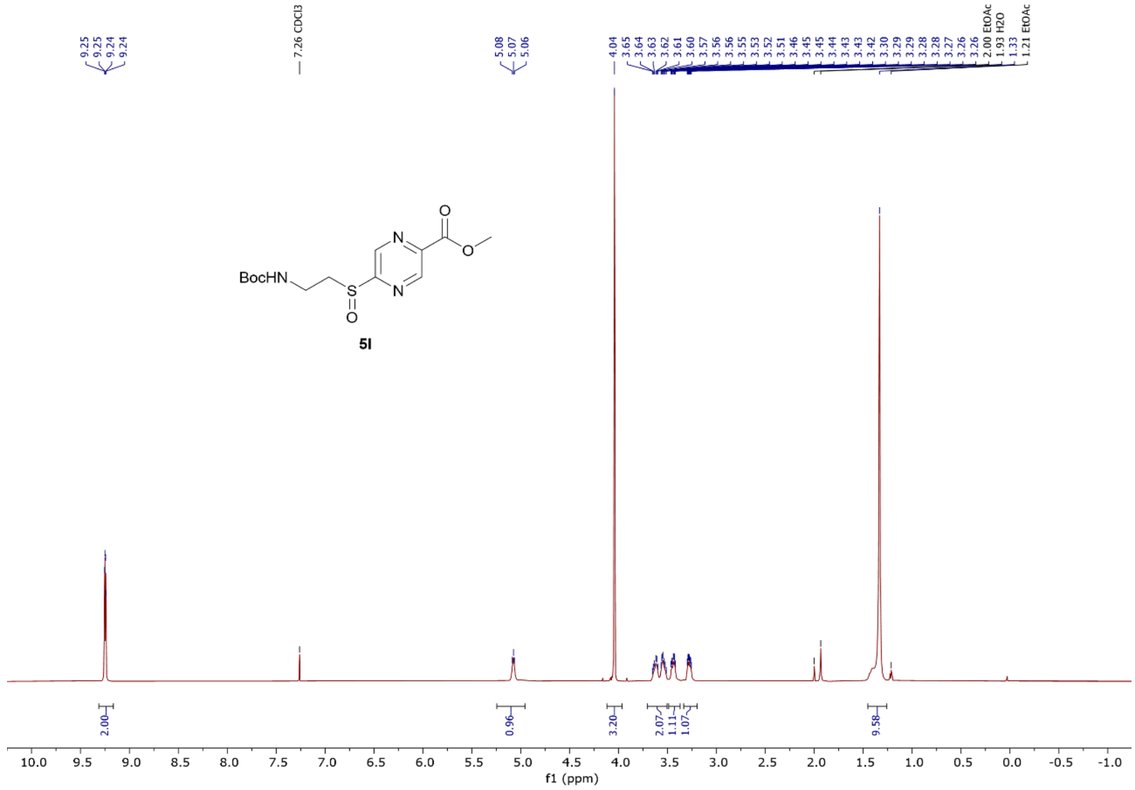

Figure S159. ^{1}H NMR spectrum of compound **5l** (600 MHz, $CDCl_3$).

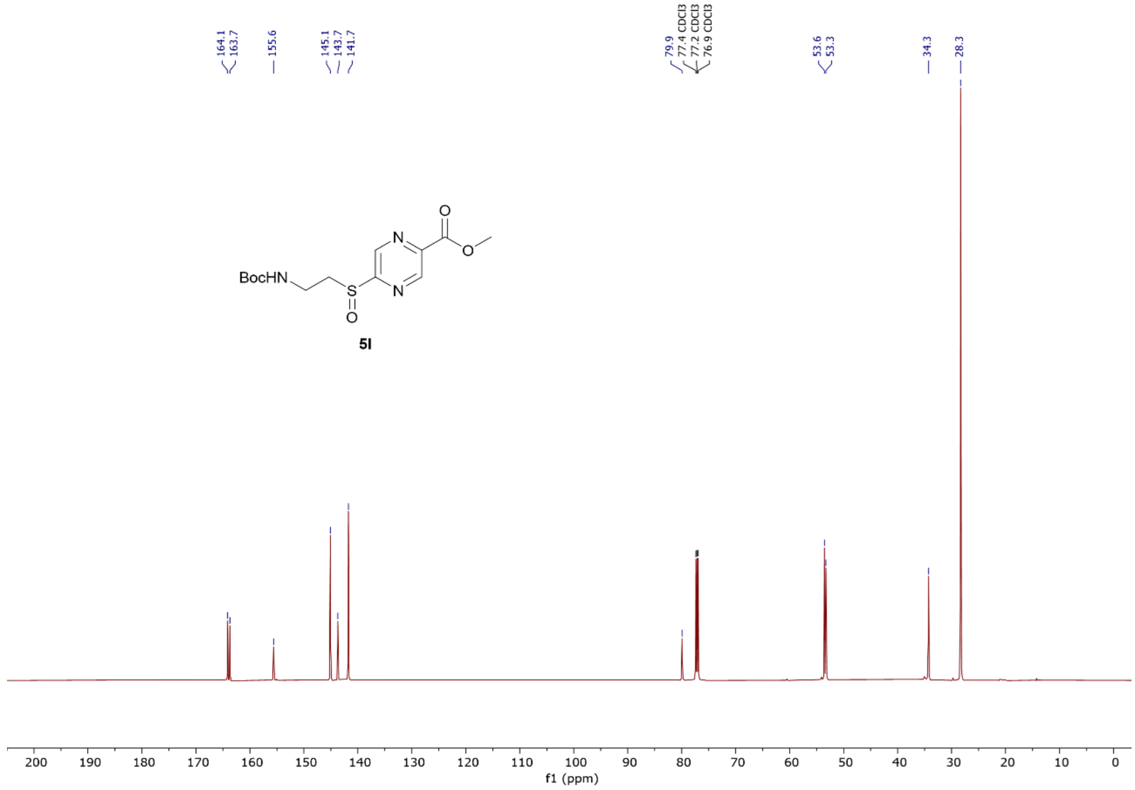

Figure S160. ^{13}C NMR spectrum of compound **5l** (151 MHz, $CDCl_3$).

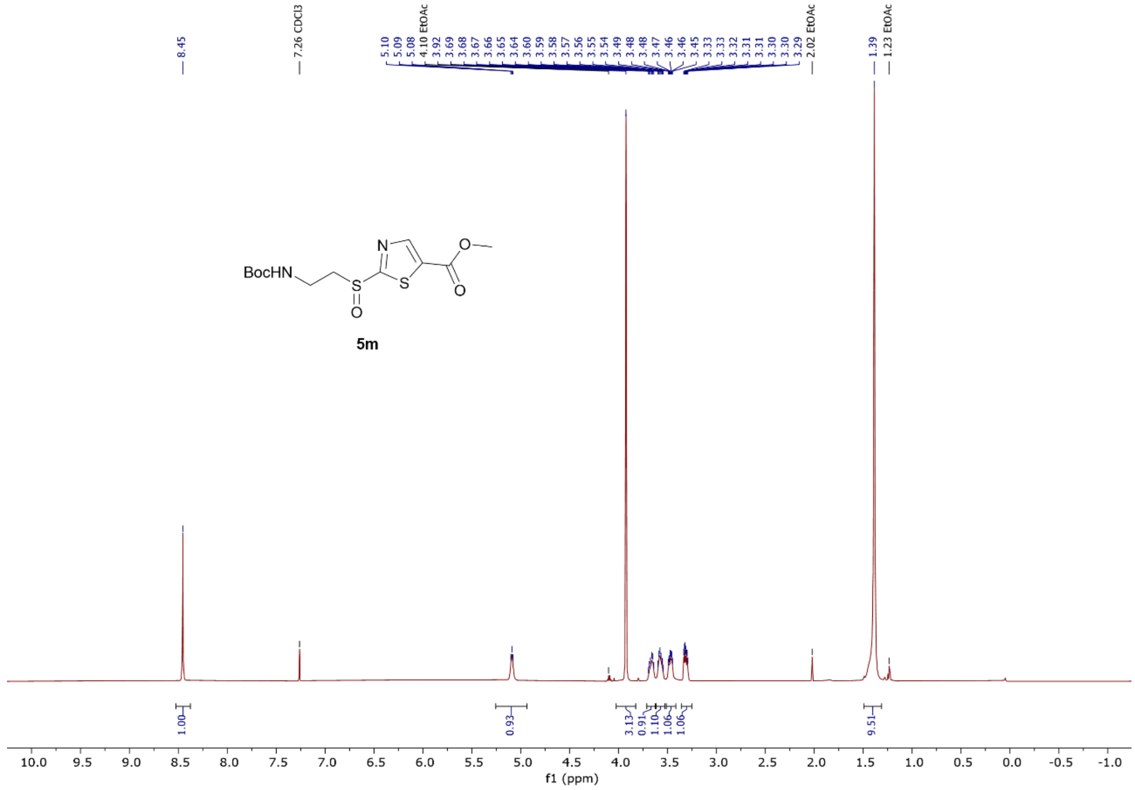

Figure S161. ^{1}H NMR spectrum of compound **5m** (600 MHz, $CDCl_3$).

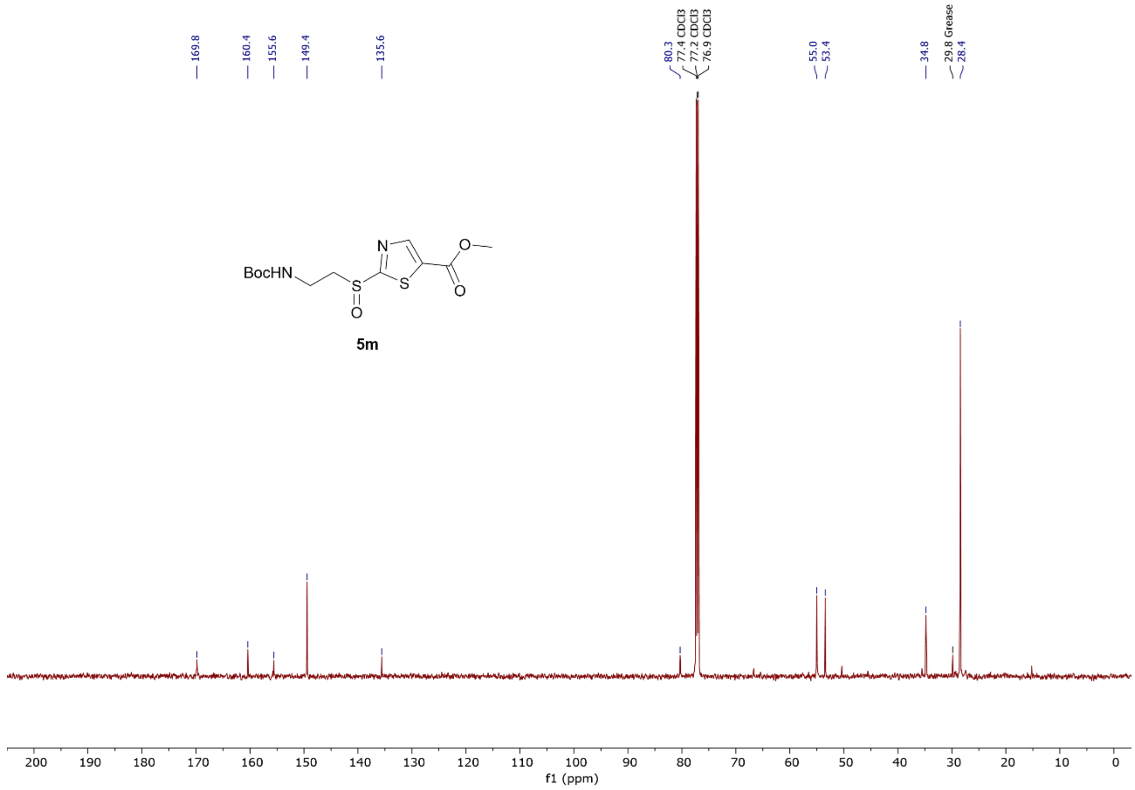

Figure S162. ^{13}C NMR spectrum of compound **5m** (151 MHz, $CDCl_3$).

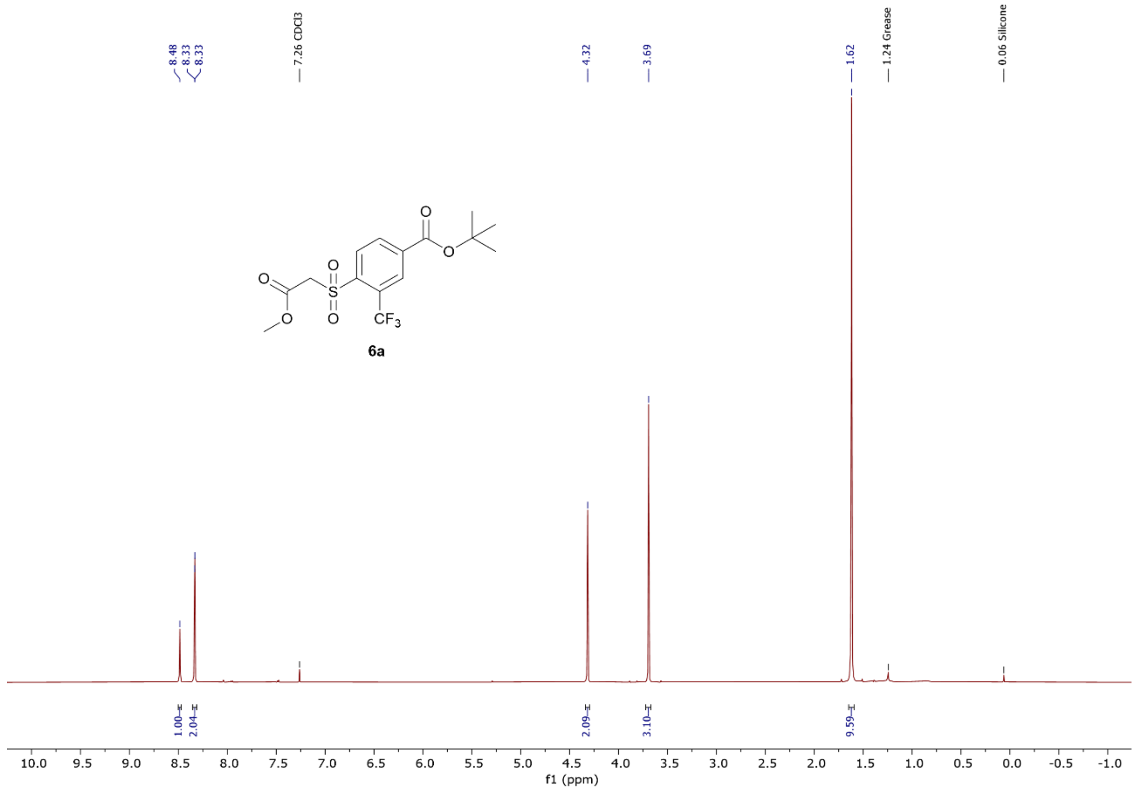

Figure S163. ^{1}H NMR spectrum of compound **6a** (600 MHz, $CDCl_3$).

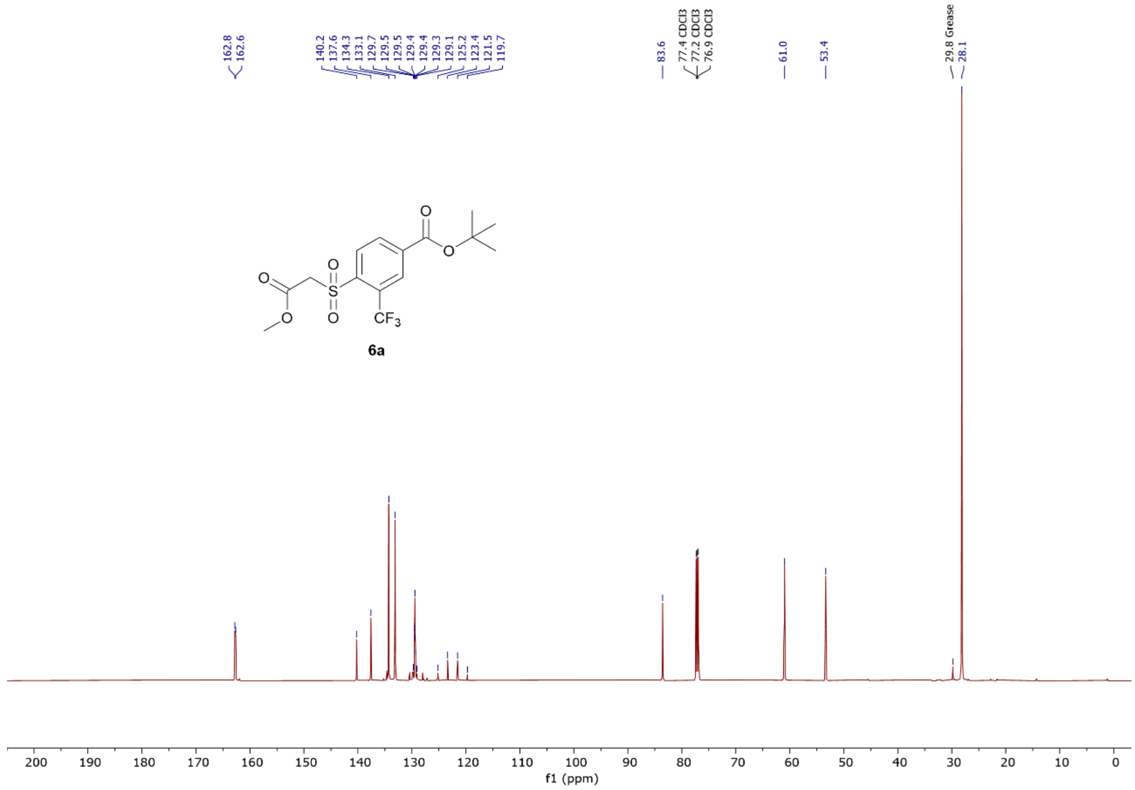

Figure S164. ^{13}C NMR spectrum of compound **6a** (151 MHz, $CDCl_3$).

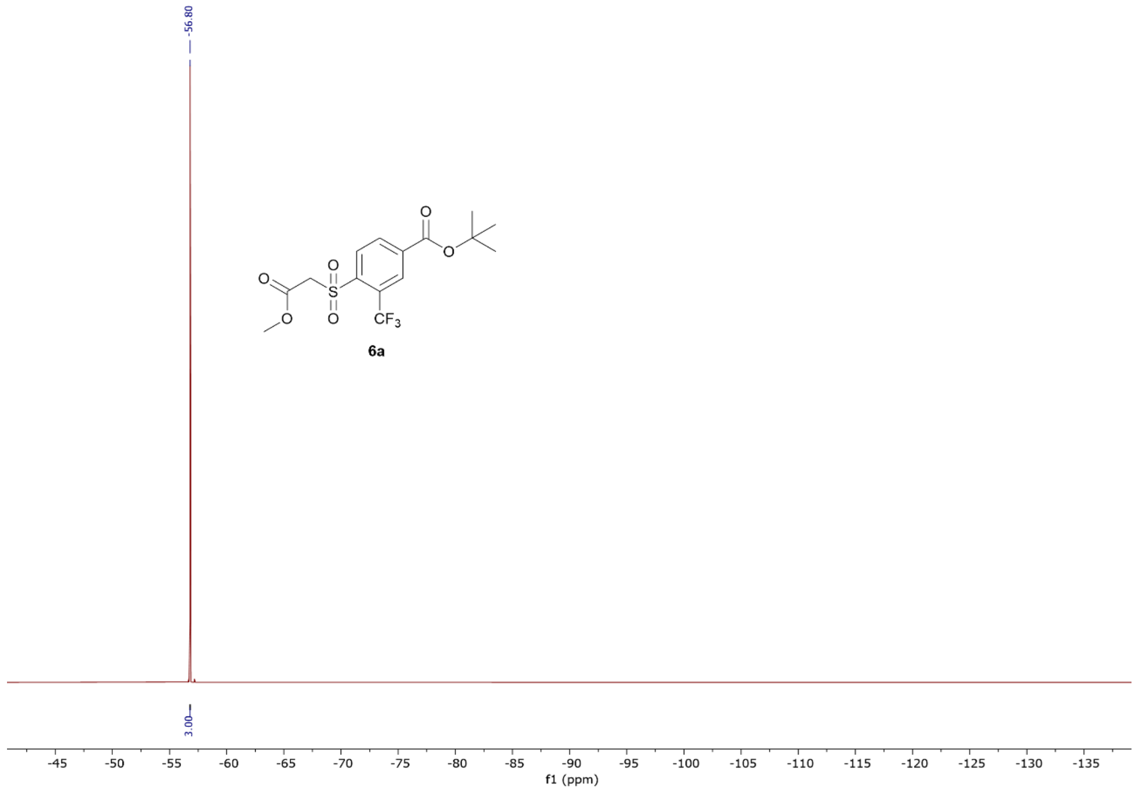

Figure S165. ^{19}F NMR spectrum of compound **6a** (564 MHz, $CDCl_3$).

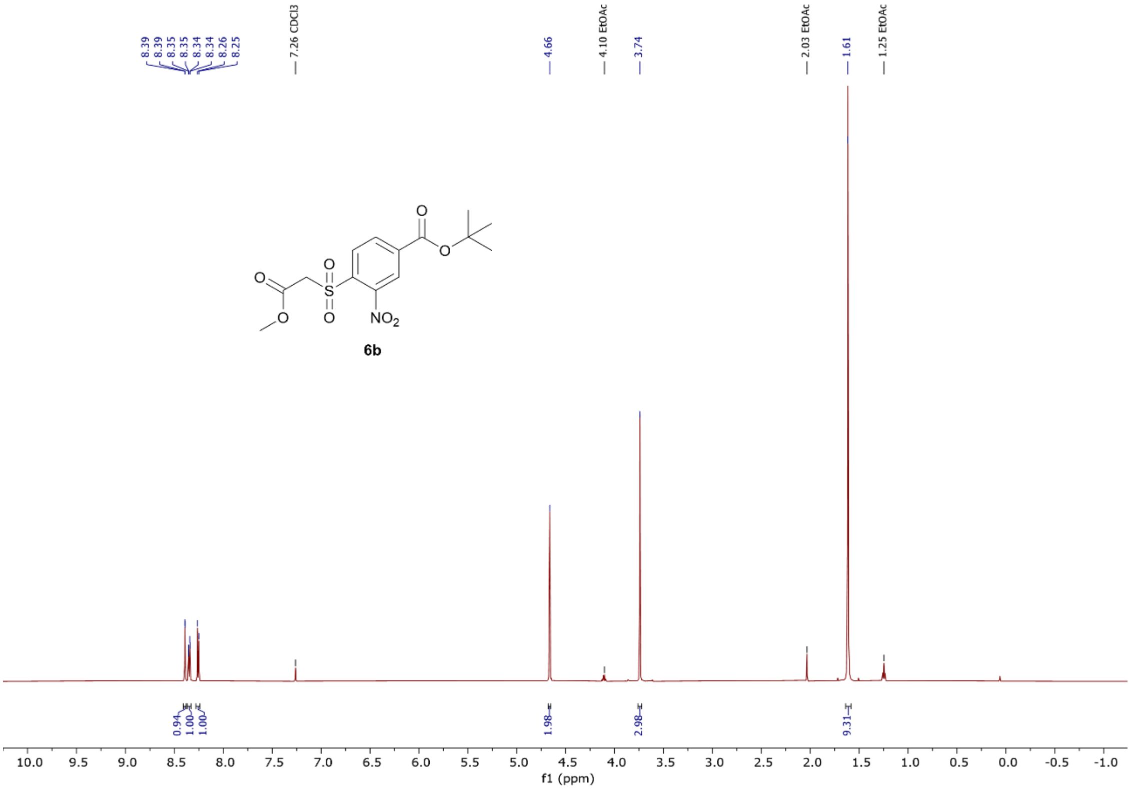

Figure S166. ^{1}H NMR spectrum of compound **6b** (600 MHz, $CDCl_3$).

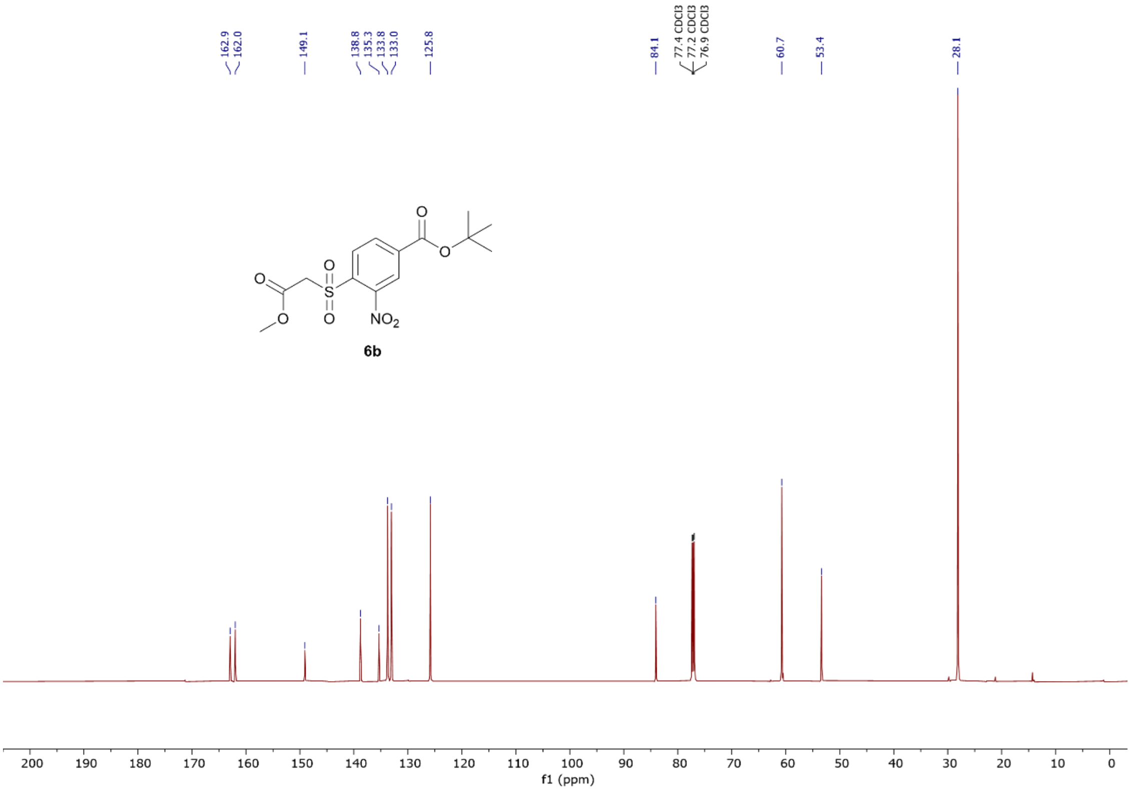

Figure S167. ^{13}C NMR spectrum of compound **6b** (151 MHz, $CDCl_3$).

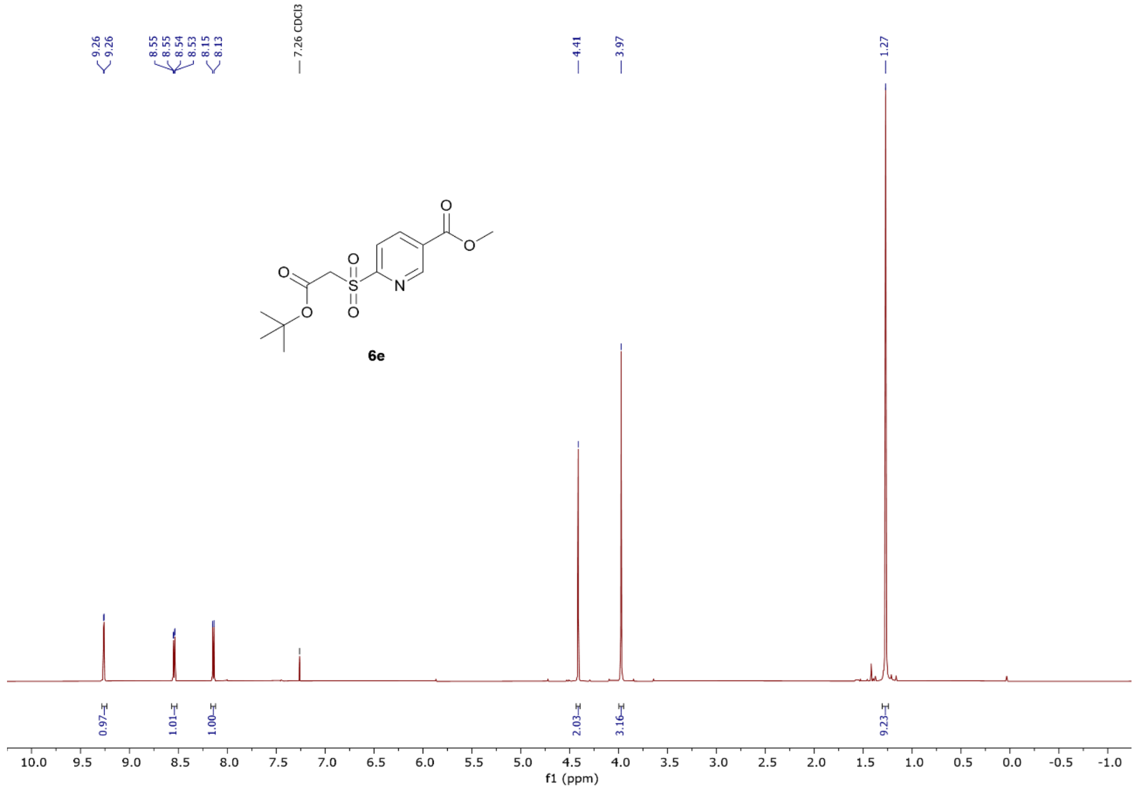

Figure S168. ^{1}H NMR spectrum of compound **6e** (600 MHz, $CDCl_3$).

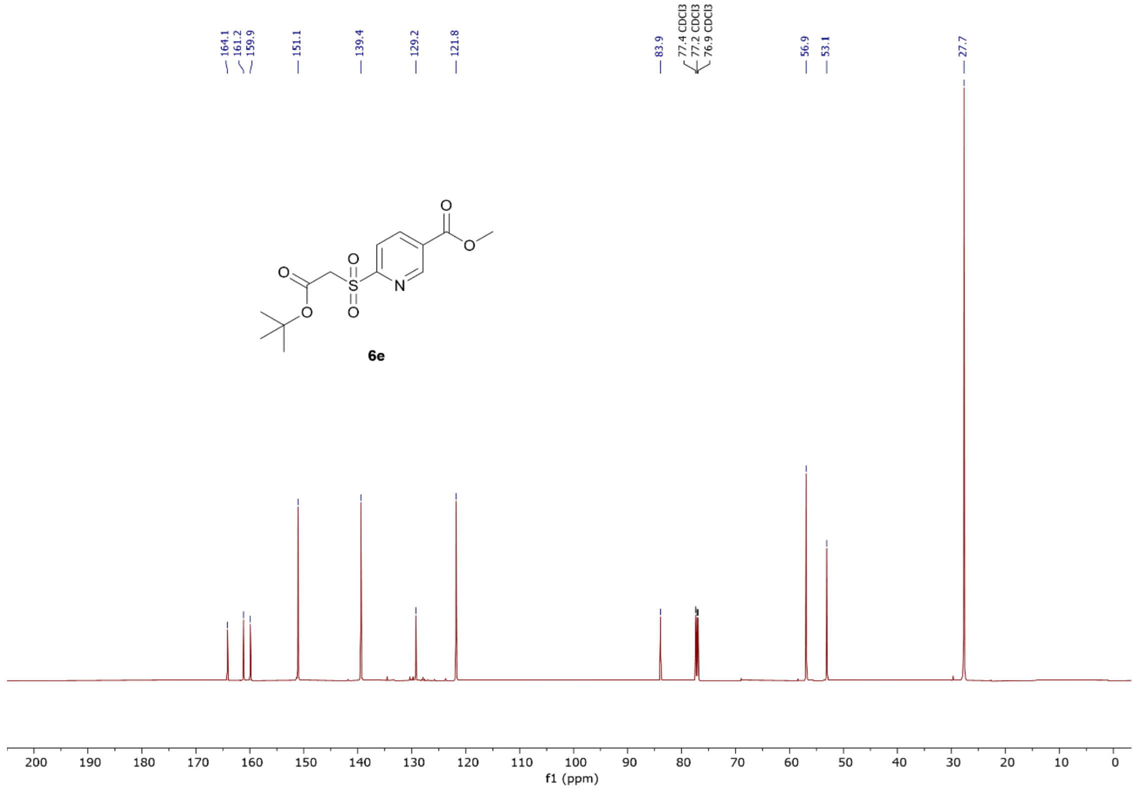

Figure S169. ^{13}C NMR spectrum of compound **6e** (151 MHz, $CDCl_3$).

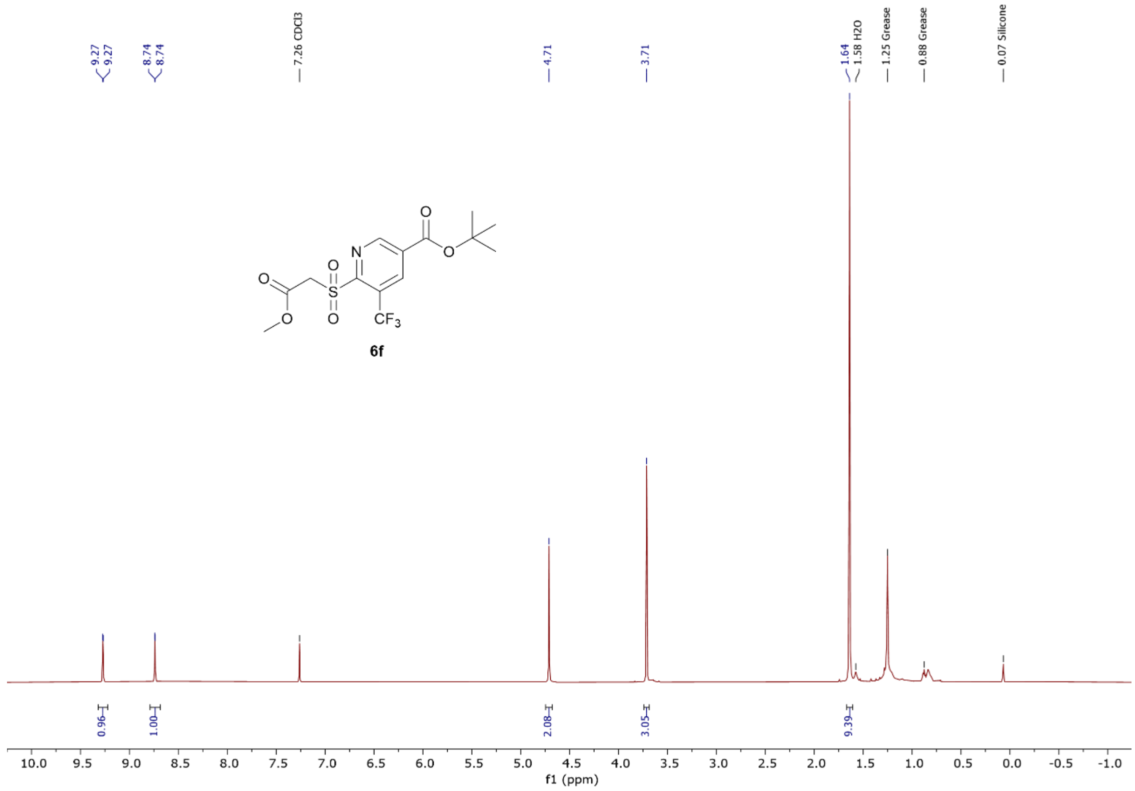

Figure S170. ^{1}H NMR spectrum of compound **6f** (600 MHz, $CDCl_3$).

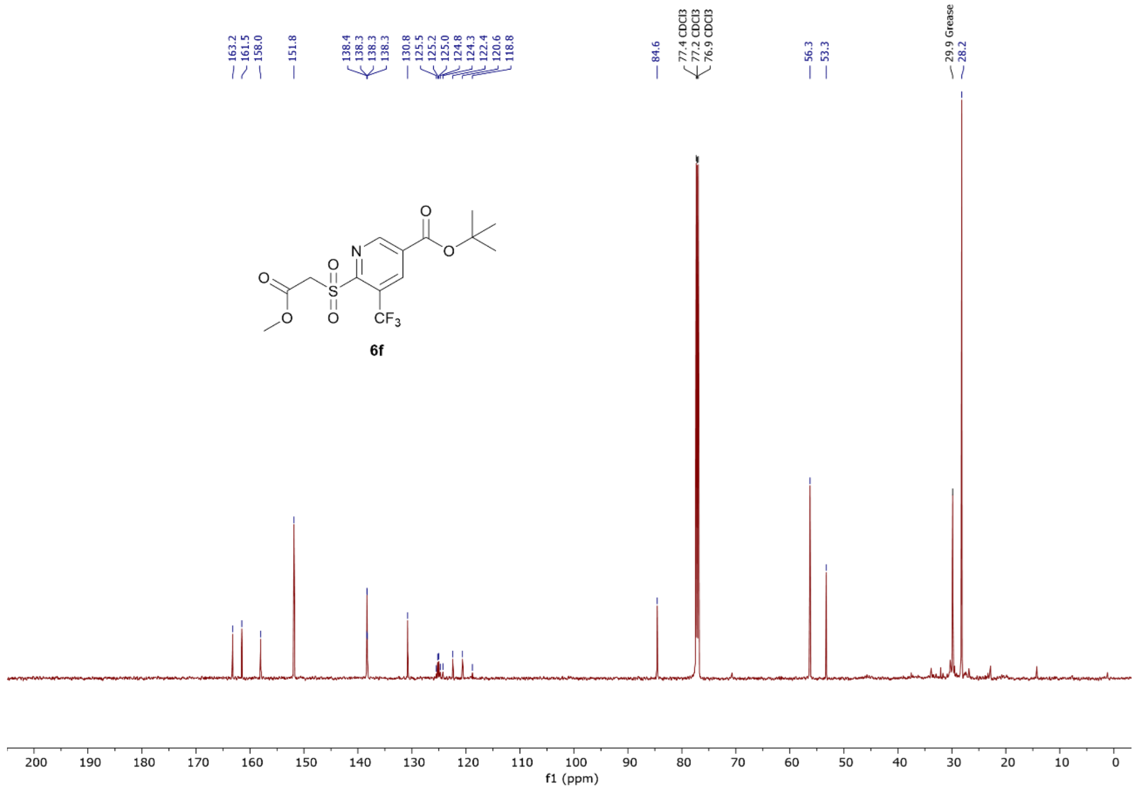

Figure S171. ^{13}C NMR spectrum of compound **6f** (151 MHz, $CDCl_3$).

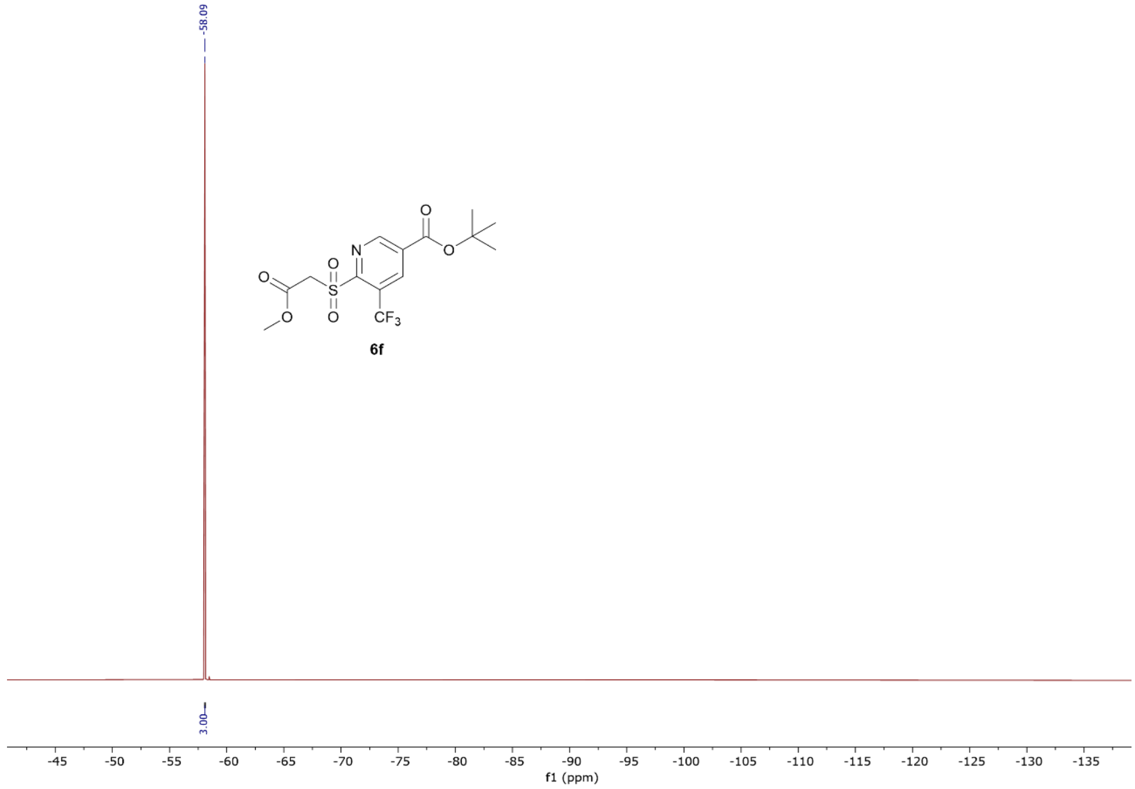

Figure S172. ^{19}F NMR spectrum of compound **6f** (564 MHz, $CDCl_3$).

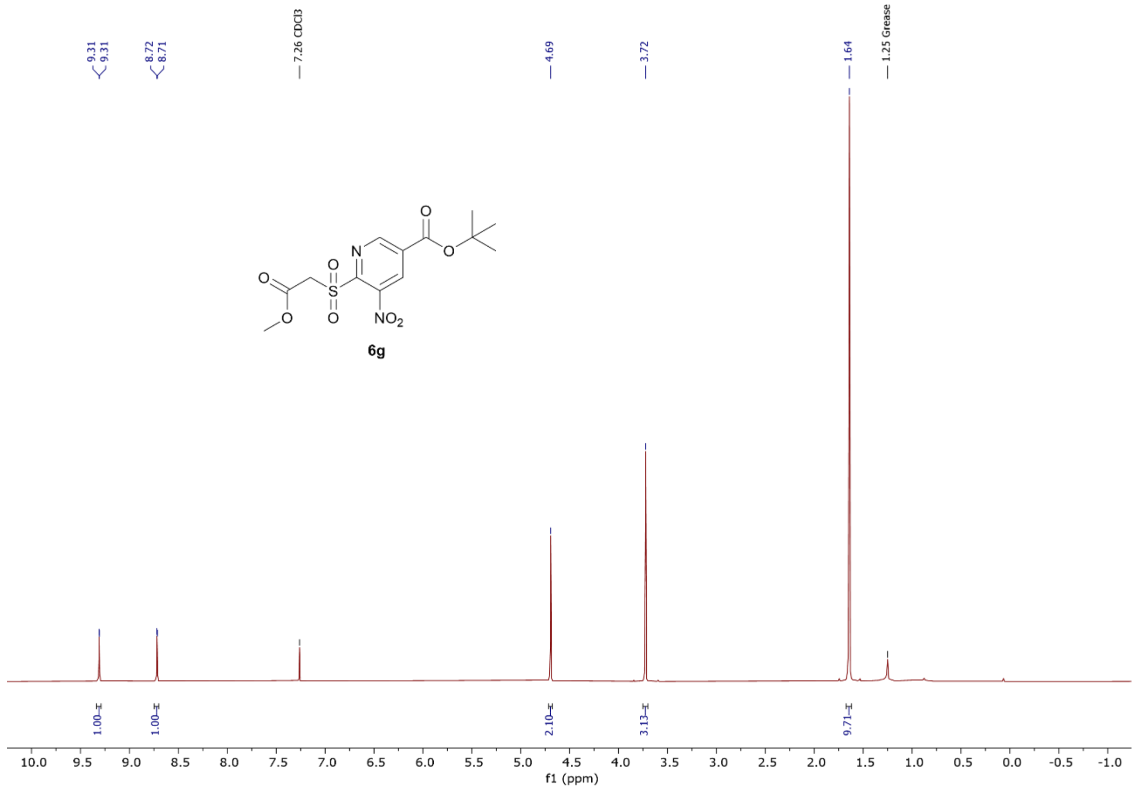

Figure S173. ^{1}H NMR spectrum of compound **6g** (600 MHz, $CDCl_3$).

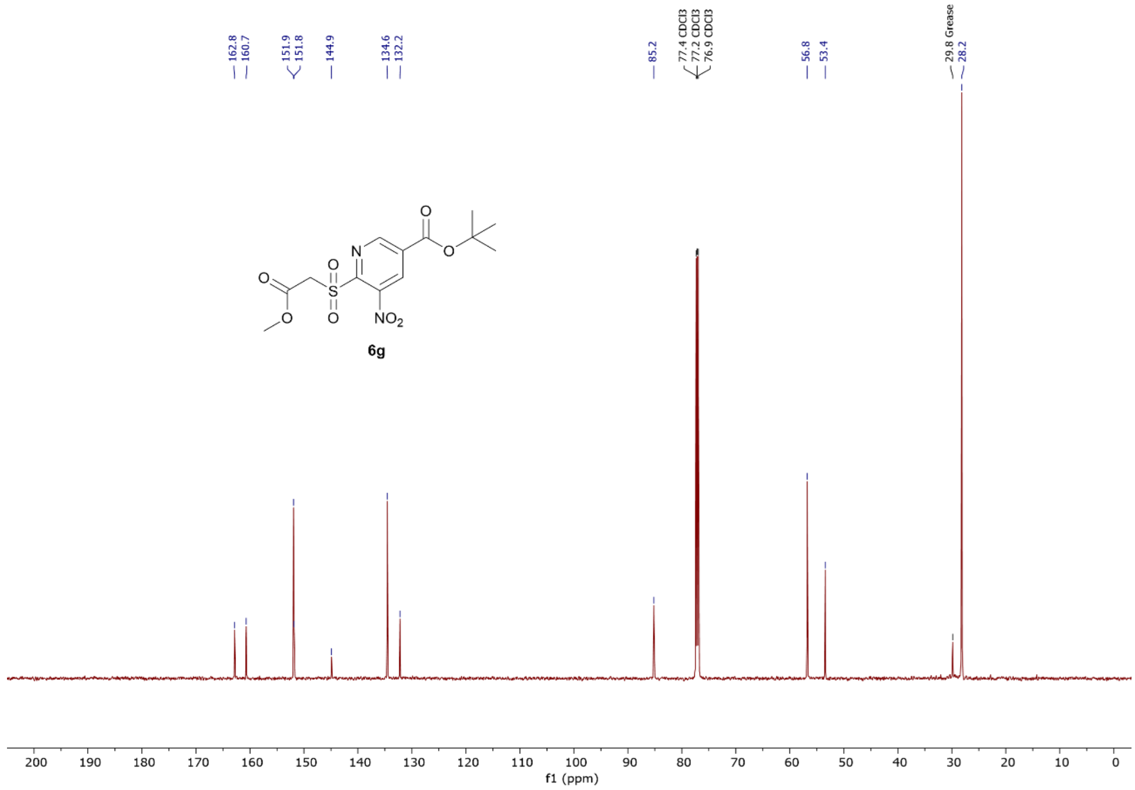

Figure S174. ^{13}C NMR spectrum of compound **6g** (151 MHz, $CDCl_3$).

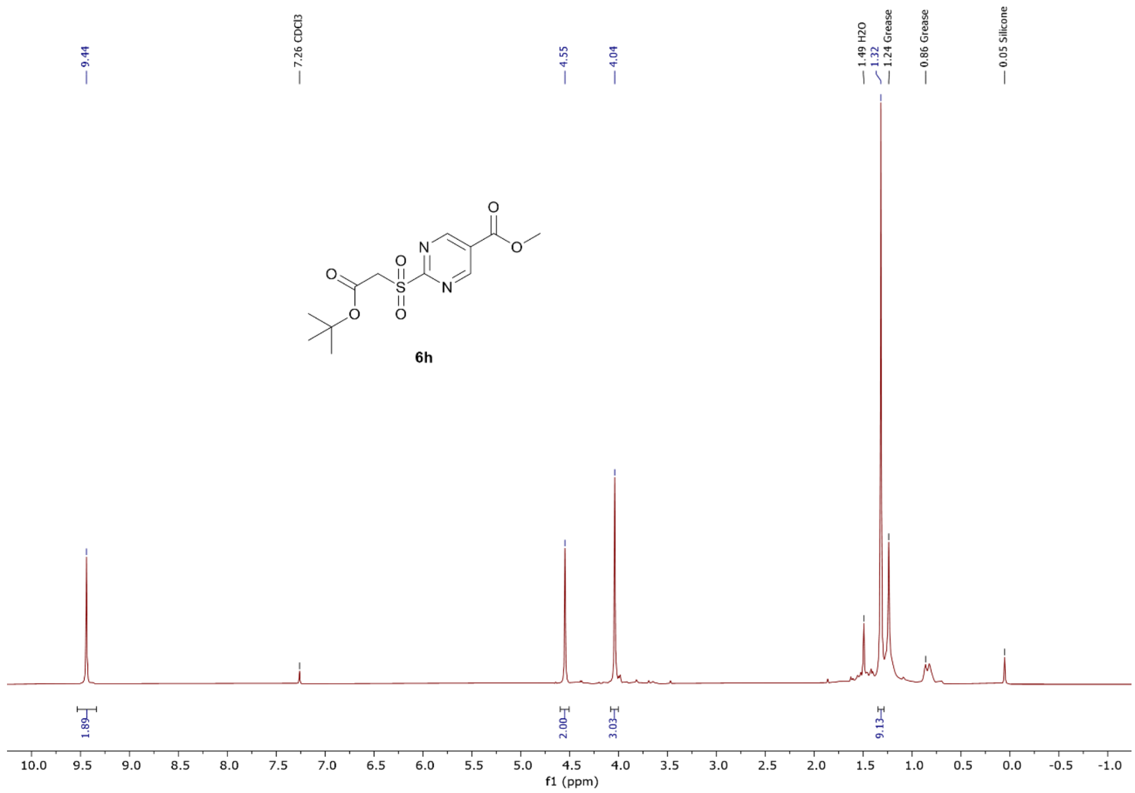

Figure S175. ^{1}H NMR spectrum of compound **6h** (600 MHz, $CDCl_3$).

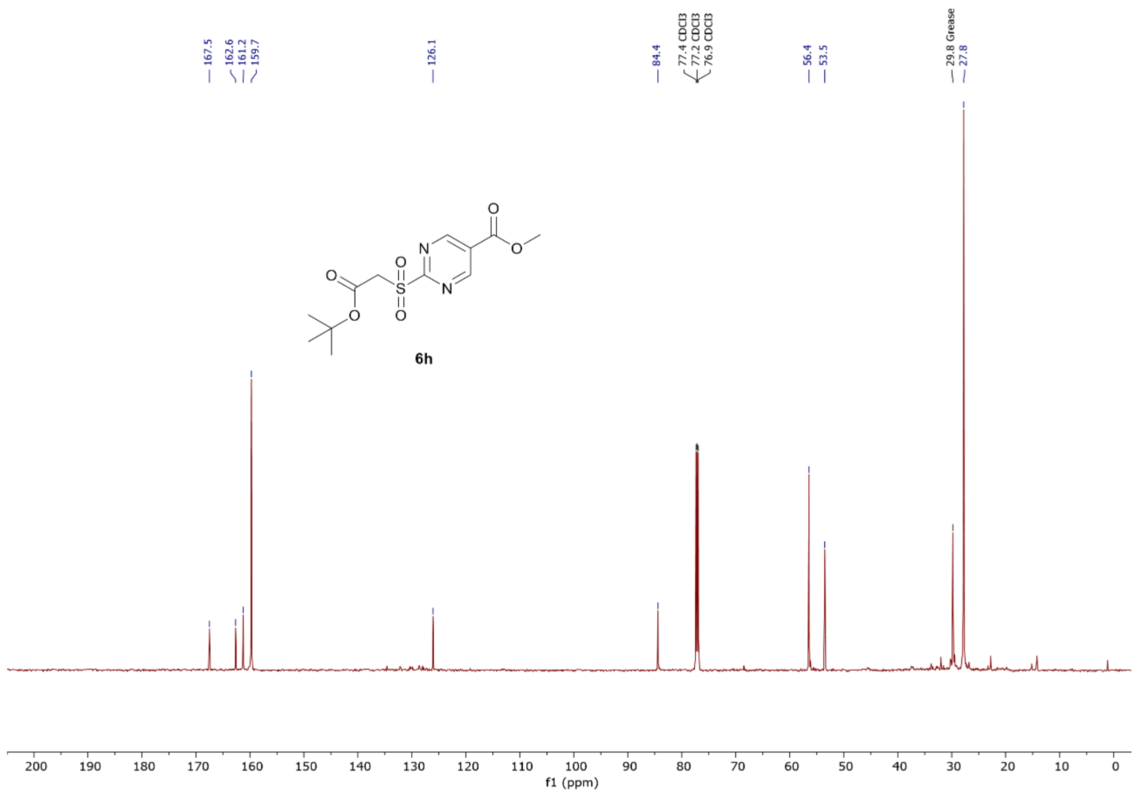

Figure S176. ^{13}C NMR spectrum of compound **6h** (151 MHz, $CDCl_3$).

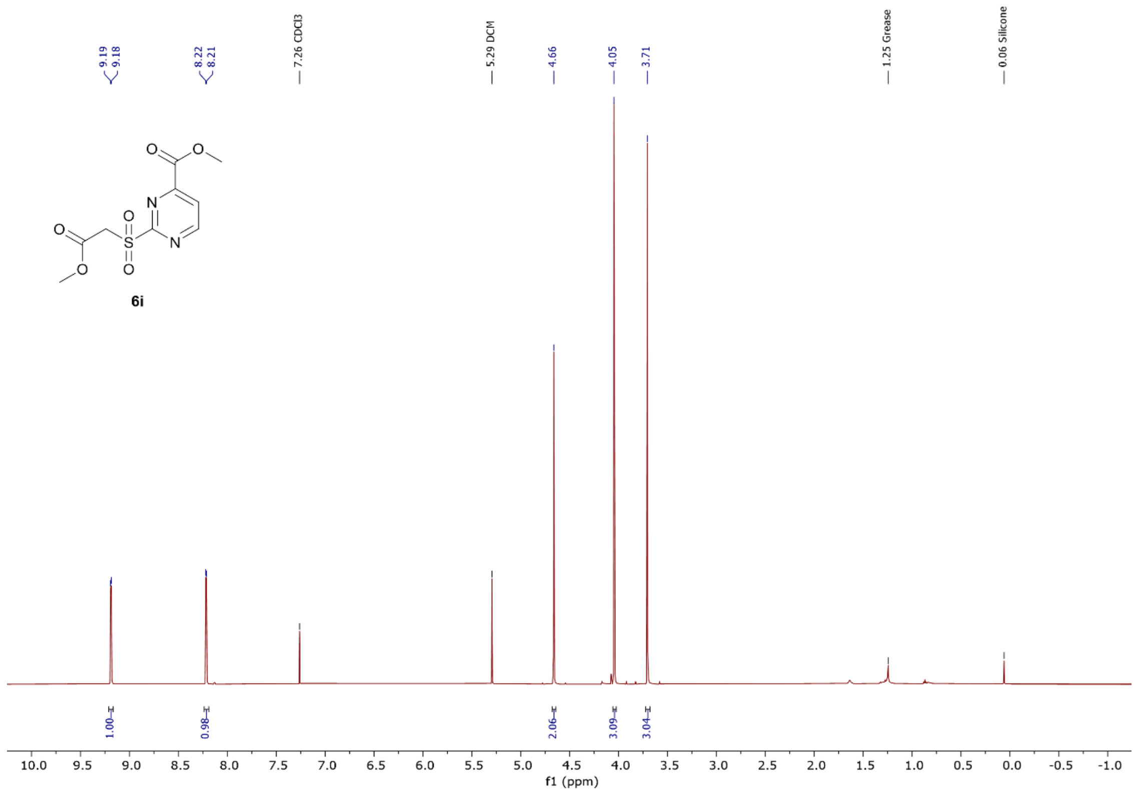

Figure S177. ^{1}H NMR spectrum of compound **6i** (600 MHz, $CDCl_3$).

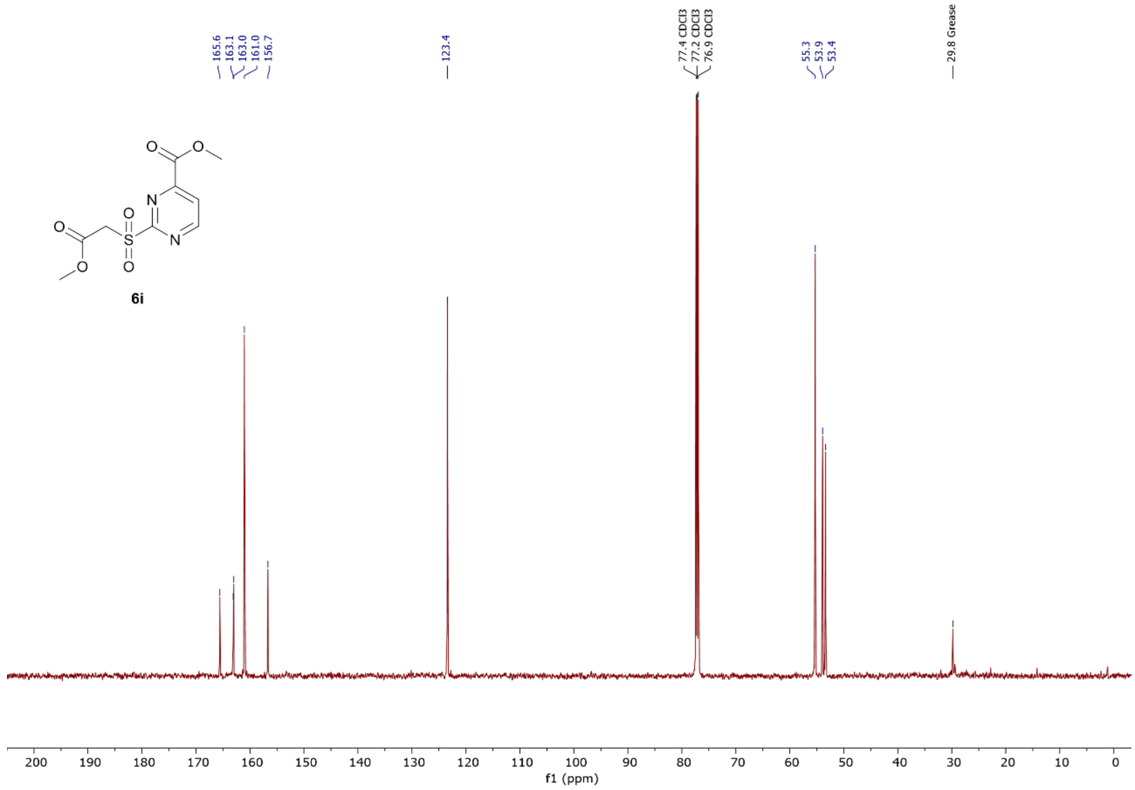

Figure S178. ^{13}C NMR spectrum of compound **6i** (151 MHz, $CDCl_3$).

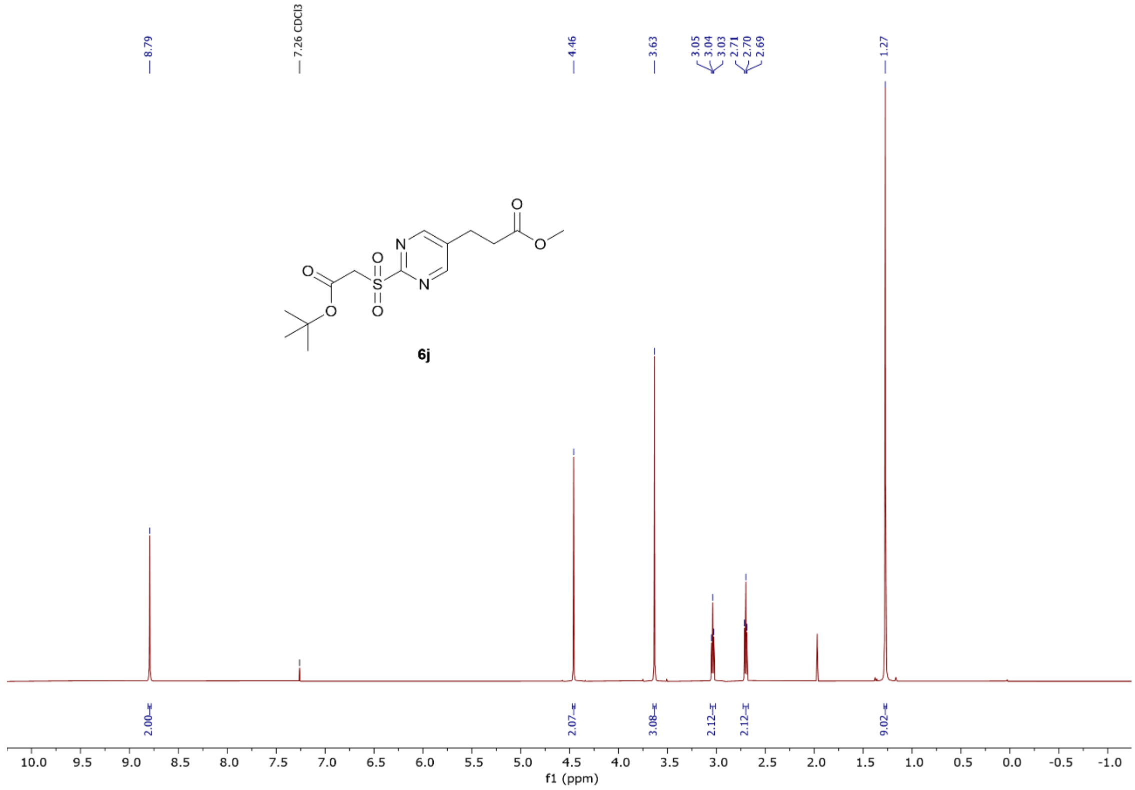

Figure S179. ^{1}H NMR spectrum of compound **6j** (600 MHz, $CDCl_3$).

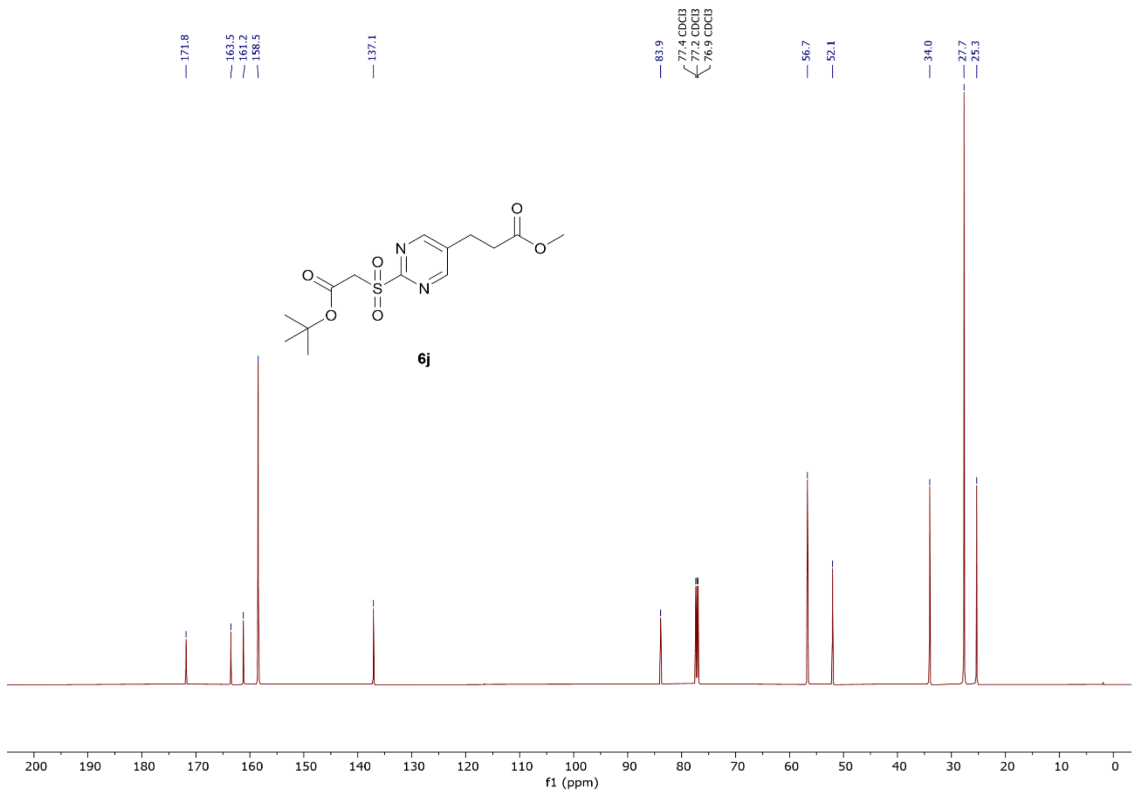

Figure S180. ^{13}C NMR spectrum of compound **6j** (151 MHz, $CDCl_3$).

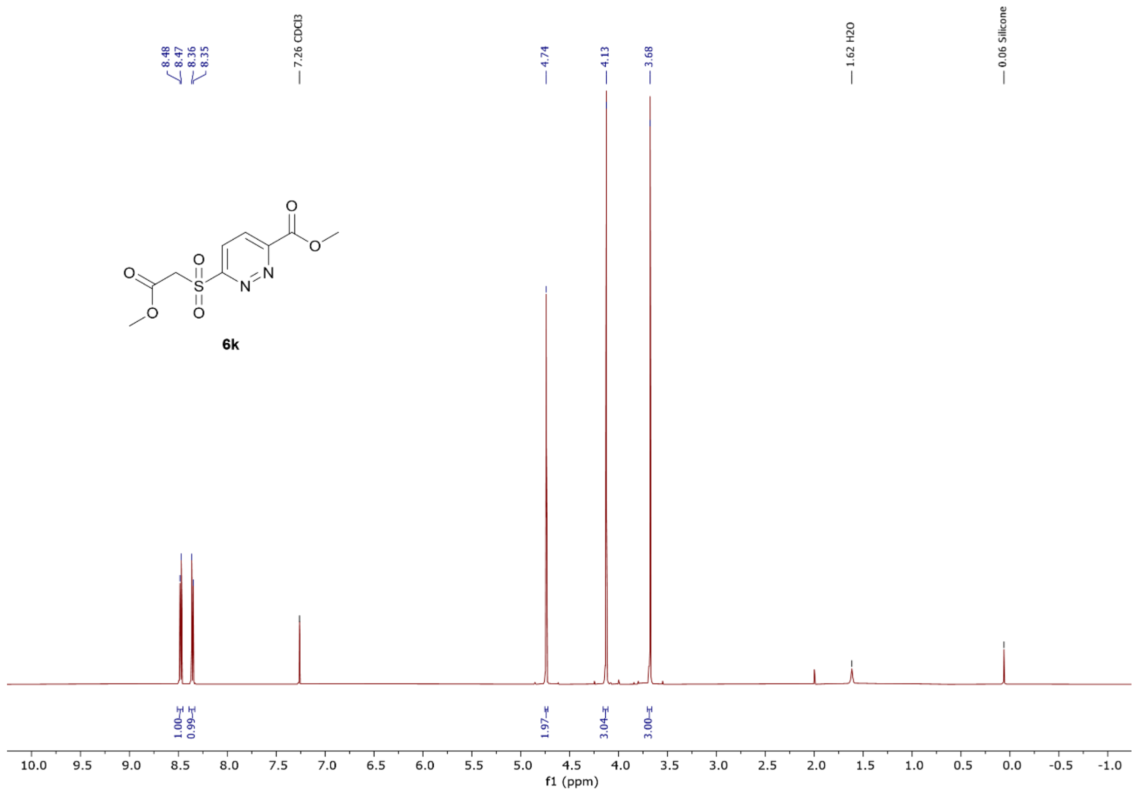

Figure S181. ^{1}H NMR spectrum of compound **6k** (600 MHz, $CDCl_3$).

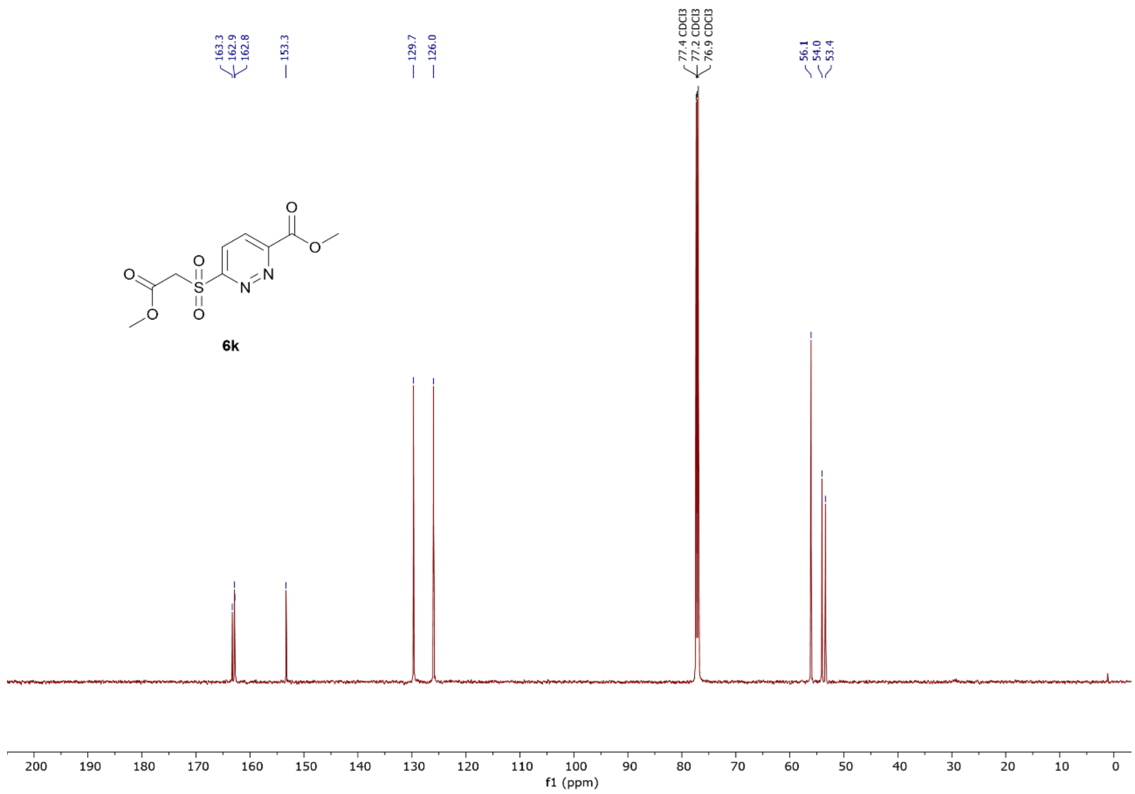

Figure S182. ^{13}C NMR spectrum of compound **6k** (151 MHz, $CDCl_3$).

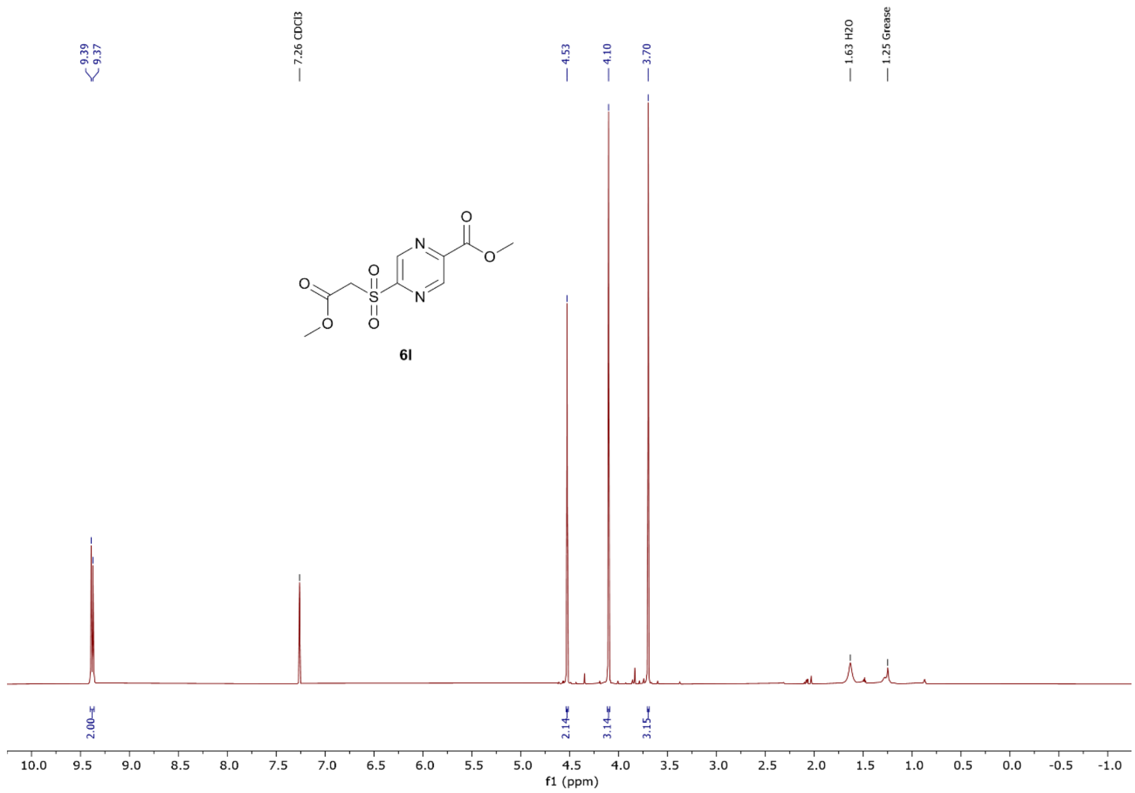

Figure S183. ^{1}H NMR spectrum of compound **6l** (800 MHz, $CDCl_3$).

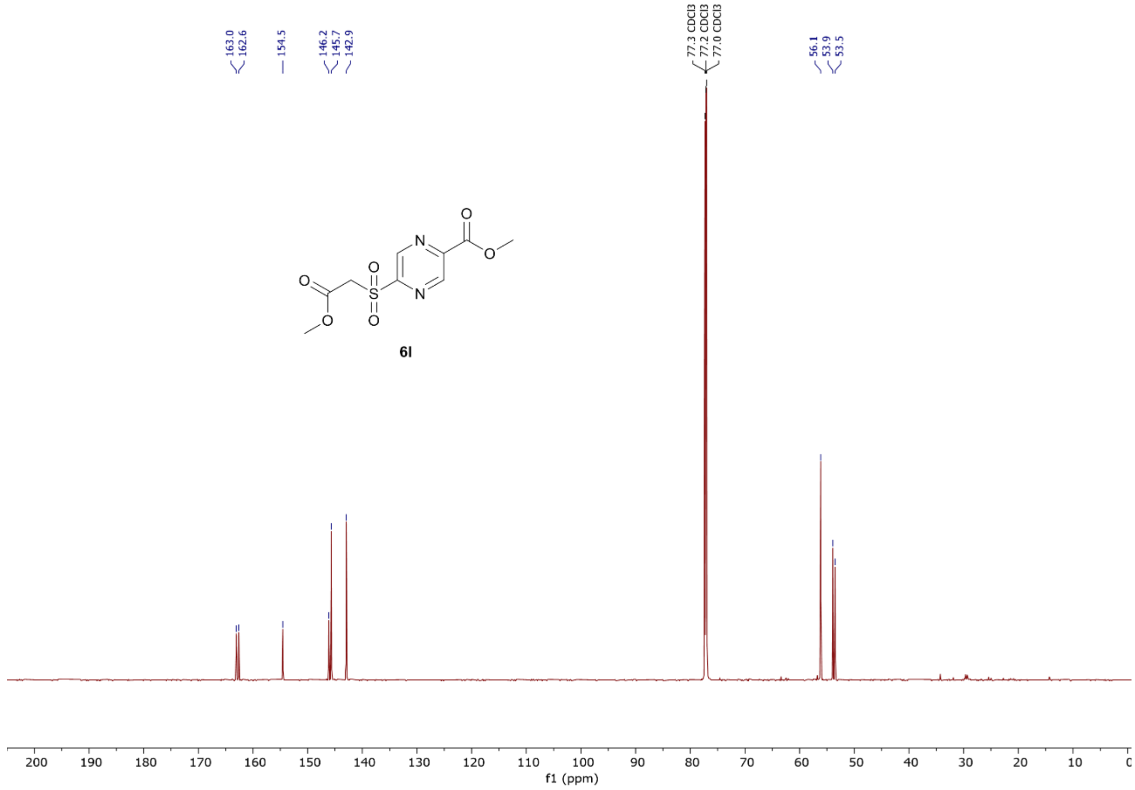

Figure S184. ^{13}C NMR spectrum of compound **6l** (201 MHz, $CDCl_3$).

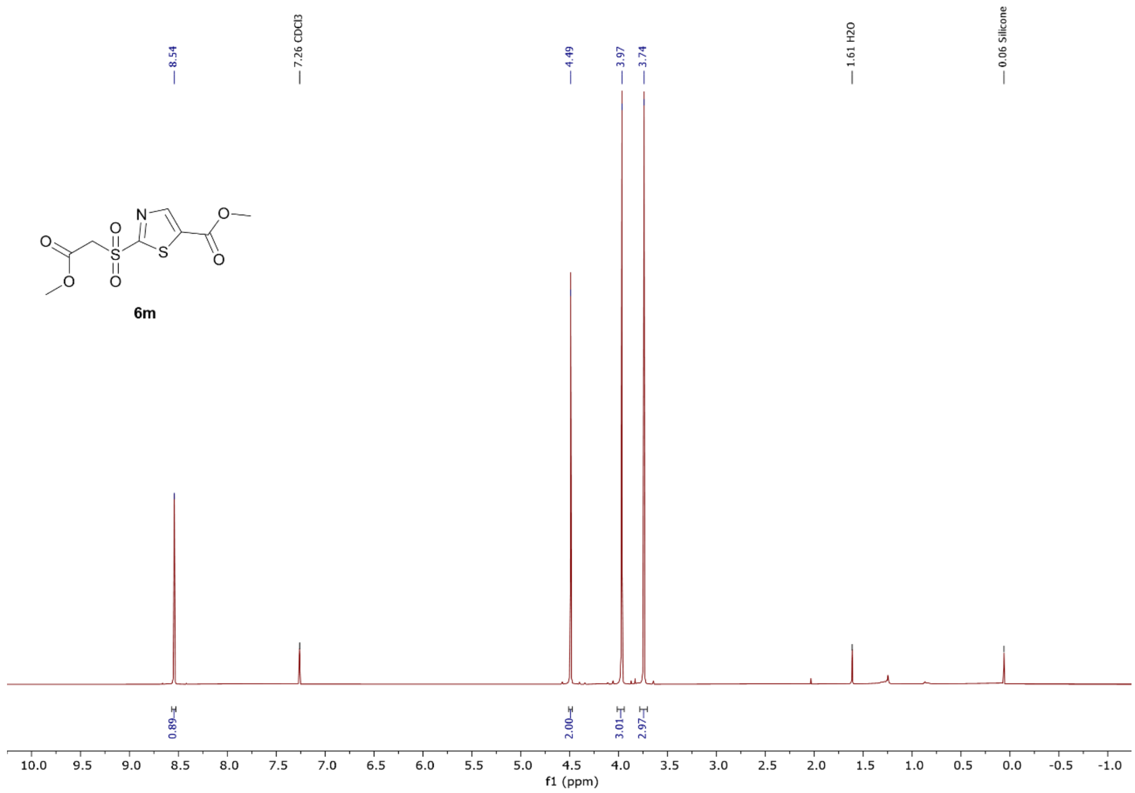

Figure S185. ^{1}H NMR spectrum of compound **6m** (800 MHz, $CDCl_3$).

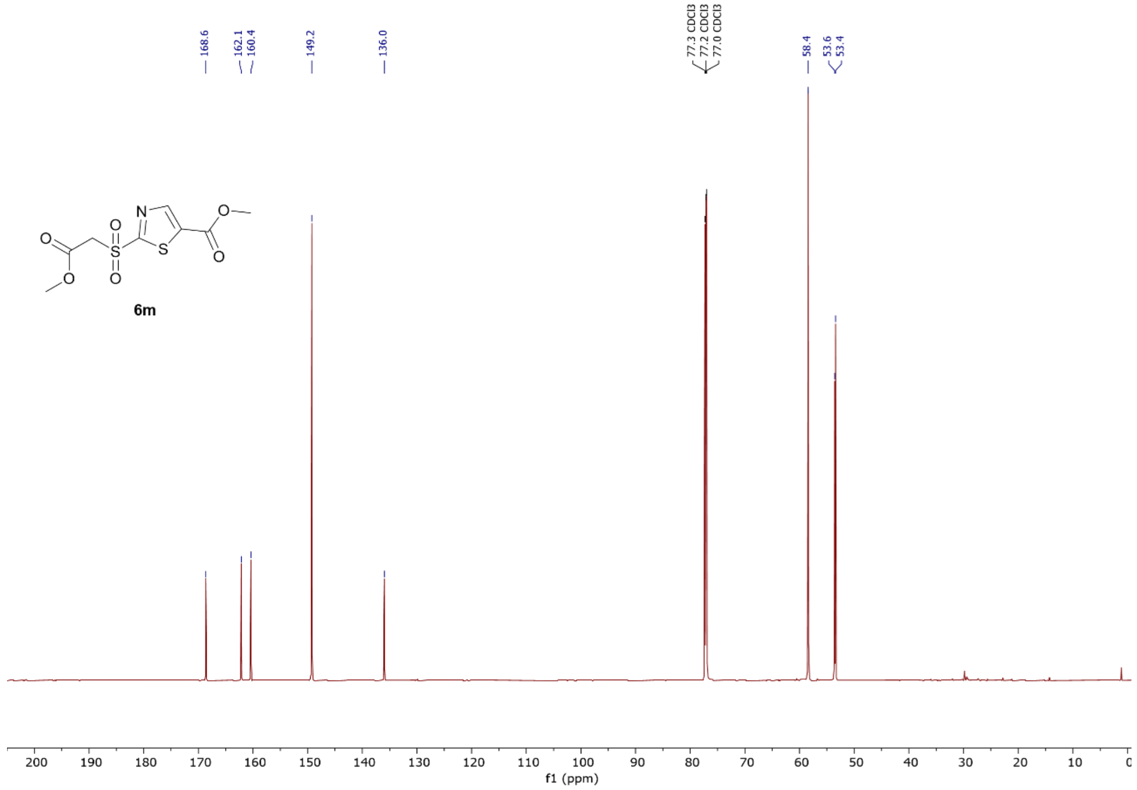

Figure S186. ^{13}C NMR spectrum of compound **6m** (201 MHz, $CDCl_3$).

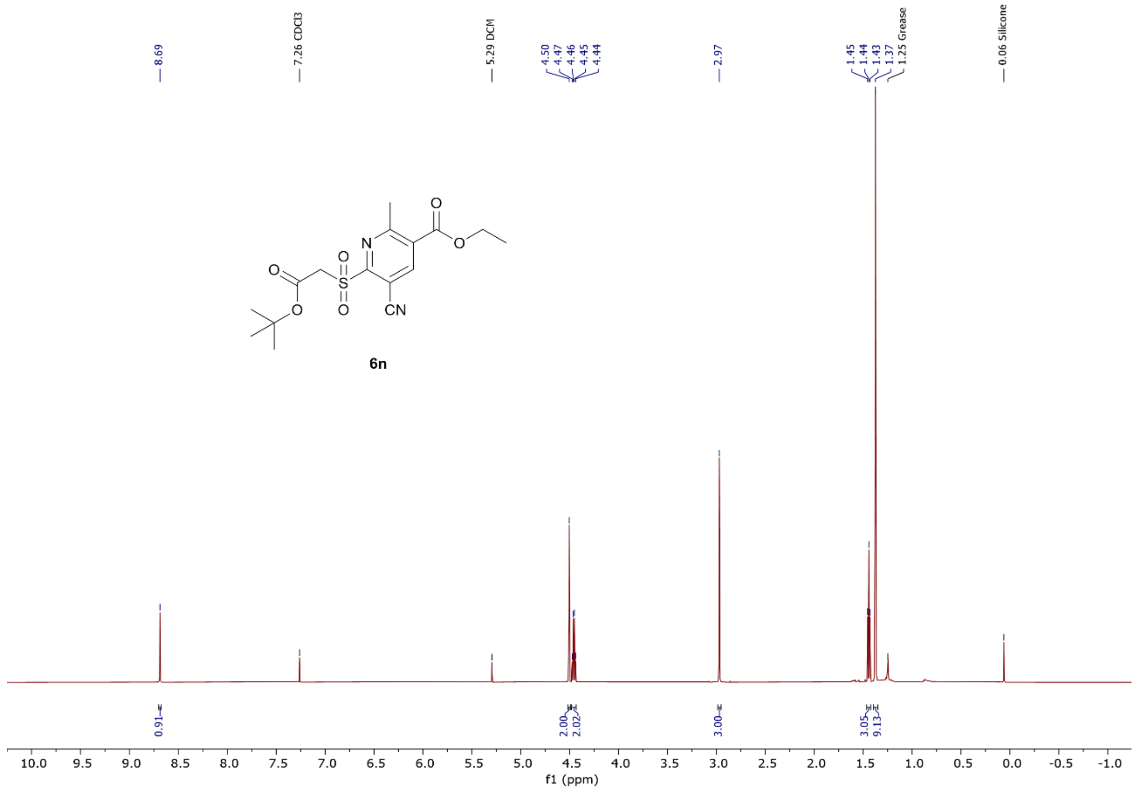

Figure S187. ^{1}H NMR spectrum of compound **6n** (600 MHz, $CDCl_3$).

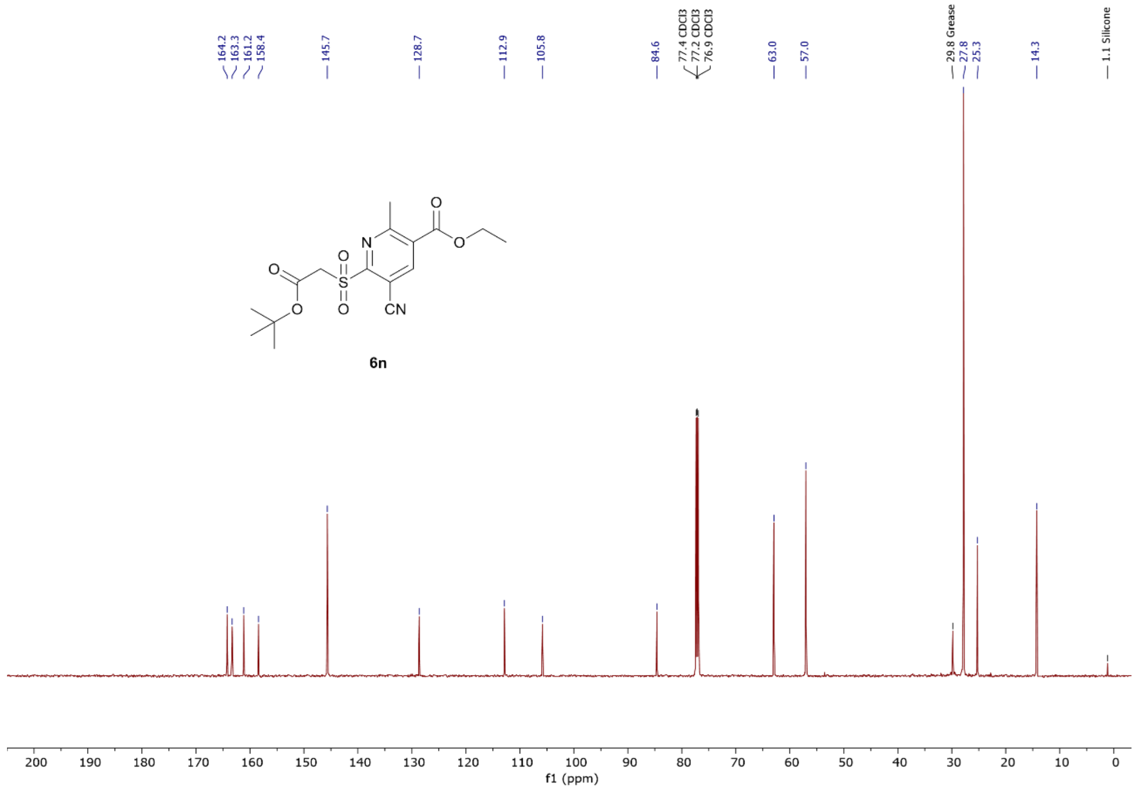

Figure S188. ^{13}C NMR spectrum of compound **6n** (151 MHz, $CDCl_3$).

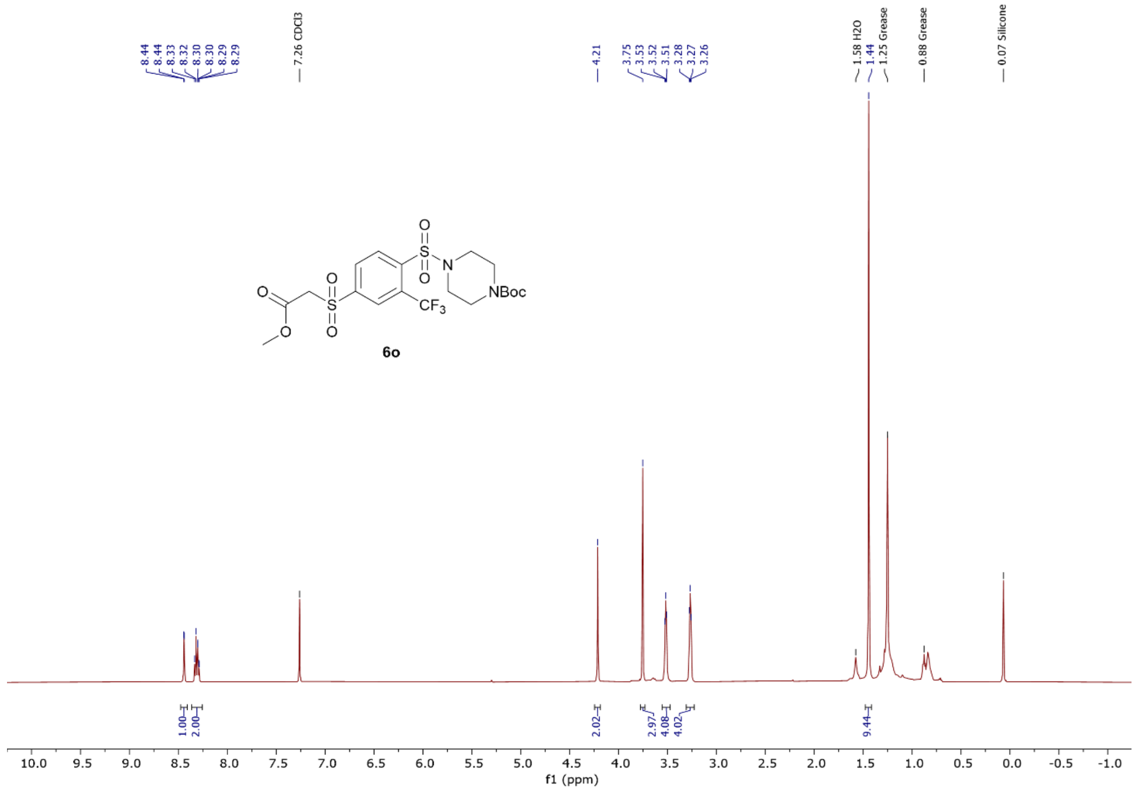

Figure S189. ^{1}H NMR spectrum of compound **6o** (600 MHz, $CDCl_3$).

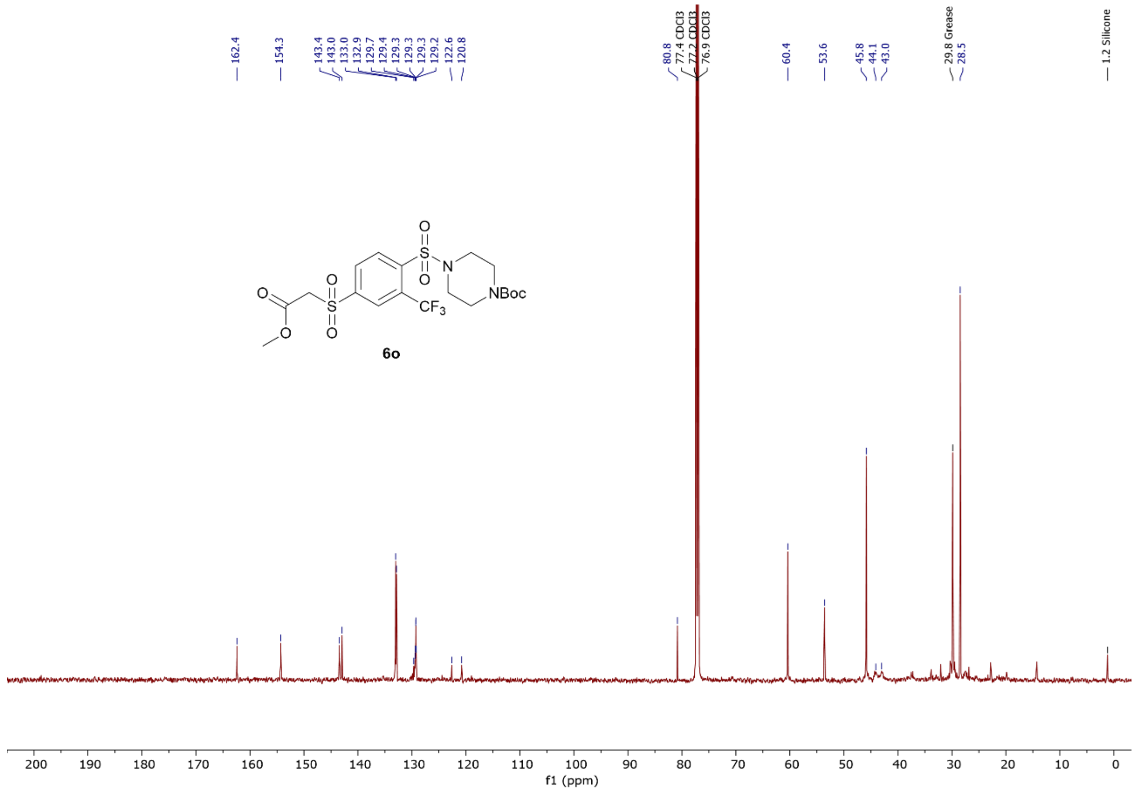

Figure S190. ^{13}C NMR spectrum of compound **6o** (151 MHz, $CDCl_3$).

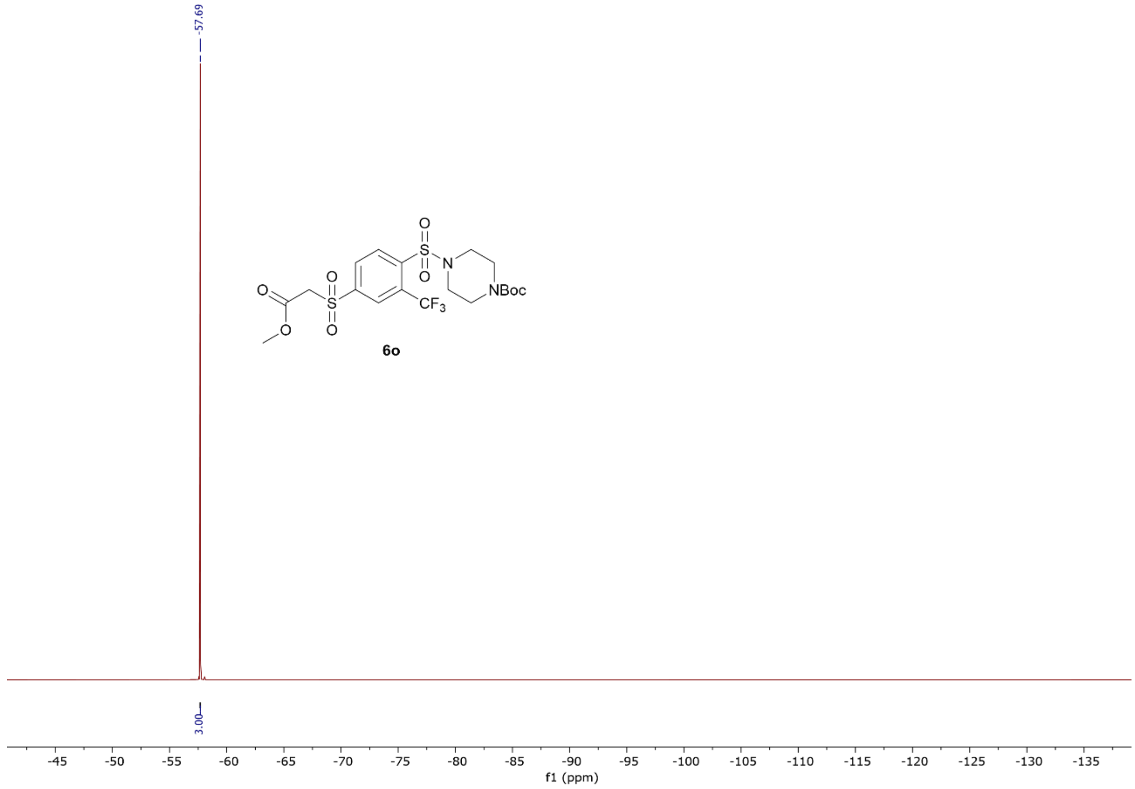

Figure S191. ^{19}F NMR spectrum of compound **6o** (564 MHz, $CDCl_3$).

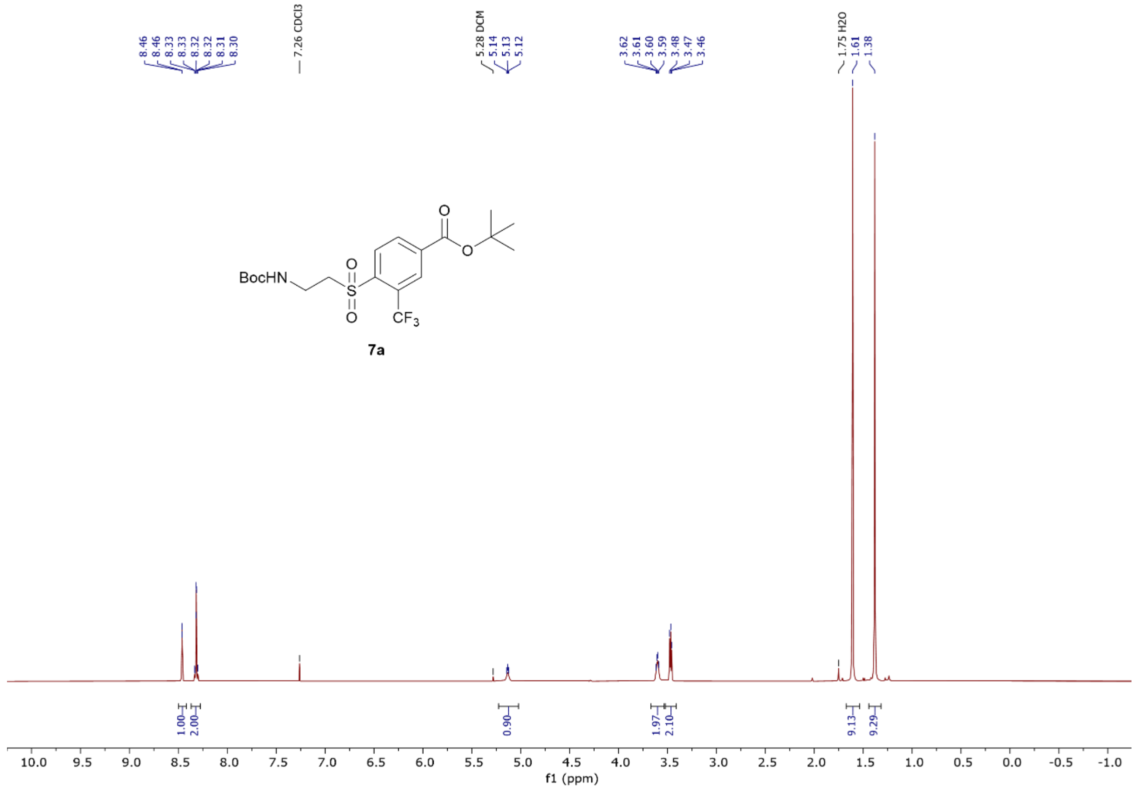

Figure S192. ^{1}H NMR spectrum of compound **7a** (600 MHz, $CDCl_3$).

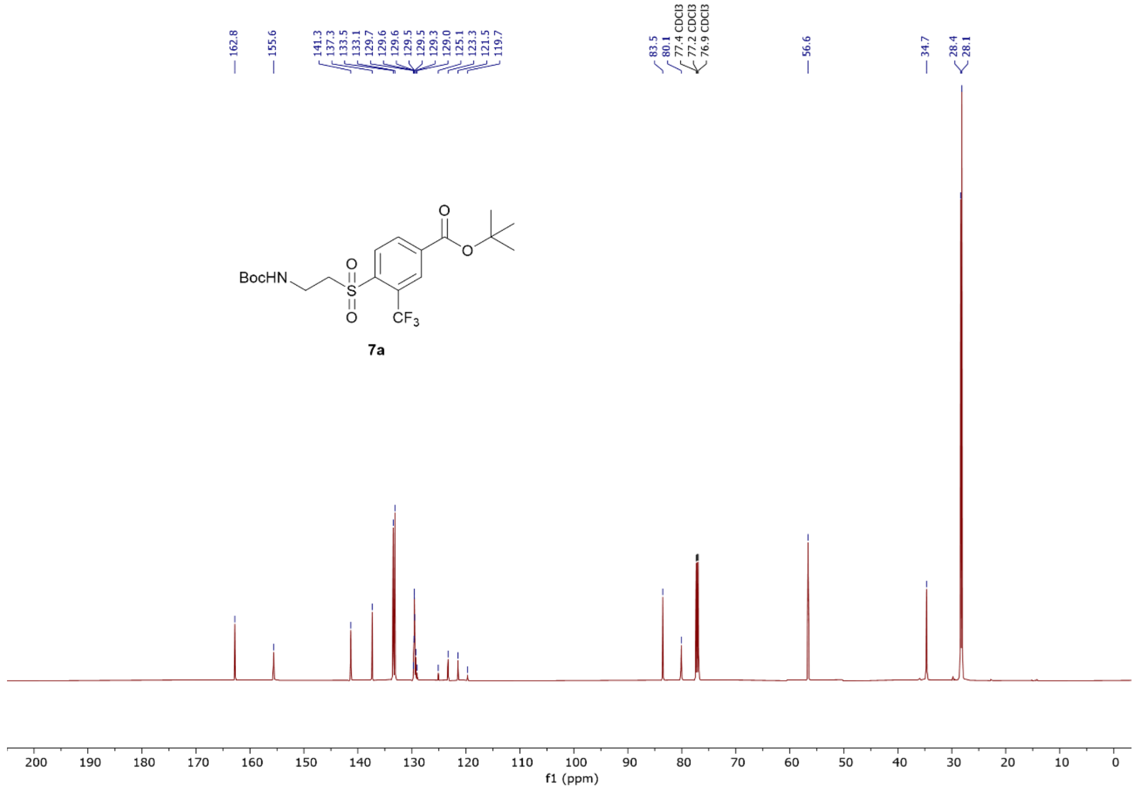

Figure S193. ^{13}C NMR spectrum of compound **7a** (151 MHz, $CDCl_3$).

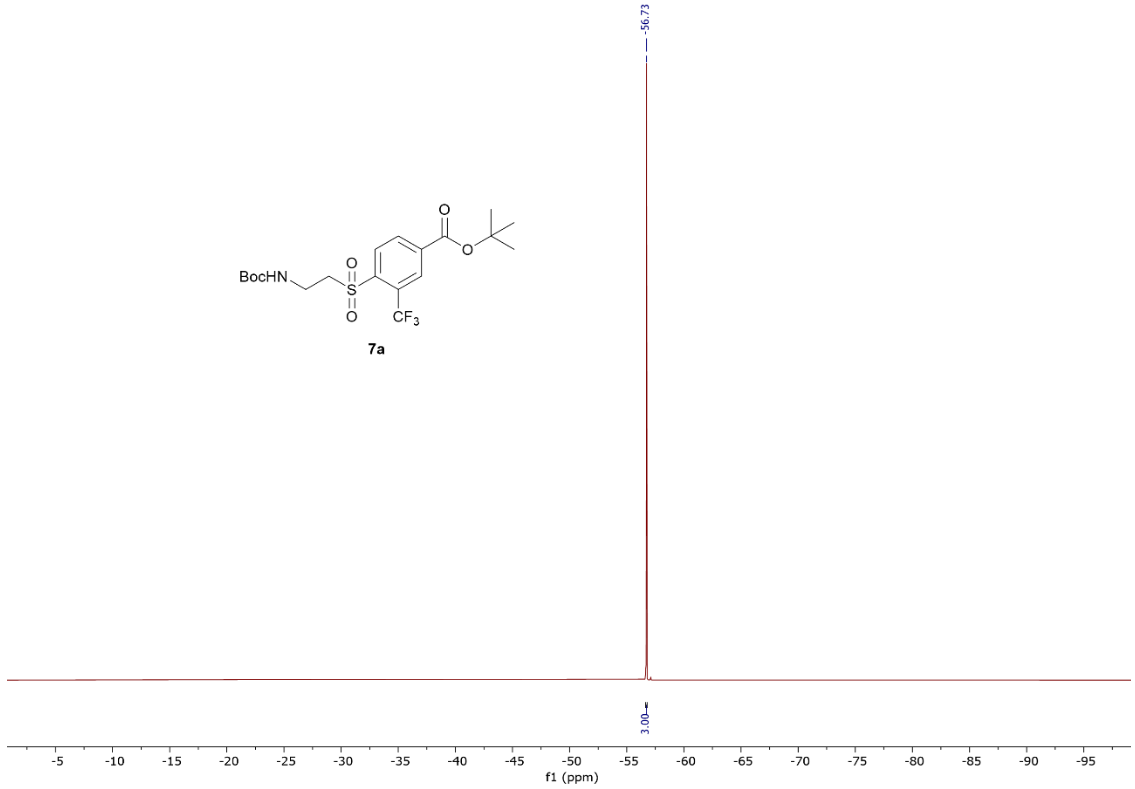

Figure S194. ^{19}F NMR spectrum of compound **7a** (564 MHz, $CDCl_3$).

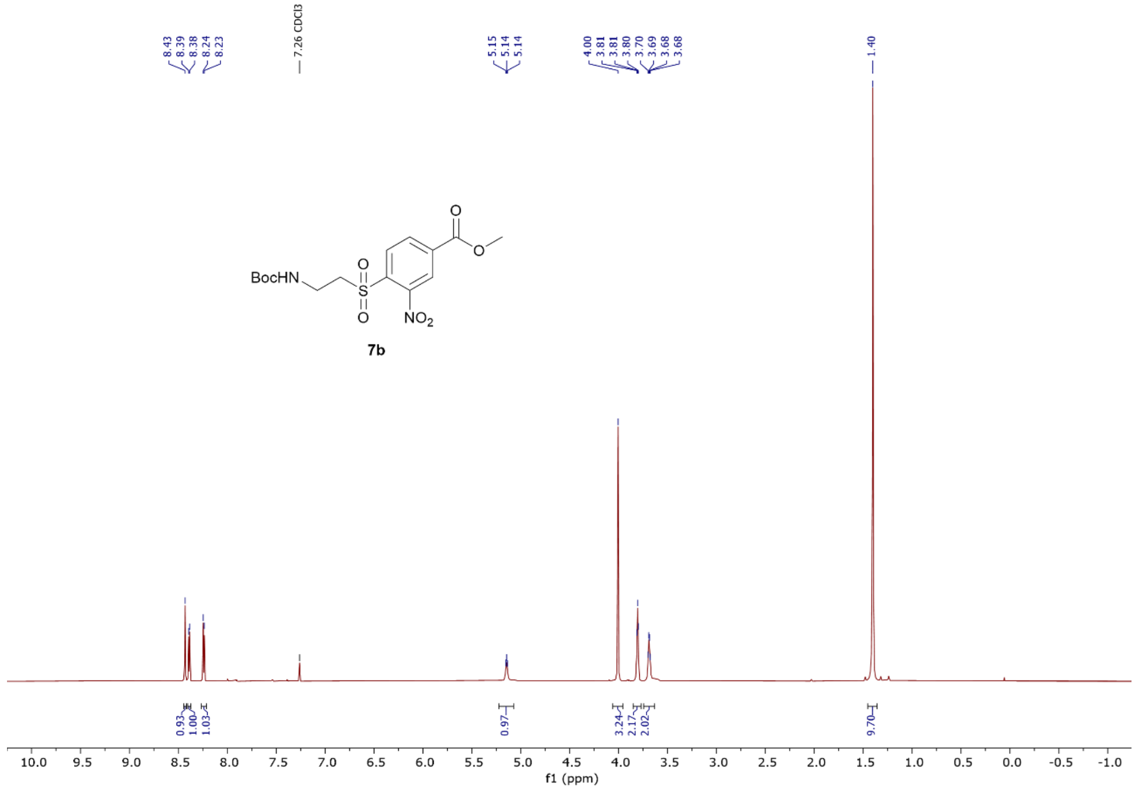

Figure S195. ^{1}H NMR spectrum of compound **7b** (800 MHz, $CDCl_3$).

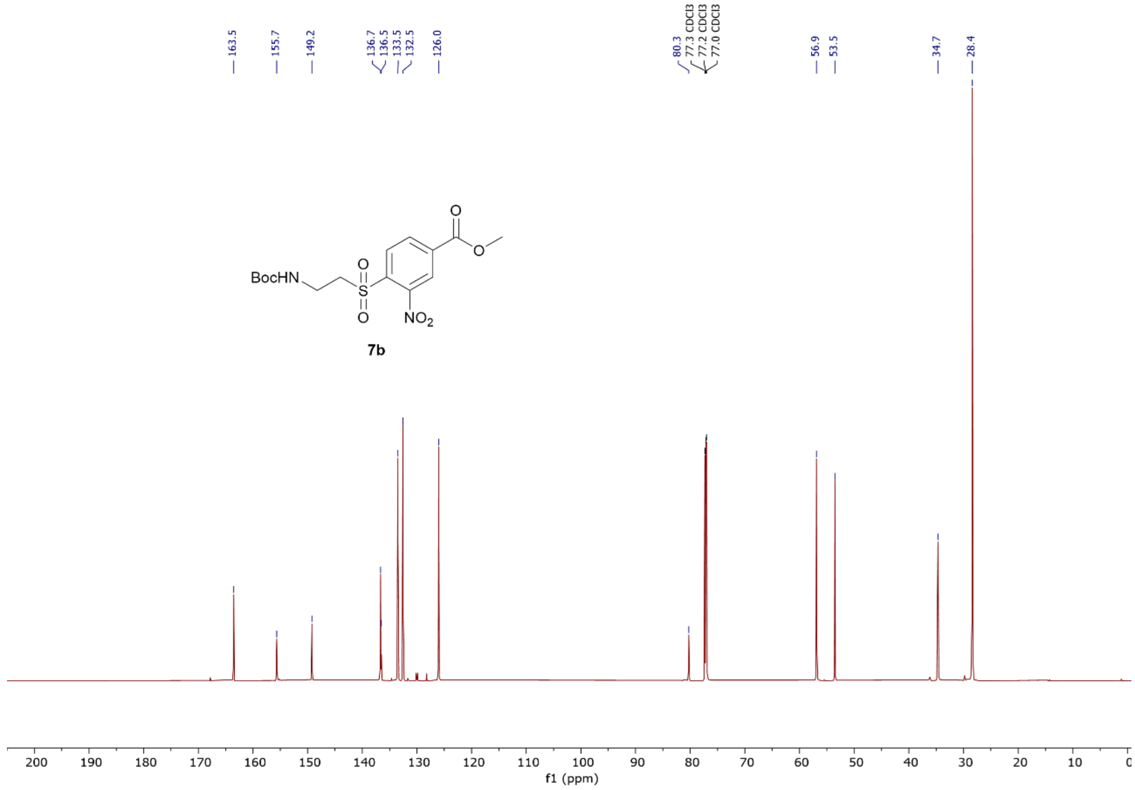

Figure S196. ^{13}C NMR spectrum of compound **7b** (201 MHz, $CDCl_3$).

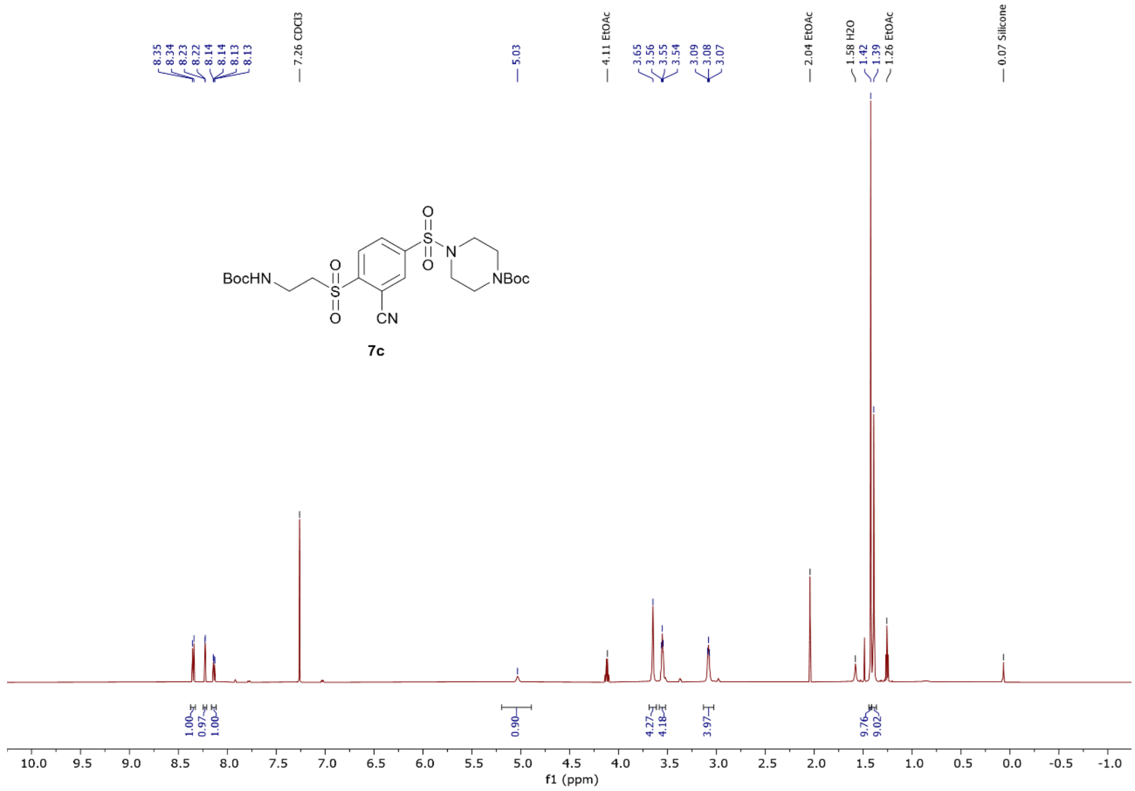

Figure S197. ^{1}H NMR spectrum of compound **7c** (600 MHz, $CDCl_3$).

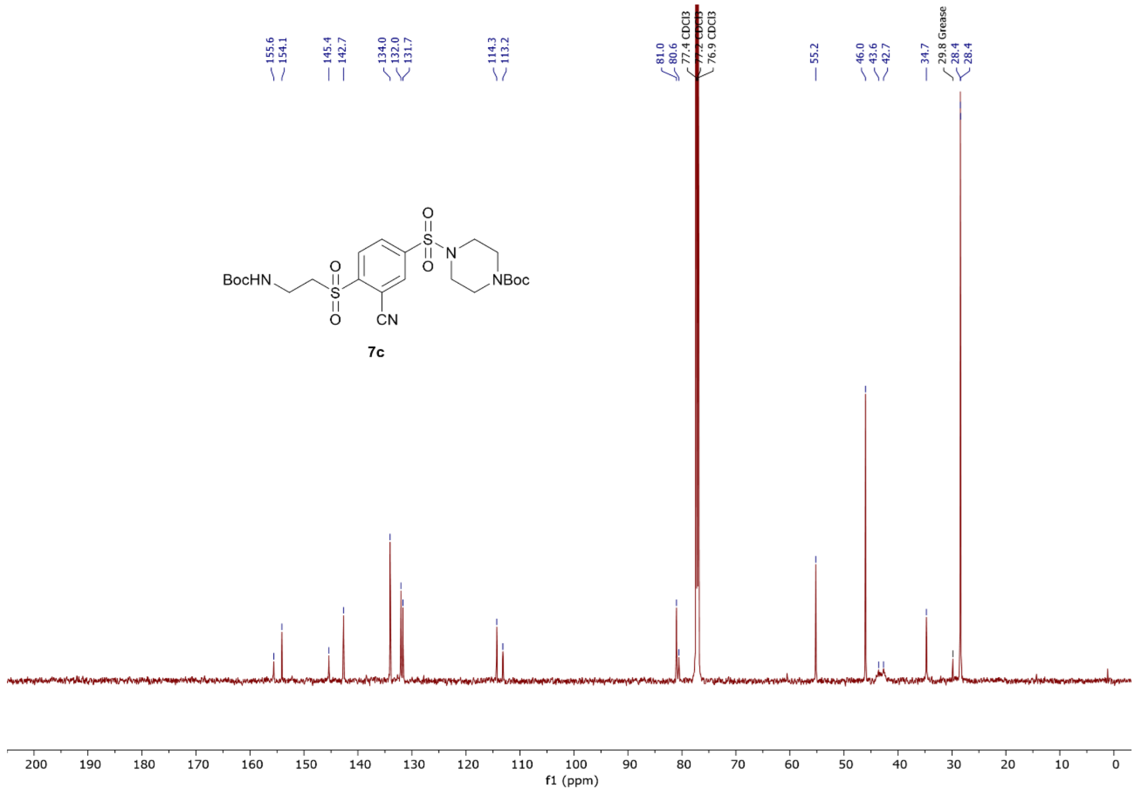

Figure S198. ^{13}C NMR spectrum of compound **7c** (151 MHz, $CDCl_3$).

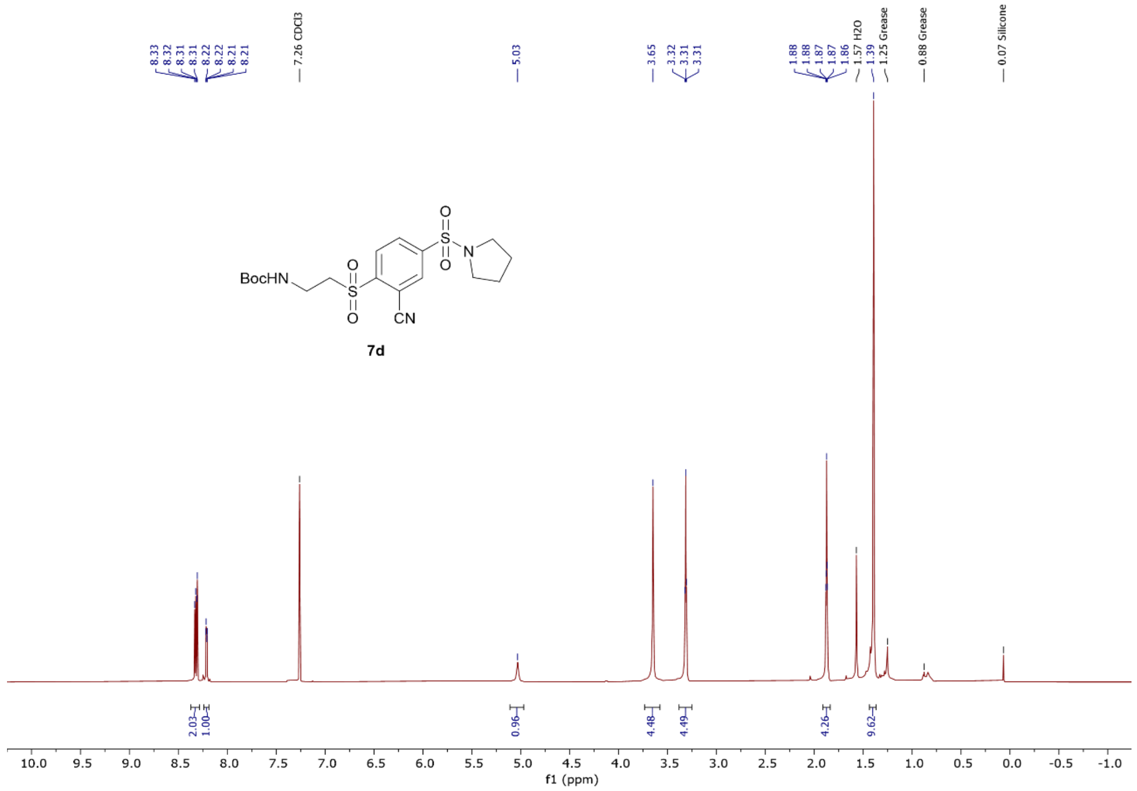

Figure S199. ^{1}H NMR spectrum of compound **7d** (800 MHz, $CDCl_3$).

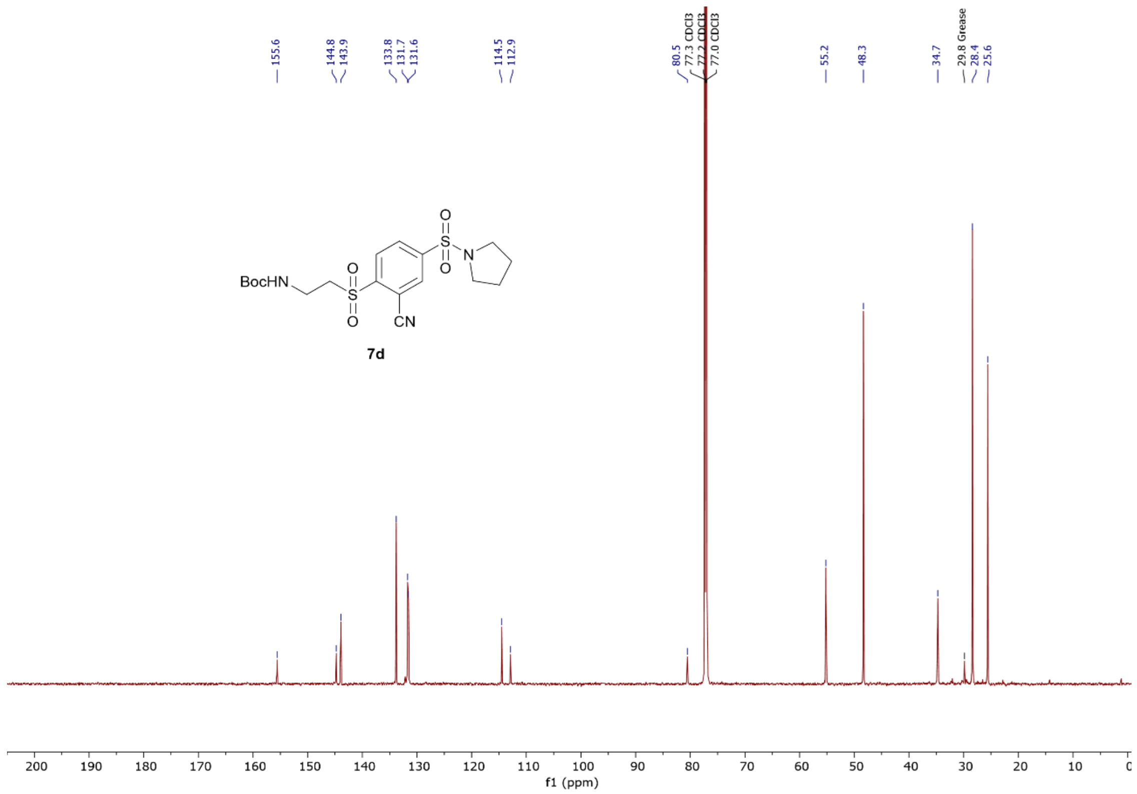

Figure S200. ^{13}C NMR spectrum of compound **7d** (201 MHz, $CDCl_3$).

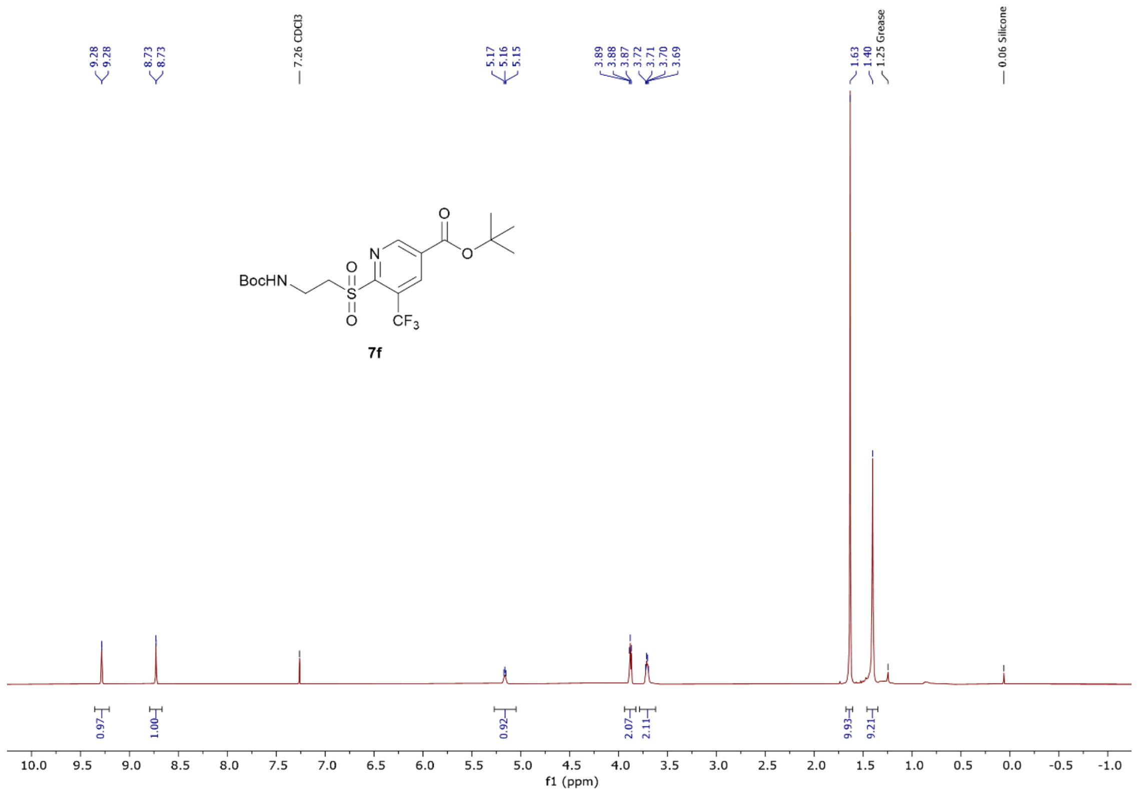

Figure S201. ^{1}H NMR spectrum of compound **7f** (600 MHz, $CDCl_3$).

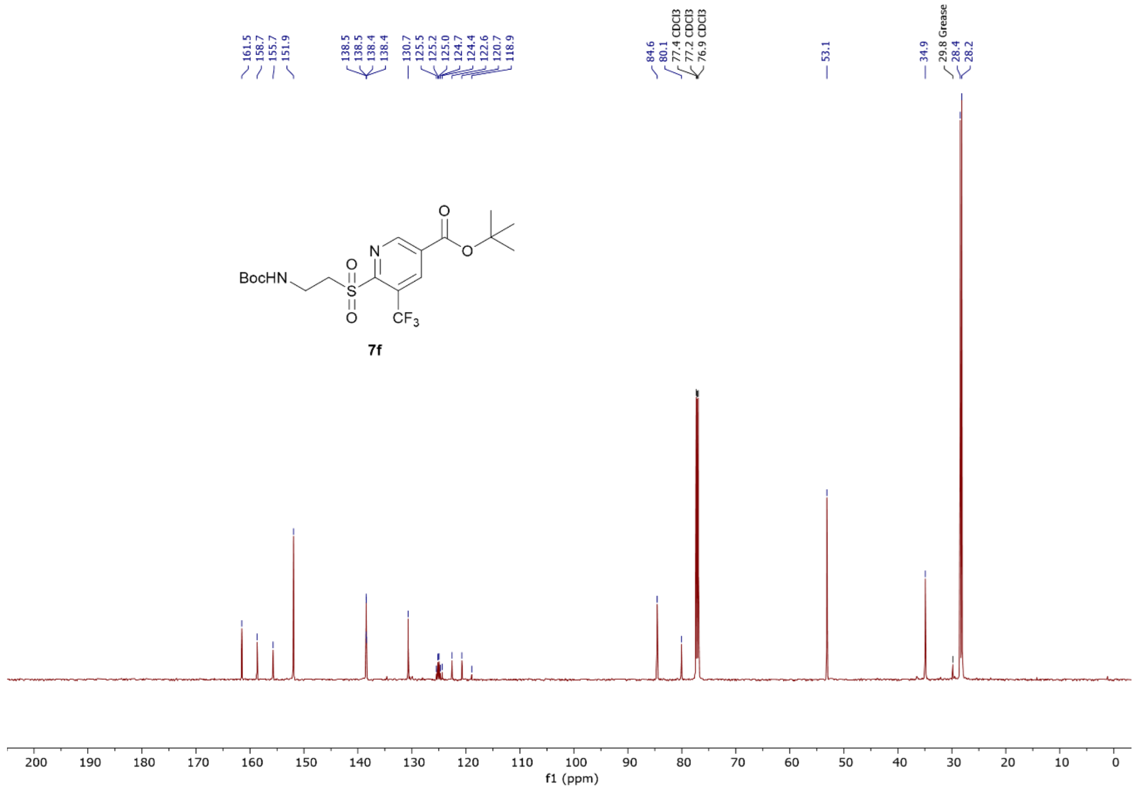

Figure S202. ^{13}C NMR spectrum of compound **7f** (151 MHz, $CDCl_3$).

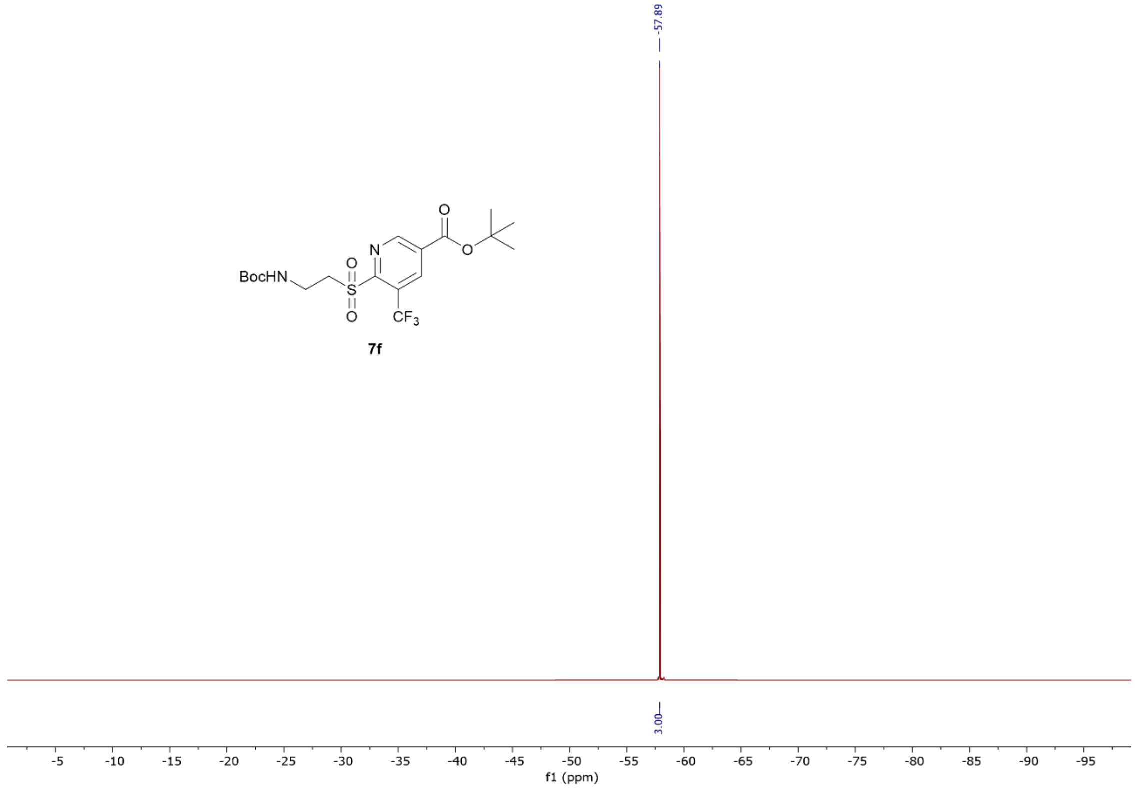

Figure S203. ^{19}F NMR spectrum of compound **7f** (564 MHz, $CDCl_3$).

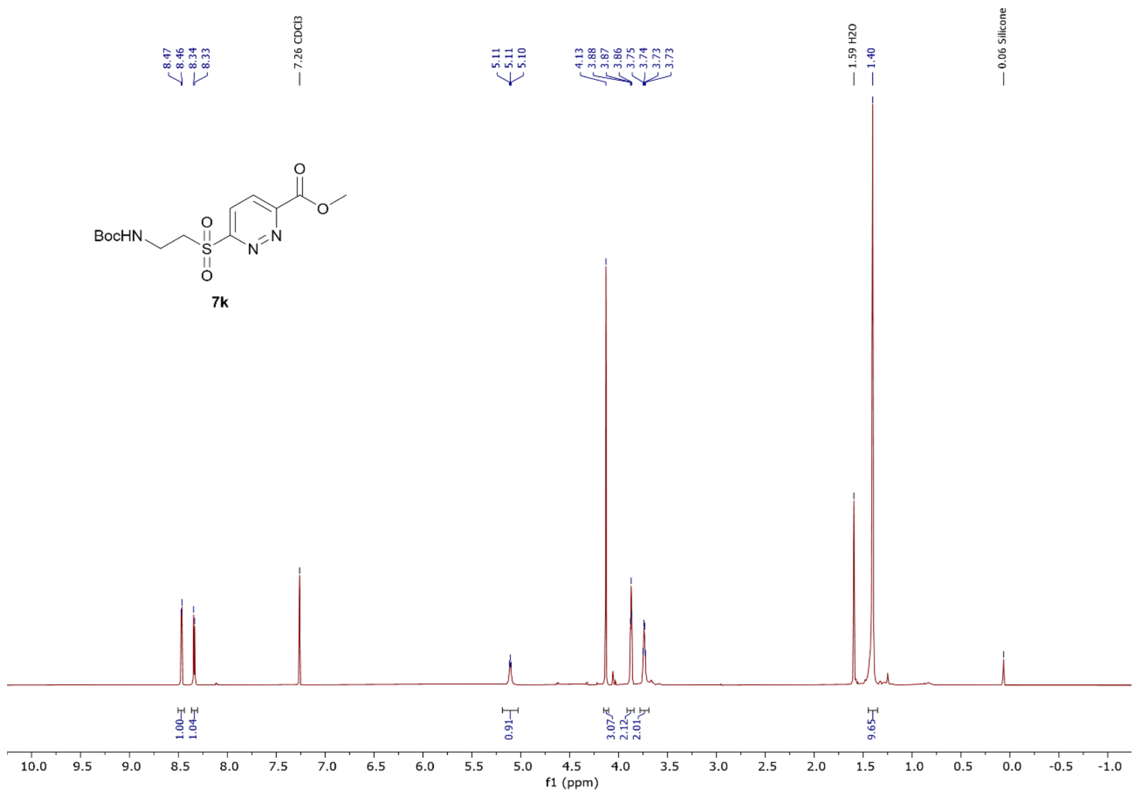

Figure S204. ^{1}H NMR spectrum of compound **7k** (800 MHz, $CDCl_3$).

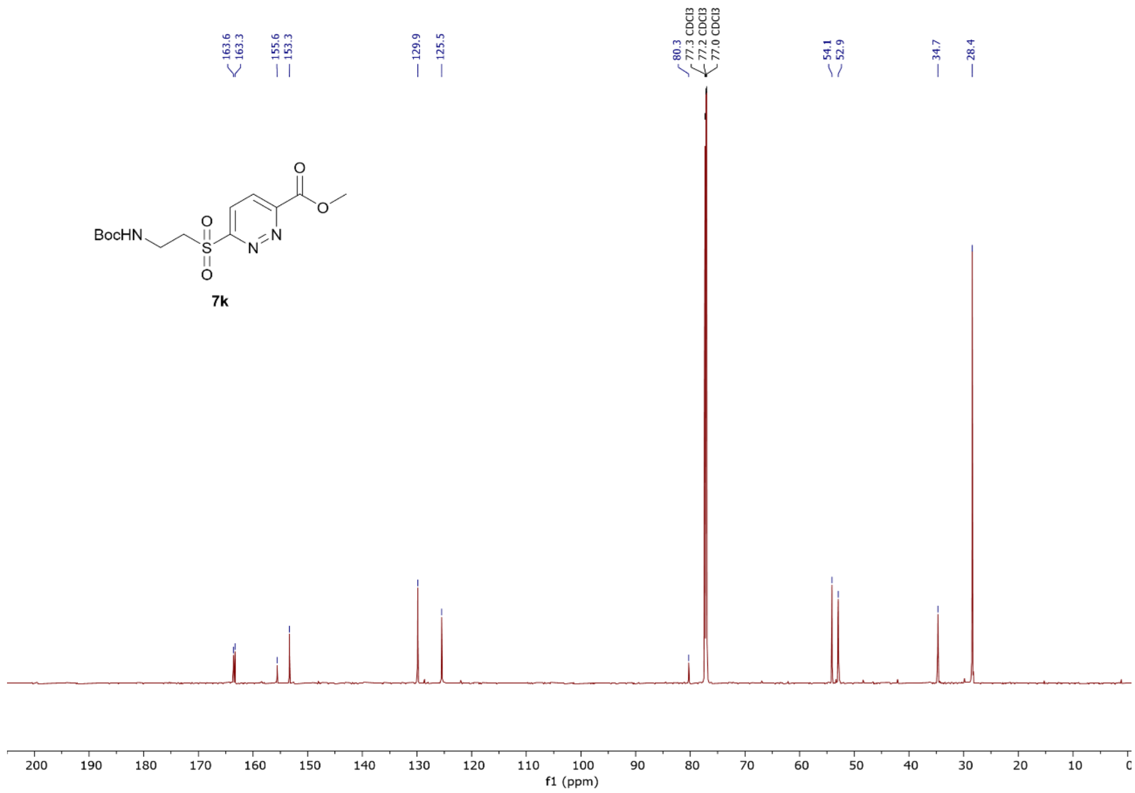

Figure S205. ^{13}C NMR spectrum of compound **7k** (201 MHz, $CDCl_3$).

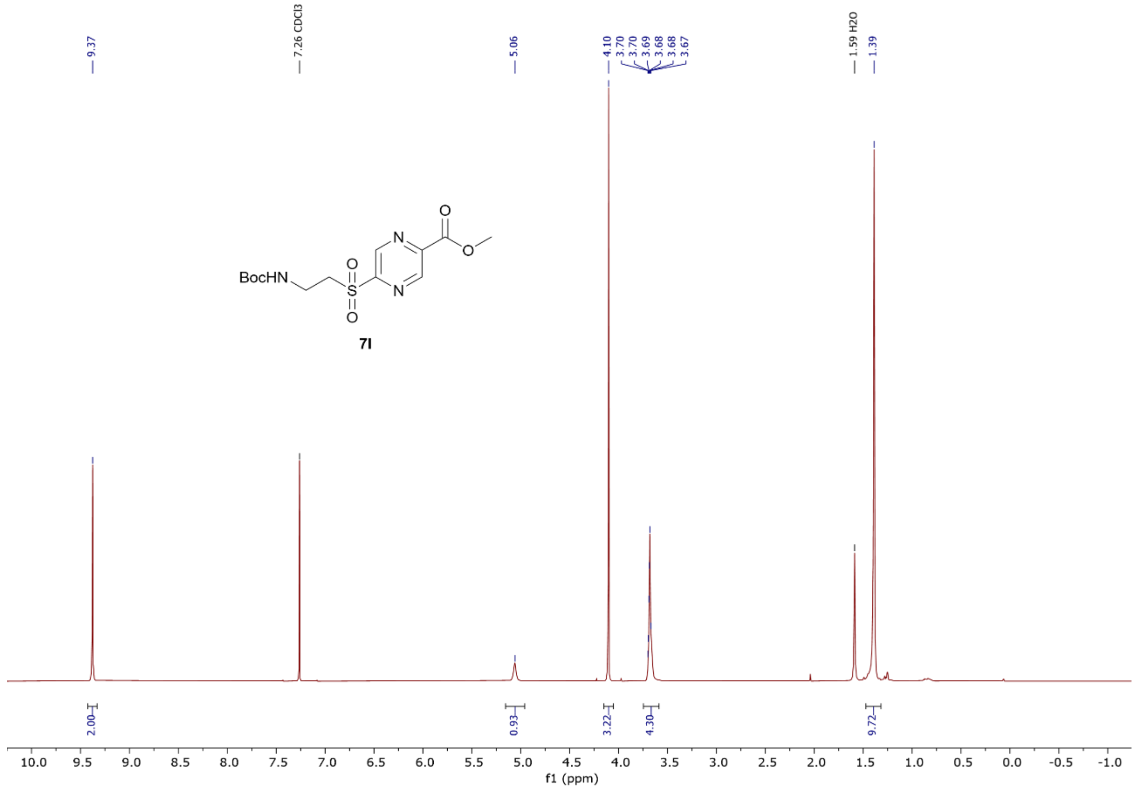

Figure S206. ^{1}H NMR spectrum of compound **7l** (600 MHz, $CDCl_3$).

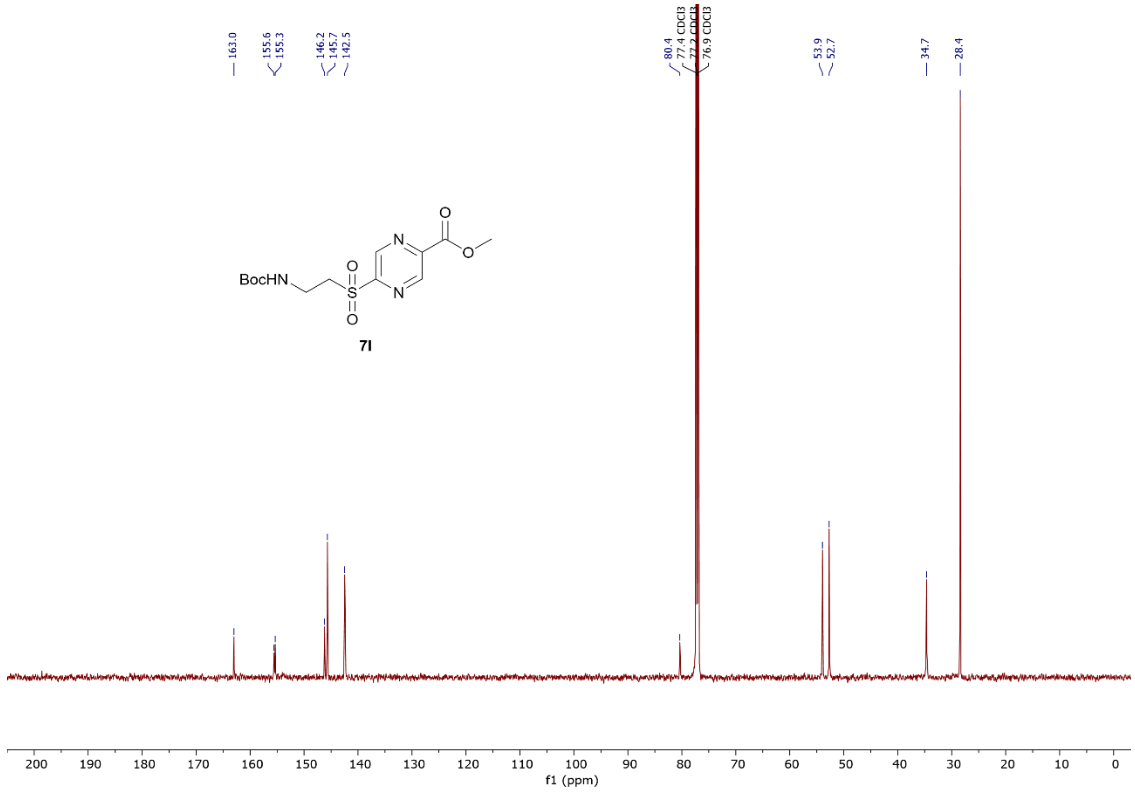

Figure S207. ^{13}C NMR spectrum of compound **7l** (151 MHz, $CDCl_3$).

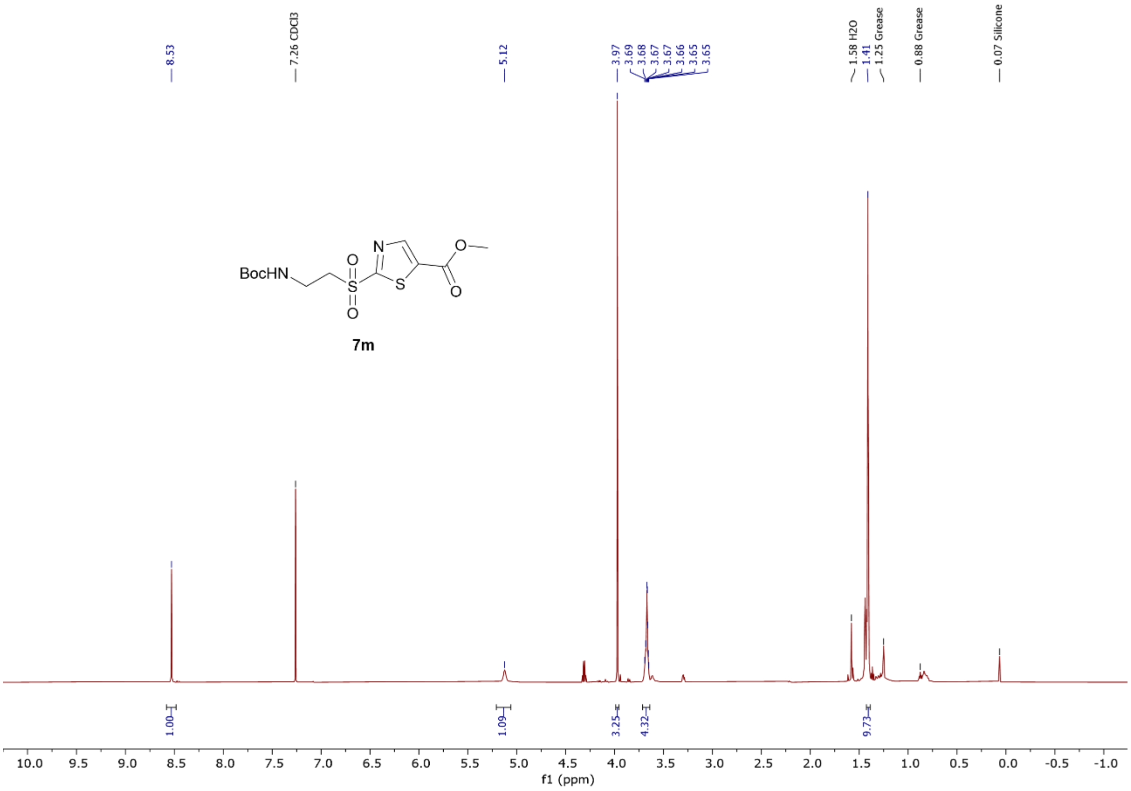

Figure S208. ^{1}H NMR spectrum of compound **7m** (600 MHz, $CDCl_3$).

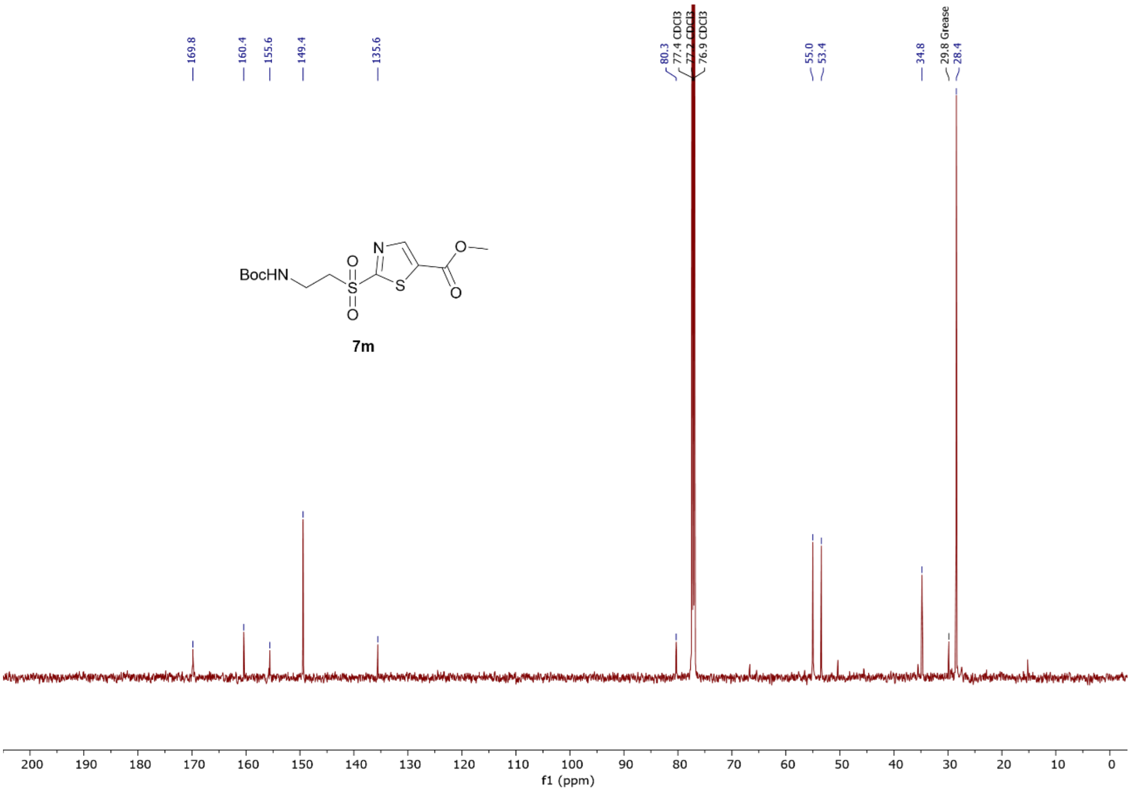

Figure S209. ^{13}C NMR spectrum of compound **7m** (151 MHz, $CDCl_3$).

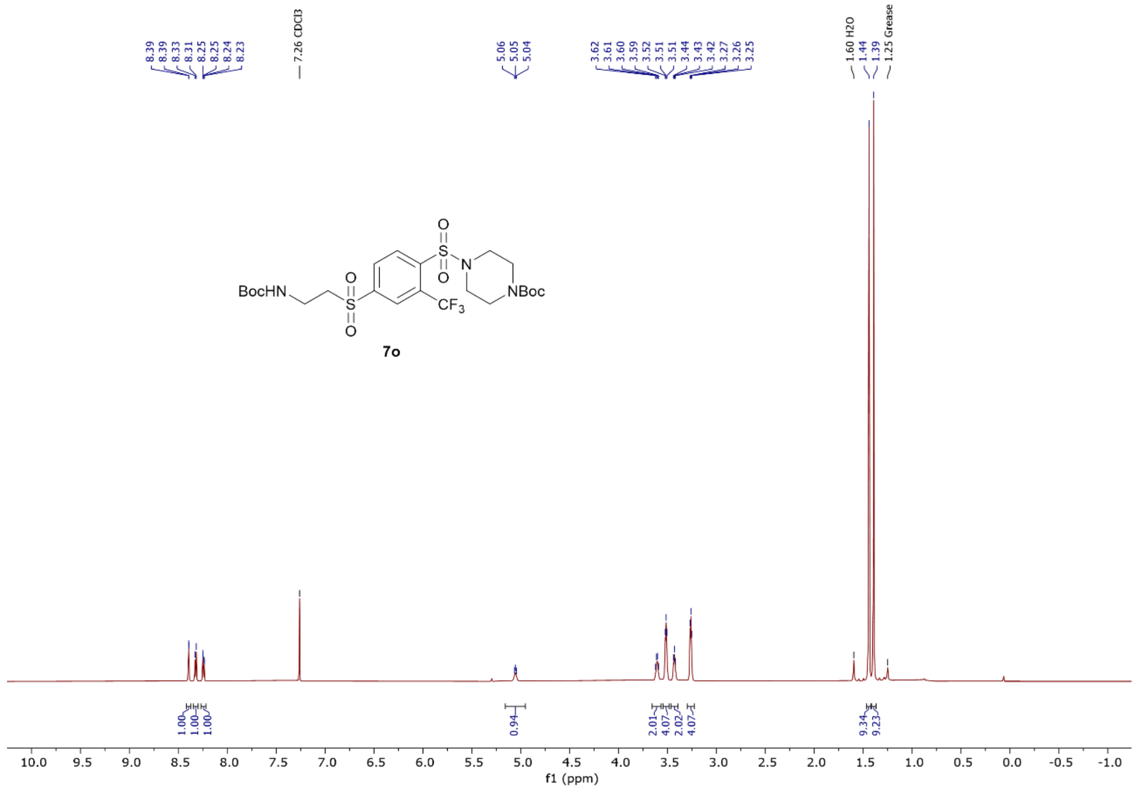

Figure S210. ^{1}H NMR spectrum of compound **7o** (600 MHz, $CDCl_3$).

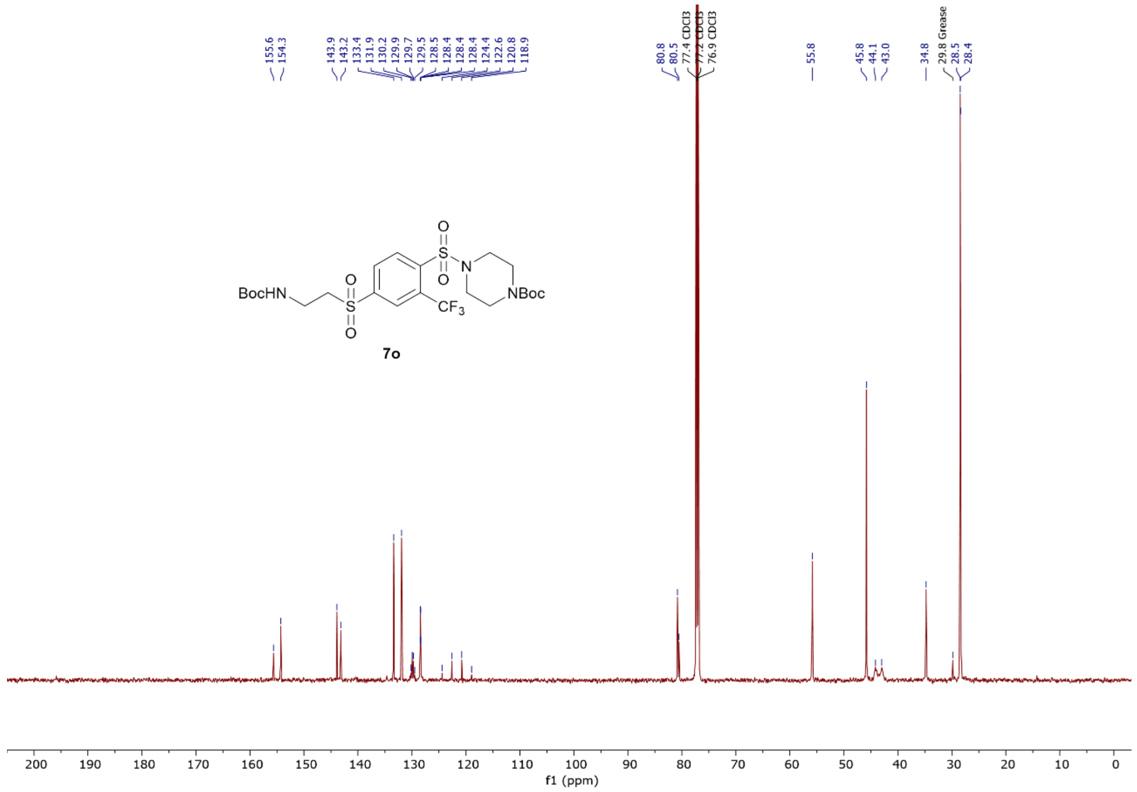

Figure S211. ^{13}C NMR spectrum of compound **7o** (151 MHz, $CDCl_3$).

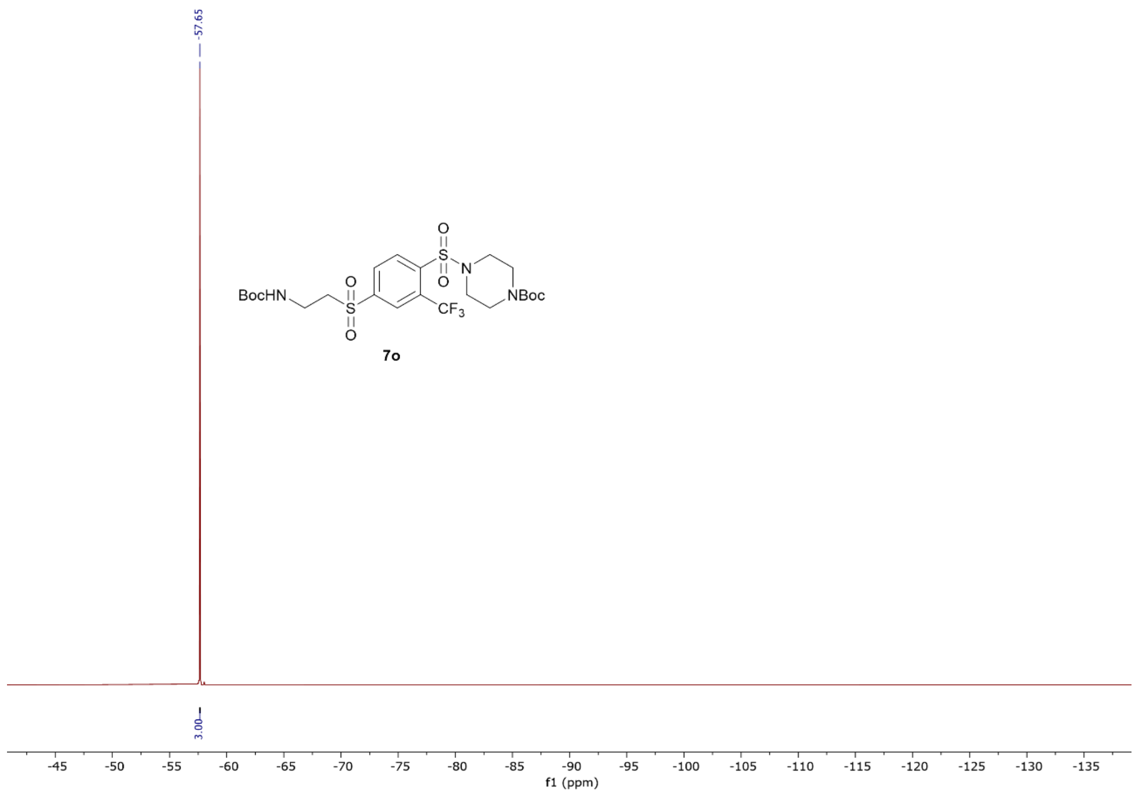

Figure S212. ^{19}F NMR spectrum of compound **7o** (564 MHz, $CDCl_3$).

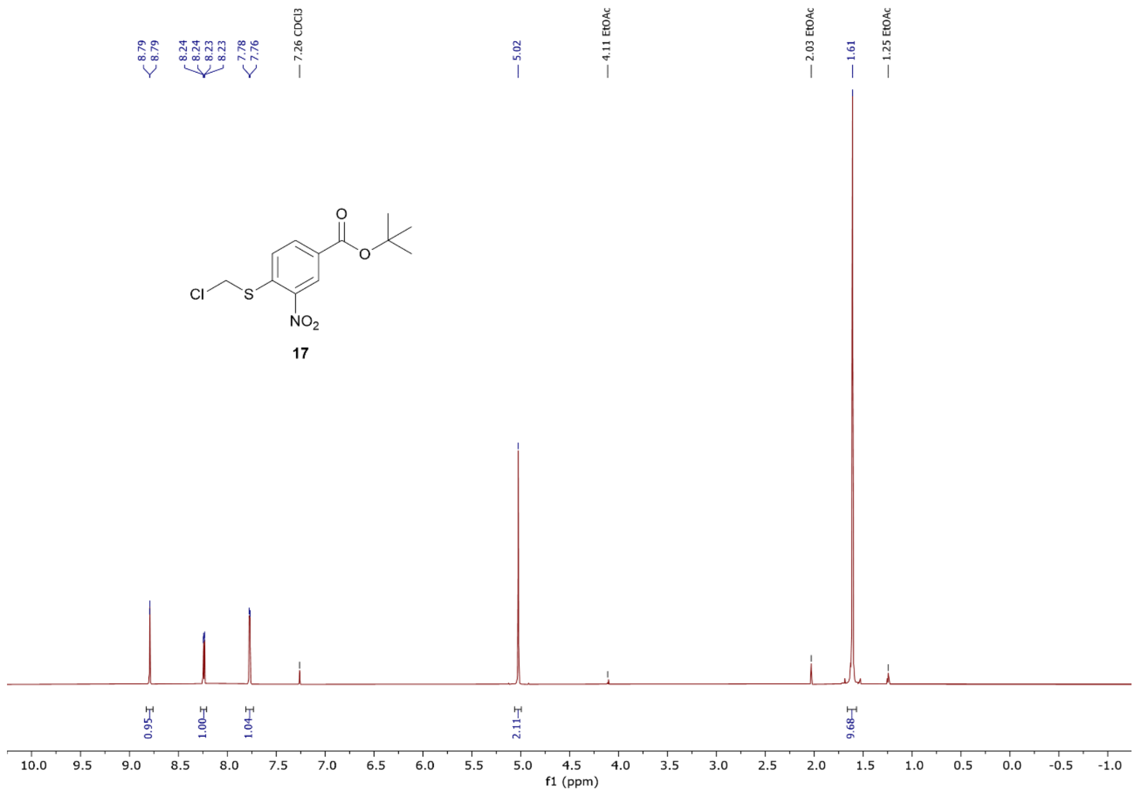

Figure S213. ^{1}H NMR spectrum of compound **17** (800 MHz, $CDCl_3$).

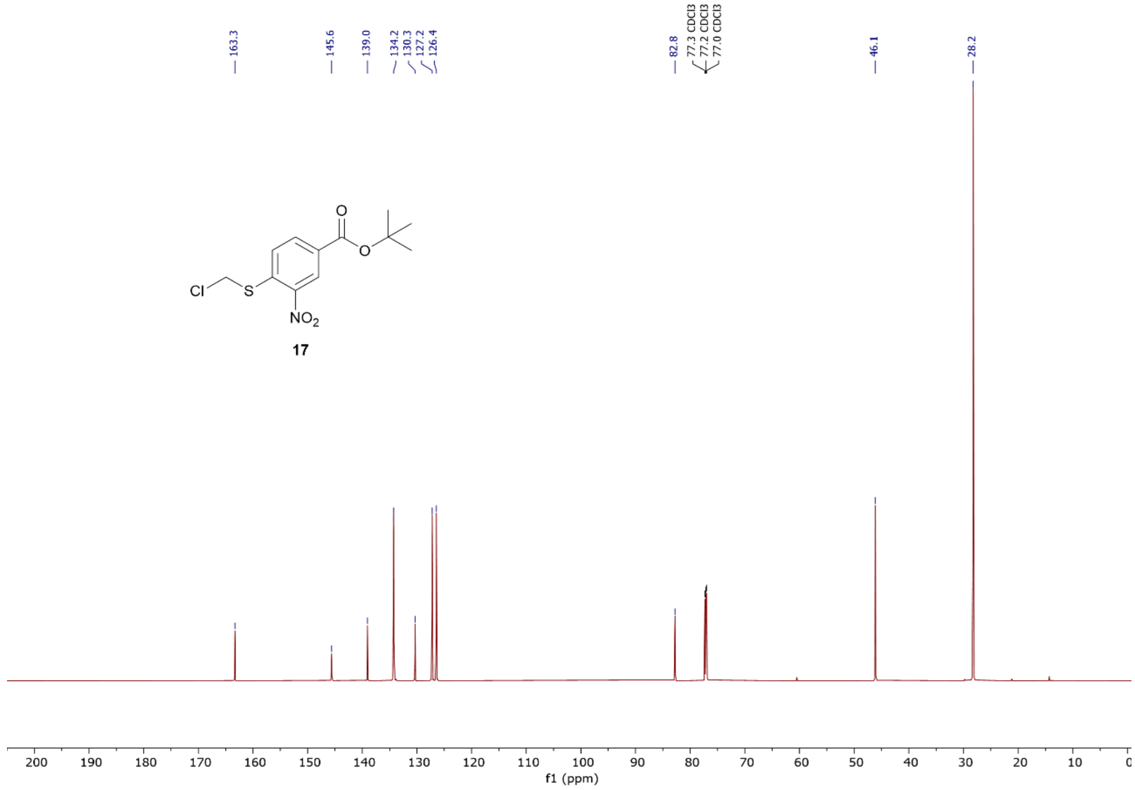

Figure S214. ^{13}C NMR spectrum of compound **17** (201 MHz, $CDCl_3$).

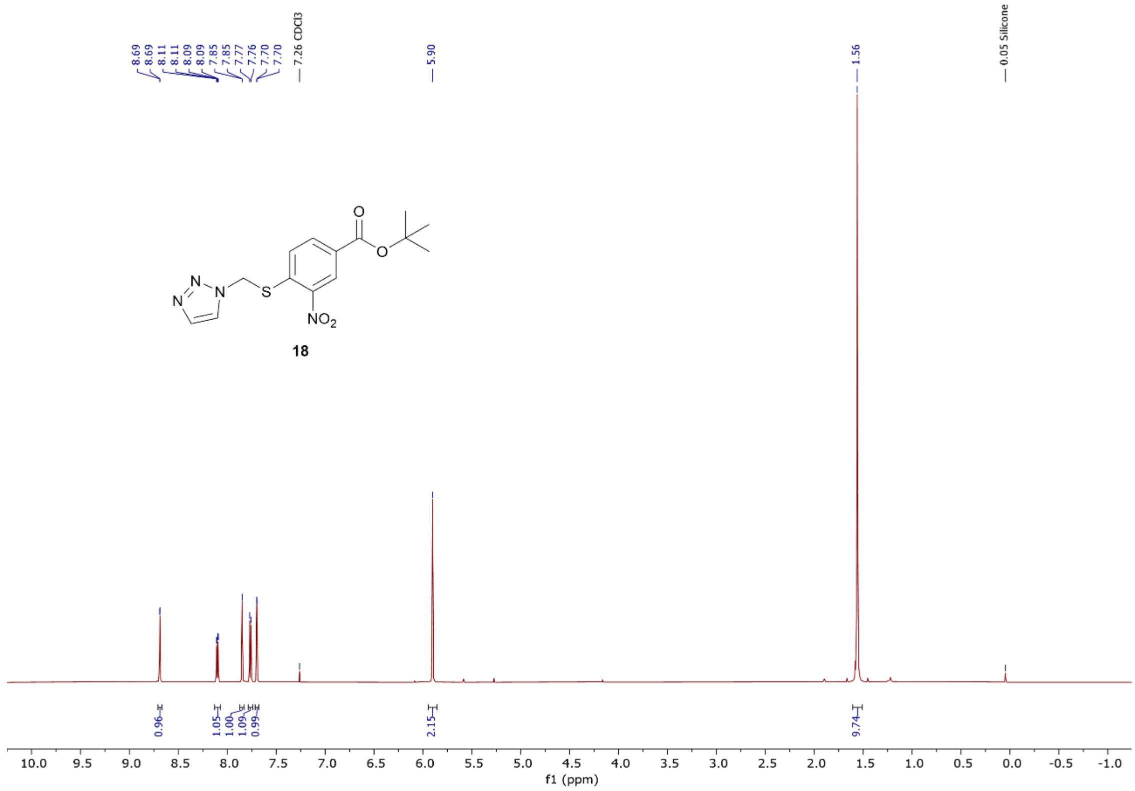

Figure S215. ^{1}H NMR spectrum of compound **18** (600 MHz, $CDCl_3$).

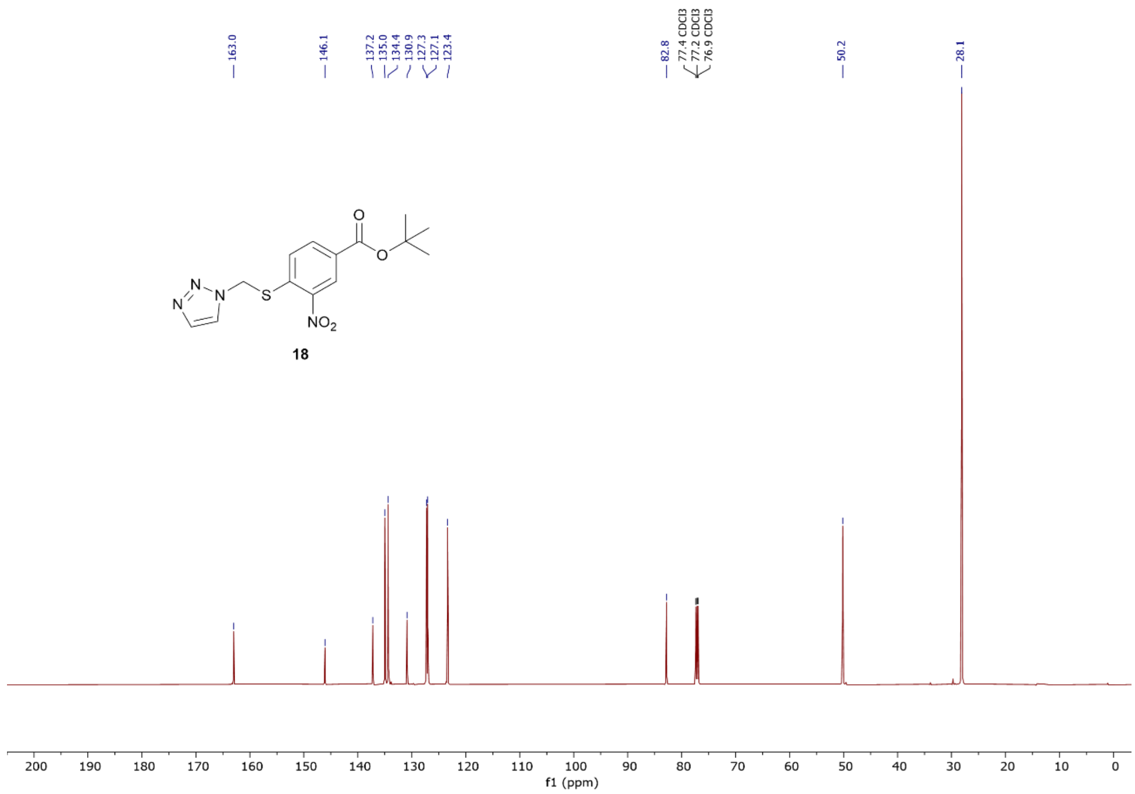

Figure S216. ^{13}C NMR spectrum of compound **18** (151 MHz, $CDCl_3$).

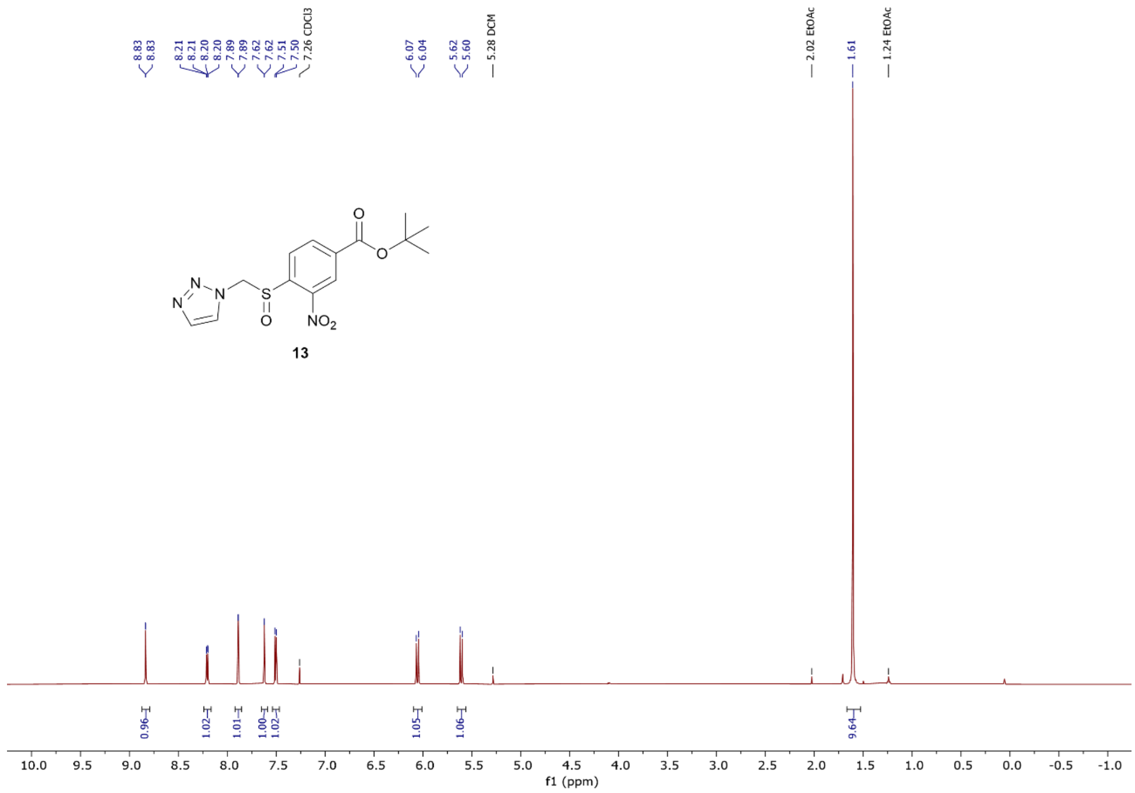

Figure S217. ^{1}H NMR spectrum of compound **13** (600 MHz, $CDCl_3$).

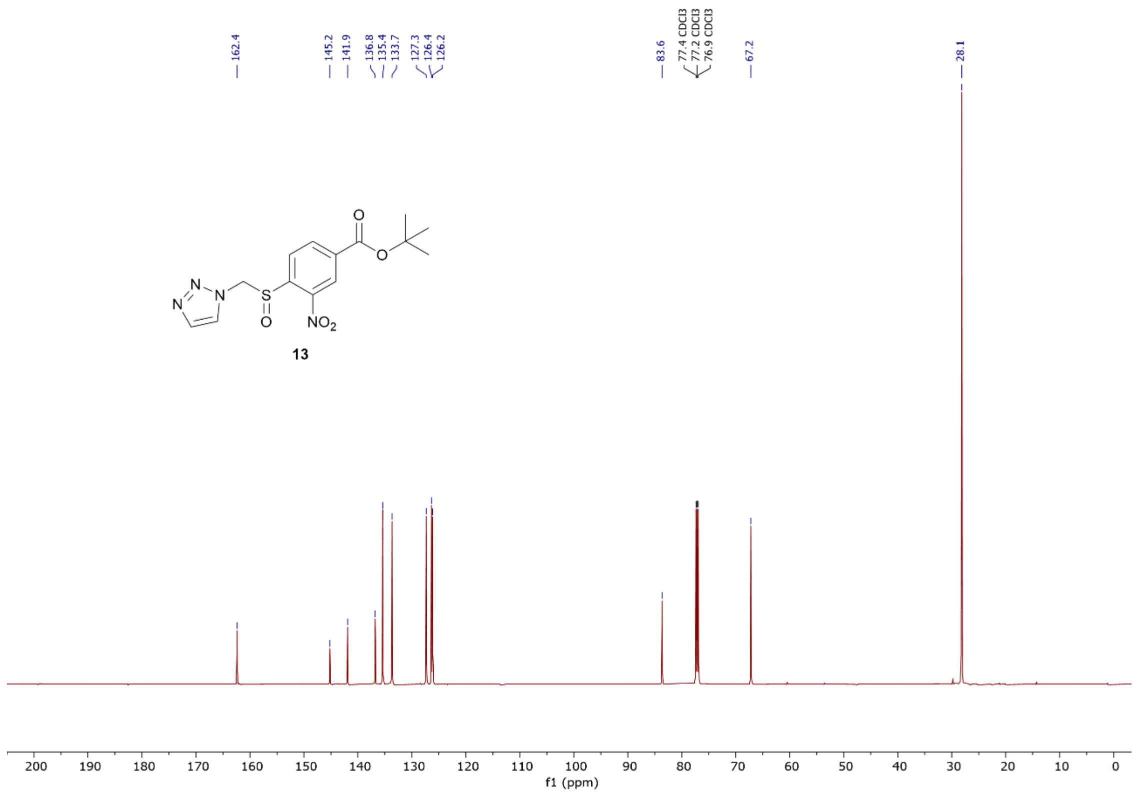

Figure S218. ^{13}C NMR spectrum of compound **13** (151 MHz, $CDCl_3$).

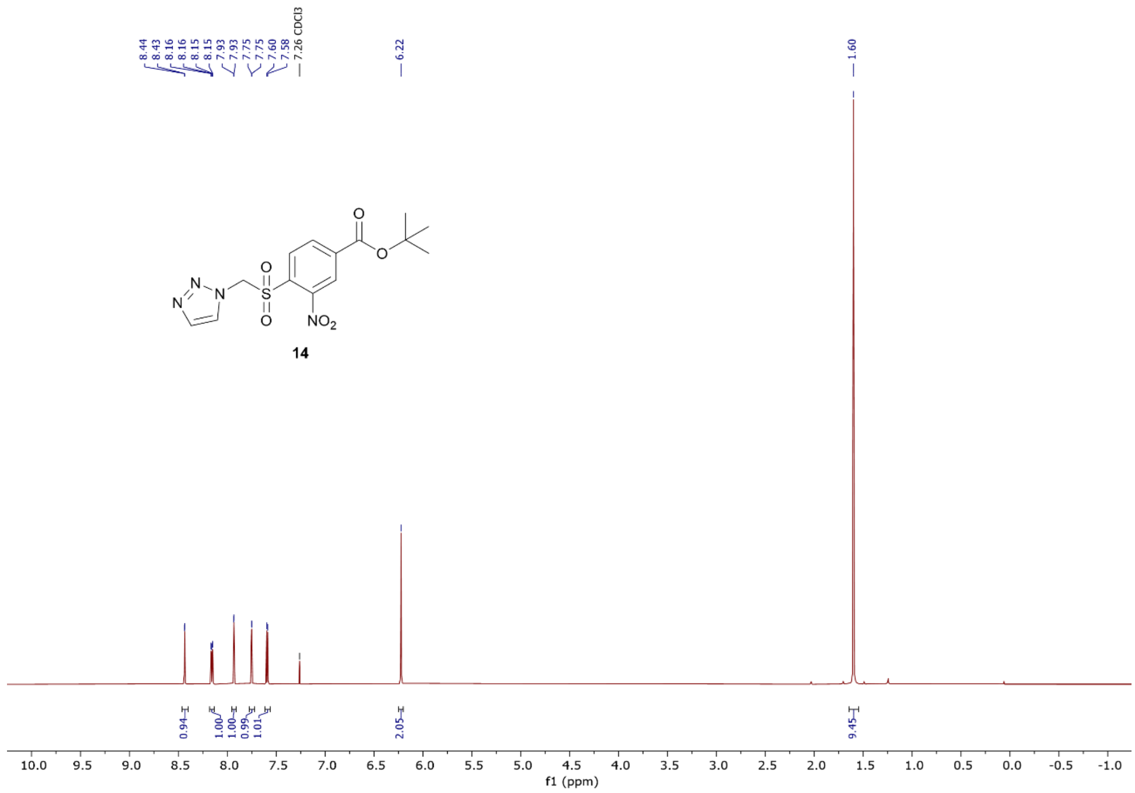

Figure S219. ^{1}H NMR spectrum of compound **14** (600 MHz, $CDCl_3$).

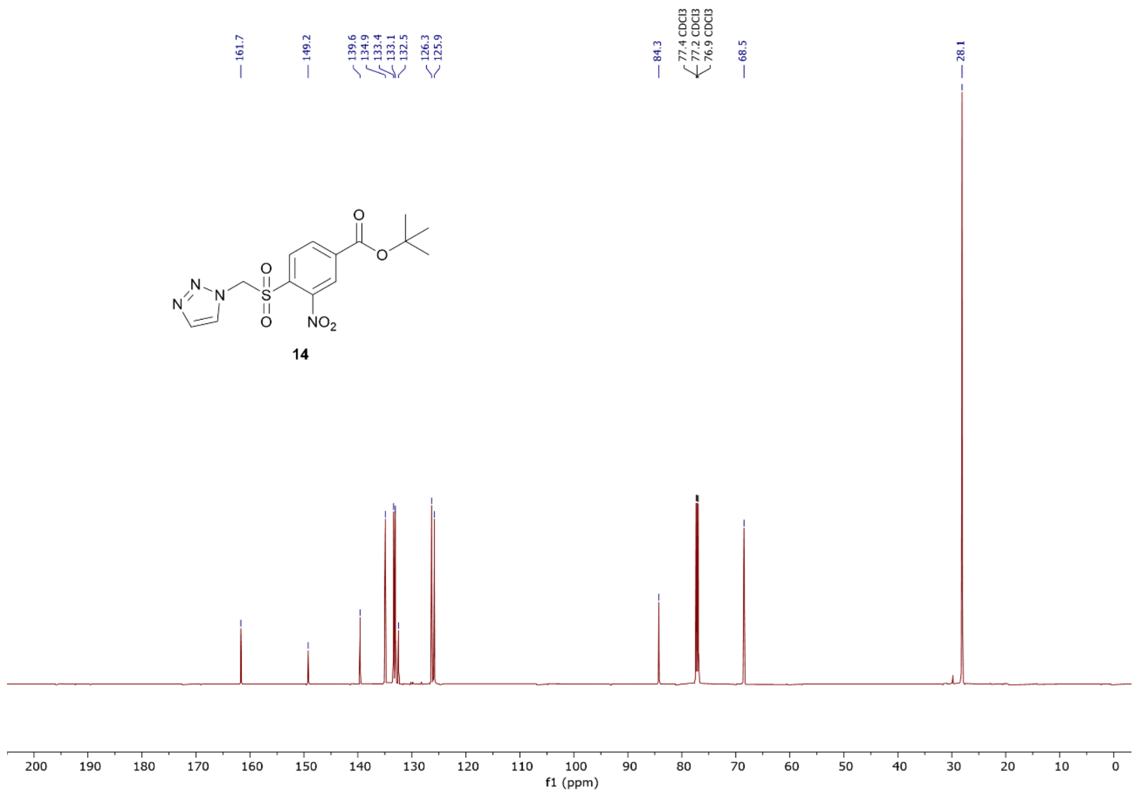

Figure S220. ^{13}C NMR spectrum of compound **14** (151 MHz, $CDCl_3$).

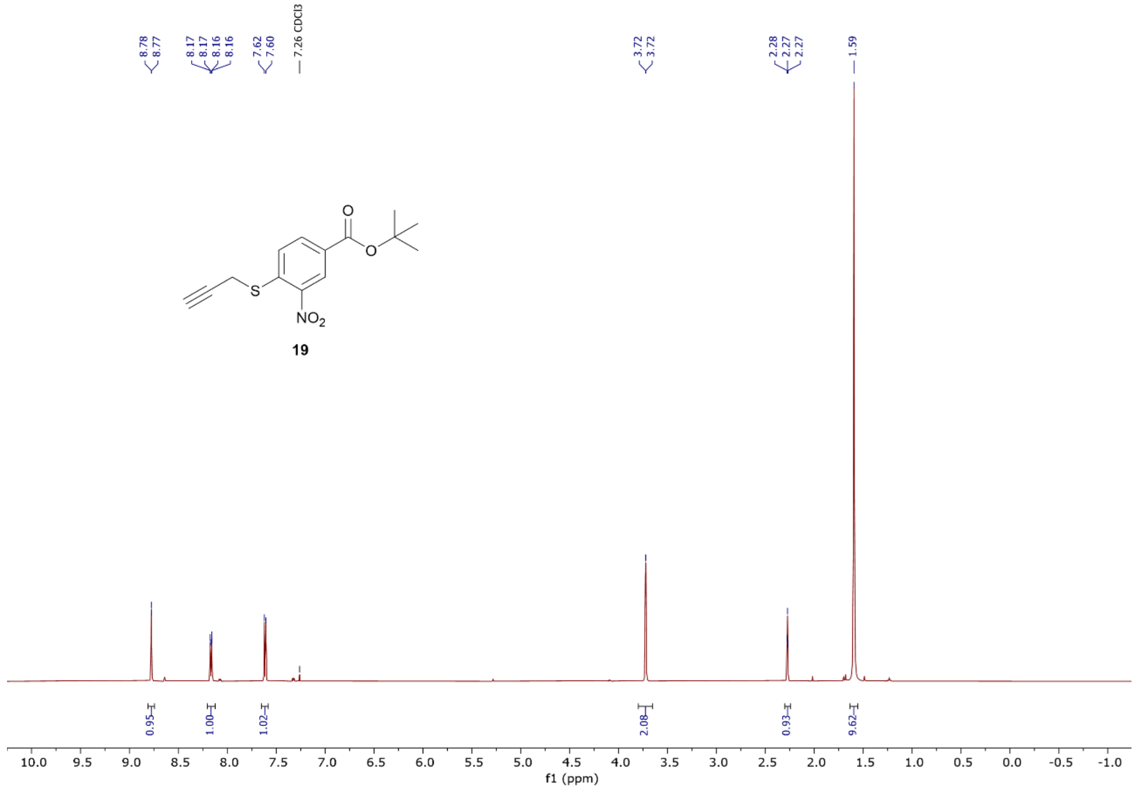

Figure S221. ^{1}H NMR spectrum of compound **19** (600 MHz, $CDCl_3$).

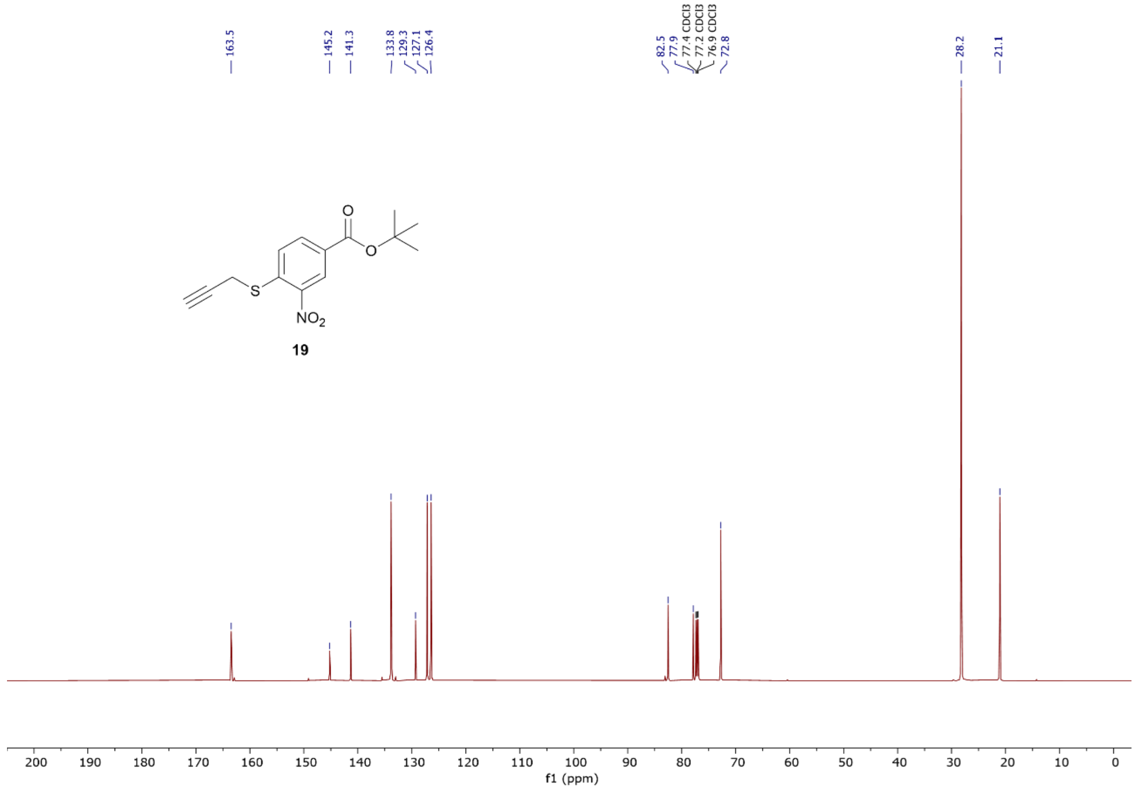

Figure S222. ^{13}C NMR spectrum of compound **19** (151 MHz, $CDCl_3$).

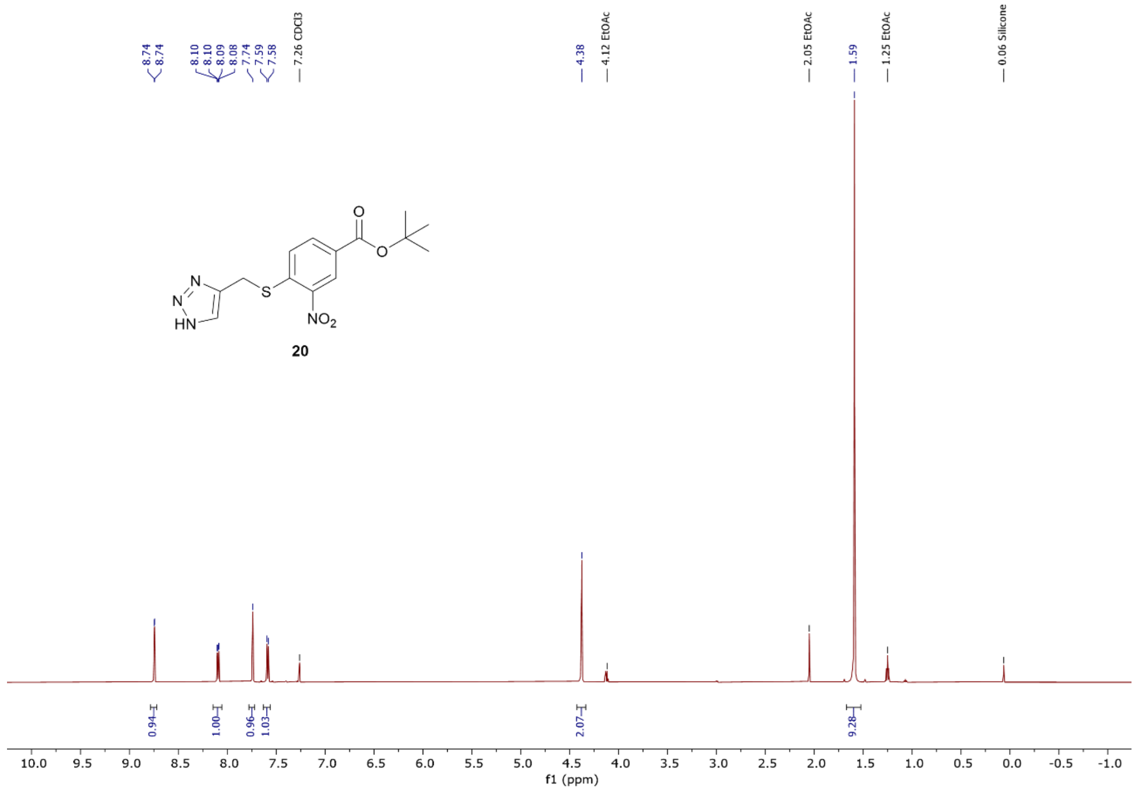

Figure S223. ^{1}H NMR spectrum of compound **20** (600 MHz, $CDCl_3$).

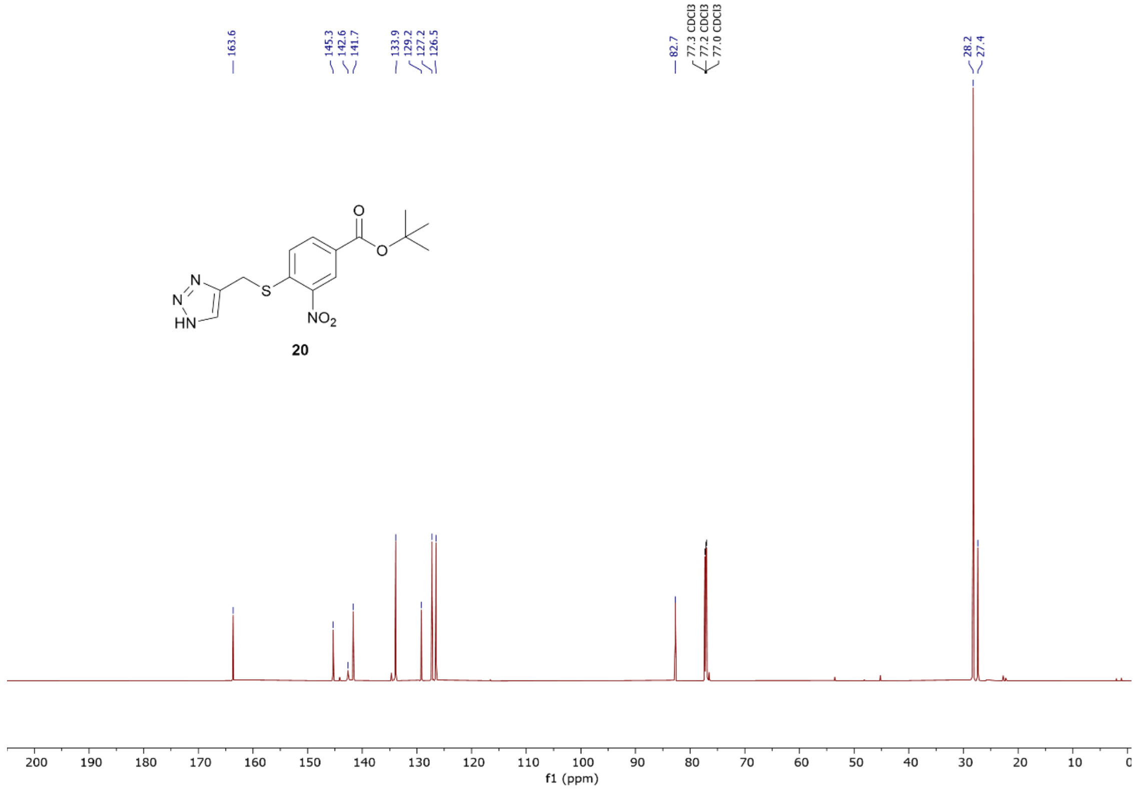

Figure S224. ^{13}C NMR spectrum of compound **20** (201 MHz, $CDCl_3$).

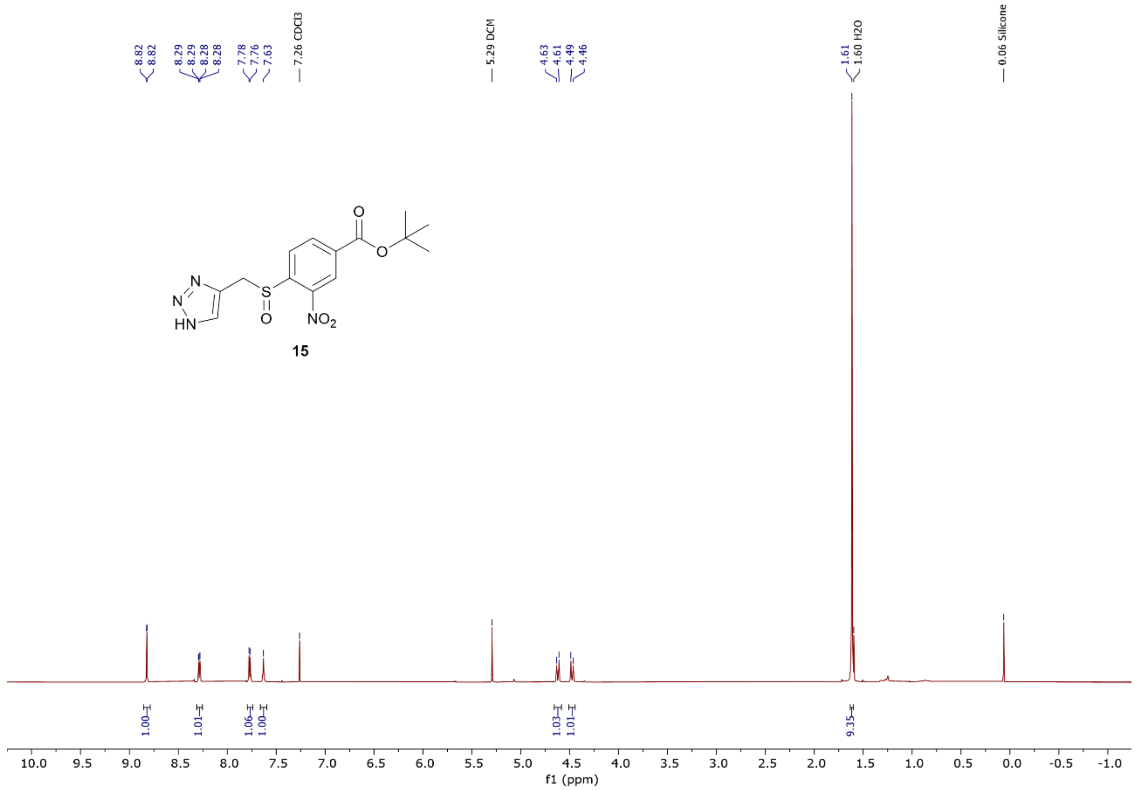

Figure S225. ^{1}H NMR spectrum of compound **15** (600 MHz, $CDCl_3$).

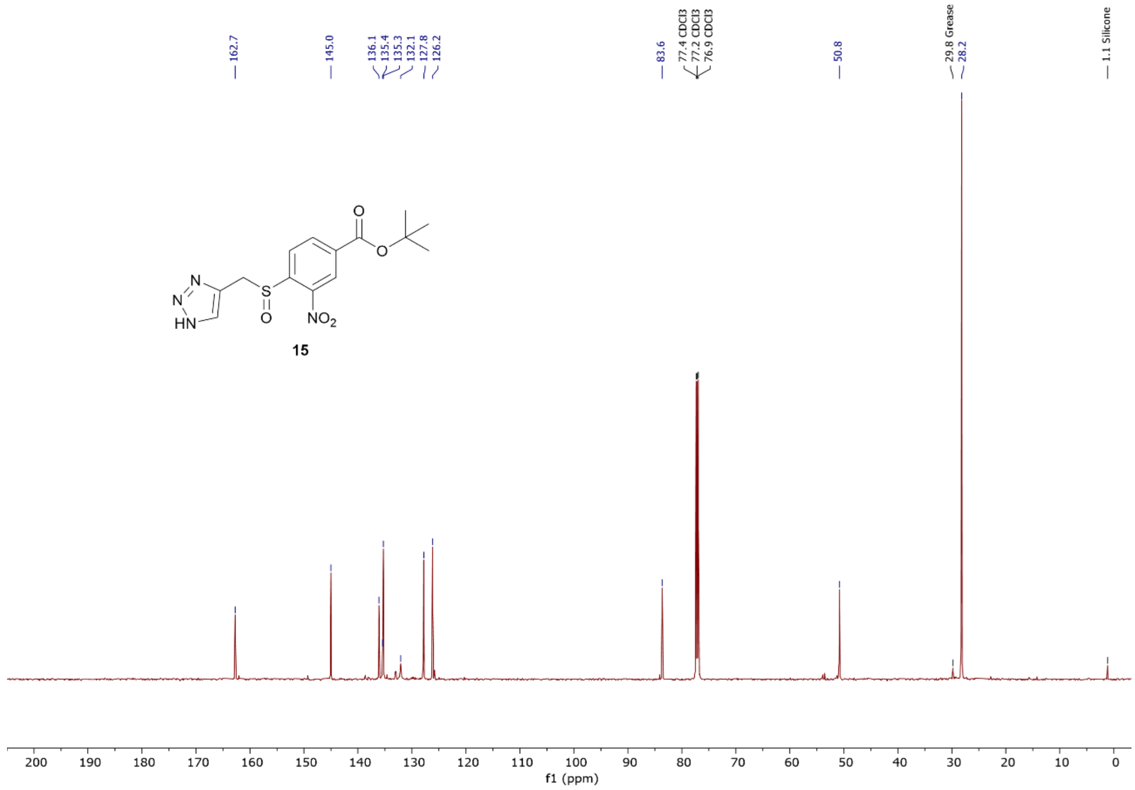

Figure S226. ^{13}C NMR spectrum of compound **15** (151 MHz, $CDCl_3$).

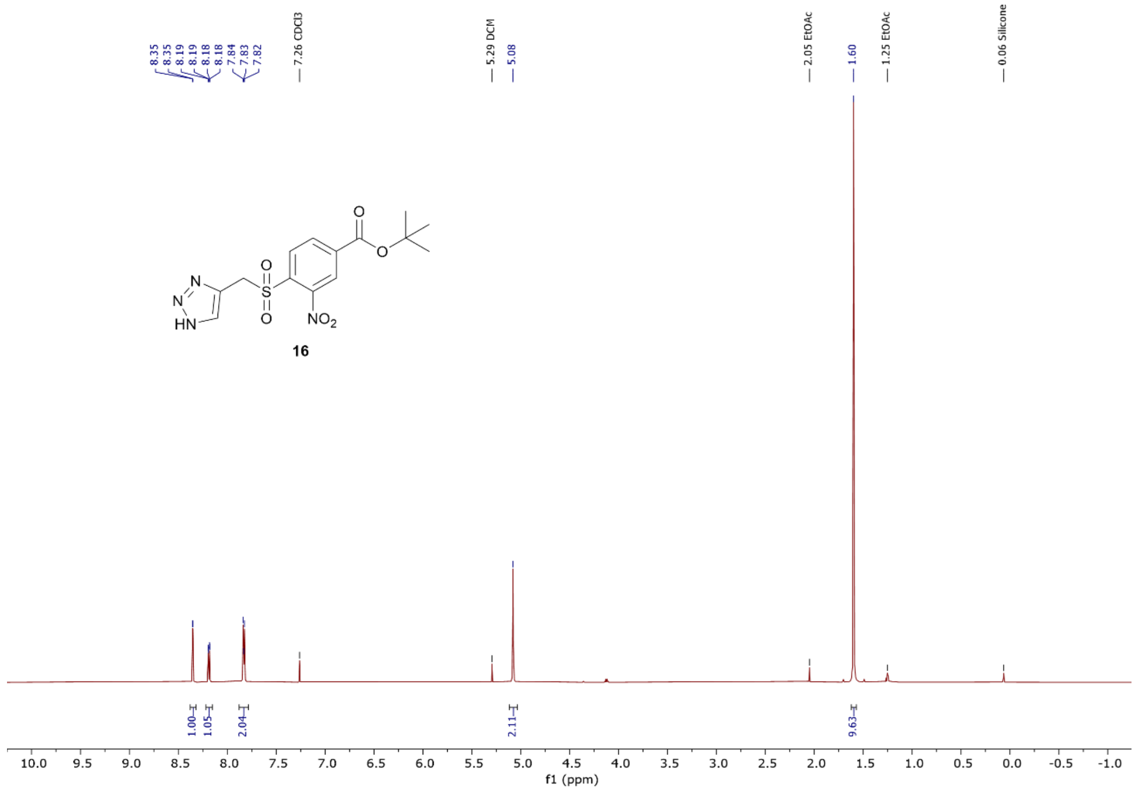

Figure S227. ^{1}H NMR spectrum of compound **16** (600 MHz, $CDCl_3$).

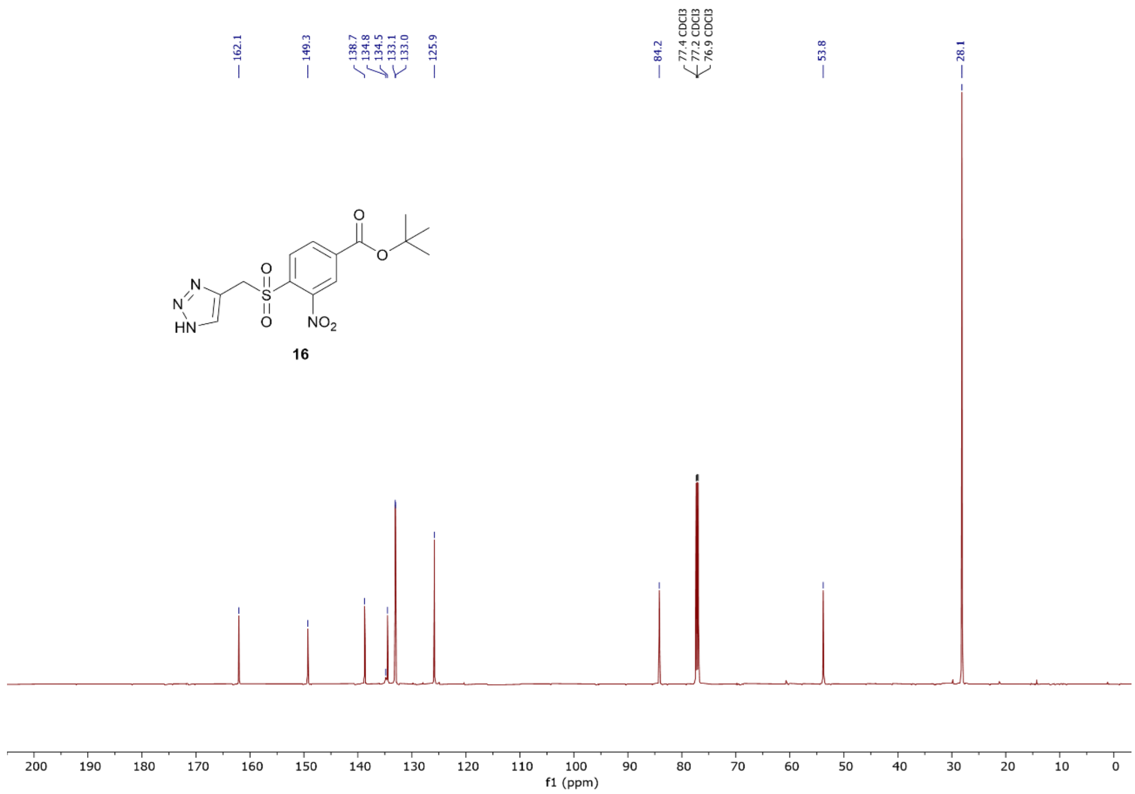

Figure S228. ^{13}C NMR spectrum of compound **16** (151 MHz, $CDCl_3$).

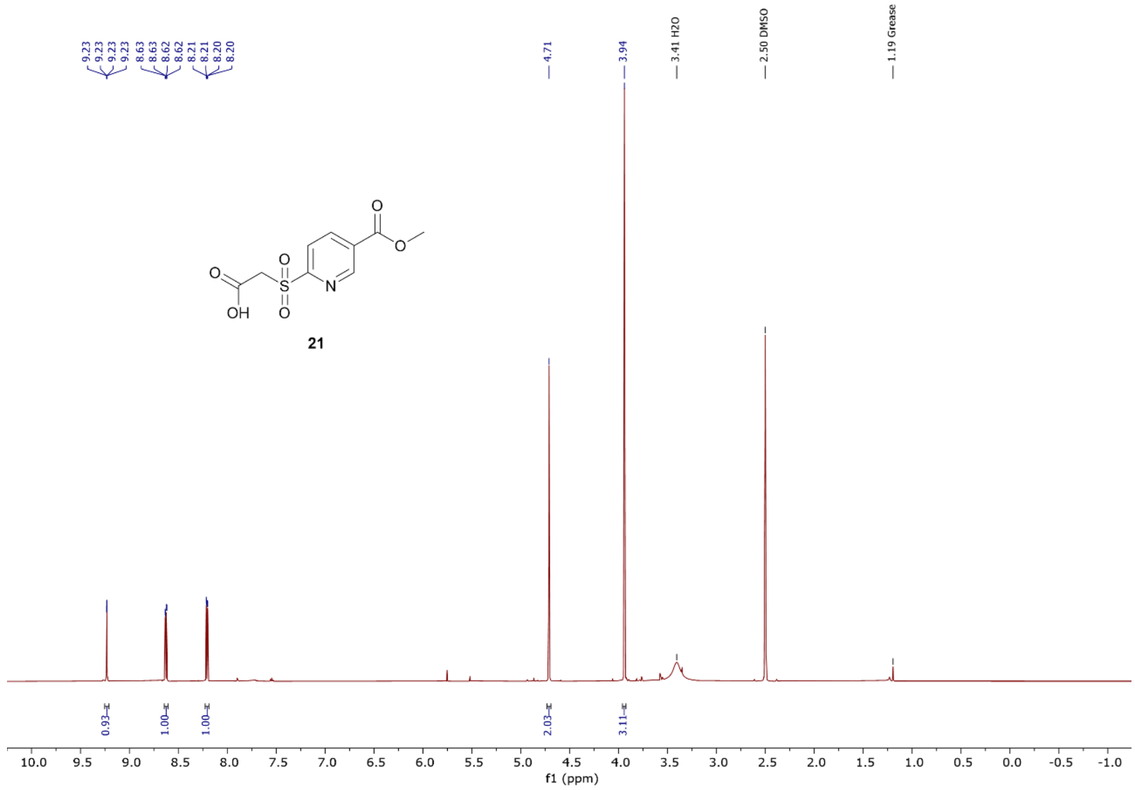

Figure S229. ^{1}H NMR spectrum of compound **21** (600 MHz, $(CD_3)_2SO$).

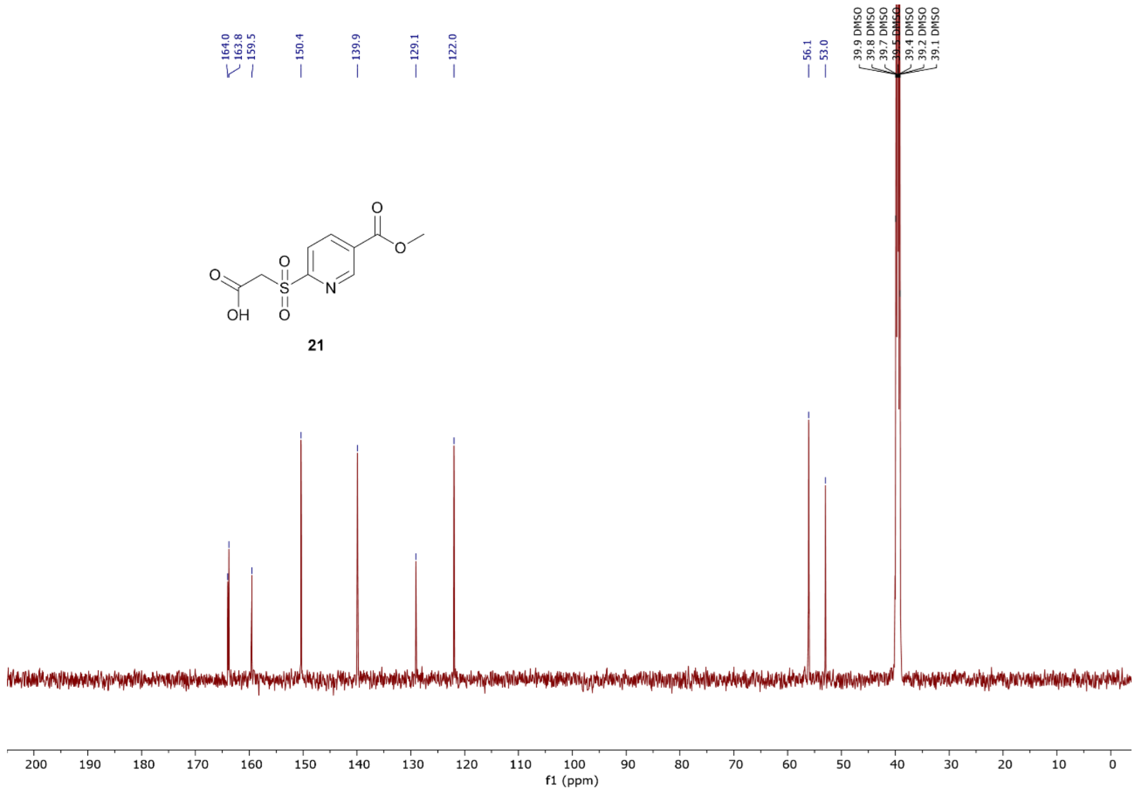

Figure S230. ^{13}C NMR spectrum of compound **21** (151 MHz, $(CD_3)_2SO$).

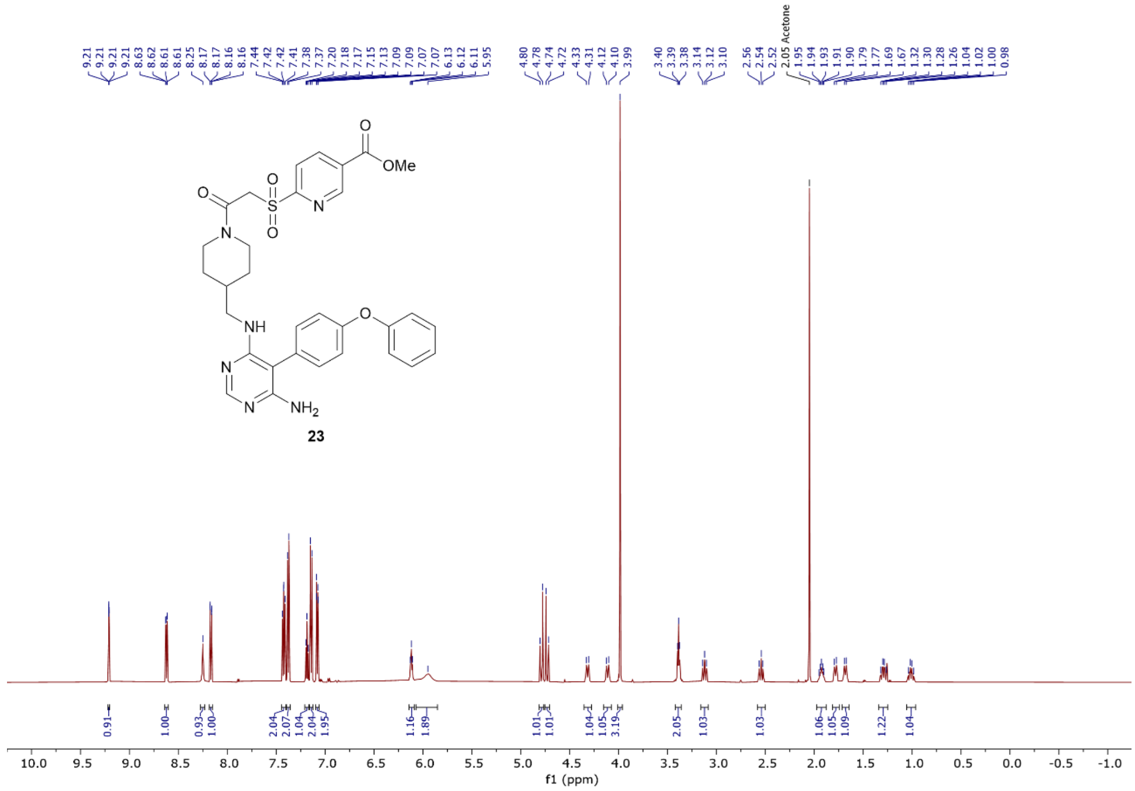

Figure S231. ^{1}H NMR spectrum of compound **23** (600 MHz, $(CD_3)_2CO$).

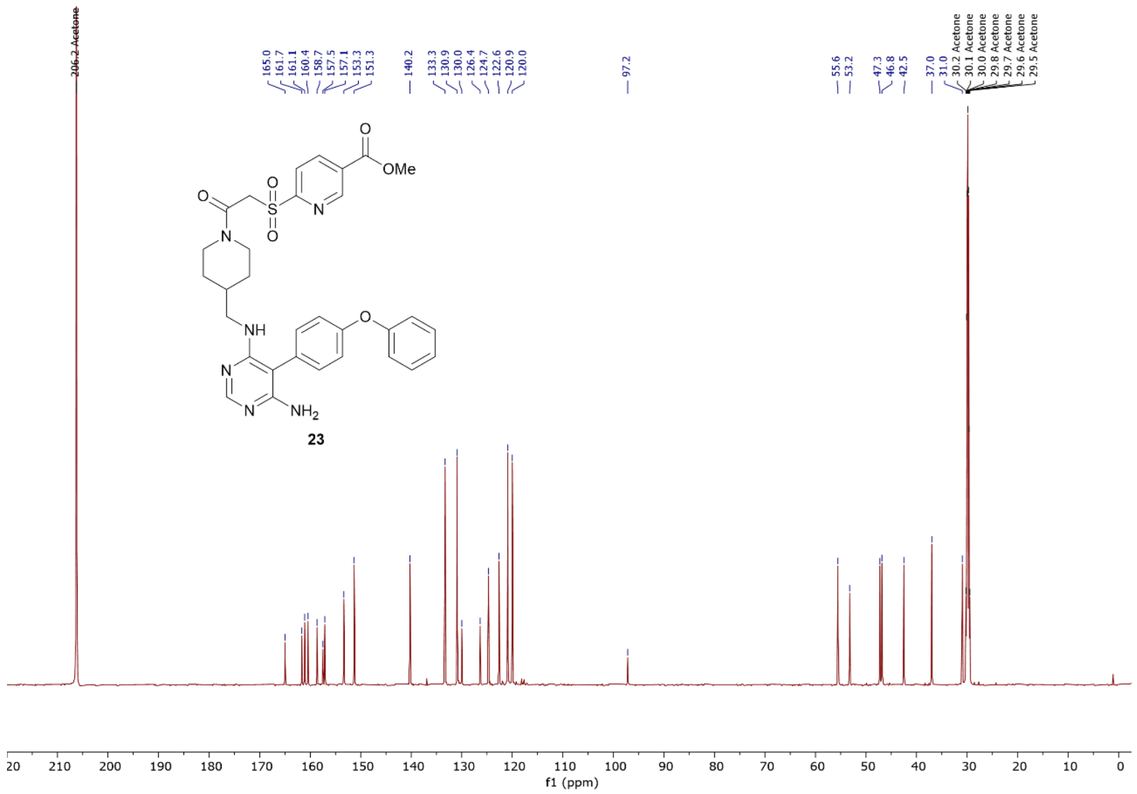

Figure S232. ^{13}C NMR spectrum of compound **23** (151 MHz, $(CD_3)_2CO$).

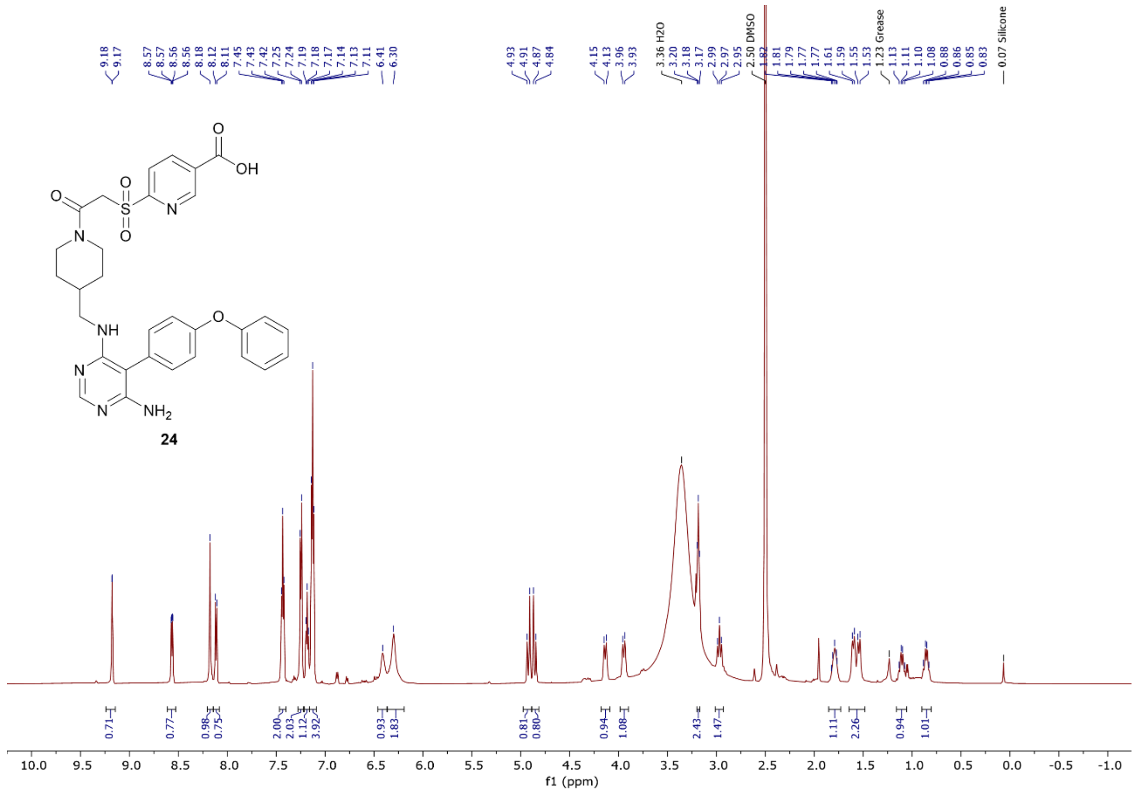

Figure S233. ^{1}H NMR spectrum of compound **24** (600 MHz, $(CD_3)_2SO$).

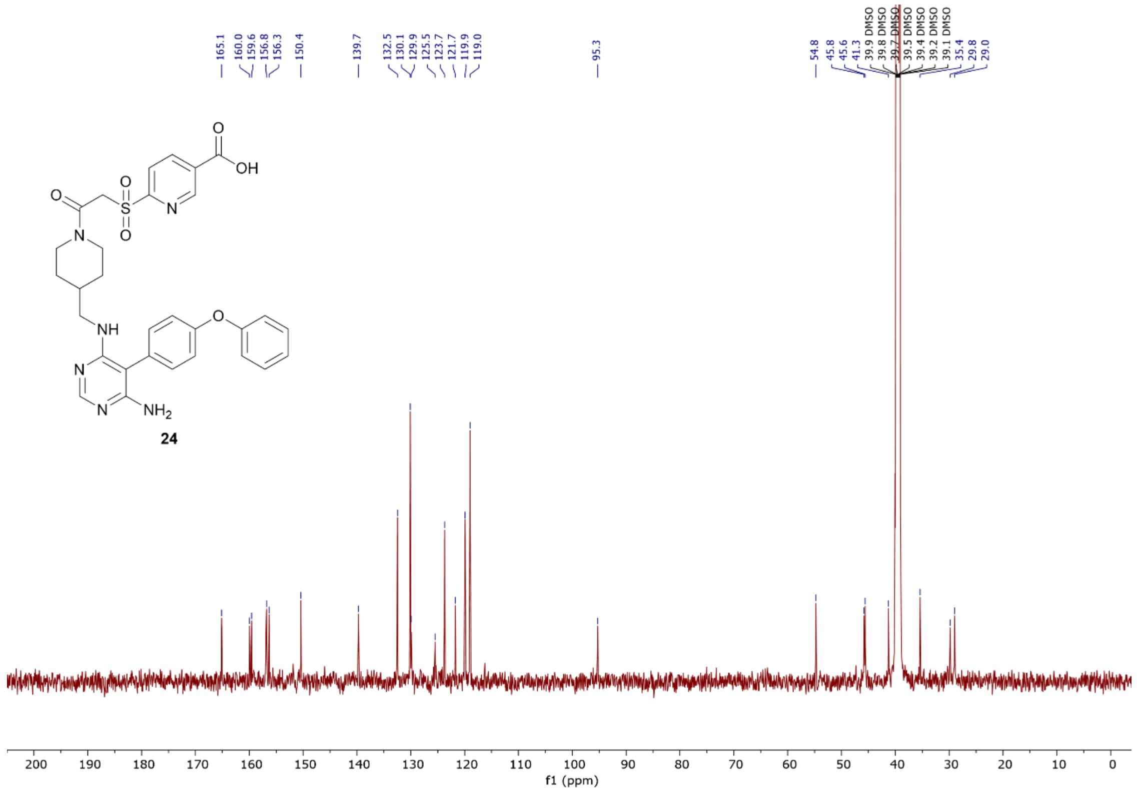

Figure S234. ^{13}C NMR spectrum of compound **24** (151 MHz, $(CD_3)_2SO$).

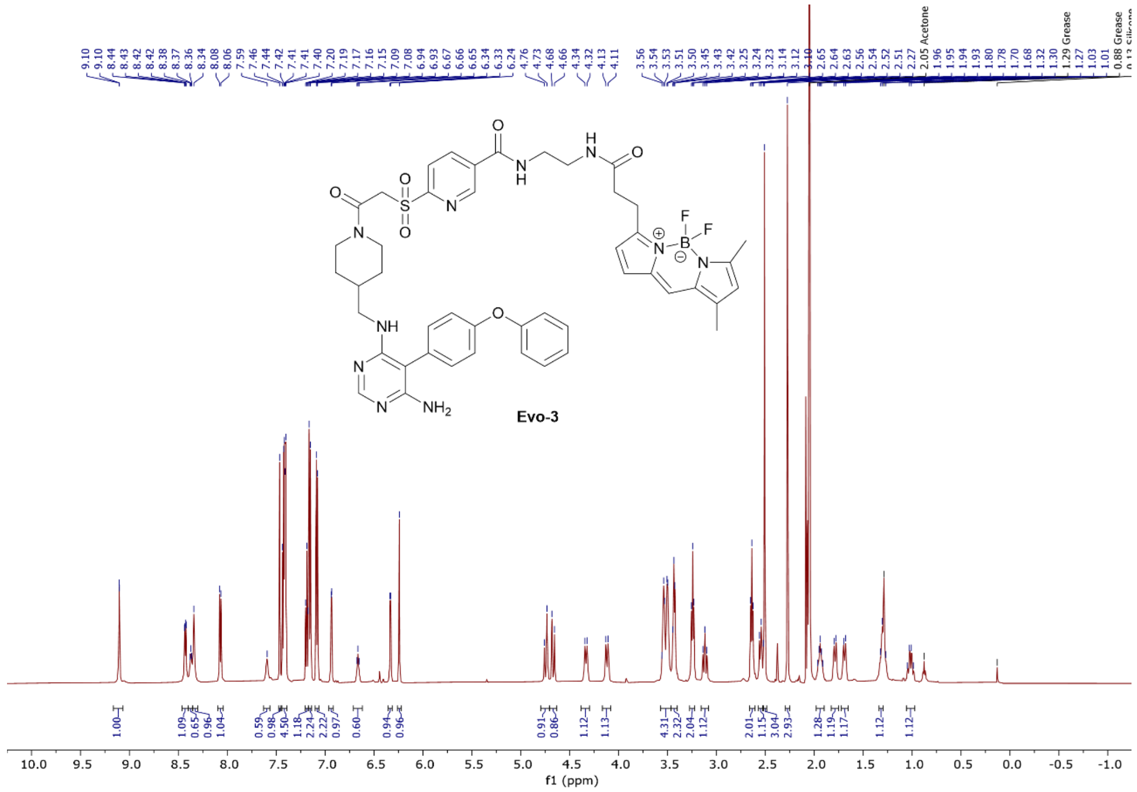

Figure S235. ^{1}H NMR spectrum of **Evo-3** (600 MHz, $(CD_3)_2CO$).

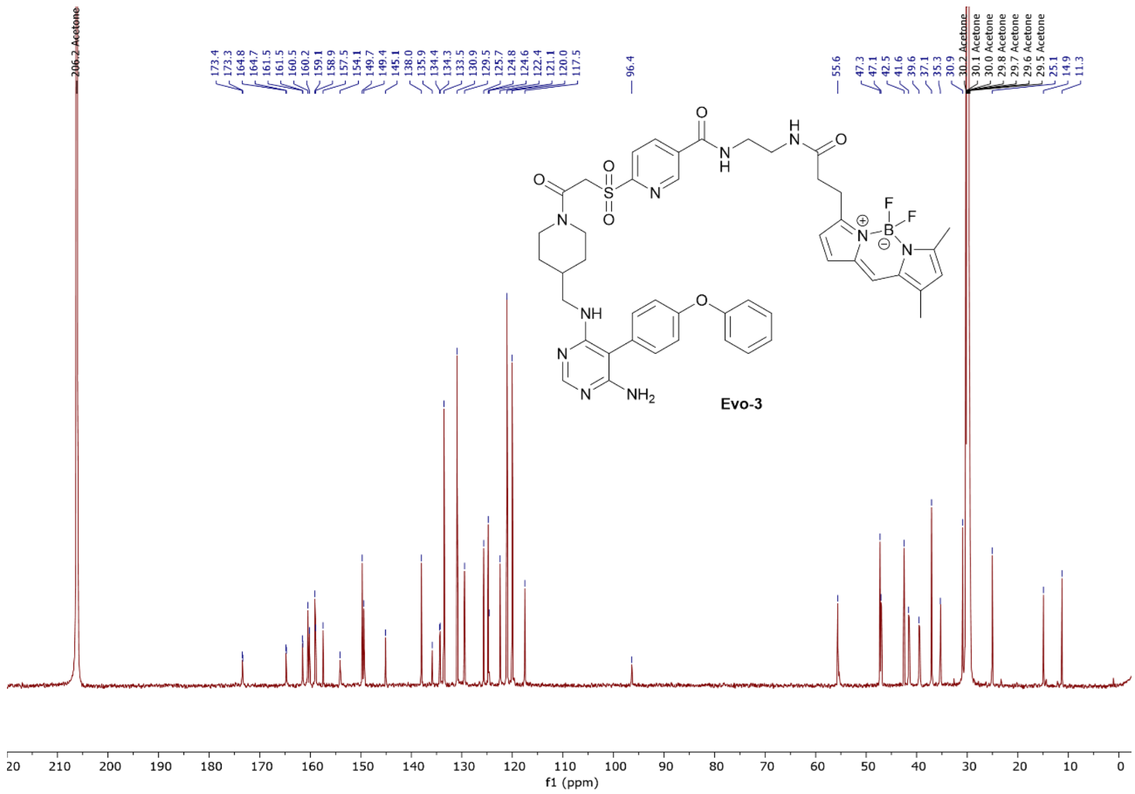

Figure S236. ^{13}C NMR spectrum of **Evo-3** (151 MHz, $(CD_3)_2CO$).

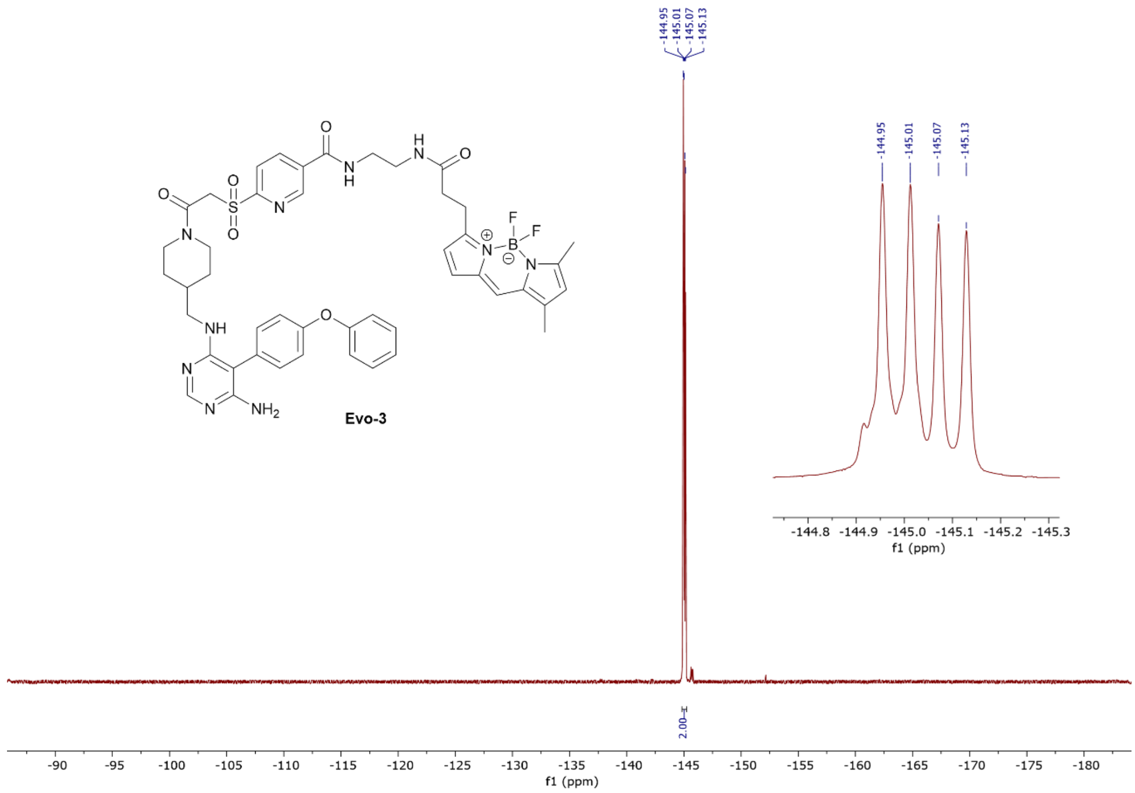

Figure S237. ^{19}F NMR spectrum of **Evo-3** (564 MHz, $(CD_3)_2CO$).

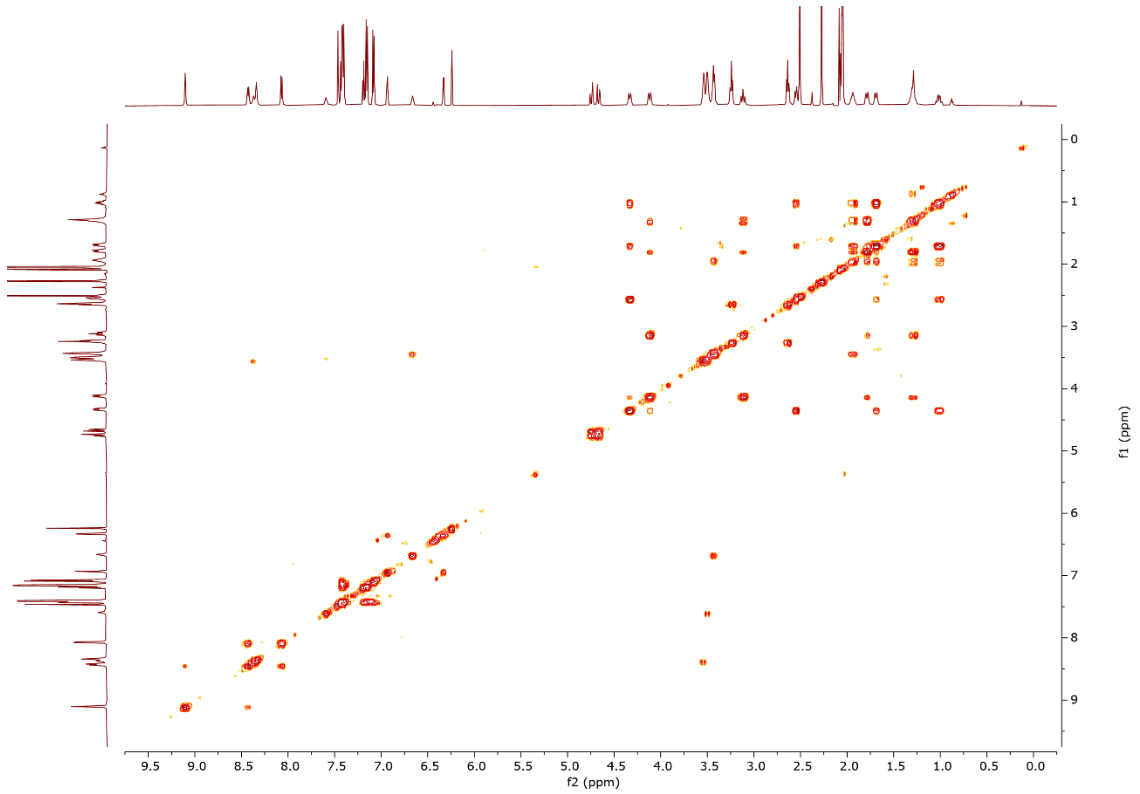

Figure S238. ^{1}H-^{1}H COSY spectrum of **Evo-3** [$(CD_3)_2CO$].

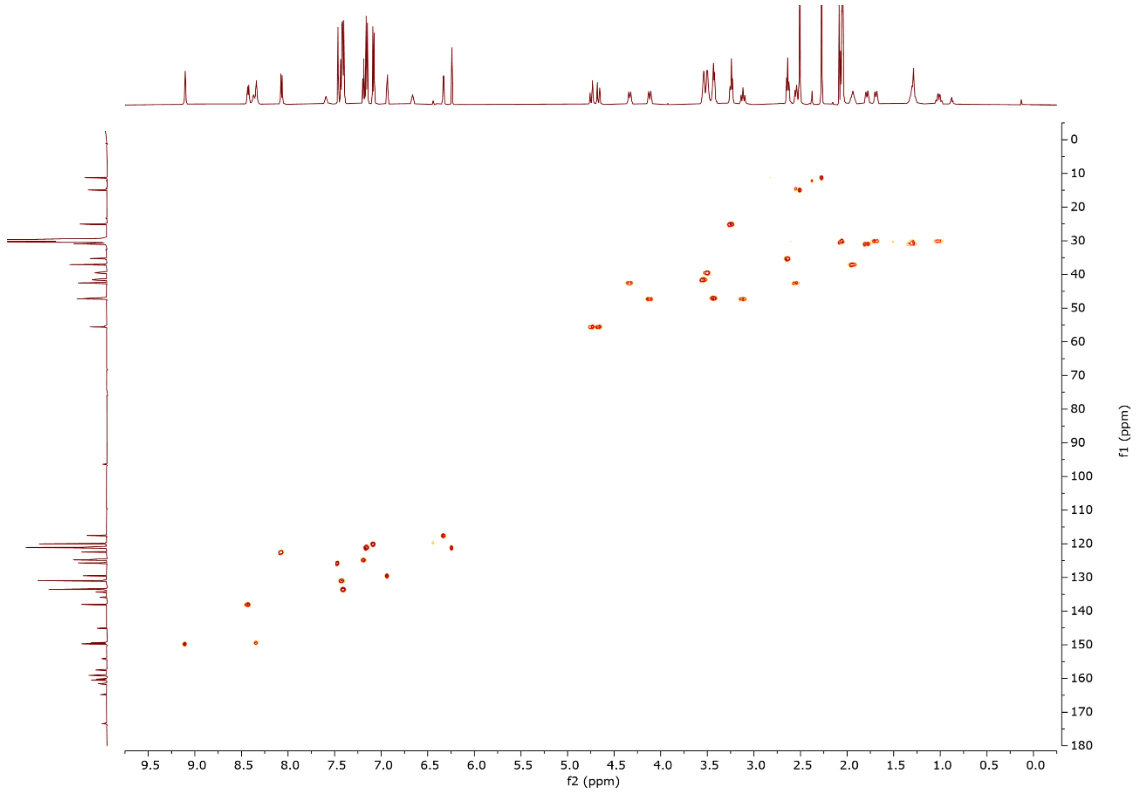

Figure S239. ^{1}H-^{13}C HSQC spectrum of **Evo-3** [$(CD_3)_2CO$].

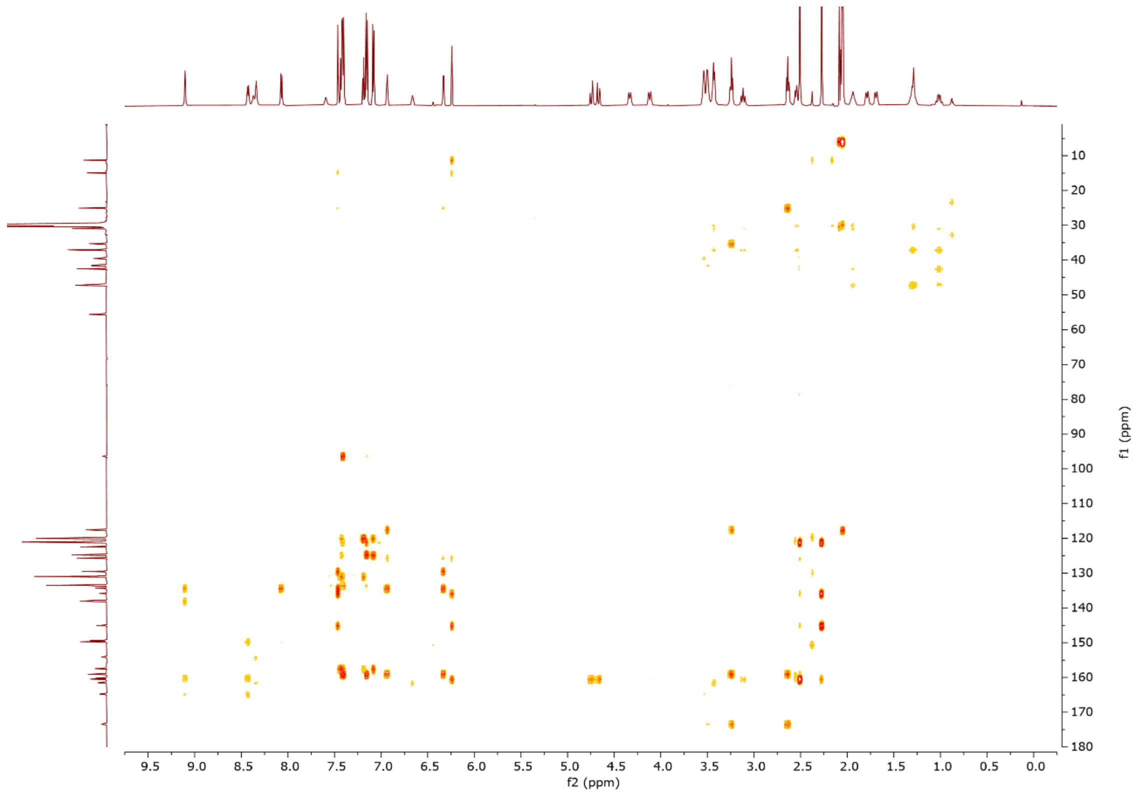

Figure S240. ^{1}H-^{13}C HMBC spectrum of **Evo-3** [$(CD_3)_2CO$].

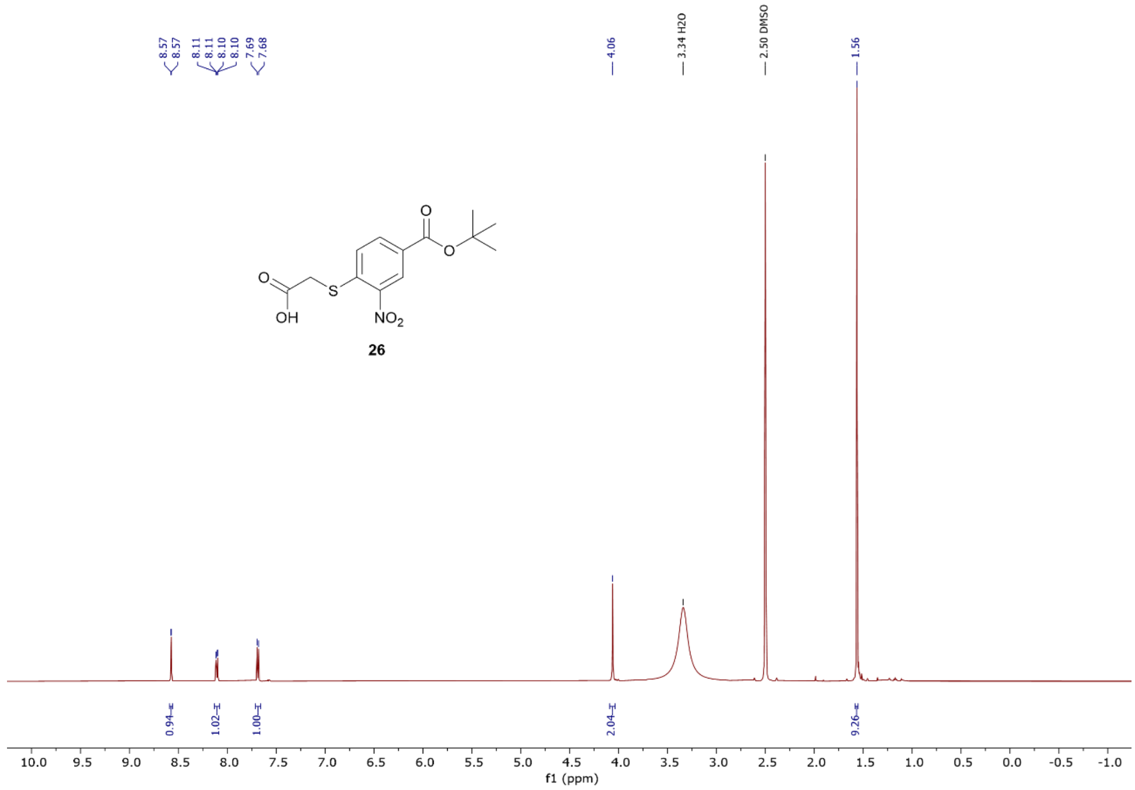

Figure S241. ^{1}H NMR spectrum of compound **26** (600 MHz, $(CD_3)_2SO$).

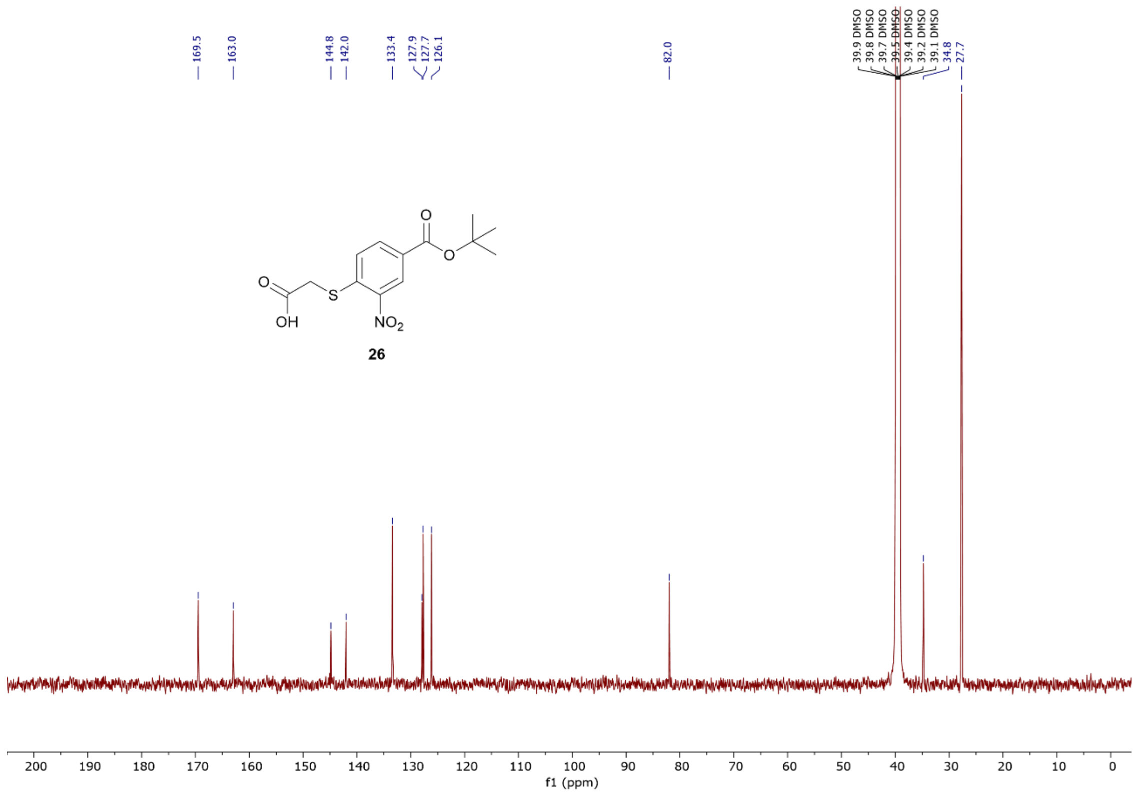

Figure S242. ^{13}C NMR spectrum of compound **26** (151 MHz, $(CD_3)_2SO$).

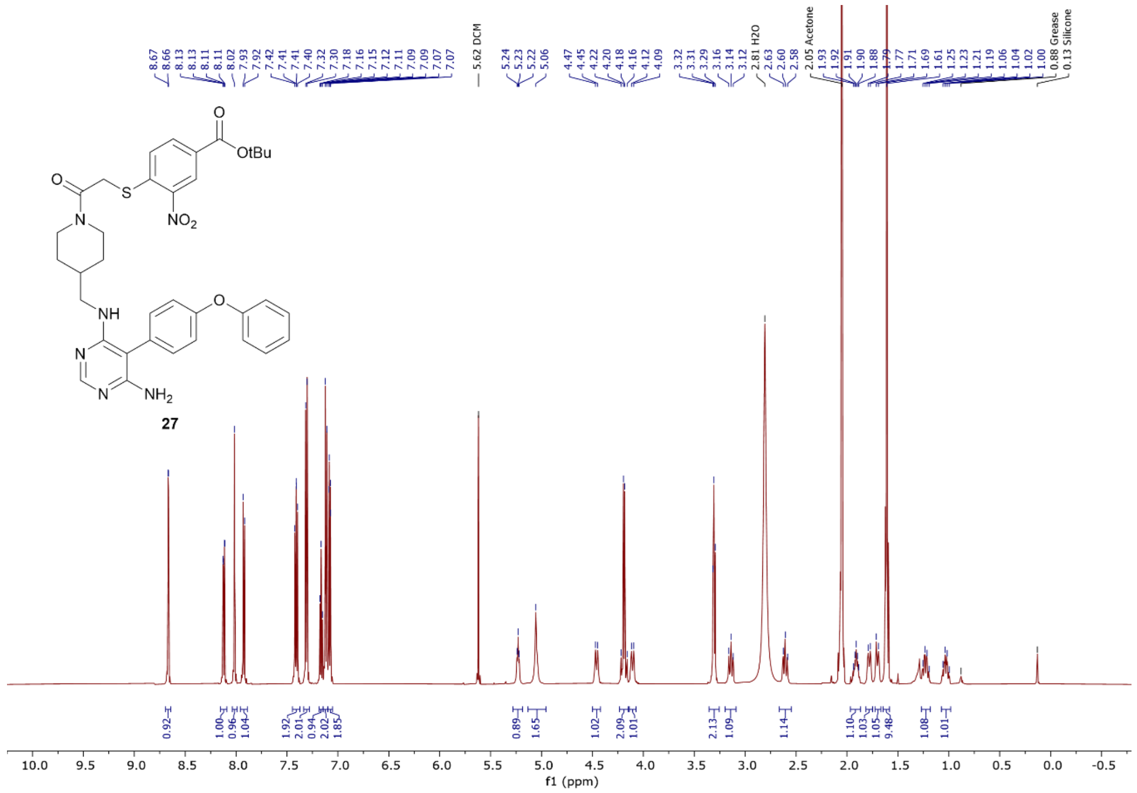

Figure S243. ^{1}H NMR spectrum of compound **27** (600 MHz, $(CD_3)_2CO$).

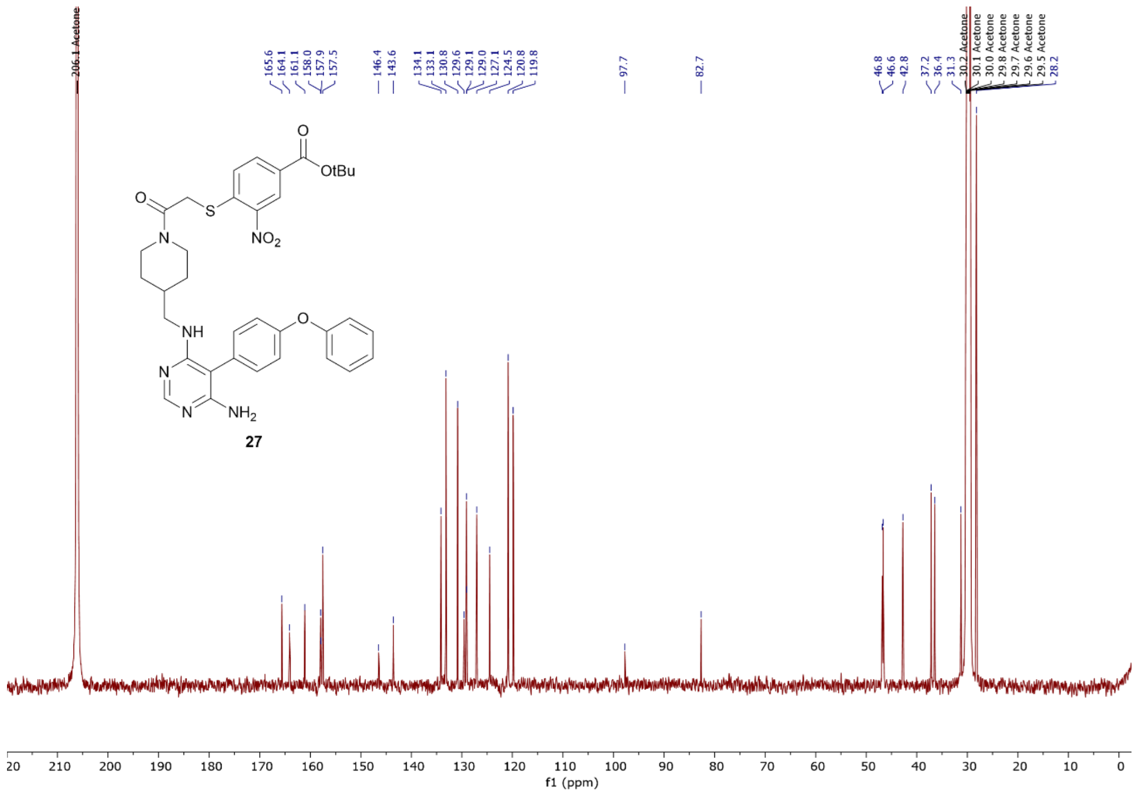

Figure S244. ^{13}C NMR spectrum of compound **27** (151 MHz, $(CD_3)_2CO$).

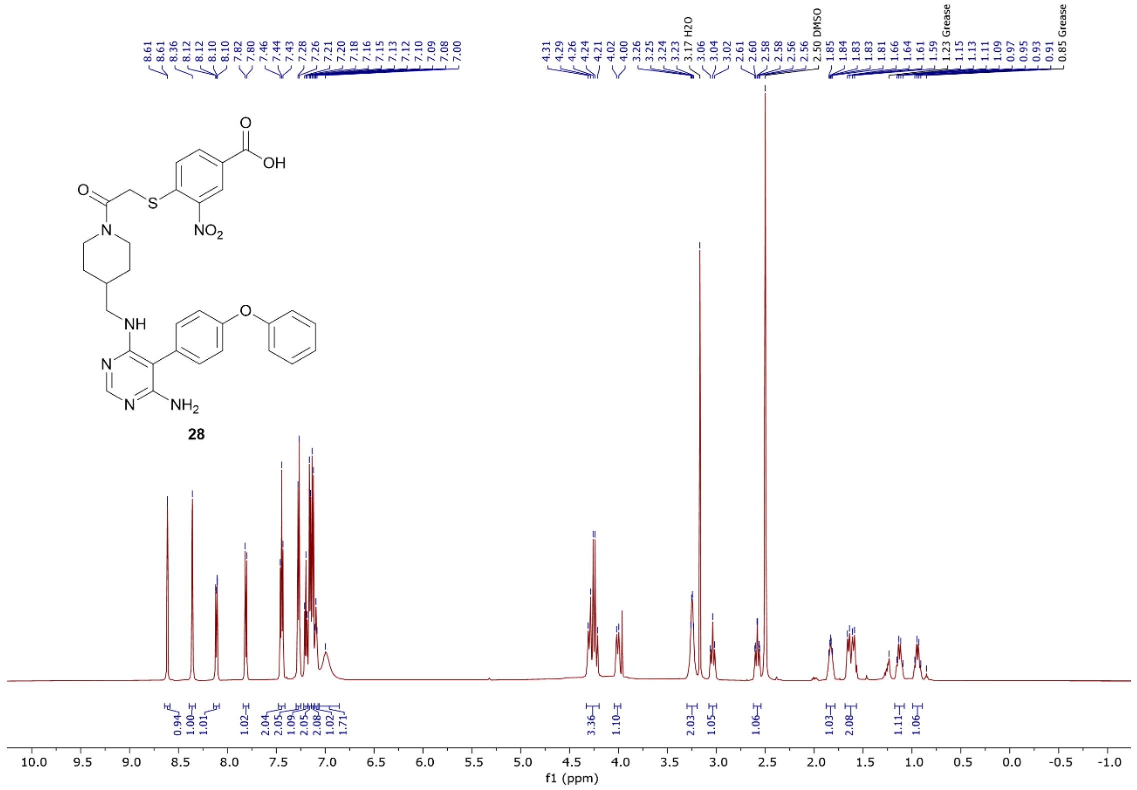

Figure S245. ^{1}H NMR spectrum of compound **28** (600 MHz, $(CD_3)_2SO$).

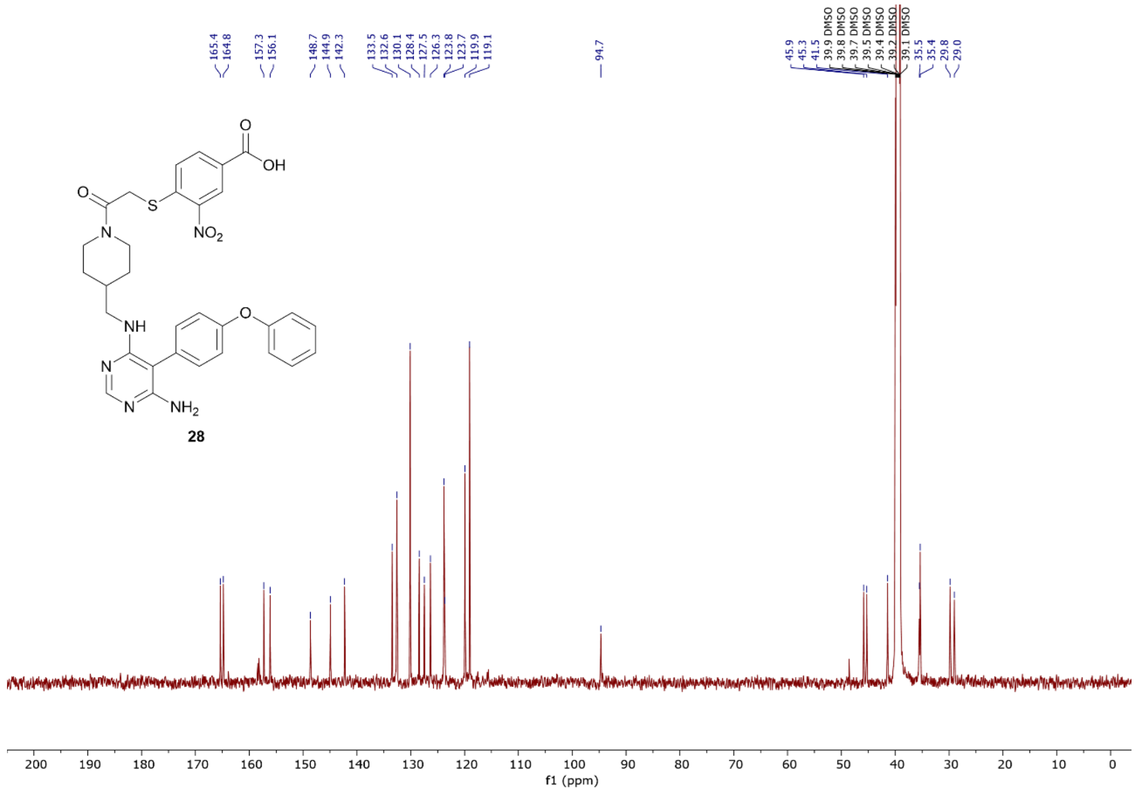

Figure S246. ^{13}C NMR spectrum of compound **28** (151 MHz, $(CD_3)_2SO$).

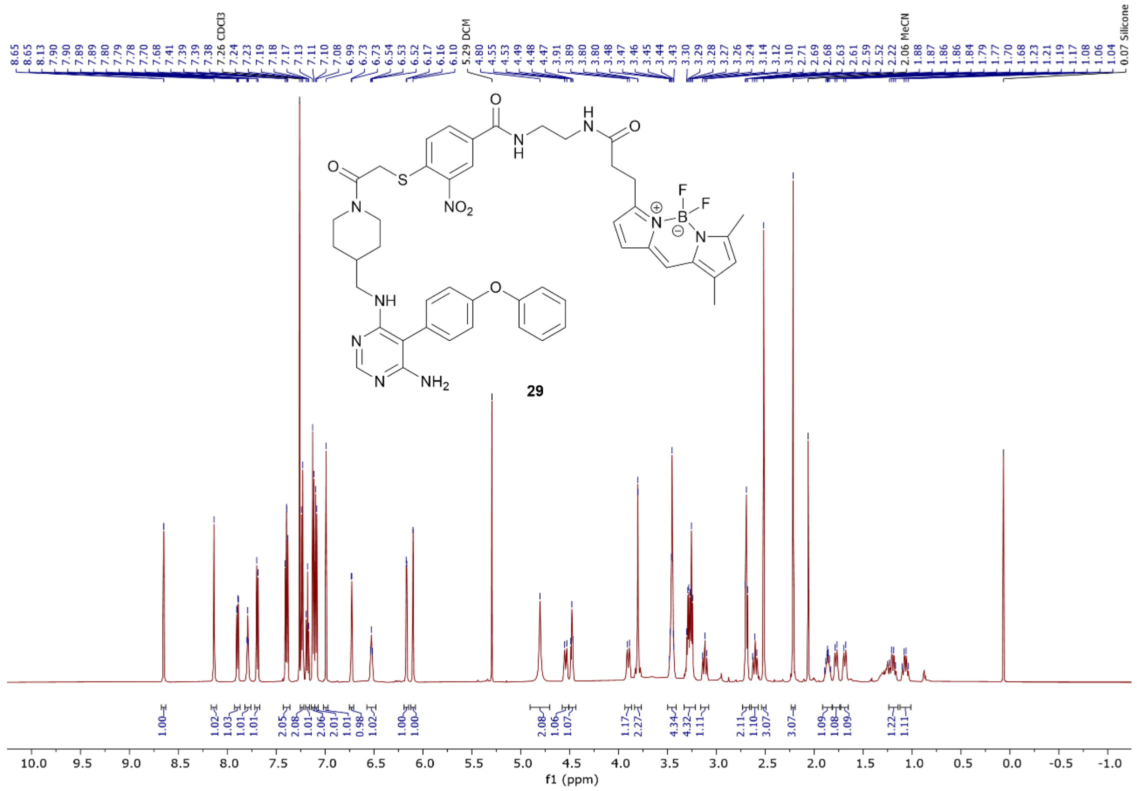

Figure S247. ^{1}H NMR spectrum of compound **29** (600 MHz, $CDCl_3$).

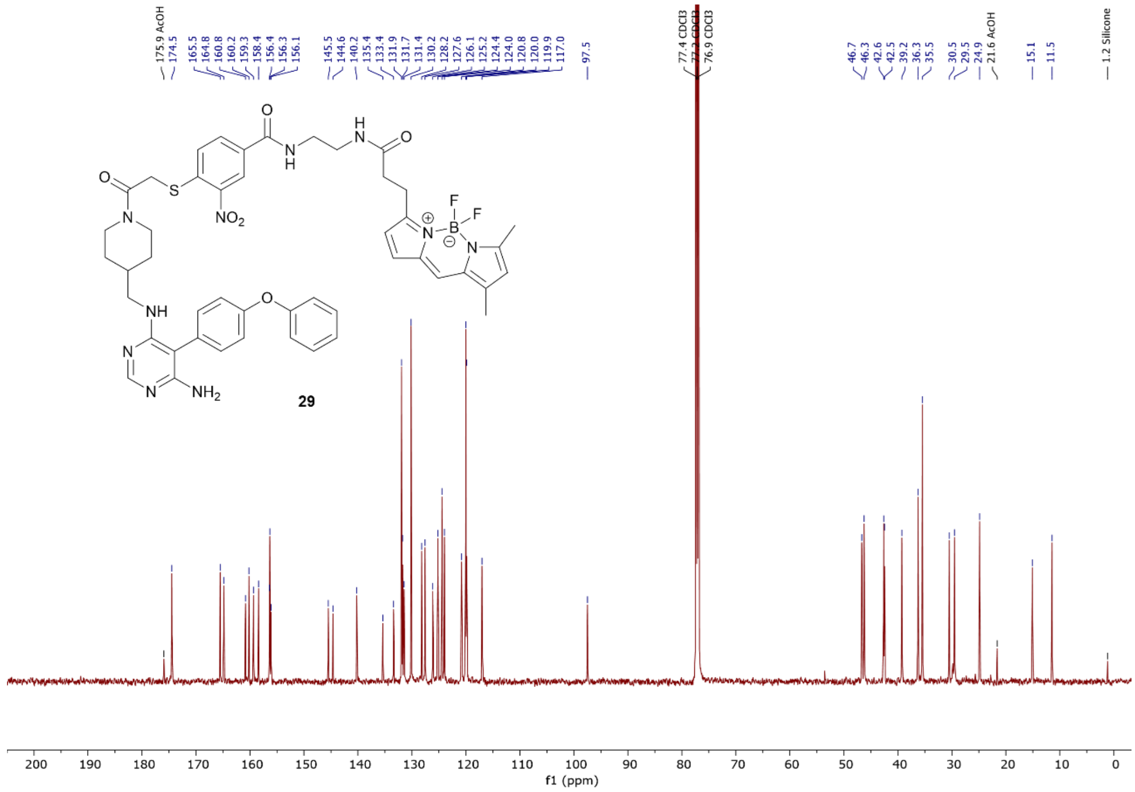

Figure S248. ^{13}C NMR spectrum of compound **29** (151 MHz, $CDCl_3$).

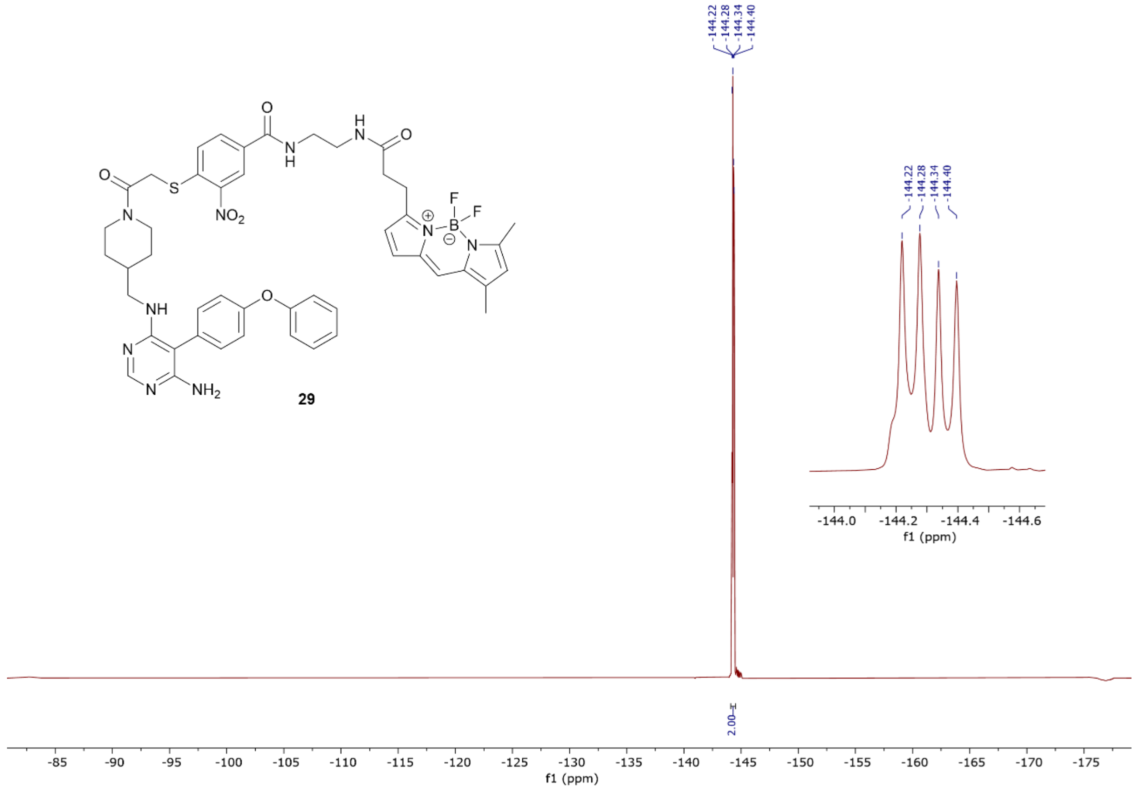

Figure S249. ^{19}F NMR spectrum compound **29** (564 MHz, $CDCl_3$).

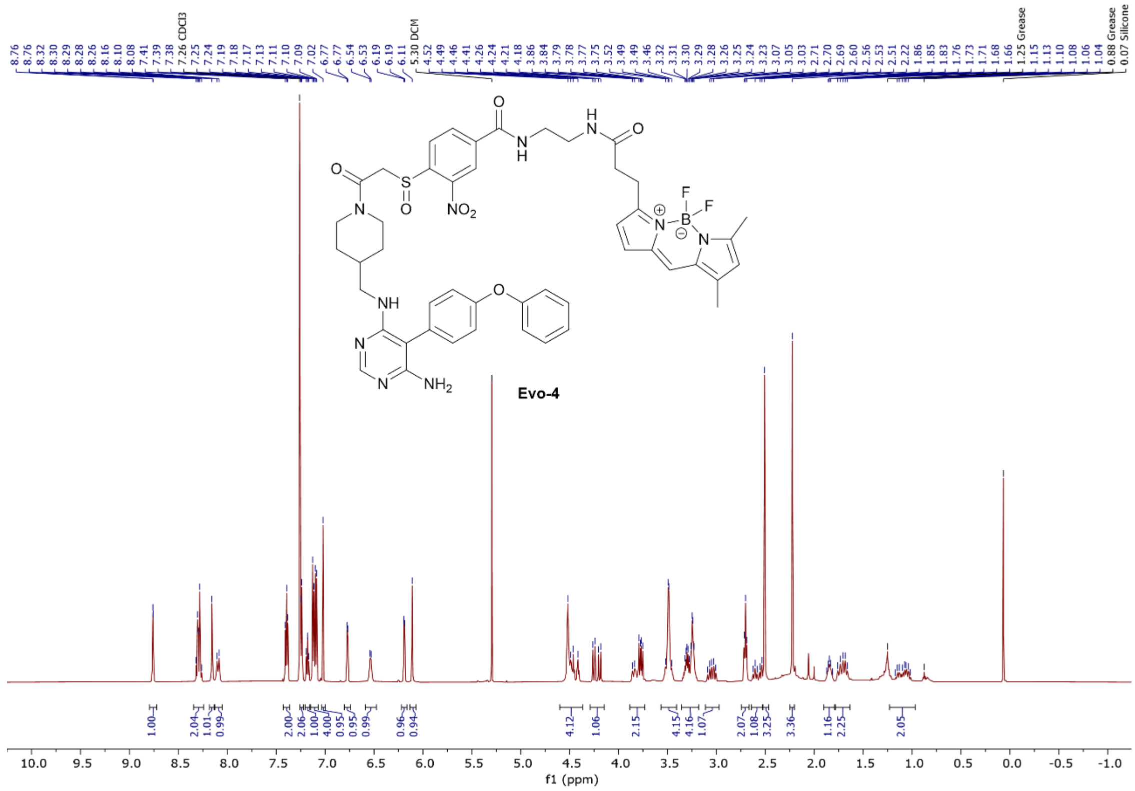

Figure S250. ^{1}H NMR spectrum of **Evo-4** (600 MHz, $CDCl_3$).

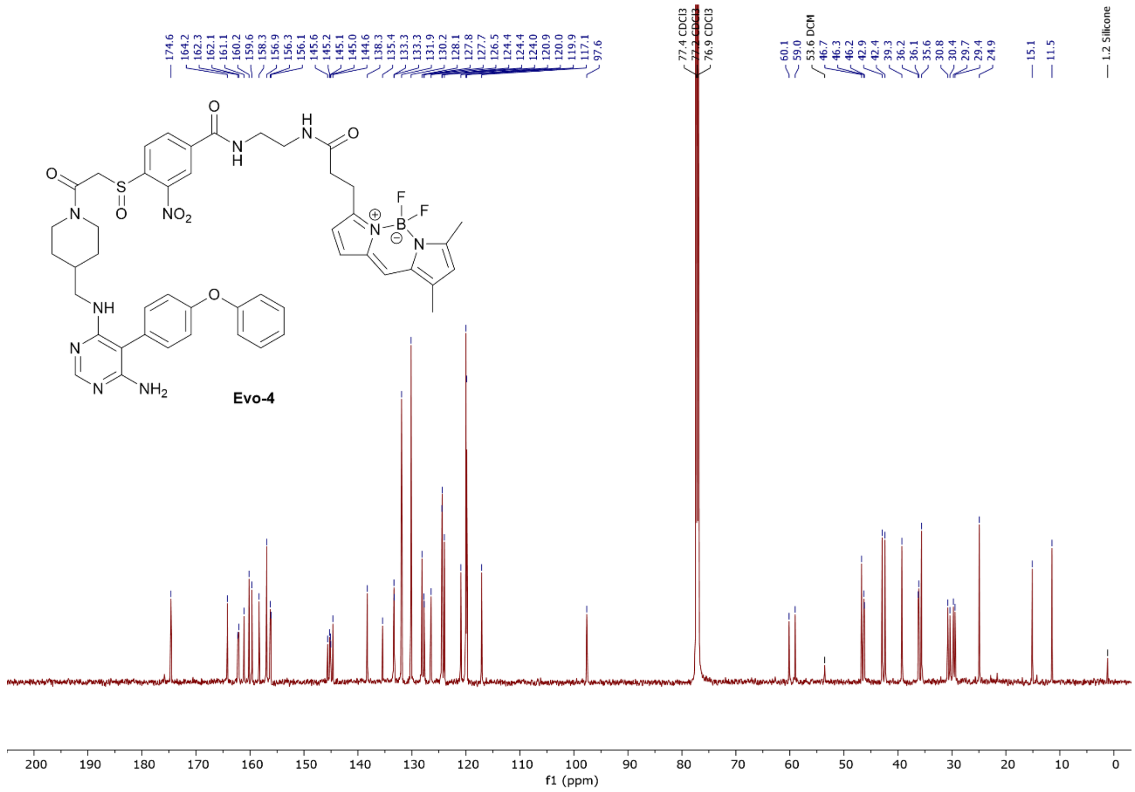

Figure S251. ^{13}C NMR spectrum of **Evo-4** (151 MHz, $CDCl_3$).

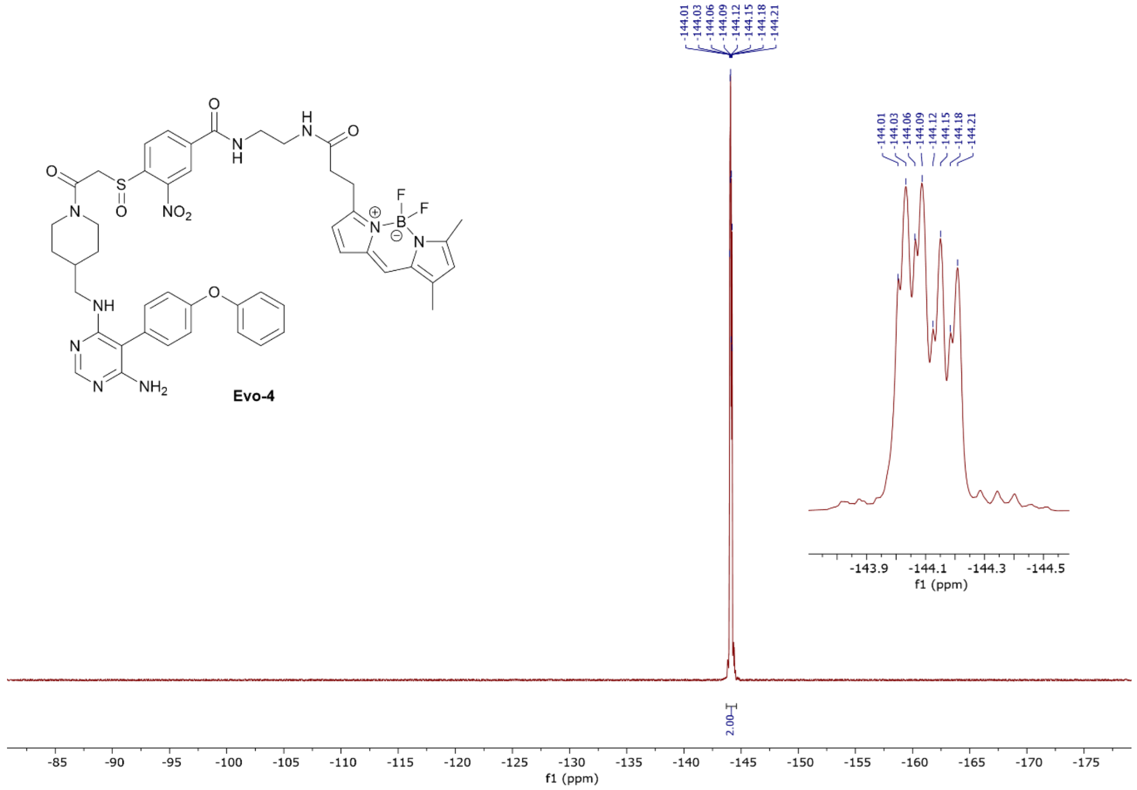

Figure S252. ^{19}F NMR spectrum of **Evo-4** (564 MHz, $CDCl_3$).

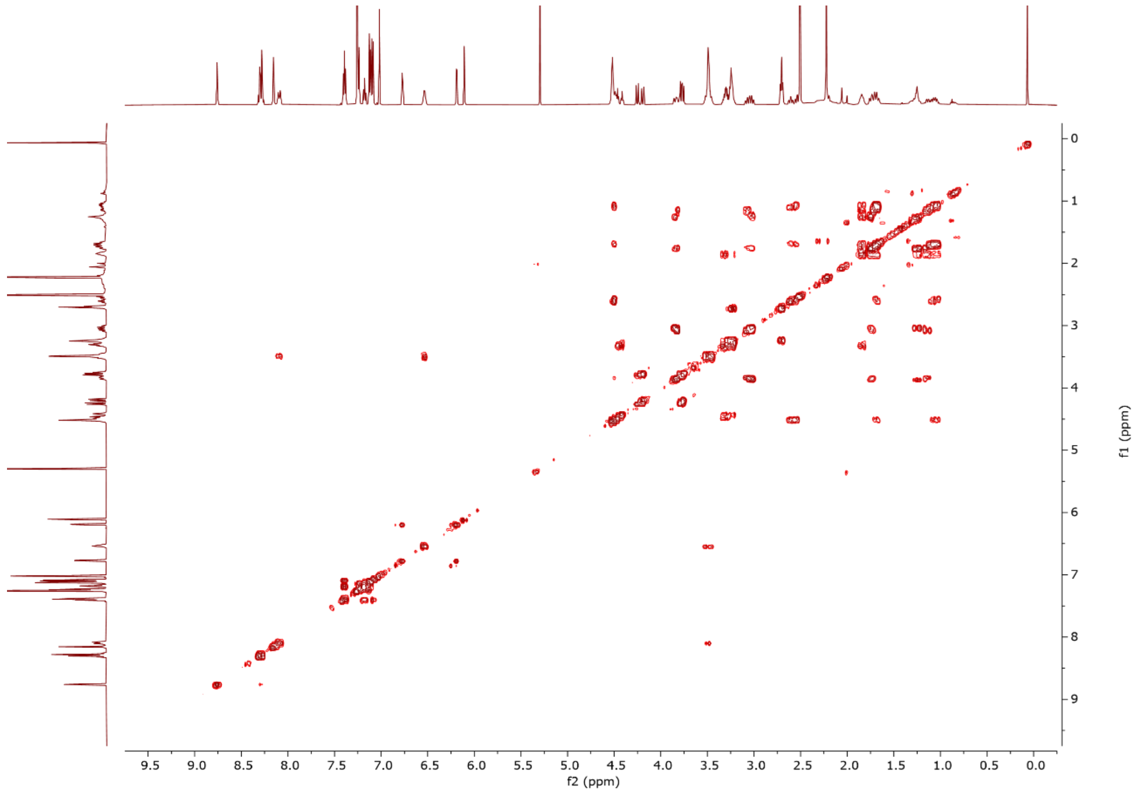

Figure S253. ^1H-^1H COSY spectrum of **Evo-4** ($CDCl_3$).

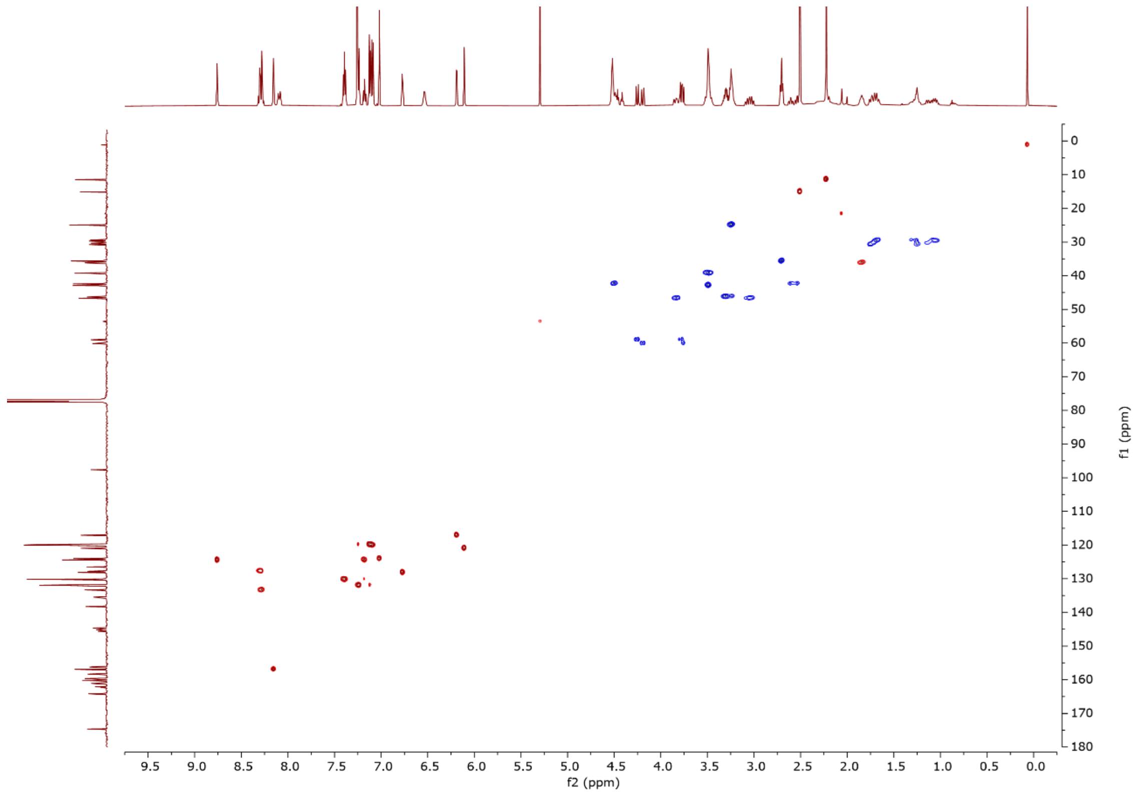

Figure S254. ^{1}H-^{13}C HSQC spectrum of **Evo-4** ($CDCl_3$).

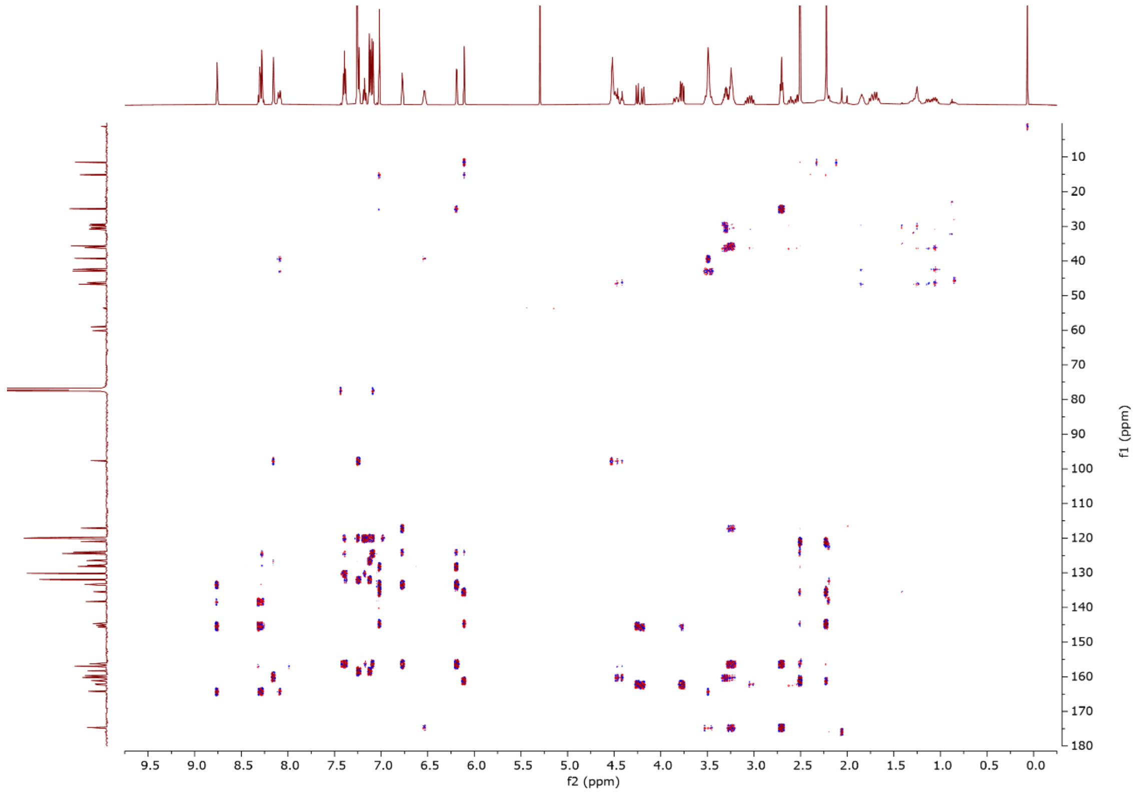

Figure S255. ^{1}H-^{13}C HMBC spectrum of **Evo-4** ($CDCl_3$).

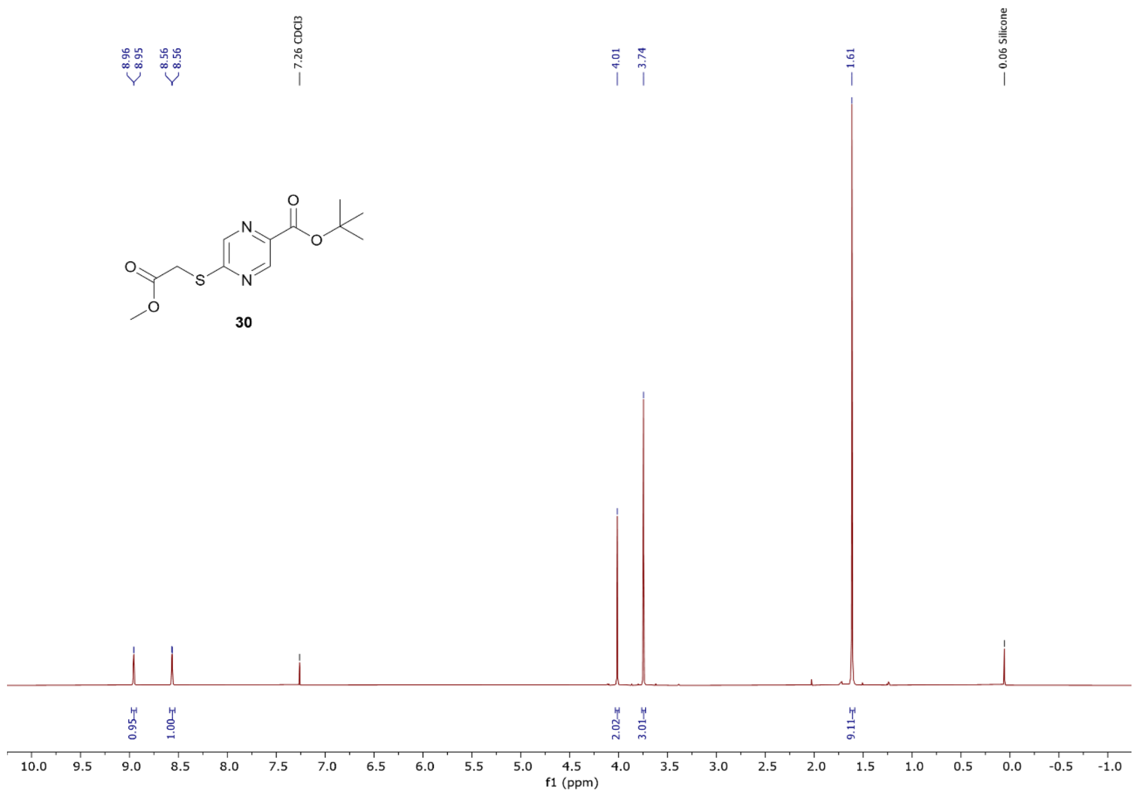

Figure S256. ^{1}H NMR spectrum of compound **30** (600 MHz, $CDCl_3$).

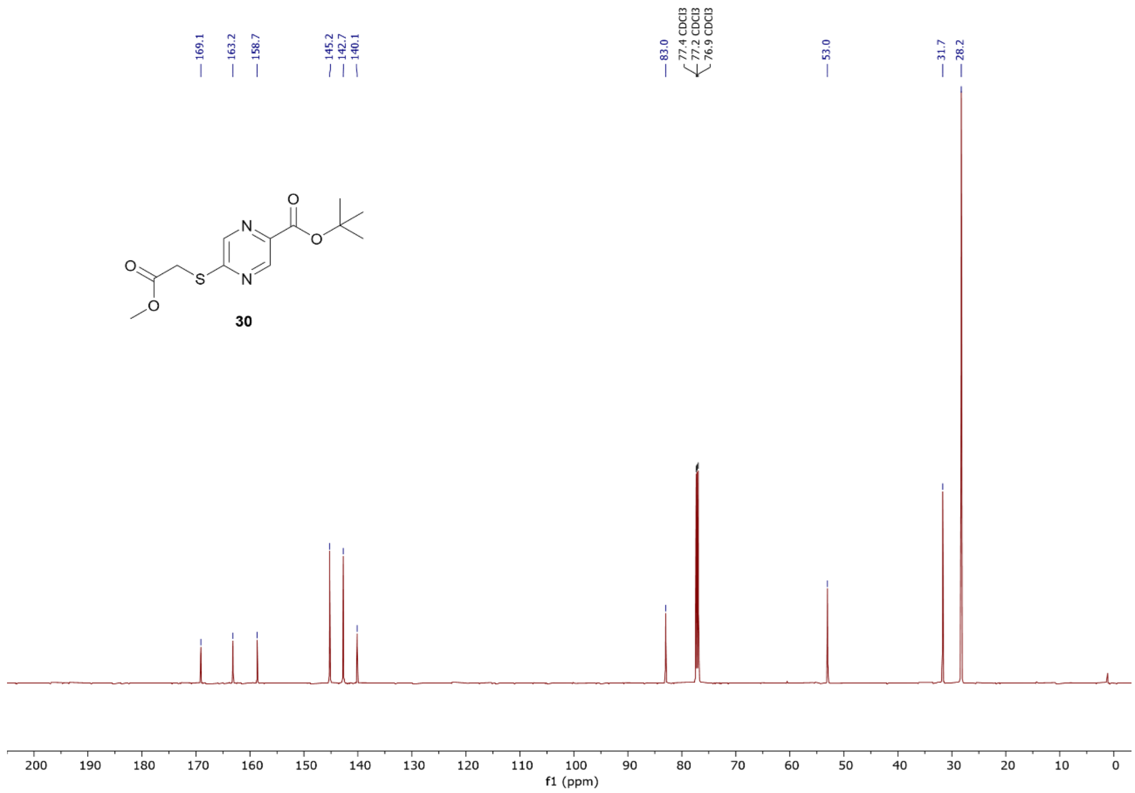

Figure S257. ^{13}C NMR spectrum of compound **30** (151 MHz, $CDCl_3$).

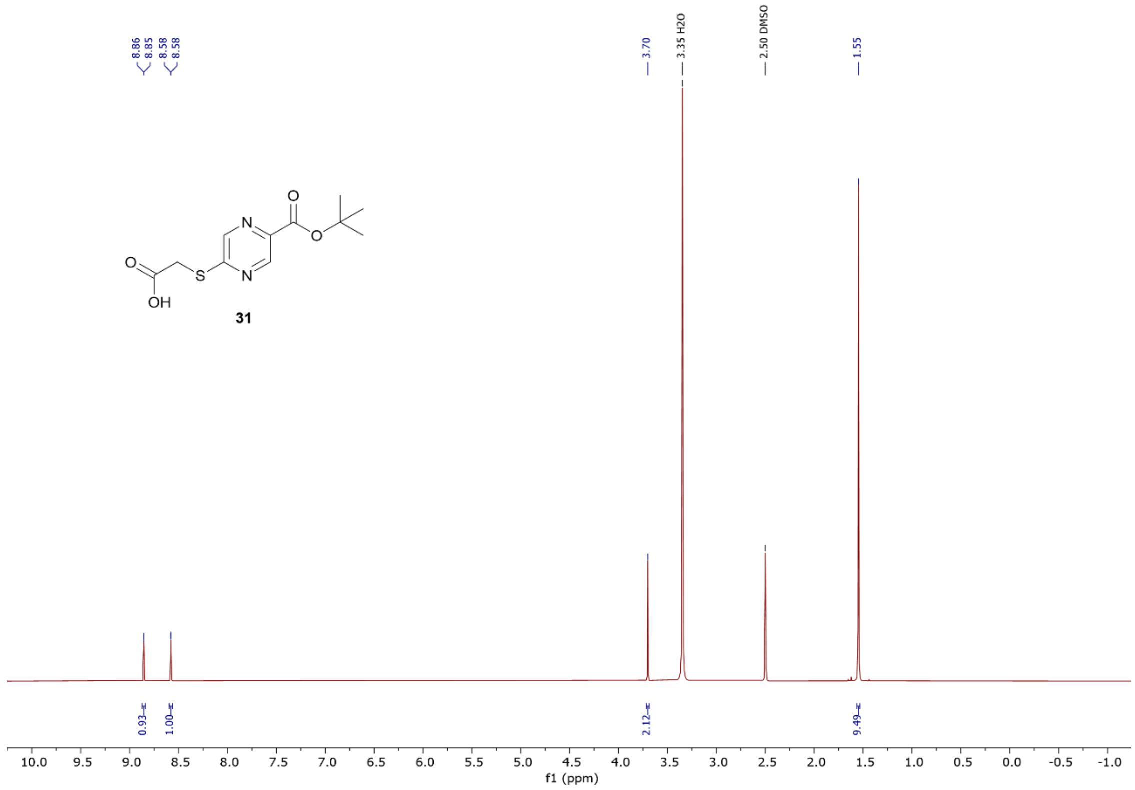

Figure S258. ^{1}H NMR spectrum of compound **31** (600 MHz, $(CD_3)_2SO$).

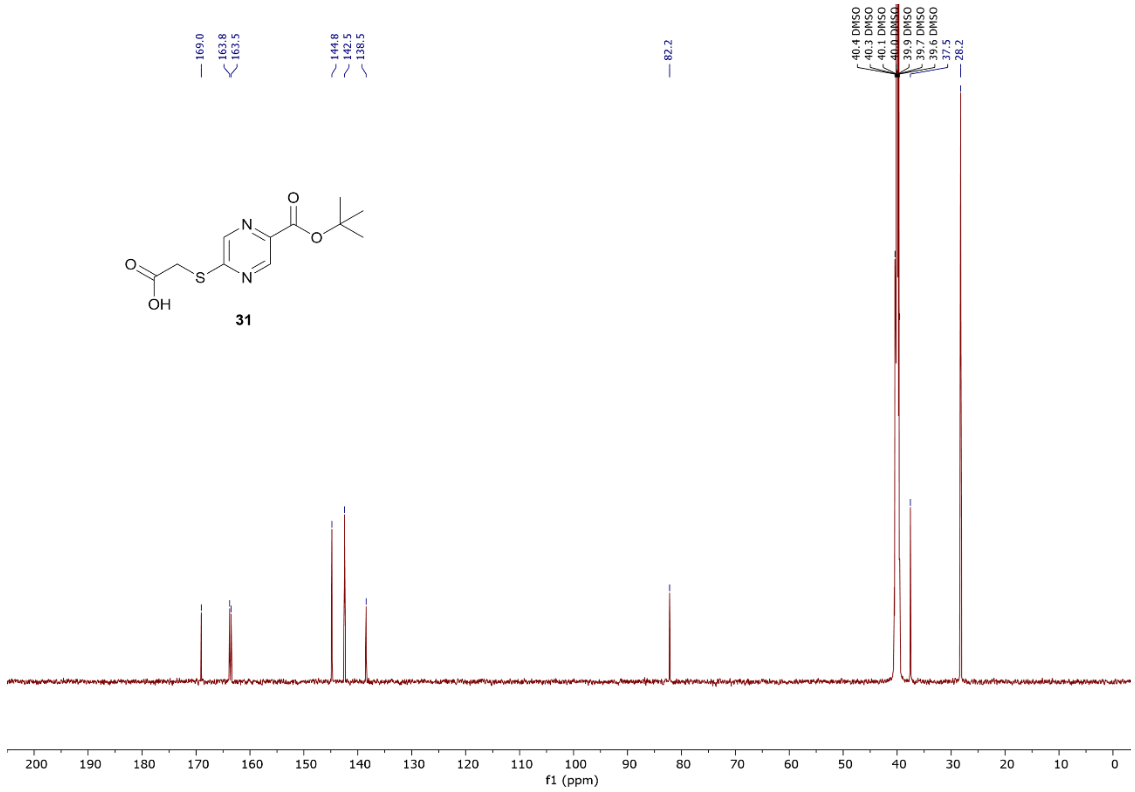

Figure S259. ^{13}C NMR spectrum of compound **31** (151 MHz, $(CD_3)_2SO$).

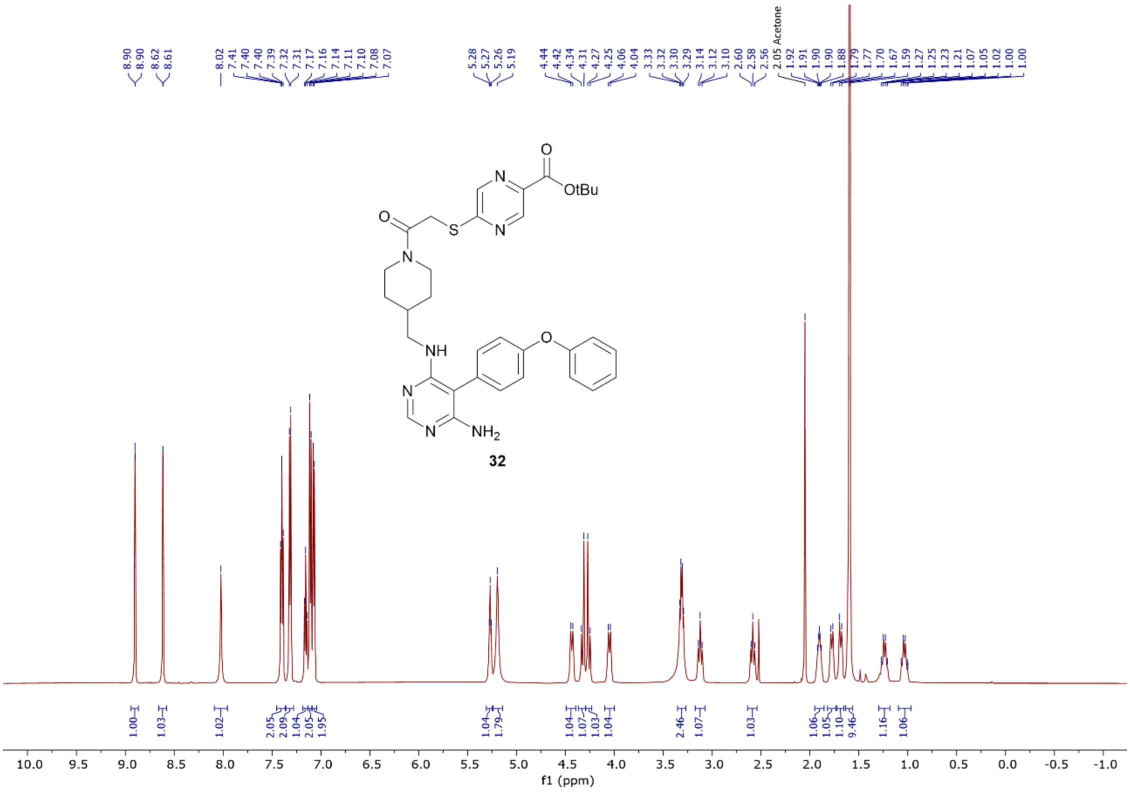

Figure S260. ^{1}H NMR spectrum of compound **32** (600 MHz, $(CD_3)_2CO$).

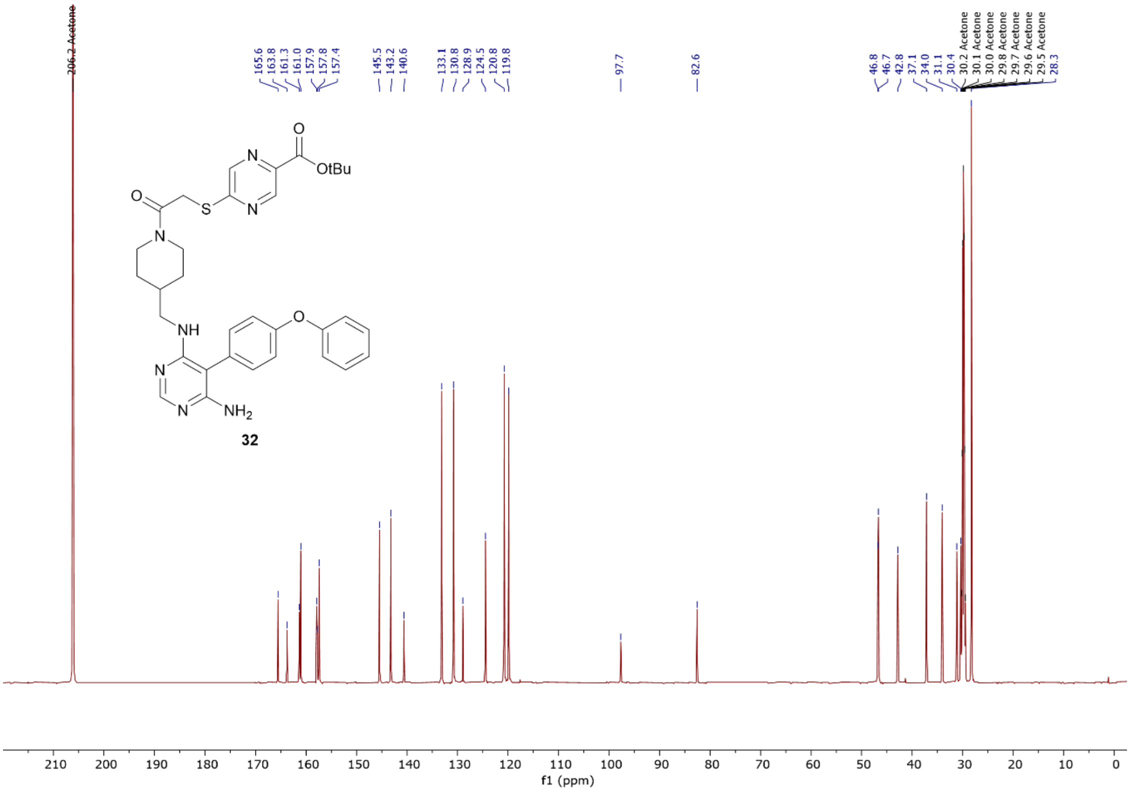

Figure S261. ^{13}C NMR spectrum of compound **32** (151 MHz, $(CD_3)_2CO$).

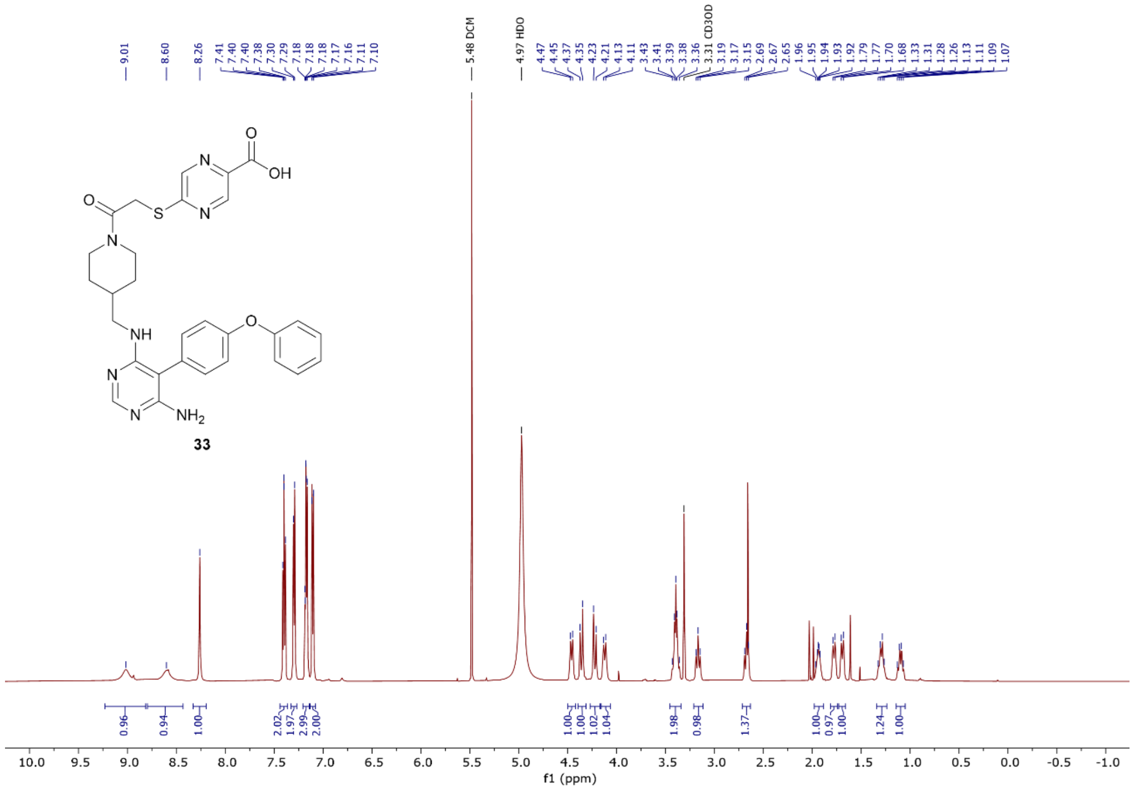

Figure S262. ^{1}H NMR spectrum of compound **33** (600 MHz, CD_3OD).

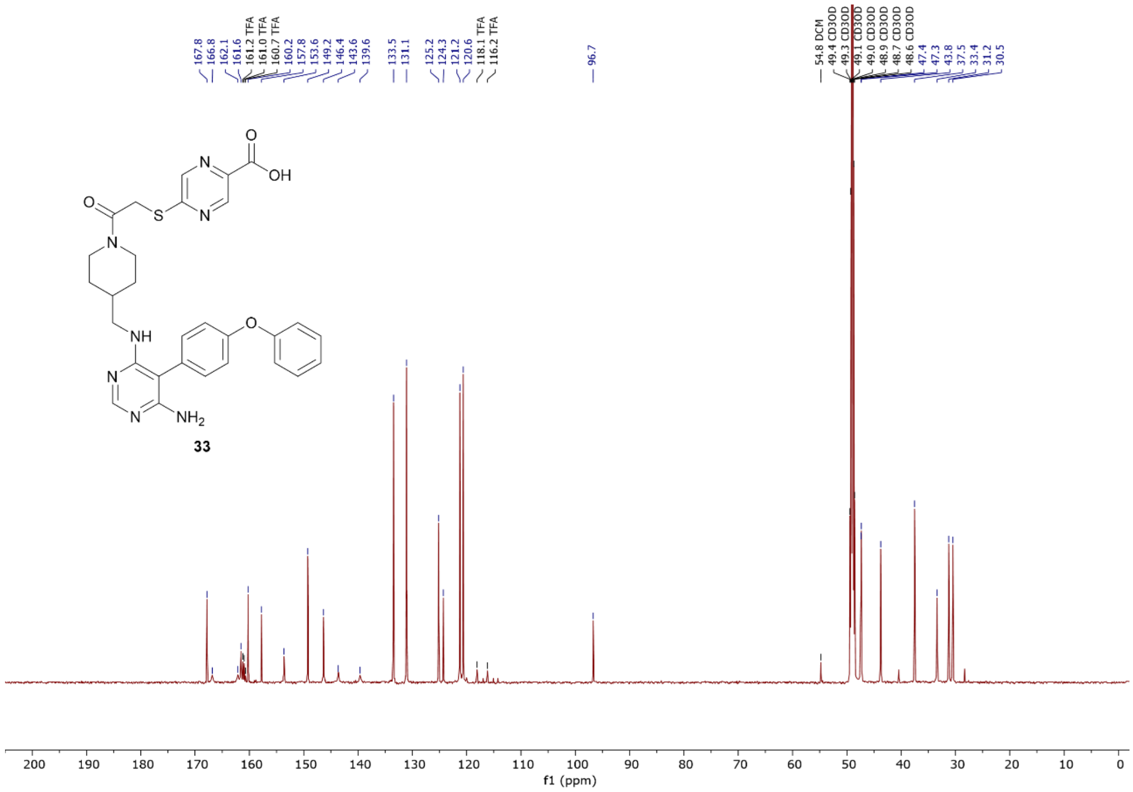

Figure S263. ^{13}C NMR spectrum of compound **33** (151 MHz, CD_3OD).

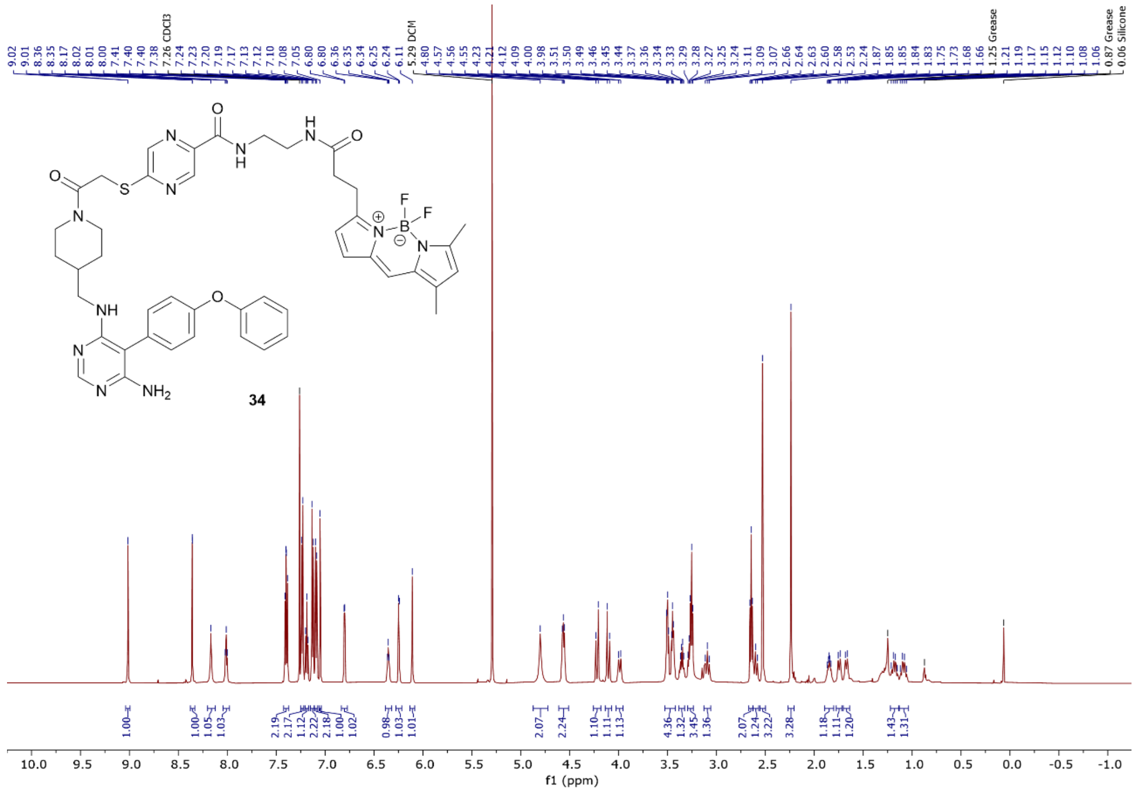

Figure S264. ^{1}H NMR spectrum of compound **34** (600 MHz, $CDCl_3$).

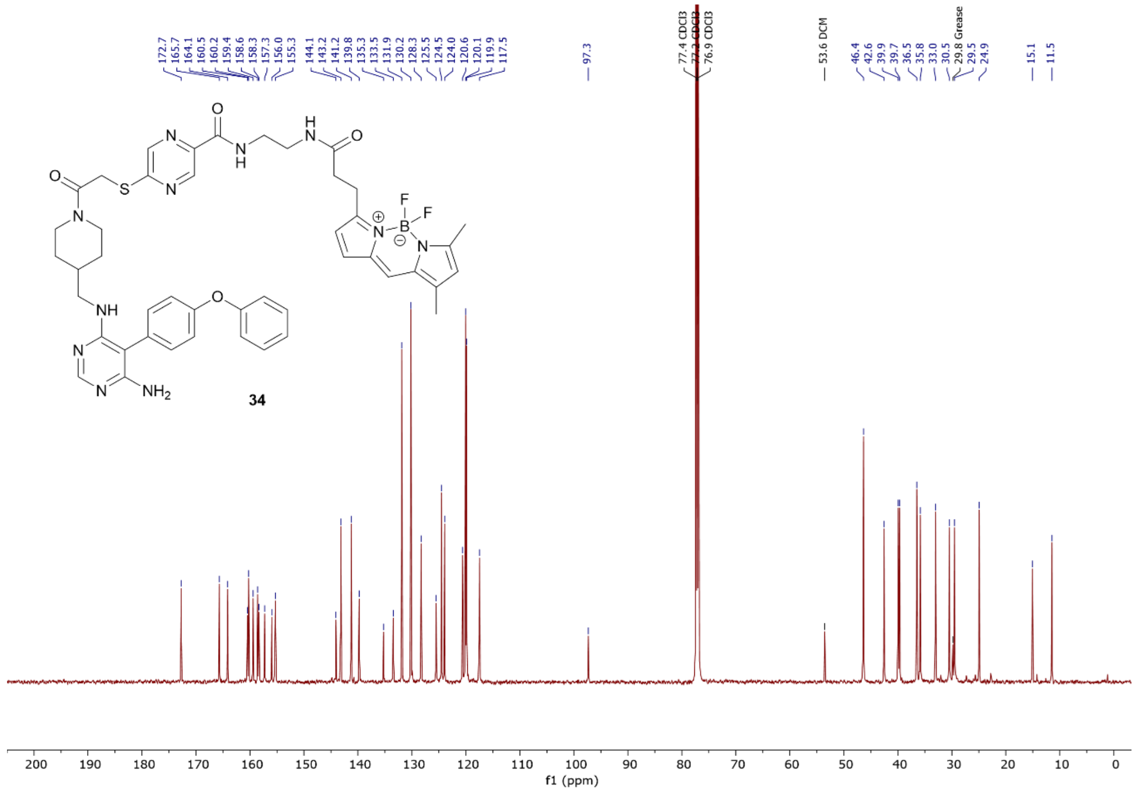

Figure S265. ^{13}C NMR spectrum of compound **34** (151 MHz, $CDCl_3$).

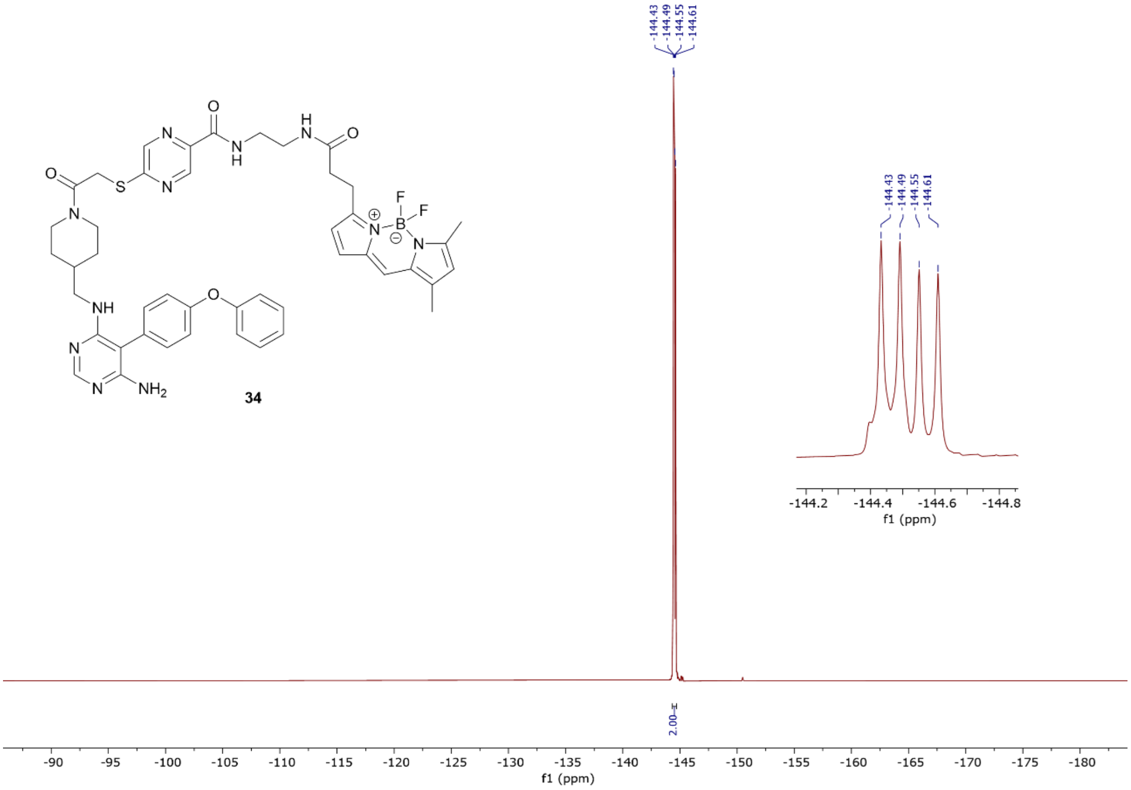

Figure S266. ^{19}F NMR spectrum compound **34** (564 MHz, $CDCl_3$).

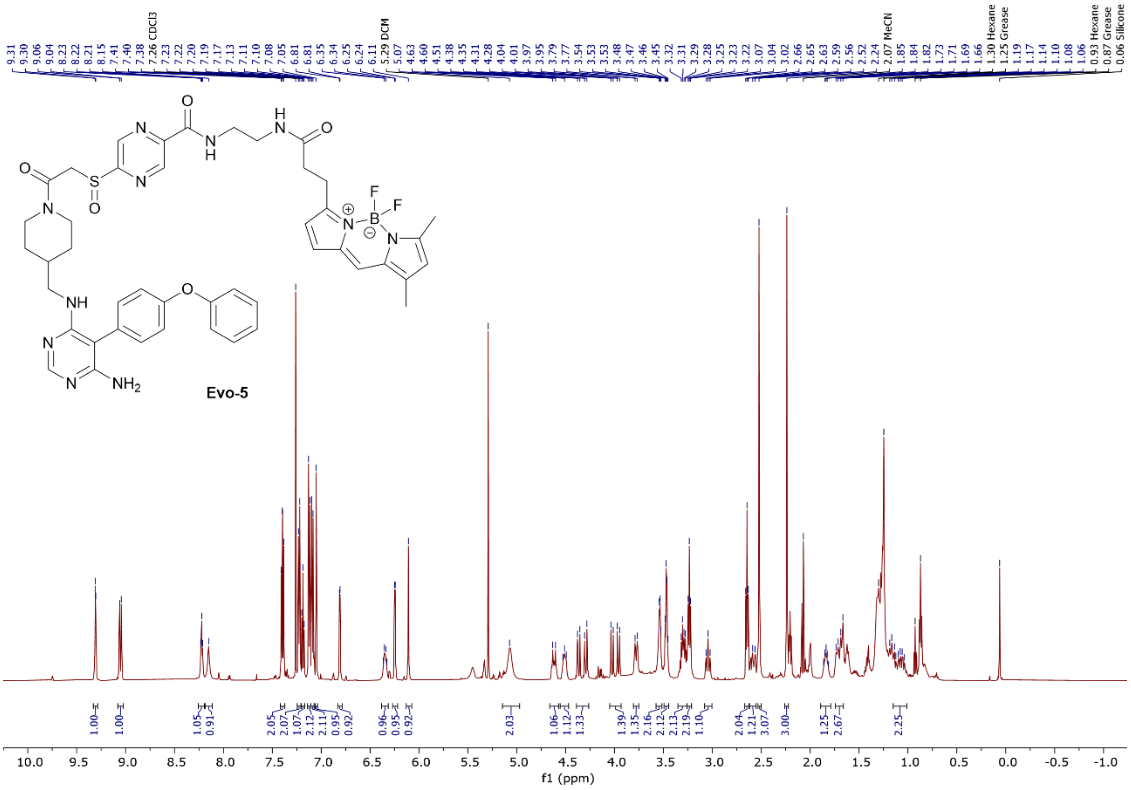

Figure S267. ^{1}H NMR spectrum of **Evo-5** (600 MHz, $CDCl_3$).

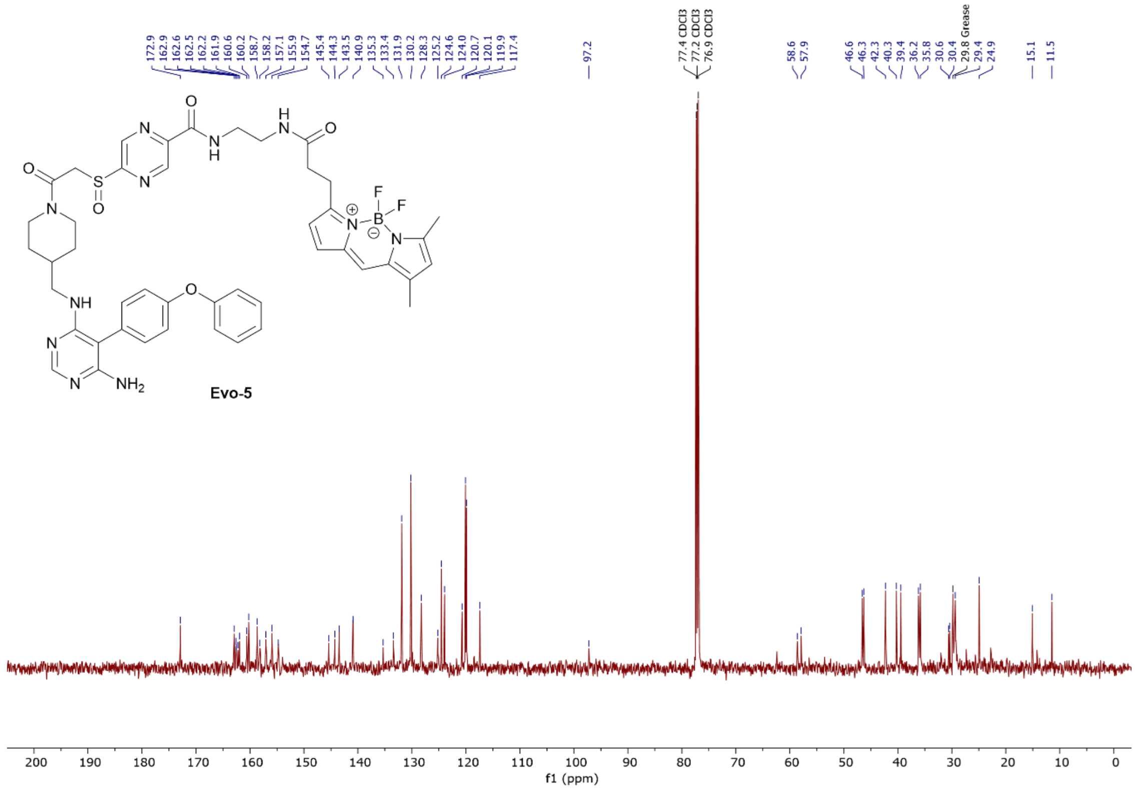

Figure S268. ^{13}C NMR spectrum of **Evo-5** (151 MHz, $CDCl_3$).

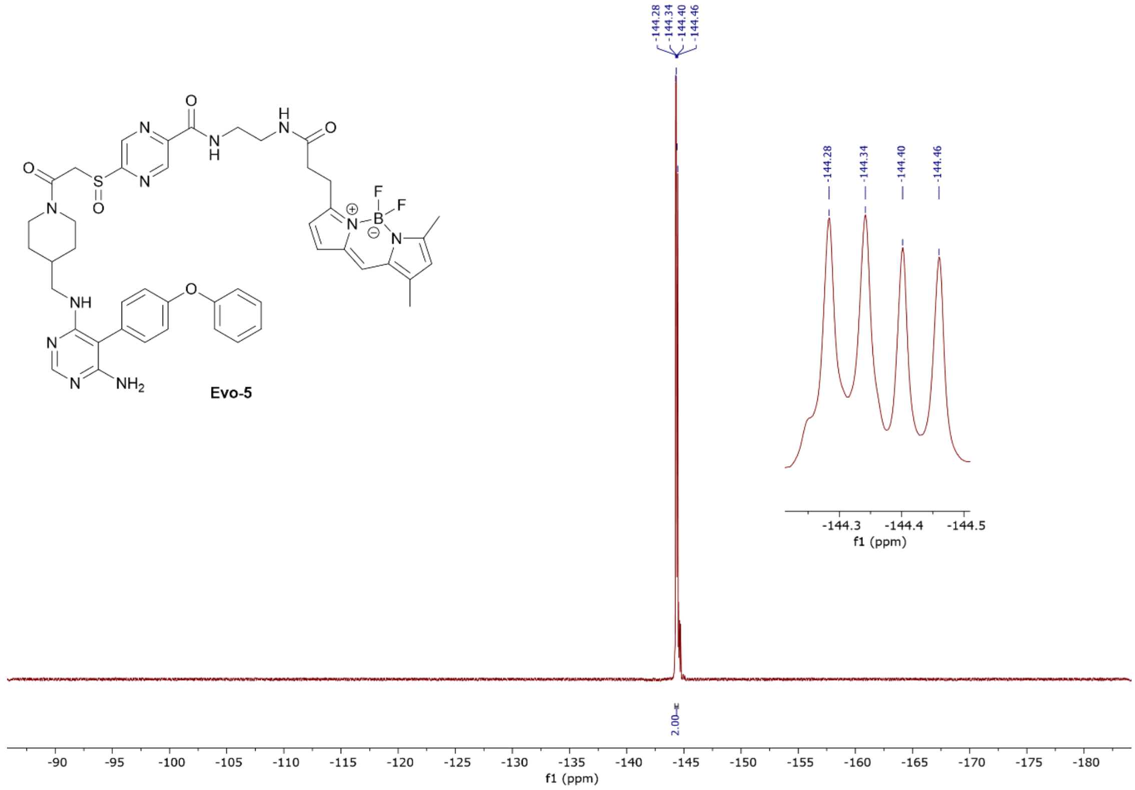

Figure S269. ^{19}F NMR spectrum of **Evo-5** (564 MHz, $CDCl_3$).

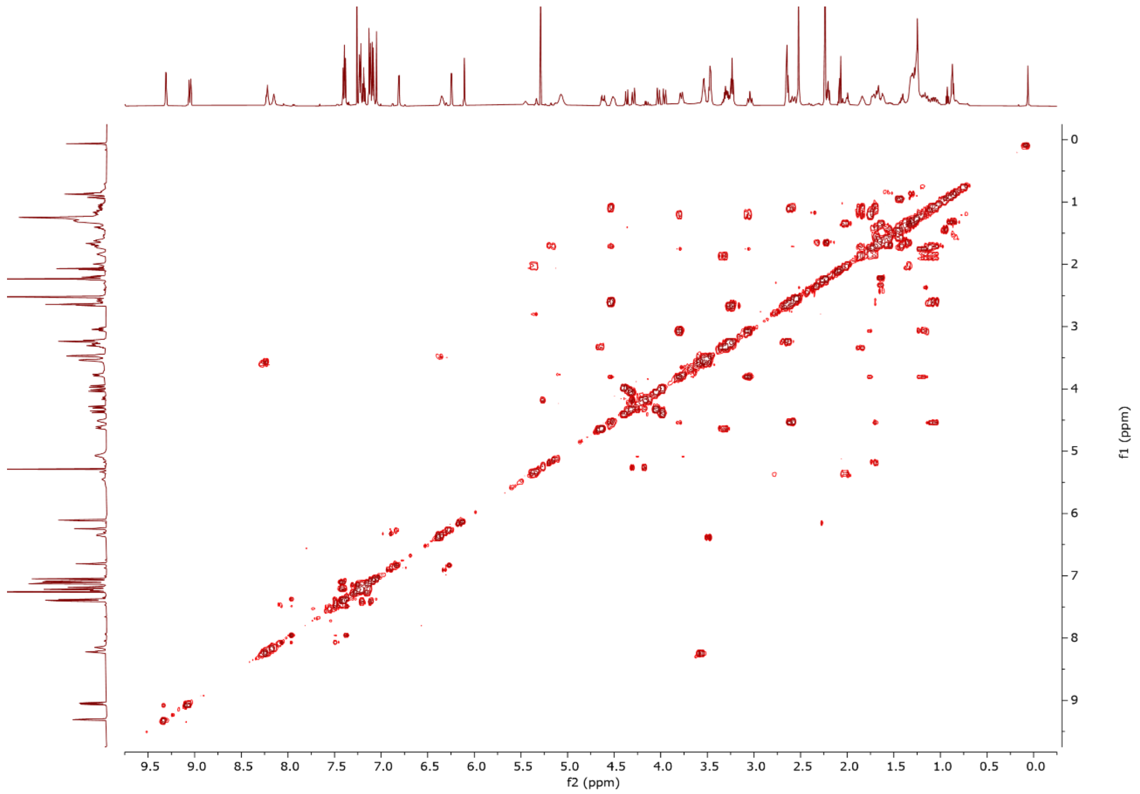

Figure S270. ^{1}H-^{1}H COSY spectrum of **Evo-5** ($CDCl_3$).

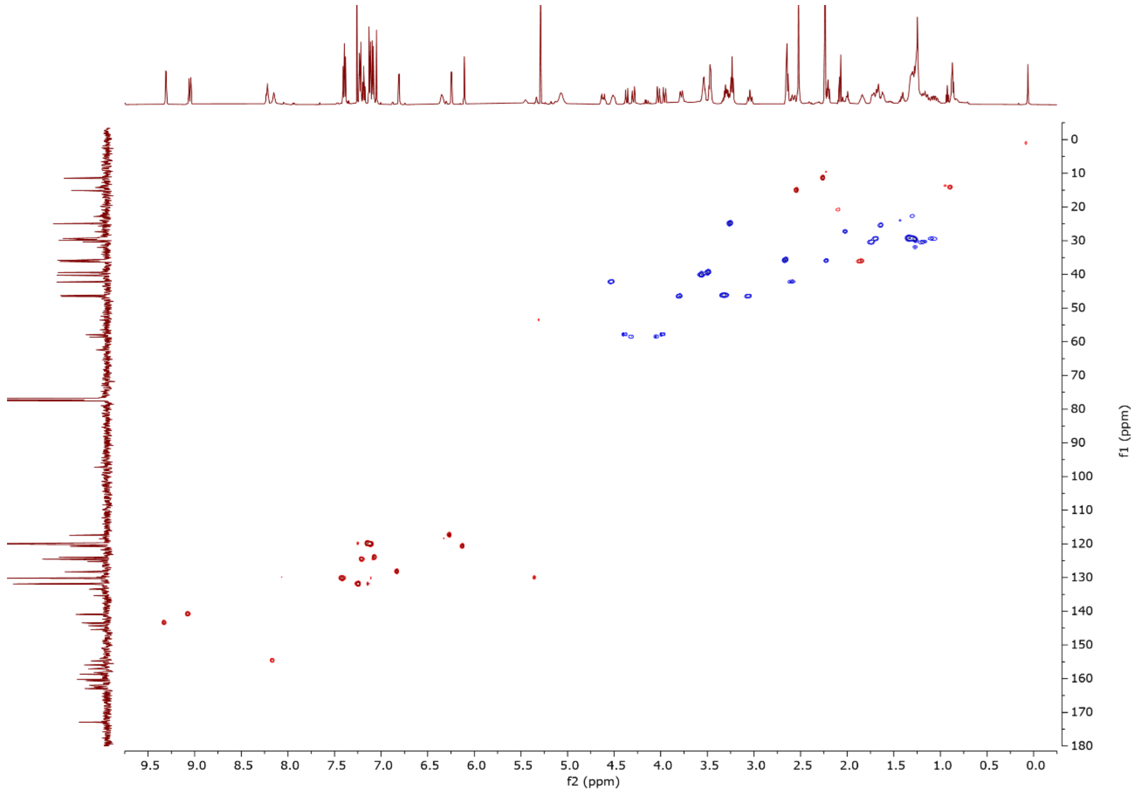

Figure S271. ^{1}H-^{13}C HSQC spectrum of **Evo-5** ($CDCl_3$).

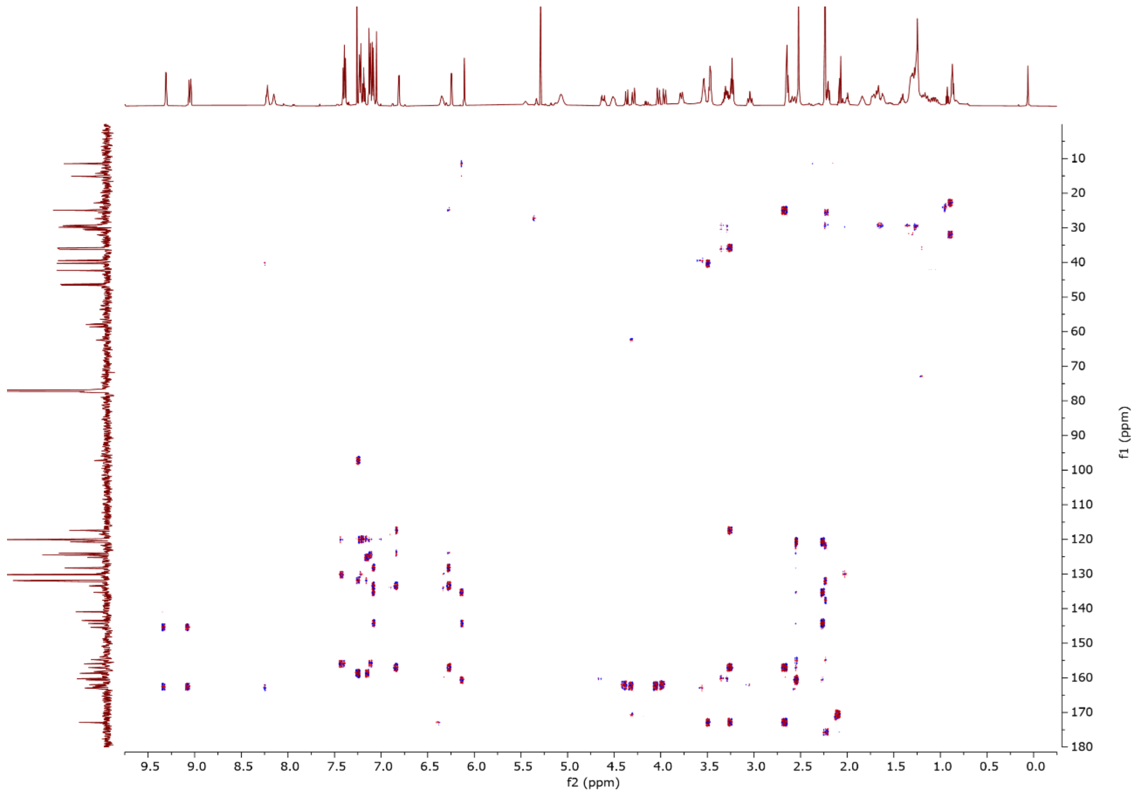

Figure S272. ^{1}H-^{13}C HMBC spectrum of **Evo-5** ($CDCl_3$).

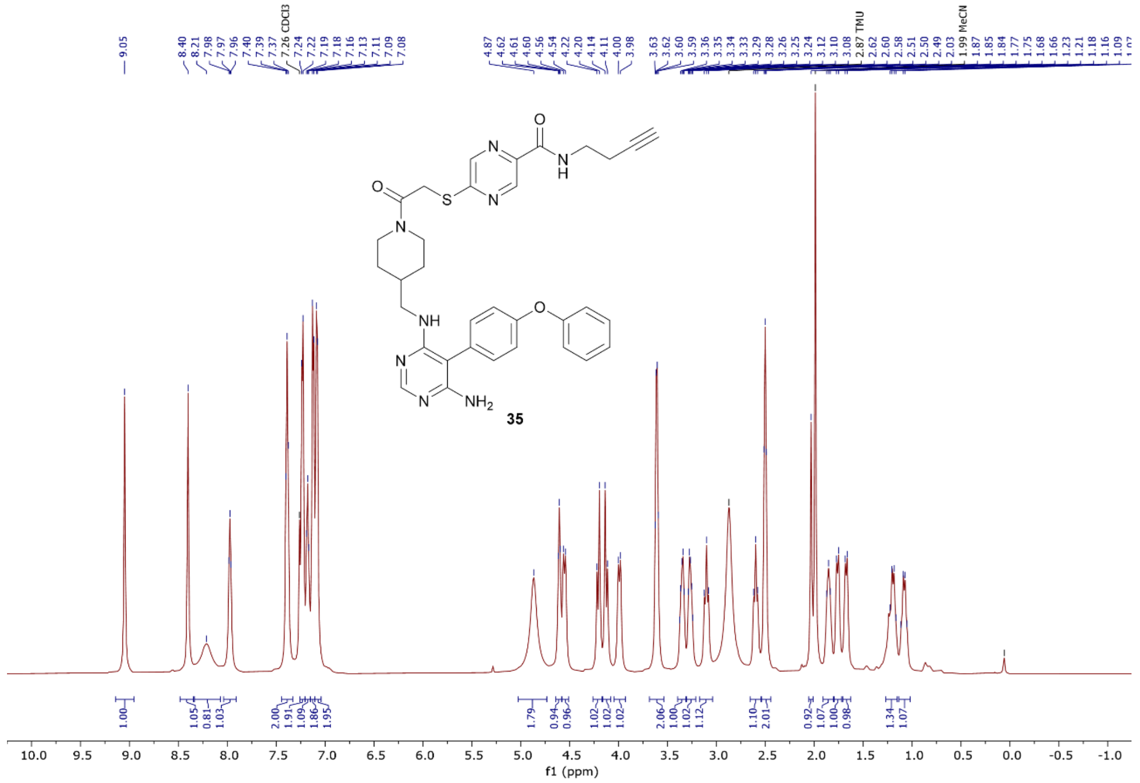

Figure S273. ^{1}H NMR spectrum of compound **35** (600 MHz, $CDCl_3$).

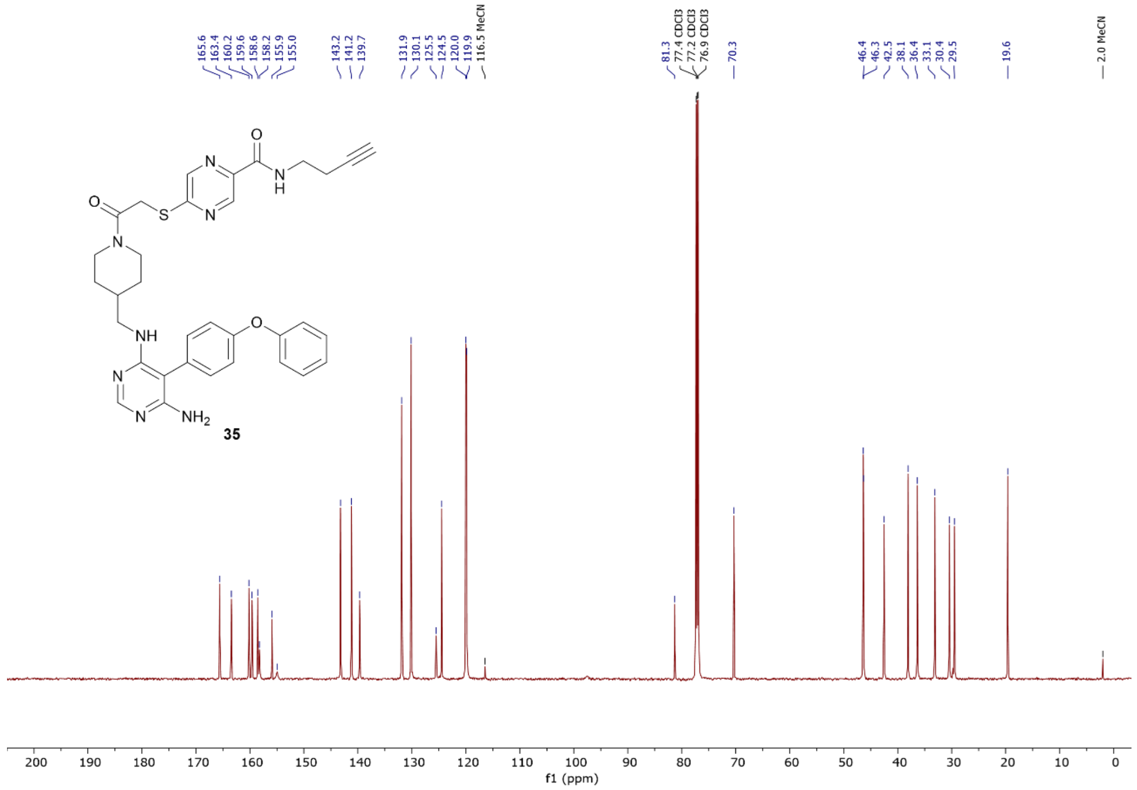

Figure S274. ^{13}C NMR spectrum of compound **35** (151 MHz, $CDCl_3$).

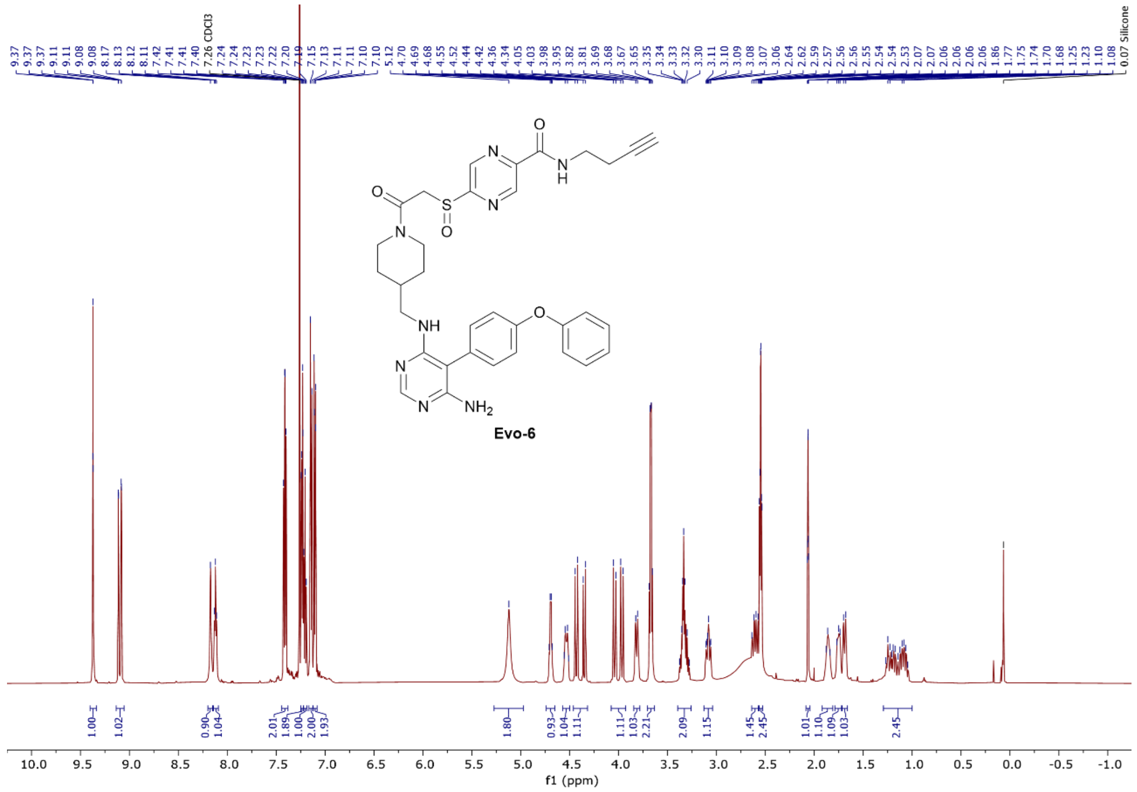

Figure S275. ^{1}H NMR spectrum of **Evo-6** (600 MHz, $CDCl_3$).

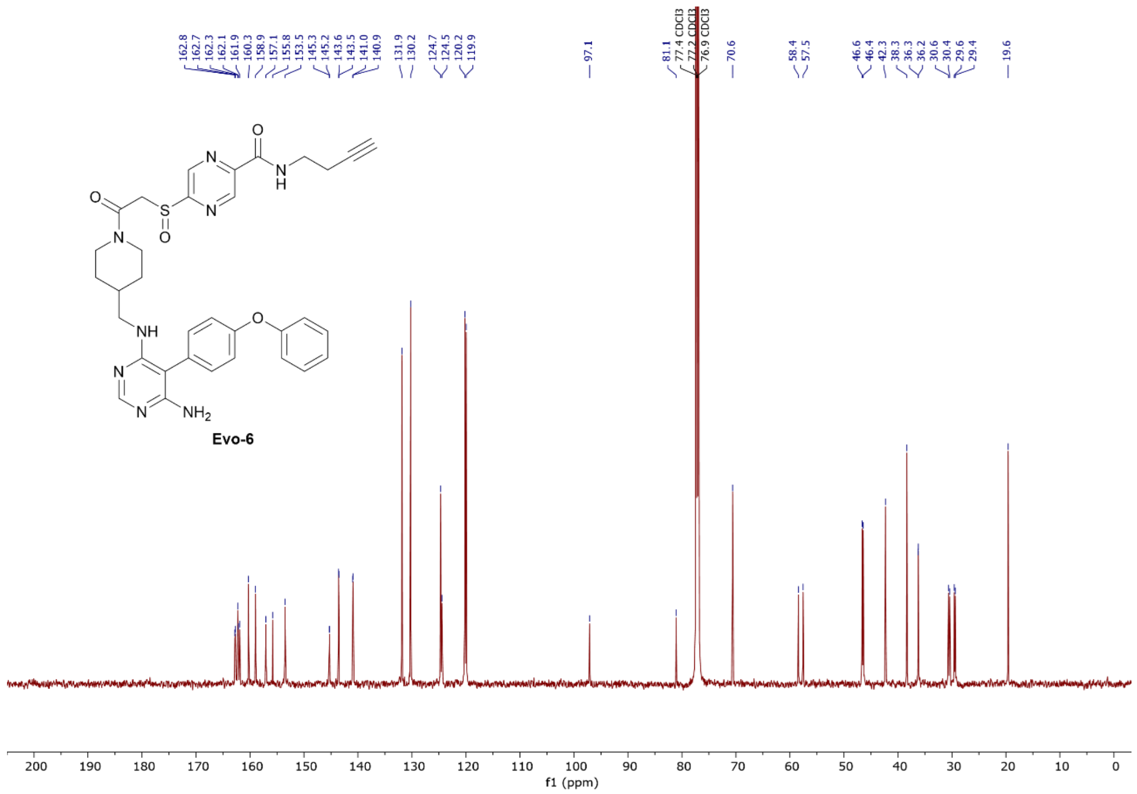

Figure S276. ^{13}C NMR spectrum of **Evo-6** (151 MHz, $CDCl_3$).

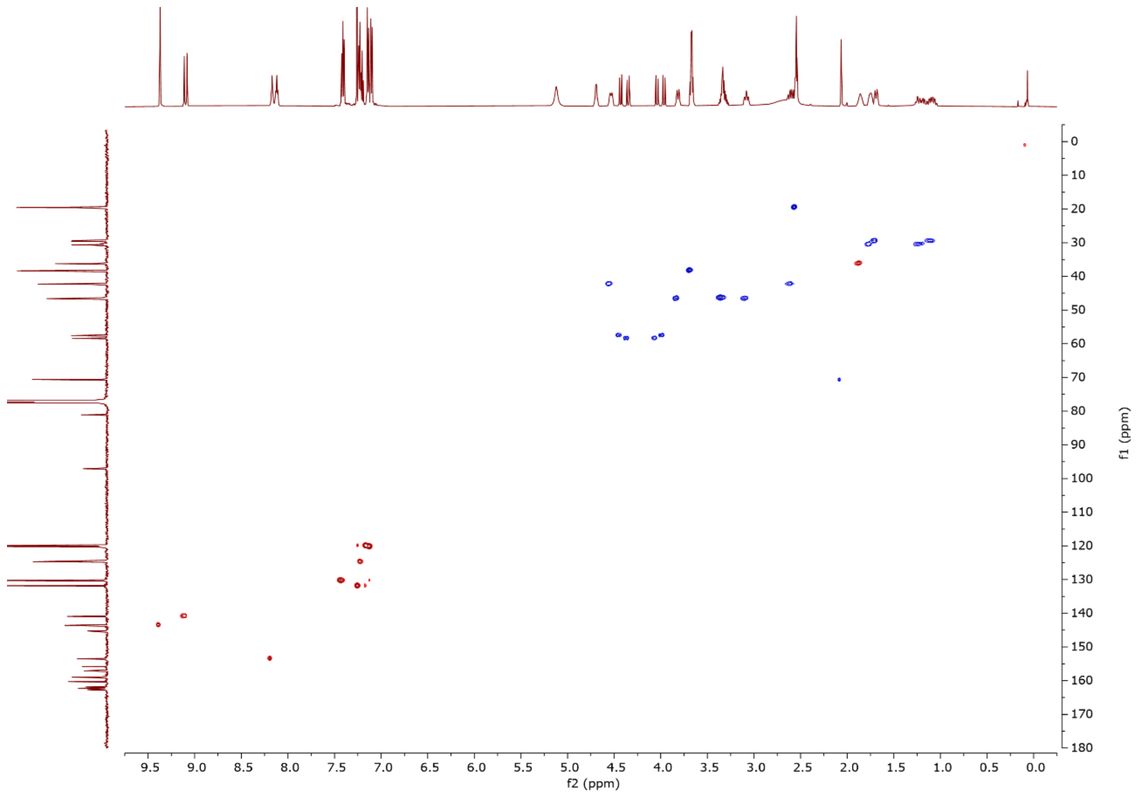

Figure S277. ^{1}H-^{13}C HSQC spectrum of **Evo-6** ($CDCl_3$).

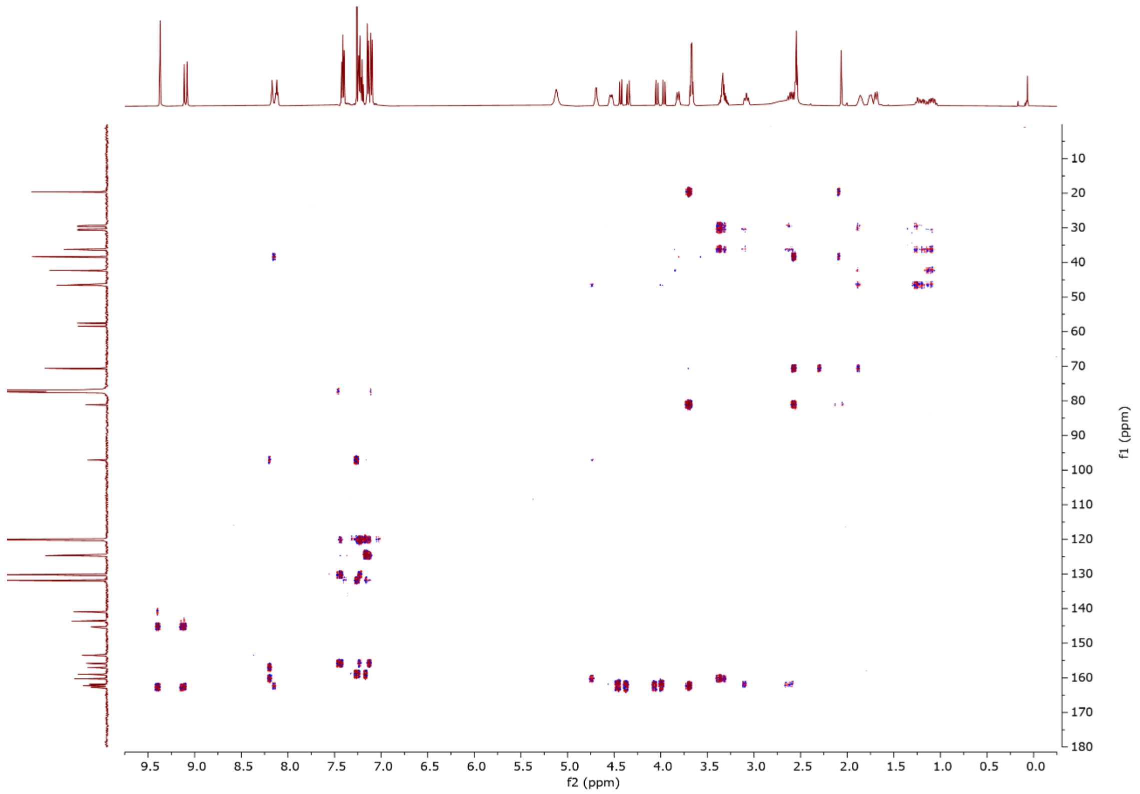

Figure S278. ^1H-^{13}C HMBC spectrum of **Evo-6** ($CDCl_3$).

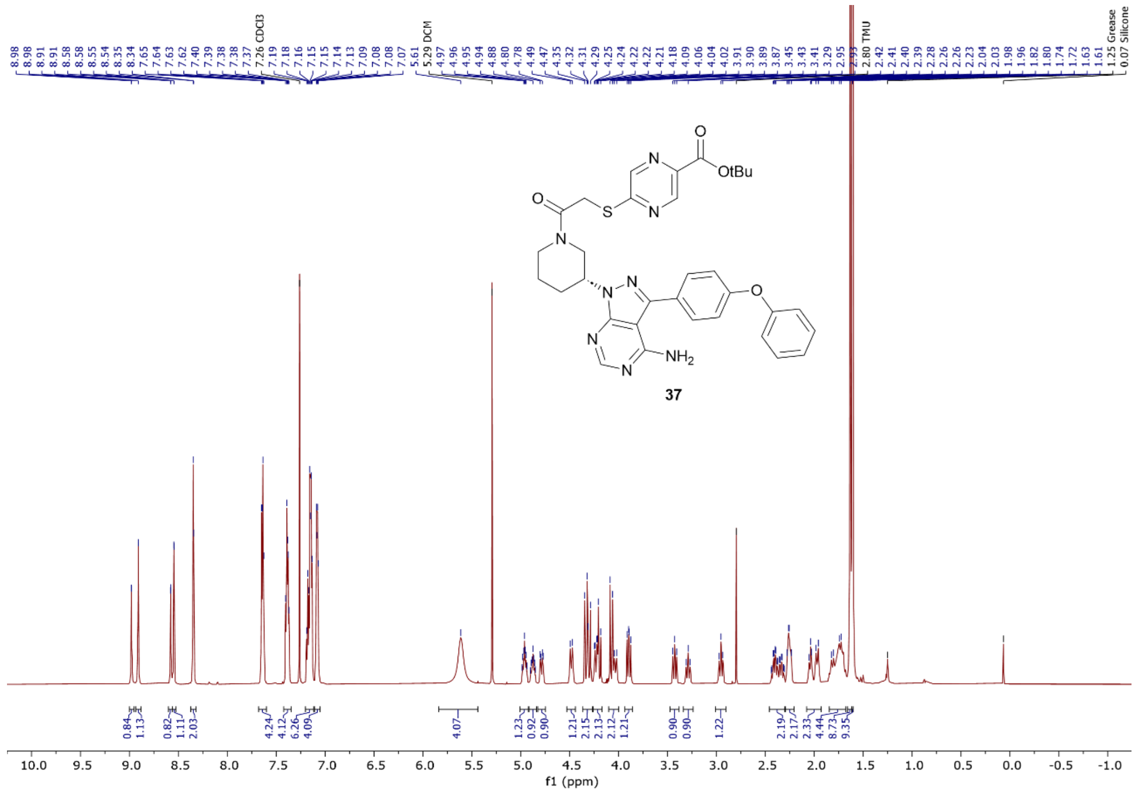

Figure S279. ^1H NMR spectrum of compound **37** (600 MHz, $CDCl_3$).

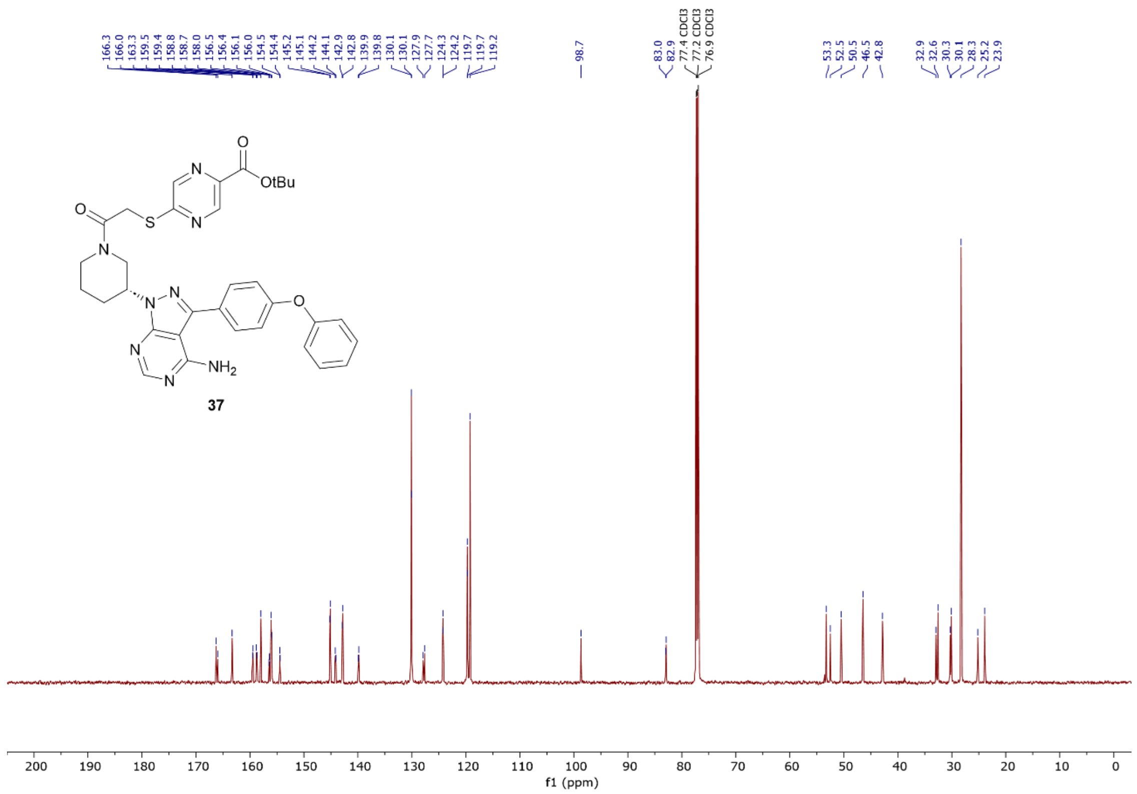

Figure S280. ^{13}C NMR spectrum of compound **37** (151 MHz, $CDCl_3$).

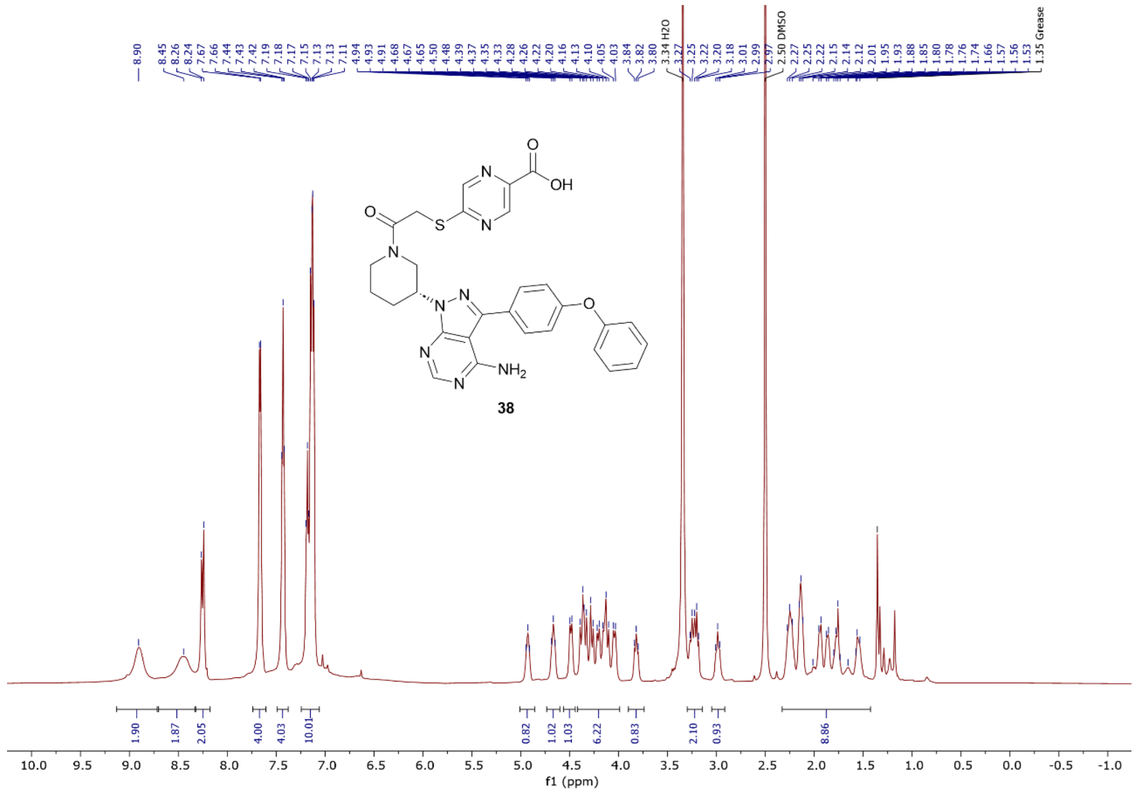

Figure S281. ^{1}H NMR spectrum of compound **38** (600 MHz, $(CD_3)_2SO$).

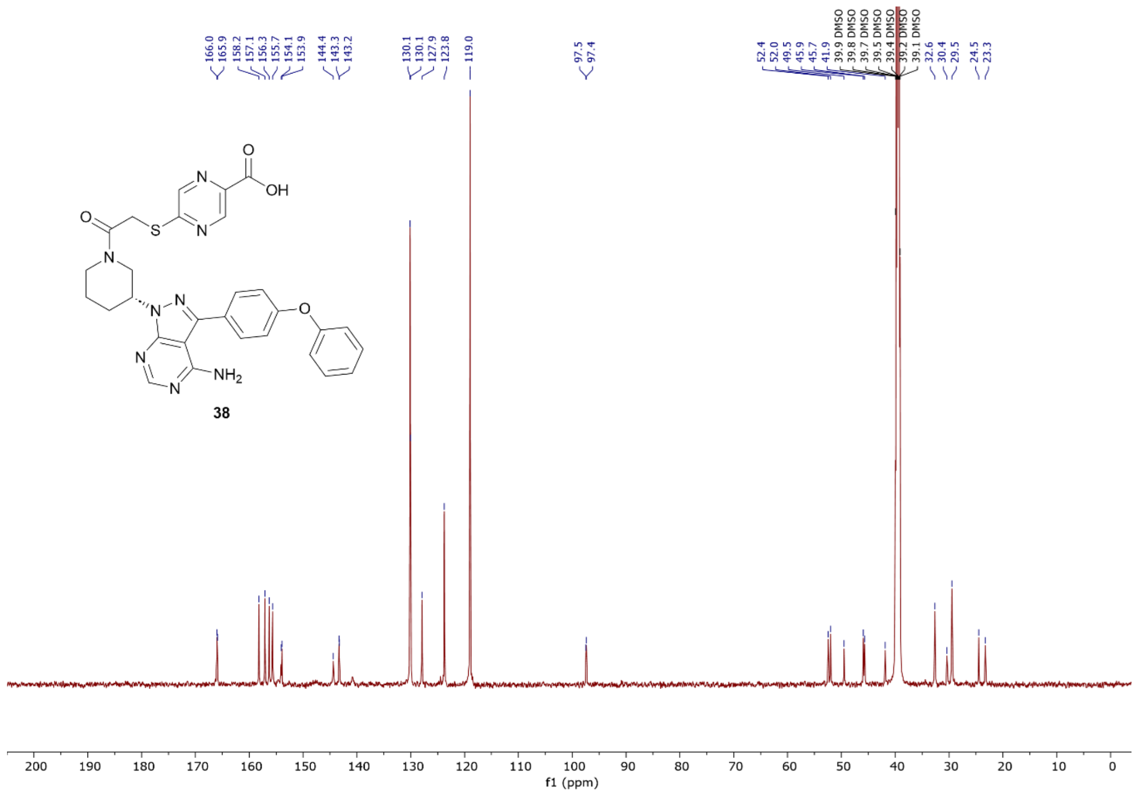

Figure S282. ^{13}C NMR spectrum of compound **38** (151 MHz, $(CD_3)_2SO$).

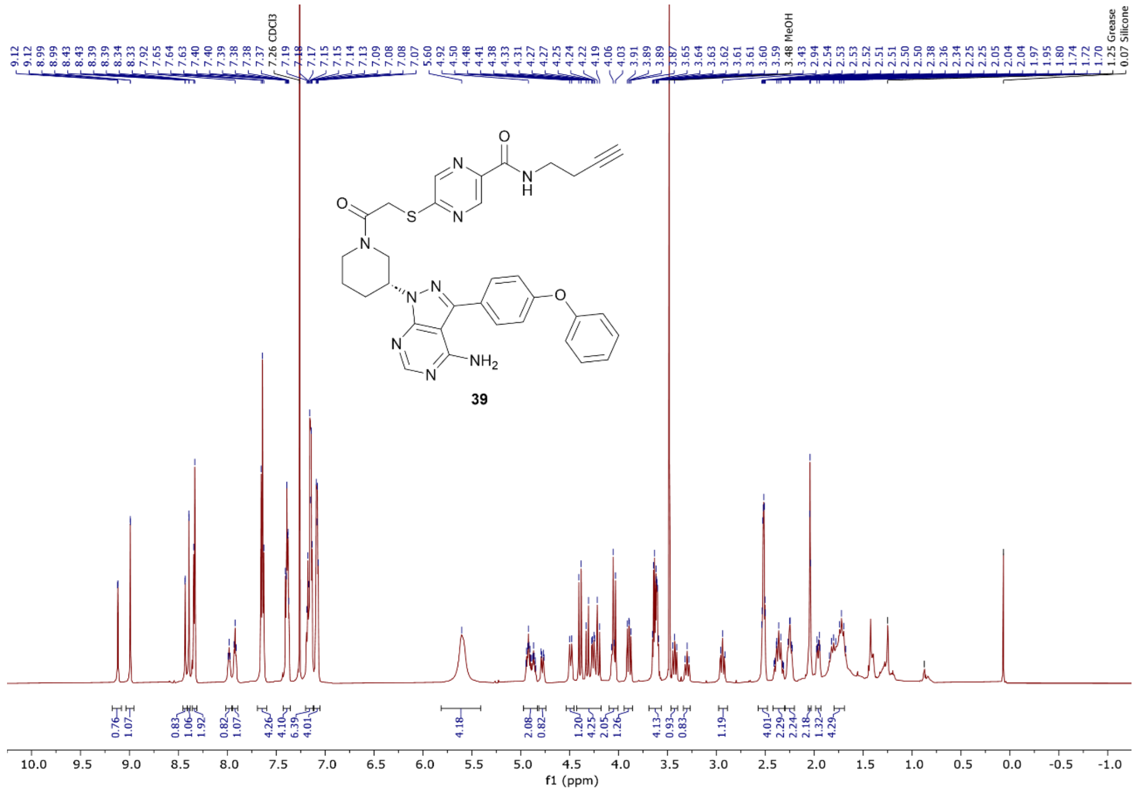

Figure S283. ^{1}H NMR spectrum of compound **39** (600 MHz, $CDCl_3$).

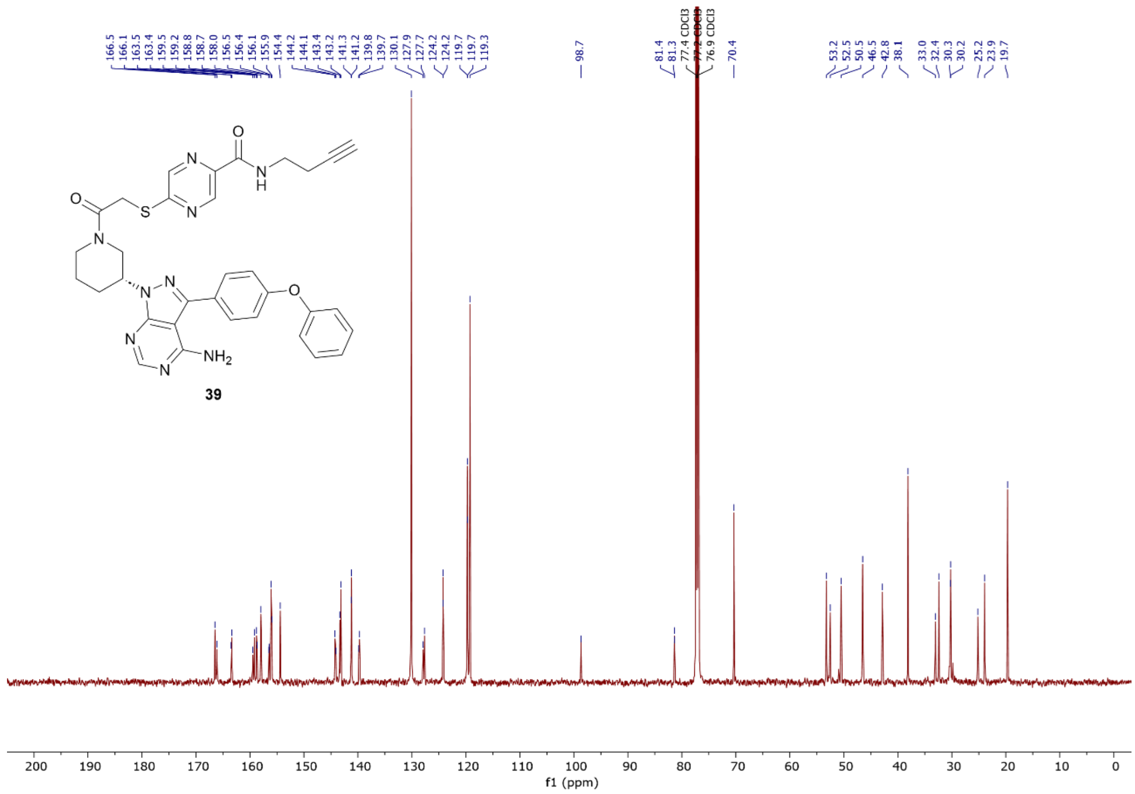

Figure S284. ^{13}C NMR spectrum of compound **39** (151 MHz, $CDCl_3$).

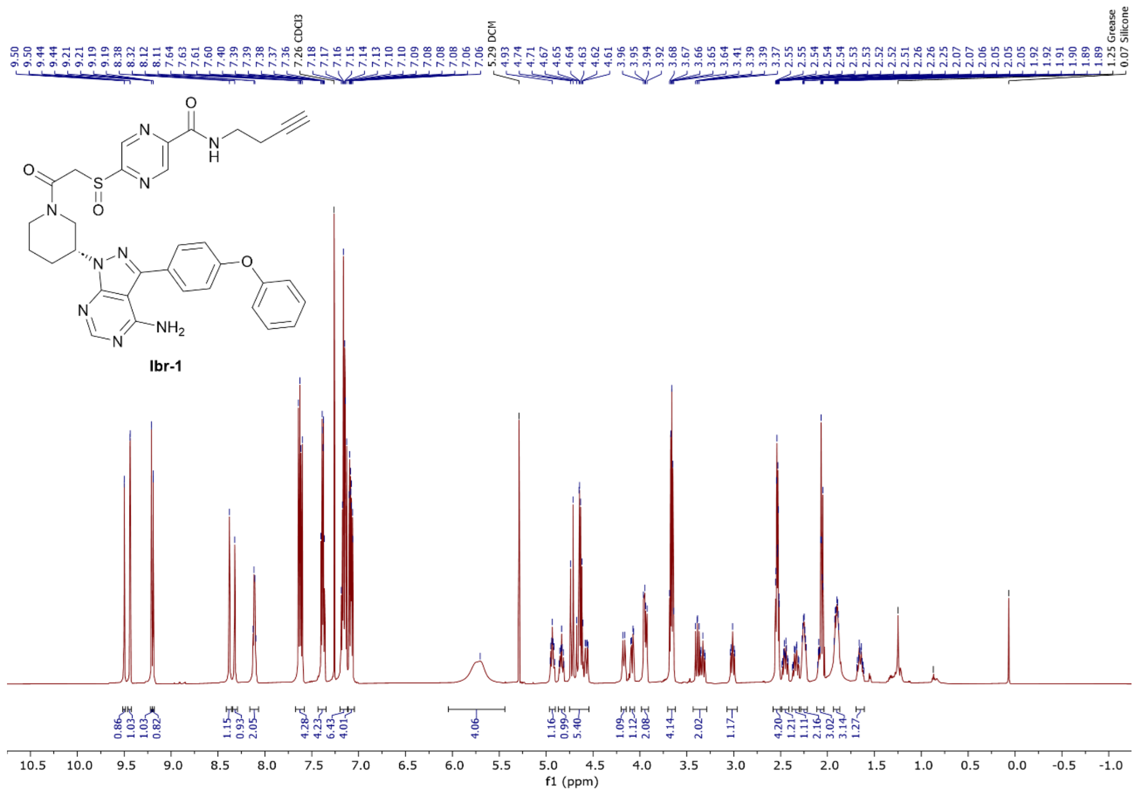

Figure S285. ^{1}H NMR spectrum of **Ibr-1** (600 MHz, $CDCl_3$).

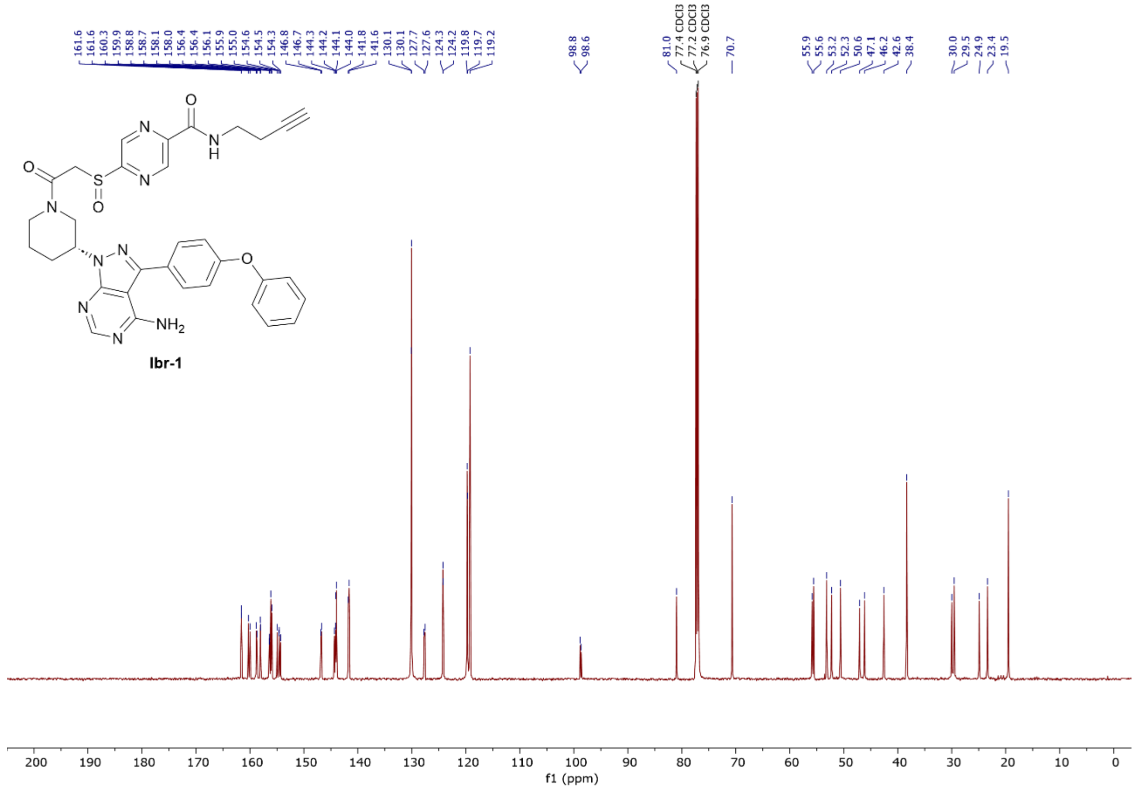

Figure S286. ^{13}C NMR spectrum of **Ibr-1** (151 MHz, $CDCl_3$).

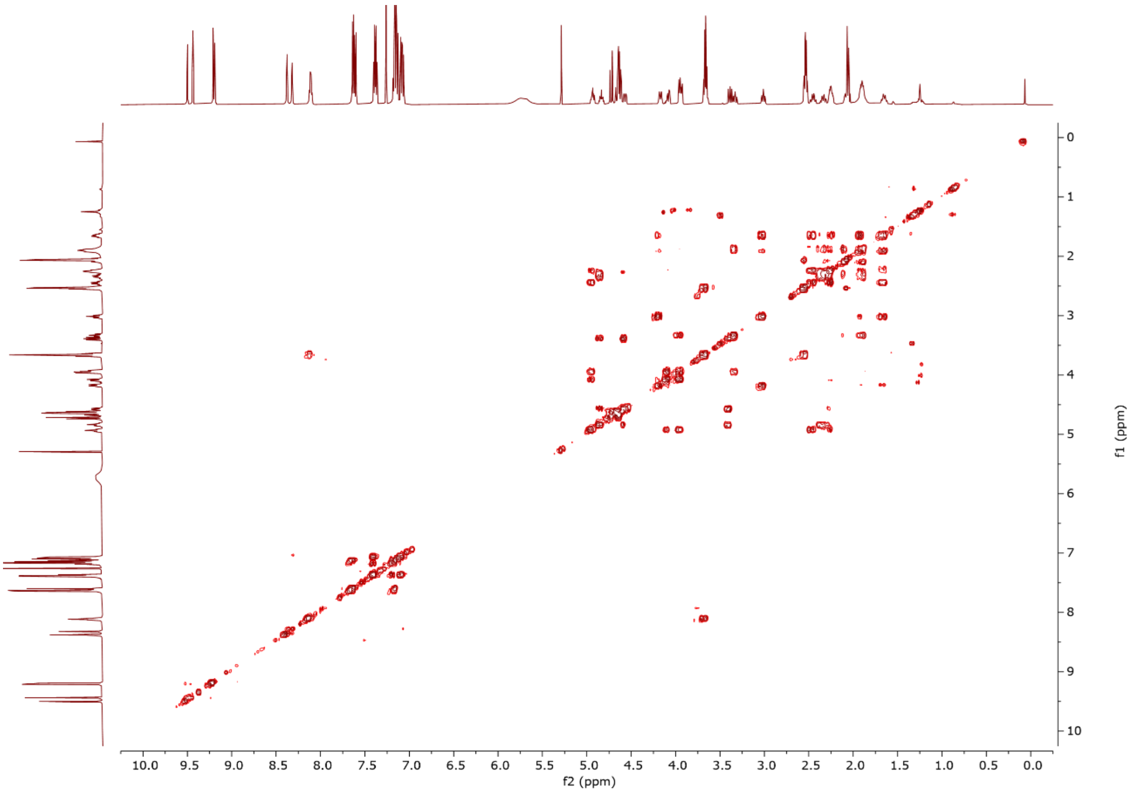

Figure S287. ^{1}H-^{1}H COSY spectrum of **Ibr-1** ($CDCl_3$).

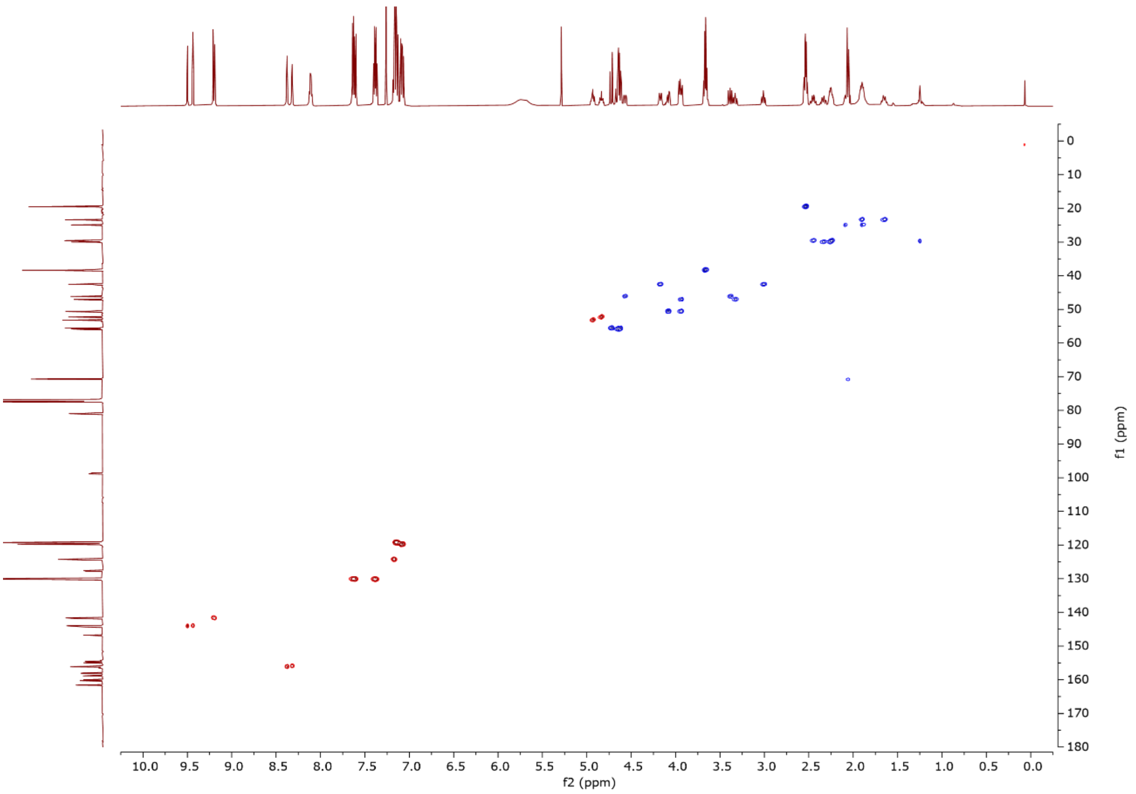

Figure S288. ^{1}H-^{13}C HSQC spectrum of **Ibr-1** ($CDCl_3$).

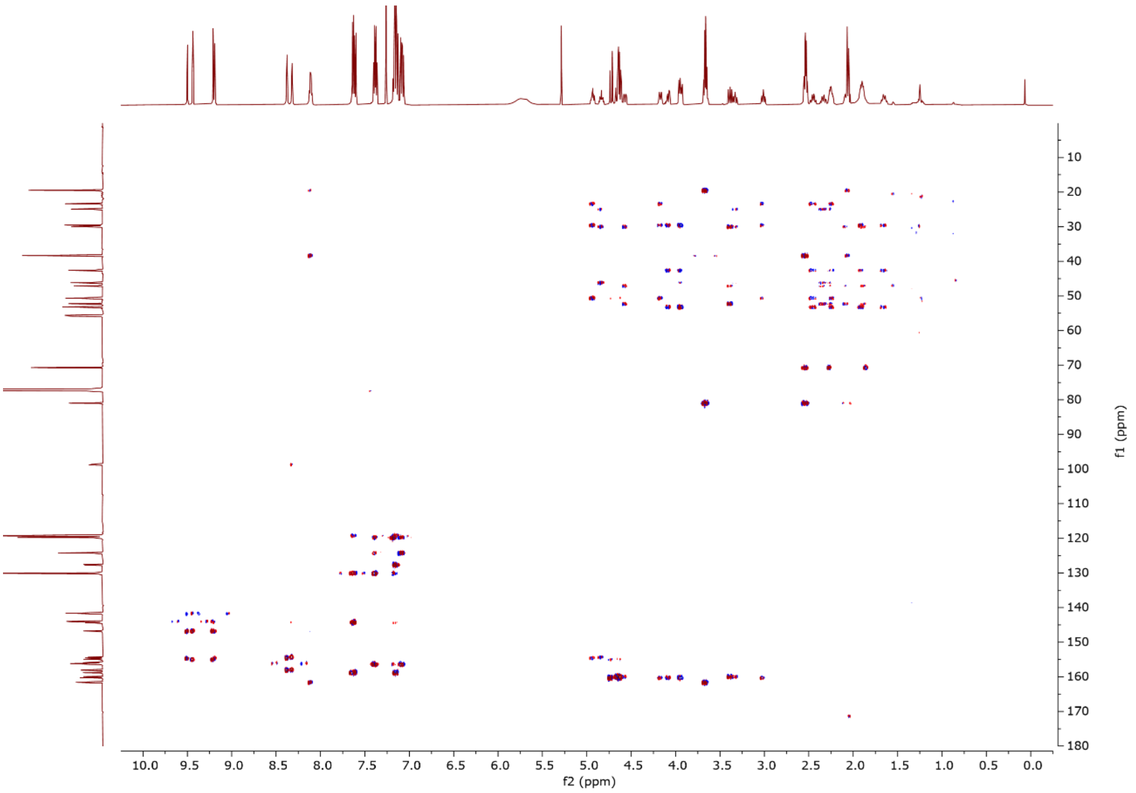

Figure S289. ^{1}H-^{13}C HMBC spectrum of **Ibr-1** ($CDCl_3$).

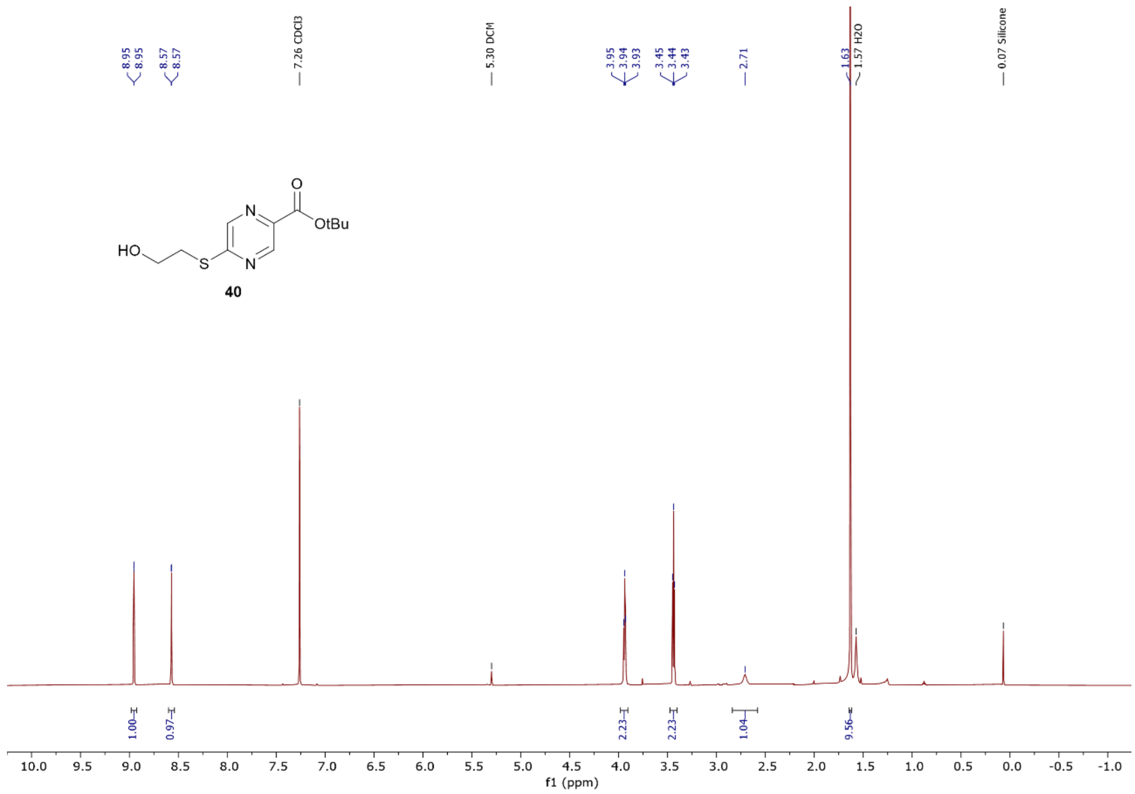

Figure S290. ^{1}H NMR spectrum of compound **40** (600 MHz, $CDCl_3$).

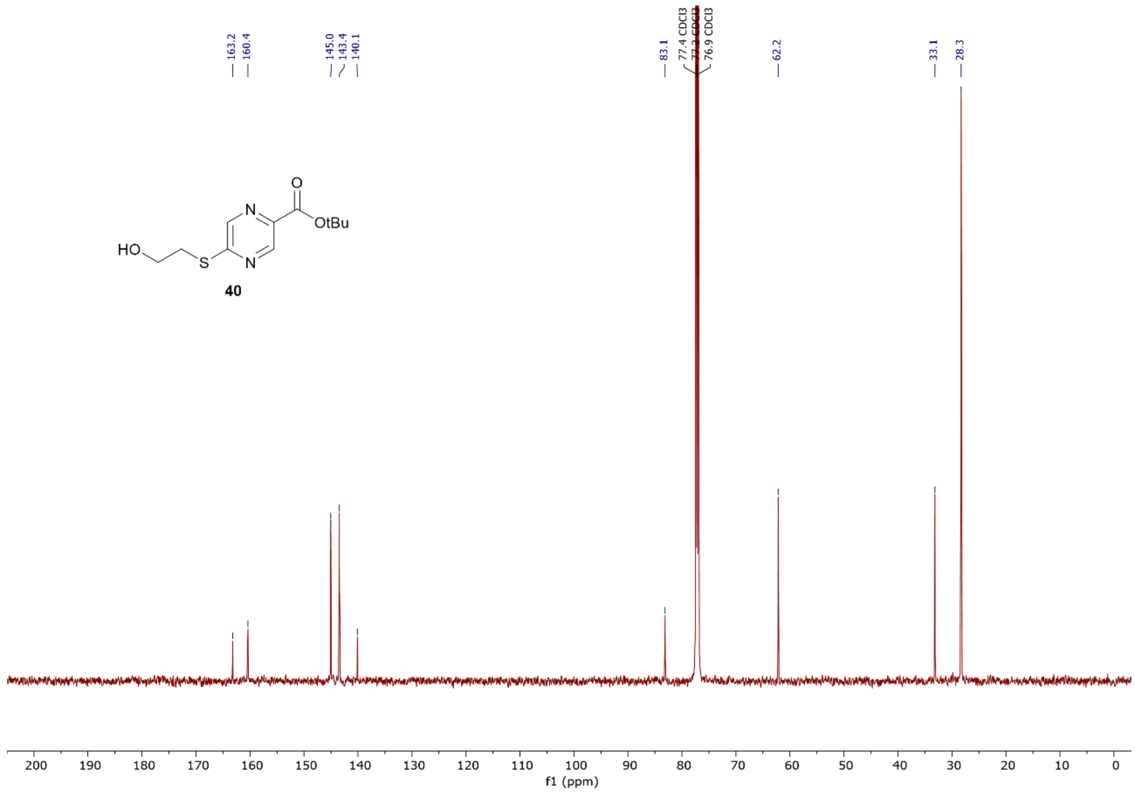

Figure S291. ^{13}C NMR spectrum of compound **40** (151 MHz, $CDCl_3$).

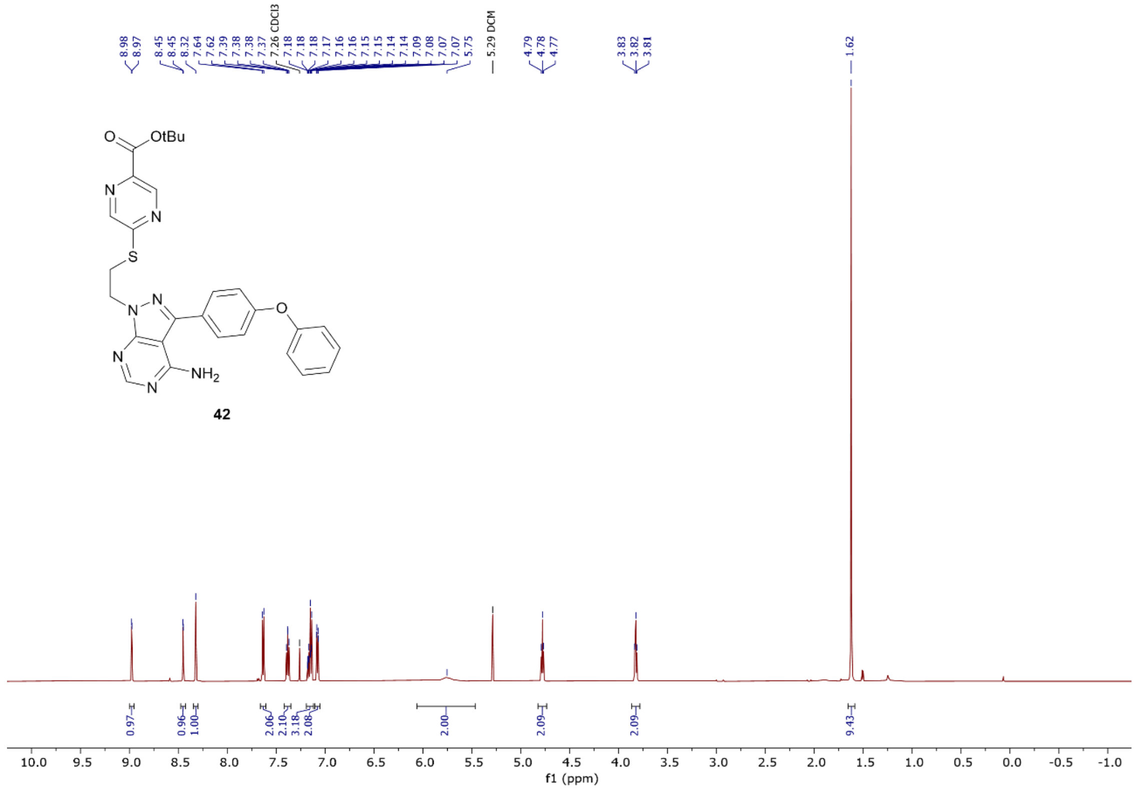

Figure S292. ^{1}H NMR spectrum of compound **42** (600 MHz, $CDCl_3$).

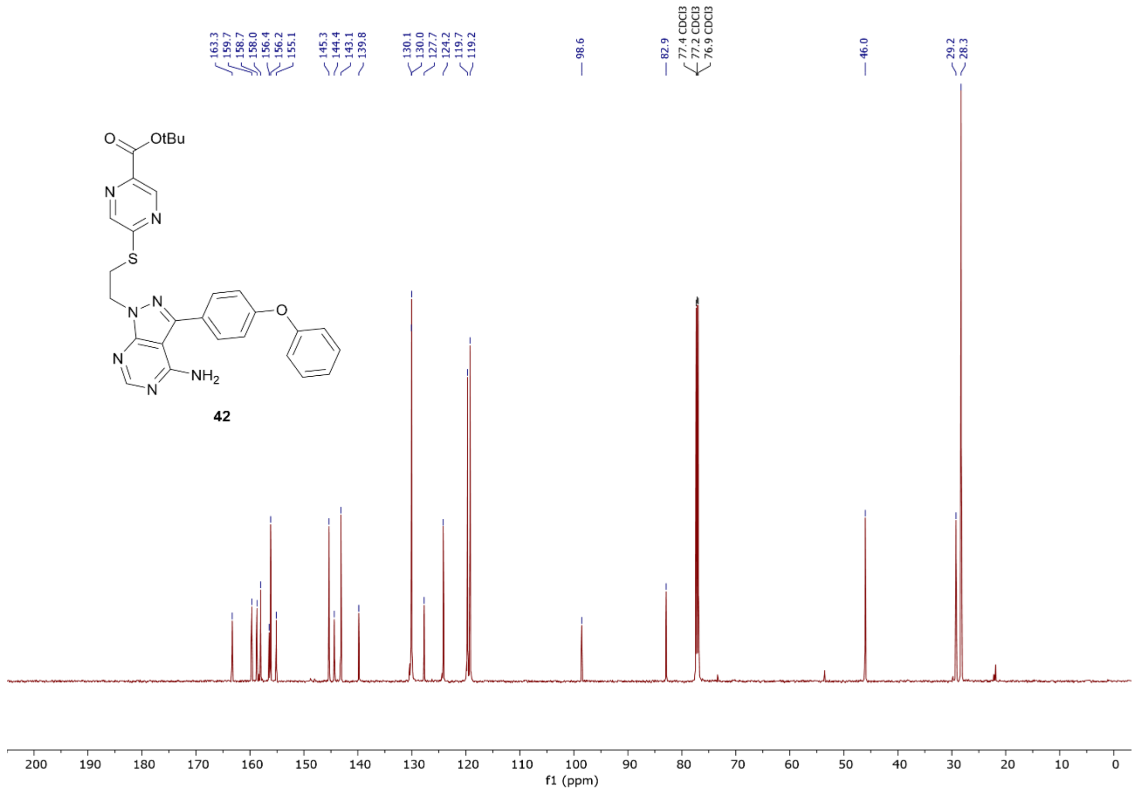

Figure S293. ^{13}C NMR spectrum of compound **42** (151 MHz, $CDCl_3$).

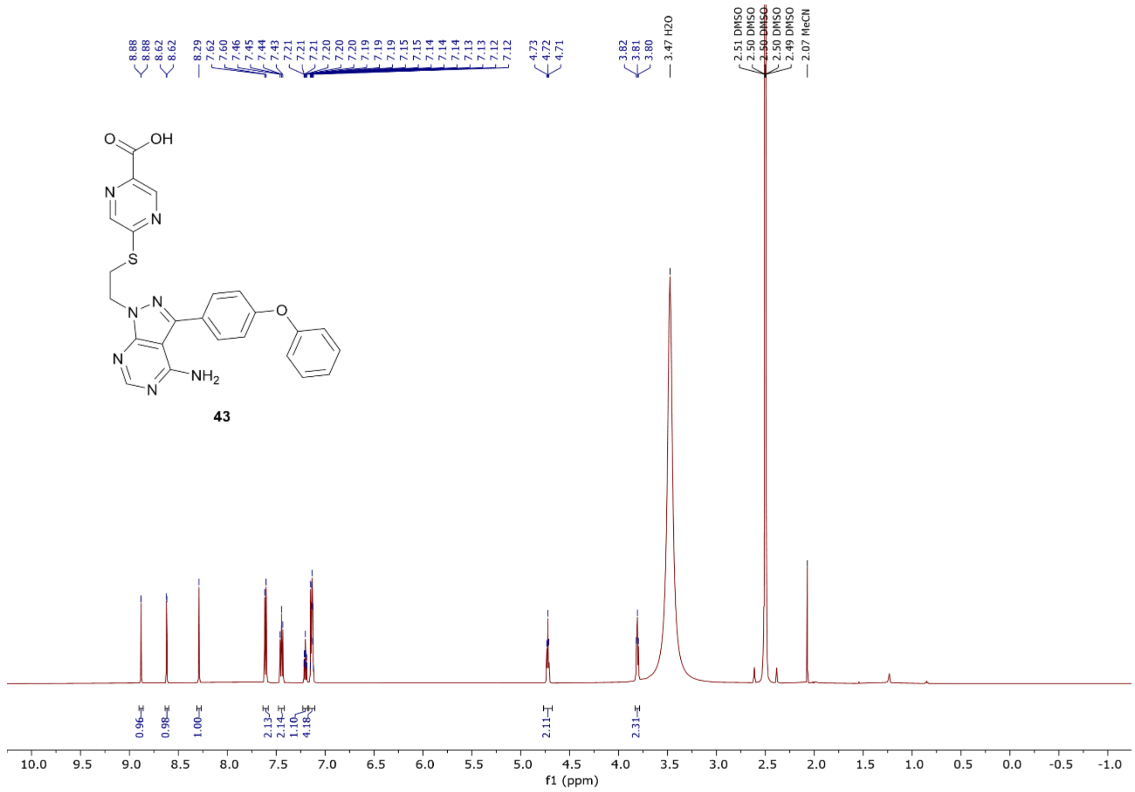

Figure S294. ^{1}H NMR spectrum of compound **43** (600 MHz, $(CD_3)_2SO$).

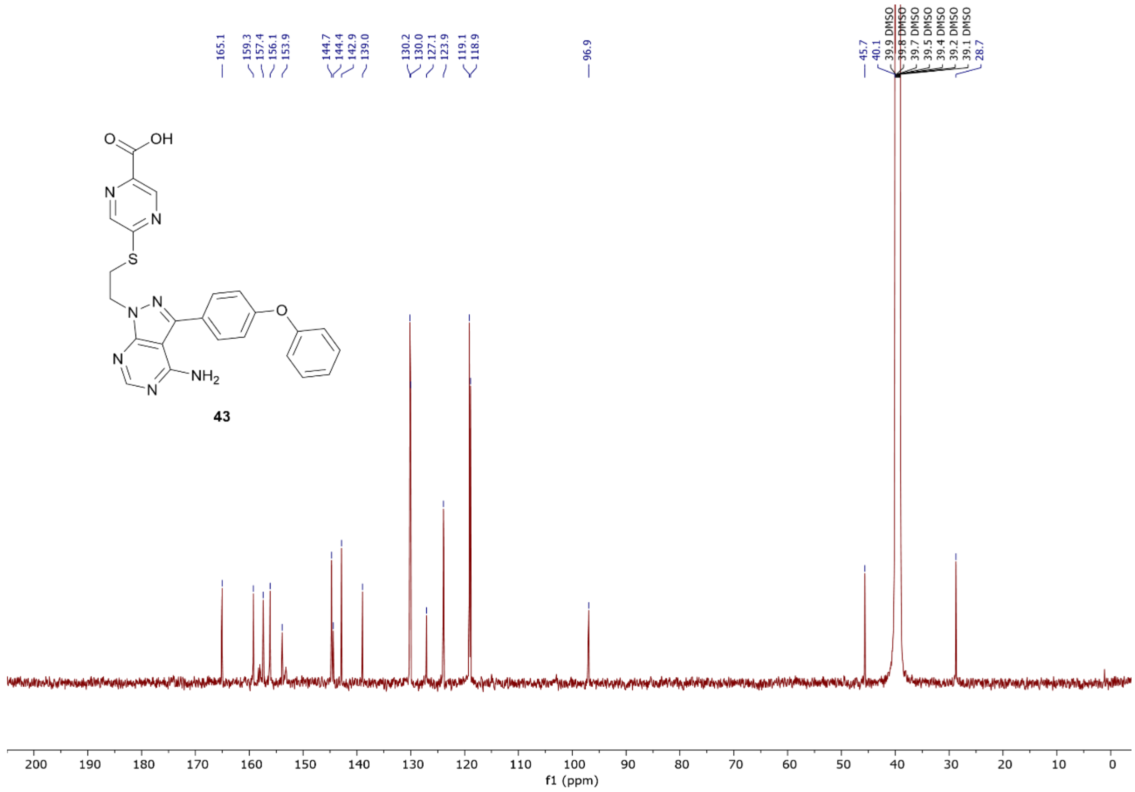

Figure S295. ^{13}C NMR spectrum of compound **43** (151 MHz, $(CD_3)_2SO$).

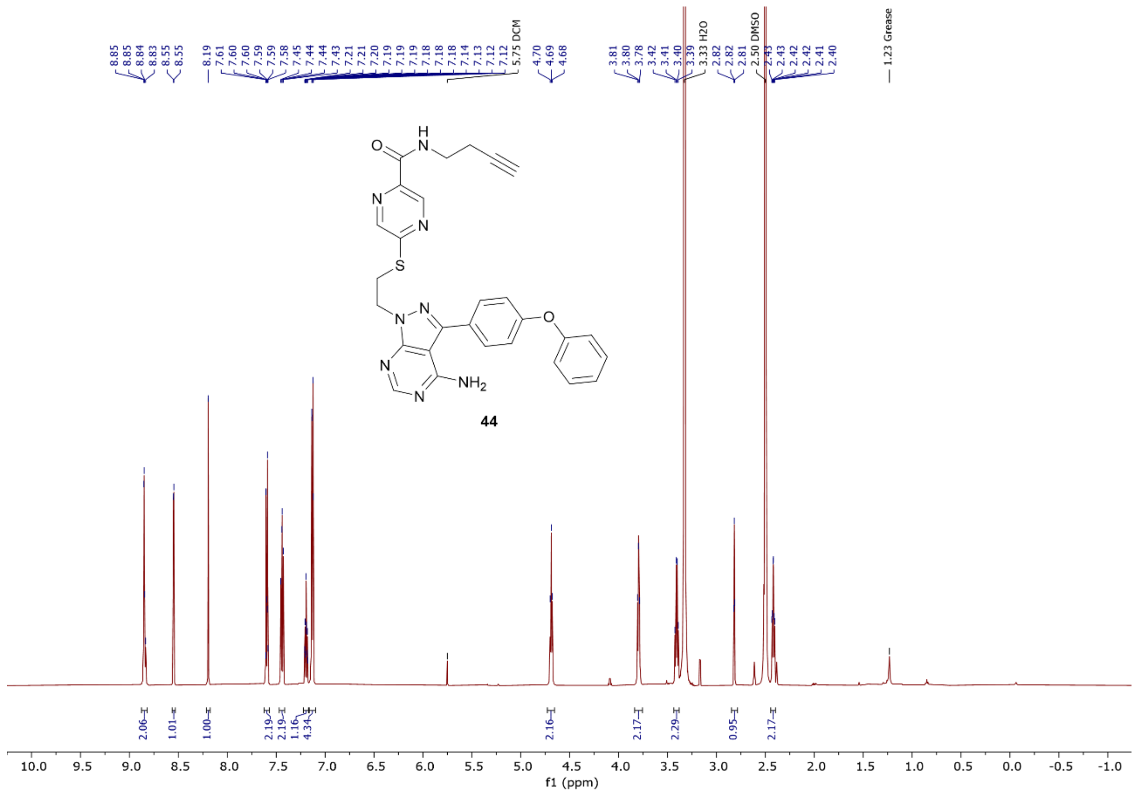

Figure S296. ^{1}H NMR spectrum of compound **44** (600 MHz, $(CD_3)_2SO$).

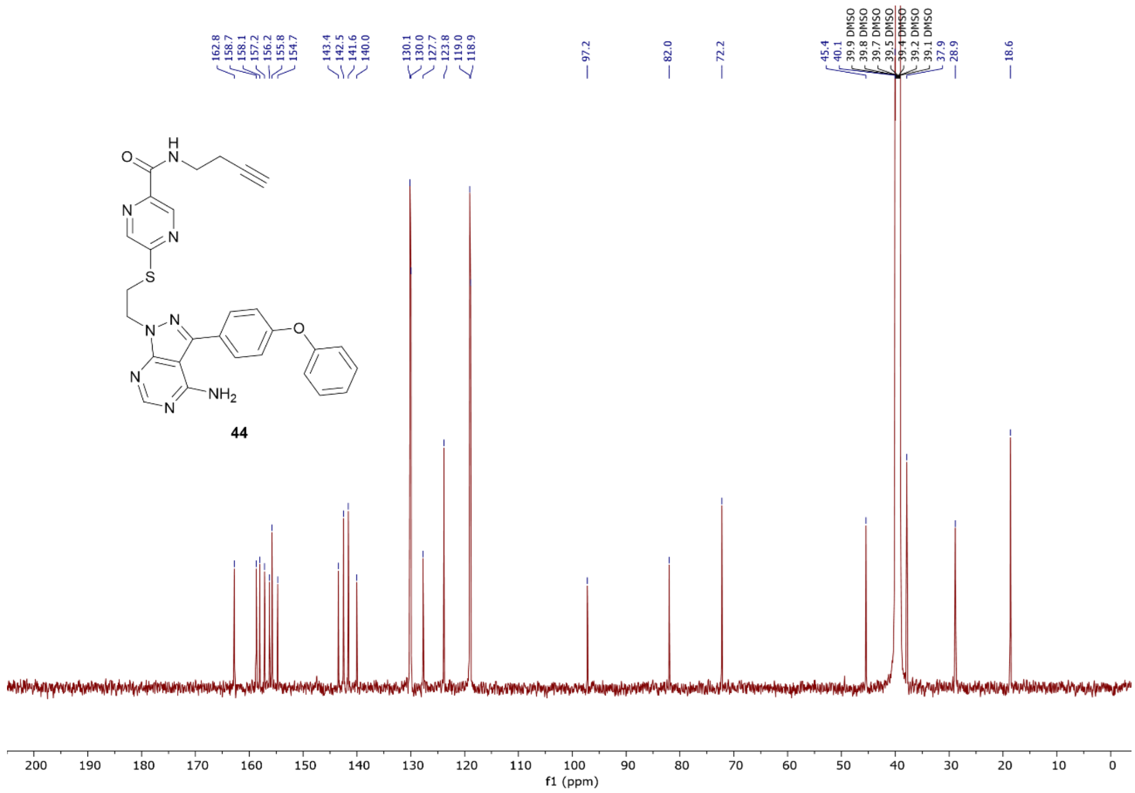

Figure S297. ^{13}C NMR spectrum of compound **44** (151 MHz, $(CD_3)_2SO$).

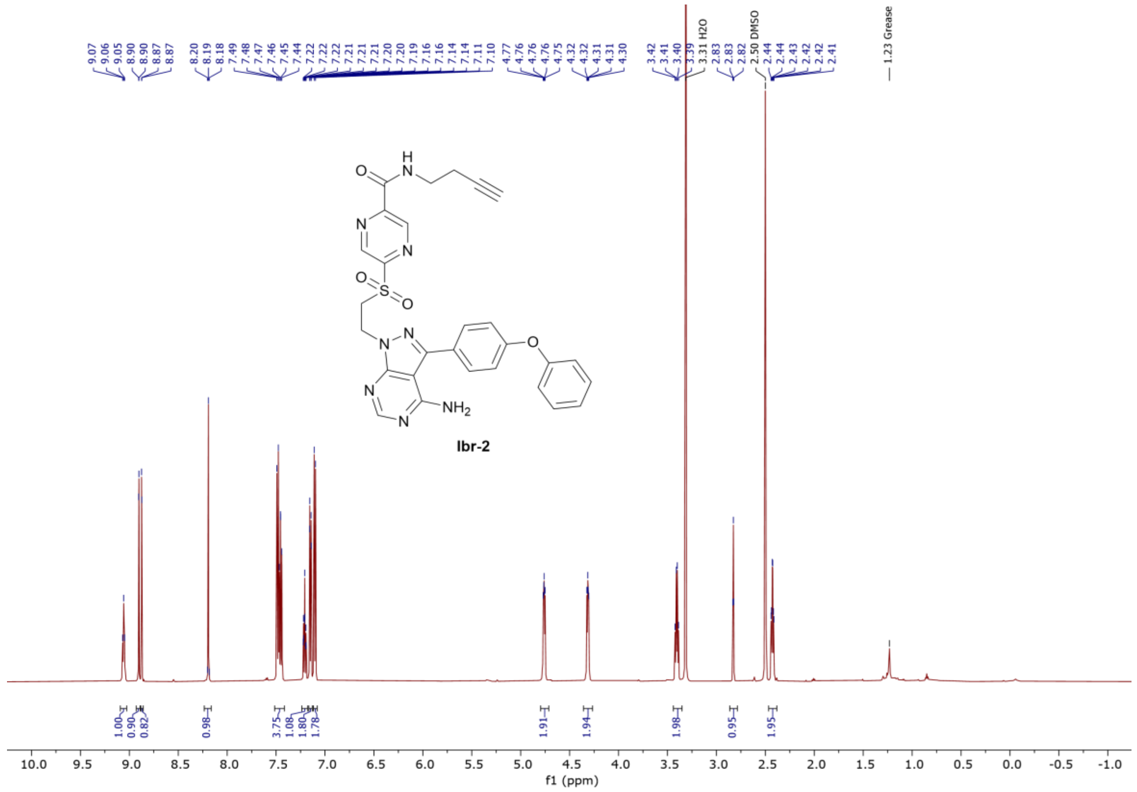

Figure S298. ^{1}H NMR spectrum of compound **Ibr-2** (600 MHz, $(CD_3)_2SO$).

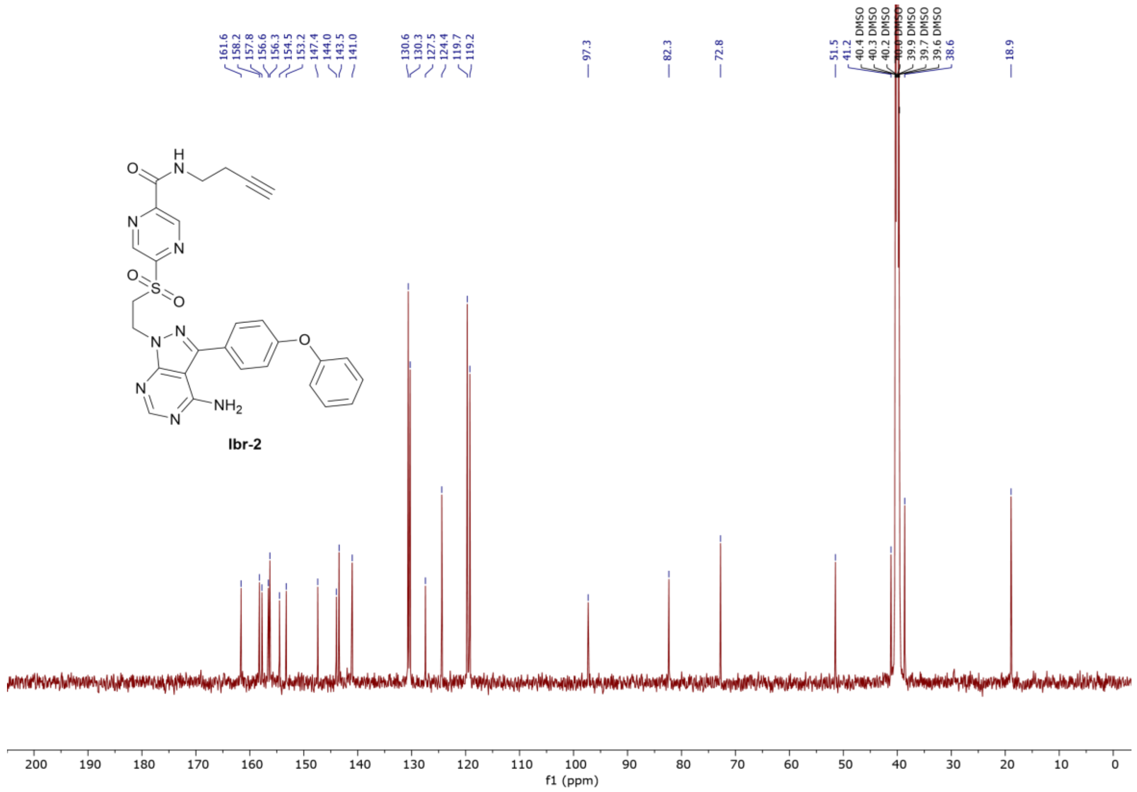

Figure S299. ^{13}C NMR spectrum of compound **Ibr-2** (151 MHz, $(CD_3)_2SO$).

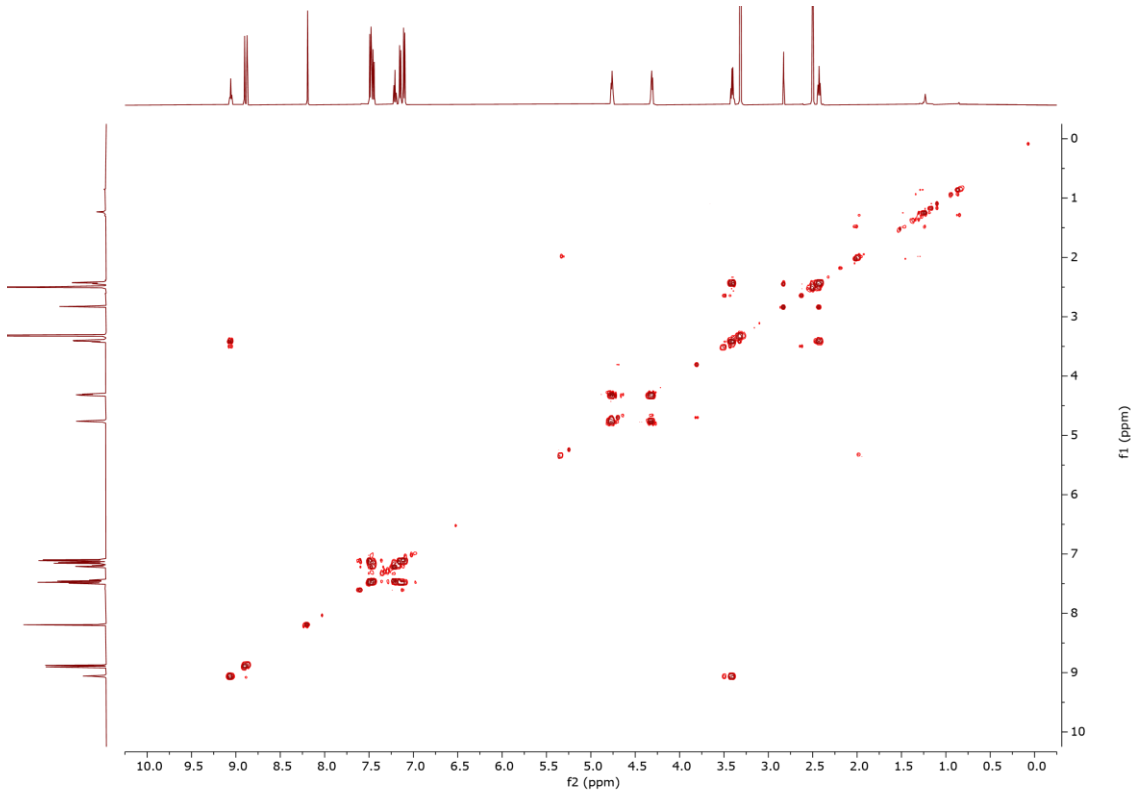

Figure S300. ^{1}H-^{1}H COSY spectrum of **Ibr-2** [$(CD_3)_2SO$].

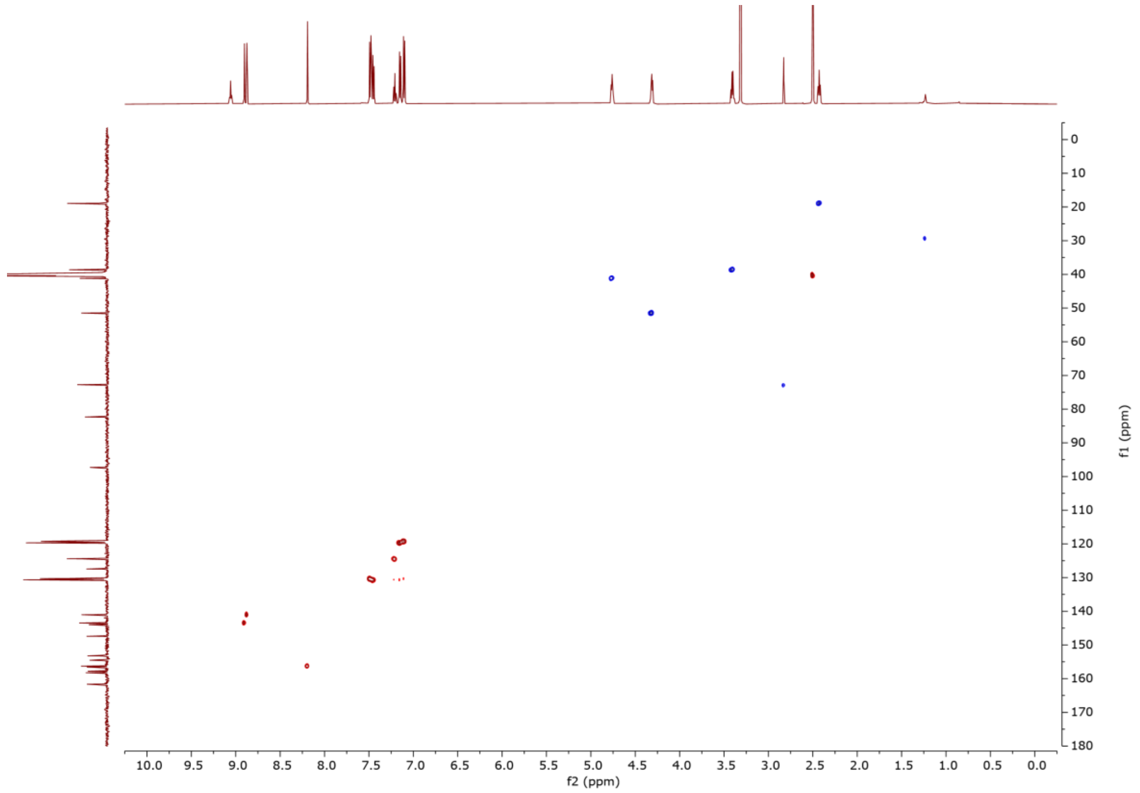

Figure S301. ^{1}H-^{13}C HSQC spectrum of **Ibr-2** [$(CD_3)_2SO$].

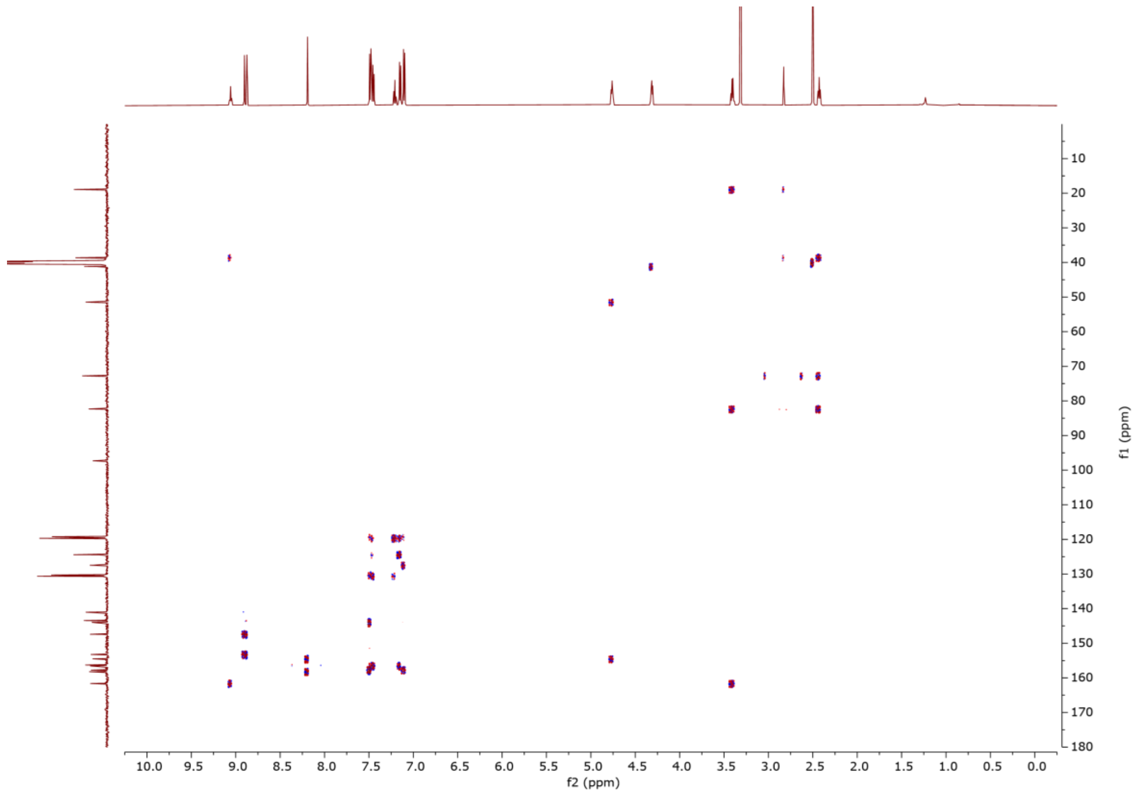

Figure S302. ^{1}H-^{13}C HMBC spectrum of **Ibr-2** [$(CD_3)_2SO$].

6 LC-MS Traces of Reactivity Assays with NAC and GSH

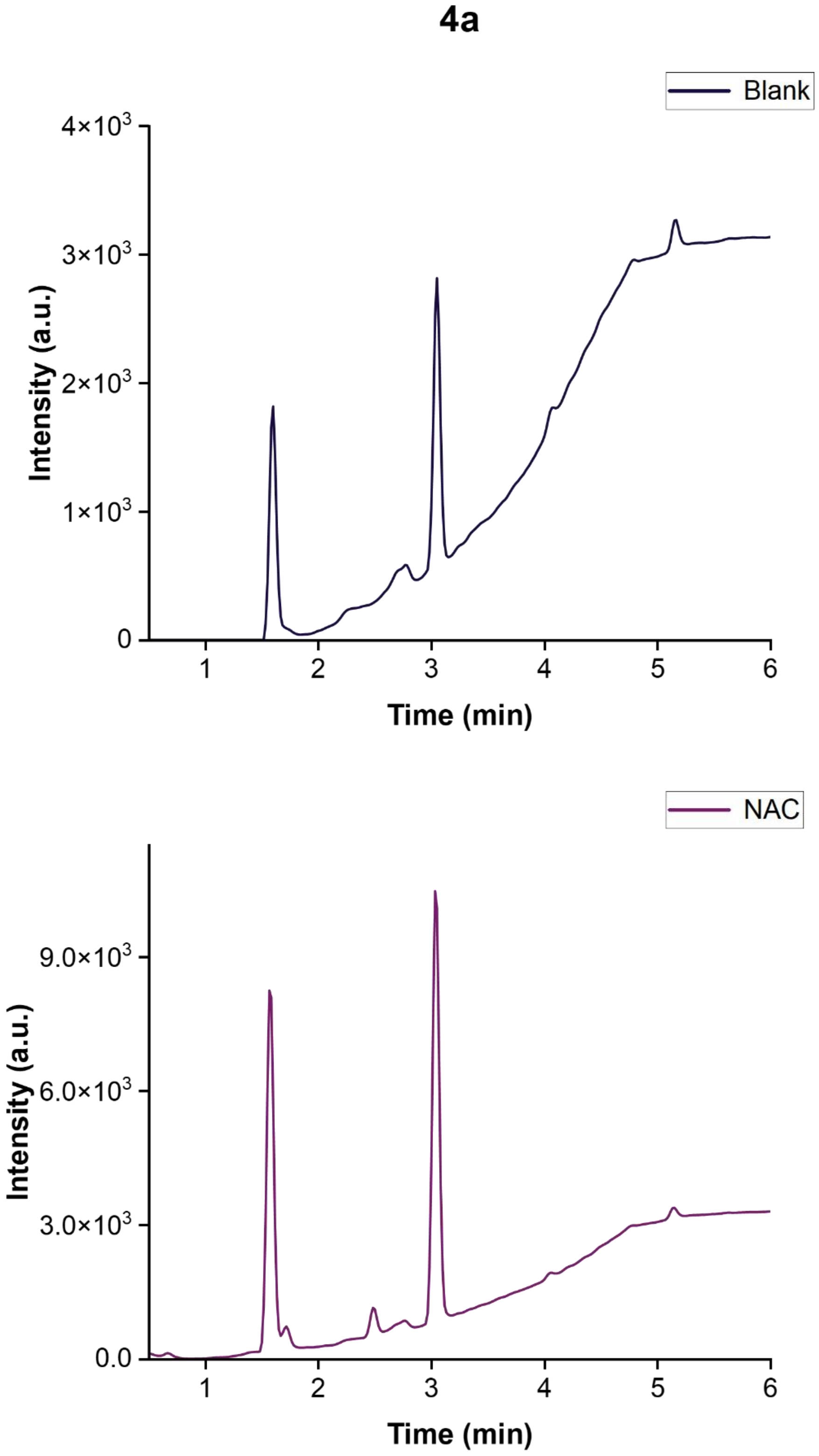

Figure S303. UV spectra (sum of absorption at 230 nm and 254 nm) of the LC-MS analysis of 100 µM of **4a** incubated with 5 mM NAC for 16 h in PBS buffer pH 7.4 at room temperature. The blank sample is shown in black while the reaction sample in magenta. Internal standard: Retention time (RT) = 1.5 min; **4a**: RT = 3.09 min.

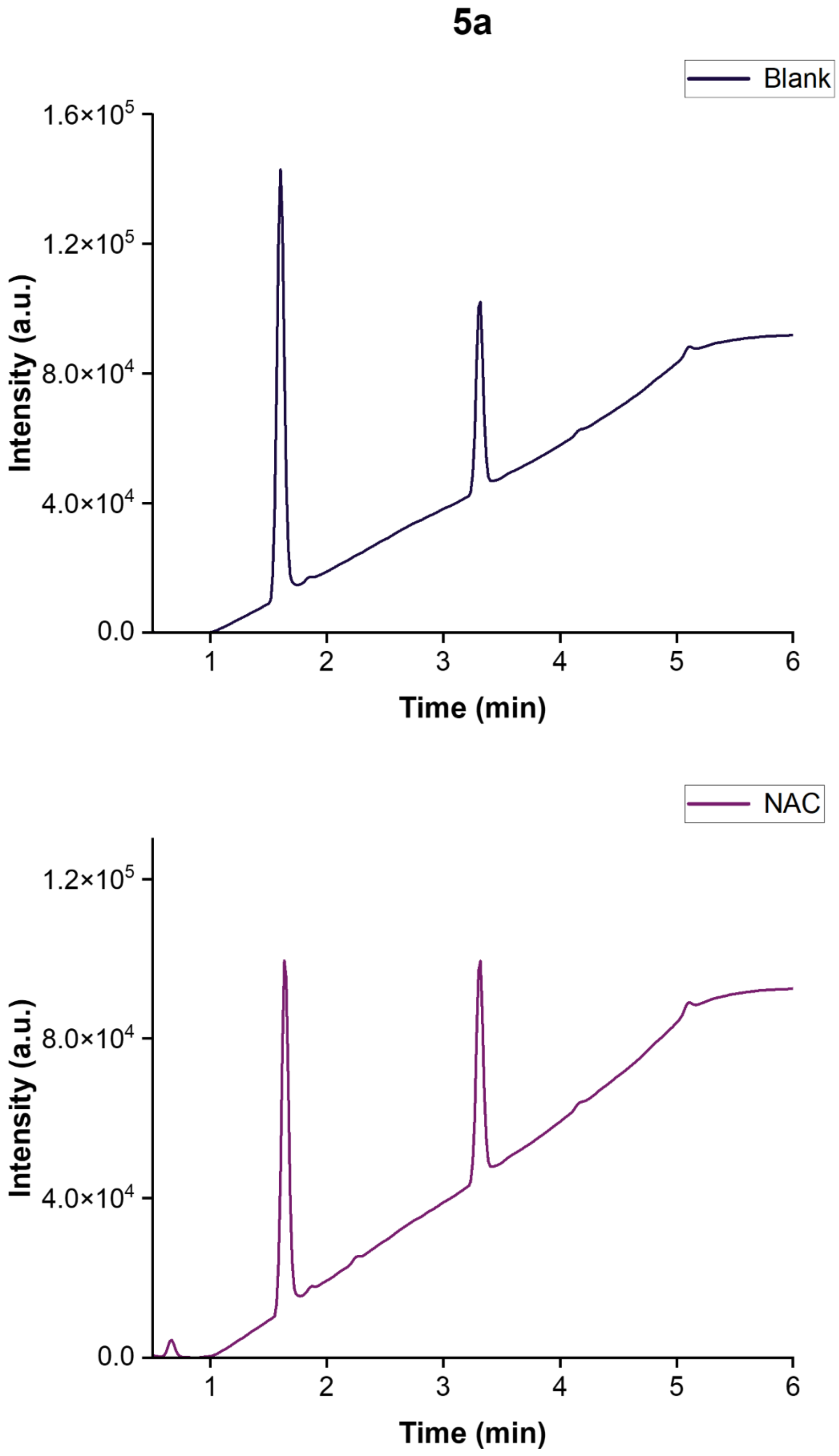

Figure S304. UV spectra (sum of absorption at 230 nm and 254 nm) of the LC-MS analysis of 100 µM of **5a** incubated with 5 mM NAC for 16 h in PBS buffer pH 7.4 at room temperature. The blank sample is shown in black while the reaction sample in magenta. Internal standard: RT = 1.5 min; **5a**: RT = 3.3 min.

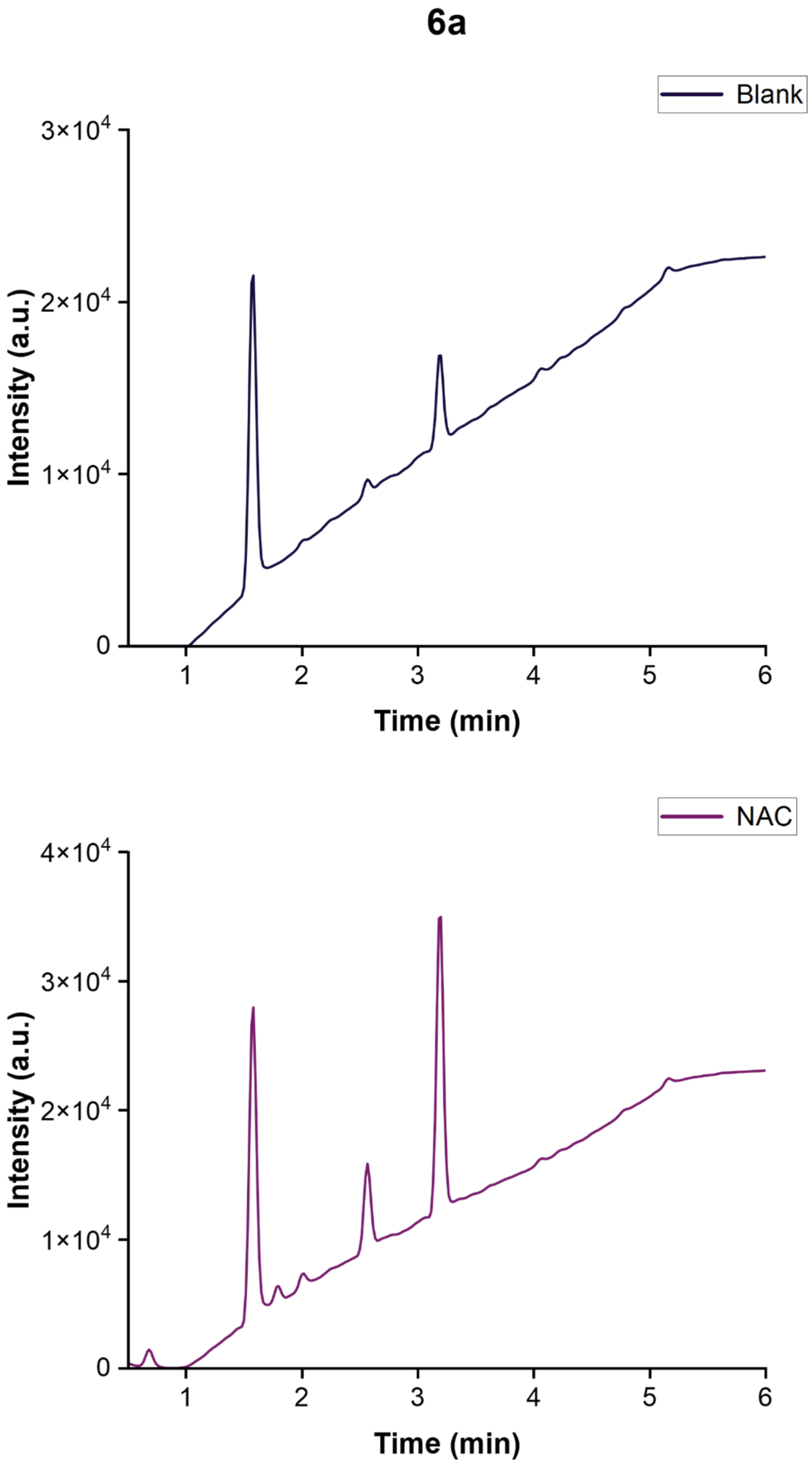

Figure S305. UV spectra (sum of absorption at 230 nm and 254 nm) of the LC-MS analysis of 100 µM of **6a** incubated with 5 mM NAC for 16 h in PBS buffer pH 7.4 at room temperature. The blank sample is shown in black while the reaction in magenta. Internal standard: RT = 1.5 min; **6a**: RT = 3.2 min.

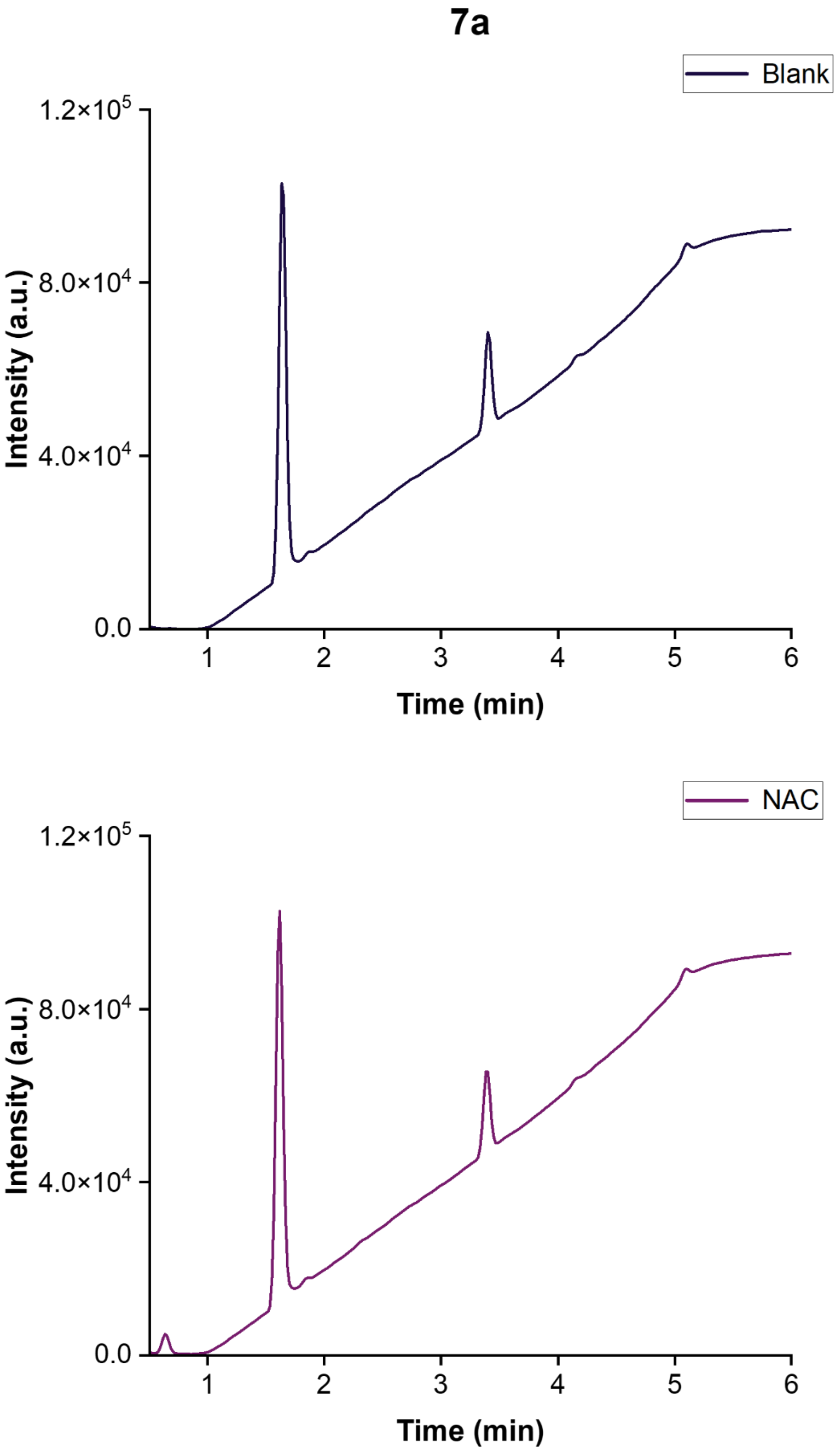

Figure S306. UV spectra (sum of absorption at 230 nm and 254 nm) of the LC-MS analysis of 100 µM of **7a** incubated with 5 mM NAC for 16 h in PBS buffer pH 7.4 at room temperature. The blank sample is shown in black while the reaction in magenta. Internal standard: RT = 1.5 min; **7a**: RT = 3.38 min.

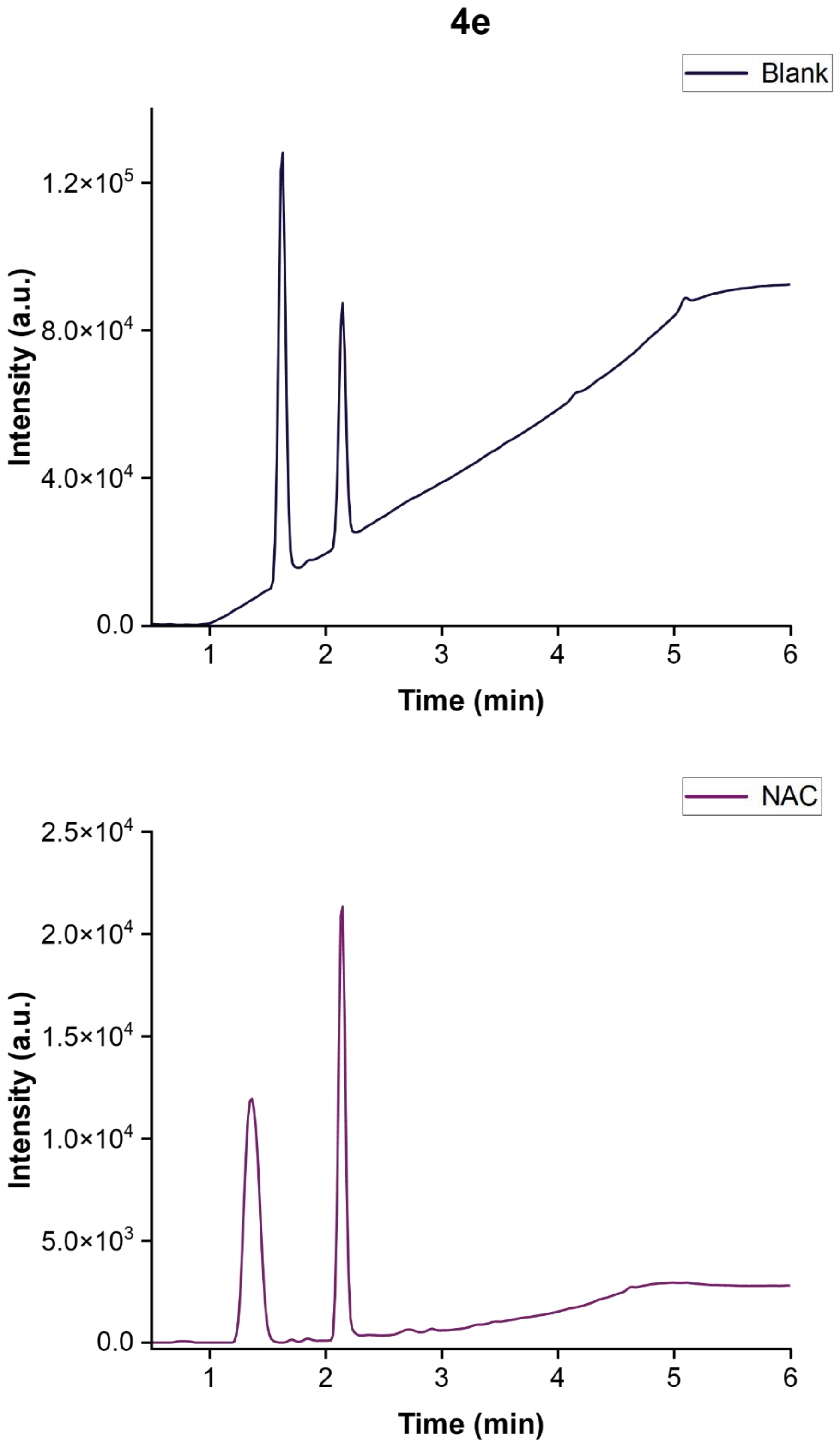

Figure S307. UV spectra (sum of absorption at 230 nm and 254 nm) of the LC-MS analysis of 100 µM of **4e** incubated with 5 mM NAC for 16 h in PBS buffer pH 7.4 at room temperature. The blank sample is shown in black while the reaction in magenta. Internal standard: RT = 1.5 min; **4e**: RT = 2.15 min.

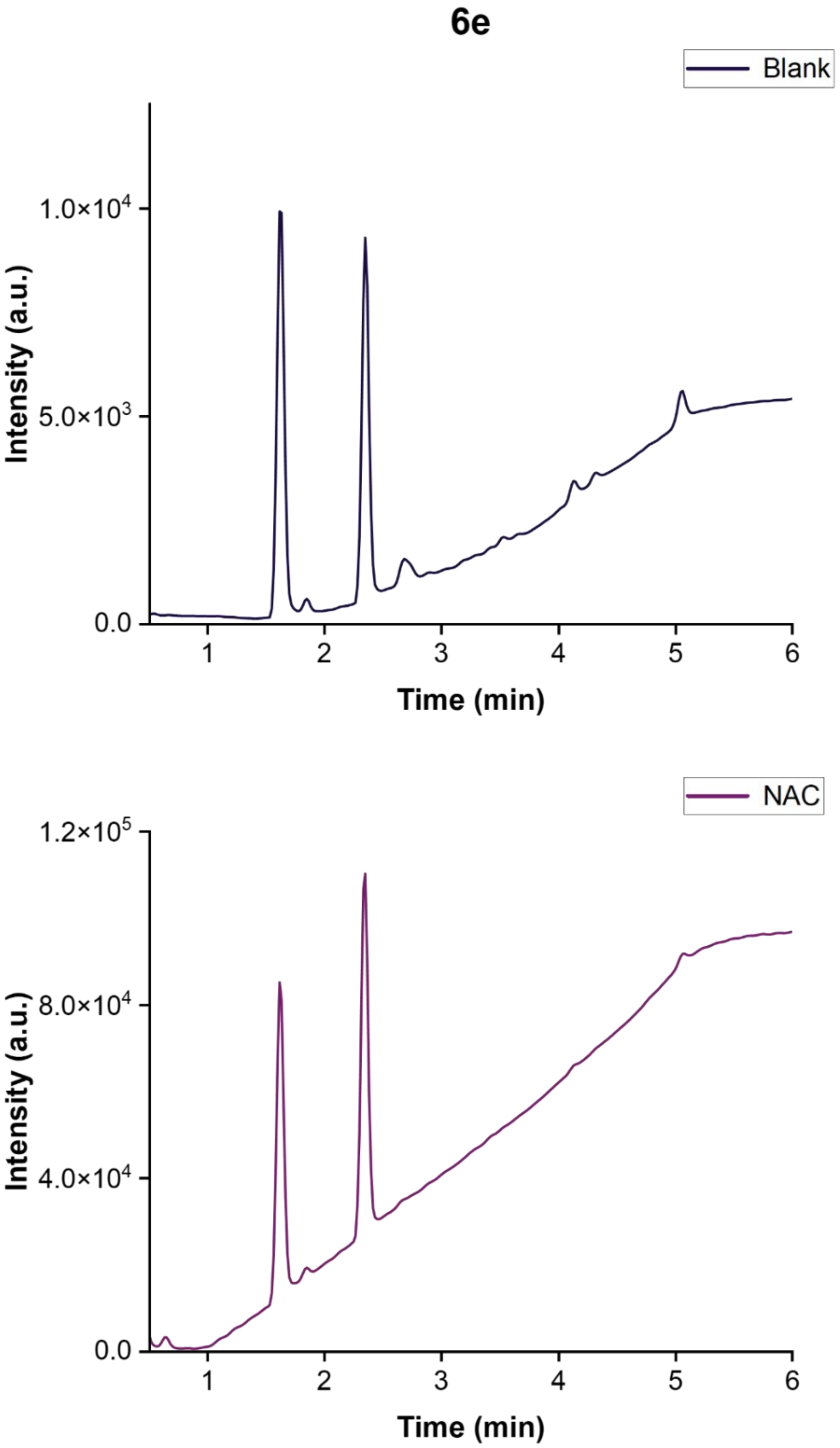

Figure S308. UV spectra (sum of absorption at 230 nm and 254 nm) of the LC-MS analysis of 100 µM of **6e** incubated with 5 mM NAC for 16 h in PBS buffer pH 7.4 at room temperature. The blank sample is shown in black while the reaction in magenta. Internal standard: RT = 1.5 min; **6e**: RT = 2.35 min.

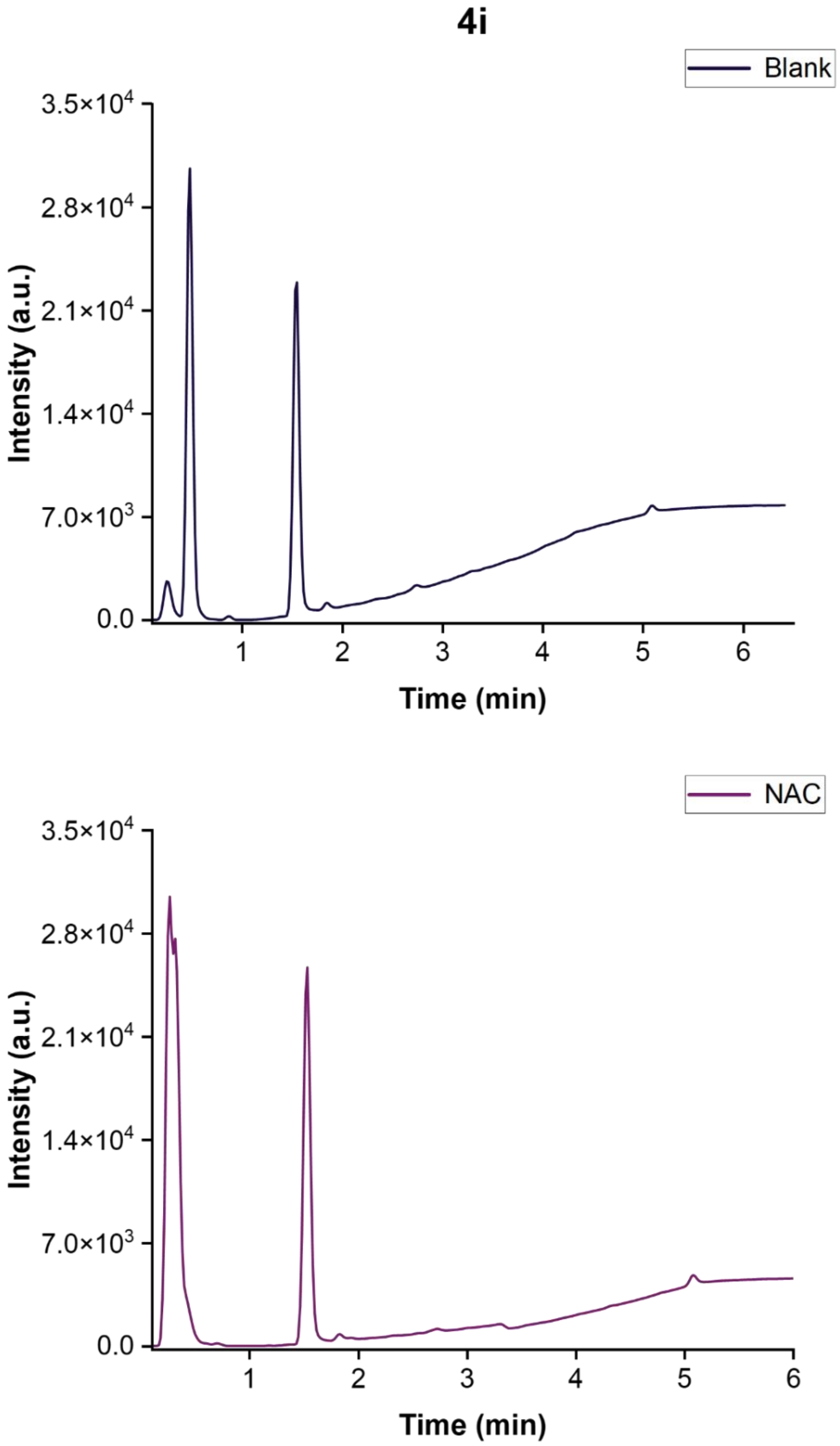

Figure S309. UV spectra (sum of absorption at 230 nm and 254 nm) of the LC-MS analysis of 100 µM of **4i** incubated with 5 mM NAC for 16 h in PBS buffer pH 7.4 at room temperature. The blank sample is shown in black while the reaction in magenta. Internal standard: RT = 1.5 min; **4i**: RT = 0.48 min.

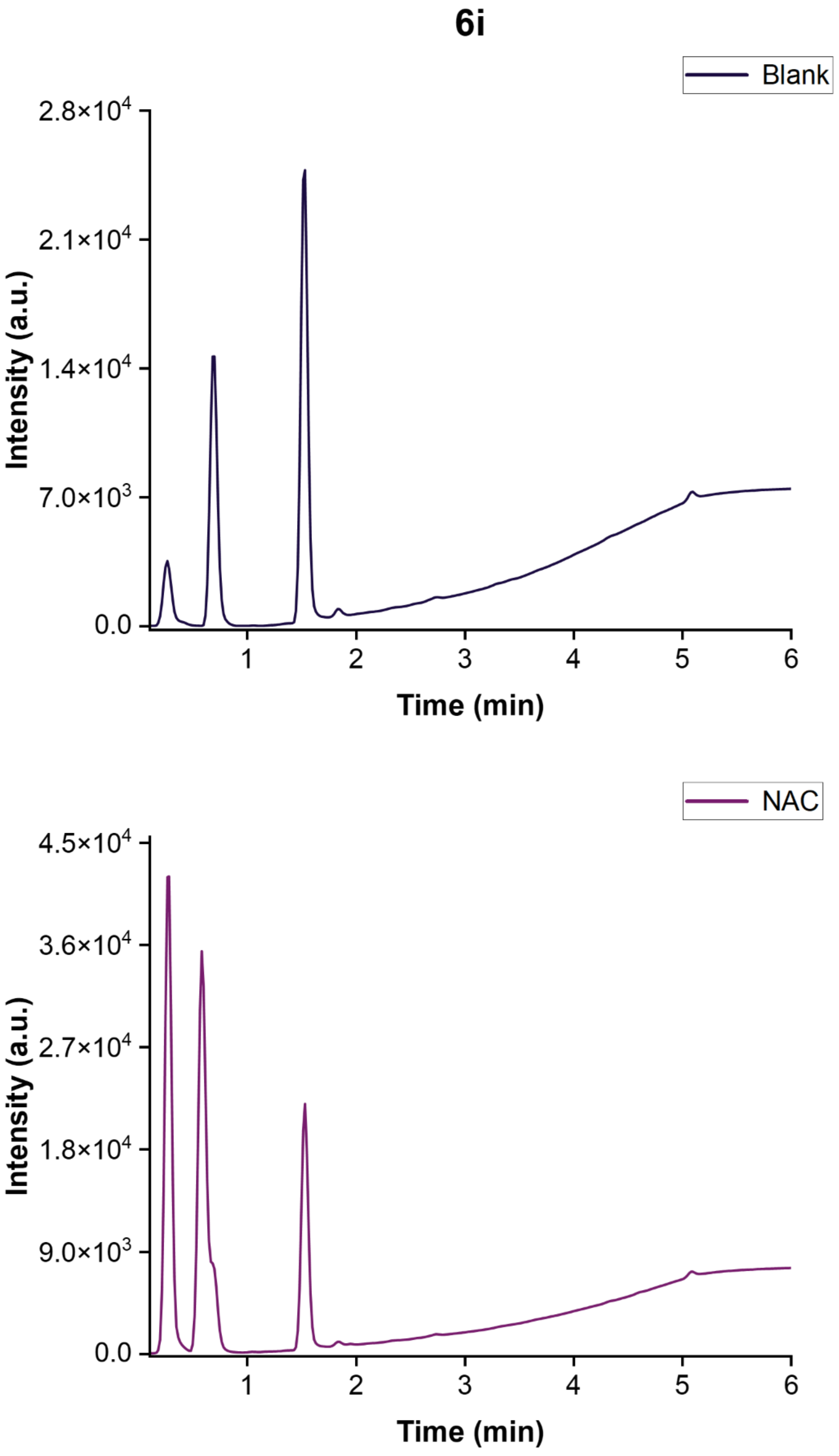

Figure S310. UV spectra (sum of absorption at 230 nm and 254 nm) of the LC-MS analysis of 100 µM of **6i** incubated with 5 mM NAC for 16 h in PBS buffer pH 7.4 at room temperature. The blank sample is shown in black while the reaction in magenta. Internal standard: RT = 1.5 min; **6i**: RT = 0.7 min.

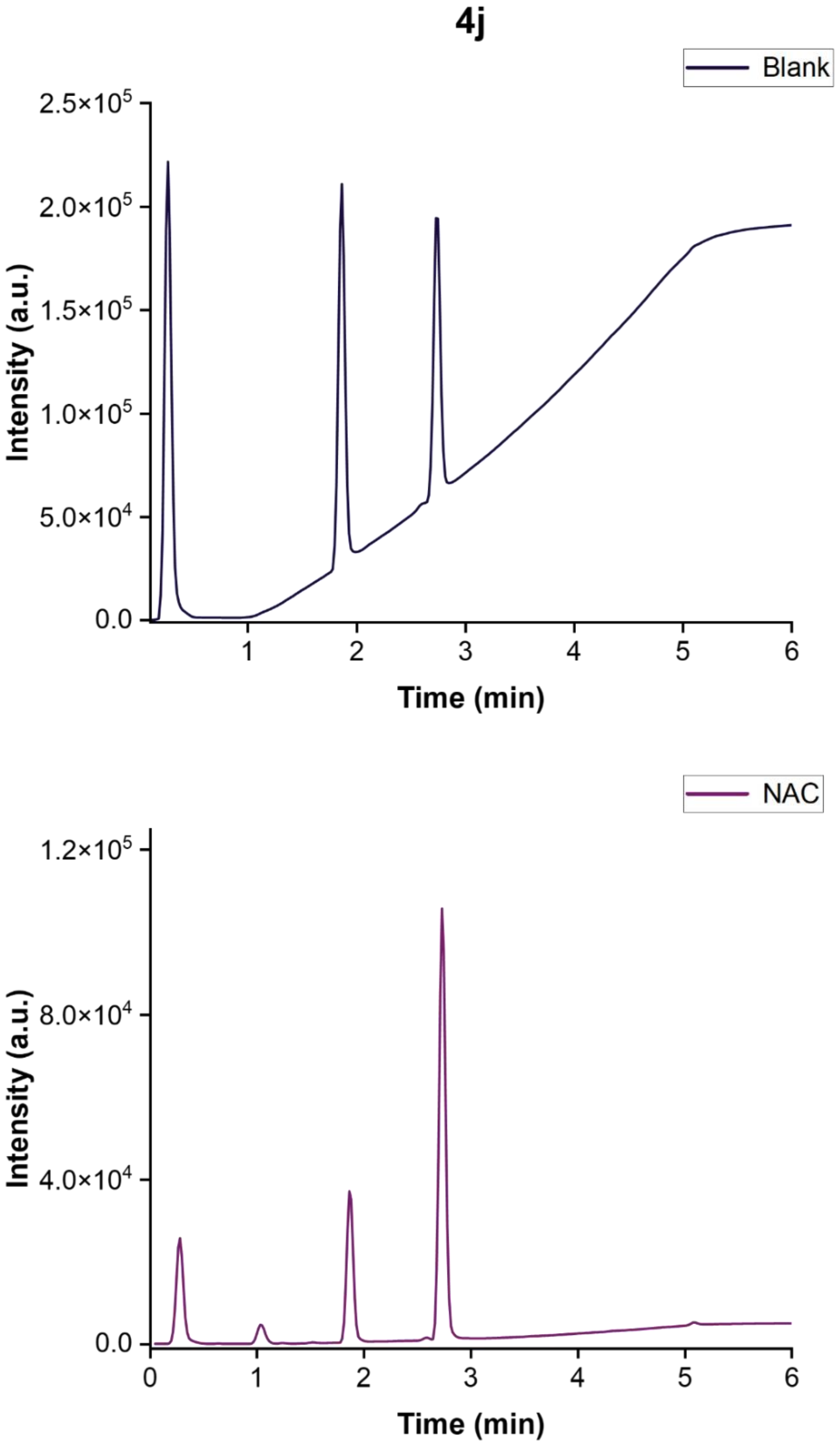

Figure S311. UV spectra (sum of absorption at 230 nm and 254 nm) of the LC-MS analysis of 100 μM of **4j** incubated with 5 mM NAC for 16 h in PBS buffer pH 7.4 at room temperature. The blank sample is shown in black while the reaction in magenta. Internal standard: RT = 2.7 min; **4j**: RT = 1.87 min.

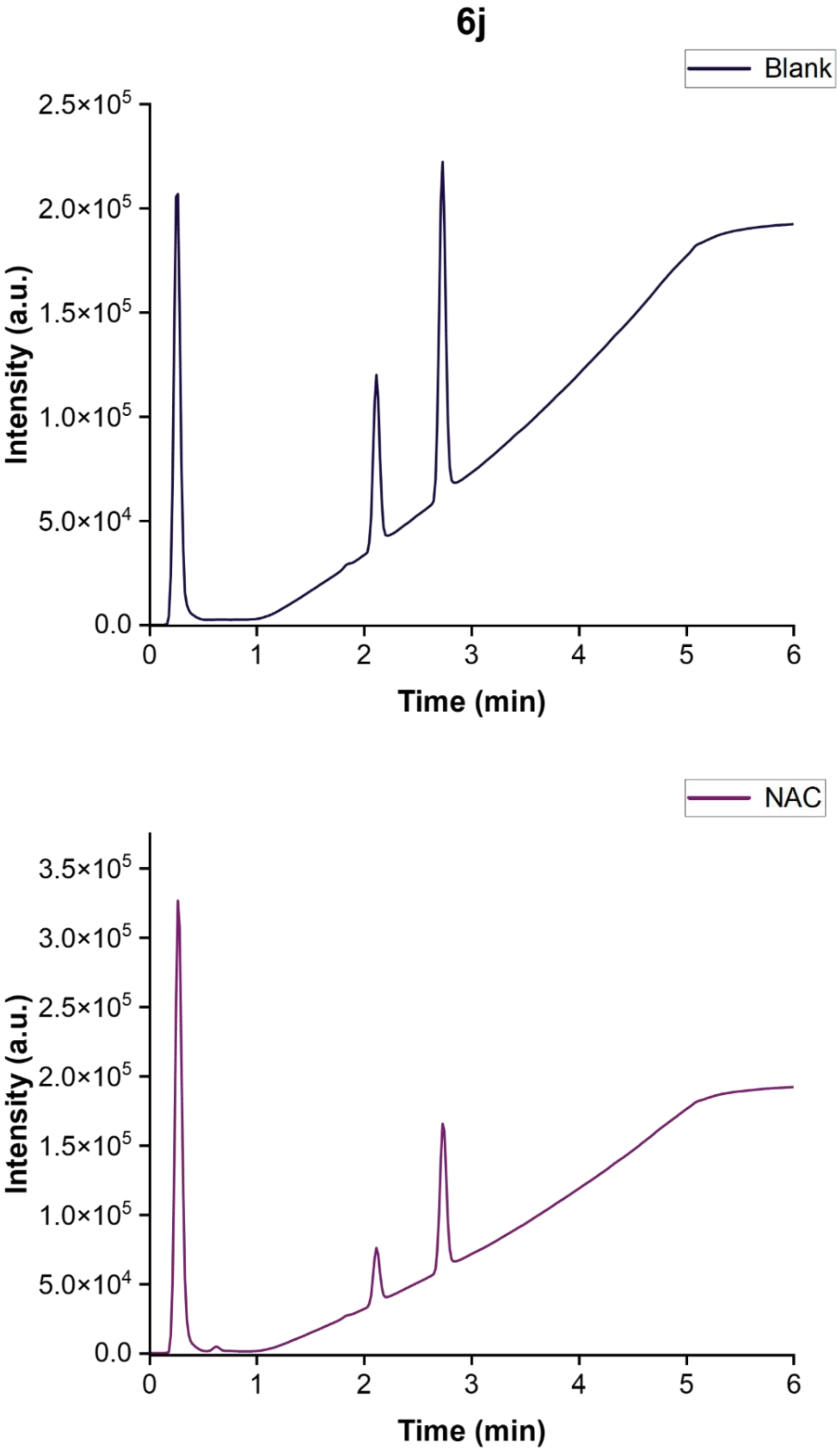

Figure S312. UV spectra (sum of absorption at 230 nm and 254 nm) of the LC-MS analysis of 100 μM of **6j** incubated with 5 mM NAC for 16 h in PBS buffer pH 7.4 at room temperature. The blank sample is shown in black while the reaction in magenta. Internal standard: RT = 2.1 min; **6j**: RT = 2.7 min.

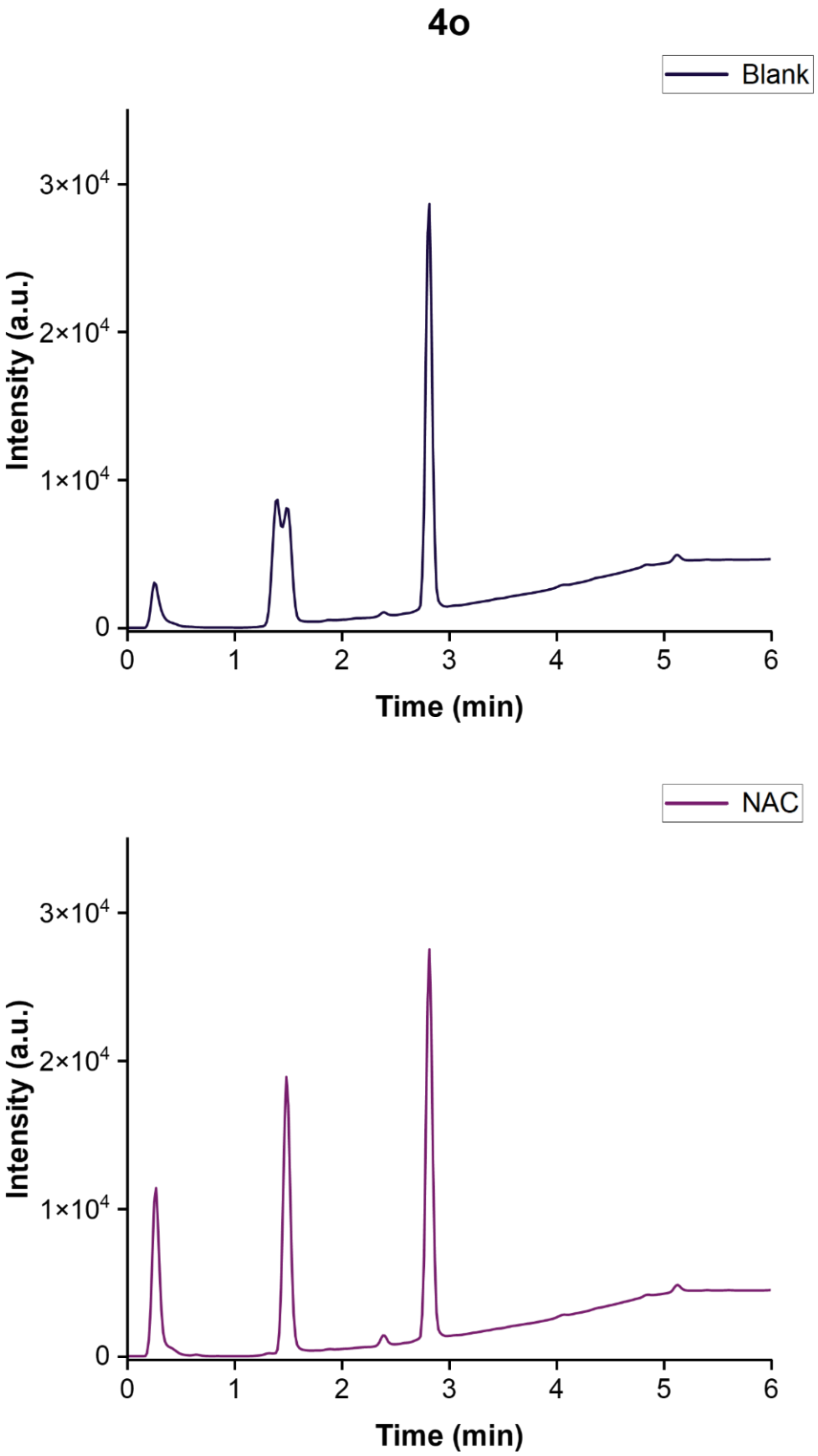

Figure S313. UV spectra (sum of absorption at 230 nm and 254 nm) of the LC-MS analysis of 100 µM of **4o** incubated with 5 mM NAC for 16 h in PBS buffer pH 7.4 at room temperature. The blank sample is shown in black while the reaction in magenta. Internal standard: RT = 1.5 min; **4o**: RT = 2.82 min.

6o

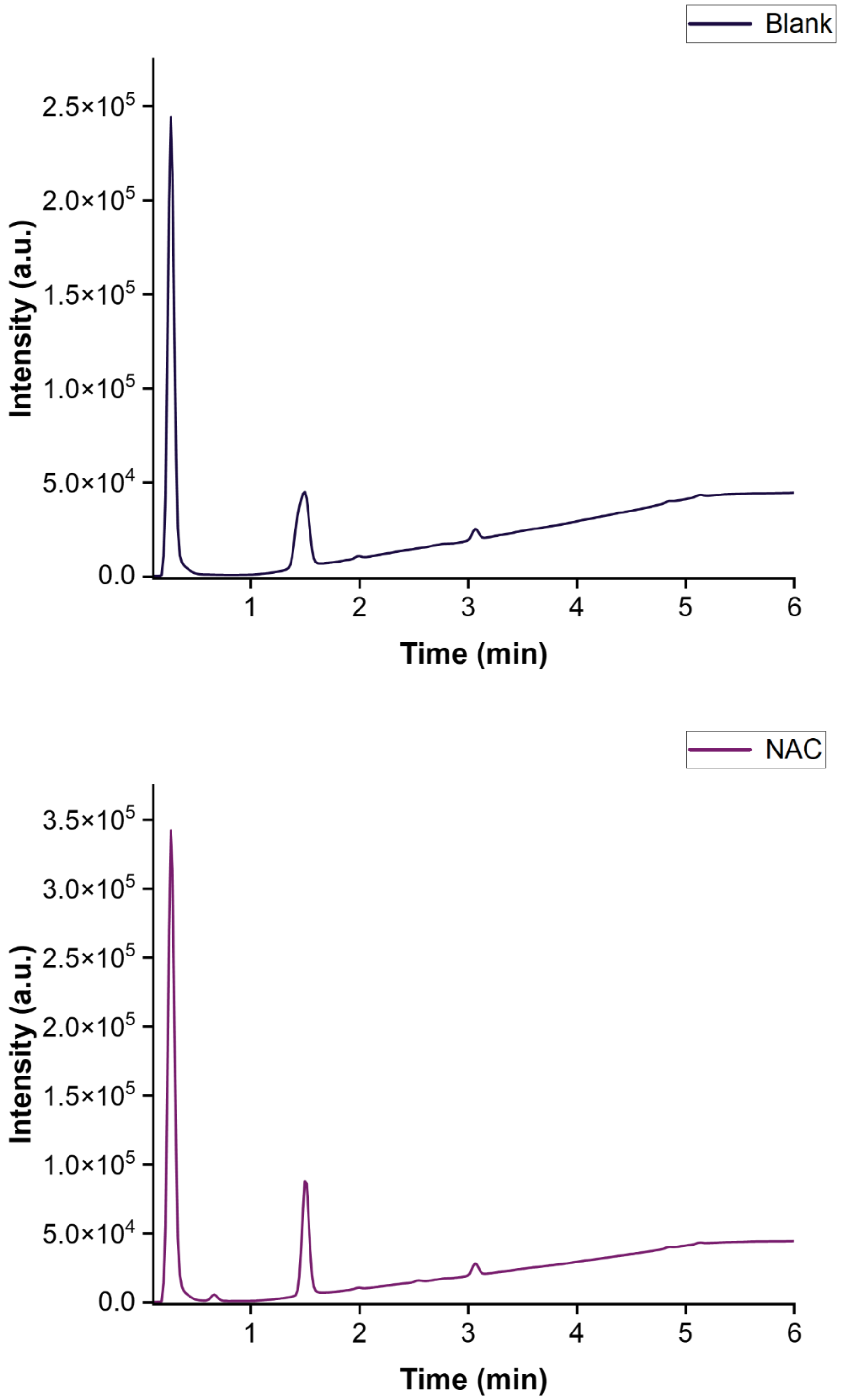

Figure S314. UV spectra (sum of absorption at 230 nm and 254 nm) of the LC-MS analysis of 100 µM of **6o** incubated with 5 mM NAC for 16 h in PBS buffer pH 7.4 at room temperature. The blank sample is shown in black while the reaction in magenta. Internal standard: RT = 1.5 min; **6o**: RT = 3.07 min.

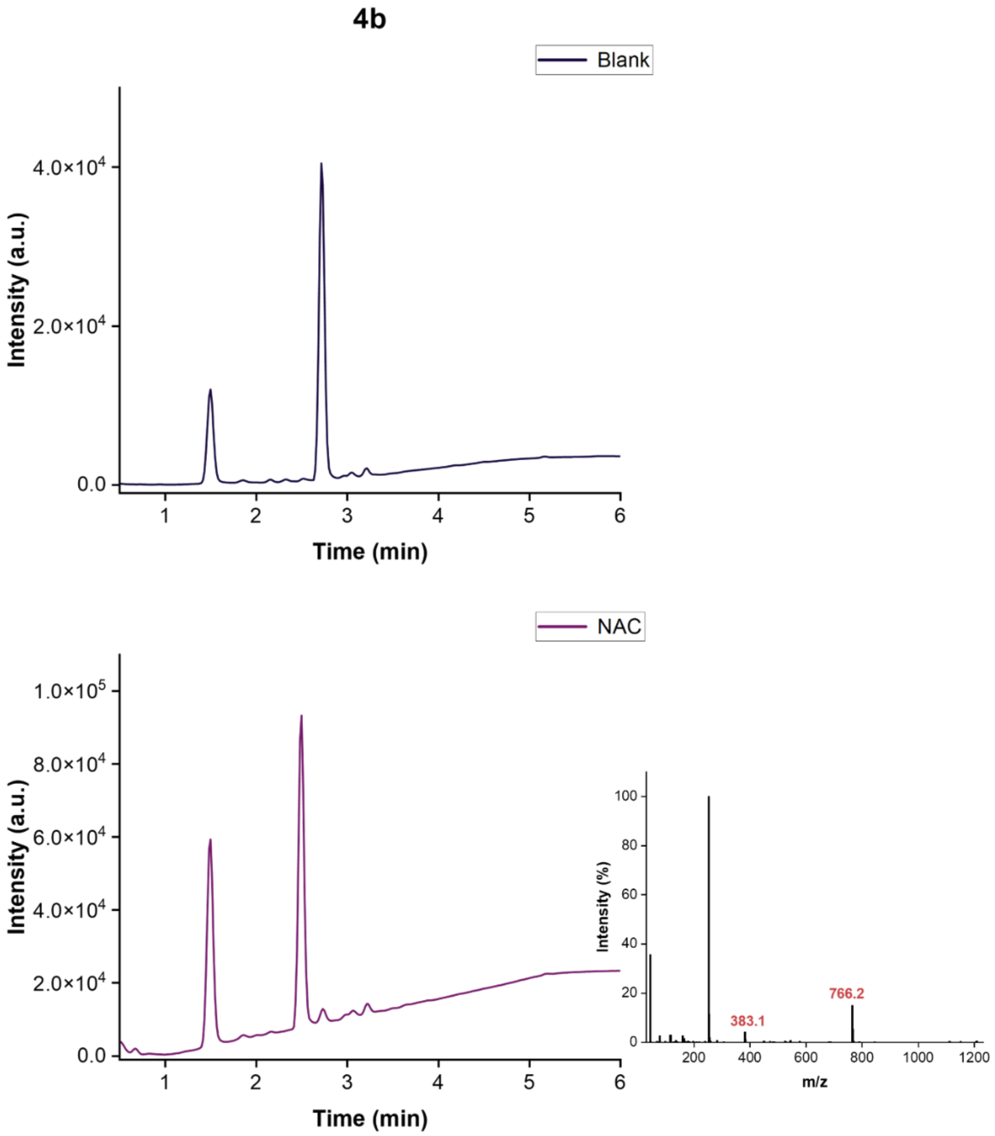

Figure S315. UV (sum of absorption at 230 nm and 254 nm) and MS spectra of the LC-MS analysis of 100 µM of **4b** incubated with 5 mM NAC for 16 h in PBS buffer pH 7.4 at room temperature. The blank sample is shown in black while the reaction in magenta. Internal standard: RT = 1.5 min; **4b**: RT = 2.72 min, NAC-adduct: RT = 2.5 min, *m/z* = 383.1 $[M-H]^-$.

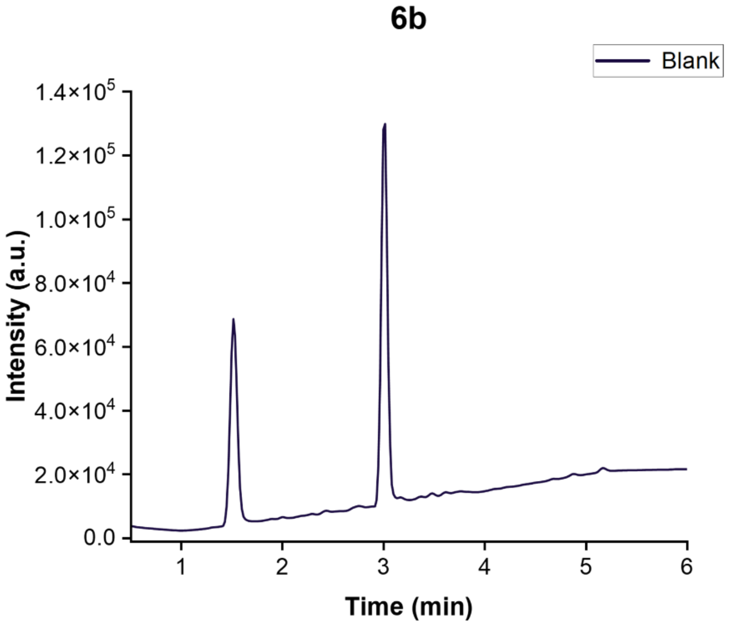

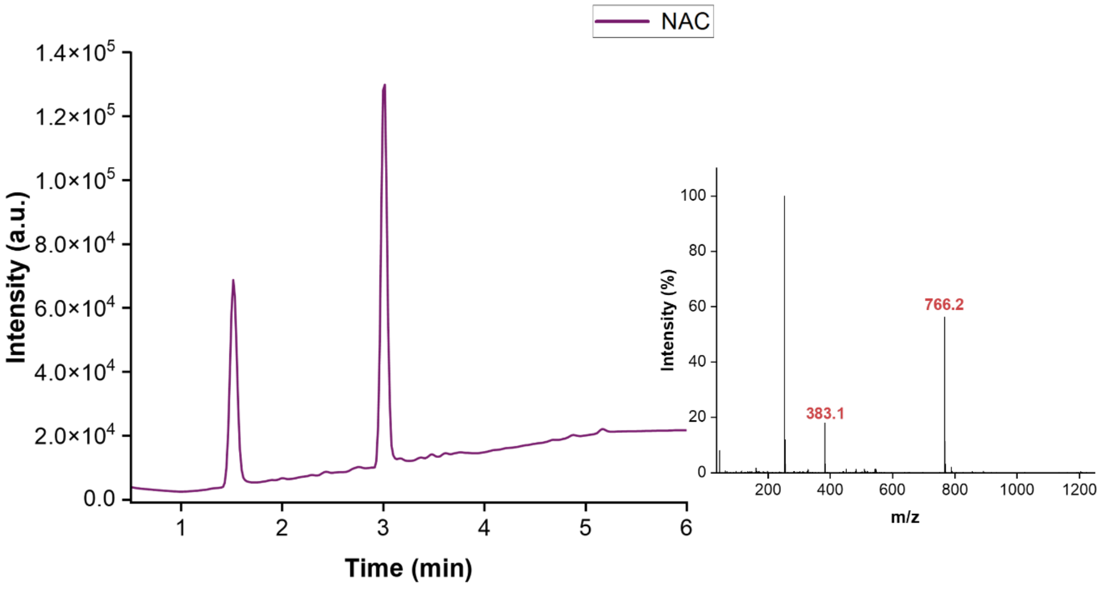

Figure S316. UV (sum of absorption at 230 nm and 254 nm) and MS spectra of the LC-MS analysis of 100 µM of **6b** incubated with 5 mM NAC for 16 h in PBS buffer pH 7.4 at room temperature. The blank sample is shown in black while the reaction in magenta. Internal standard: RT = 1.5 min; **6b**: RT = 3 min, NAC-adduct: RT = 2.5 min, *m/z* = 383.1 $[M-H]^-$.

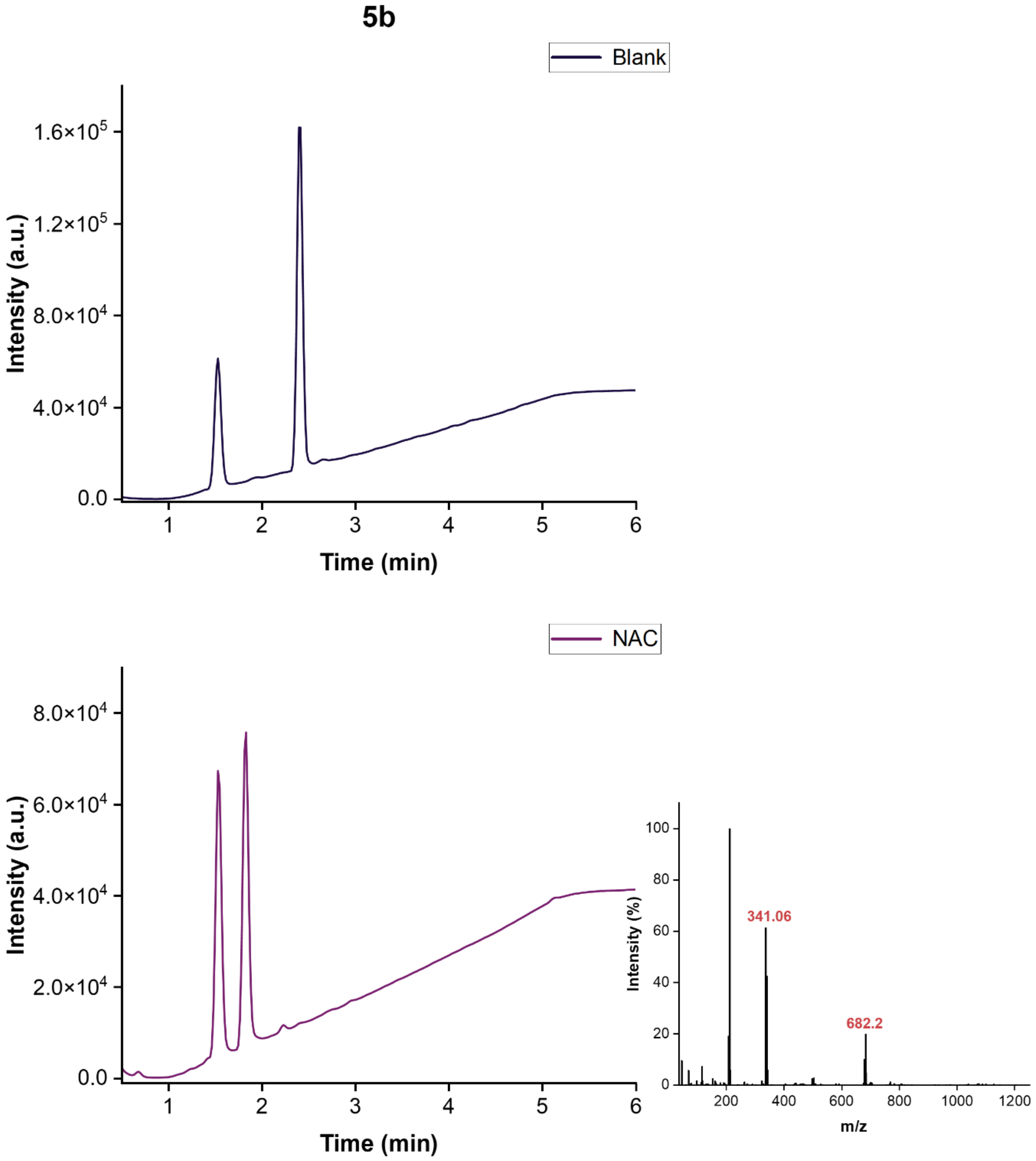

Figure S317. UV (sum of absorption at 230 nm and 254 nm) and MS spectra of the LC-MS analysis of 100 µM of **5b** incubated with 5 mM NAC for 16 h in PBS buffer pH 7.4 at room temperature. The blank sample is shown in black while the reaction in magenta. Internal standard: RT = 1.5 min; **5b**: RT = 2.4 min, NAC-adduct: RT = 1.8 min, *m/z* = 341.06 [M-H]$^-$.

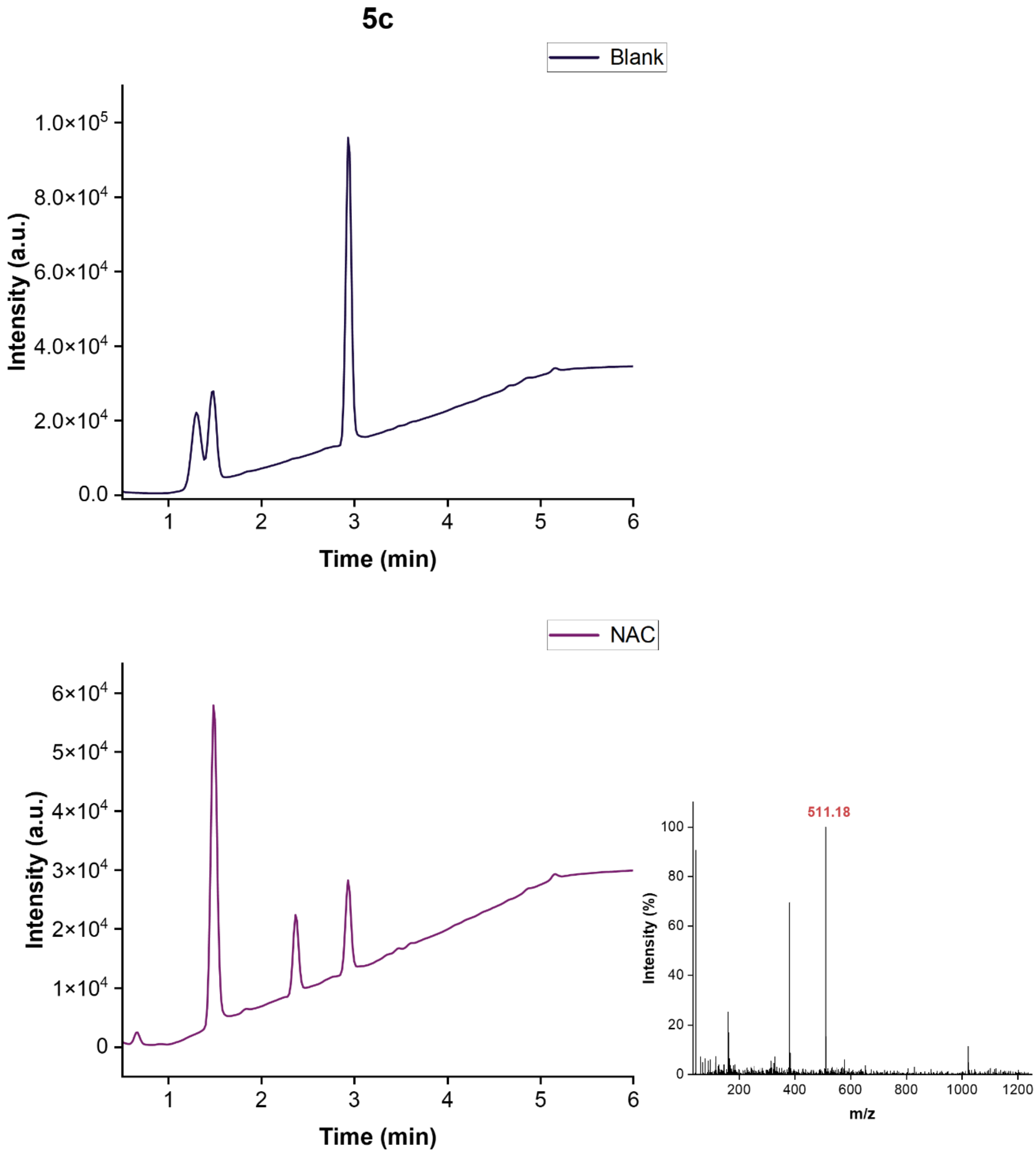

Figure S318. UV (sum of absorption at 230 nm and 254 nm) and MS spectra of the LC-MS analysis of 100 μM of **5c** incubated with 5 mM NAC for 16 h in PBS buffer pH 7.4 at room temperature. The blank sample is shown in black while the reaction in magenta. Internal standard: RT = 1.5 min; **5c**: RT = 2.93 min, NAC-adduct: RT = 2.37 min, *m/z* = 511.18 [M-H]⁻.

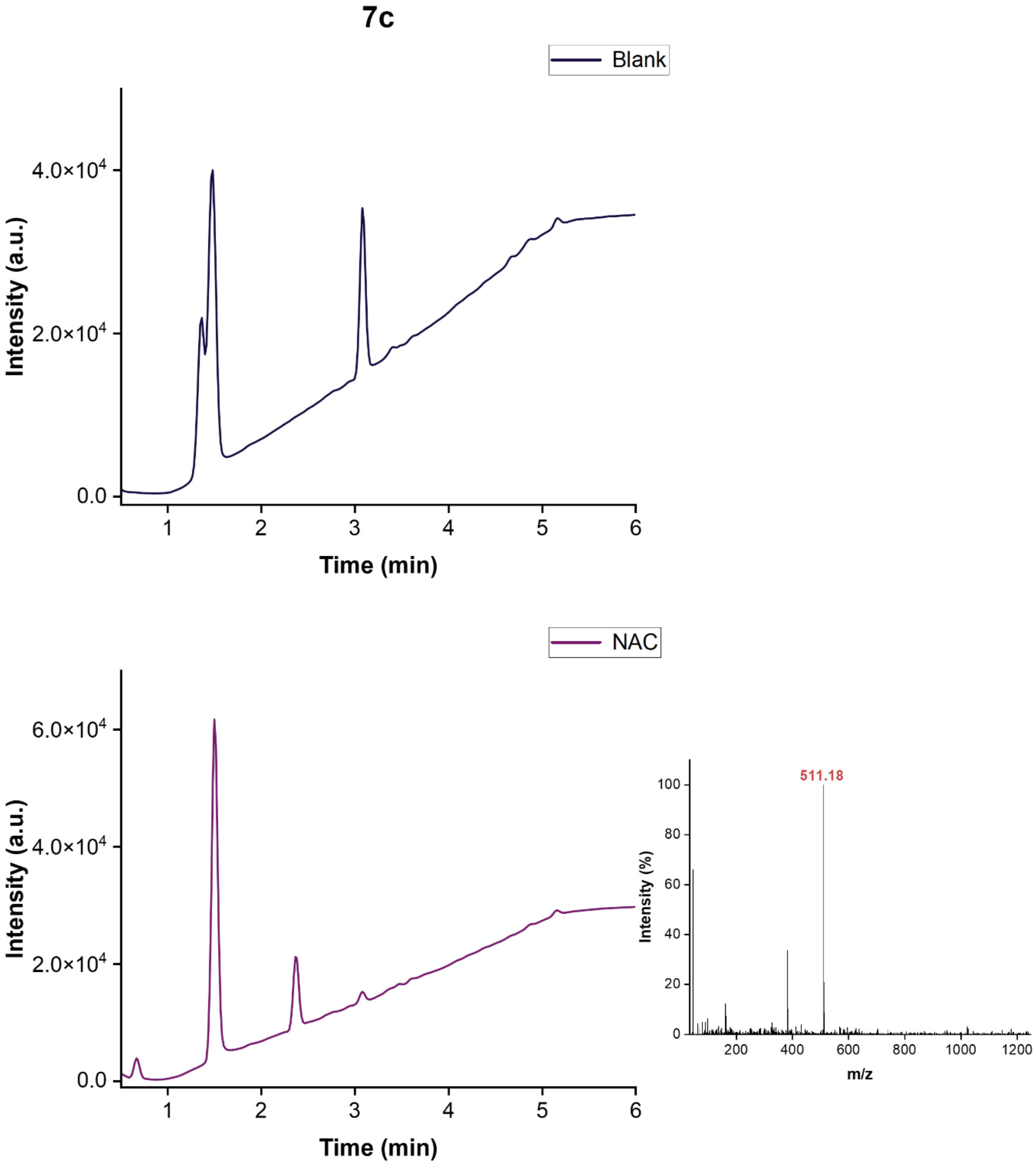

Figure S319. UV (sum of absorption at 230 nm and 254 nm) and MS spectra of the LC-MS analysis of 100 μM of **7c** incubated with 5 mM NAC for 16 h in PBS buffer pH 7.4 at room temperature. The blank sample is shown in black while the reaction in magenta. Internal standard: RT = 1.5 min; **7c**: RT = 3.08 min, NAC-adduct: RT = 2.37 min, *m/z* = 511.18 $[M-H]^-$.

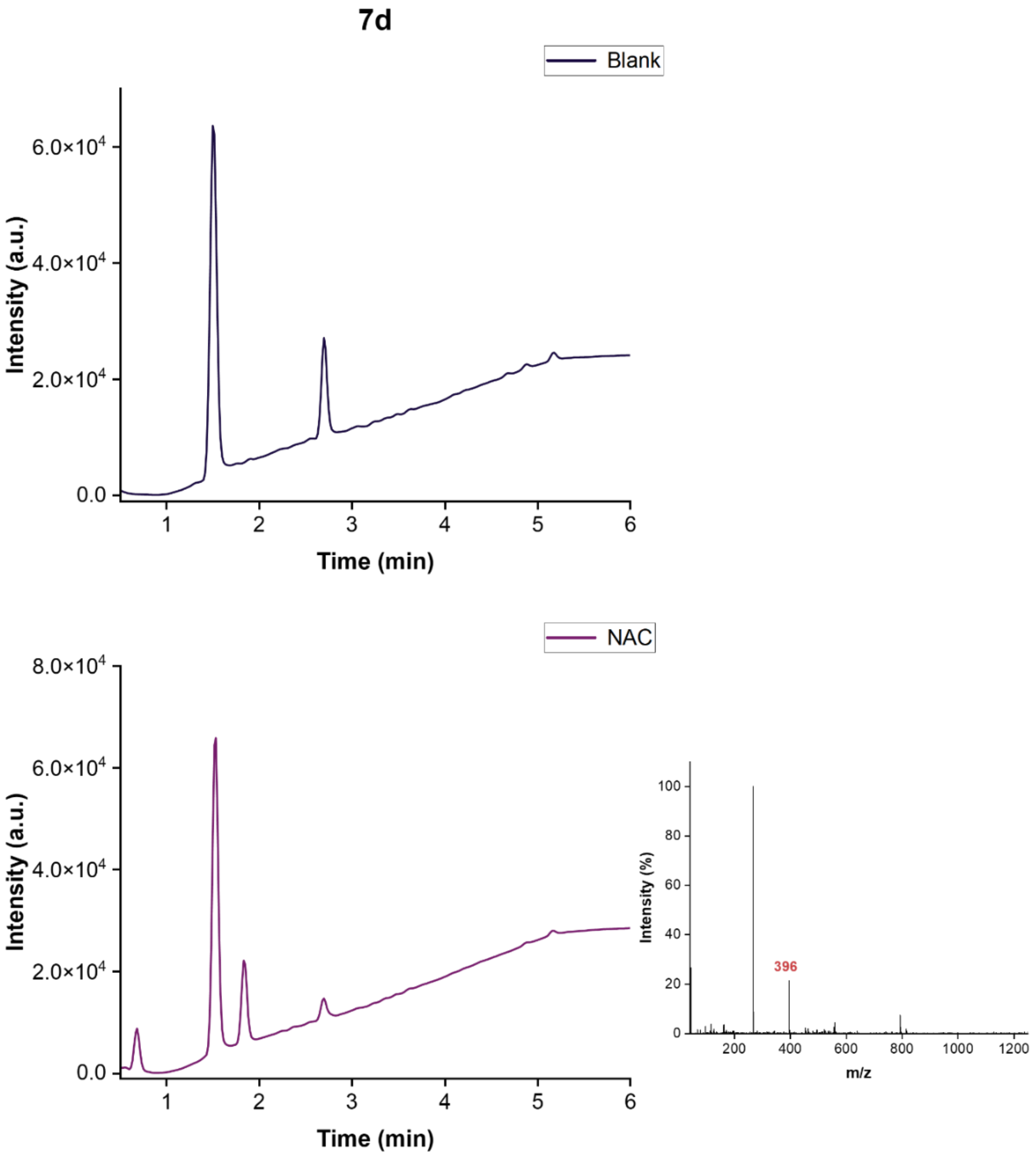

Figure S320. UV (sum of absorption at 230 nm and 254 nm) and MS spectra of the LC-MS analysis of 100 μM of **7d** incubated with 5 mM NAC for 16 h in PBS buffer pH 7.4 at room temperature. The blank sample is shown in black while the reaction in magenta. Internal standard: RT = 1.5 min; **7d**: RT = 2.7 min, NAC-adduct: RT = 1.8 min, *m/z* = 396 $[M-H]^-$.

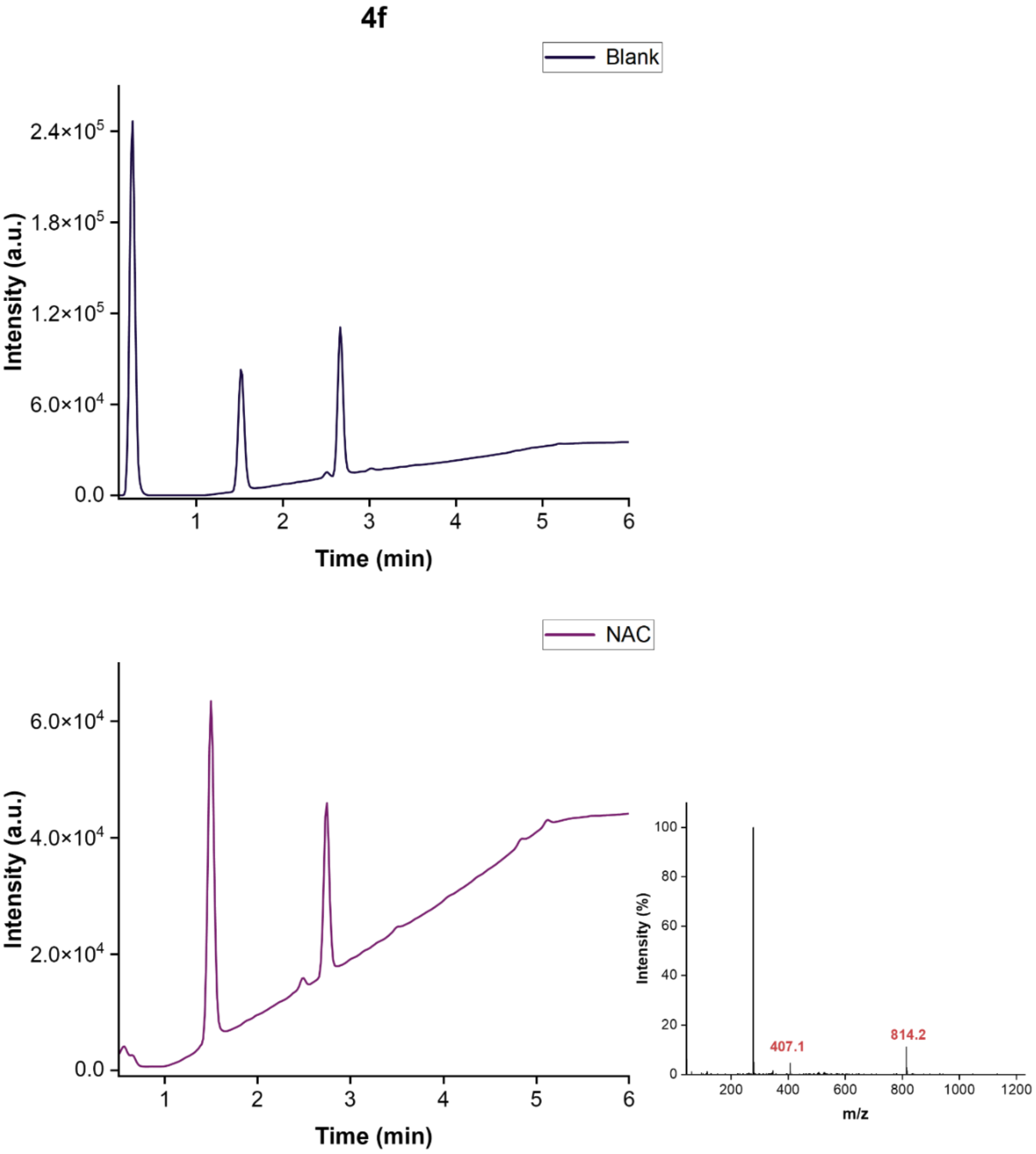

Figure S321. UV (sum of absorption at 230 nm and 254 nm) and MS spectra of the LC-MS analysis of 100 µM of **4f** incubated with 5 mM NAC for 16 h in PBS buffer pH 7.4 at room temperature. The blank sample is shown in black while the reaction in magenta. Internal standard: RT = 1.5 min; **4f**: RT = 3 min, NAC-adduct: RT = 2.75 min, *m/z* = 407.17 [M-H]$^-$.

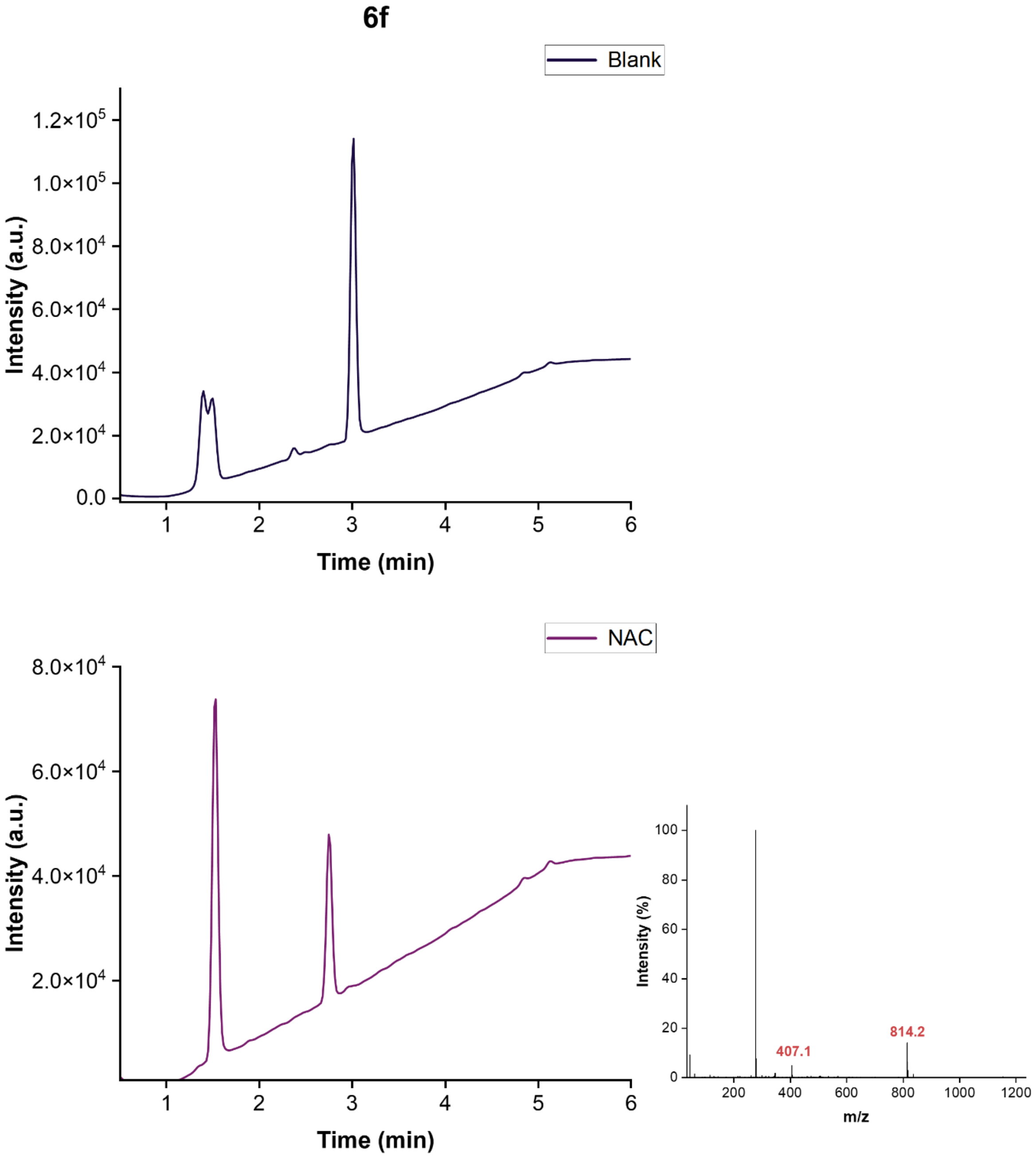

Figure S322. UV (sum of absorption at 230 nm and 254 nm) and MS spectra of the LC-MS analysis of 100 µM of **6f** incubated with 5 mM NAC for 16 h in PBS buffer pH 7.4 at room temperature. The blank sample is shown in black while the reaction in magenta. Internal standard: RT = 1.5 min; **6f**: RT = 3 min, NAC-adduct: RT = 2.75 min, *m/z* = 407.17 [M-H]$^-$.

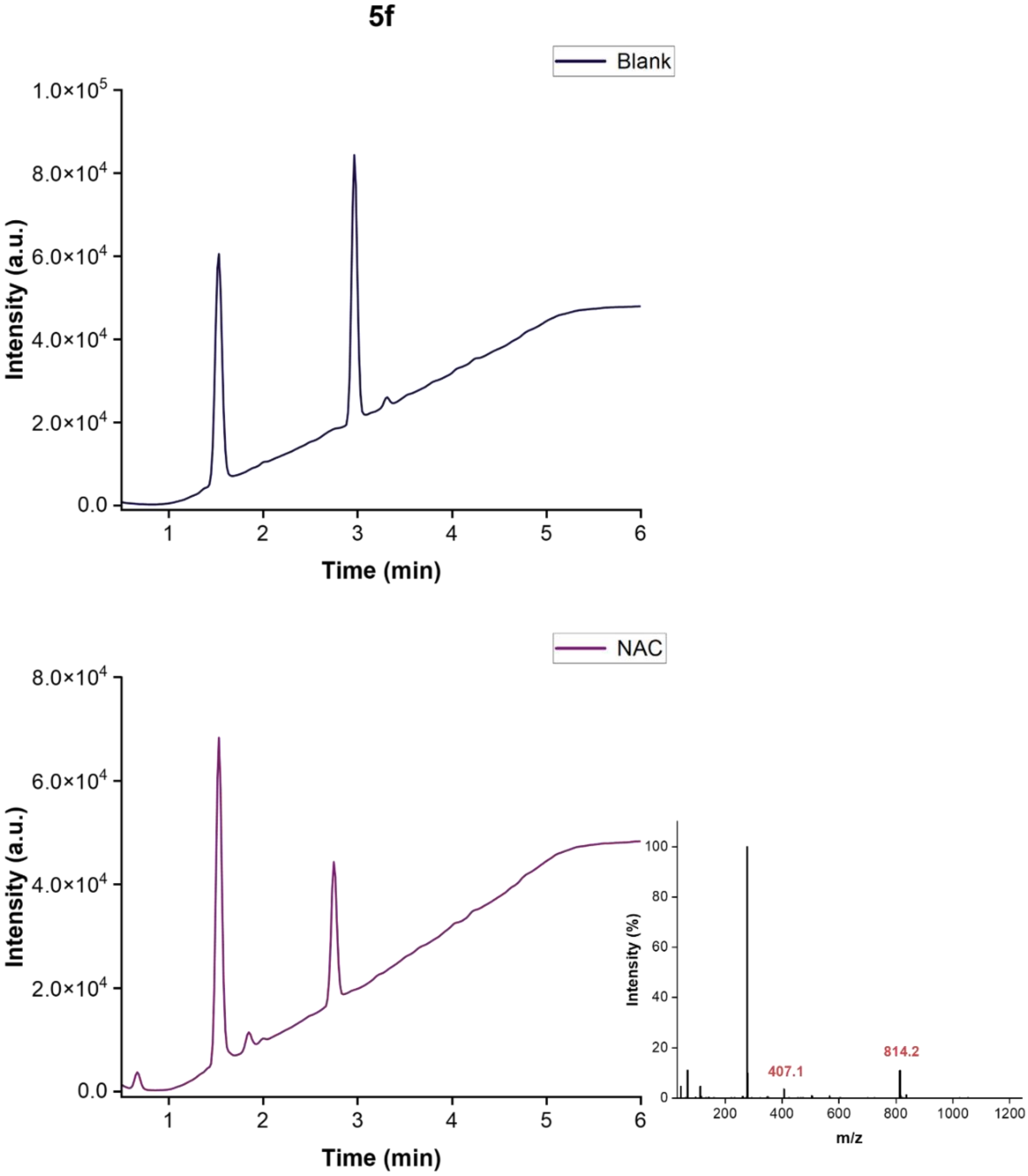

Figure S323. UV (sum of absorption at 230 nm and 254 nm) and MS spectra of the LC-MS analysis of 100 µM of **5f** incubated with 5 mM NAC for 16 h in PBS buffer pH 7.4 at room temperature. The blank sample is shown in black while the reaction in magenta. Internal standard: RT = 1.5 min; **5f**: RT = 2.97 min, NAC-adduct: RT = 2.75 min, *m/z* = 407.17 $[M-H]^-$.

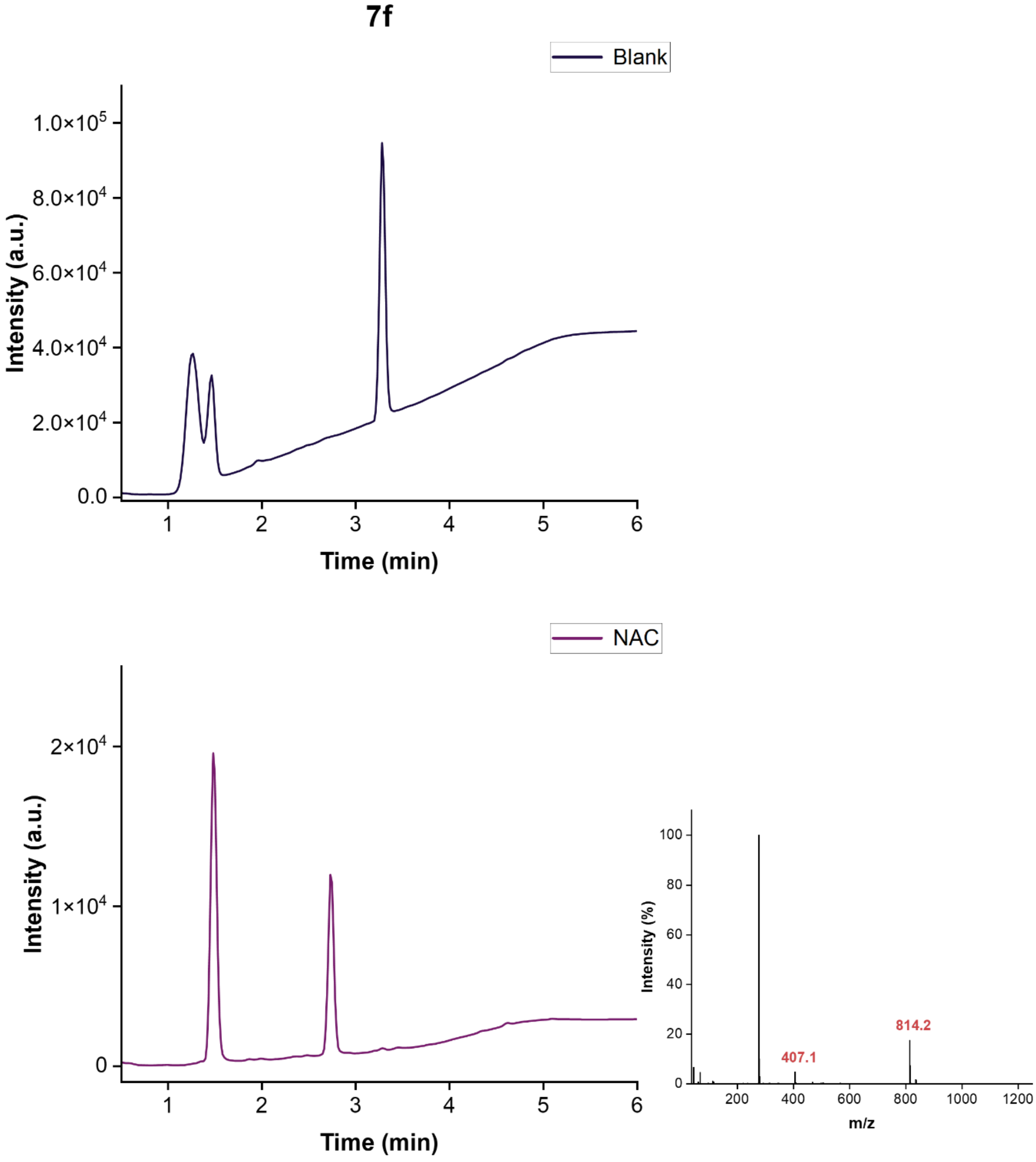

Figure S324. UV (sum of absorption at 230 nm and 254 nm) and MS spectra of the LC-MS analysis of 100 μM of **7f** incubated with 5 mM NAC for 16 h in PBS buffer pH 7.4 at room temperature. The blank sample is shown in black while the reaction in magenta. Internal standard: RT = 1.5 min; **7f**: RT = 3.3 min, NAC-adduct: RT = 2.75 min, *m/z* = 407.17 $[M-H]^-$.

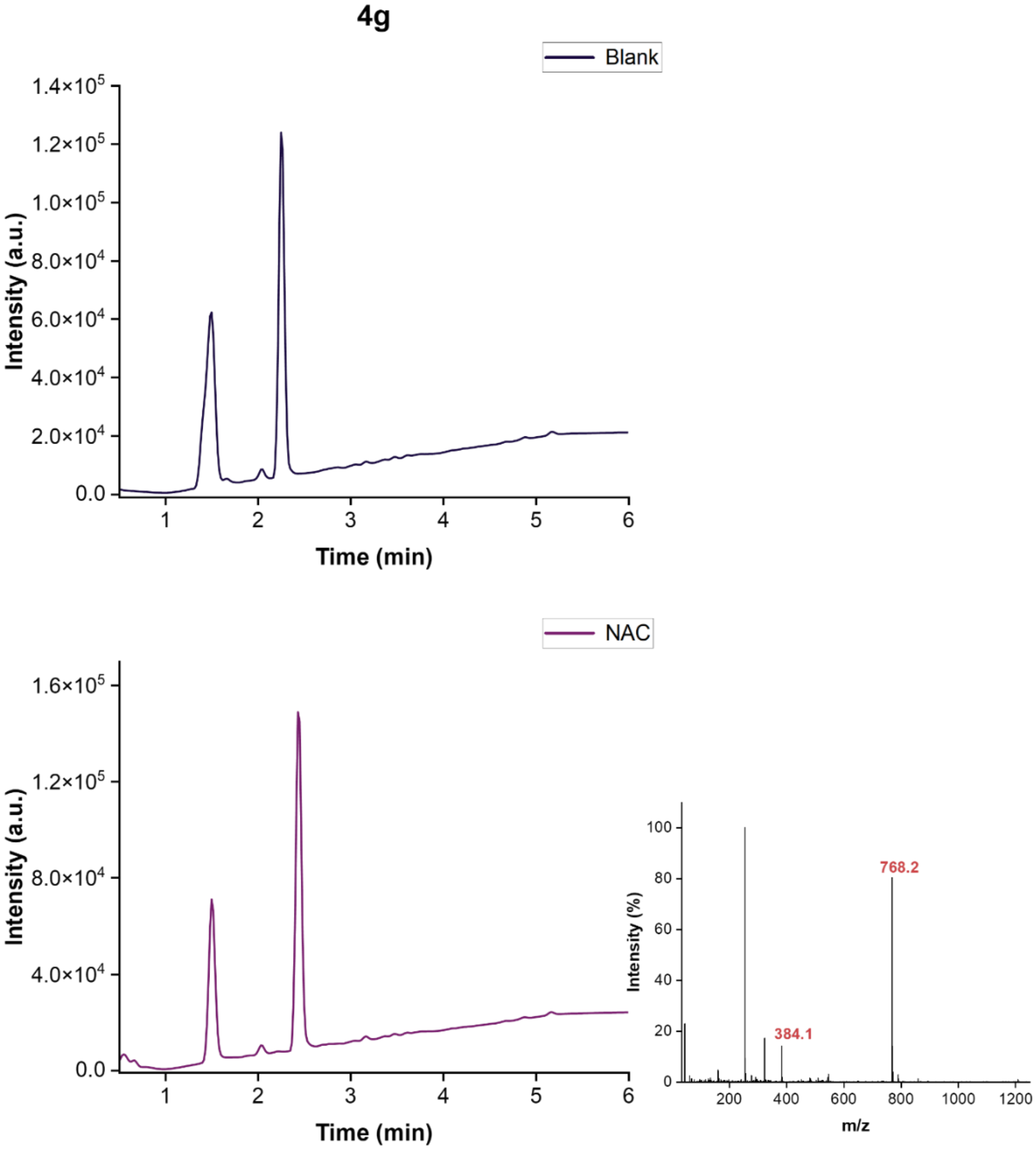

Figure S325. UV (sum of absorption at 230 nm and 254 nm) and MS spectra of the LC-MS analysis of 100 μM of **4g** incubated with 5 mM NAC for 16 h in PBS buffer pH 7.4 at room temperature. The blank sample is shown in black while the reaction in magenta. Internal standard: RT = 1.5 min; **4g**: RT = 2.2 min, NAC-adduct: RT = 2.4 min, *m/z* = 384.1 $[M-H]^-$.

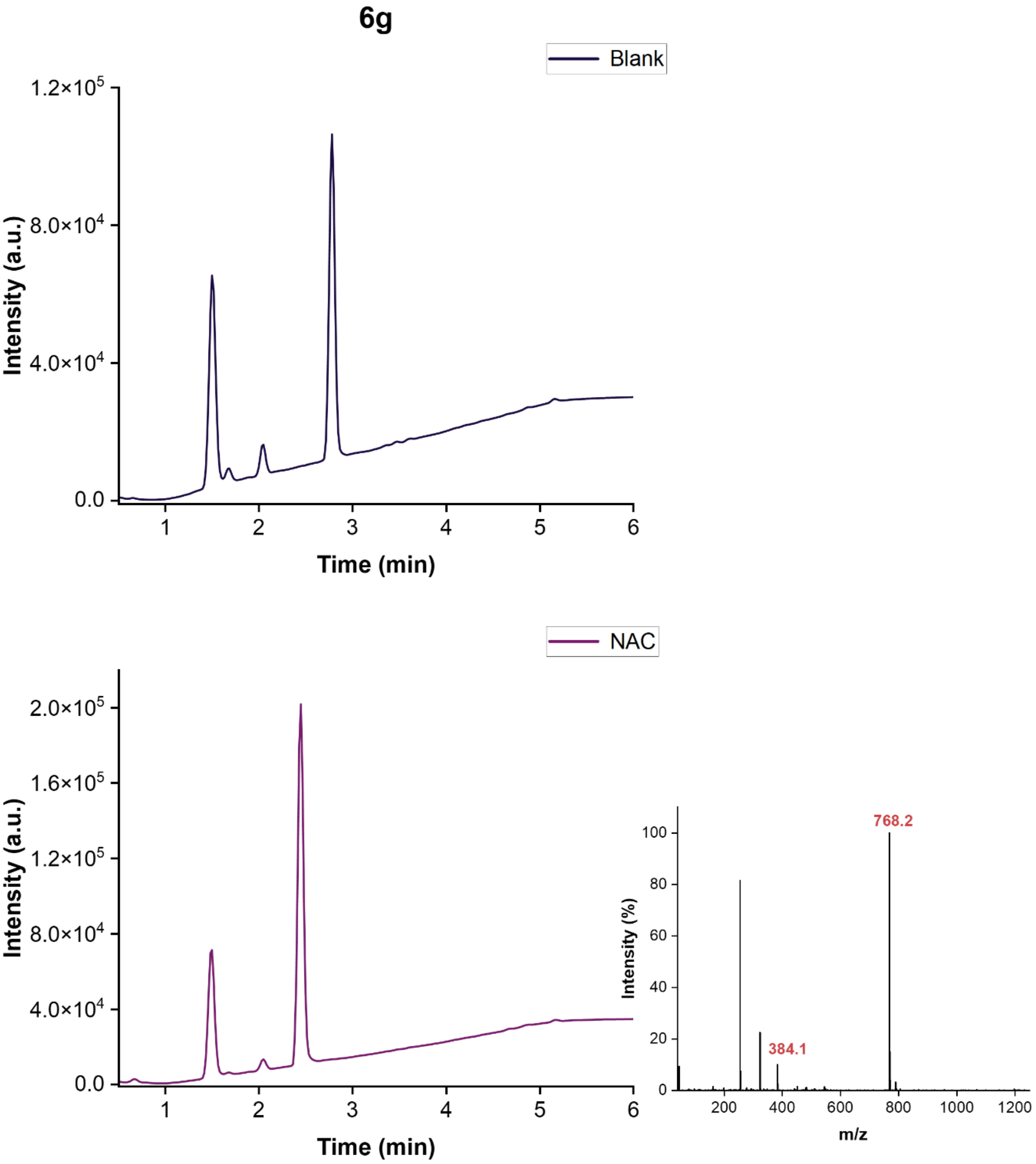

Figure S326. UV (sum of absorption at 230 nm and 254 nm) and MS spectra of the LC-MS analysis of 100 μM of **6g** incubated with 5 mM NAC for 16 h in PBS buffer pH 7.4 at room temperature. The blank sample is shown in black while the reaction in magenta. Internal standard: RT = 1.5 min; **6g**: RT = 2.78 min, NAC-adduct: RT = 2.4 min, *m/z* = 384.1 $[M-H]^-$.

5g

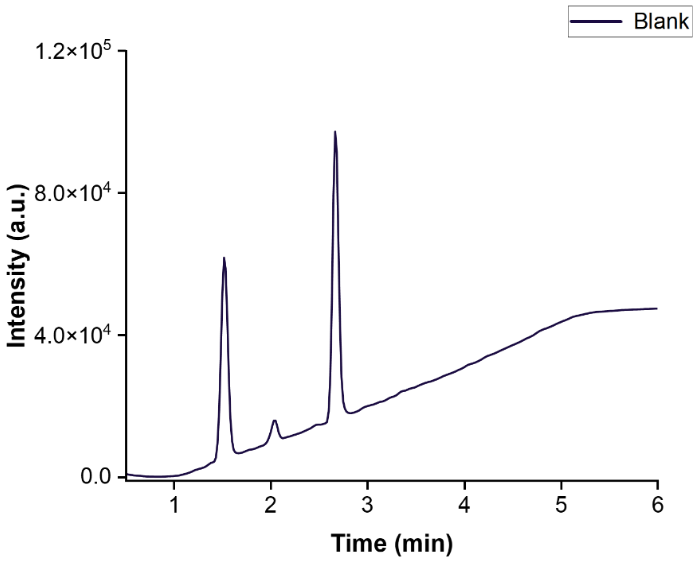

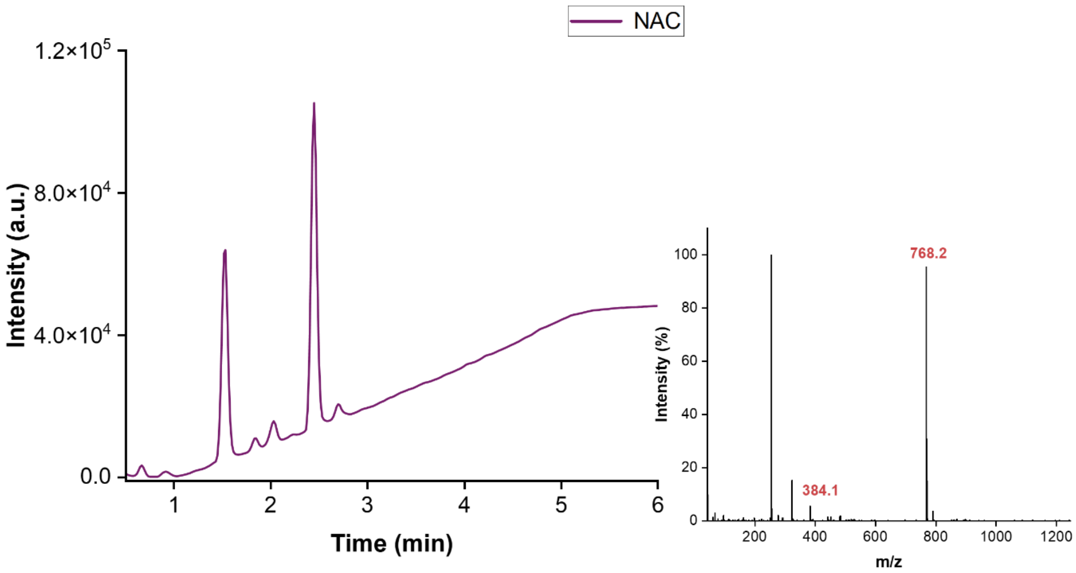

Figure S327. UV (sum of absorption at 230 nm and 254 nm) and MS spectra of the LC-MS analysis of 100 µM of **5g** incubated with 5 mM NAC for 16 h in PBS buffer pH 7.4 at room temperature. The blank sample is shown in black while the reaction in magenta. Internal standard: RT = 1.5 min; **5g**: RT = 2.67 min, NAC-adduct: RT = 2.4 min, *m/z* = 384.1 [M-H]$^-$.

4h

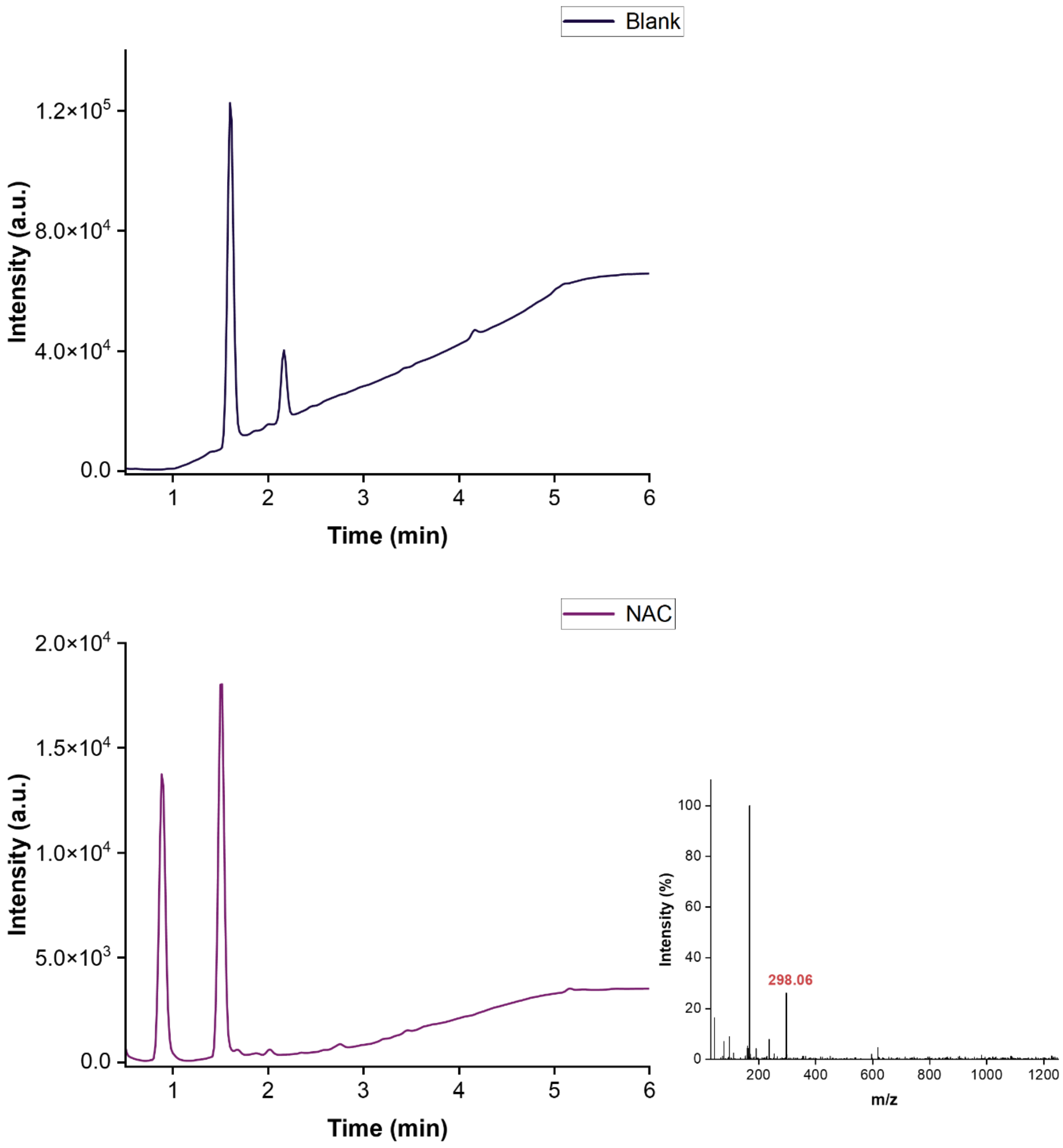

Figure S328. UV (sum of absorption at 230 nm and 254 nm) and MS spectra of the LC-MS analysis of 100 μM of **4h** incubated with 5 mM NAC for 16 h in PBS buffer pH 7.4 at room temperature. The blank sample is shown in black while the reaction in magenta. Internal standard: RT = 1.5 min; **4h**: RT = 2.2 min, NAC-adduct: RT = 0.88 min, m/z = 289.06 [M-H]$^-$.

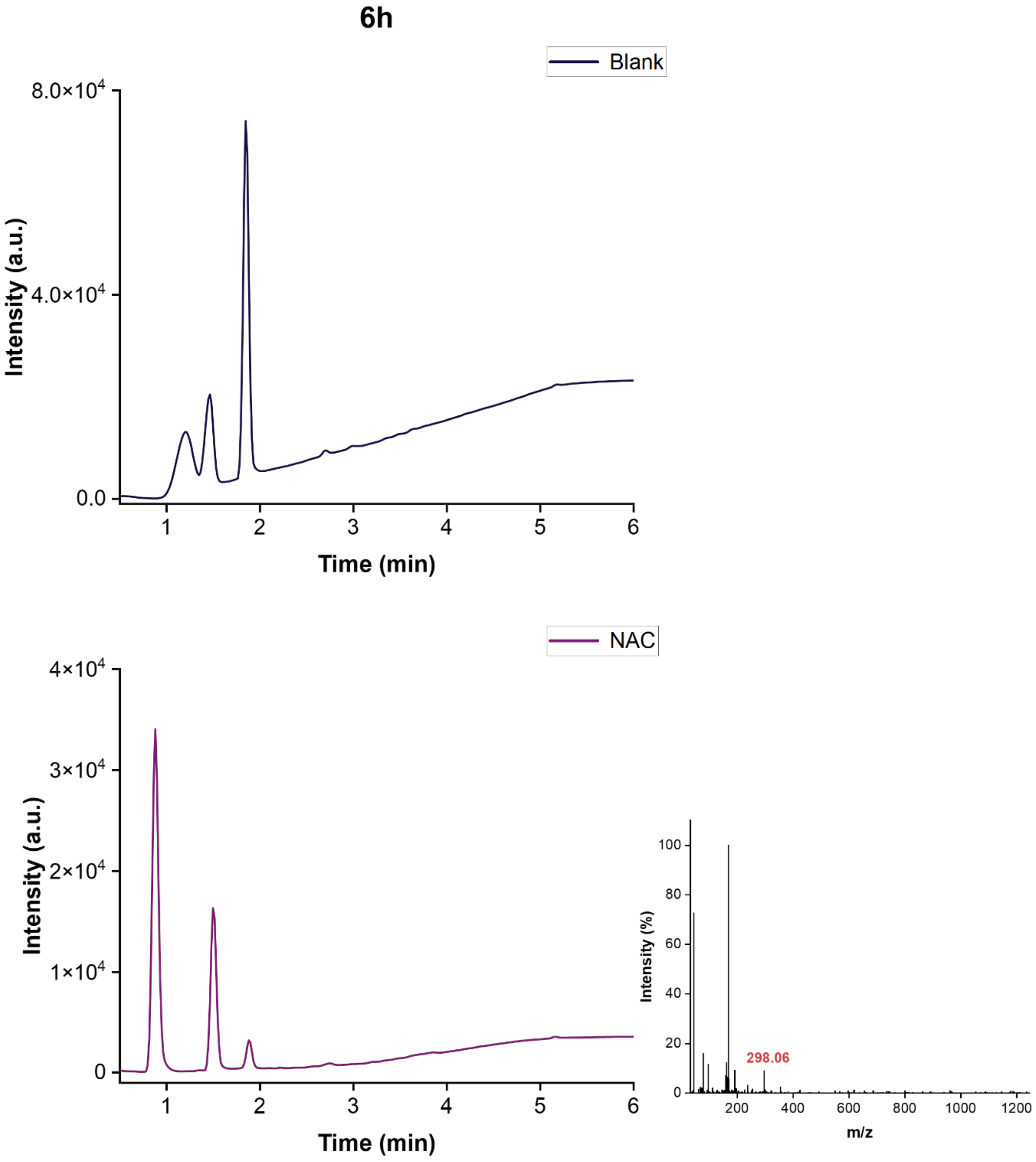

Figure S329. UV (sum of absorption at 230 nm and 254 nm) and MS spectra of the LC-MS analysis of 100 μM of **6h** incubated with 5 mM NAC for 16 h in PBS buffer pH 7.4 at room temperature. The blank sample is shown in black while the reaction in magenta. Internal standard: RT = 1.5 min; **6h**: RT = 1.88 min, NAC-adduct: RT = 0.88 min, *m/z* = 289.06 $[M-H]^-$.

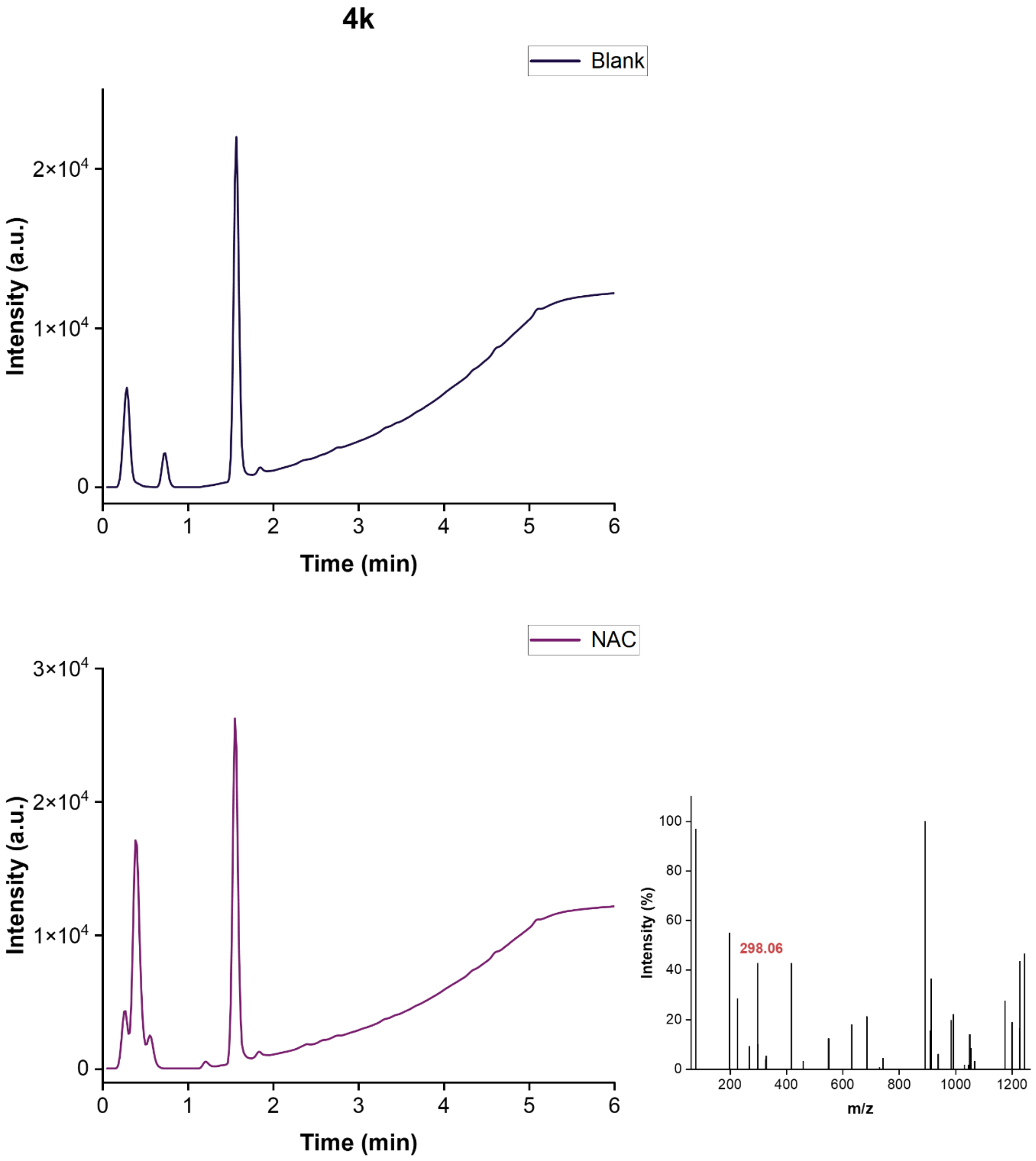

Figure S330. UV (sum of absorption at 230 nm and 254 nm) and MS spectra of the LC-MS analysis of 100 µM of **4k** incubated with 5 mM NAC for 16 h in PBS buffer pH 7.4 at room temperature. The blank sample is shown in black while the reaction in magenta. Internal standard: RT = 1.5 min; **4k**: RT = 0.6 min, NAC-adduct: RT = 0.58 min, m/z = 298.09 $[M\text{-}H]^-$.

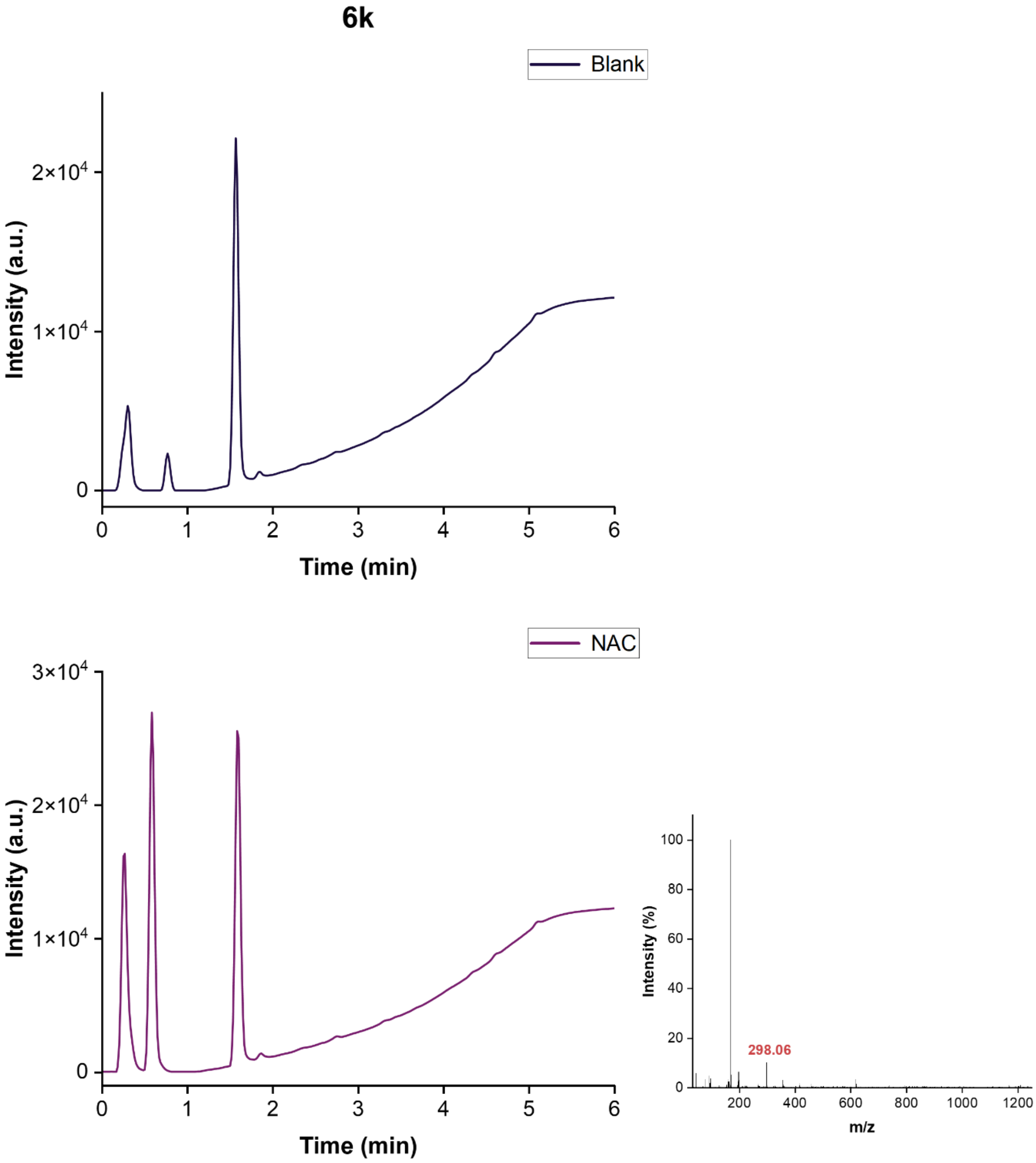

Figure S331. UV (sum of absorption at 230 nm and 254 nm) and MS spectra of the LC-MS analysis of 100 μM of **6k** incubated with 5 mM NAC for 16 h in PBS buffer pH 7.4 at room temperature. The blank sample is shown in black while the reaction in magenta. Internal standard: RT = 1.5 min; **6k**: RT = 0.77 min, NAC-adduct: RT = 0.58 min, *m/z* = 298.09 $[M-H]^-$.

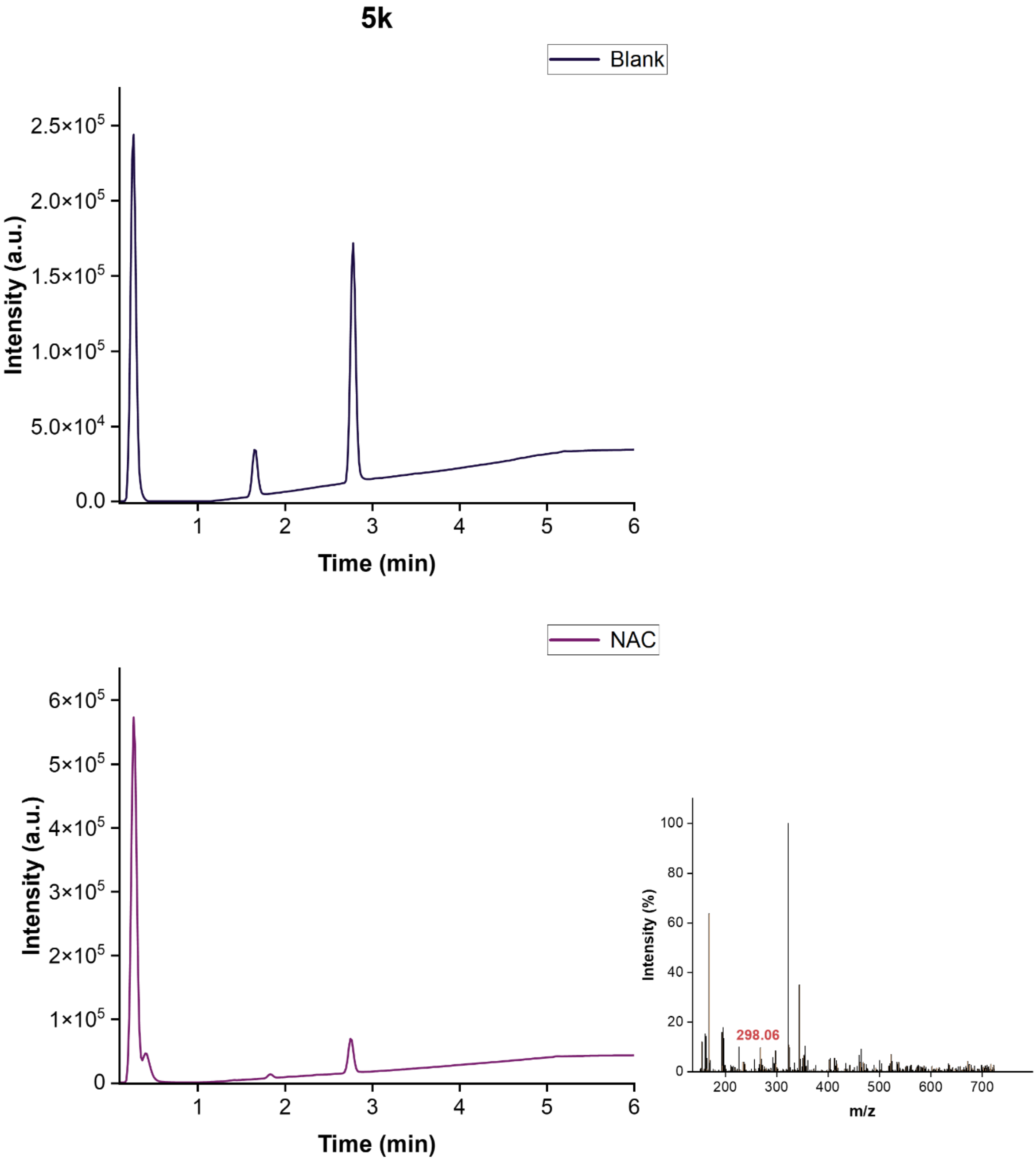

Figure S332. UV (sum of absorption at 230 nm and 254 nm) and MS spectra of the LC-MS analysis of 100 μM of **5k** incubated with 5 mM NAC for 16 h in PBS buffer pH 7.4 at room temperature. The blank sample is shown in black while the reaction in magenta. Internal standard: RT = 2.77 min; **5k**: RT = 0.6 min, NAC-adduct: RT = 0.58 min, *m/z* = 298.06 $[M-H]^-$.

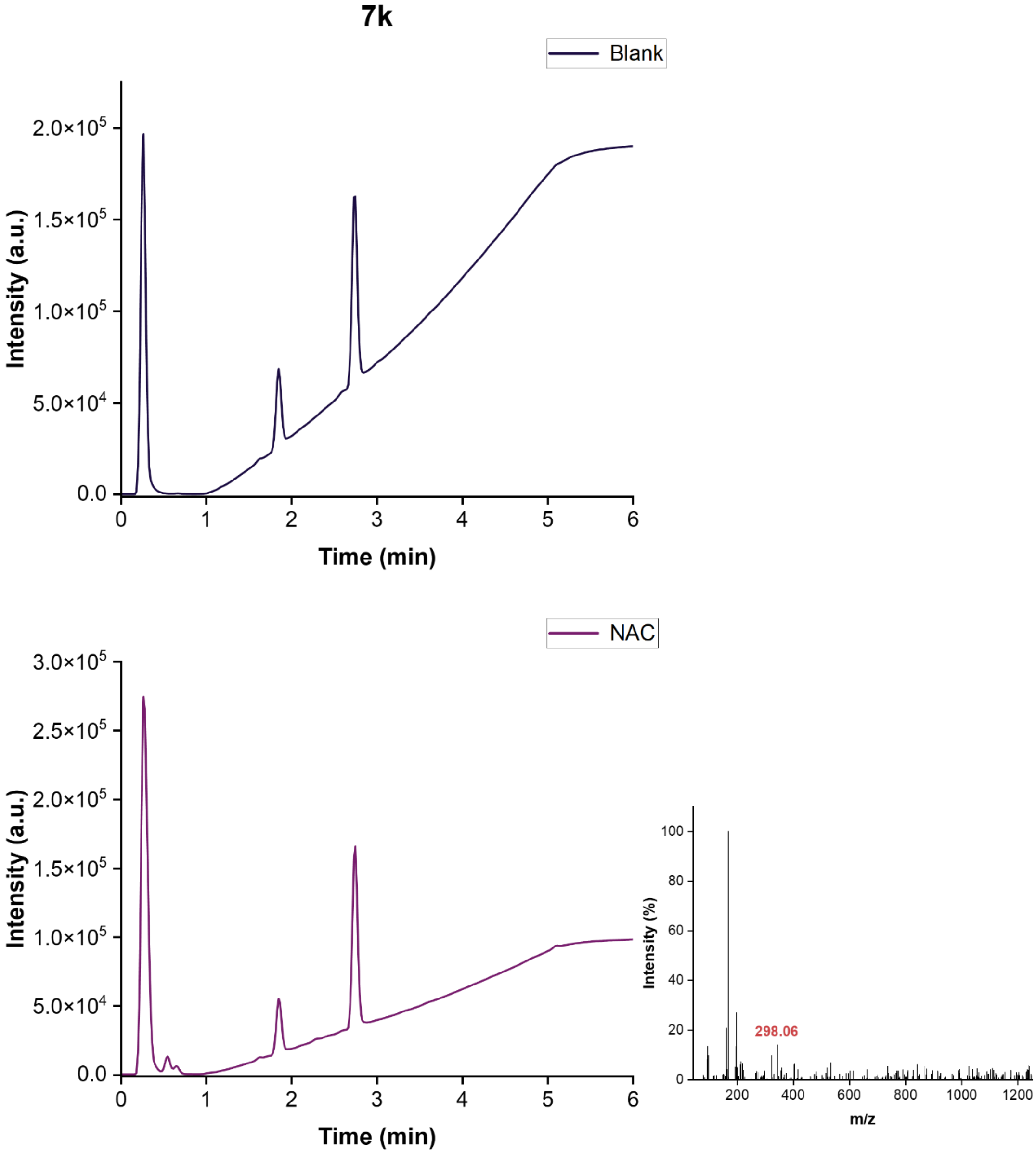

Figure S333. UV (sum of absorption at 230 nm and 254 nm) and MS spectra of the LC-MS analysis of 100 µM of **7k** incubated with 5 mM NAC for 16 h in PBS buffer pH 7.4 at room temperature. The blank sample is shown in black while the reaction in magenta. Internal standard: RT = 2.77 min; **7k**: RT = 1.85 min, NAC-adduct: RT = 0.58 min, *m/z* = 298.06 $[M-H]^-$.

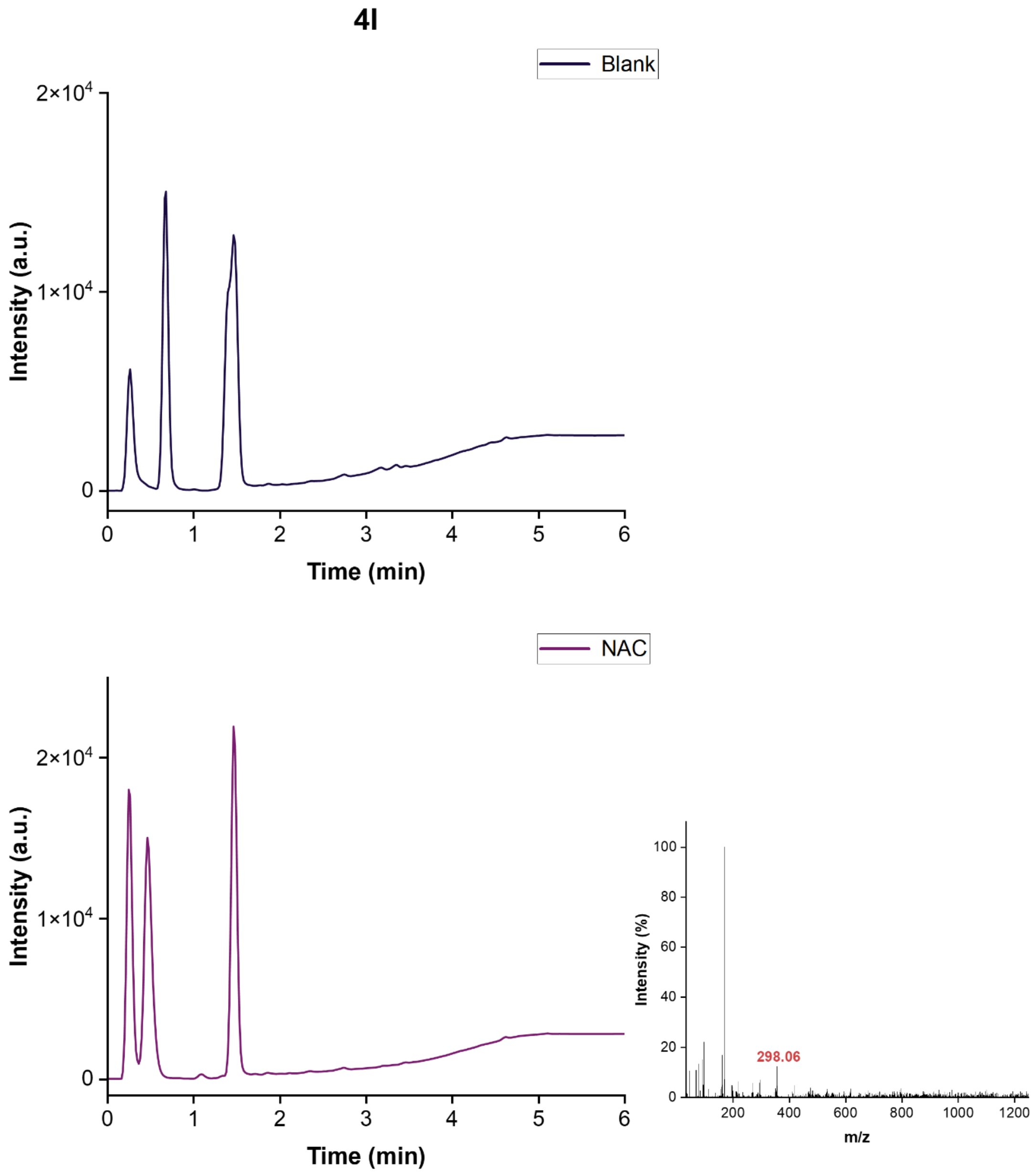

Figure S334. UV (sum of absorption at 230 nm and 254 nm) and MS spectra of the LC-MS analysis of 100 μM of **4l** incubated with 5 mM NAC for 16 h in PBS buffer pH 7.4 at room temperature. The blank sample is shown in black while the reaction in magenta. Internal standard: RT = 1.5 min; **4l**: RT = 0.68 min, NAC-adduct: RT = 0.47min, *m/z* = 298.06 $[M-H]^-$.

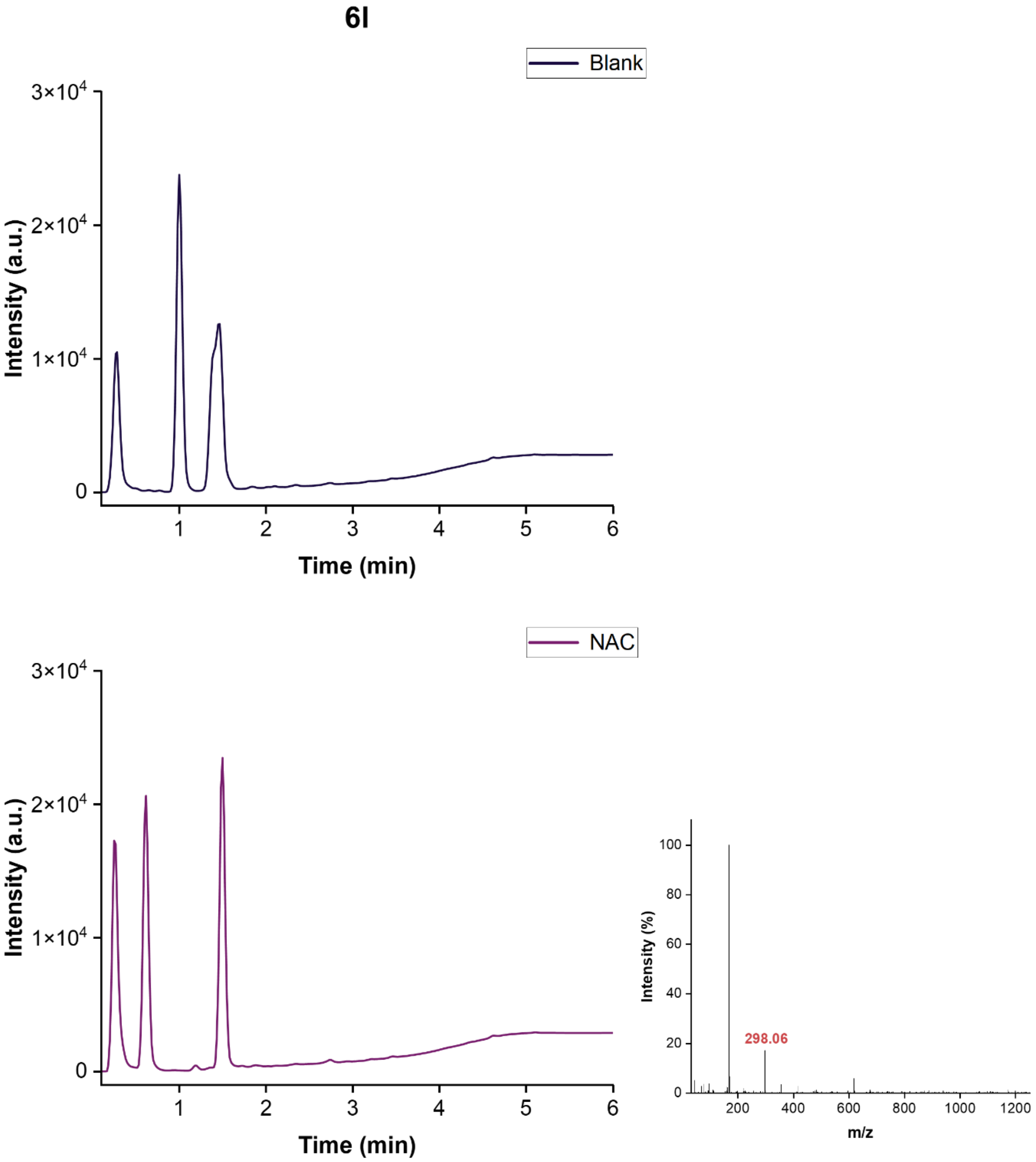

Figure S335. UV (sum of absorption at 230 nm and 254 nm) and MS spectra of the LC-MS analysis of 100 µM of **6l** incubated with 5 mM NAC for 16 h in PBS buffer pH 7.4 at room temperature. The blank sample is shown in black while the reaction in magenta. Internal standard: RT = 1.5 min; **6l**: RT = 1 min, NAC-adduct: RT = 0.6 min, *m/z* = 298.06 $[M-H]^-$.

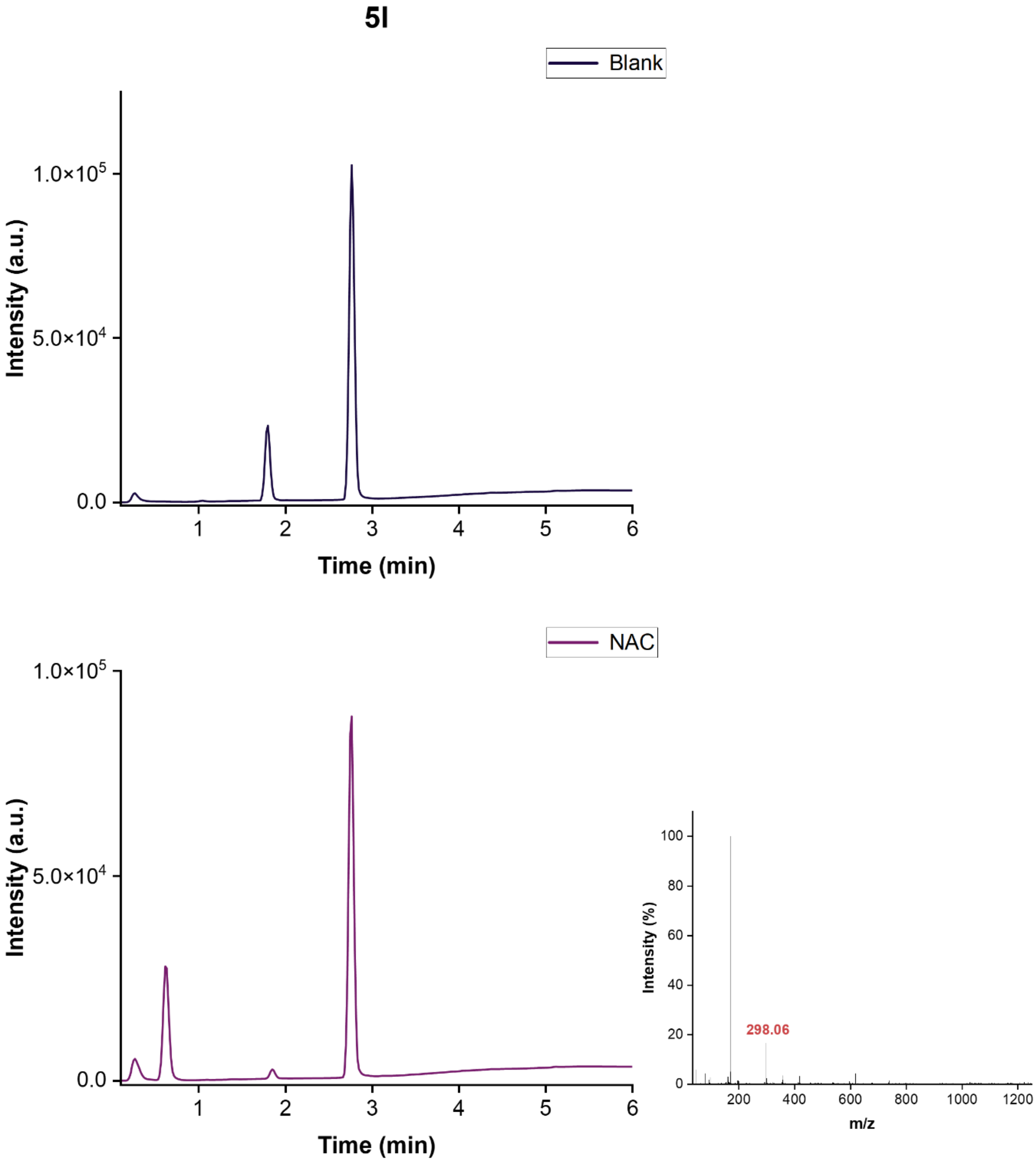

Figure S336. UV (sum of absorption at 230 nm and 254 nm) and MS spectra of the LC-MS analysis of 100 μM of **5l** incubated with 5 mM NAC for 16 h in PBS buffer pH 7.4 at room temperature. The blank sample is shown in black while the reaction in magenta. Internal standard: RT = 2.77 min; **5l**: RT = 1.98 min, NAC-adduct: RT = 0.55 min, *m/z* = 298.06 $[M-H]^-$.

7l

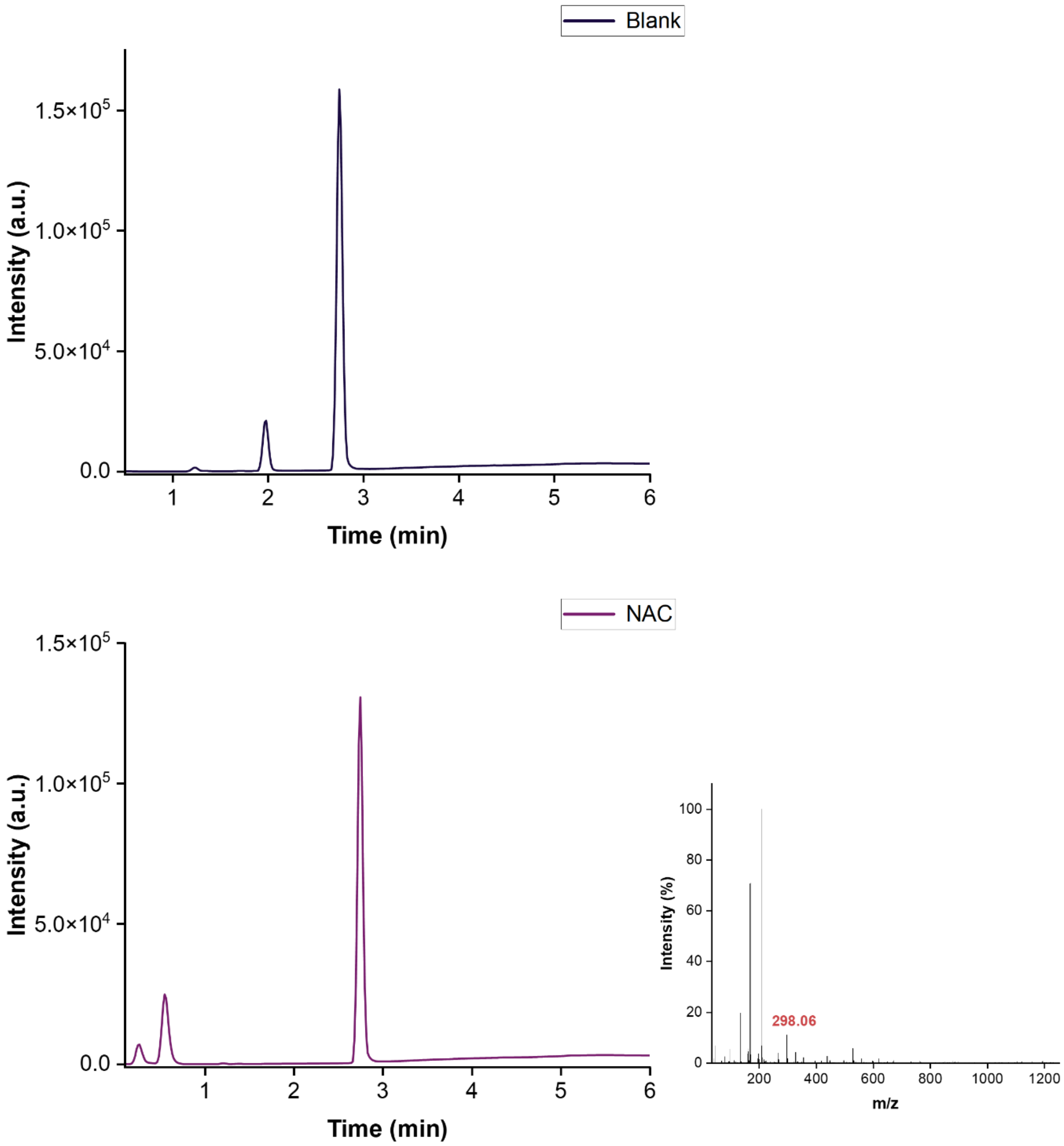

Figure S337. UV (sum of absorption at 230 nm and 254 nm) and MS spectra of the LC-MS analysis of 100 μM of **7l** incubated with 5 mM NAC for 16 h in PBS buffer pH 7.4 at room temperature. The blank sample is shown in black while the reaction in magenta. Internal standard: RT = 2.77 min; **7l**: RT = 1.98 min, NAC-adduct: RT = 0.55 min, *m/z* = 298.06 $[M-H]^-$.

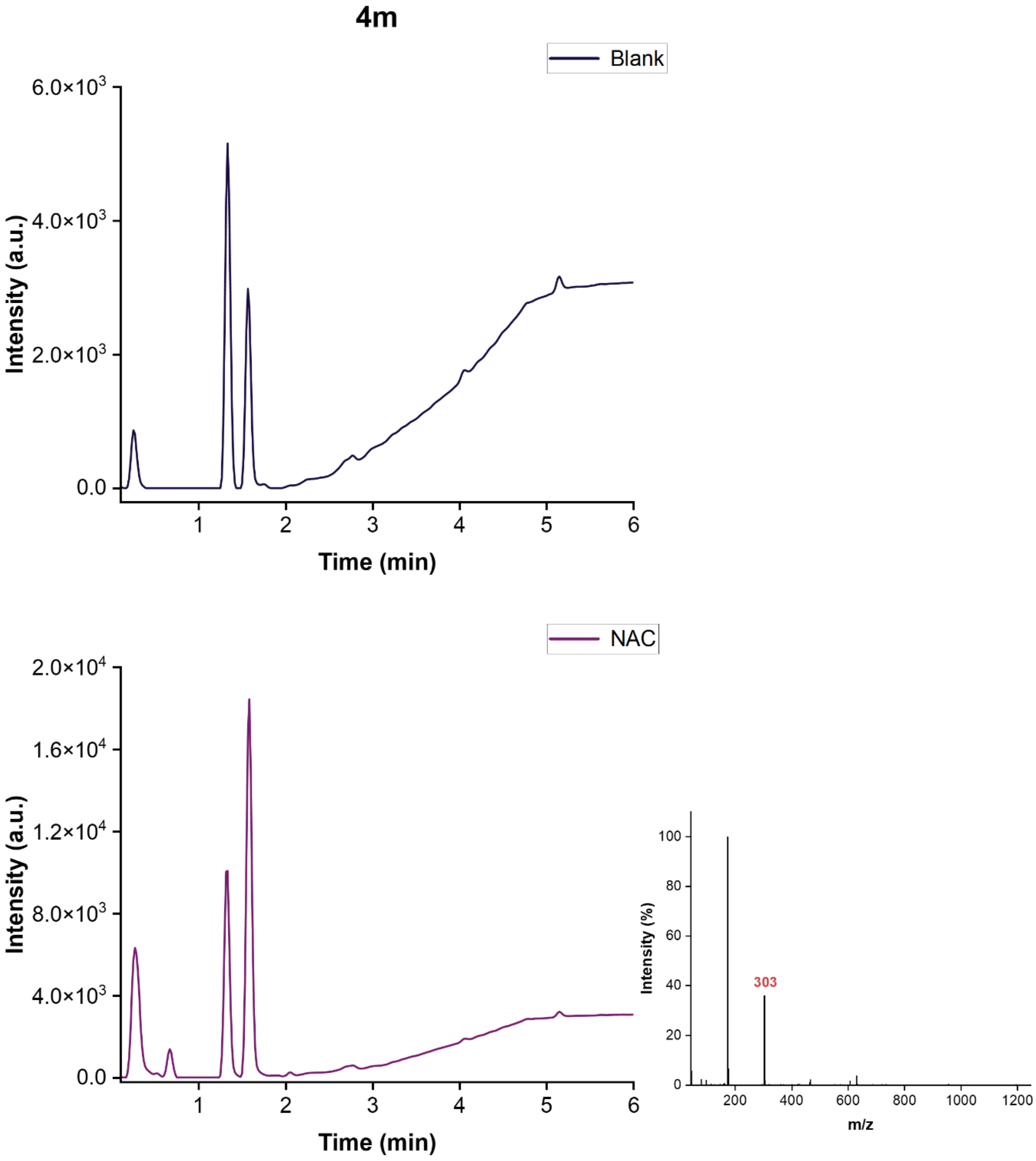

Figure S338. UV (sum of absorption at 230 nm and 254 nm) and MS spectra of the LC-MS analysis of 100 μM of **4m** incubated with 5 mM NAC for 16 h in PBS buffer pH 7.4 at room temperature. The blank sample is shown in black while the reaction in magenta. Internal standard: RT = 1.5 min; **4m**: RT = 1.33 min, NAC-adduct: RT = 1.33 min, *m/z* = 303 $[M-H]^-$.

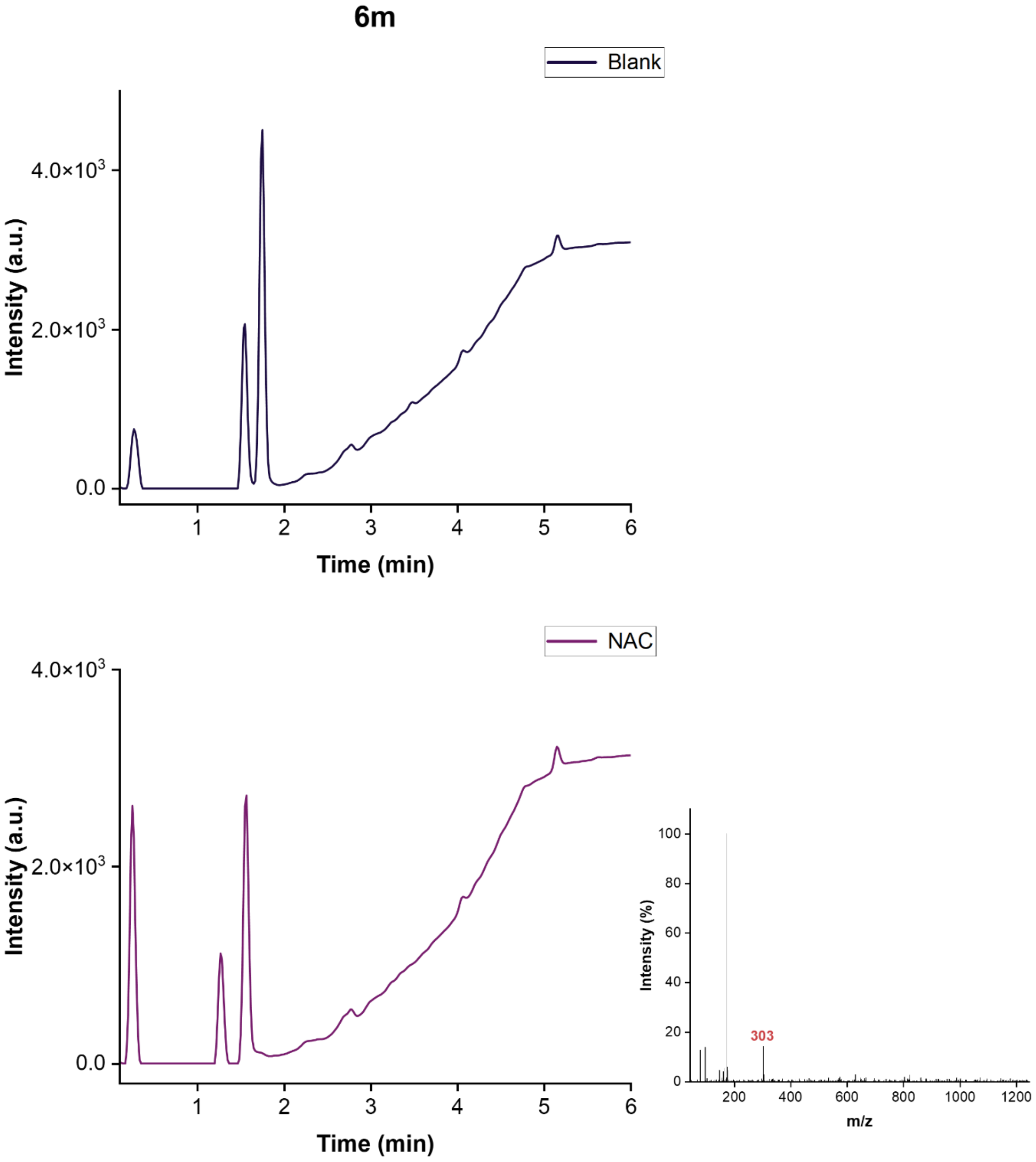

Figure S339. UV (sum of absorption at 230 nm and 254 nm) and MS spectra of the LC-MS analysis of 100 µM of **6m** incubated with 5 mM NAC for 16 h in PBS buffer pH 7.4 at room temperature. The blank sample is shown in black while the reaction in magenta. Internal standard: RT = 1.5 min; **6m**: RT = 1.75 min, NAC-adduct: RT = 1.33 min, *m/z* = 303 $[M\text{-}H]^-$.

5m

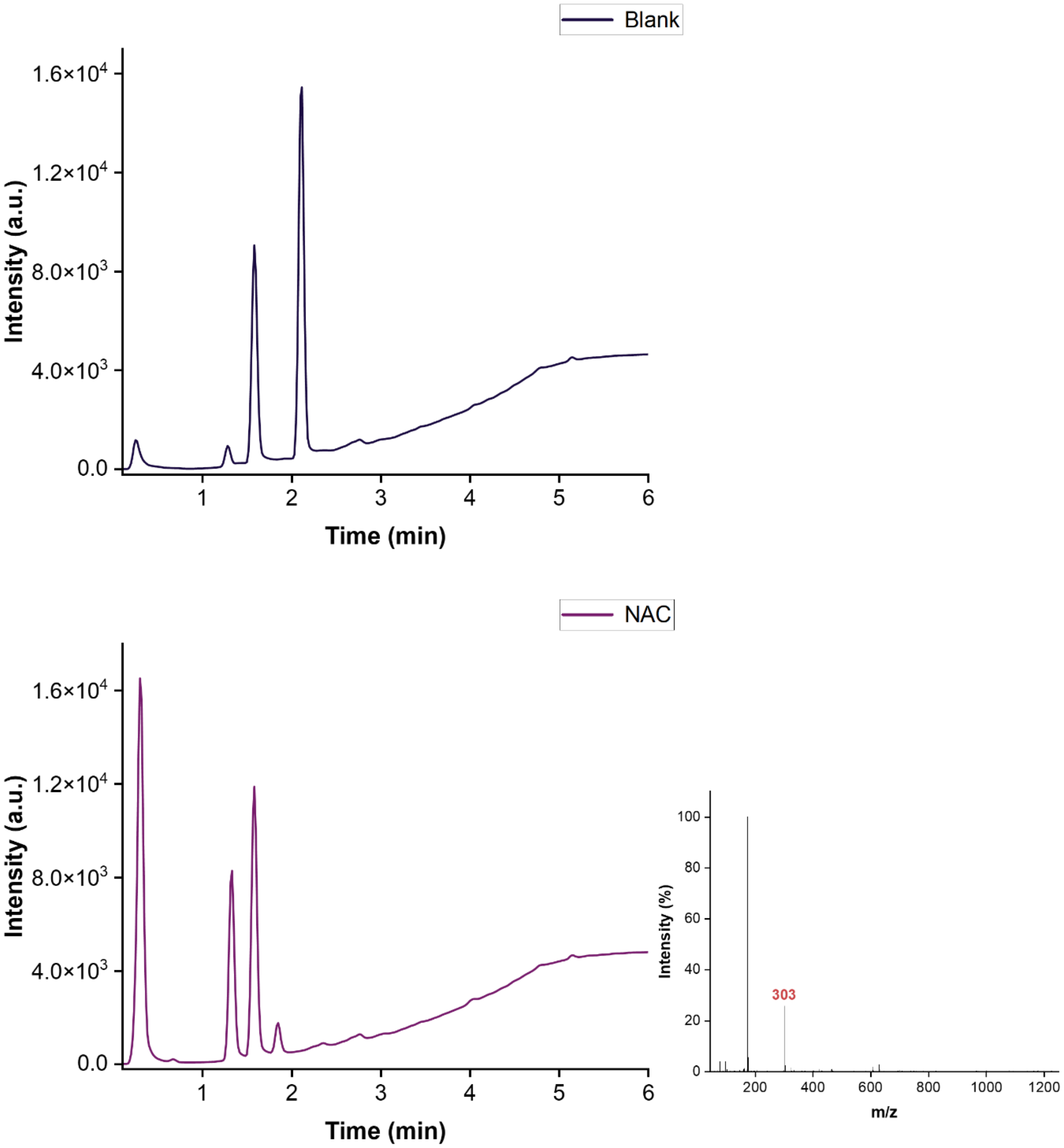

Figure S340. UV (sum of absorption at 230 nm and 254 nm) and MS spectra of the LC-MS analysis of 100 μM of **5m** incubated with 5 mM NAC for 16 h in PBS buffer pH 7.4 at room temperature. The blank sample is shown in black while the reaction in magenta. Internal standard: RT = 1.5 min; **5m**: RT = 2.12 min, NAC-adduct: RT = 1.33 min, *m/z* = 303 $[M\text{-}H]^-$.

7m

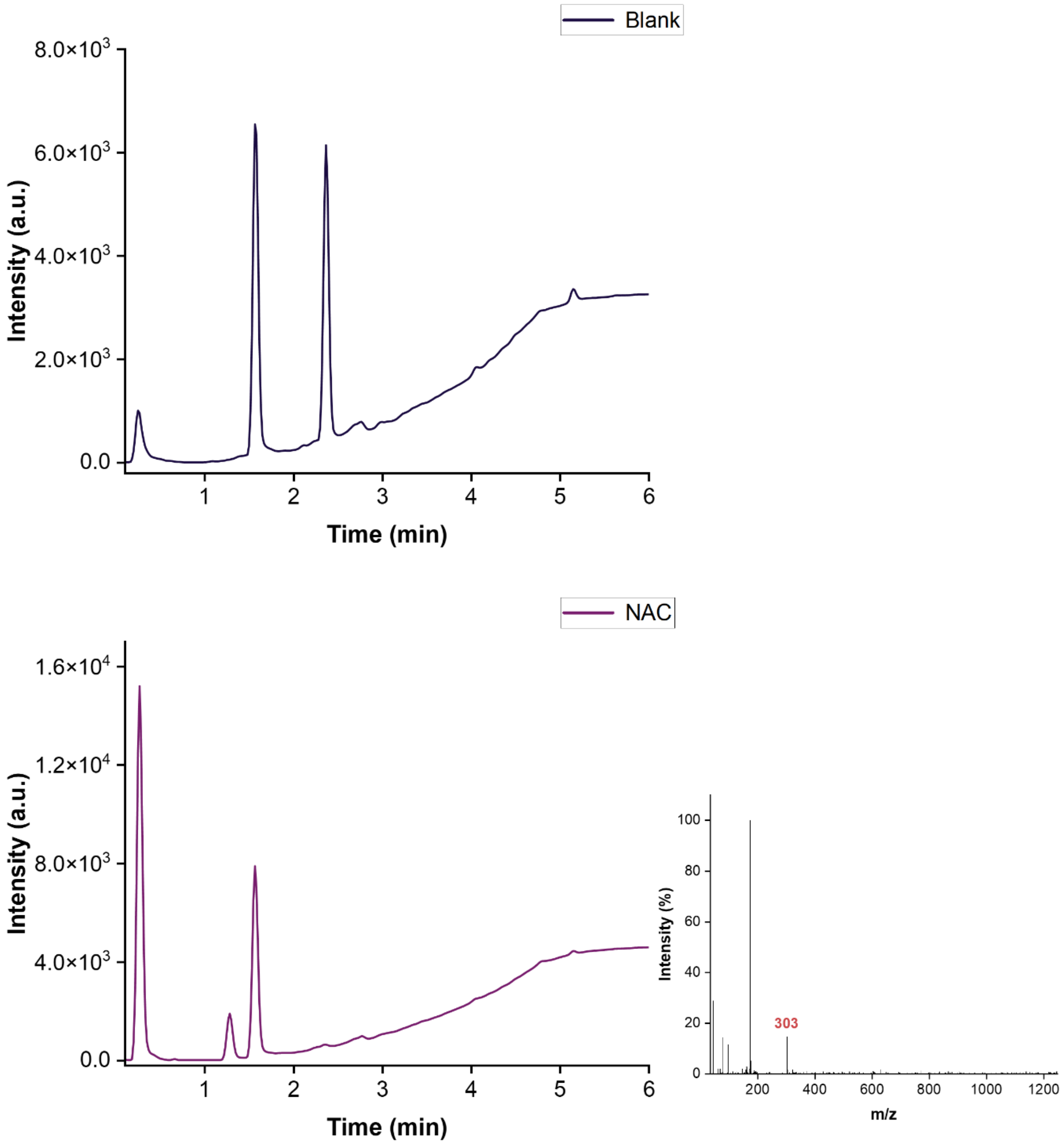

Figure S341. UV (sum of absorption at 230 nm and 254 nm) and MS spectra of the LC-MS analysis of 100 µM of **7m** incubated with 5 mM NAC for 16 h in PBS buffer pH 7.4 at room temperature. The blank sample is shown in black while the reaction in magenta. Internal standard: RT = 1.5 min; **7m**: RT = 2.37 min, NAC-adduct: RT = 1.33 min, *m/z* = 303 $[M-H]^-$.

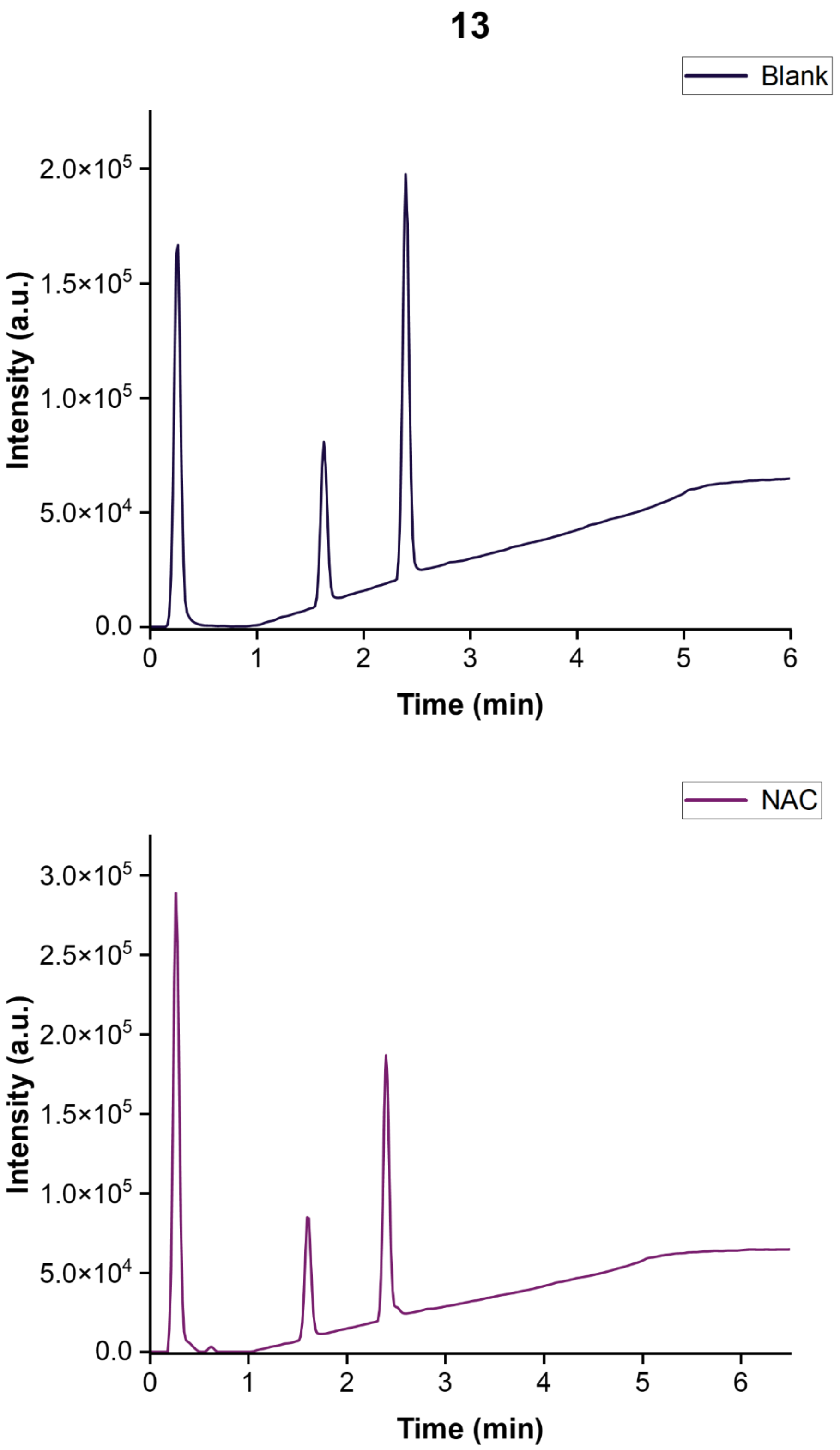

Figure S342. UV spectra (sum of absorption at 230 nm and 254 nm) of the LC-MS analysis of 100 µM of **13** incubated with 5 mM NAC for 16 h in PBS buffer pH 7.4 at room temperature. The blank sample is shown in black while the reaction in magenta. Internal standard: RT = 1.5 min; **13**: RT = 2.41 min.

14

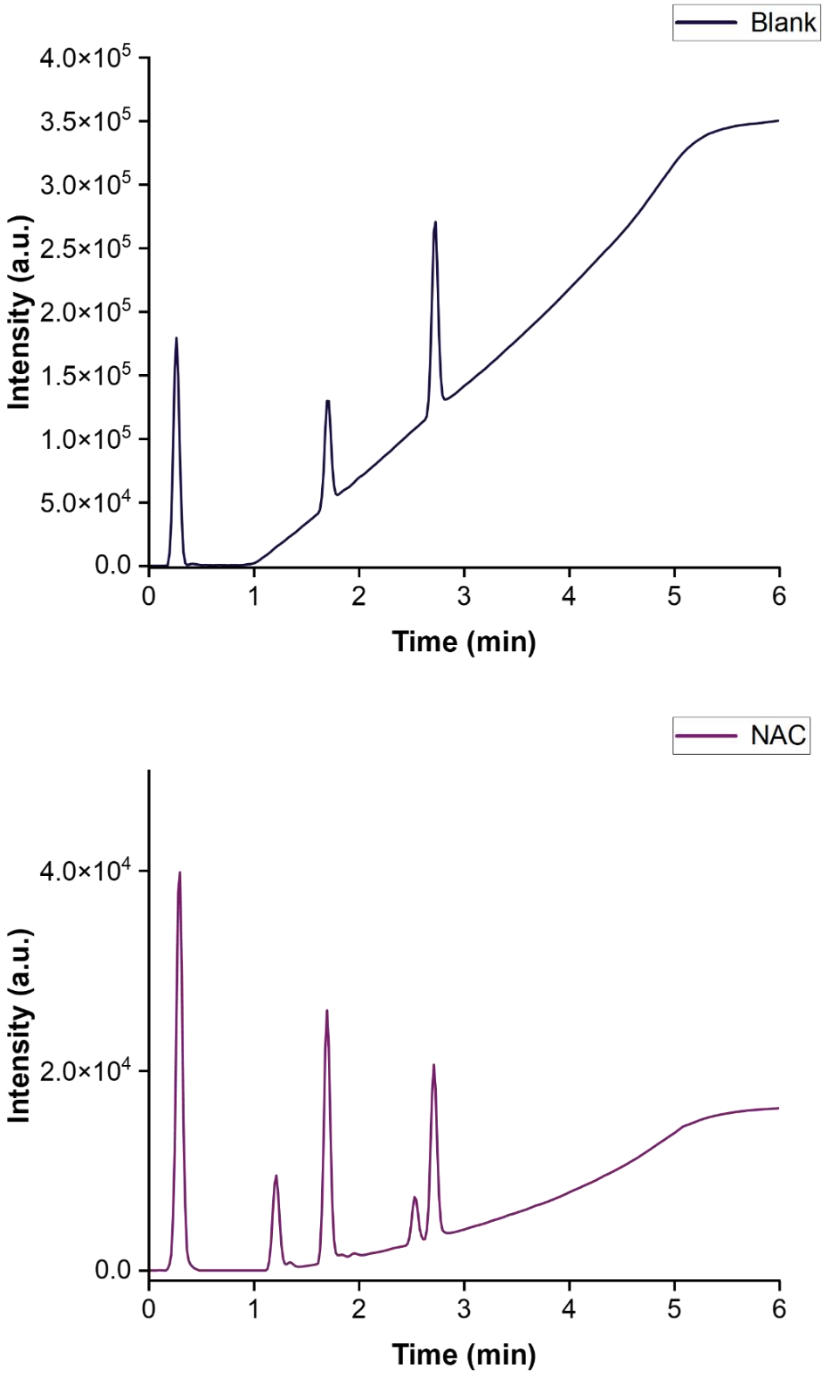

Figure S343. UV spectra (sum of absorption at 230 nm and 254 nm) of the LC-MS analysis of 100 µM of **14** incubated with 5 mM NAC for 16 h in PBS buffer pH 7.4 at room temperature. The blank sample is shown in black while the reaction in magenta. Internal standard: RT = 1.5 min; **14**: RT = 2.74 min.

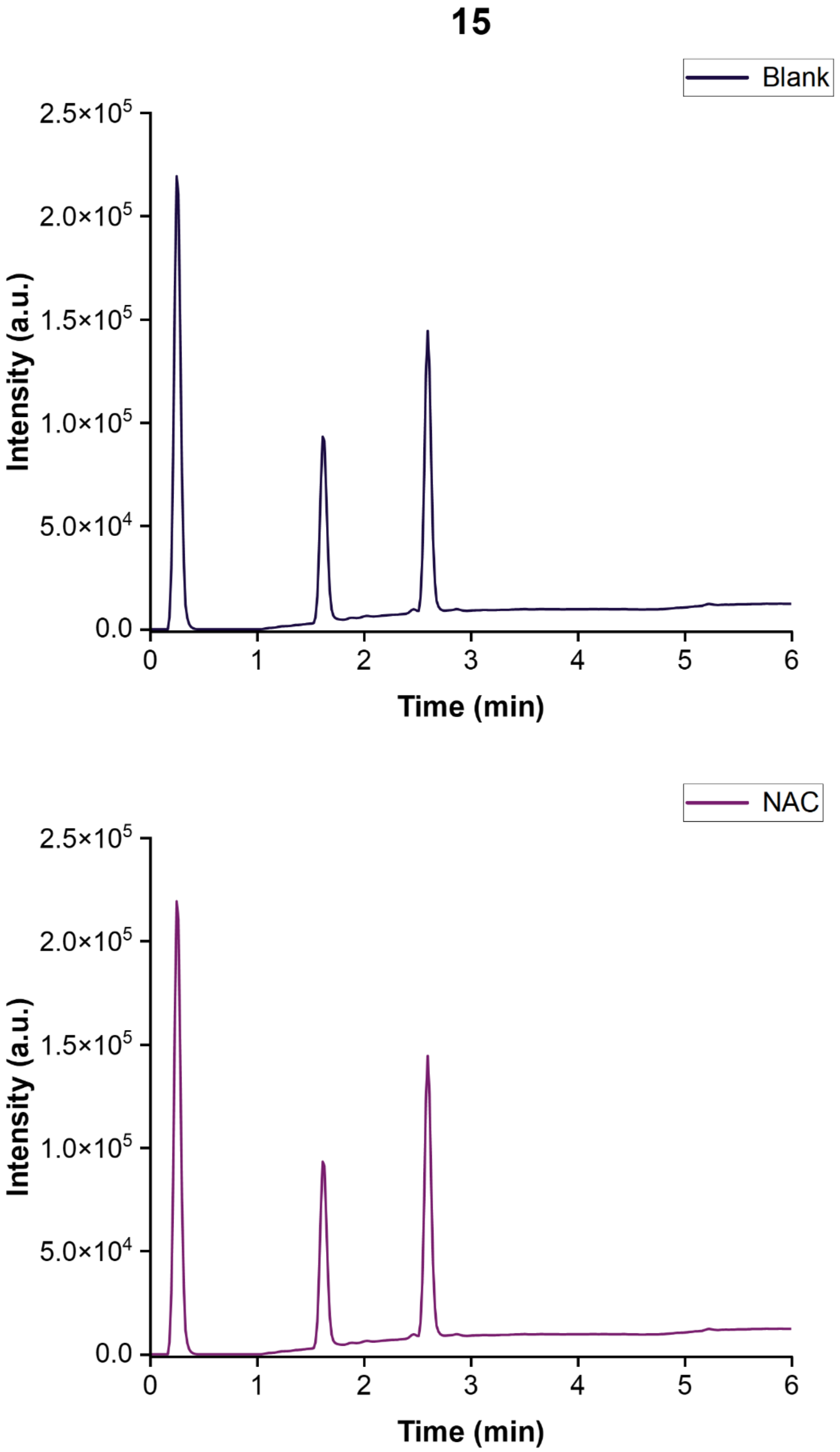

Figure S344. UV spectra (sum of absorption at 230 nm and 254 nm) of the LC-MS analysis of 100 µM of **15** incubated with 5 mM NAC for 16 h in PBS buffer pH 7.4 at room temperature. The blank sample is shown in black while the reaction in magenta. Internal standard: RT = 1.5 min; **15**: RT = 2.34 min.

16

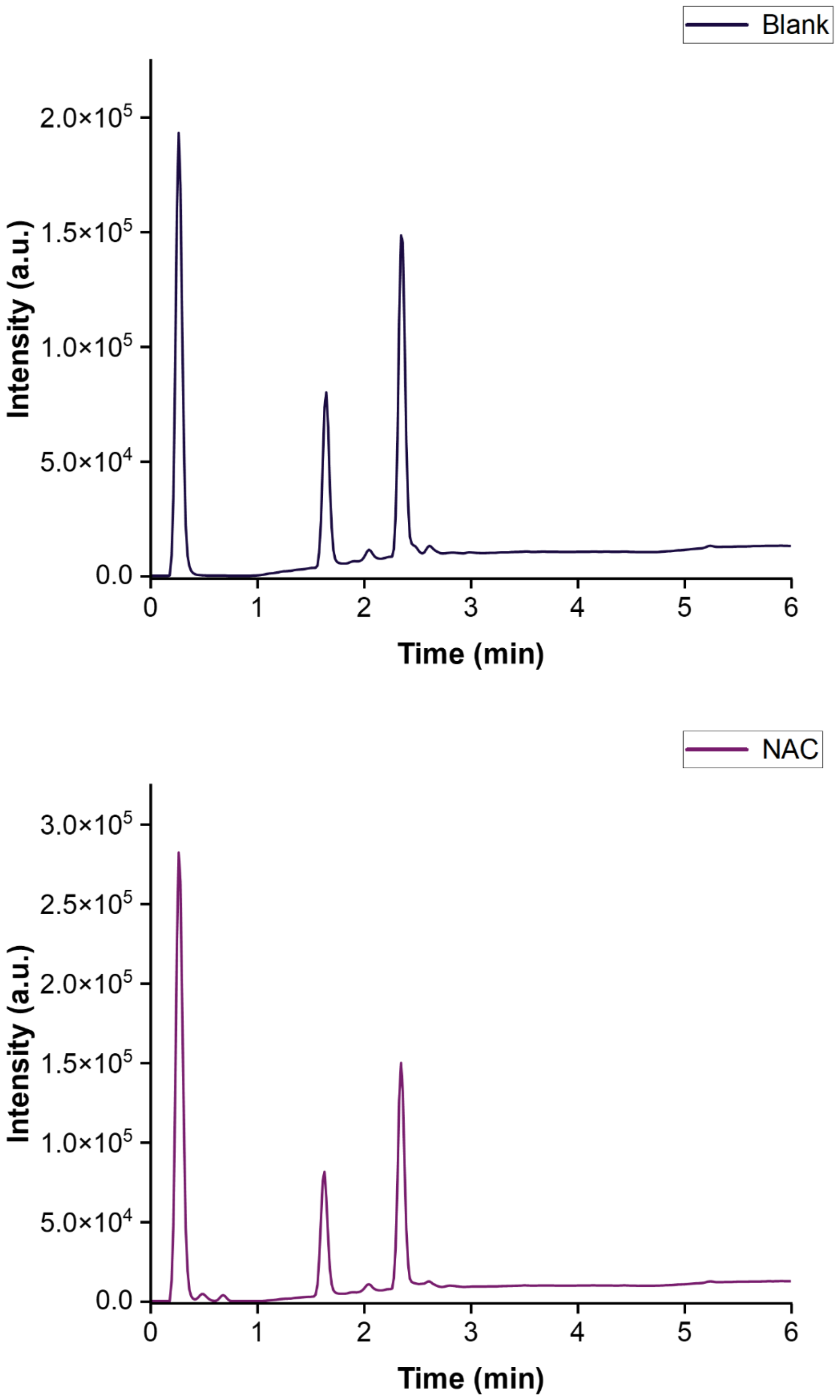

Figure S345. UV spectra (sum of absorption at 230 nm and 254 nm) of the LC-MS analysis of 100 µM of **16** incubated with 5 mM NAC for 16 h in PBS buffer pH 7.4 at room temperature. The blank sample is shown in black while the reaction in magenta. Internal standard: RT = 1.5 min; **16**: RT = 2.6 min.

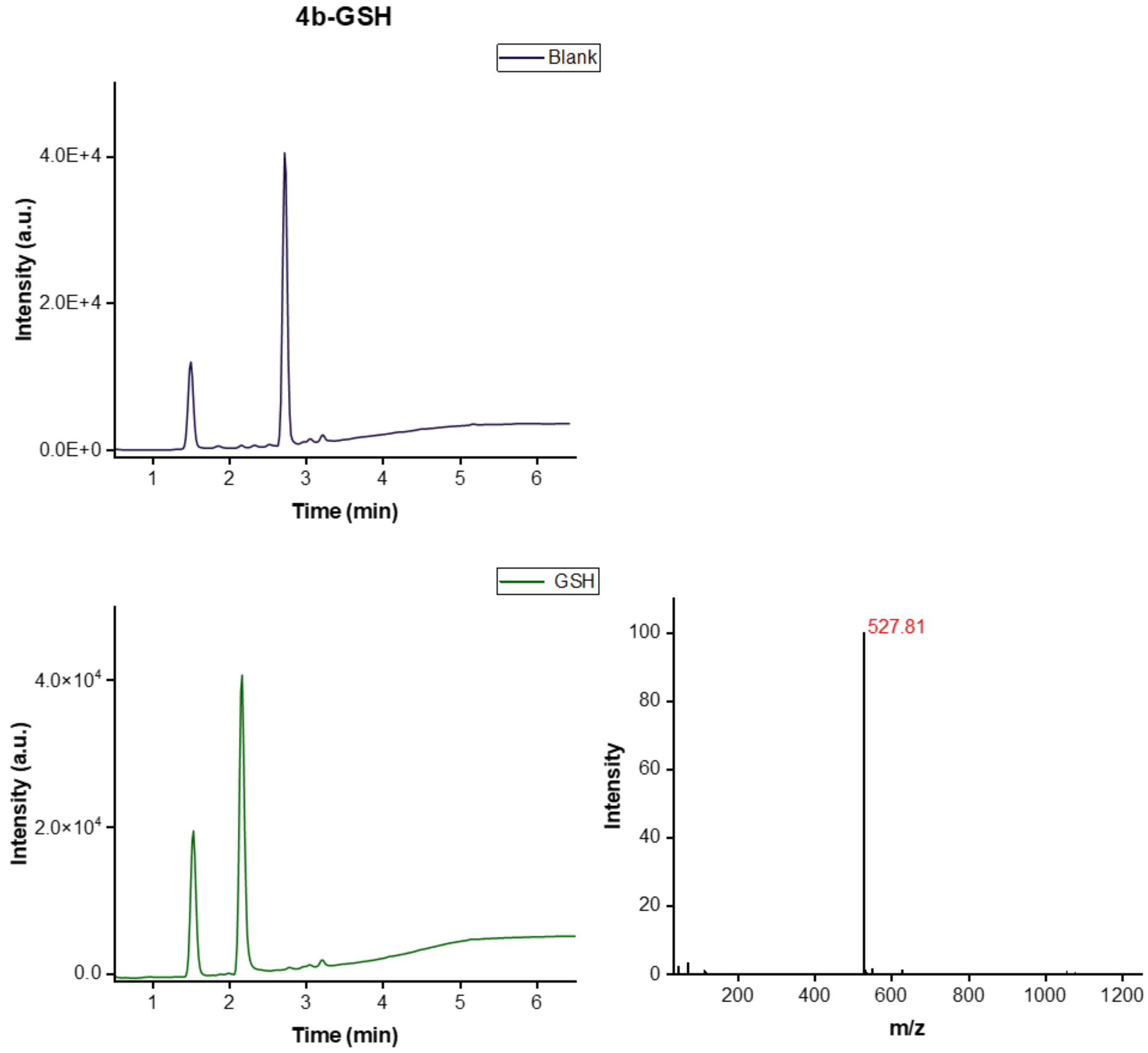

Figure S346. UV spectra (sum of absorption at 230 nm and 254 nm) of the LC-MS analysis of 100 µM of **4b** incubated with 5 mM GSH for 16 h in PBS buffer pH 7.4 at room temperature. The blank sample is shown in black while the reaction in green. Internal standard: RT = 1.5 min; **4b**: RT = 2.7 min; GSH-adduct: RT = 2.2 min, *m/z* = 527.81 $[M\text{-}H]^-$

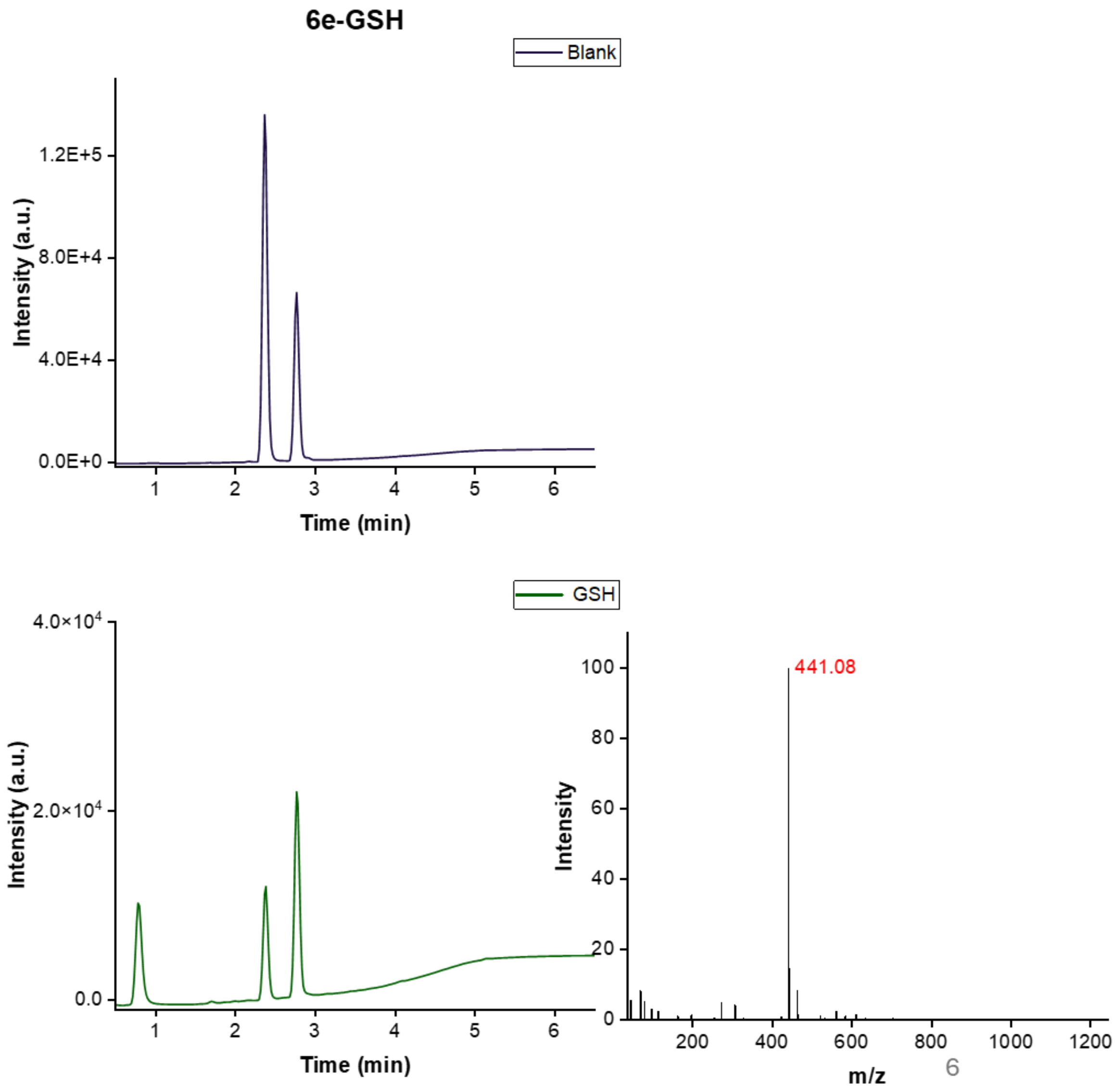

Figure S347. UV spectra (sum of absorption at 230 nm and 254 nm) of the LC-MS analysis of 100 µM of **6e** incubated with 5 mM GSH for 16 h in PBS buffer pH 7.4 at room temperature. The blank sample is shown in black while the reaction in green. Internal standard: RT = 2.7 min; **6e**: RT = 2.5 min; GSH-adduct: RT = 0.9 min, *m/z* = 441.08 $[M-H]^-$

7 HPLC Traces of Probes

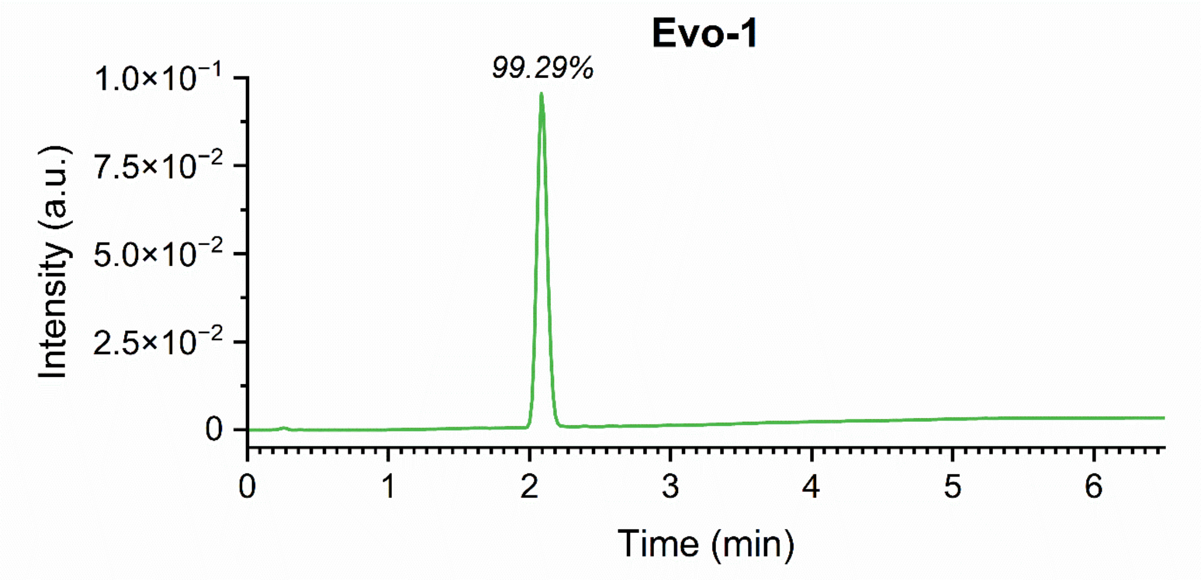

Figure S348. UV spectrum (sum of absorption at 254 nm and 360 nm) of the HPLC analysis of **Evo-1**. a.u. = absorbance units.

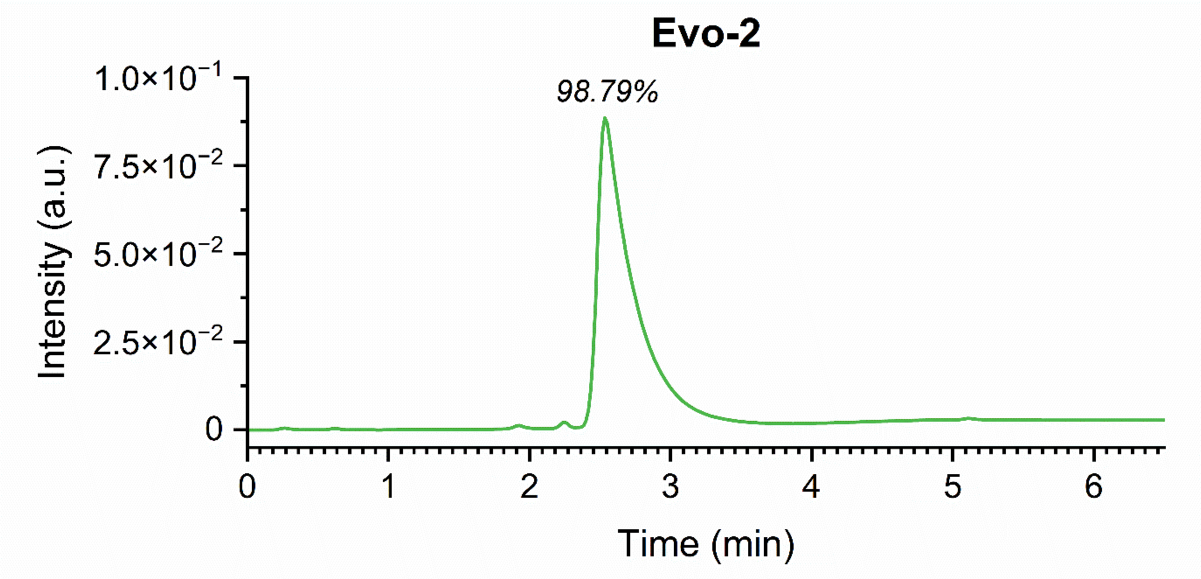

Figure S349. UV spectrum (sum of absorption at 254 nm and 360 nm) of the HPLC analysis of **Evo-2**. a.u. = absorbance units.

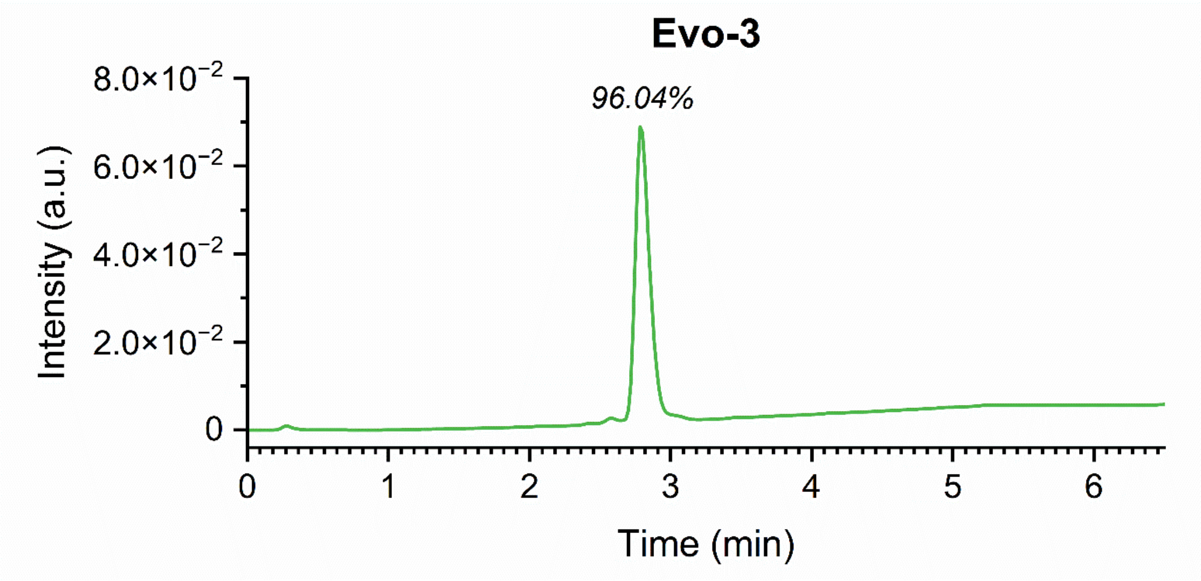

Figure S350. UV spectrum (sum of absorption at 254 nm and 360 nm) of the HPLC analysis of **Evo-3**. a.u. = absorbance units.

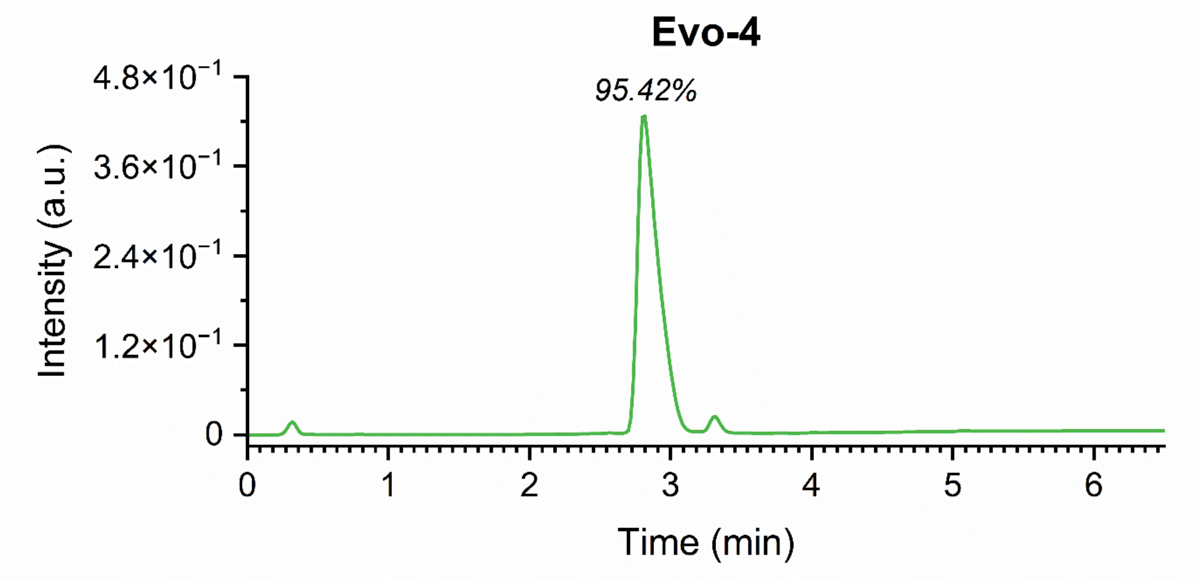

Figure S351. UV spectrum (sum of absorption at 254 nm and 360 nm) of the HPLC analysis of **Evo-4**. a.u. = absorbance units.

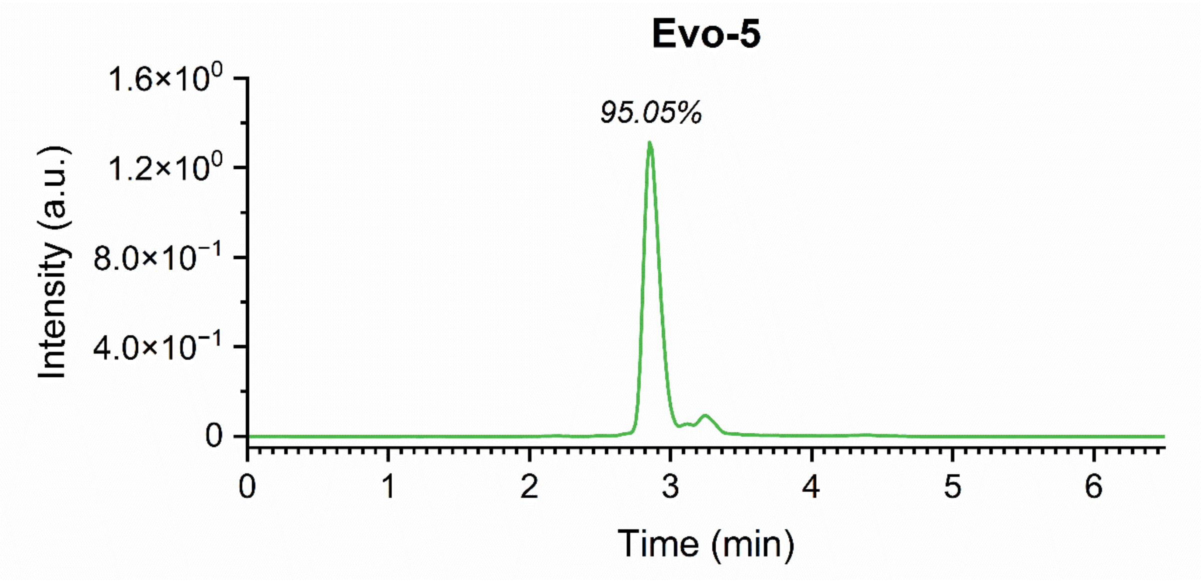

Figure S352. UV spectrum (sum of absorption at 500 nm and 600 nm) of the HPLC analysis of **Evo-5**. a.u. = absorbance units.

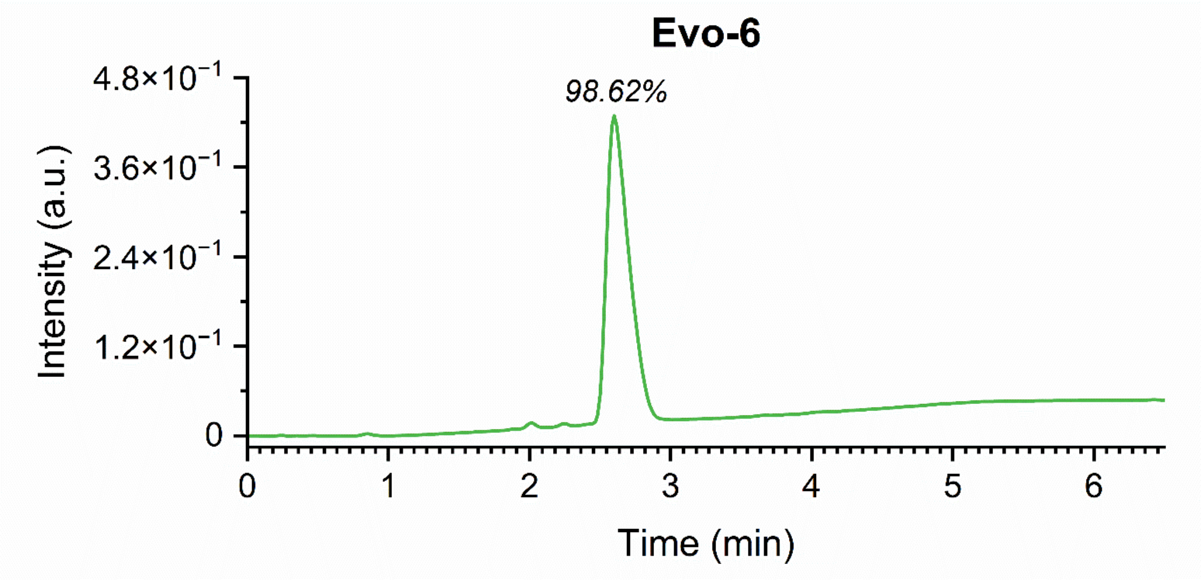

Figure S353. UV spectrum (sum of absorption at 230 nm and 254 nm) of the HPLC analysis of **Evo-6**. a.u. = absorbance units.

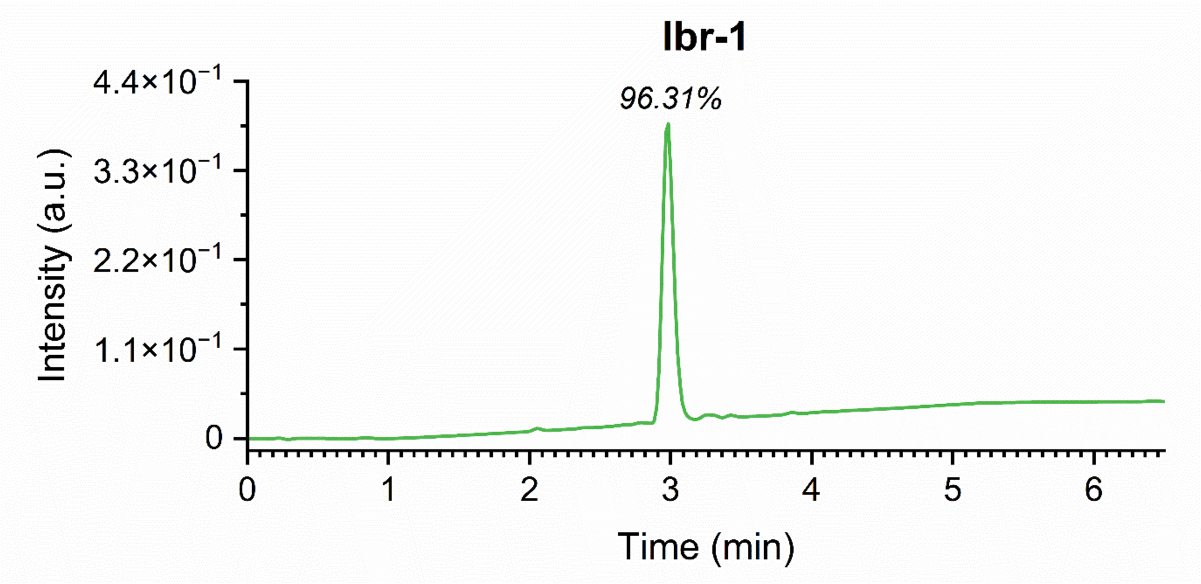

Figure S354. UV spectrum (sum of absorption at 230 nm and 254 nm) of the HPLC analysis of **Ibr-1**. a.u. = absorbance units.

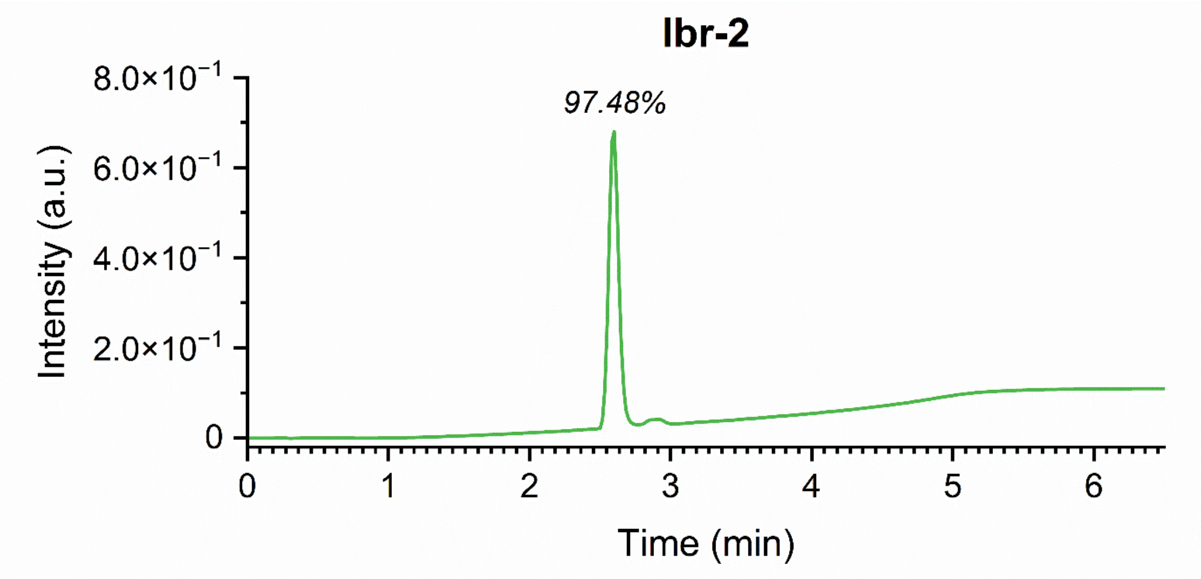

Figure S355. UV spectrum (sum of absorption at 254 nm and 360 nm) of the HPLC analysis of **Ibr-2**. a.u. = absorbance units.
